# Supplementary material for: High-throughput functional annotation of natural products by integrated activity profiling
Source: Proc Natl Acad Sci U S A. 2022 Nov 30;119(49):e2208458119. doi: 10.1073/pnas.2208458119 (PMC9894231; doi:10.1073/pnas.2208458119)
Supplement: Supplementary file 1 — Appendix 01 (PDF) [file pnas.2208458119.sapp.pdf]

PNAS
www.pnas.org

Supplementary Information for

High-Throughput Functional Annotation of Natural Products by Integrated Activity Profiling

Suzie K. Hight[1,8], Trevor N. Clark[2], Kenji L. Kurita[2,6], Elizabeth A. McMillan[1], Walter Bray[3], Anam F. Shaikh[4], Aswad Khadilkar[3], F. P. Jake Haeckl[2], Fausto Carnevale-Neto[2], Scott La[3], Akshar Lohith[3], Rachel M. Vaden[1], Jeon Lee[5], Shuguang Wei[4], R. Scott Lokey[3], Michael A. White[1,7], Roger G. Linington[2*], John B. MacMillan[3,4*]*

*Corresponding authors: Michael White; Roger Linington; John Macmillan

Email: Michael White mikewhite3224@gmail.com; Roger Linington rliningt@sfu.ca; John Macmillan jomacmil@ucsc.edu

This PDF file includes:

Supplementary text

Figures S1 to S33 and legends

Tables S1 to S6

SI References

Other supplementary materials for this manuscript include the following:

Dataset S1: List of all Selleck compounds included in the reference library and their target class annotations.

Dataset S2: Raw gene expression data for parkamycin A.

SUPPORTING INFORMATION

Contents

Supplementary Note 1: Chemical Libraries 3
Supplementary Note 2: Metabolomics 6
Supplementary Note 3: Bioassays 8
Supplementary Note 4: Statistical analysis 12
Supplementary Note 5: Compound isolation 14
Supplementary Note 6: Parkamycins A/B isolation, structure elucidation, and NMR table 16
Supplementary Figure 1: Selleck library target class membership. 21
Supplementary Figure 2: Pearson correlation distributions in FUSION and CP. 22
Supplementary Figure 3: Venn diagrams illustrating overlap among target classes identified as significantly enriched in a k-means cluster. 23
Supplementary Figure 4: Annotated heatmaps showing which target classes are significantly enriched in k-means clusters. 24
Supplementary Figure 5. Alluvial diagrams for each target class using k-means clustering of the FUSION and CP datasets. 25
Supplementary Figure 6. Alluvial diagrams for each cluster in FUSION and each cluster in CP. 55
Supplementary Figure 7: Adapted similarity network fusion workflow. 115
Supplementary Figure 8: CDF plots comparing pairwise associations between in-class and out-of-class associations 116
Supplementary Figure 9: “Dead” Compounds in CP and FUSION cluster together in the SNF-Euclidean APC map 190
Supplementary Figure 10. Affinity propagation clustering map of the SNF-pearson network 191
Supplementary Figure 11: Bokeh Server demonstration of functionality. 194
Supplementary Figure 12. Density distributions of SNF scores for unique metabolites in the natural product fraction library. 195
Supplementary Figure 13: Compound Activity map for combined SNF profiles and untargeted metabolomics features. 196
Supplementary Figure 14: Signatures from natural product fractions containing trichostatin A. 197

Supplementary Figure 15. Signatures from natural product fractions containing surugamide. 198
Supplementary Figure 16. Signatures from natural product fractions containing parkamycin A. 199
Supplementary Figure 17. ^{1}H NMR of parkamycin A. 200
Supplementary Figure 18. ^{13}C NMR of parkamycin A. 201
Supplementary Figure 19. HSQC NMR spectrum of parkamycin A. 202
HSQC NMR spectrum collected at 600 MHz in DMSO-d6. 202
Supplementary Figure 21. Expanded HSQC NMR spectrum of parkamycin A, region 2. 204
Supplementary Figure 22. COSY NMR spectrum of parkamycin A. 205
Supplementary Figure 23. HMBC NMR spectrum of parkamycin A. 206
Supplementary Figure 24. Expanded HMBC NMR spectrum of parkamycin A, region 1. 207
Supplementary Figure 25. Expanded HMBC NMR spectrum of parkamycin A, region 2. 208
Supplementary Figure 26. ^{15}N-HMBC NMR spectrum of parkamycin A. 209
Supplementary Figure 27. ^{1}H NMR spectrum of parkamycin B (semi pure). 210
Supplementary Figure 28. ^{13}C NMR spectrum of parkamycin B (semi pure). 211
Supplementary Figure 29. HSQC NMR spectrum of parkamycin B (semi pure). 212
Supplementary Figure 30. HMBC NMR spectrum of parkamycin B (semi pure). 213
Supplementary Figure 31. Volcano plot displaying differential gene expression in the Nanostring metabolism panel after treatment with RLUS-2088 (Parkamycin A). 214
Supplementary Figure 32. Metabolic pathway analysis on gene expression data collected after parkamycin treatment. 215
Supplementary Figure 33. Comparison of pathway scores for select pathways altered with RLUS2088 (Parkamycin-A) treatment compared to DMSO. 216
Supplementary Table 1. Selleck library target representation. 217
Supplementary Table 2: K-means cluster membership in FUSION and CP for compounds in the top 30 largest target classes. 222
Supplementary Table 3: SNF-Euclidean APC hypergeometric test p-values. 237
Supplementary Table 4: Target class enrichment in APC clusters. 240
Supplementary Table 5: Cluster #49 members. 241
Supplementary Table 6: Differential expression of top 20 genes in the Nanostring metabolism panel. 242

Supplementary Note 1: Chemical Libraries

Selleck Library

The reference library is a set of 2027 molecules spanning 196 compound classes, with 789 compounds not belonging to any annotated class. The library was purchased from Selleck as a premade library, combining compounds from the Bioactive, Kinase Inhibitor, and FDA-approved drug libraries. The compounds were formatted into two sets of 384 well plates at 10 mM and 2 mM.

Natural product libraries

Two natural product libraries were utilized in this study. The MacMillan lab collection used in this study was comprised of ~500 fractions derived from 25 marine-derived bacterial strains. The library of microbial natural product fractions was derived from marine-derived Actinomycetes (20), Firmicutes (5). These bacteria were cultivated from marine sediment samples collected in Tonga, the Gulf of Mexico (Texas, Louisiana), estuaries in South Carolina, and the Bahamas. A variety of techniques were utilized to isolate strains, including the use of nutrient-limited isolation media, such as those composed of only humic or fulvic acid, the use of small-molecule signaling compounds (N-acylhomoserine lactones, siderophores) that mimic the natural environment of the bacteria of interest, and isolation of spores using density gradient ultracentrifugation. Selection of bacterial isolates was carried out based on morphological appearance and followed up by phylogenetic characterization using 16S rRNA analysis using the Universal 16S rRNA primers FC27 and RC 1492 for the majority of the phylogenetic analysis. 16S rRNA sequences were compared to sequences in available databases using the Basic Local Alignment Search Tool.

To generate the fraction library used in this study, bacterial strains were fermented in 5 × 2.8 L Fernbach flasks each containing 1 L of a seawater based medium (10 g starch, 4 g yeast extract, 2 g peptone, 1 g $CaCO_3$, 40 mg $Fe_2(SO_4)_3 \cdot 4H_2O$, 100 mg KBr) and shaken at 200 rpm for seven days at 27 ºC. After seven days of cultivation, XAD7-HP resin (20 g/L) was added to adsorb the organic products, and the culture and resin were shaken at 200 rpm for 2 h. The resin was filtered through cheesecloth, washed with deionized water, and eluted with acetone to give a crude extract, with an average yield of 2.0 g of crude extract/strain. Further fractionation of the bacterial crude extracts (~500 mg) was accomplished using an Isco medium pressure automatic purification system (equipped with UV and ELSD detectors) using reversed phase C18 chromatography (gradient from 90:10 H_2O:CH_3CN to 0:100 H_2O:CH_3CN over 25 minutes, RediSep Rf Gold High Performance column with 600 mg capacity). Fermentation of each bacterial strain gives rise to a total of either 10 or 20 natural product fractions/strain. All natural product fractions in the library are standardized to a concentration of 10 mg/mL in DMSO. All fractions have been analyzed by low resolution LC/MS using an Agilent Model 6130 single quadrupole instrument.

Due to the nature of the fractionation approach, there is by design, the potential for sequential fractions to contain the same compounds. For example, if the peak for staurosporine is split between fraction 6 and 7, one could expect to have a similar biological signature between the sequential fractions.

The Linington natural product fraction library contains >5000 microbial fractions. The library is comprised of extracts of marine sediment-derived bacterial strains isolated by the Linington laboratory over the past 10 years. The library contains a cross section of Gram-positive genera, enriched in Actinobacterial strains. These sediments were collected by hand using SCUBA from over 70 discrete dive sites on the west coast of the United States and Canada. Collection sites include locations from the Channel Islands (near Los Angeles, CA) to the San Juan Islands in northern Washington state and sites on Vancouver Island, and are part of one of the largest systematic sampling campaigns for marine microbial chemistry performed in this area. Samples were collected with 15-mL centrifuge tubes and isolates were obtained from one of 8 selection media each supplemented with 50 mg of nalidixic acid and cycloheximide. Selective media listed below which end in "F" contain 1 L of MilliQ water and media ending in "S" contain 750 mL of 0.2 μm-filtered seawater, and 250 mL of MilliQ water to a total volume of 1 L. These media are as follows: actinomycete isolation agar (AIF and AIS, Difco™), SNF and SNS[1], NTF and NTS, and HVF and HVS[2]. Plates were incubated at room temperature until the appearance of desired colony morphologies consistent with actinobacteria. Colonies were picked which displayed characteristic actinobacterial morphologies and were subcultured onto either MB (37.4 g DifcoTM Marine Broth, 18 g agar, 1 L Milli-Q water) or A1 (18 g agar, 20 g starch, 10 g glucose, 5 g yeast extract, 5 g NZ-amine, 1 g $CaCO_3$, 50 mg nalidixic acid, 50 mg cycloheximide, 31.2 g Instant Ocean, 1 L Milli-Q water) agar plates.

Isolates were inoculated from MB (18 g agar, 37.4 g marine broth (Difco™), 1 L MilliQ water) or A1 (18 g agar, 20 g soluble starch, 10 g glucose, 5 g yeast extract, 5 g NZ-amine, 1 g $CaCO_3$, 1 L MilliQ water) agar plates into 7 mL of modified SYP (mSYP) liquid media (10 g starch, 4 g peptone, 2 g yeast extract, 1 L Milli-Q water, 31.2 g Instant Ocean® sea salt) in 25 x 150 mm glass culture tubes for 2-3 days at room temperature with shaking at 200 rpm. These small-scale cultures were shaken at 25°C and 200 rpm for a minimum of three days before moving to 60-mL medium-scales. Cultures were stepped up to medium-scale by inoculating 3 mL of the small-scale culture into 60 mL of freshly prepared mSYP in wide-mouthed 250-mL Erlenmeyer flasks with small springs. Medium-scale cultures were shaken at 25°C and 200 rpm for 3-7 days. Large scale cultures were prepared by inoculating 40 mL of medium-scale culture into 1 L of freshly prepared mSYP in 2.8-L Fernbach flasks with a large spring and 20 g of pre-washed Amberlite XAD-16 adsorbent resin (DCM, MeOH, and water; Sigma). Large-scale cultures were shaken at 25°C and 200 rpm for 7-10 days. At the end of the fermentation period, cells and resin were separated from the culture medium by vacuum filtration using a Whatman® glass microfiber filter and washed with deionized water. Resin and cells from each culture flask were extracted with 250 mL of 1:1 DCM/MeOH. The organic

extract was separated from the cells and resin by vacuum filtration and concentrated *in vacuo*.

Crude organic extracts from marine-derived actinobacteria were subjected to solid phase extraction using a Supelco-Discovery C18 cartridge (5 g) and eluted using a MeOH/H_2O step gradient (40 mL; 10% MeOH, 20% MeOH, 40% MeOH, 60% MeOH, 80% MeOH, 100% MeOH, 100% EtOAc) to afford seven fractions. The 10% MeOH fraction was discarded and the remaining six (fractions A – F) concentrated to dryness in vacuo. Dry pre-fractions were resolubilized in 1 mL of dimethyl sulfoxide (DMSO) and transferred to deep-well 96-well plates for long-term storage at -70°C.

For biological analysis, DMSO stocks of library fractions were thawed, sonicated, and reformatted to 384-well format for high-throughput screening.

For mass spectrometry analysis, DMSO stocks of library fractions were thawed, sonicated, and diluted 1:20,000 (5:200 DMSO, 10:240 50% MeOH/H_2O, 10:200 50% MeOH/H_2O) with mixing in 96-well format. Plates were reformatted into 384-well format, sealed, centrifuged at 1000 rpm for 30s, and analyzed.

Supplementary Note 2: Metabolomics

Data-Independent (DIA) UPLC-MS/MS Data Acquisition

All measurements were performed with an Acquity UPLC H-Class (Waters) using an HSS T3 C18, 100 mm × 2.1 mm, 1.7 µm column (Waters). Separation of 5 µL sample was achieved by a gradient of (A) H_2O + 0.01% FA to (B) MeCN + 0.01% FA at a flow rate of 500 µL/min and 45°C for 7.5 min (5% MeCN, 0-0.3 min; 5-90% MeCN, 0.3-4.7 min; 90-98% MeCN, 4.7-5.5 min; 98% MeCN, 5.5-5.8 min; 5% MeCN, 5.81-7.5 min). The LC flow was directly infused into a Synapt G2-Si operated in positive ion mode. Analysis was conducted using the $HDMS^E$ mode which was set to alternate between collision energies of 0eV and 30eV every 0.3 sec. The instrument was operated in electrospray mode with 20 µg/mL leucine enkephalin lockspray infusion enabled every 10 seconds. Mass spectra were acquired from 50-1500 *m/z* at a 2Hz scan rate in continuum mode without lockmass correction.

Data Processing

All samples were analyzed in triplicate for downstream processing. All samples were measured as 384-well plate batches. Measurements were performed over the course of 7 months. Measurements for each 384-well plate were performed within two weeks of each other, with all replicates of a single 384-well plate batch measured before moving on to the next set of samples. The instrument was mass calibrated and collisional-cross section (CCS) calibrated using MajorMix (Waters) between every 384-well set of samples run. The average *m/z* error was never greater than 0.9 ppm for the calibrant signals prior to acquisition.

Metabolomics processing pipeline

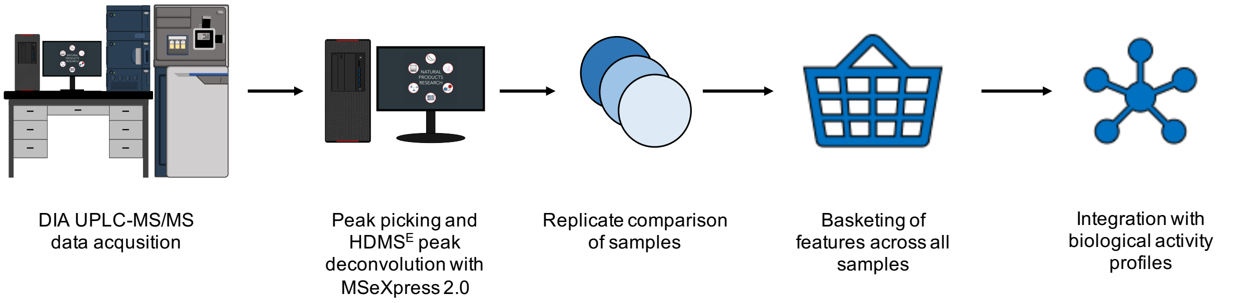

All raw data files were processed using a customized workflow developed in collaboration with Waters. The initial peak detection and $HDMS^E$ deconvolution software package MSeXpress 2.0 was employed to generate peak lists of precursor ions with their associated product ions for each sample. To ensure the validity of signals across replicates, another custom Python script (replicate comparison) removed any *m/z* feature that was not present in at least two of three analytical replicates within 0.03 Da and 0.05 min. Finally, all replicate-compared samples were basketed to give the distribution of all

features across the sample set. Features within 0.03 Da and 0.05 min baskets were collapsed to single features, annotated by the samples in which they appear. Features that appeared in solvent blanks were removed prior to bioactivity integration in the Jupyter notebook.

Supplementary Note 3: Bioassays

Cytological Profiling

General

Briefly, HeLa cells were cultured and seeded into 384-well at 2,500 cells/well. After a 24-hour incubation, cells were pinned with test fractions (or pure compounds) using a Janus MDT robot (PerkinElmer). Two stain sets were used; Stain set 1: Hoechst, EdU-rhodamine, and anti-Phosphohistone H3, Stain Set 2: Hoechst, FITC-alpha tubulin, and rhodamine-phalloidin. For stain set 1, cells were incubated with 20 μM EdU for 1 h prior to fixing in 4 % formaldehyde solution in PBS for 20 min. Cells were then washed with PBS and permeabilized with 0.5% Triton-X in PBS for 10 min before blocking with 2% BSA-PBS solution for at least 1 h. Following this, cells were incubated with primary antibodies overnight at 4ºC. The following day, excess primary antibody was washed off with PBS and secondary antibodies and Hoechst solution were incubated for 1 h. Plates were washed with PBS and preserved with 0.1% sodium azide in PBS solution prior to imaging. For stain set 2, cells were fixed with a 4% formaldehyde solution in PBS for 20 min. Cells were then washed with PBS and permeabilized with 0.5% Triton-X in PBS for 10 min before blocking with 2% BSA-PBS solution for at least 1 h. Following this, cells were incubated with primary antibodies overnight at 4ºC. After blocking the cells were washed and incubated with FITC conjugated anti-alpha tubulin antibody overnight at 4ºC. The following day the cells are washed and then incubated with rhodamine-phalloidin and Hoechst stain for 20 minutes. The cells were then given one final wash before imaging.

Selleck chemicals were screened at both 50 μM and 10 μM in CP. In the downstream analysis, signatures that were inactive in the 10 μM dose were replaced with the signature obtained from 50 μM treatment. Otherwise the signature produced at 10 μM was used for downstream analysis. Macmillan natural product fractions were screened at 10 μg/mL, and Linington natural product fractions were screened at 1000 x dilution from stock. NPFs that were cytotoxic in initial screening were run in 8 pt. 3-fold dilutions starting from 50 μg/ml to obtain active but not dead CP signatures. CP signatures of the first NPF dilutions that was within three SDs of the mean control cell count replaced the initial 50 μg/ml signatures and contributed CP data for SNF merging.

Imaging and Analysis

Two images per well were captured with an ImageXpress Micro XLS automated epiflourescent microscope (Molecular Devices, Sunnyvale). Images were then processed as previously described[3]. Briefly, initial image processing was performed using MetaXpress image analysis software, using built-in morphometry metrics, the multiwavelength cell scoring, transfluor, and micronuclei modules. Custom written scripts were used to compare the treated measurements with the measurements of the DMSO control wells, and then convert each feature to a “histogram difference” (HD) score. This produced a 408-feature vector fingerprint which was then reduced to 251 features using

additional feature reduction steps. Uninformative features with zero standard deviation across all perturbations were removed. In addition, redundancy was further reduced using the find Correlation function in the R-package caret (version 6.0-79; R-version 3.3.3). Briefly, all feature pairs with Pearson correlation coefficients greater than 0.95 were flagged and the member of each pair with the highest mean correlation to all other features was removed, resulting in the final 251-feature CP fingerprint. Compound treatment wells were labeled as ‘dead’ if the cell count for the treatment well was < 10% of the Median cell Count in the treatment plate. This resulted in 31 compounds and NPFs to be labeled as 'dead'. CP scores were calculated as the square-root of the sum of the squares of the CP features.

$$CPscore = \sqrt{\sum_{i=feature\ 1}^{n\ features} {x_i}^2}$$

FUSION

Assay and data processing

All perturbagens were screened in triplicate in the human lung cancer cell line NCI-H23 in 384-well microtiter plate format. Cells were seeded at a density of 5000 cells/well in either 50 or 60 µL of RPMI containing 5% FBS and 1000 U/mL Penicillin-Streptomycin (ThermoFisher). The next day, cells were treated with natural product fraction extract (10 µg/mL from the Macmillan library, and 1000x dilution from the Linington library) or Selleck chemicals (10 µM) using an Echo 555 Liquid Handler (Labcyte; Sunnvale, CA). After 24 hours of incubation, QuantiGene Plex 2.0 Assay Lysis Mixture (ThermoFisher) with 10 µL/mL proteinase K was added to each well for a final media:Lysis Mixture ratio of 2:1. Cells were lysed at 54°C for 30 min, and then stored at -80°C.

The FUSION assay concept was described previously[4]. We extended this concept to a lung cancer context by selecting a new set of genes that can report on the physiological state of lung cancer cell lines in particular. Expression of 14 dynamic reporter genes (*DUSP6, FAM3C, GCNT3, GRHL2, HSD17B7, KIAA0922, LCN2, LTBR, RRM2, SIRPA, TLE2, TMEM30B, WSB2, YAP1*) and 2 static reporter genes (*EEF1A1, SIRT6*) were detected using a 16-plex QuantiGene Plex 2.0 Assay (ThermoFisher). Using a panel of four different sets of 16-plex magnetic and fluorescent beads allowed 4 experiments to be multiplexed into each well, for a final plex of 4 experiments x 16 genes = 64 measurements. An aliquot of cell lysate (~44-47% of the original volume) was then transferred to V-bottom 384-well plates and incubated with bead and hybridization probe mixes at 54°C in a MaxQ 4450 benchtop orbital shaker at 300 rpm for a minimum of 18 hours. After hybridization, samples were 4-plexed by bead set into flat-bottom 384-well plates using a Biomek robotic liquid handler and 384-well plate magnet. Plates were washed in Quantigene Assay Wash Buffer using an ELx405 CW plate washer with a 384-well plate magnet. The signal was then amplified per manufacturer’s protocol using pre-amplifier, amplifier, biotin label, at a ratio of 7.5 µL/mL Label Probe Diluent, and

streptavidin-phycoerythrin reagents at a ratio of 7.5 µL/mL SAPE Diluent. Plates were then washed in SAPE Wash Buffer and read on a Flexmap 3D (Luminex).

FUSION gene expression data is processed first by deconvolution into bead sets, and then normalizing the raw median fluorescence intensity (MFI) value for each gene to the median MFI of that gene on a plate-by-plate basis. To control for cell number, each gene is normalized to the geometric mean of the static controls (*EEF1A1* and *SIRT6)* on a well-by-well basis. Expression values which are > 2x the standard deviation of all values for that gene are then filtered out to eliminate spurious outliers, and only perturbagens with at least 2 replicate values for each gene are processed further. Gene signatures are then normalized to the median of "no treatment" control wells on a plate-by-plate basis, collapsed to the median of each gene, and $\log_2$ transformed to obtain the final FUSION signature. Perturbagens were flagged as 'dead' in the FUSION dataset if the geometric mean of the plate-median normalized MFI values for EEF1A1 and SIRT6 was ≤ 8.

Compound activity assessment

Immunoblot analysis

H23 cells were plated in 6-well format at a density of 150,000 cells/well and allowed to incubate overnight before drug treatment. Upon 24 hour drug treatment, cells were lysed in 1% Triton X-100 buffer mixed with 1X protease and phosphatase inhibitor cocktails (Thermo Scientific). 20 µg of each lysate was loaded and electrophoresed on a 10% SDS-PAGE gel (Bio-Rad) and transferred to a PVDF membrane using the Trans-blot turbo transfer system (Bio-Rad). After blocking with Starting Block (PBS) Blocking Buffer (Thermo Scientific), membranes were probed overnight with primary antibodies diluted at 1:1,000 at 4°C according to manufacturer recommendations. Antibodies against GAPDH (#5174), Rb (# 9309), p-Rb (# 8516) were purchased from Cell Signaling Technology. After washing and incubation with the secondary antibody (HRP-conjugated anti-mouse and anti-rabbit IgG antibodies, Jackson Immunoresearch), protein signals were visualized with ECL Select detection solution (Amersham). GAPDH was used as whole cell lysate loading control.

Gene expression assay after parkamycin treatment

H23 cells were seeded in 6 well plates, 24 h later dosed with DMSO (0.1 %) or RLUS-2088 (10 µM) in DMSO and incubated for 6 h. Cells were lysed and RNA was extracted with Monarch Total RNA Miniprep kit according to manufacturer's instructions (Catalog T2010S). RNA and yield determined using Nanodrop ND-100 spectrophotometer (ThermoFisher). RNA integrity was determined using Agilent Tape station 4150 (Agilent Technologies, Seattle WA) (RIN > 8.5). 100 ng of RNA was hybridized with Human Metabolic Pathways Panel XT-CSO-HMP1-12. Briefly, 70 µl of hybridization buffer was added to Reporter CodeSet to prepare the master mix. To set up the hybridization

reactions, each sample tube contained 8 µl of master mix and 5 µl of diluted RNA sample. Capture ProbeSet (2 µl) was added to each tube and samples were hybridized at 65°C for 18 hours. Samples were processed on an nCounter MAX GEN2 prep station and digital analyzer. Quality control of nCounter data, data normalization and gene expression differences were performed using nSolver and Advanced Analysis Version 2.0.

Supplementary Note 4: Statistical analysis

Similarity Network Fusion (SNF) Methodology

SNF is a novel similarity metric designed to aggregate information across multiple datasets and assign a similarity score to perturbations based on evidence from multiple datasets. SNF was performed as previously described[5], with some modifications (Supplementary Figure 1). Briefly, similarity within a dataset was first calculated. For perturbations i and j, similarity within a single dataset is calculated as:

$$\mathbf{W}(i,j) = \exp\left(-\frac{\rho^2(x_i, x_j)}{\mu\varepsilon_{i,j}}\right)$$

where $w_{i,j}$ gives the scaled similarity and $\rho(x, x_j)$ gives a similarity score (i.e., Euclidean distances) between perturbations i and j in one dataset. For Euclidean distances we used the dist2 function in the SNFtool package. For Pearson correlations, the distance matrix function from the R package ClassDiscovery was used to calculate a Pearson distance similarity matrix. W is calculated for each input dataset to be fused with SNF. The μ value is a hyperparameter that is empirically set and ε is used to eliminate the scaling problem. ε is given as:

$$\varepsilon_{i,j} = \frac{\text{mean}(\rho(x_i, N_i)) + \text{mean}(\rho(x_j, N_j)) + \rho(x_i, x_j)}{3}$$

To compute similarity across multiple sources of data with different scales of measurement a normalized weight matrix, P, was defined where $P = D^{-1}W$ where is D is the diagonal matrix whose entries D(i,i) = $\sum_j W(i,j)$. From here, K-nearest neighbors was used to measure self-similarities as follows:

$$\mathbf{S}(i,j) = \begin{cases} \dfrac{\mathbf{W}(i,j)}{\sum_{k \in N_i} \mathbf{W}(i,k)}, & j \in N_i \\ 0 & \text{otherwise} \end{cases}$$

In this step, the k-nearest neighbors to a perturbation will be assigned similarity scores and the others will be set to zero. We found this step to be critical in introducing errors into the similarity measures for our purposes. Setting the similarity value to zero for similarity measures outside the k-nearest neighbors (KNN) effectively masked perturbation similarity in otherwise significantly similar associations. Thus, we chose to vary k from k=2 to k=n/2, where n is the total number of perturbations in each dataset, and use an agglomerate value of similarity across all k. Networks were then fused by prorogation information from each dataset into the other according to the following equation:

$$\mathbf{P}_{t+1}^{(1)} = \mathbf{S}^{(1)} \times \mathbf{P}_t^{(2)} \times (\mathbf{S}^{(1)})^T$$

$$\mathbf{P}_{t+1}^{(2)} = \mathbf{S}^{(2)} \times \mathbf{P}_t^{(1)} \times (\mathbf{S}^{(2)})^T$$

P and S are defined above where P carries information about the full similarity network and S carries information about similarity for KNN. The superscripts (1) and (2) indicate similarity in data types 1 or 2 (here FUSION or CP). After each iteration, the P matrices were re-normalized as described above. This process is iteratively updated for t-steps after which the final fused matrix is calculated as:

$$\mathbf{P}^{(c)} = \frac{\mathbf{P}_t^{(1)} + \mathbf{P}_t^{(2)}}{2} \; .$$

As stated above, we perform SNF individually for values of k varying from k=2 to k=n/2, resulting in n/2-1 matrices total. To compute an aggregate matrix, we first normalize within each matrix by dividing by the maximum non-diagonal value and then take the average value between all matrices to result in a final, fused aggregate similarity matrix. This matrix was then $\log_{10}$-transformed and clustered by affinity propagation clustering using either Euclidean distance or Pearson correlation as the similarity metric.

Integration of Metabolomics to SNF, CP, and FUSION data

Integration of the basketed metabolomics data was performed similarly to previous studies[7]. This approach treats every observed MS feature or basket as an individual chemical entity and asks the question, on average, what biological phenotype is expected when cells in the high content assays are treated with compound? It is acknowledged that combinations of multiple bioactive species as well as huge differences in titre are to be expected; however, the simplicity and naivety of the question serves as a reasonable starting point for further investigation.

In practice, each basket is treated as an object and assigned five numeric descriptors or attributes specific to the biological data acquisition: CP Cluster Score, CP Activity Score, FUSION Cluster Score, FUSION Activity Score, and SNF Cluster score. The Activity Score describes the strength or magnitude of the phenotype predicted when a basket is detected in an extract. The Activity Score values were calculated by first computing a numeric average of each of the observed attributes for each independent assay. The magnitude, square root of the sum of the squares, of this average predicted phenotype is the Activity Score. The Cluster Score describes the similarity of the phenotypes observed in the biological assays amongst all natural product extracts in which the basket was detected. The Cluster Score is computed using the NXN similarity matrices from each assay or the combined SNF similarity matrix and is simply the average of the nondiagonal values of the sub NXN matrix consisting of all the natural product fractions containing that basket. These descriptors are then exported as a table that can be used for discovery and visualized using tools such as the custom Bokeh server.

Supplementary Note 5: Compound isolation

General

Solvents used for HPLC chromatography were Optima grade and were used without further purification. NMR spectra were acquired on a Bruker 600 MHz Avance II spectrometer equipped with a 5 mm QCI cryoprobe and referenced to residual solvent proton and carbon signals (δ_H 2.50, δ_C 39.5 for DMSO-*d6*, or δ_H 3.31, δ_C 49.0 for MeOD-*d4*). High-resolution mass spectrometry data were acquired using an electrospray ionization (ESI) accurate-mass time-of-flight (TOF) liquid chromatograph−mass spectrometer (Acquity UPLC H-Class tandem Synapt G2-Si).

Fermentation, and extraction

Isolate RL12-067-NTF-D (resulting extract SW218953), isolate RL12-121-HVF-C (resulting extract SW218858), and isolate RL12-115-HVF-E (resulting extracts SW218754, SW218755, SW218756) were inoculated from A1 agar plates into 10 mL of modified SYP (mSYP) liquid media (10 g starch, 4 g peptone, 2 g yeast extract, 1 L Milli-Q water, 31.2 g Instant Ocean) in 25 × 150 mm glass culture tubes for 2 days at room temperature with shaking at 200 rpm before moving to 60-mL medium-scale cultures. Cultures were stepped up to medium-scale by inoculating 3 mL of the small-scale culture into 60 mL of freshly prepared mSYP in wide-mouthed 250-mL Erlenmeyer flasks with small springs. Medium-scale cultures were shaken at 25°C and 200 rpm for 3 days. Large-scale cultures were prepared by inoculating 40 mL of medium scale culture into 1 L of freshly prepared mSYP in 2.8-L Fernbach flasks with a large spring and 20 g of pre-washed Amberlite HP-7 adsorbent resin (DCM, MeOH, and water; Sigma). Large scale cultures were shaken at 25°C and 200 rpm for 7days. At the end of the fermentation period, cells and resin were separated from the culture medium by vacuum filtration using a Whatman® glass microfiber filter and washed with deionized water. Resin and cells from each culture flask were extracted with 250 mL of 1:1 DCM/MeOH. The organic extract was separated from the cells and resin by vacuum filtration and concentrated *in vacuo*.

Fractionation

Crude organic extracts were subjected to solid phase extraction using a TeledyneIsco CombiFlash C18 cartridge (5 g) and eluted using a MeOH/H_2O step gradient at 20 mL/min (40 mL; 10% MeOH, 20% MeOH, 40% MeOH, 60% MeOH, 80% MeOH, 100% MeOH, 100% EtOAc) to afford seven fractions. The 10% MeOH fraction was discarded and the remaining six (fractions A – F) concentrated to dryness *in vacuo*.

An aliquot (2.6 mg) of SW218953 (60 % MeOH) was found to be pure, providing structure **1** (2.6 mg, trichostatin A). All of fraction (153 mg) SW218858 (80% MeOH) was subjected to RP-HPLC (20 ml/min, 62:38, H_2O: MeCN with 0.02 % formic acid) using an Atlantis T3 OBD (19 x 250 mm, 5 µm) column to give compound **2** (4.6 mg, surugamide A). All of fraction (96 mg) SW218756 was subjected to RP-HPLC (1.2 ml/min, 55:45, H_2O:MeCN

with 0.02% formic acid) using a Phenomenex Kinetix C18 (4.6 x 300 mm, 2.6 μm) column to give compound **3** (7.9 mg, parkamycin A).

Yields

Approximate yields per liter of fermentation was as follows:

Compound **1**(trichostatin A) = 100 mg/L

Compound **2** (surugamide A) = 4.6 mg/L

Compound **3**/**4** (parkamycin A/B) = 4.0 mg/L

Supplementary Note 6: Parkamycins A/B isolation, structure elucidation, and NMR table

Compound Isolation: Isolation of Parkamycins A and B was performed via reverse phase HPLC as described above with two clearly separated peaks (12 and 17 minutes, for B then A, respectively). HR-UPLC-MS analysis of each purified peak revealed rapid interconversion to a mixture of the two compounds in both cases, precluding chemical and biological analysis of the pure compounds. To reduce possible thermally mediated interconversion the purification was repeated collecting into test tubes placed in dry ice. However, HR-UPLC-MS analysis of the resulting fractions again revealed rapid interconversion. To reduce photochemically mediated interconversion the purification was repeated a second time in dark conditions, collecting into covered flasks. This resulted in clean separation of the two isomeric products with purities of >95% and >80% respectively based on integration of UV peaks for the two stereoisomers.

Structure elucidation: The molecular formula for parkamycin A was determined to be $C_{26}H_{34}N_2O_5$ from HRESIMS data for the protonated and sodiated adducts (455.2536 and 477.2345 respectively, calcd. 455.2450 and 477.2360) revealing eleven degrees of unsaturation. The ^{1}H and ^{13}C NMR spectra indicated that the structure contained two carbonyl groups (δ_C 174.6 and 174.6) and 12 additional sp^2 hybridized centers (δ_C 121.1, 123.3, 126.2, 127.0, 128.5, 130.0, 130.2, 130.9, 134.7, 137.8, 142.0, and 148.9). ^{1}H and ^{13}C NMR also revealed 12 sp^3 hybridized methylene carbons (δ_C 24.6, 24.6, 28.4, 28.4, 28.6, 28.7, 30.2, 30.5, 30.6, 31.2, 34.0, and 34.0). Overall, the spectra displayed a high degree of repetition, suggestive of a pseudo-dimeric structure. COSY and HMBC correlations combined with long-range $^4J_{HH}$ meta-couplings confirmed the presence of two ortho-substituted aromatic rings, accounting for 8 of the degrees of unsaturation and 12 of the 14 sp^2 hybridized carbons. COSY and HMBC correlations identified two pendant aliphatic chains, each containing six methylene units and terminating in a carbonyl group.

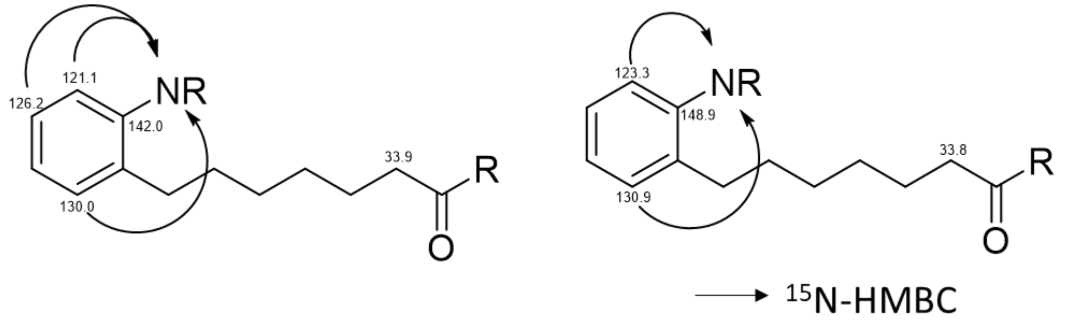

To place the remaining atoms, a ^{15}N-HMBC spectrum was employed to determine the locations of the nitrogen atoms adjacent to each aromatic ring. This left five atoms (H_2O_3) remaining, as well as one double bond equivalent. This could be satisfied by either a cyclic compound with the carbonyls connected head to tail with the aryl nitrogen groups, or with an azoxy moeity joining the two aryl subunits and two carboxylic acid

termini. To confirm the presense of the carboxylic acid termini, parkamycin A (0.1 mg) was treated with TMS-diazomethane (100 eq. dissovled in hexanes) in anhydrous methanol (0.5 mL) at room temperature for 15 minutes. LCMS analysis revealed both both partial (one conversion of COOH to COOMe (+14 Da)) and complete methylation products (two conversions of COOH to COOMe (+28 Da)) confirming the presence of the two acid termini.

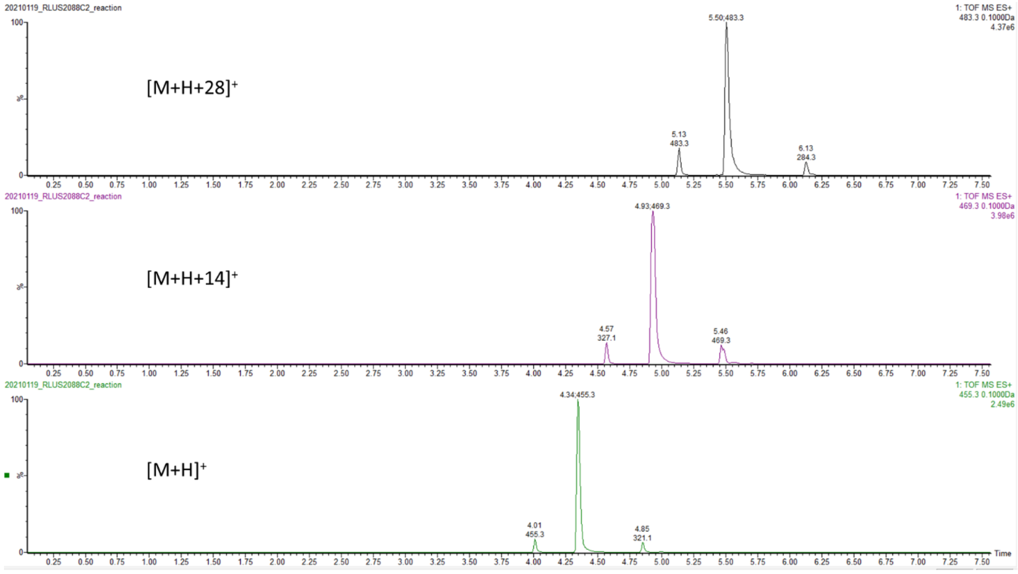

The remaining oxygen atom and double bond equivalent could only be satisfied by the presence of an azoxy moiety between the two aryl rings. This hypothesis was supported by the rapid interconversion between the cis and trans forms upon exposure to sunlight.

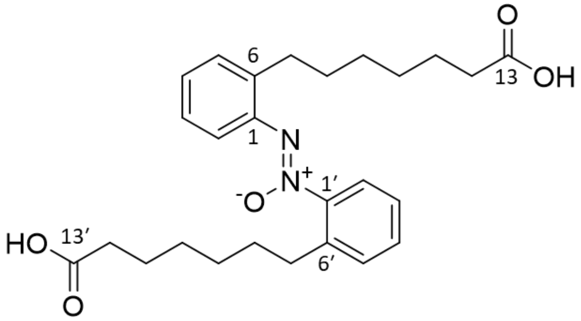

NMR data was acquired on pure parkamycin A for structure elucidation as described above (Figures S17-S26), whereas a sample containing ~85% parkamycin B was used for data collection and assignment of parkamycin B (Figures S27-S30).

Parkamycin A (**3**): UV (MeOH) λ_{max} (log ε) 235 (2.45), 275 (2.31) nm; IR ν_{max} 2933, 1710, 1465 cm^{-1}. ^{1}H NMR (DMSO-*d6*, 600 MHz) δ 7.84 (1H, d, *J* =7.1 Hz, H-2′), 7.62 (1H, d, *J* = 8.4 Hz, H-2), 7.51 (1H, t, *J* =7.5 Hz, H-4), 7.46 (1H, d, *J* =7.5 Hz, H-5), 7.42 (1H, t, *J* =7.6 Hz, H-3), 7.37 (1H, d, *J* =6.7 Hz, H-5′), 7.32 (2H, m, H-3′, H-4′), 2.73 (2H, t, *J* =7.7 Hz, H-7), 2.61 (2H, t, *J* =7.7 Hz, H-7′), 2.11 (4H, m, H-12, H-12′), 1.58 (2H, p, *J*

=7.2, H-8), 1.50 (2H, *J* = 7.0, p, H-8′), 1.43 (4H, m, H-11, H-11′), 1.24 (8H, m, H-9, H-8, H-9′, H-8′) ^{13}C NMR (DMSO-*d6,* 150 MHz) δ 174.6 (C, C-13), 174.6 (C, C-13′), 148.9 (CH, C-1), 142.0 (CH, C-1′), 137.8 (CH, C-6′), 134.7 (CH, C-6), 130.9 (CH, C-5), 130.2 (CH, C-4), 130.0 (CH, C-5′), 128.5 (CH, C-4′), 127.0 (CH, C-3), 126.2 (CH, C-3′), 123.3 (CH, C-2), 121.1 (CH, C-2′), 34.0 (CH2, C-12), 34.0 (CH2, C-12′), 31.2 (CH2, C-7′), 30.6 (CH2, C-7), 30.5 (CH2, C-8′), 30.2 (CH2, C-8), 28.7 (CH2, C-10), 28.6 (CH2, C-10′), 28.4 (CH2, C-9), 28.4 (CH2, C-9′), 24.6 (CH2, C-11), 24.6 (CH2, C-11′), ^{15}N NMR (DMSO-d6, 60 MHz) δ 333.2, 346.8, HRESIMS *m/z* 455.2560 (calcd. for $C_{26}H_{35}N_2O_5$, 455.2540). SMILES: OC(CCCCCCC1=CC=CC=C1/[N+]([O-])=N/C2=CC=CC=C2CCCCCCC(O)=O)=O

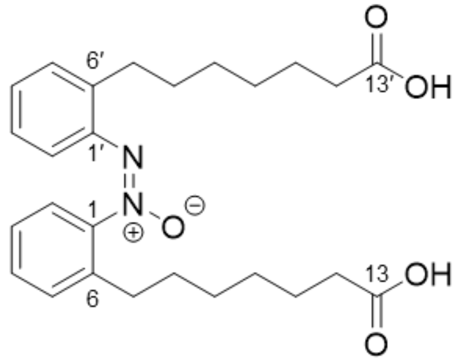

Parkamycin B (**4**): ^{1}H NMR (DMSO-*d6*, 600 MHz) δ 7.31 (2H, m, H-4 and H-5), 7.23 (1H, d, *J* = 7.6 Hz, H-5′), 7.13 (2H, m, H-2 and H-3), 7.05 (1H, t, *J* =7.5 Hz, H-4′), 6.90 (1H, t, *J* =6.9 Hz, H-3′), 6.50 (1H, d, *J* =8.0 Hz, H-2′), 2.67 (2H, m, H-7′), 2.46 (2H, m, H-7), 2.20 (2H, m, H-12), 2.12 (2H, m, H-12′), 1.61 (4H, m, H-8 and H8′), 1.49 (4H, m, H-11 and H11′), 1.29 (8H, m, H-9, H-10, H9′, and H-10′) ^{13}C NMR (DMSO-*d6,* 150 MHz) δ 174.5 (C, C-13), 174.5 (C, C-13′), 147.2 (CH, C-1), 143.3 (CH, C-1′), 135.6 (CH, C-6′), 134.3 (CH, C-6), 130.1 (CH, C-5), 129.9 (CH, C-4), 129.6 (CH, C-5′), 127.3 (CH, C-4′), 126.6 (CH, C-3), 126.1 (CH, C-3′), 122.7 (CH, C-2), 121.4 (CH, C-2′), 33.7 (CH2, C-12), 33.6 (CH2, C-12′), 30.9 (CH2, C-7′), 30.6 (CH2, C-7), 29.5 (CH2, C-8′), 29.4 (CH2, C-8), 28.8 (CH2, C-10′), 28.6 (CH2, C-10), 28.5 (CH2, C-9), 28.3 (CH2, C-9′), 24.5 (CH2, C-11), 24.4 (CH2, C-11) HRESIMS *m/z* 455.2560 (calcd. for $C_{26}H_{35}N_2O_5$, 455.2540). SMILES: OC(CCCCCCC1=CC=CC=C1/[N+]([O-])=N\C2=CC=CC=C2CCCCCCC(O)=O)=O

Cis/trans isomer interconversion: To determine equilibrium ratios between the two forms of this compound (parkamycins A and B), purified samples of each compound were prepared as solutions in both clear and amber vials (0.5 mL of 0.1mg/mL solution in MeOH). All four vials were exposed to ambient light at 10-minute intervals and aliquots from each timepoint analyzed by HR-UPLC-MS.

Under exposure to light (clear vial, top plot), both samples reached an equilibrium ratio of ~85:15 in under 20 minutes. By contrast, exposure to ambient light in amber vials (bottom plot) did not lead to appreciable levels of interconversion. These results demonstrate that parkamycin A is stable under long-term storage conditions, provided

that light is excluded. Further, the relative ratios of the two products at equilibrium supports the assignment of the azoxy configuration as trans for parkamycin A (the more stable isomer) due to steric arguments.

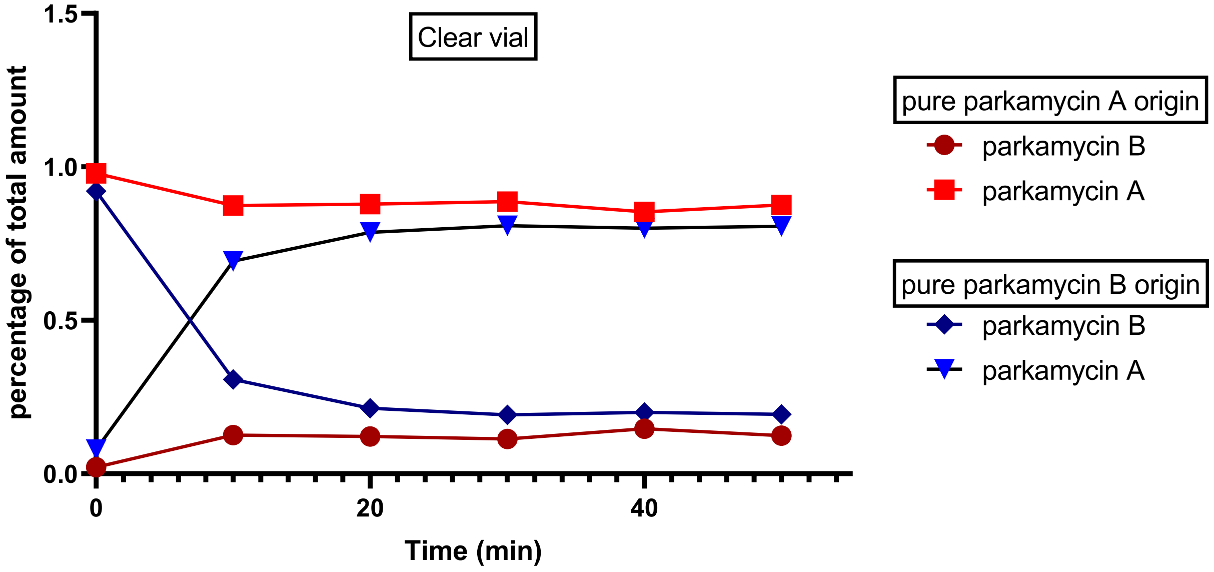

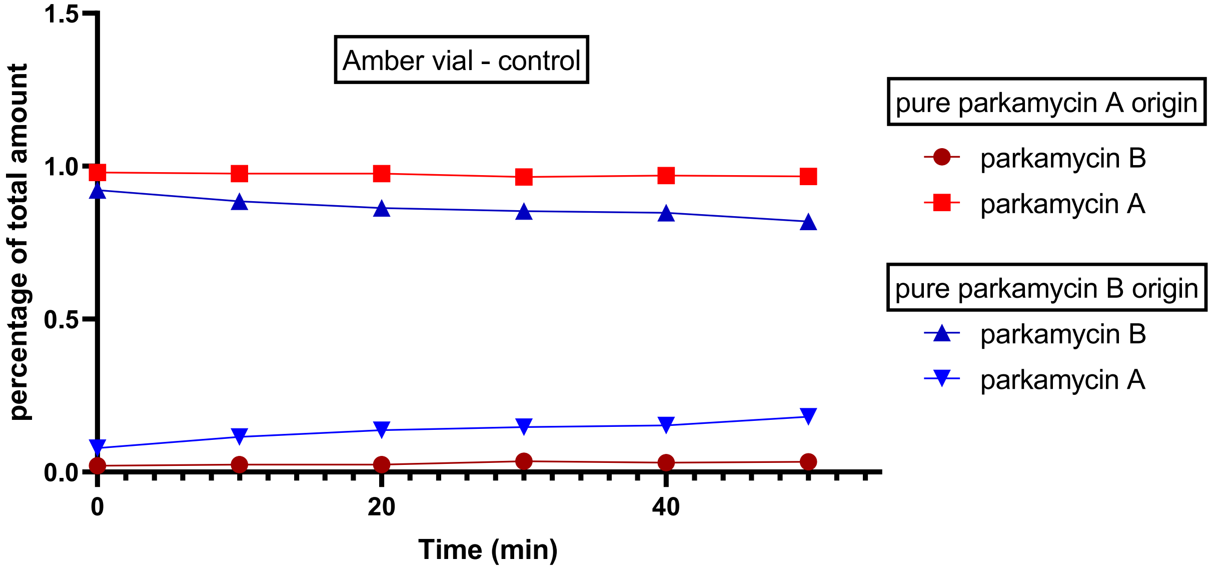

NMR table for parkamycin A and B, acquired at 600 MHz in DMSO-*d6*.

	Parkamycin A			Parkamycin B		
Position	δ_C	δ_H	*J* (Hz)	δ_C	δ_H	*J* (Hz)
1	148.9			147.2		
2	123.3	7.62	8.4	122.7	7.13	
3	127.0	7.42	7.6	126.6	7.13	
4	130.2	7.51	7.5	129.9	7.31	
5	130.9	7.46	7.5	130.1	7.31	
6	134.7			134.3		
7	30.6	2.73	7.7	30.6	2.46	
8	30.2	1.58	7.2	29.4	1.61	
9	28.4	1.24		28.3	1.29	
10	28.7	1.24		28.6	1.29	
11	24.6	1.43		24.5	1.49	
12	34.0	2.11		33.7	2.20	
13	174.6			174.5		
1′	142.0			143.3		
2′	121.1	7.84	7.1	121.4	6.50	8.0
3′	126.2	7.32		126.1	6.90	6.9
4′	128.5	7.32		127.3	7.05	7.5
5′	130.0	7.37	6.7	129.6	7.23	7.6
6′	137.8			135.6		
7′	31.2	2.61	7.7	30.9	2.67	
8′	30.5	1.50	7.0	29.5	1.61	
9′	28.4	1.24		28.5	1.29	
10′	28.6	1.24		28.8	1.29	
11′	24.6	1.43		24.4	1.49	
12′	34.0	2.11		33.6	2.12	
13′	174.6			174.5		

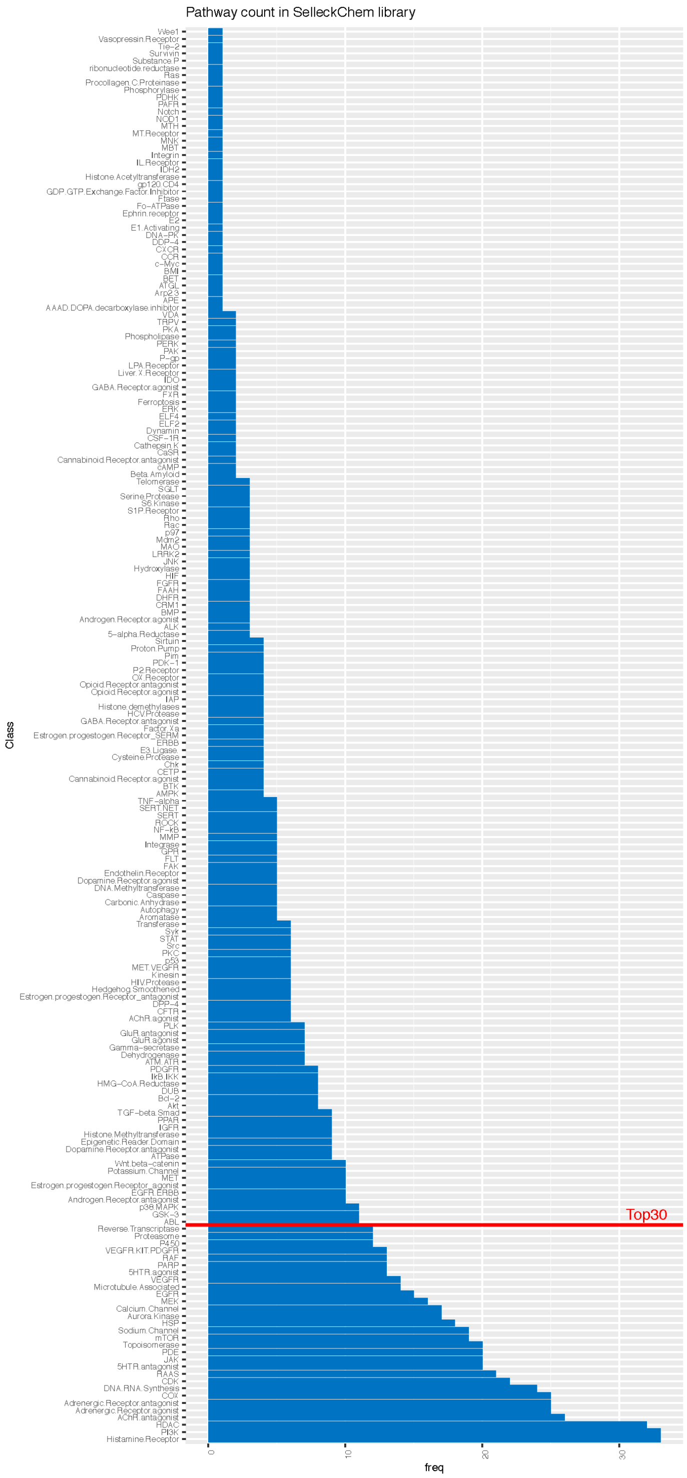

Supplementary Figure 1: Selleck library target class membership.
Annotated targets in the Selleck library are listed in order of the number of chemicals that are assigned to that class. The top 30 largest target classes were selected for comparison by k-means clustering in Figure 1.

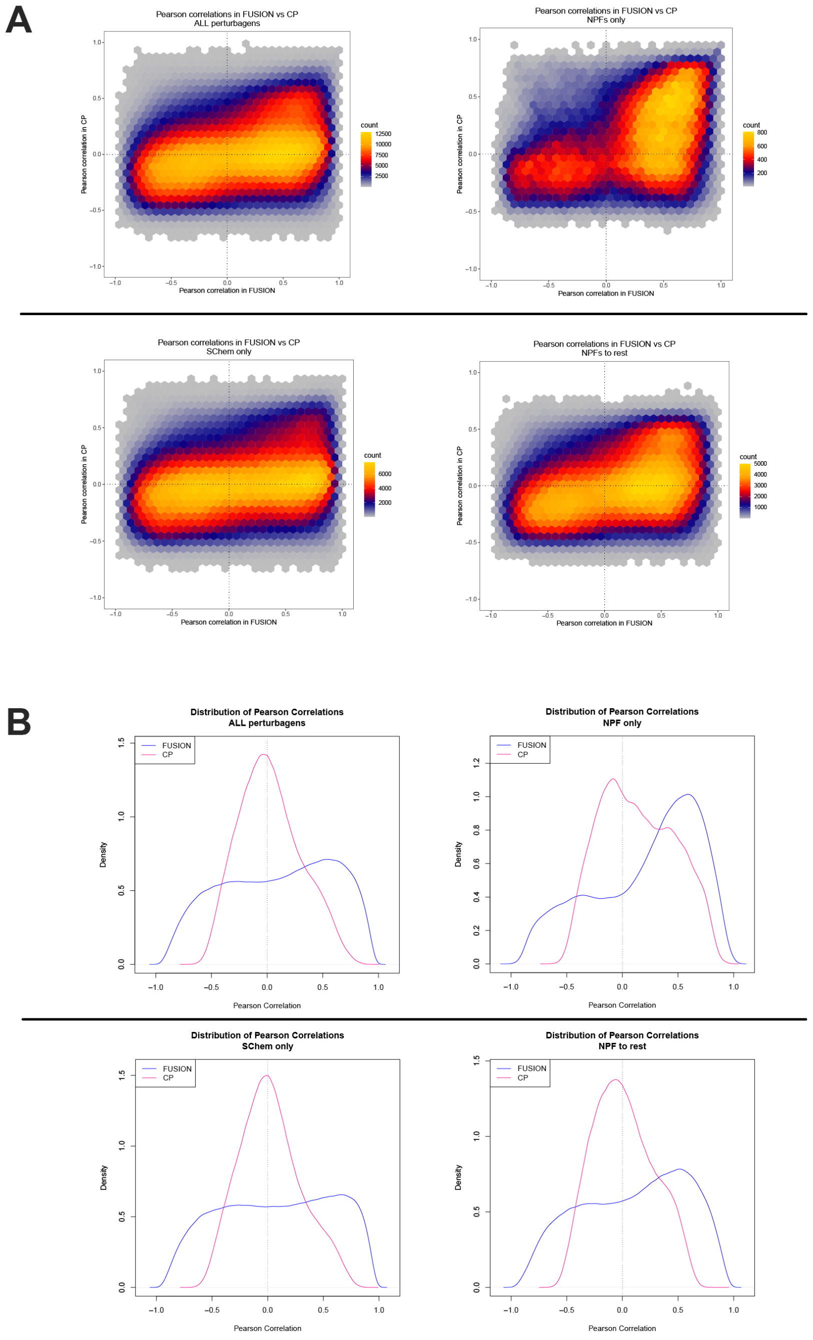

Supplementary Figure 2: Pearson correlation distributions in FUSION and CP.
A) Hexplots and B) density plots illustrating the trends in Pearson correlations between indicated perturbagen types.

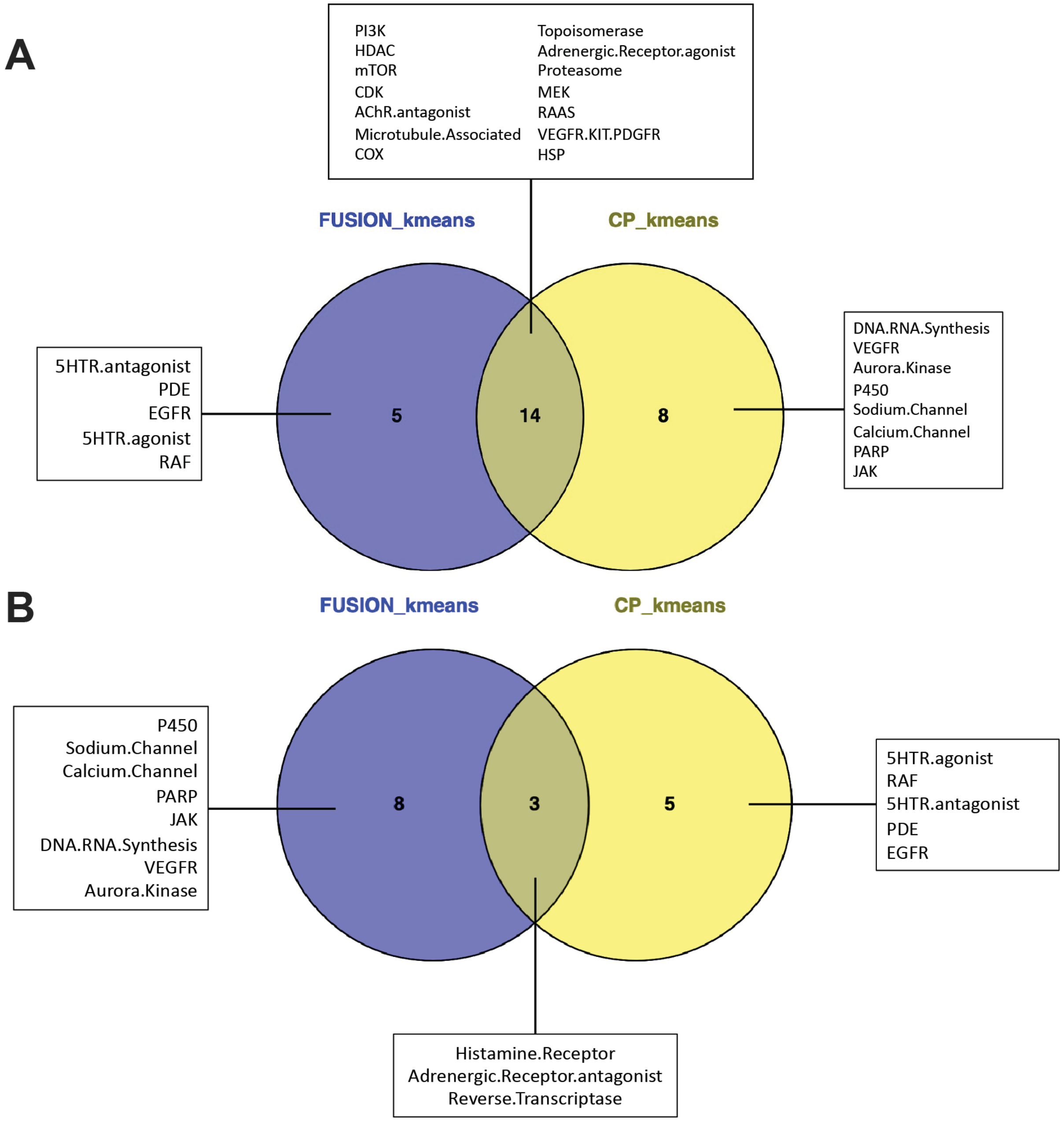

Supplementary Figure 3: Venn diagrams illustrating overlap among target classes identified as significantly enriched in a k-means cluster.
A) Overlap among target classes that were identified as significantly enriched in at least one kmeans (k=30) cluster by hypergeometric test with Bonferroni correction. B) Overlap among target classes that were not identified as significantly enriched by the same method.

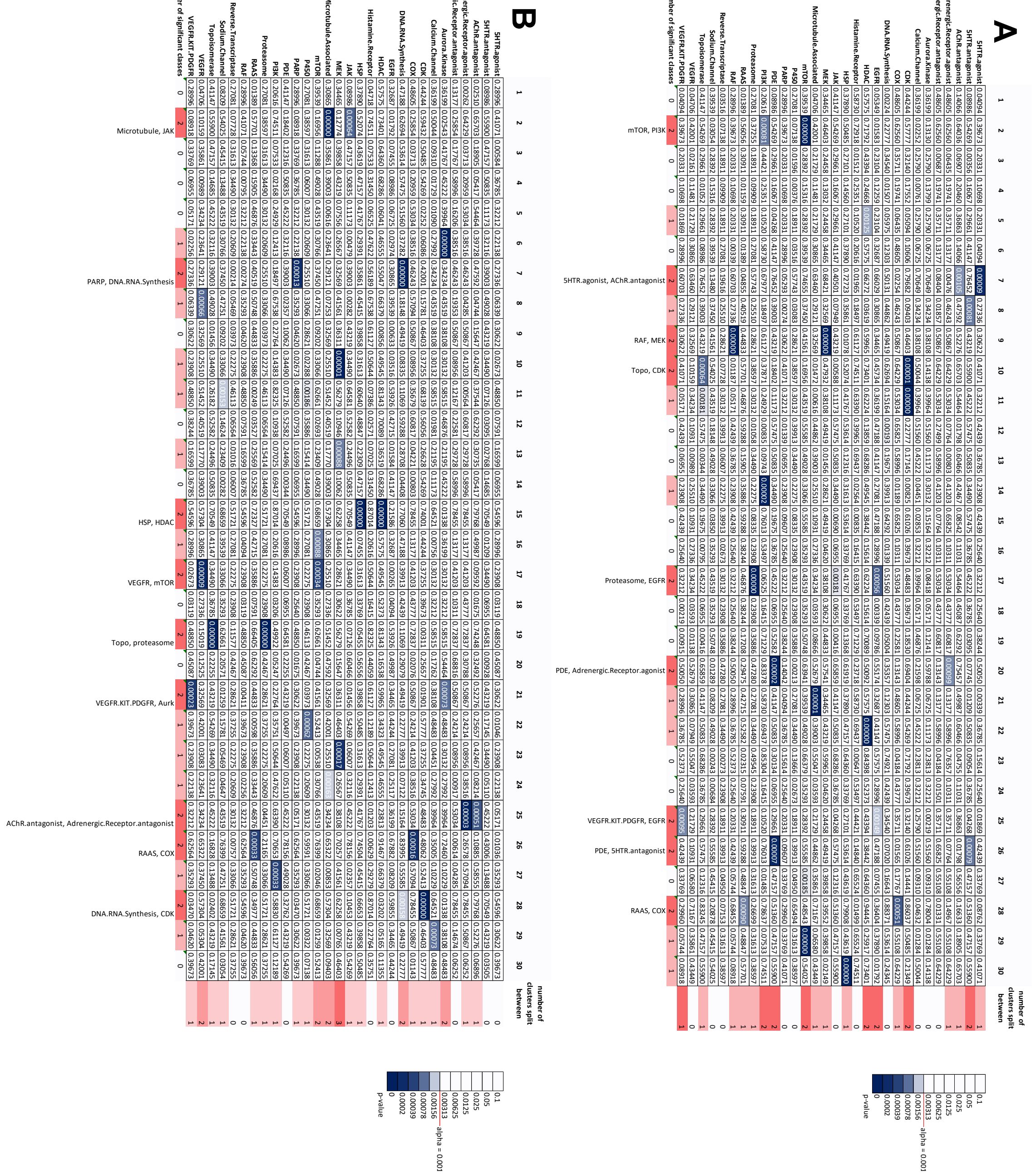

Supplementary Figure 4: Annotated heatmaps showing which target classes are significantly enriched in k-means clusters.

Enrichment was assessed by hypergeometric test (Bonferroni-corrected alpha = 0.0016667) in k-means clusters (k=30) in A) FUSION and B) CP datasets. "Number of significant classes" refers to a count of the number of target classes found to be significantly enriched in that cluster (p<0.00167). "Number of clusters split between" refers to a count of the number of clusters in which a target class was significantly enriched.

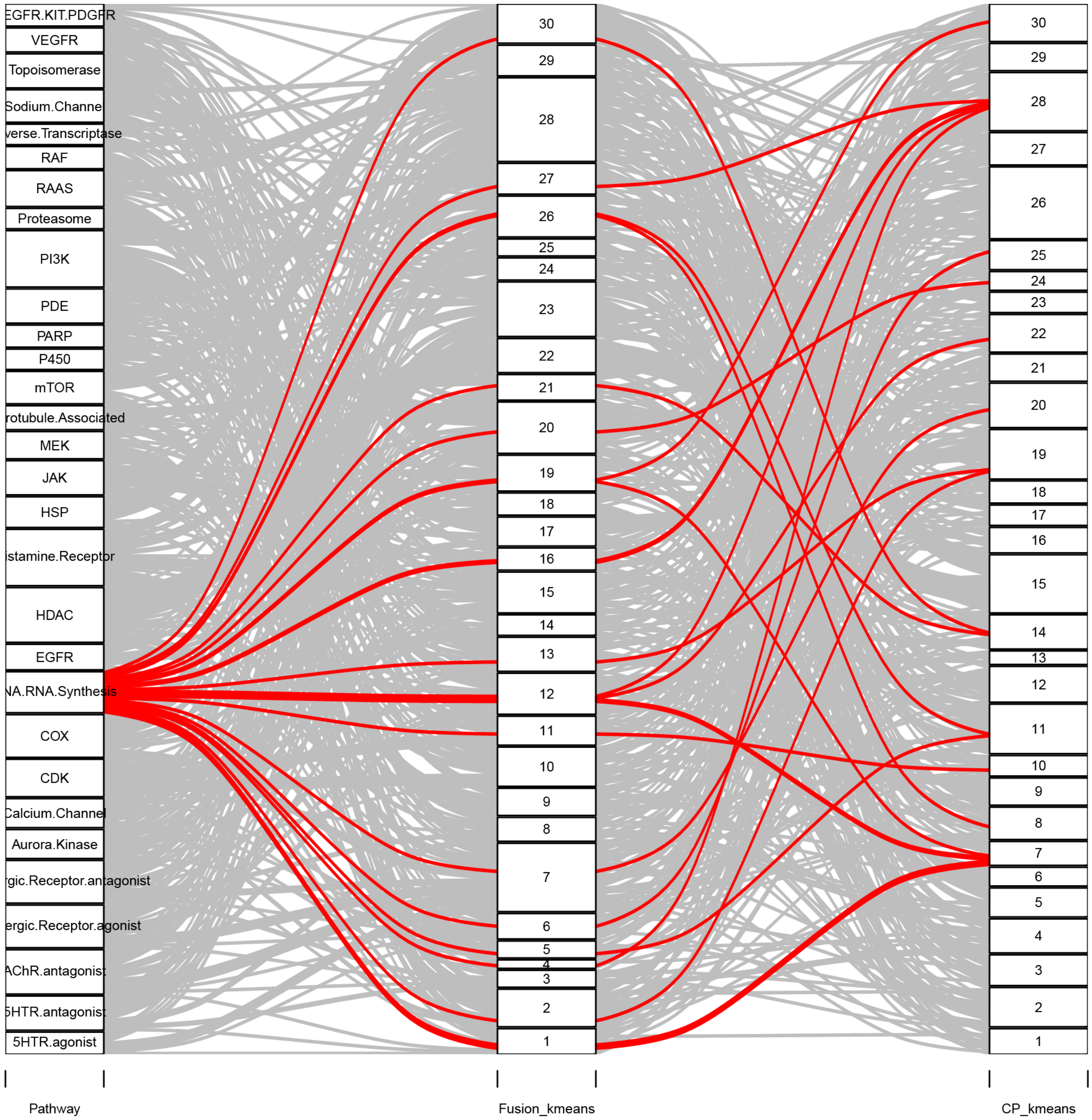

Supplementary Figure 5. Alluvial diagrams for each target class using k-means clustering of the FUSION and CP datasets.

Comparison of k-means clustering (k=30) of FUSION and CP signatures for the top 30 target classes in the Selleck chemical library. Each line represents a compound, and each panel displays a separate target class with chemicals belonging to that class highlighted in red.

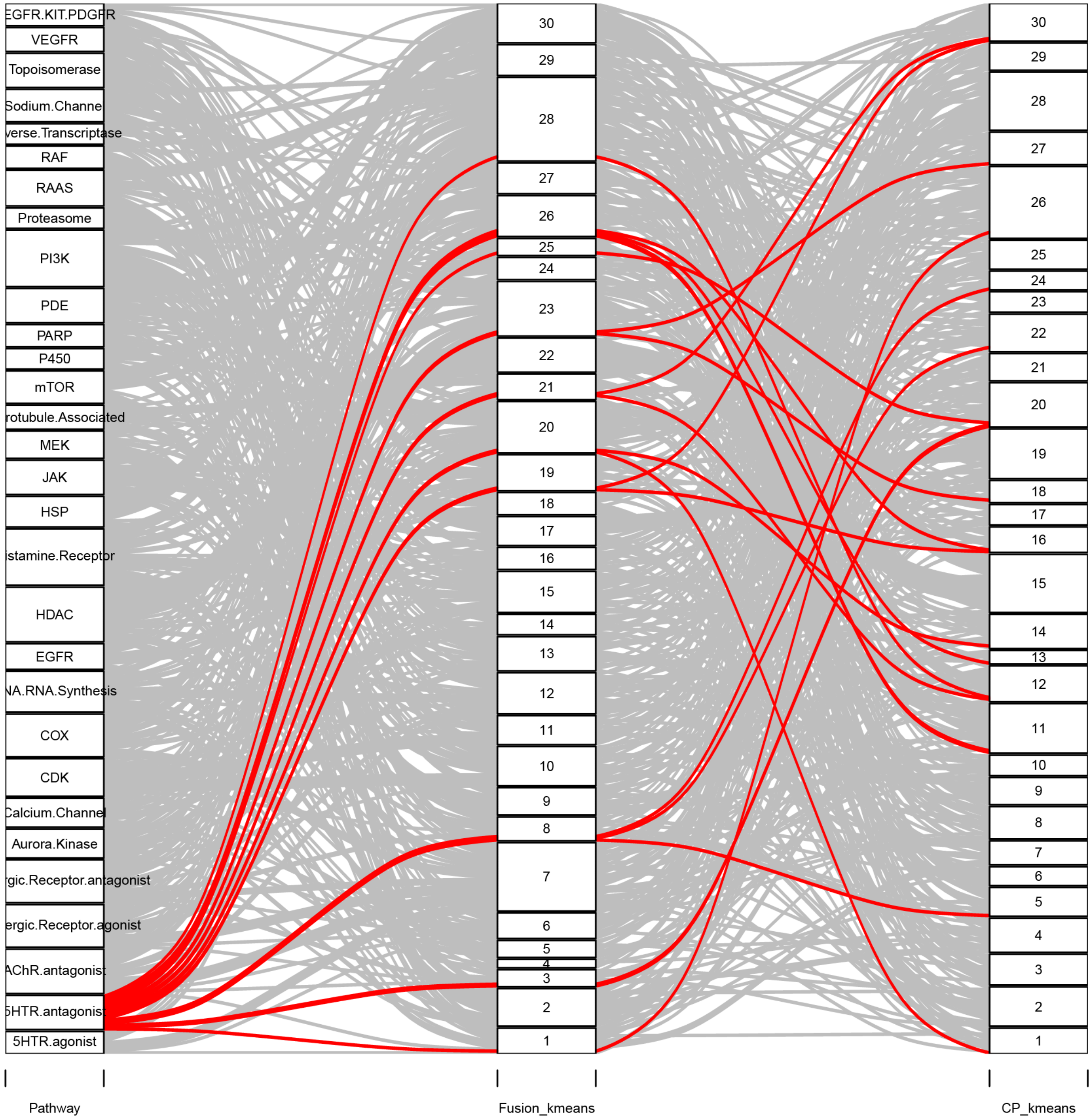

EGFR.KIT.PDGFR
VEGFR
Topoisomerase
Sodium.Channel
verse.Transcriptase
RAF
RAAS
Proteasome
PI3K
PDE
PARP
P450
mTOR
rotubule.Associated
MEK
JAK
HSP
istamine.Receptor
HDAC
EGFR
NA.RNA.Synthesis
COX
CDK
Calcium.Channel
Aurora.Kinase
rgic.Receptor.antagonist
ergic.Receptor.agonist
AChR.antagonist
5HTR.antagonist
5HTR.agonist
30 29 28 27 26 25 24 23 22 21 20 19 18 17 16 15 14 13 12 11 10 9 8 7 6 5 4 3 2 1
30 29 28 27 26 25 24 23 22 21 20 19 18 17 16 15 14 13 12 11 10 9 8 7 6 5 4 3 2 1
Pathway
Fusion_kmeans
CP_kmeans

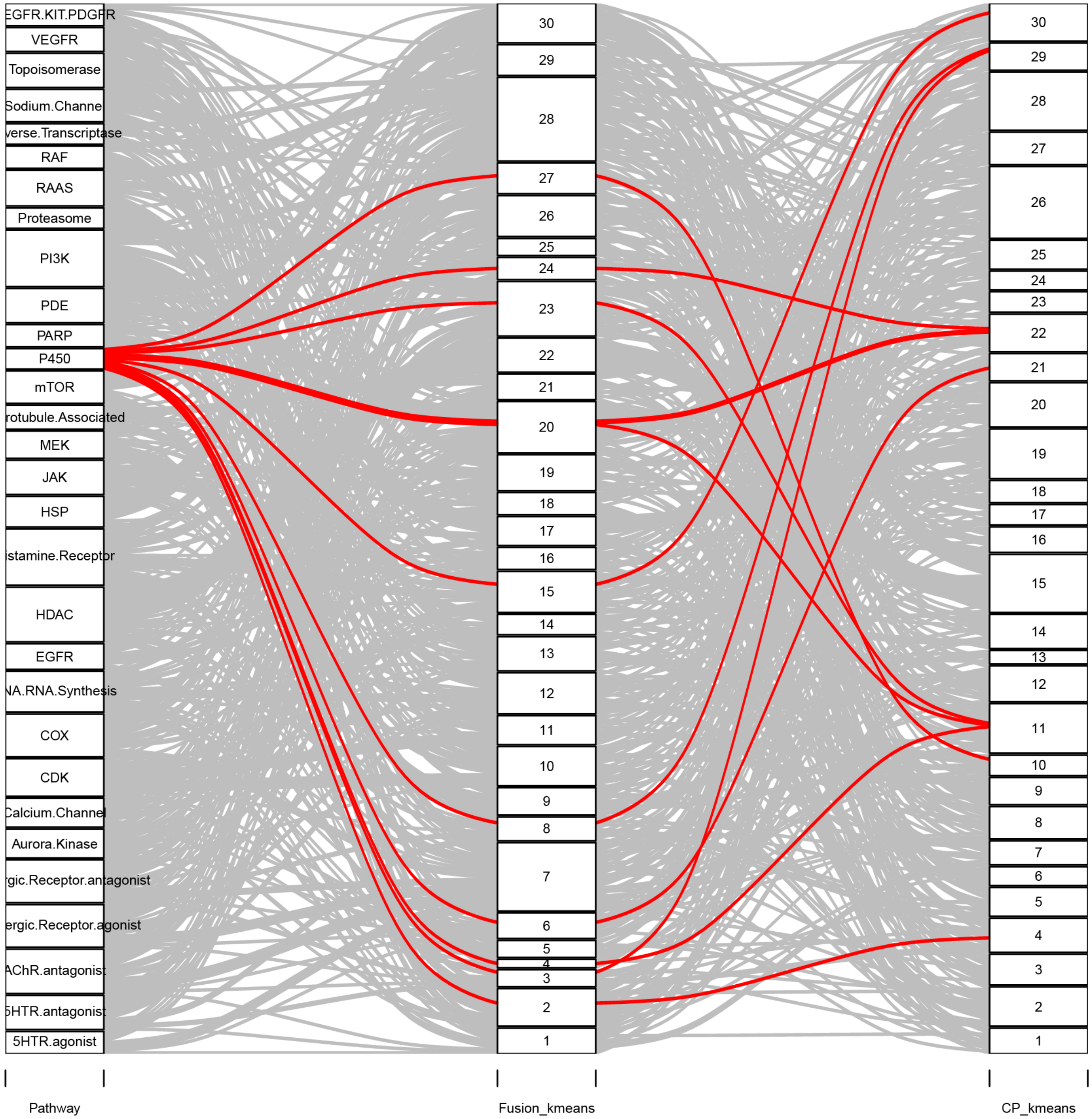
EGFR.KIT.PDGFR
VEGFR
Topoisomerase
Sodium.Channel
verse.Transcriptase
RAF
RAAS
Proteasome
PI3K
PDE
PARP
P450
mTOR
rotubule.Associated
MEK
JAK
HSP
istamine.Receptor
HDAC
EGFR
NA.RNA.Synthesis
COX
CDK
Calcium.Channel
Aurora.Kinase
rgic.Receptor.antagonist
ergic.Receptor.agonist
AChR.antagonist
5HTR.antagonist
5HTR.agonist
30
29
28
27
26
25
24
23
22
21
20
19
18
17
16
15
14
13
12
11
10
9
8
7
6
5
4
3
2
1
Pathway
Fusion_kmeans
CP_kmeans

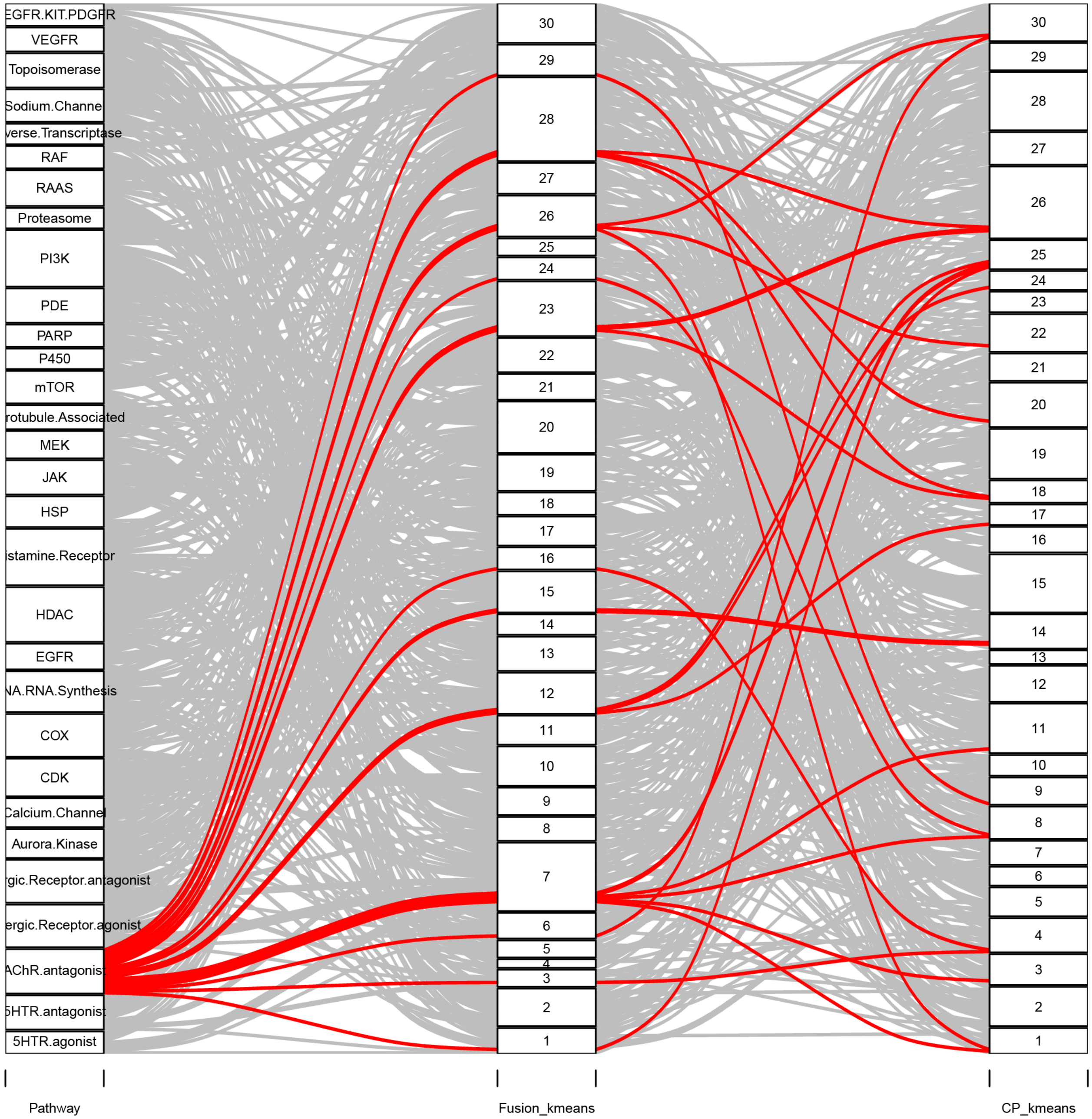
EGFR.KIT.PDGFR
VEGFR
Topoisomerase
Sodium.Channel
verse.Transcriptase
RAF
RAAS
Proteasome
PI3K
PDE
PARP
P450
mTOR
rotubule.Associated
MEK
JAK
HSP
istamine.Receptor
HDAC
EGFR
NA.RNA.Synthesis
COX
CDK
Calcium.Channel
Aurora.Kinase
rgic.Receptor.antagonist
ergic.Receptor.agonist
AChR.antagonist
5HTR.antagonist
5HTR.agonist
Pathway
Fusion_kmeans
CP_kmeans

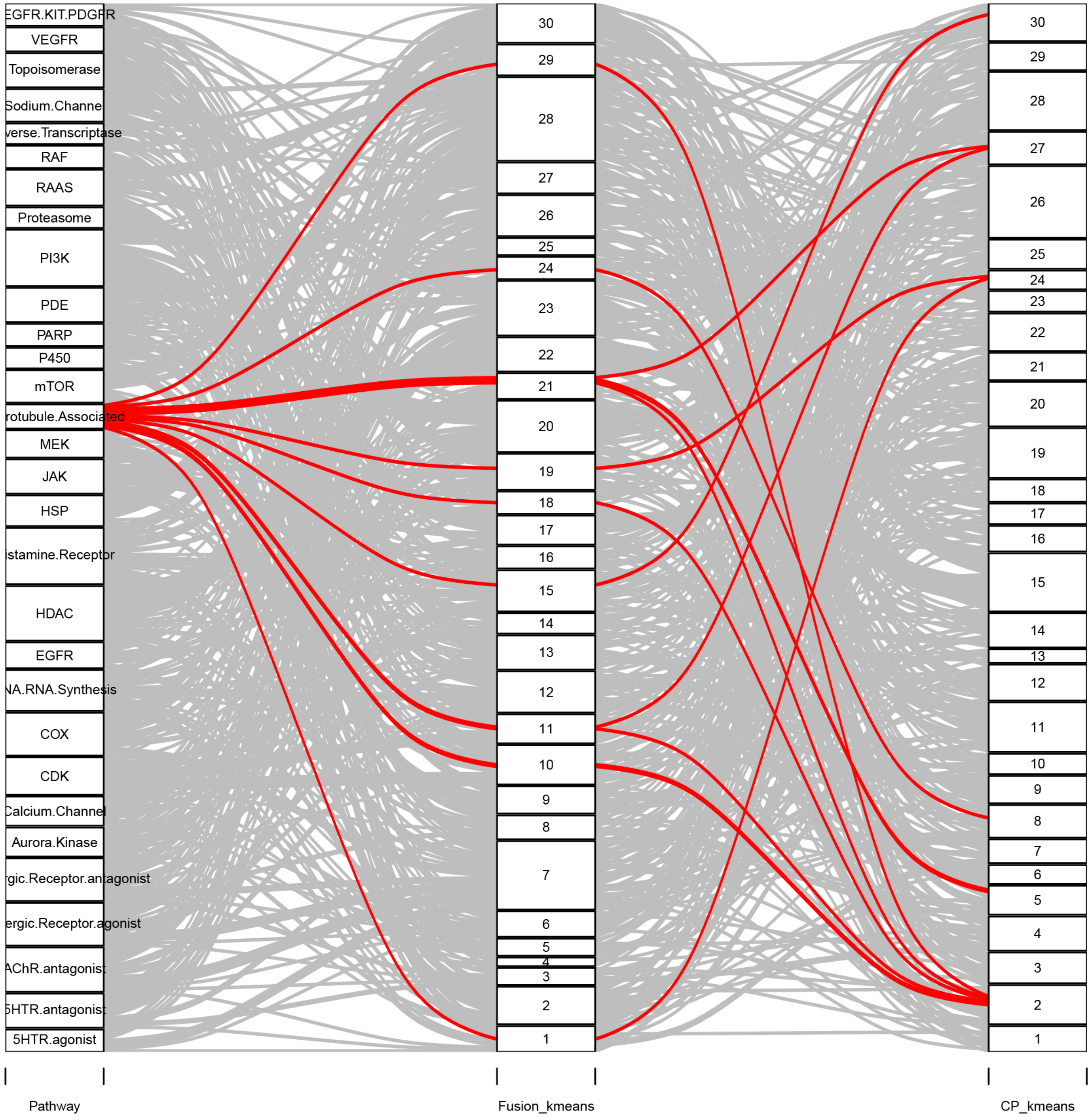
EGFR.KIT.PDGFR
VEGFR
Topoisomerase
Sodium.Channel
verse.Transcriptase
RAF
RAAS
Proteasome
PI3K
PDE
PARP
P450
mTOR
rotubule.Associated
MEK
JAK
HSP
istamine.Receptor
HDAC
EGFR
NA.RNA.Synthesis
COX
CDK
Calcium.Channel
Aurora.Kinase
rgic.Receptor.antagonist
ergic.Receptor.agonist
AChR.antagonist
5HTR.antagonist
5HTR.agonist
Pathway
Fusion_kmeans
CP_kmeans

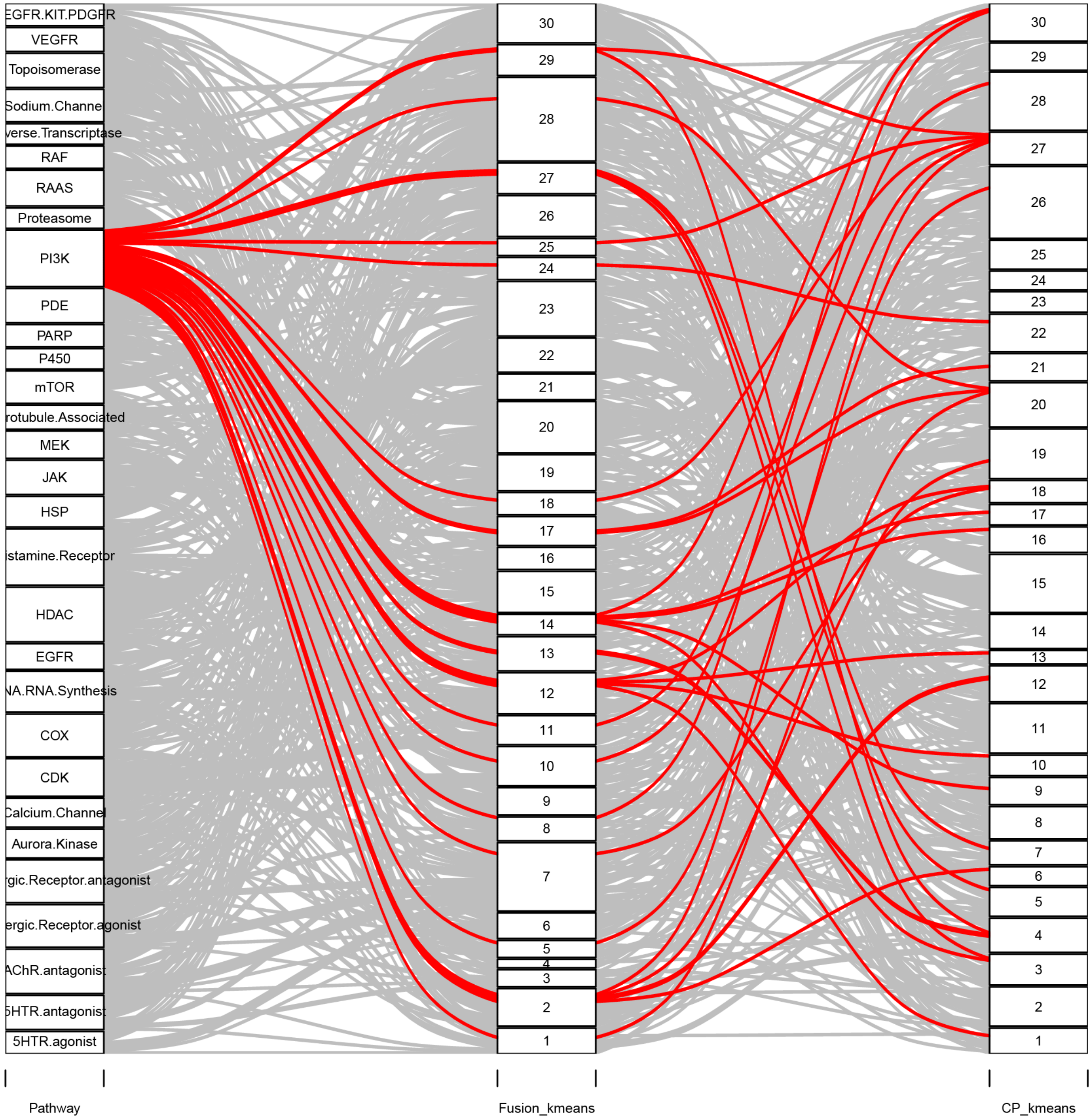
EGFR.KIT.PDGFR
VEGFR
Topoisomerase
Sodium.Channel
verse.Transcriptase
RAF
RAAS
Proteasome
PI3K
PDE
PARP
P450
mTOR
rotubule.Associated
MEK
JAK
HSP
istamine.Receptor
HDAC
EGFR
NA.RNA.Synthesis
COX
CDK
Calcium.Channel
Aurora.Kinase
rgic.Receptor.antagonist
ergic.Receptor.agonist
AChR.antagonist
5HTR.antagonist
5HTR.agonist
Pathway
Fusion_kmeans
CP_kmeans

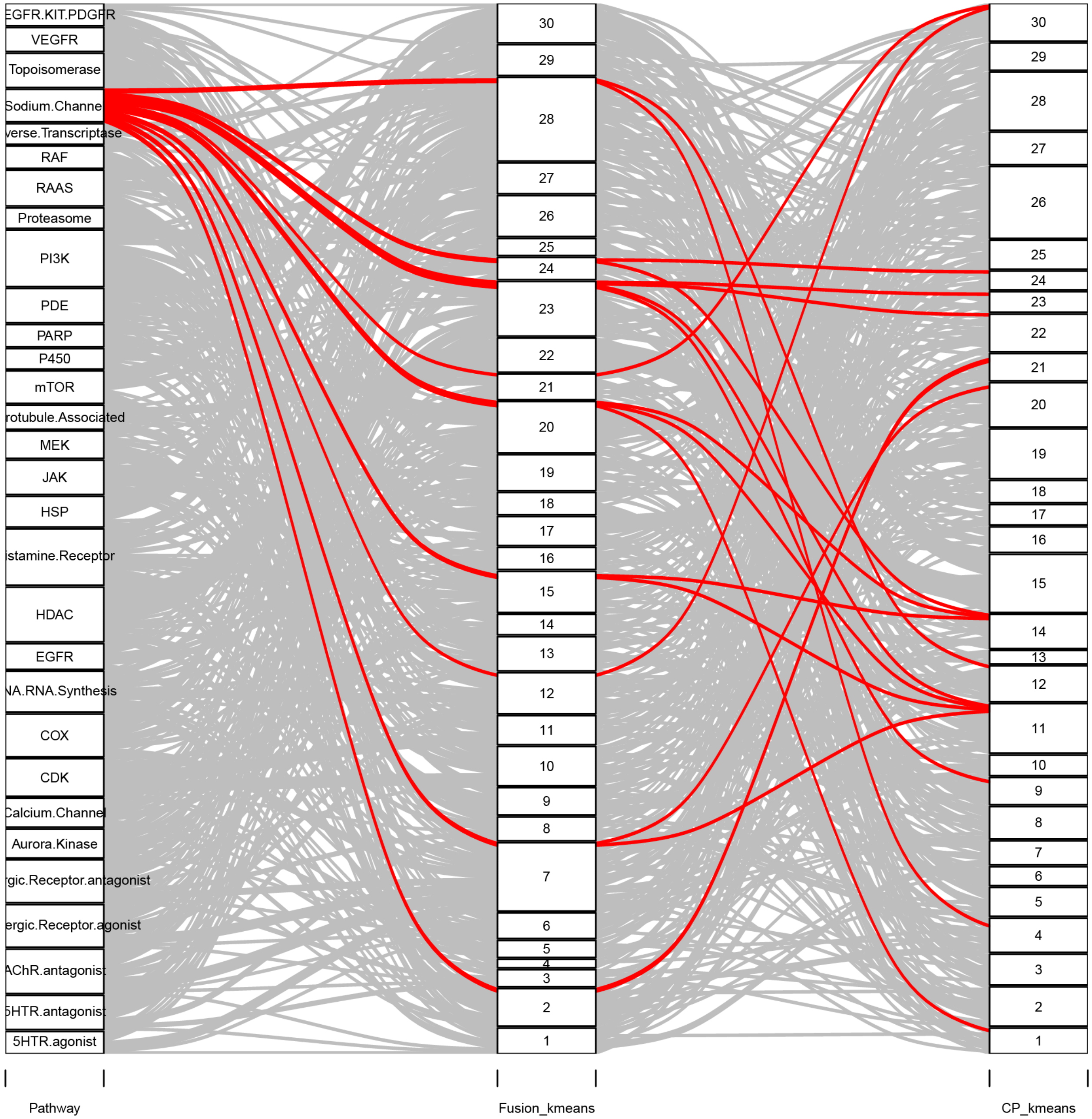
EGFR.KIT.PDGFR
VEGFR
Topoisomerase
Sodium.Channel
verse.Transcriptase
RAF
RAAS
Proteasome
PI3K
PDE
PARP
P450
mTOR
rotubule.Associated
MEK
JAK
HSP
istamine.Receptor
HDAC
EGFR
NA.RNA.Synthesis
COX
CDK
Calcium.Channel
Aurora.Kinase
rgic.Receptor.antagonist
ergic.Receptor.agonist
AChR.antagonist
5HTR.antagonist
5HTR.agonist
Pathway
Fusion_kmeans
CP_kmeans

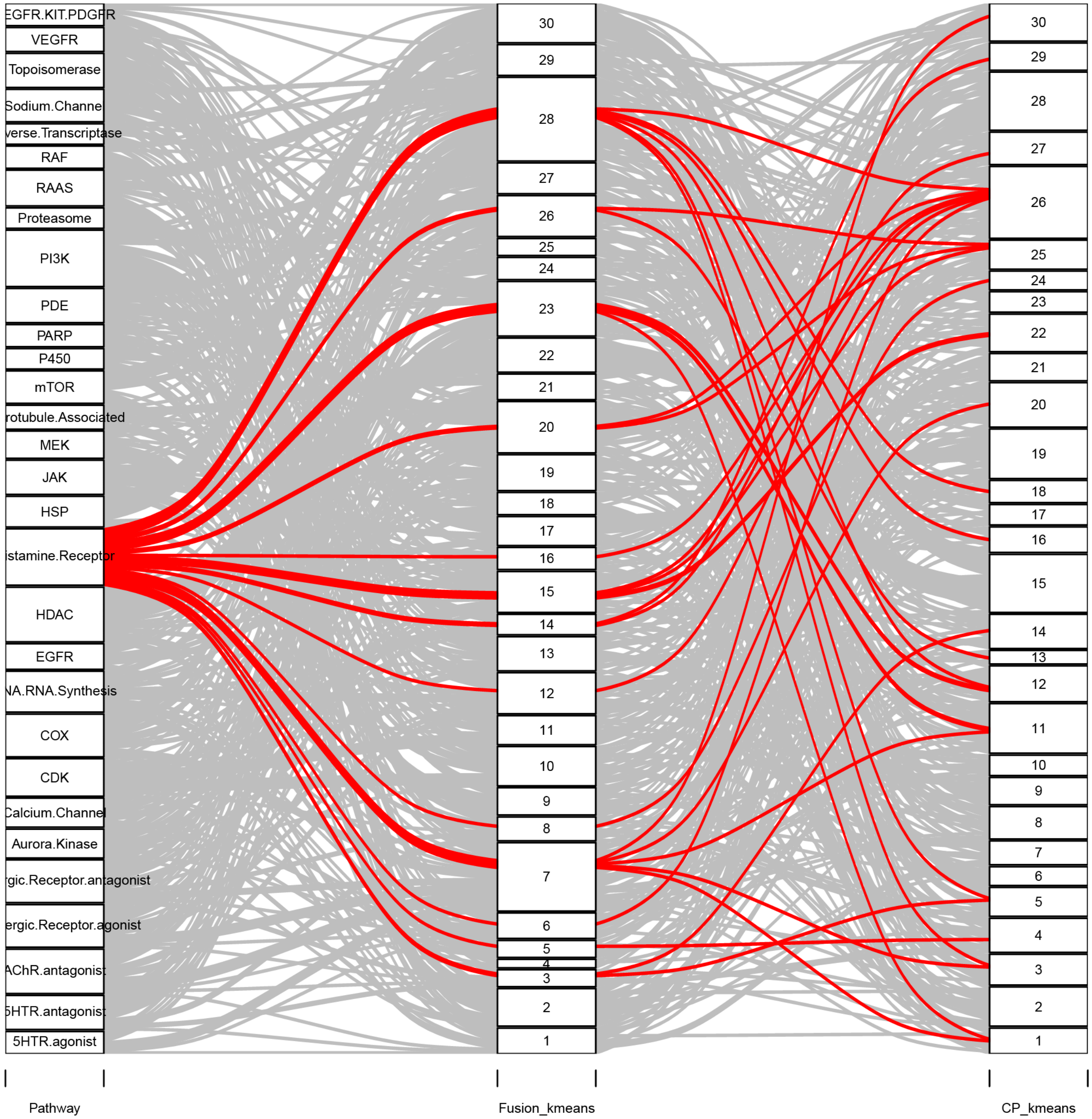
EGFR.KIT.PDGFR
VEGFR
Topoisomerase
Sodium.Channel
verse.Transcriptase
RAF
RAAS
Proteasome
PI3K
PDE
PARP
P450
mTOR
rotubule.Associated
MEK
JAK
HSP
istamine.Receptor
HDAC
EGFR
NA.RNA.Synthesis
COX
CDK
Calcium.Channel
Aurora.Kinase
rgic.Receptor.antagonist
ergic.Receptor.agonist
AChR.antagonist
5HTR.antagonist
5HTR.agonist
Pathway
Fusion_kmeans
CP_kmeans

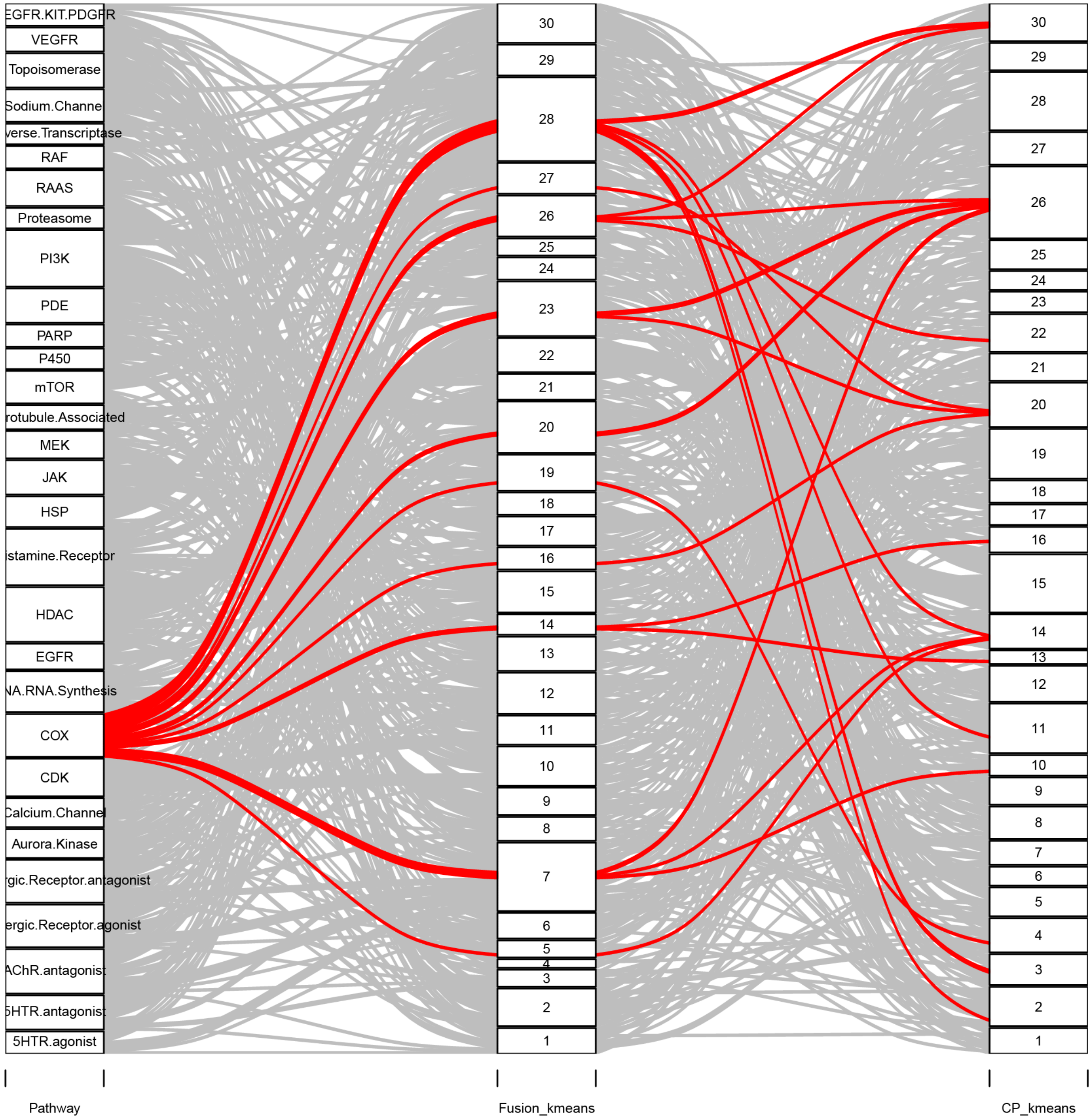
EGFR.KIT.PDGFR
VEGFR
Topoisomerase
Sodium.Channel
verse.Transcriptase
RAF
RAAS
Proteasome
PI3K
PDE
PARP
P450
mTOR
rotubule.Associated
MEK
JAK
HSP
istamine.Receptor
HDAC
EGFR
NA.RNA.Synthesis
COX
CDK
Calcium.Channel
Aurora.Kinase
rgic.Receptor.antagonist
ergic.Receptor.agonist
AChR.antagonist
5HTR.antagonist
5HTR.agonist
Pathway
Fusion_kmeans
CP_kmeans

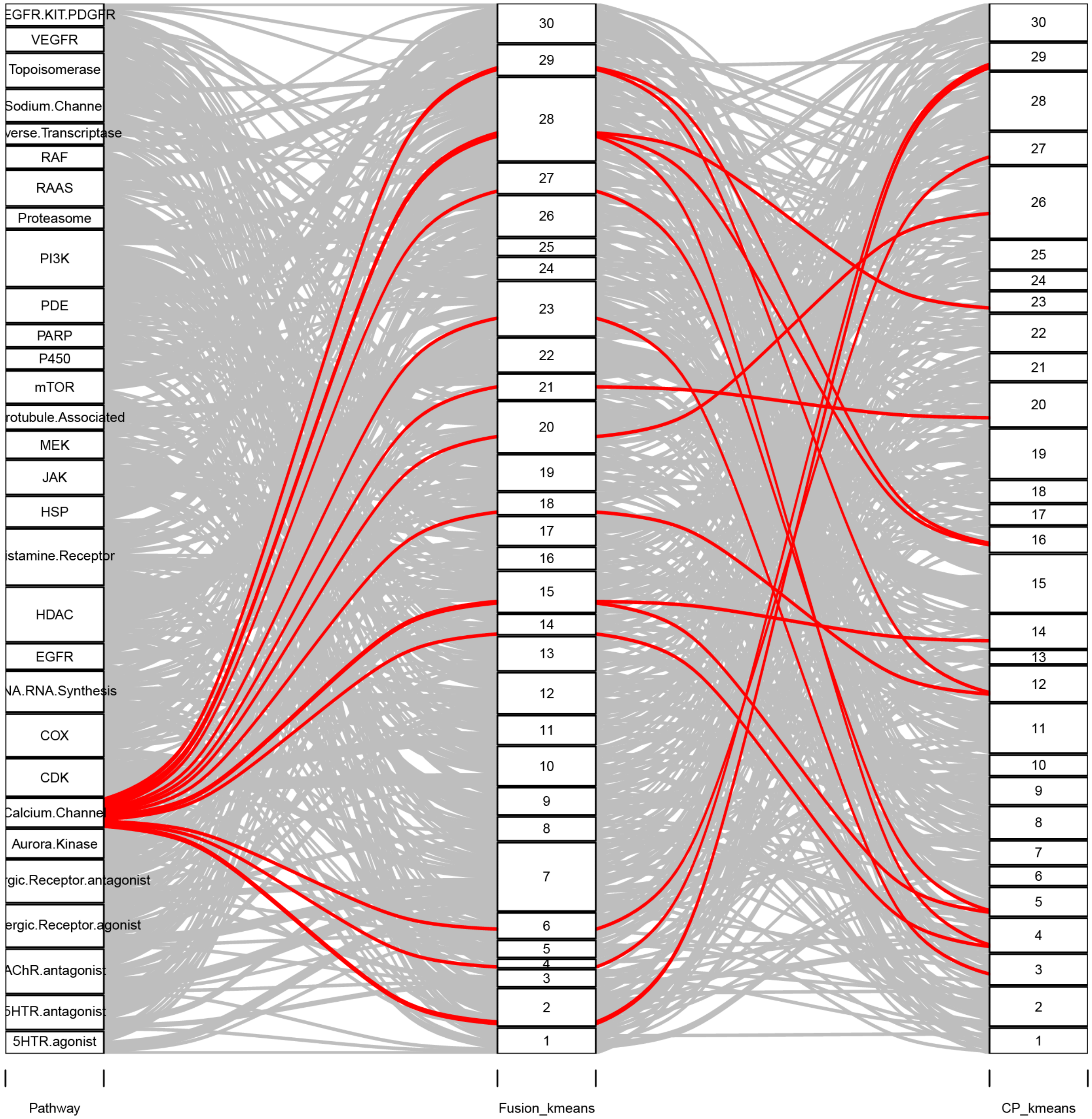

EGFR.KIT.PDGFR
VEGFR
Topoisomerase
Sodium.Channel
verse.Transcriptase
RAF
RAAS
Proteasome
PI3K
PDE
PARP
P450
mTOR
rotubule.Associated
MEK
JAK
HSP
istamine.Receptor
HDAC
EGFR
NA.RNA.Synthesis
COX
CDK
Calcium.Channel
Aurora.Kinase
rgic.Receptor.antagonist
ergic.Receptor.agonist
AChR.antagonist
5HTR.antagonist
5HTR.agonist
Pathway
Fusion_kmeans
CP_kmeans

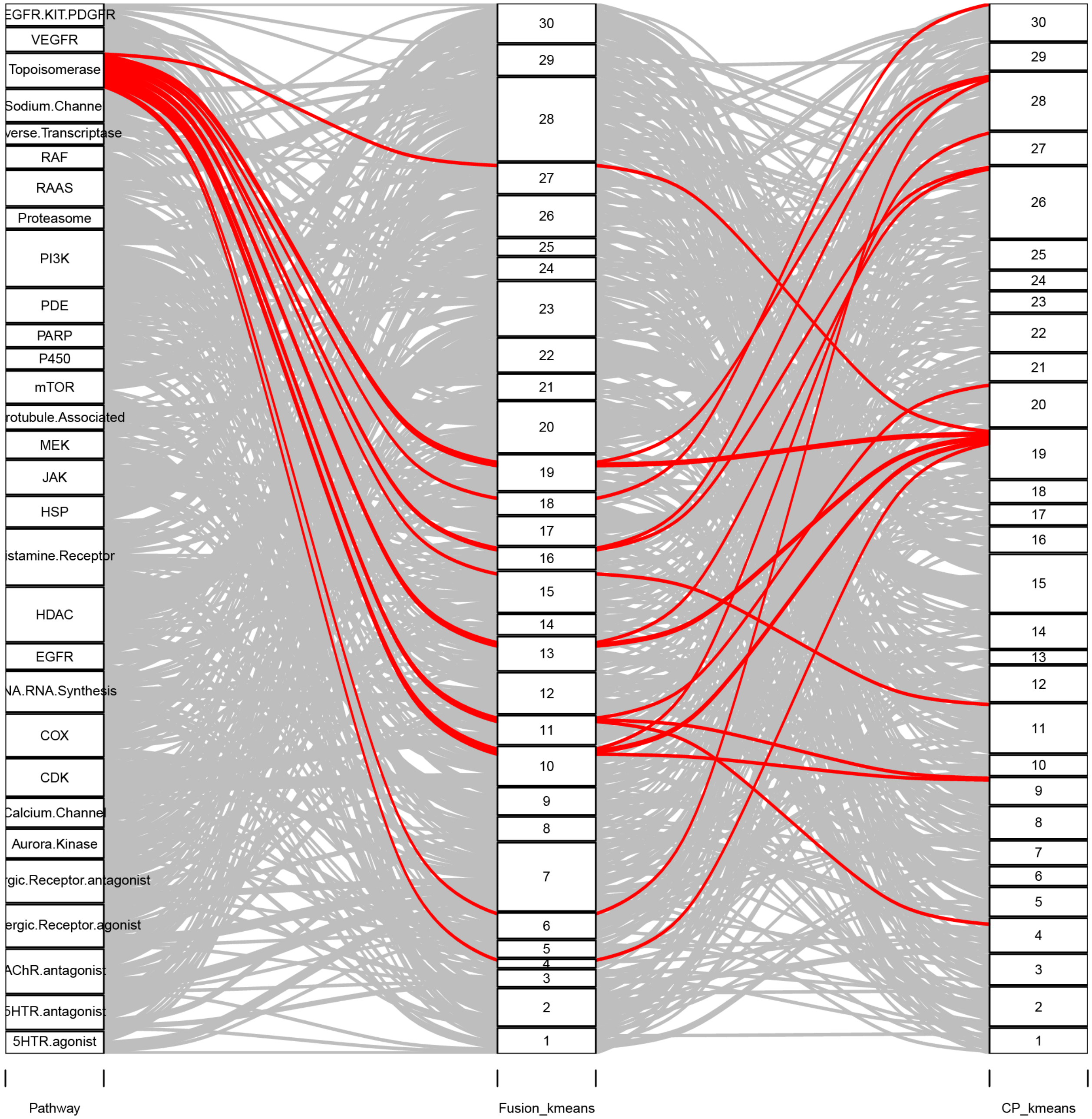

EGFR.KIT.PDGFR
VEGFR
Topoisomerase
Sodium.Channel
verse.Transcriptase
RAF
RAAS
Proteasome
PI3K
PDE
PARP
P450
mTOR
rotubule.Associated
MEK
JAK
HSP
istamine.Receptor
HDAC
EGFR
NA.RNA.Synthesis
COX
CDK
Calcium.Channel
Aurora.Kinase
rgic.Receptor.antagonist
ergic.Receptor.agonist
AChR.antagonist
5HTR.antagonist
5HTR.agonist
30
29
28
27
26
25
24
23
22
21
20
19
18
17
16
15
14
13
12
11
10
9
8
7
6
5
4
3
2
1
Pathway
Fusion_kmeans
CP_kmeans

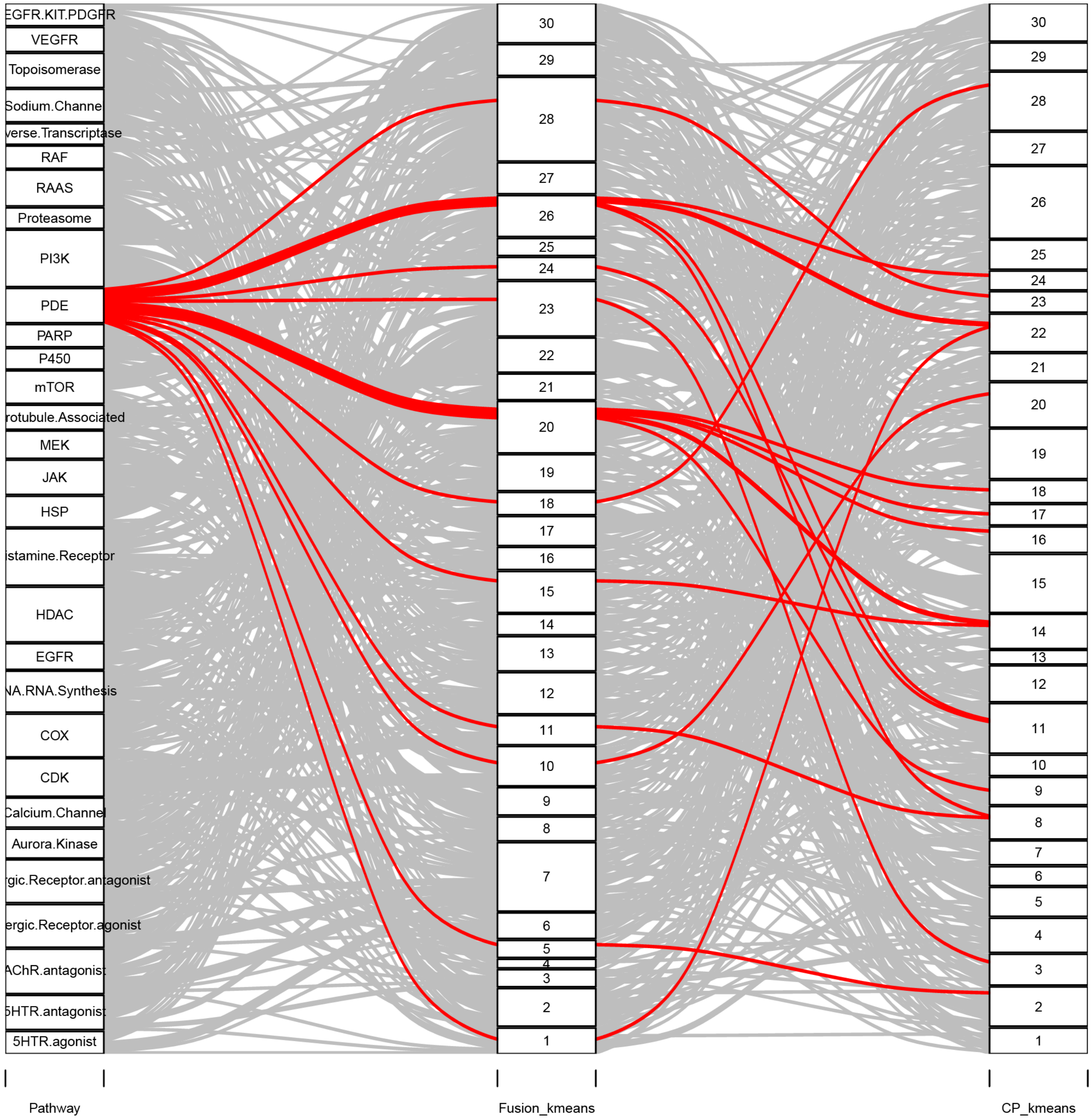

EGFR.KIT.PDGFR
VEGFR
Topoisomerase
Sodium.Channel
verse.Transcriptase
RAF
RAAS
Proteasome
PI3K
PDE
PARP
P450
mTOR
rotubule.Associated
MEK
JAK
HSP
istamine.Receptor
HDAC
EGFR
NA.RNA.Synthesis
COX
CDK
Calcium.Channel
Aurora.Kinase
rgic.Receptor.antagonist
ergic.Receptor.agonist
AChR.antagonist
5HTR.antagonist
5HTR.agonist
30
29
28
27
26
25
24
23
22
21
20
19
18
17
16
15
14
13
12
11
10
9
8
7
6
5
4
3
2
1
Pathway
Fusion_kmeans
CP_kmeans

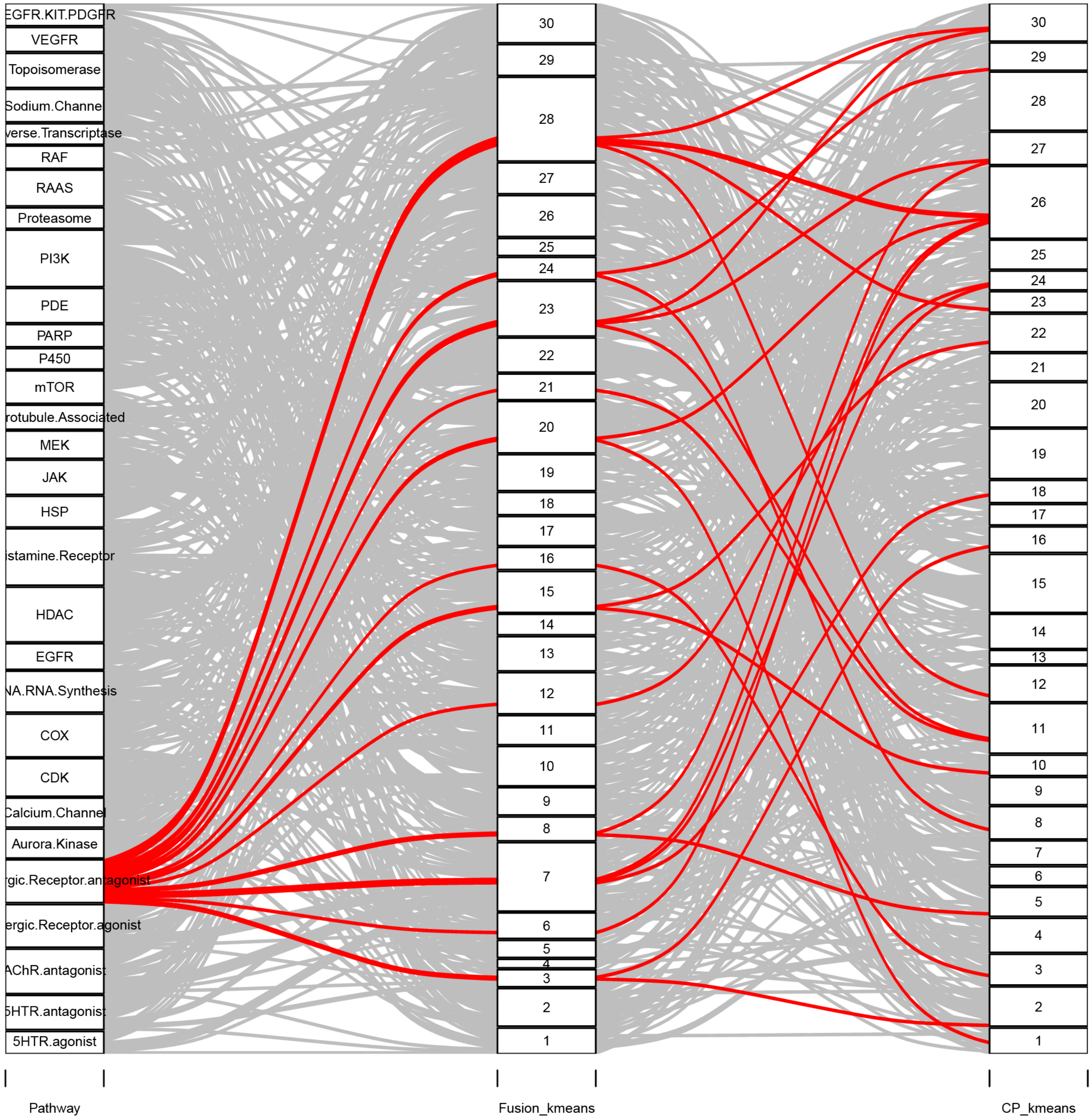
EGFR.KIT.PDGFR
VEGFR
Topoisomerase
Sodium.Channel
verse.Transcriptase
RAF
RAAS
Proteasome
PI3K
PDE
PARP
P450
mTOR
rotubule.Associated
MEK
JAK
HSP
istamine.Receptor
HDAC
EGFR
NA.RNA.Synthesis
COX
CDK
Calcium.Channel
Aurora.Kinase
rgic.Receptor.antagonist
ergic.Receptor.agonist
AChR.antagonist
5HTR.antagonist
5HTR.agonist
Pathway
Fusion_kmeans
CP_kmeans

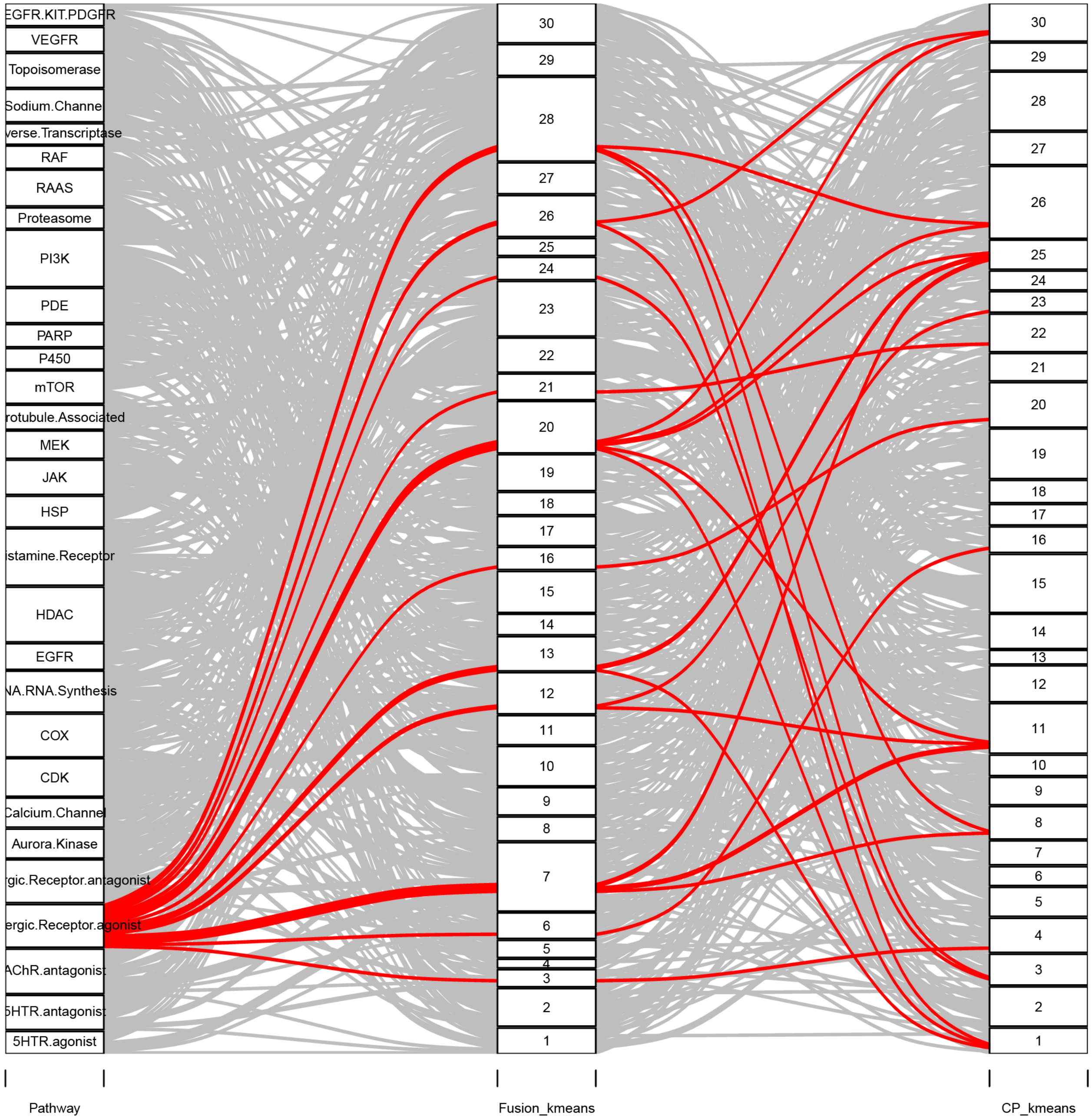

EGFR.KIT.PDGFR
VEGFR
Topoisomerase
Sodium.Channel
verse.Transcriptase
RAF
RAAS
Proteasome
PI3K
PDE
PARP
P450
mTOR
rotubule.Associated
MEK
JAK
HSP
istamine.Receptor
HDAC
EGFR
NA.RNA.Synthesis
COX
CDK
Calcium.Channel
Aurora.Kinase
rgic.Receptor.antagonist
ergic.Receptor.agonist
AChR.antagonist
5HTR.antagonist
5HTR.agonist
30
29
28
27
26
25
24
23
22
21
20
19
18
17
16
15
14
13
12
11
10
9
8
7
6
5
4
3
2
1
Pathway
Fusion_kmeans
CP_kmeans

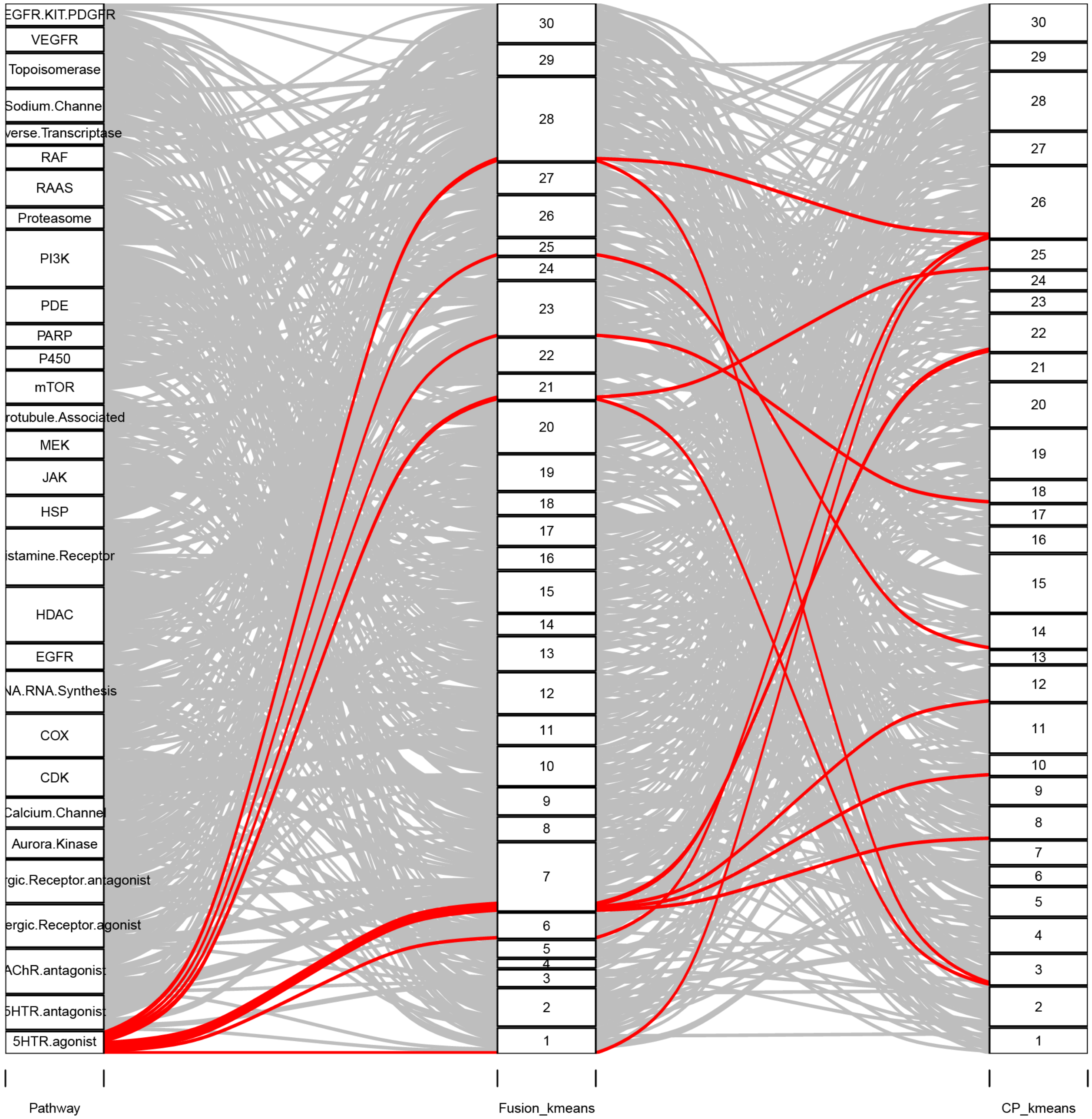

EGFR.KIT.PDGFR
VEGFR
Topoisomerase
Sodium.Channel
verse.Transcriptase
RAF
RAAS
Proteasome
PI3K
PDE
PARP
P450
mTOR
rotubule.Associated
MEK
JAK
HSP
istamine.Receptor
HDAC
EGFR
NA.RNA.Synthesis
COX
CDK
Calcium.Channel
Aurora.Kinase
rgic.Receptor.antagonist
ergic.Receptor.agonist
AChR.antagonist
5HTR.antagonist
5HTR.agonist
Pathway
Fusion_kmeans
CP_kmeans

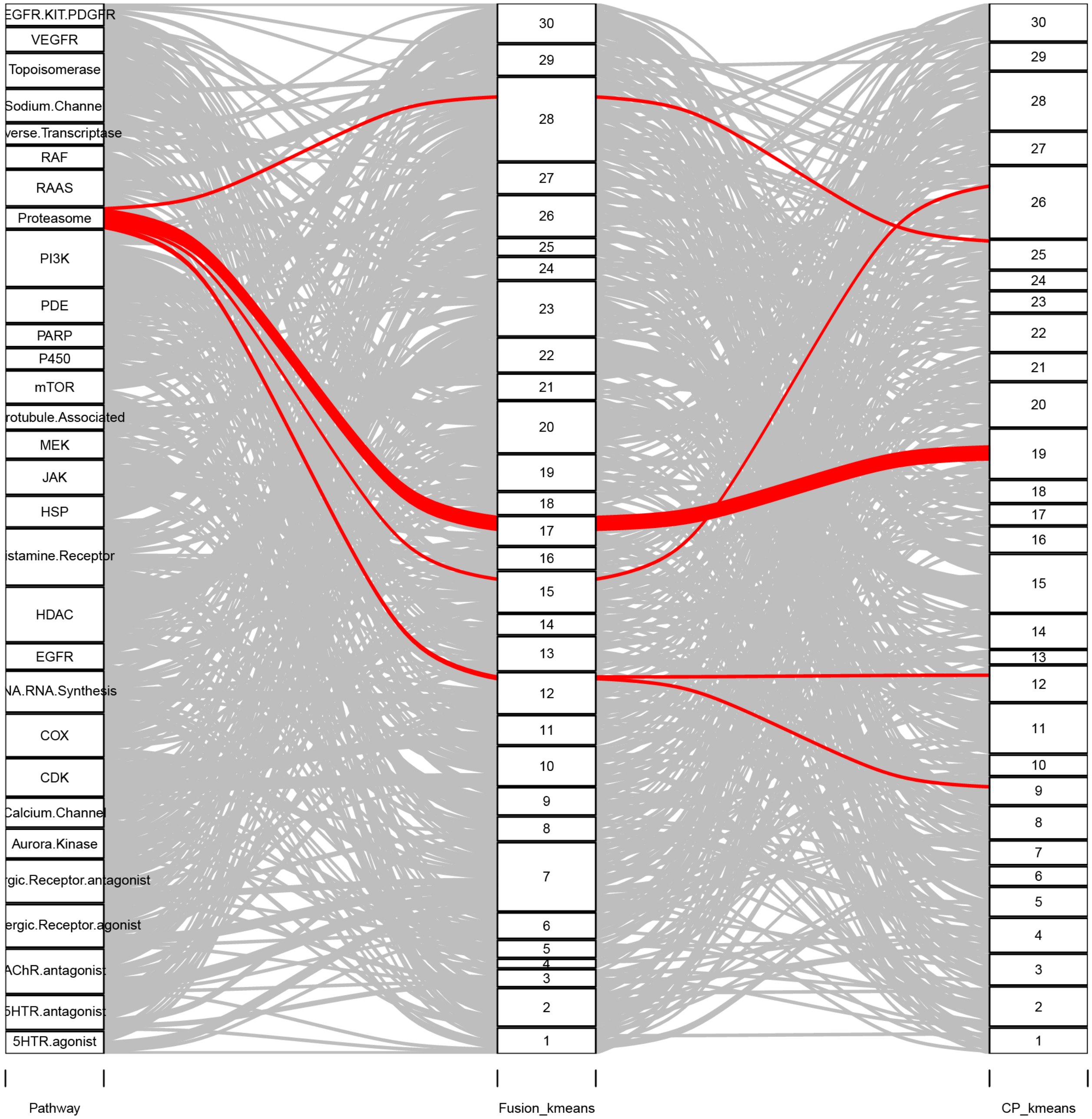
EGFR.KIT.PDGFR
VEGFR
Topoisomerase
Sodium.Channel
verse.Transcriptase
RAF
RAAS
Proteasome
PI3K
PDE
PARP
P450
mTOR
rotubule.Associated
MEK
JAK
HSP
istamine.Receptor
HDAC
EGFR
NA.RNA.Synthesis
COX
CDK
Calcium.Channel
Aurora.Kinase
rgic.Receptor.antagonist
ergic.Receptor.agonist
AChR.antagonist
5HTR.antagonist
5HTR.agonist
Pathway
Fusion_kmeans
CP_kmeans

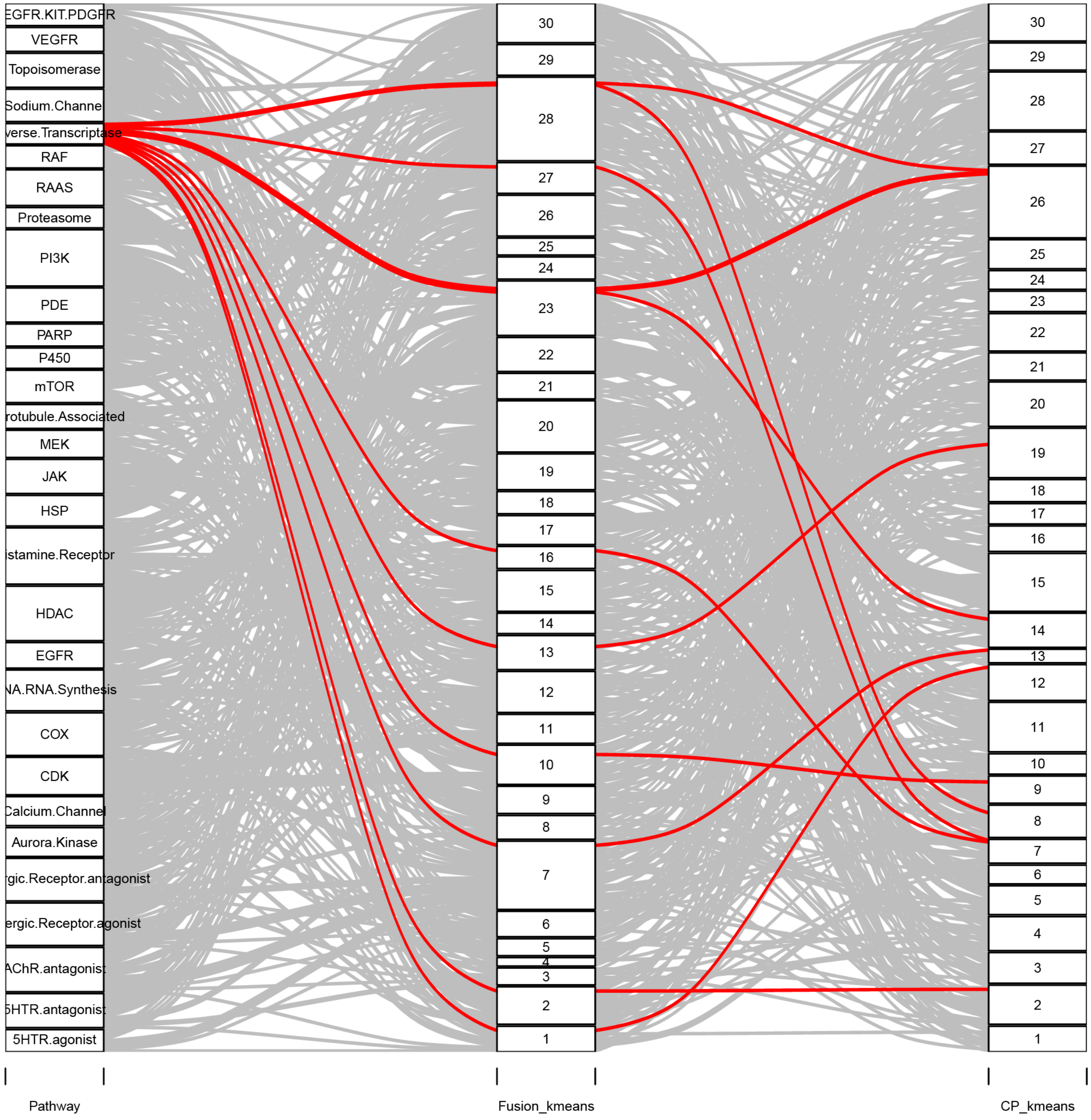
EGFR.KIT.PDGFR
VEGFR
Topoisomerase
Sodium.Channel
verse.Transcriptase
RAF
RAAS
Proteasome
PI3K
PDE
PARP
P450
mTOR
rotubule.Associated
MEK
JAK
HSP
istamine.Receptor
HDAC
EGFR
NA.RNA.Synthesis
COX
CDK
Calcium.Channel
Aurora.Kinase
rgic.Receptor.antagonist
ergic.Receptor.agonist
AChR.antagonist
5HTR.antagonist
5HTR.agonist
Pathway
Fusion_kmeans
CP_kmeans

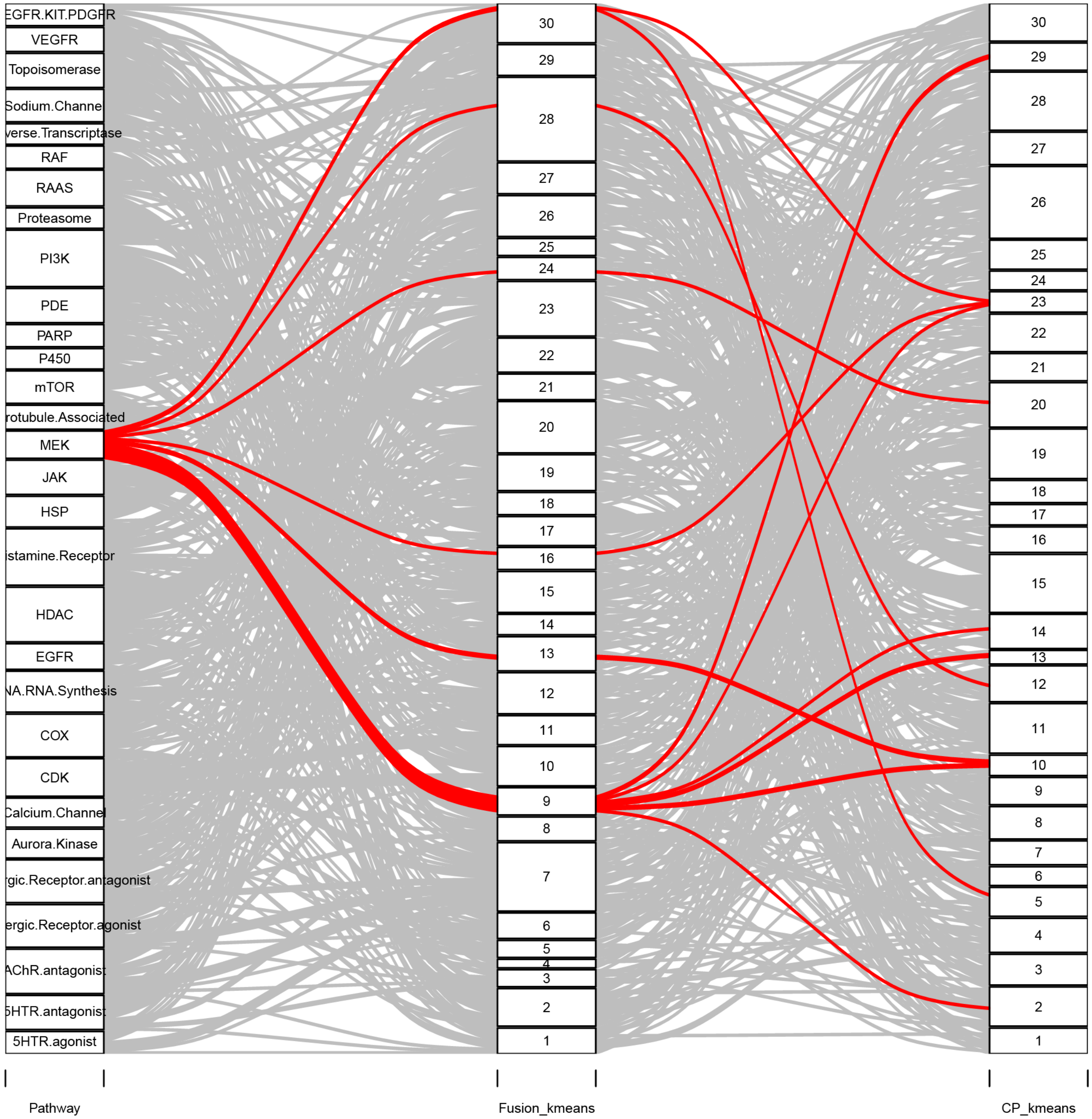

EGFR.KIT.PDGFR
VEGFR
Topoisomerase
Sodium.Channel
verse.Transcriptase
RAF
RAAS
Proteasome
PI3K
PDE
PARP
P450
mTOR
rotubule.Associated
MEK
JAK
HSP
istamine.Receptor
HDAC
EGFR
NA.RNA.Synthesis
COX
CDK
Calcium.Channel
Aurora.Kinase
rgic.Receptor.antagonist
ergic.Receptor.agonist
AChR.antagonist
5HTR.antagonist
5HTR.agonist
Pathway
Fusion_kmeans
CP_kmeans

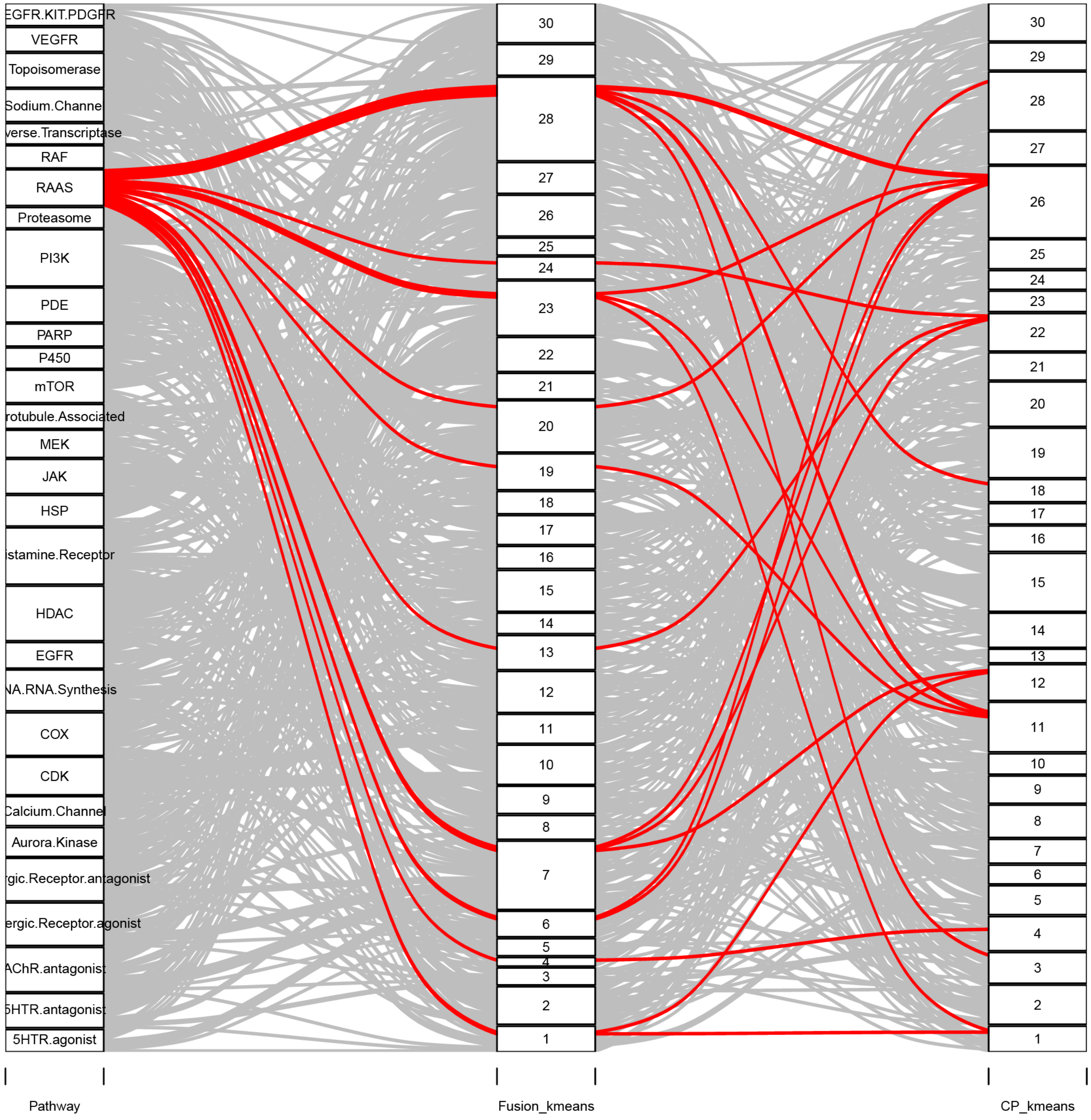
EGFR.KIT.PDGFR
VEGFR
Topoisomerase
Sodium.Channel
verse.Transcriptase
RAF
RAAS
Proteasome
PI3K
PDE
PARP
P450
mTOR
rotubule.Associated
MEK
JAK
HSP
istamine.Receptor
HDAC
EGFR
NA.RNA.Synthesis
COX
CDK
Calcium.Channel
Aurora.Kinase
rgic.Receptor.antagonist
ergic.Receptor.agonist
AChR.antagonist
5HTR.antagonist
5HTR.agonist
30
29
28
27
26
25
24
23
22
21
20
19
18
17
16
15
14
13
12
11
10
9
8
7
6
5
4
3
2
1
Pathway
Fusion_kmeans
CP_kmeans

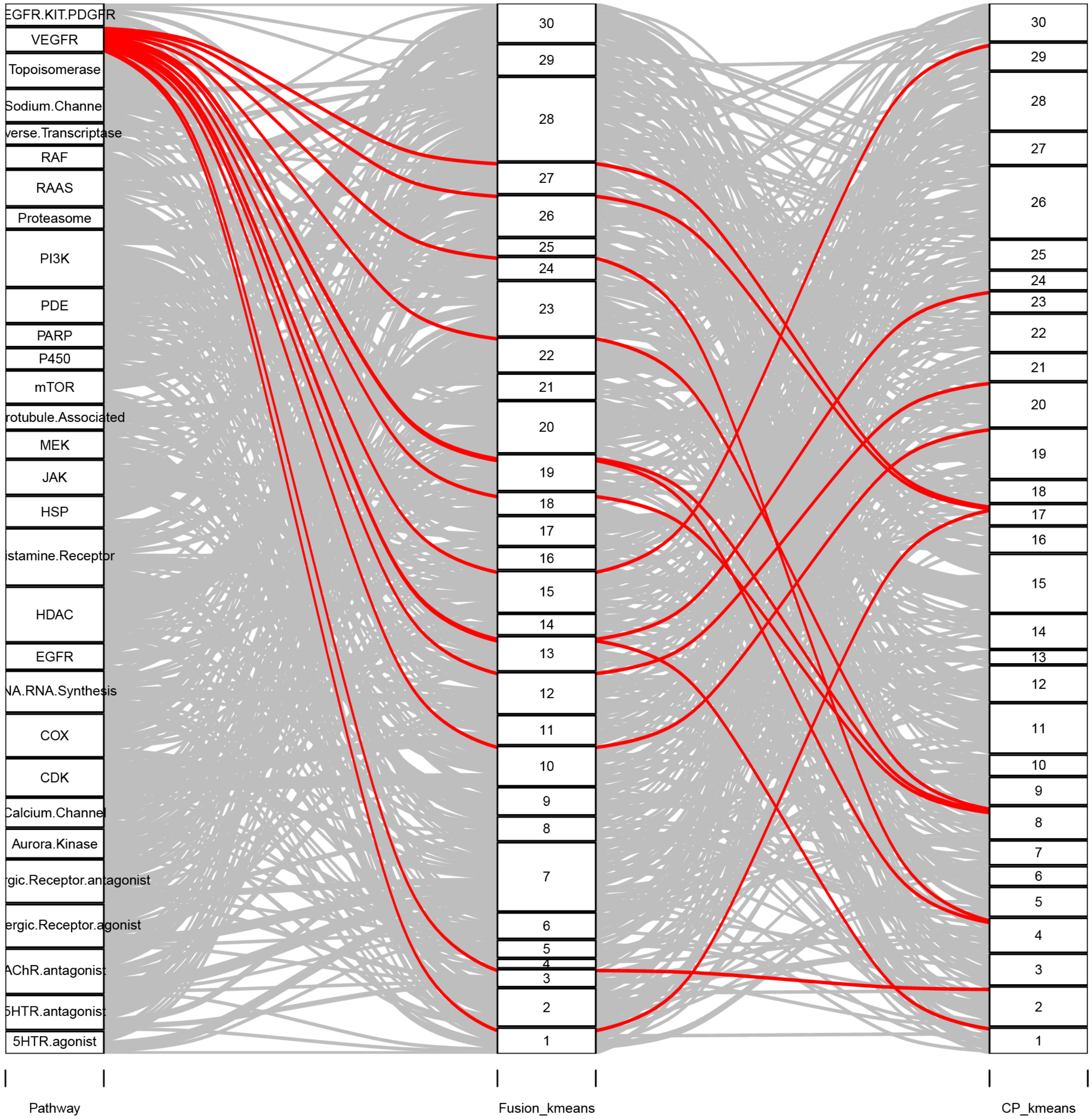
EGFR.KIT.PDGFR
VEGFR
Topoisomerase
Sodium.Channel
verse.Transcriptase
RAF
RAAS
Proteasome
PI3K
PDE
PARP
P450
mTOR
rotubule.Associated
MEK
JAK
HSP
istamine.Receptor
HDAC
EGFR
NA.RNA.Synthesis
COX
CDK
Calcium.Channel
Aurora.Kinase
rgic.Receptor.antagonist
ergic.Receptor.agonist
AChR.antagonist
5HTR.antagonist
5HTR.agonist
Pathway
Fusion_kmeans
CP_kmeans

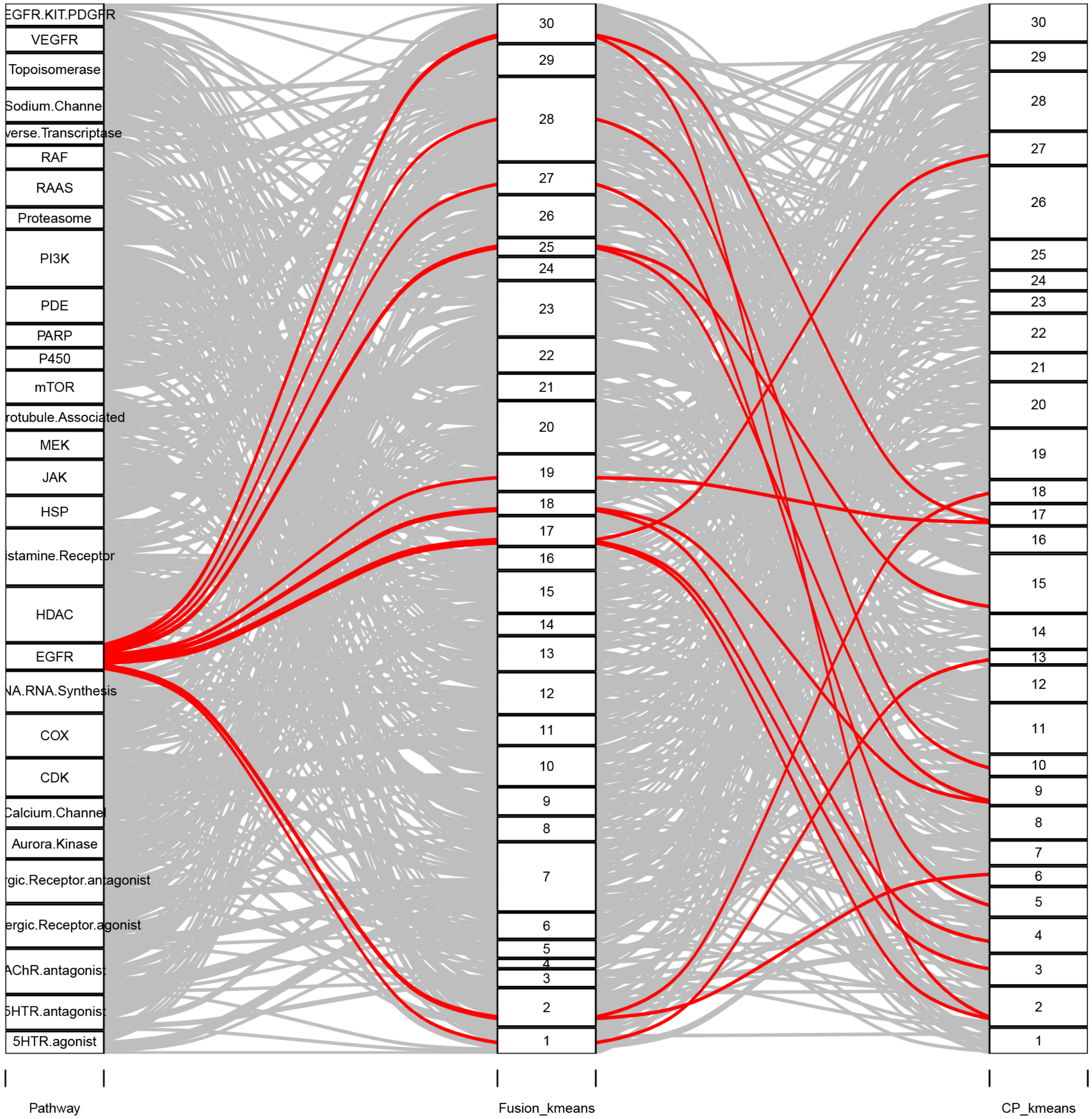

GFR.KIT.PDGFR
VEGFR
Topoisomerase
Sodium.Channel
verse.Transcriptase
RAF
RAAS
Proteasome
PI3K
PDE
PARP
P450
mTOR
rotubule.Associated
MEK
JAK
HSP
istamine.Receptor
HDAC
EGFR
NA.RNA.Synthesis
COX
CDK
Calcium.Channel
Aurora.Kinase
rgic.Receptor.antagonist
ergic.Receptor.agonist
AChR.antagonist
5HTR.antagonist
5HTR.agonist
30
29
28
27
26
25
24
23
22
21
20
19
18
17
16
15
14
13
12
11
10
9
8
7
6
5
4
3
2
1
Pathway
Fusion_kmeans
CP_kmeans

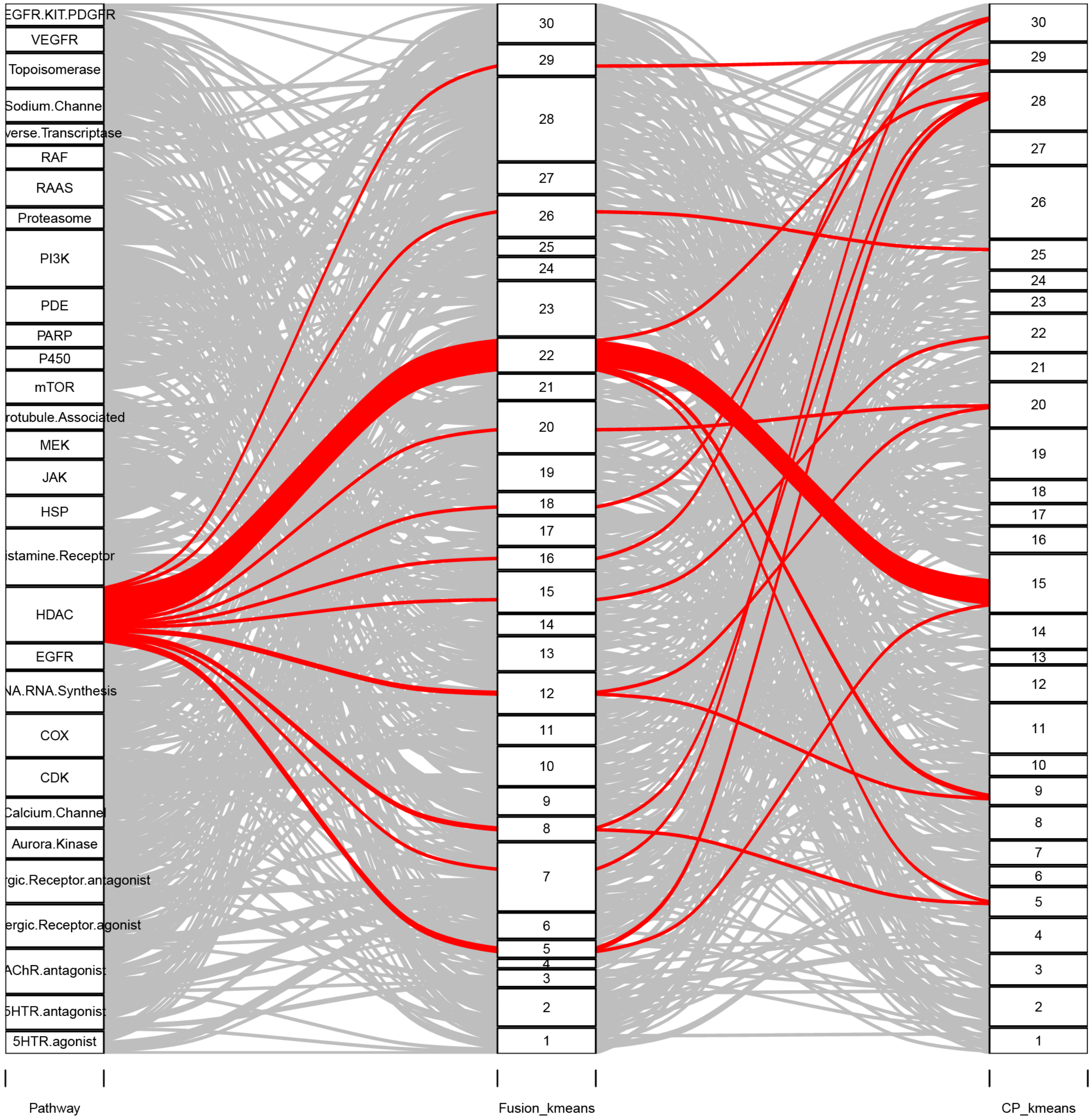
EGFR.KIT.PDGFR
VEGFR
Topoisomerase
Sodium.Channel
verse.Transcriptase
RAF
RAAS
Proteasome
PI3K
PDE
PARP
P450
mTOR
rotubule.Associated
MEK
JAK
HSP
istamine.Receptor
HDAC
EGFR
NA.RNA.Synthesis
COX
CDK
Calcium.Channel
Aurora.Kinase
rgic.Receptor.antagonist
ergic.Receptor.agonist
AChR.antagonist
5HTR.antagonist
5HTR.agonist
30 29 28 27 26 25 24 23 22 21 20 19 18 17 16 15 14 13 12 11 10 9 8 7 6 5 4 3 2 1
30 29 28 27 26 25 24 23 22 21 20 19 18 17 16 15 14 13 12 11 10 9 8 7 6 5 4 3 2 1
Pathway
Fusion_kmeans
CP_kmeans

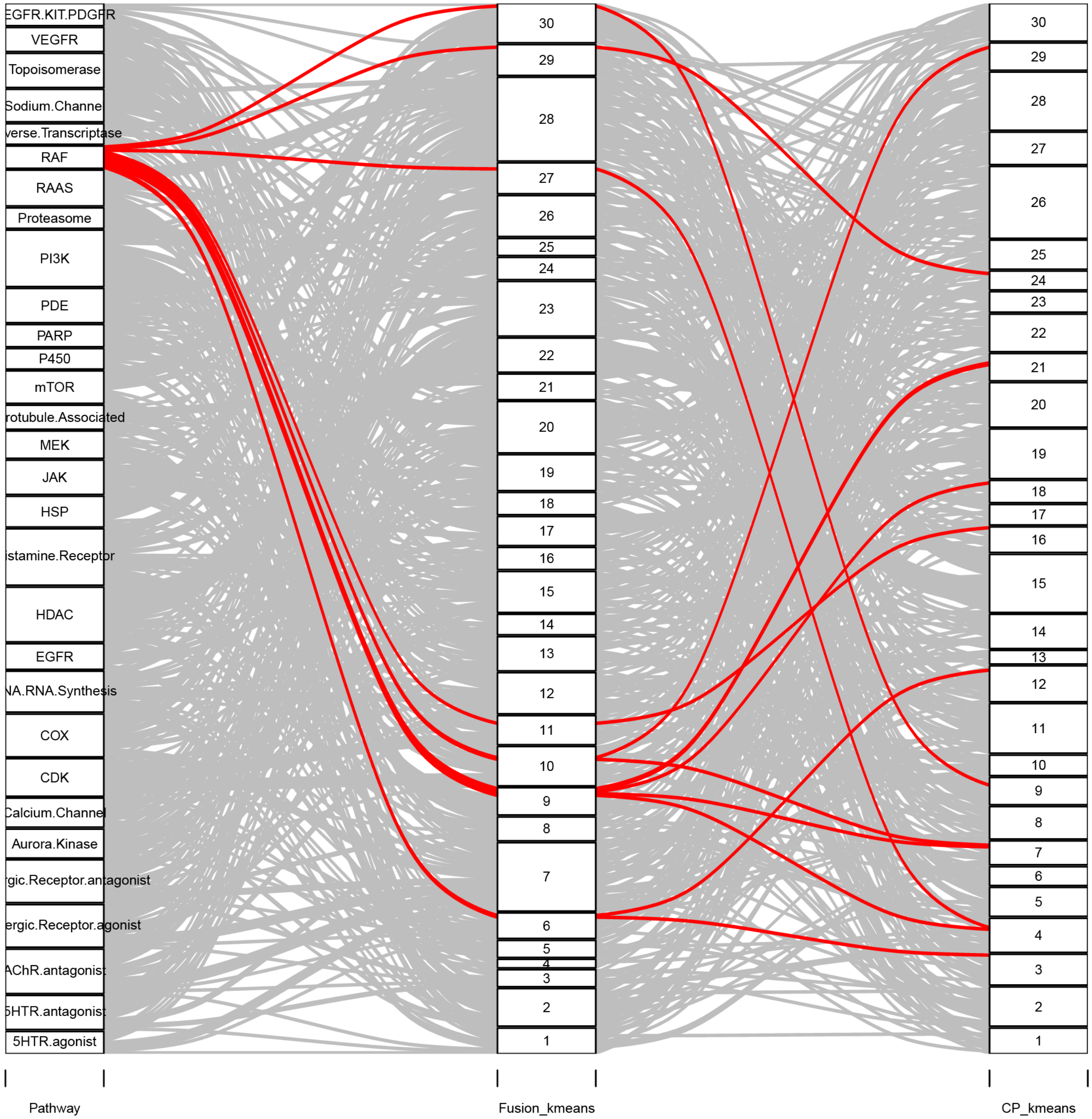
EGFR.KIT.PDGFR
VEGFR
Topoisomerase
Sodium.Channel
verse.Transcriptase
RAF
RAAS
Proteasome
PI3K
PDE
PARP
P450
mTOR
rotubule.Associated
MEK
JAK
HSP
istamine.Receptor
HDAC
EGFR
NA.RNA.Synthesis
COX
CDK
Calcium.Channel
Aurora.Kinase
rgic.Receptor.antagonist
ergic.Receptor.agonist
AChR.antagonist
5HTR.antagonist
5HTR.agonist
30
29
28
27
26
25
24
23
22
21
20
19
18
17
16
15
14
13
12
11
10
9
8
7
6
5
4
3
2
1
Pathway
Fusion_kmeans
CP_kmeans

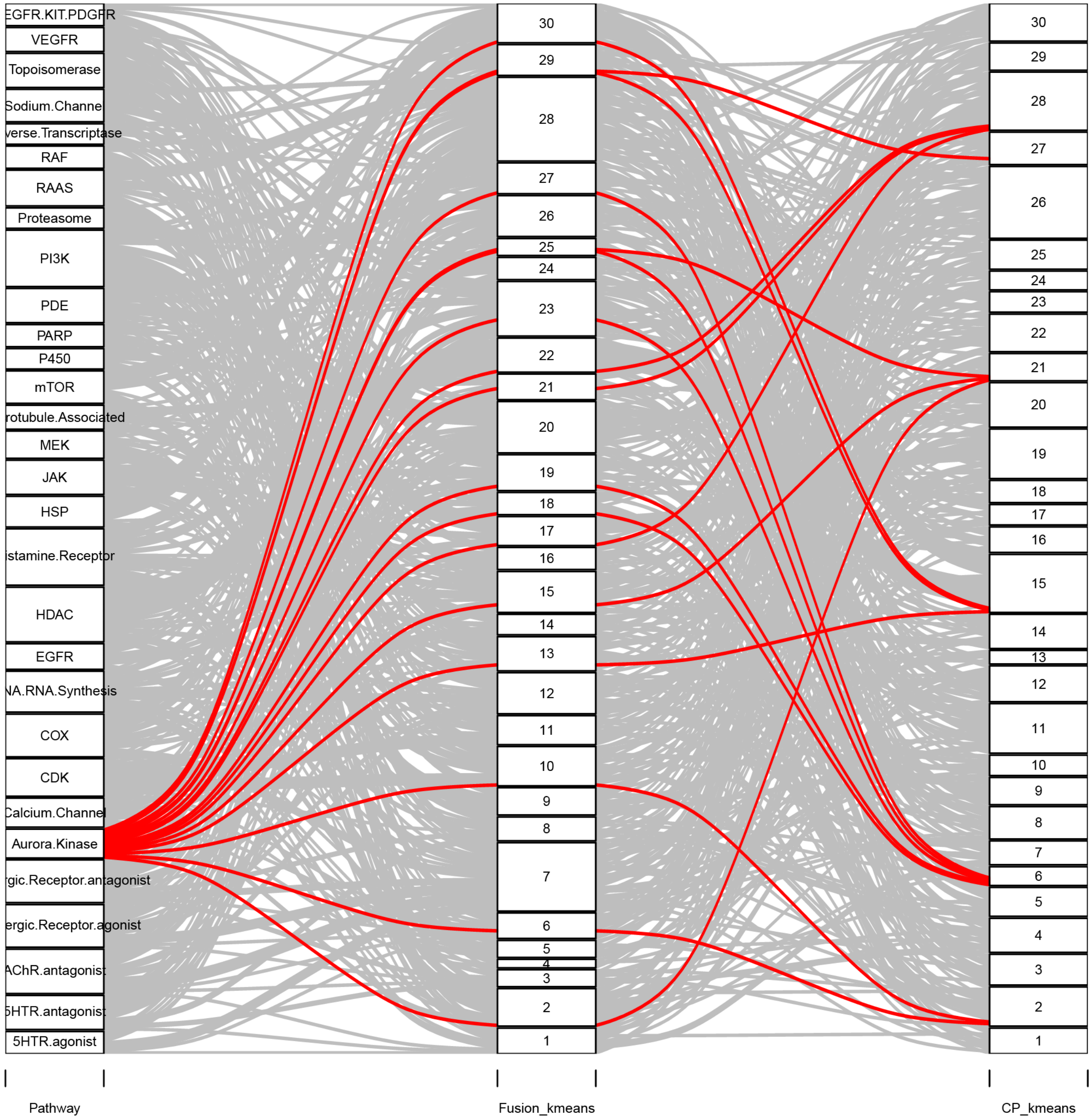
EGFR.KIT.PDGFR
VEGFR
Topoisomerase
Sodium.Channel
verse.Transcriptase
RAF
RAAS
Proteasome
PI3K
PDE
PARP
P450
mTOR
rotubule.Associated
MEK
JAK
HSP
istamine.Receptor
HDAC
EGFR
NA.RNA.Synthesis
COX
CDK
Calcium.Channel
Aurora.Kinase
rgic.Receptor.antagonist
ergic.Receptor.agonist
AChR.antagonist
5HTR.antagonist
5HTR.agonist
30
29
28
27
26
25
24
23
22
21
20
19
18
17
16
15
14
13
12
11
10
9
8
7
6
5
4
3
2
1
Pathway
Fusion_kmeans
CP_kmeans

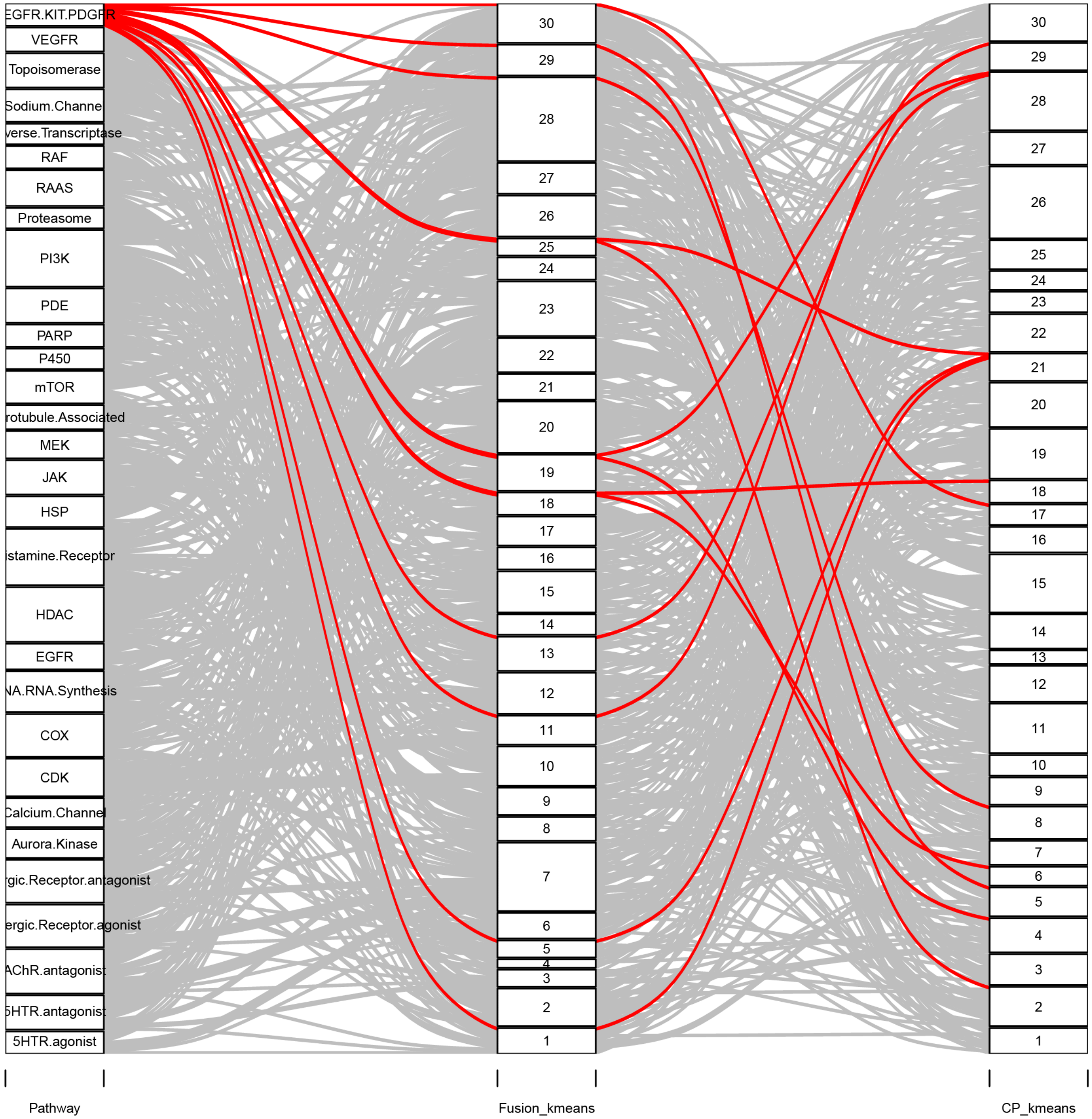
EGFR.KIT.PDGFR
VEGFR
Topoisomerase
Sodium.Channe
verse.Transcriptase
RAF
RAAS
Proteasome
PI3K
PDE
PARP
P450
mTOR
rotubule.Associated
MEK
JAK
HSP
istamine.Receptor
HDAC
EGFR
NA.RNA.Synthesis
COX
CDK
Calcium.Channel
Aurora.Kinase
rgic.Receptor.antagonist
ergic.Receptor.agonist
AChR.antagonist
5HTR.antagonist
5HTR.agonist
30
29
28
27
26
25
24
23
22
21
20
19
18
17
16
15
14
13
12
11
10
9
8
7
6
5
4
3
2
1
30
29
28
27
26
25
24
23
22
21
20
19
18
17
16
15
14
13
12
11
10
9
8
7
6
5
4
3
2
1
Pathway
Fusion_kmeans
CP_kmeans

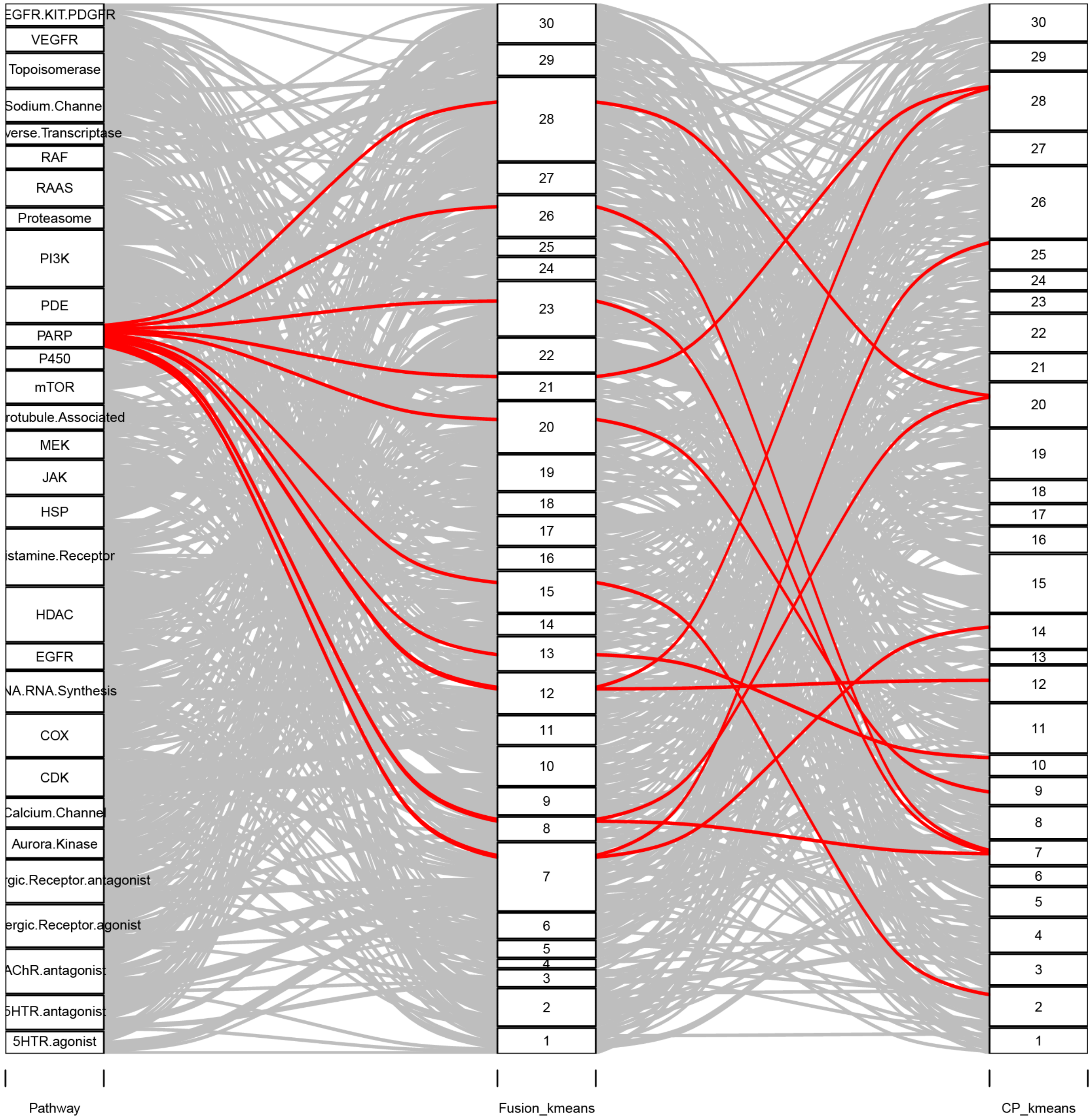
EGFR.KIT.PDGFR
VEGFR
Topoisomerase
Sodium.Channel
verse.Transcriptase
RAF
RAAS
Proteasome
PI3K
PDE
PARP
P450
mTOR
rotubule.Associated
MEK
JAK
HSP
istamine.Receptor
HDAC
EGFR
NA.RNA.Synthesis
COX
CDK
Calcium.Channel
Aurora.Kinase
rgic.Receptor.antagonist
ergic.Receptor.agonist
AChR.antagonist
5HTR.antagonist
5HTR.agonist
30 29 28 27 26 25 24 23 22 21 20 19 18 17 16 15 14 13 12 11 10 9 8 7 6 5 4 3 2 1
30 29 28 27 26 25 24 23 22 21 20 19 18 17 16 15 14 13 12 11 10 9 8 7 6 5 4 3 2 1
Pathway
Fusion_kmeans
CP_kmeans

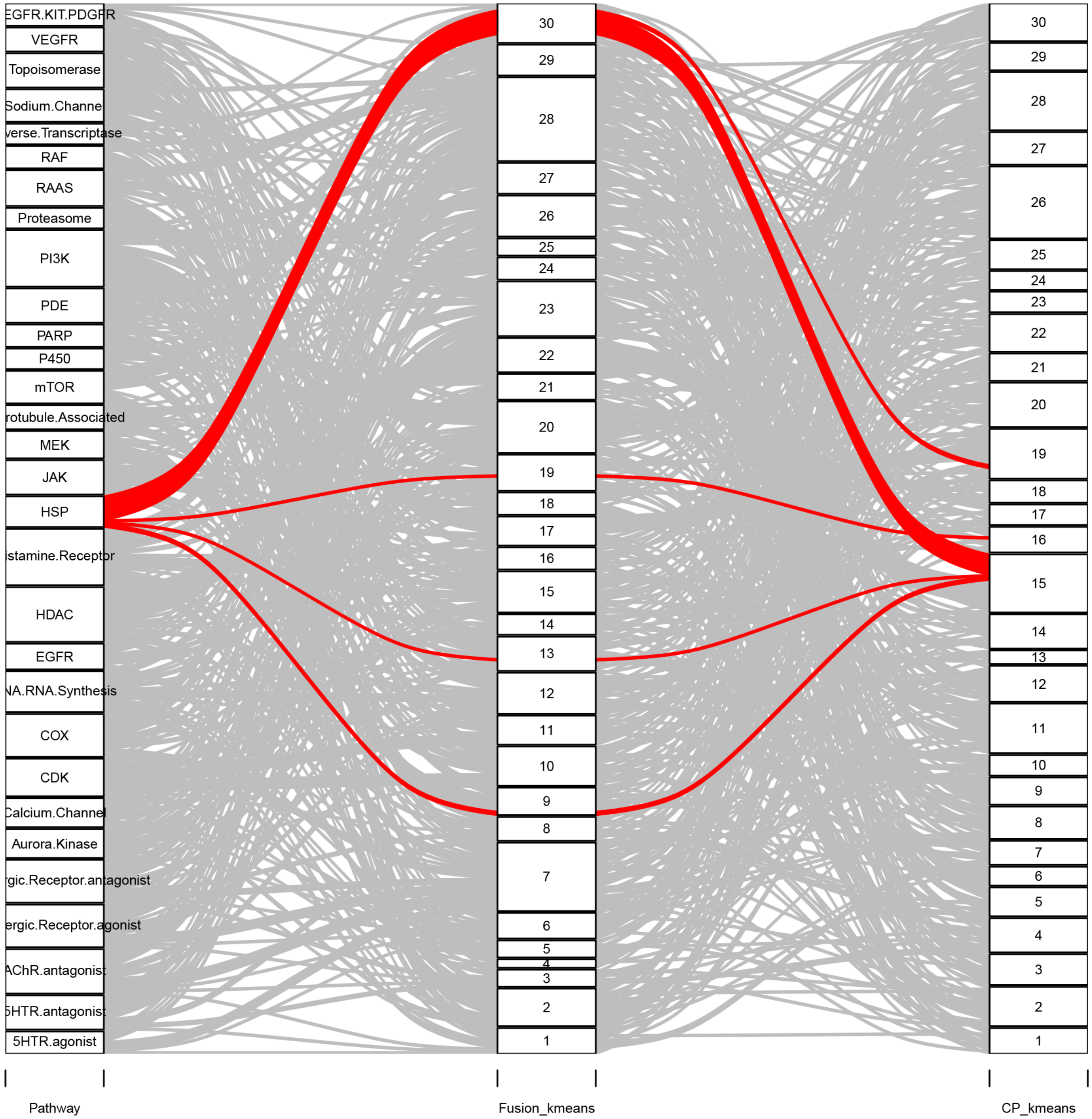
EGFR.KIT.PDGFR
VEGFR
Topoisomerase
Sodium.Channel
verse.Transcriptase
RAF
RAAS
Proteasome
PI3K
PDE
PARP
P450
mTOR
rotubule.Associated
MEK
JAK
HSP
istamine.Receptor
HDAC
EGFR
NA.RNA.Synthesis
COX
CDK
Calcium.Channel
Aurora.Kinase
rgic.Receptor.antagonist
ergic.Receptor.agonist
AChR.antagonist
5HTR.antagonist
5HTR.agonist
30
29
28
27
26
25
24
23
22
21
20
19
18
17
16
15
14
13
12
11
10
9
8
7
6
5
4
3
2
1
Pathway
Fusion_kmeans
CP_kmeans

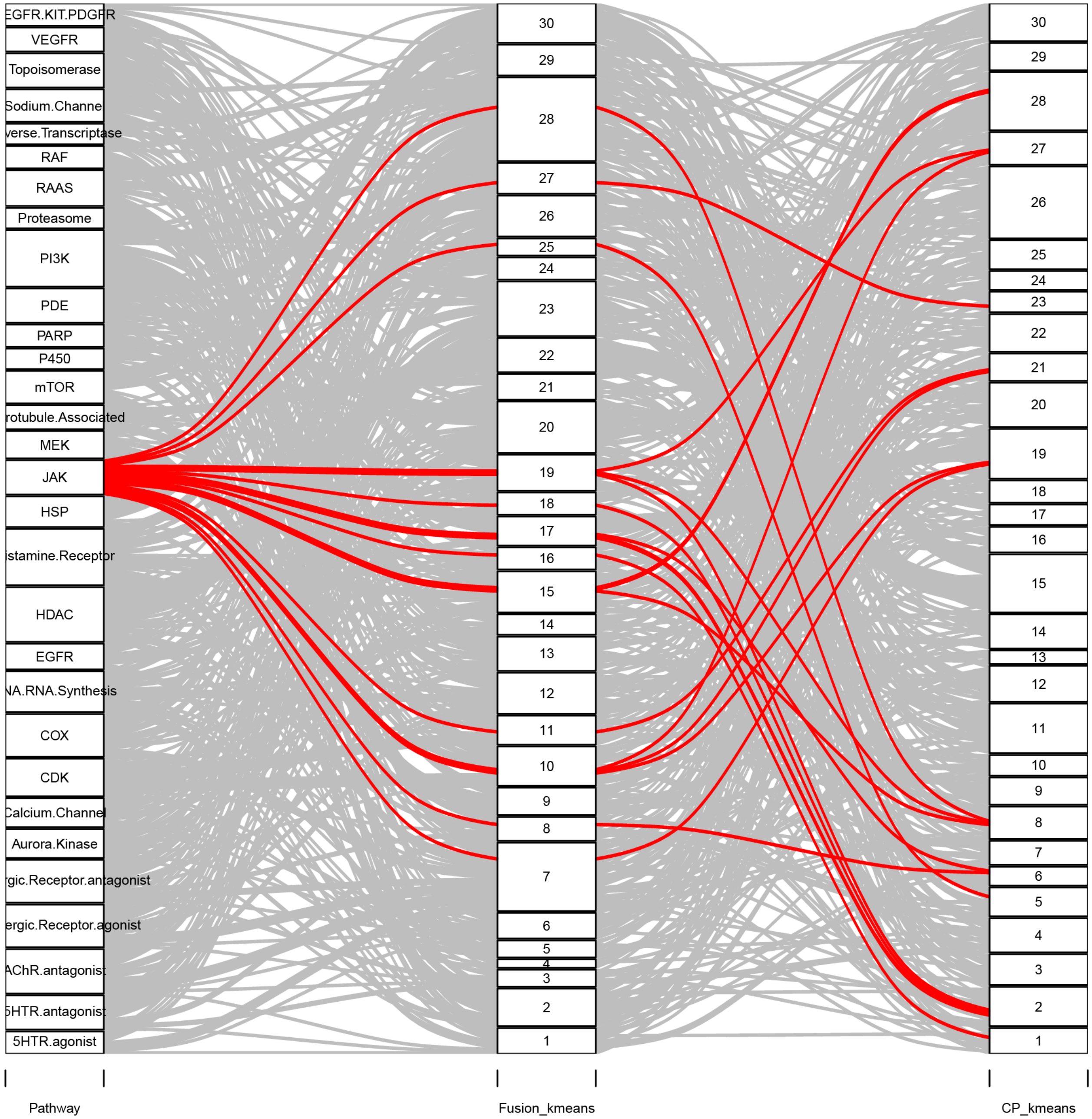
EGFR.KIT.PDGFR
VEGFR
Topoisomerase
Sodium.Channel
verse.Transcriptase
RAF
RAAS
Proteasome
PI3K
PDE
PARP
P450
mTOR
rotubule.Associated
MEK
JAK
HSP
istamine.Receptor
HDAC
EGFR
NA.RNA.Synthesis
COX
CDK
Calcium.Channel
Aurora.Kinase
rgic.Receptor.antagonist
ergic.Receptor.agonist
AChR.antagonist
5HTR.antagonist
5HTR.agonist
Pathway
Fusion_kmeans
CP_kmeans

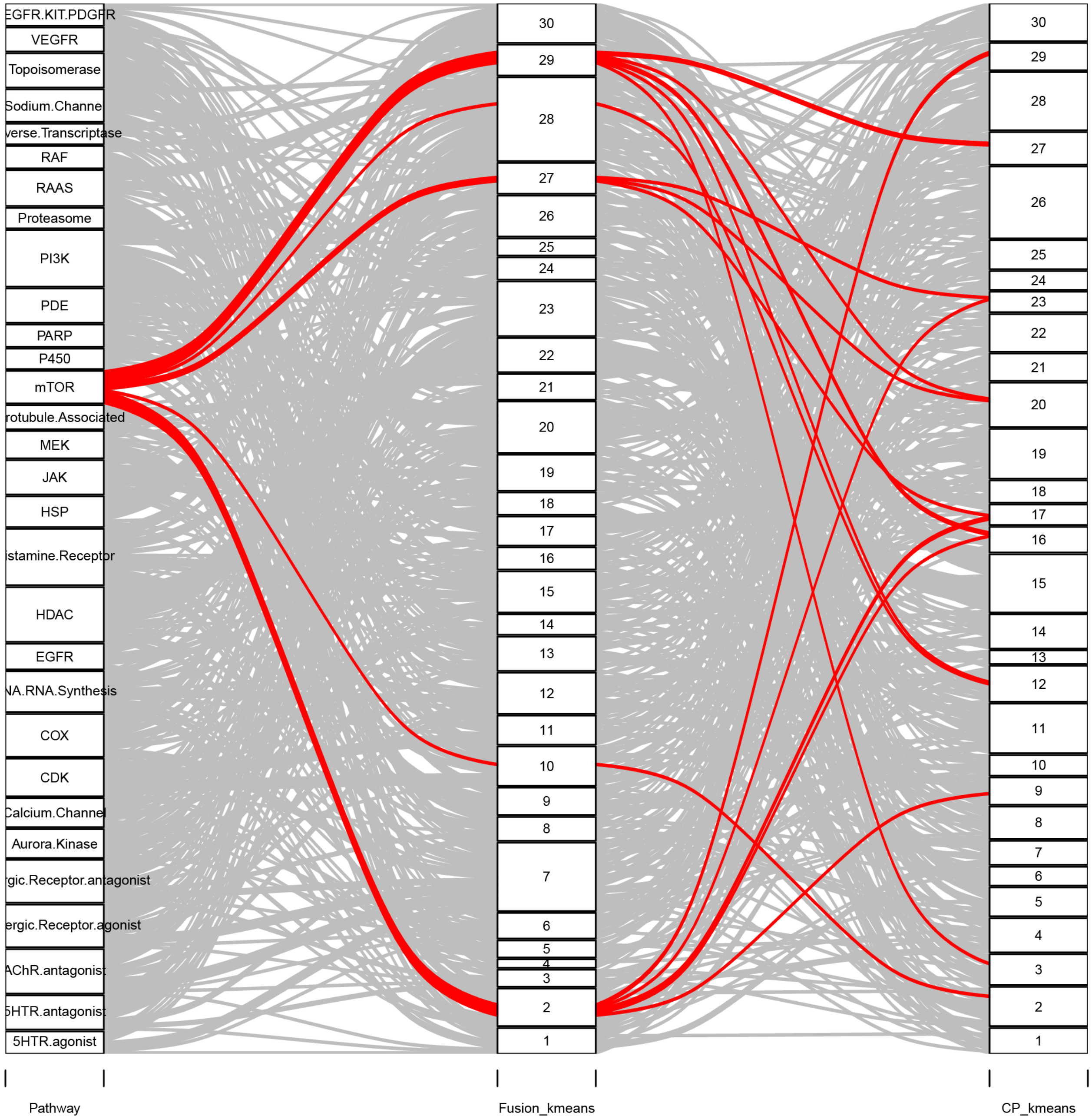

EGFR.KIT.PDGFR
VEGFR
Topoisomerase
Sodium.Channel
verse.Transcriptase
RAF
RAAS
Proteasome
PI3K
PDE
PARP
P450
mTOR
rotubule.Associated
MEK
JAK
HSP
istamine.Receptor
HDAC
EGFR
NA.RNA.Synthesis
COX
CDK
Calcium.Channel
Aurora.Kinase
rgic.Receptor.antagonist
ergic.Receptor.agonist
AChR.antagonist
5HTR.antagonist
5HTR.agonist
Pathway
Fusion_kmeans
CP_kmeans

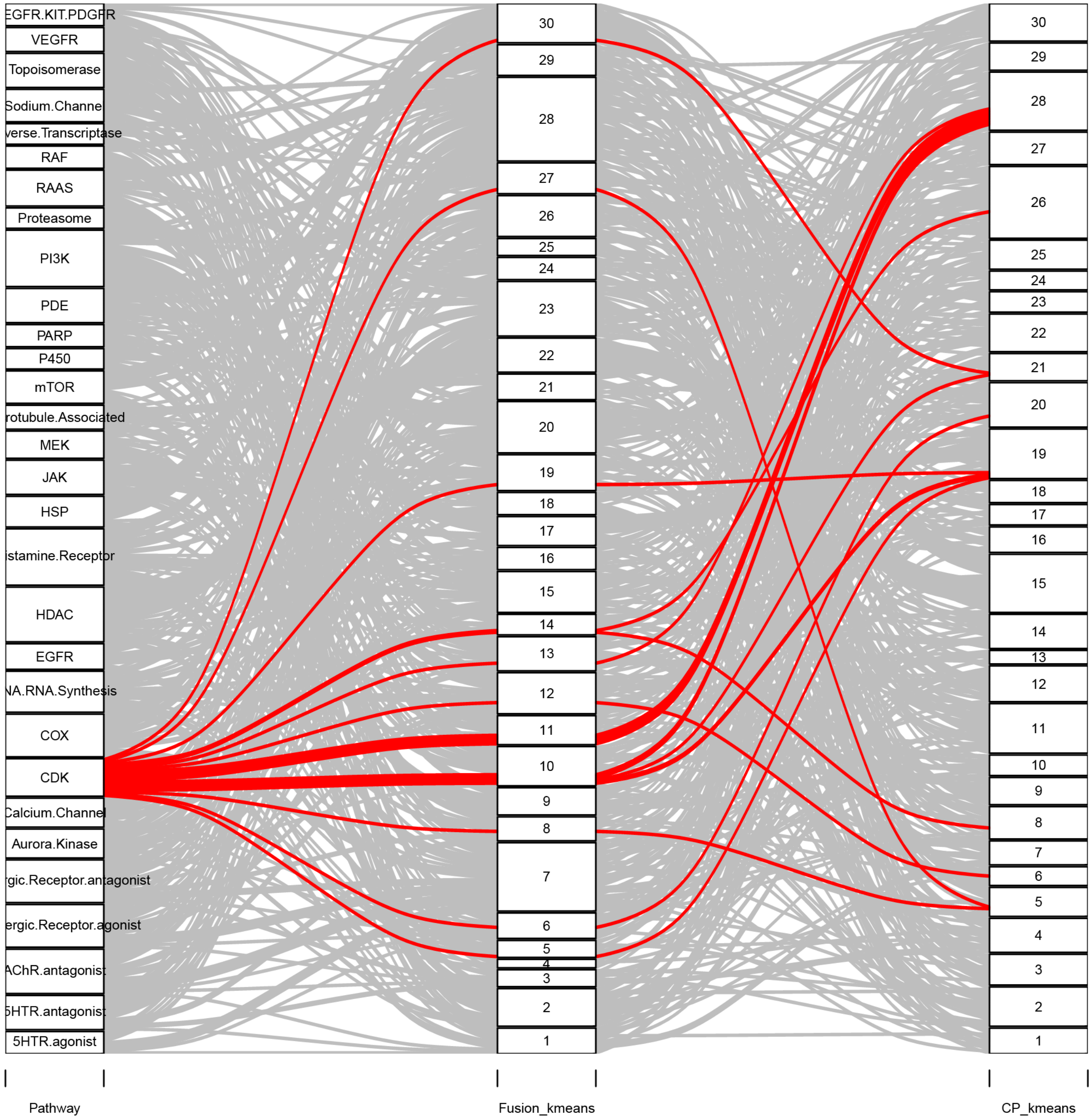
EGFR.KIT.PDGFR
VEGFR
Topoisomerase
Sodium.Channel
verse.Transcriptase
RAF
RAAS
Proteasome
PI3K
PDE
PARP
P450
mTOR
rotubule.Associated
MEK
JAK
HSP
istamine.Receptor
HDAC
EGFR
NA.RNA.Synthesis
COX
CDK
Calcium.Channel
Aurora.Kinase
rgic.Receptor.antagonist
ergic.Receptor.agonist
AChR.antagonist
5HTR.antagonist
5HTR.agonist
Pathway
Fusion_kmeans
CP_kmeans

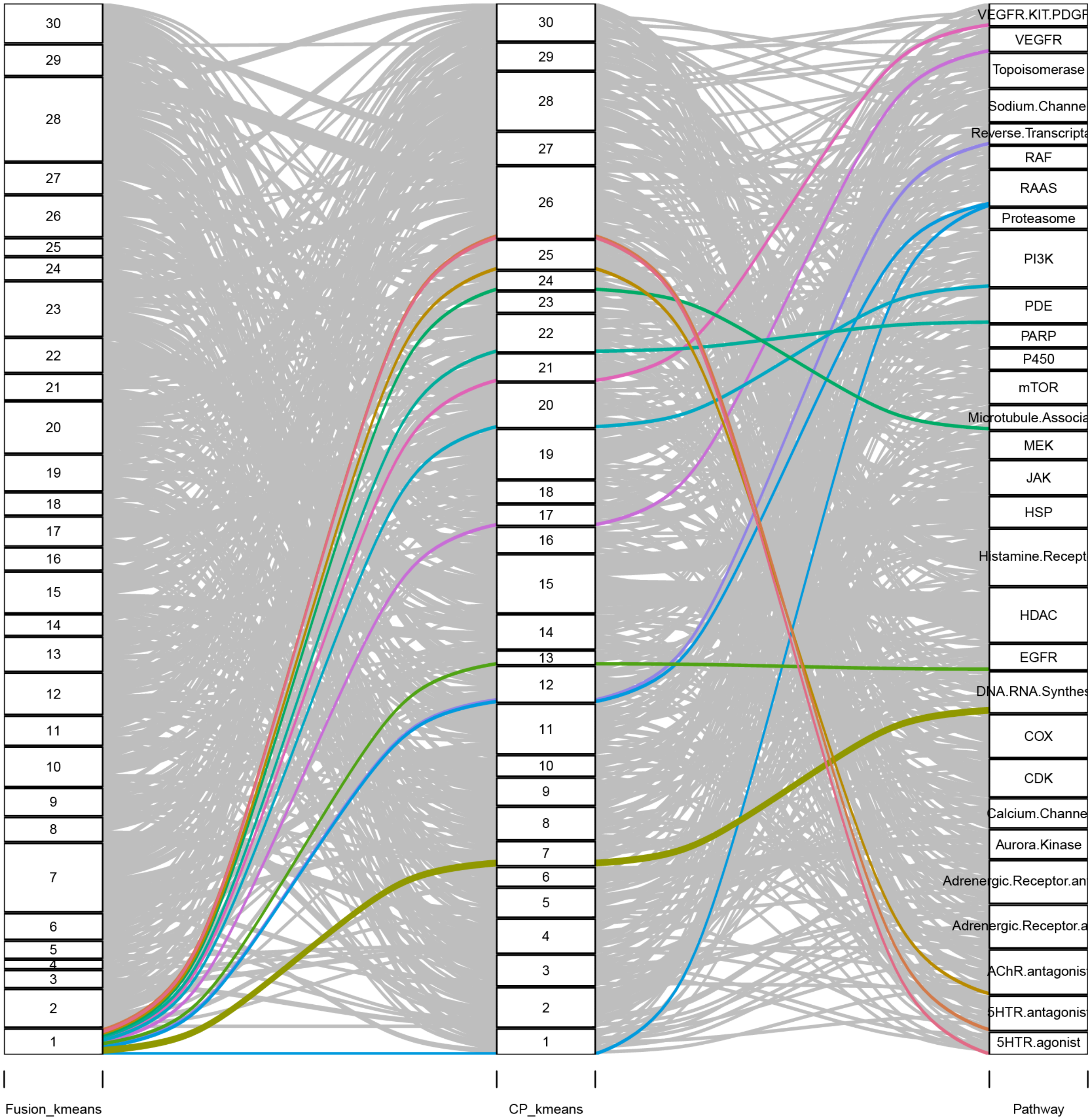

Supplementary Figure 6. Alluvial diagrams for each cluster in FUSION and each cluster in CP. Alluvial diagrams for each k-means cluster in the FUSION (A) and CP (B) datasets. Comparison of target class associations using k-means clustering (k=30) of FUSION and CP signatures for the top 30 target classes in the Selleck chemical library. Each line represents a compound, and each panel highlights a different cluster. Chemicals belonging to different target classes are indicated by different colors.

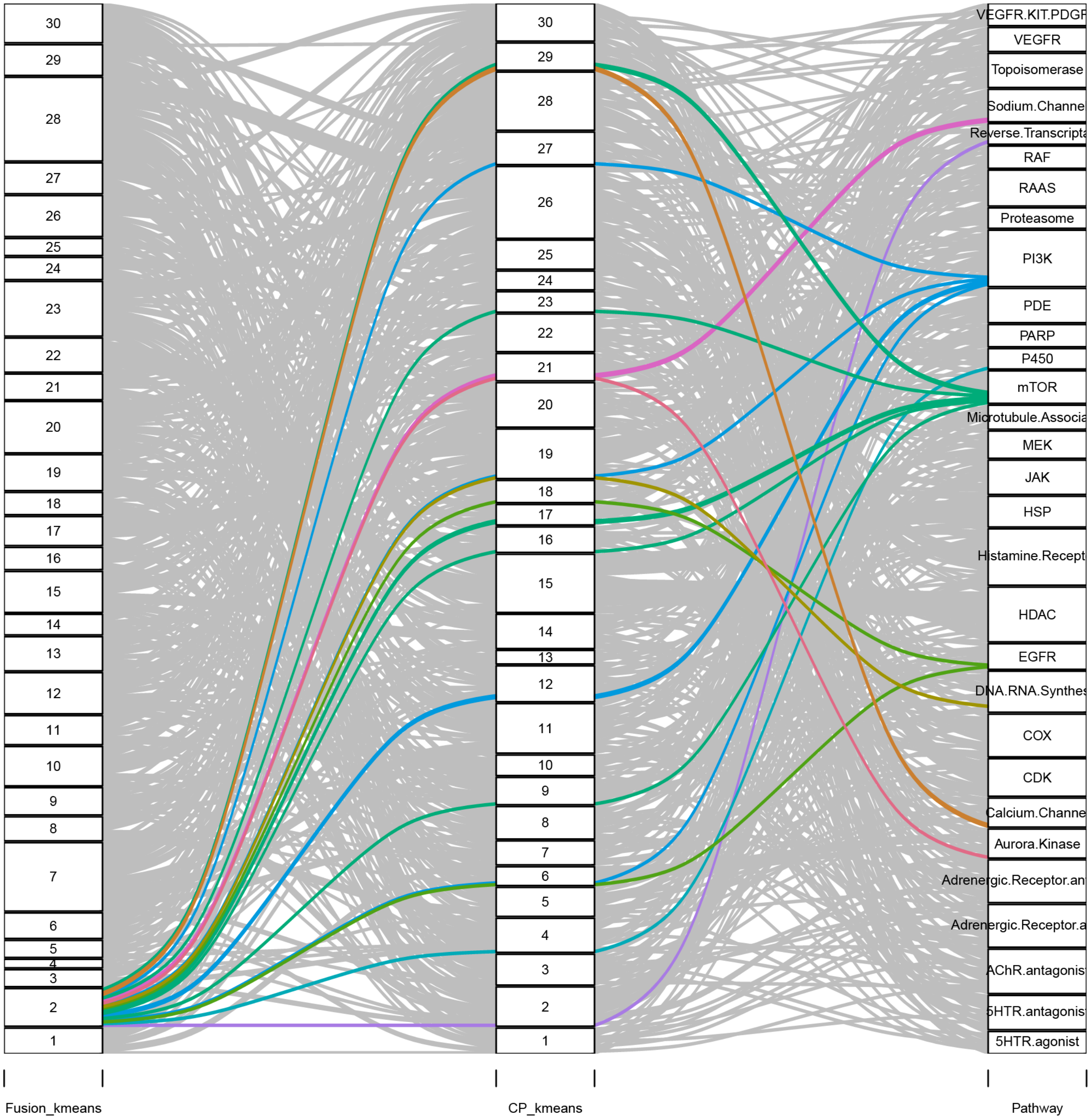

Fusion_kmeans
CP_kmeans
Pathway
VEGFR.KIT.PDGF
VEGFR
Topoisomerase
Sodium.Channel
Reverse.Transcripta
RAF
RAAS
Proteasome
PI3K
PDE
PARP
P450
mTOR
Microtubule.Associa
MEK
JAK
HSP
Histamine.Recept
HDAC
EGFR
DNA.RNA.Synthes
COX
CDK
Calcium.Channe
Aurora.Kinase
Adrenergic.Receptor.an
Adrenergic.Receptor.a
AChR.antagonis
5HTR.antagonis
5HTR.agonist

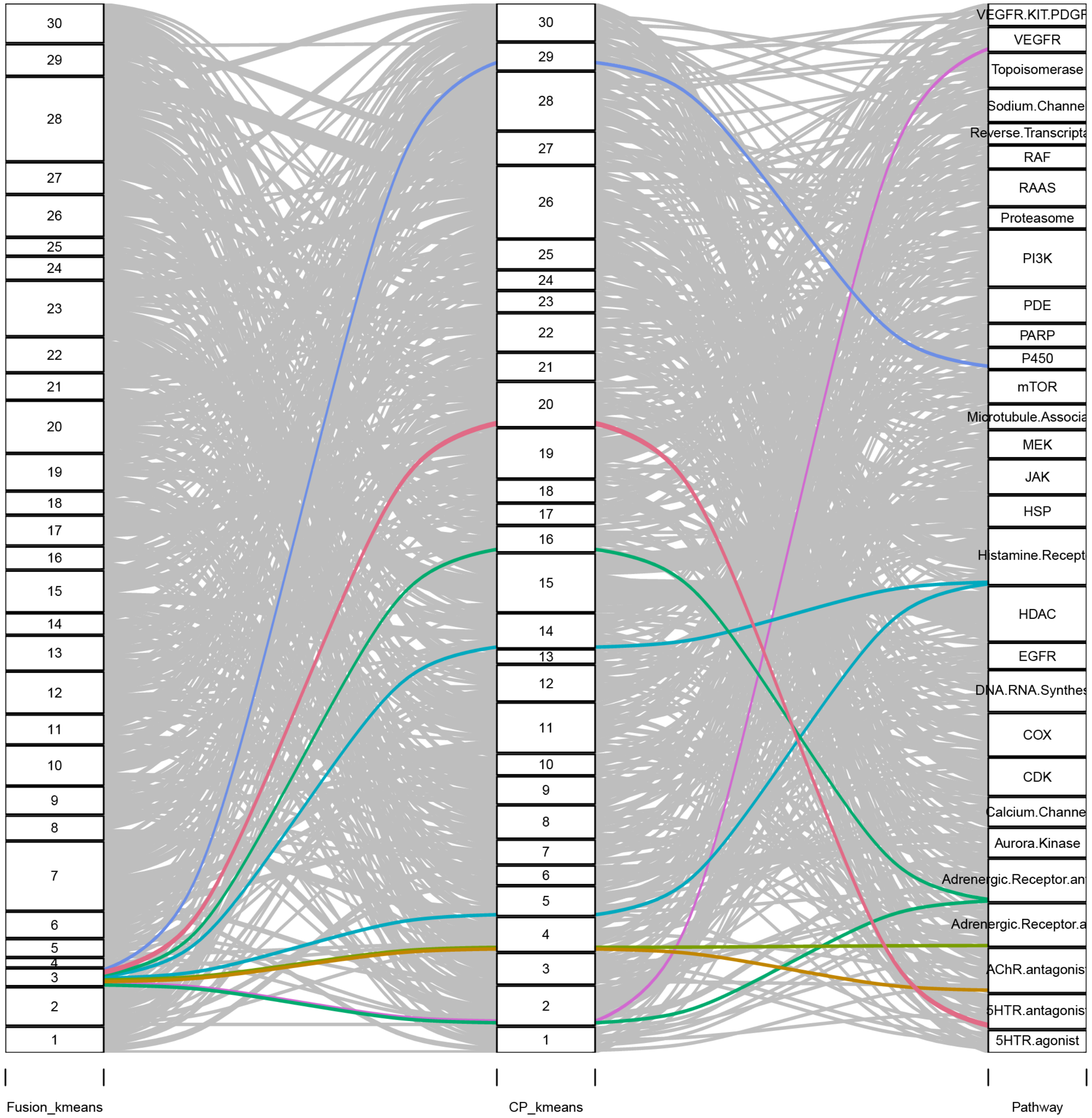
Fusion_kmeans
CP_kmeans
Pathway
30
29
28
27
26
25
24
23
22
21
20
19
18
17
16
15
14
13
12
11
10
9
8
7
6
5
4
3
2
1
VEGFR.KIT.PDGF
VEGFR
Topoisomerase
Sodium.Channel
Reverse.Transcripta
RAF
RAAS
Proteasome
PI3K
PDE
PARP
P450
mTOR
Microtubule.Associa
MEK
JAK
HSP
Histamine.Recept
HDAC
EGFR
DNA.RNA.Synthes
COX
CDK
Calcium.Channe
Aurora.Kinase
Adrenergic.Receptor.an
Adrenergic.Receptor.a
AChR.antagonis
5HTR.antagonis
5HTR.agonist

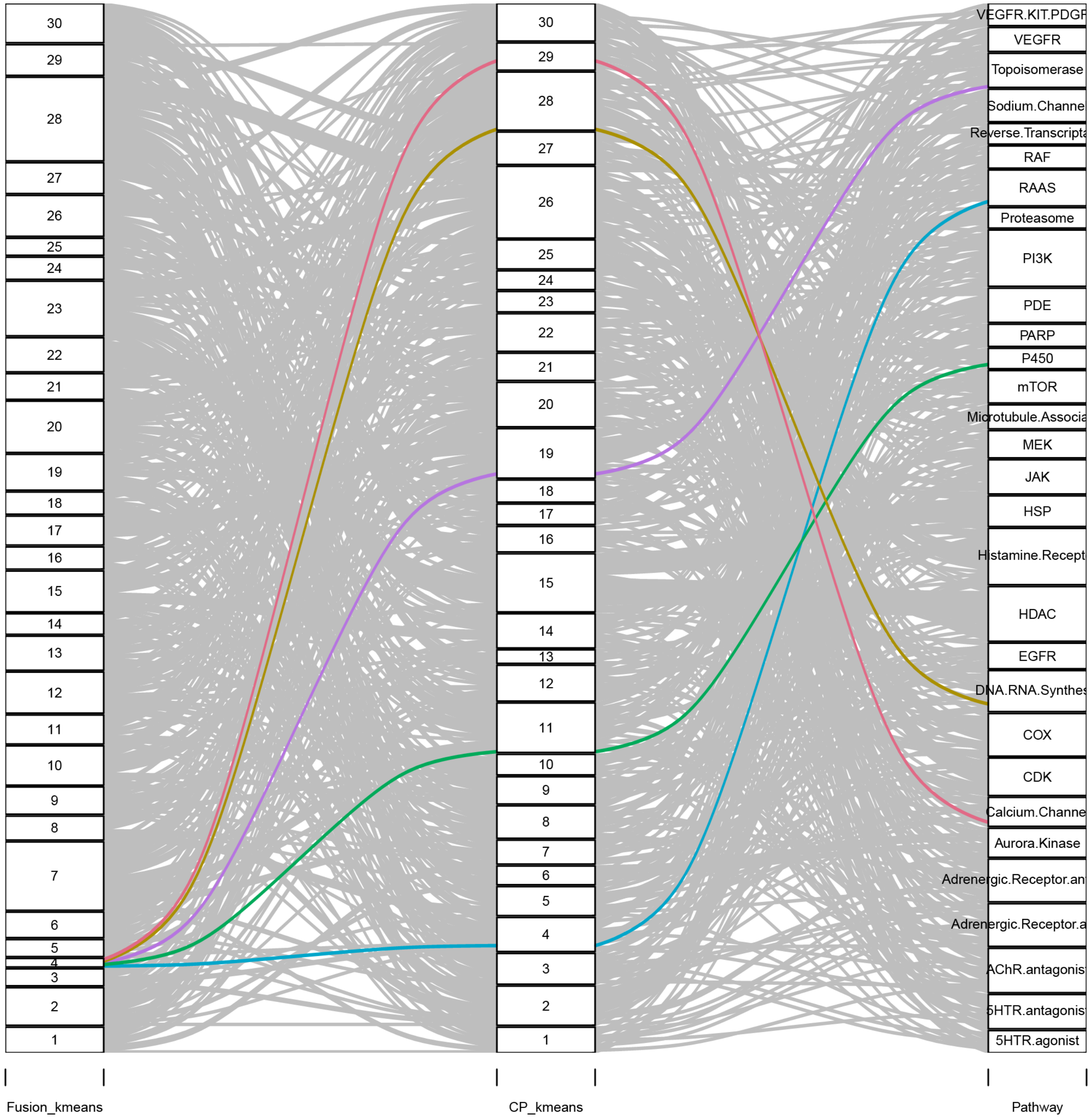
30
29
28
27
26
25
24
23
22
21
20
19
18
17
16
15
14
13
12
11
10
9
8
7
6
5
4
3
2
1
VEGFR.KIT.PDGF
VEGFR
Topoisomerase
Sodium.Channel
Reverse.Transcripta
RAF
RAAS
Proteasome
PI3K
PDE
PARP
P450
mTOR
Microtubule.Associa
MEK
JAK
HSP
Histamine.Recept
HDAC
EGFR
DNA.RNA.Synthes
COX
CDK
Calcium.Channe
Aurora.Kinase
Adrenergic.Receptor.an
Adrenergic.Receptor.a
AChR.antagonis
5HTR.antagonis
5HTR.agonist
Fusion_kmeans
CP_kmeans
Pathway

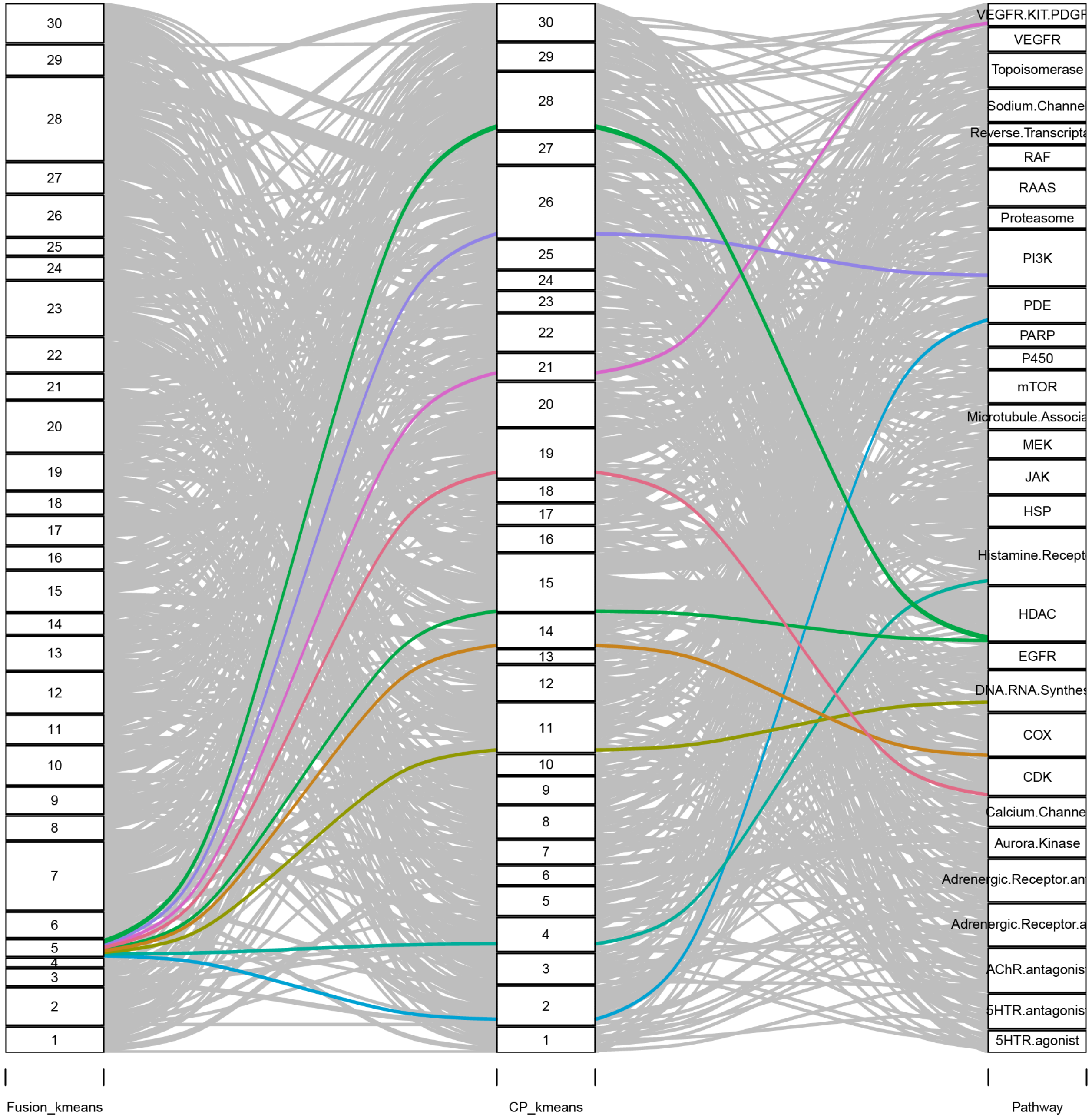
30
29
28
27
26
25
24
23
22
21
20
19
18
17
16
15
14
13
12
11
10
9
8
7
6
5
4
3
2
1
VEGFR.KIT.PDGF
VEGFR
Topoisomerase
Sodium.Channel
Reverse.Transcripta
RAF
RAAS
Proteasome
PI3K
PDE
PARP
P450
mTOR
Microtubule.Associa
MEK
JAK
HSP
Histamine.Recept
HDAC
EGFR
DNA.RNA.Synthes
COX
CDK
Calcium.Channe
Aurora.Kinase
Adrenergic.Receptor.an
Adrenergic.Receptor.a
AChR.antagonis
5HTR.antagonis
5HTR.agonist
Fusion_kmeans
CP_kmeans
Pathway

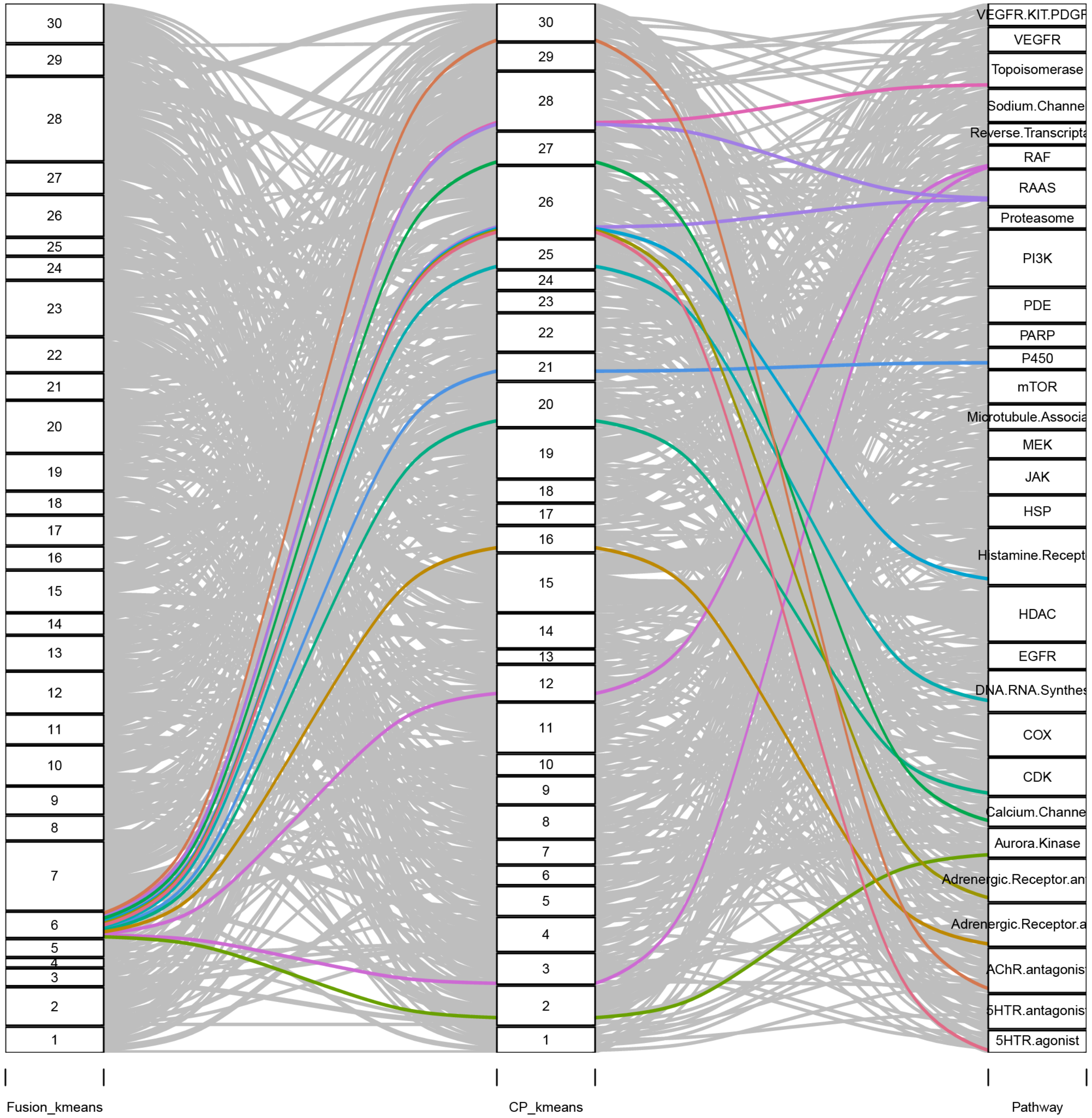
Fusion_kmeans
CP_kmeans
Pathway
30
29
28
27
26
25
24
23
22
21
20
19
18
17
16
15
14
13
12
11
10
9
8
7
6
5
4
3
2
1
VEGFR.KIT.PDGF
VEGFR
Topoisomerase
Sodium.Channel
Reverse.Transcripta
RAF
RAAS
Proteasome
PI3K
PDE
PARP
P450
mTOR
Microtubule.Associa
MEK
JAK
HSP
Histamine.Recept
HDAC
EGFR
DNA.RNA.Synthes
COX
CDK
Calcium.Channe
Aurora.Kinase
Adrenergic.Receptor.an
Adrenergic.Receptor.a
AChR.antagonis
5HTR.antagonis
5HTR.agonist

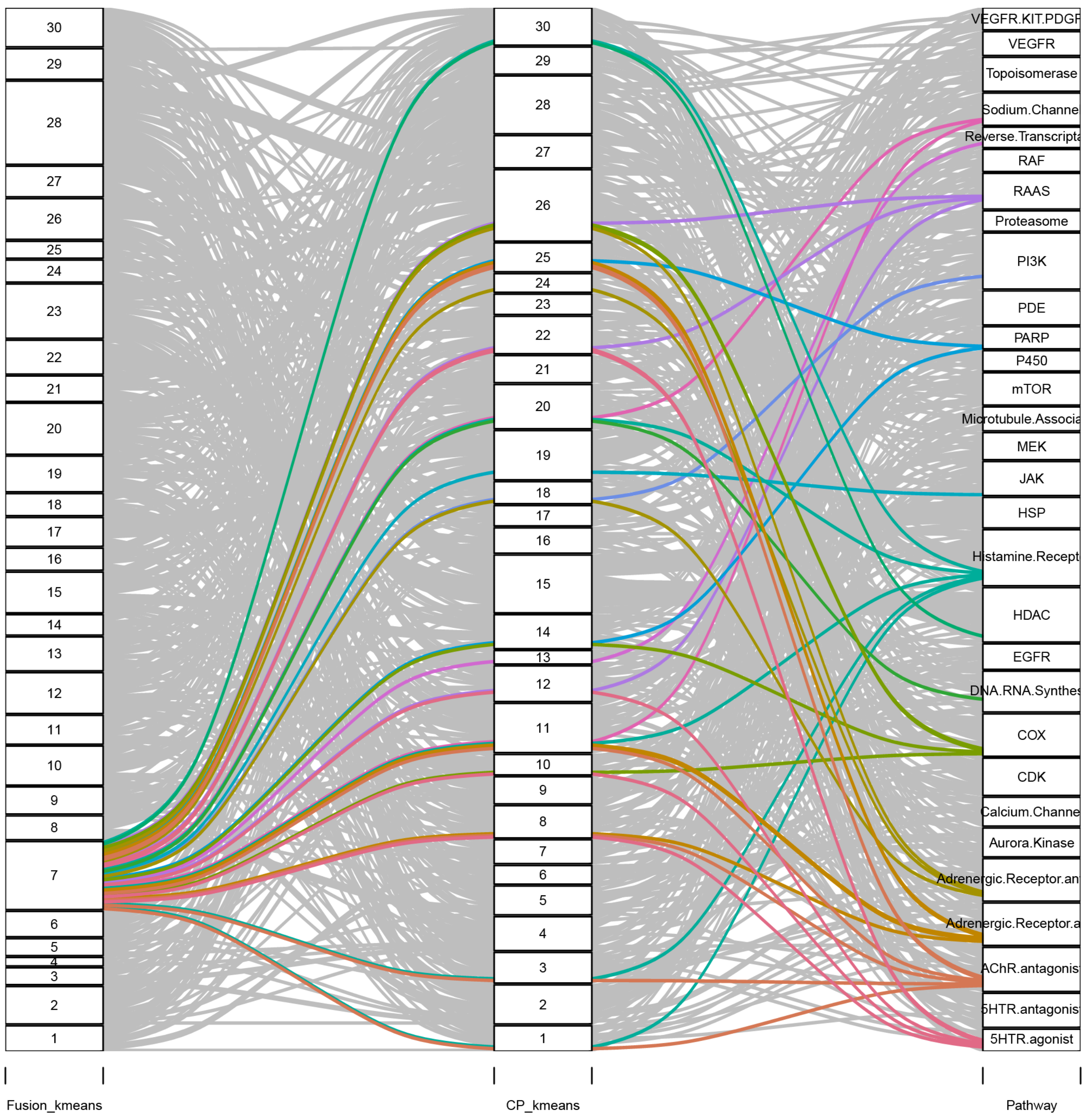
30
29
28
27
26
25
24
23
22
21
20
19
18
17
16
15
14
13
12
11
10
9
8
7
6
5
4
3
2
1
VEGFR.KIT.PDGF
VEGFR
Topoisomerase
Sodium.Channel
Reverse.Transcripta
RAF
RAAS
Proteasome
PI3K
PDE
PARP
P450
mTOR
Microtubule.Associa
MEK
JAK
HSP
Histamine.Recept
HDAC
EGFR
DNA.RNA.Synthes
COX
CDK
Calcium.Channe
Aurora.Kinase
Adrenergic.Receptor.an
Adrenergic.Receptor.a
AChR.antagonis
5HTR.antagonis
5HTR.agonist
Fusion_kmeans
CP_kmeans
Pathway

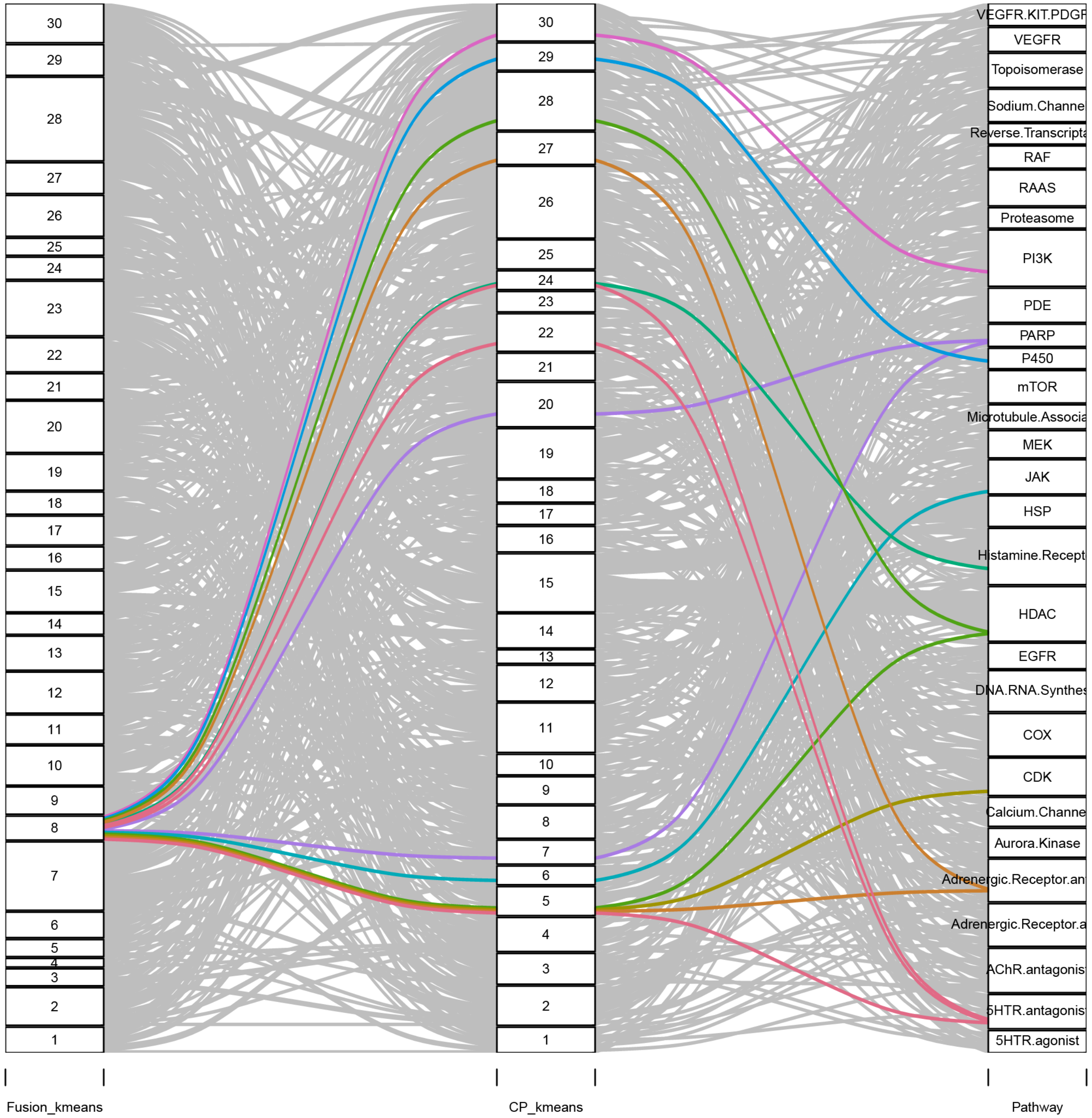
30
29
28
27
26
25
24
23
22
21
20
19
18
17
16
15
14
13
12
11
10
9
8
7
6
5
4
3
2
1
VEGFR.KIT.PDGF
VEGFR
Topoisomerase
Sodium.Channel
Reverse.Transcripta
RAF
RAAS
Proteasome
PI3K
PDE
PARP
P450
mTOR
Microtubule.Associa
MEK
JAK
HSP
Histamine.Recept
HDAC
EGFR
DNA.RNA.Synthes
COX
CDK
Calcium.Channe
Aurora.Kinase
Adrenergic.Receptor.an
Adrenergic.Receptor.a
AChR.antagonis
5HTR.antagonis
5HTR.agonist
Fusion_kmeans
CP_kmeans
Pathway

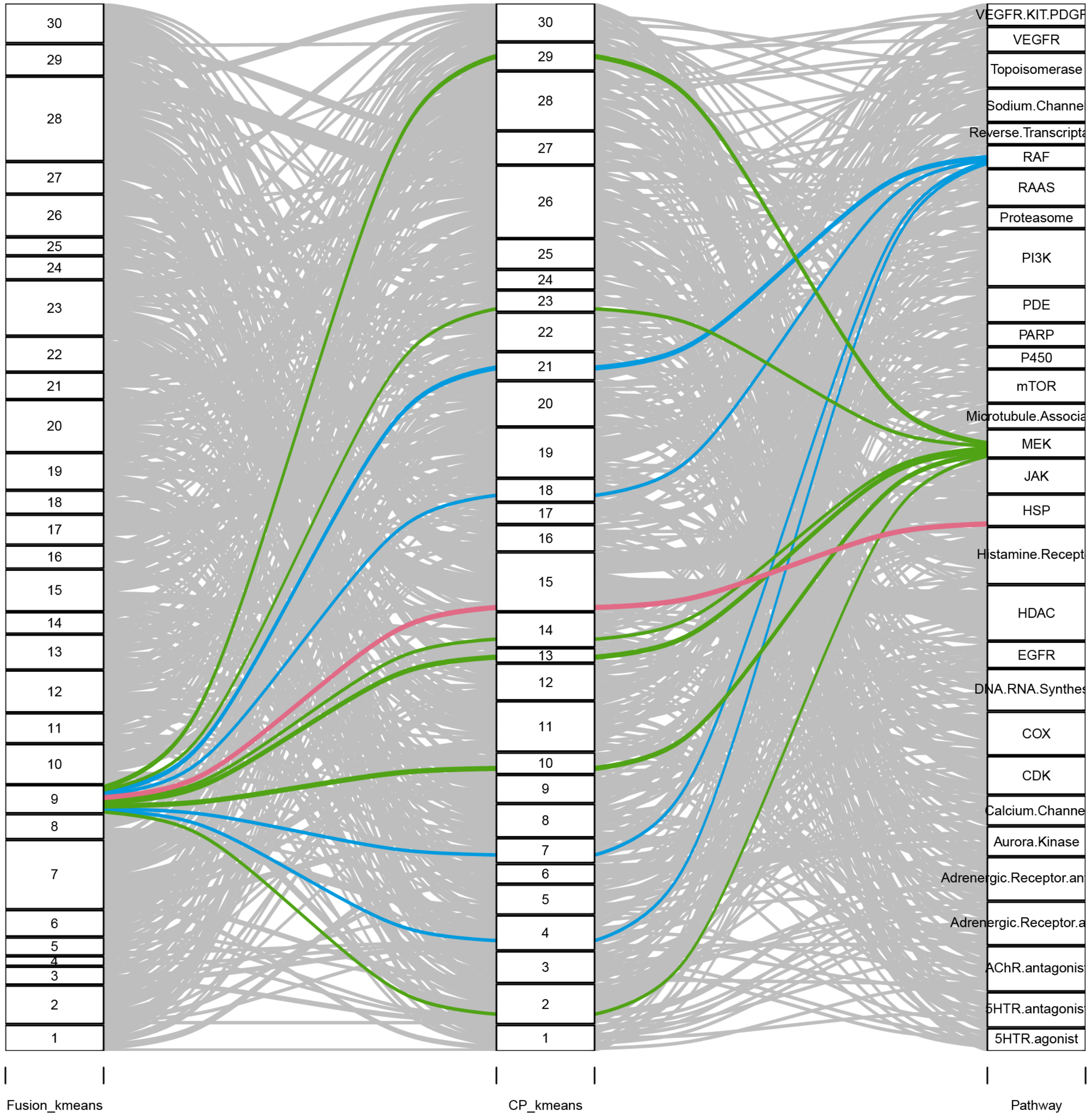

30
29
28
27
26
25
24
23
22
21
20
19
18
17
16
15
14
13
12
11
10
9
8
7
6
5
4
3
2
1
VEGFR.KIT.PDGF
VEGFR
Topoisomerase
Sodium.Channel
Reverse.Transcripta
RAF
RAAS
Proteasome
PI3K
PDE
PARP
P450
mTOR
Microtubule.Associa
MEK
JAK
HSP
Histamine.Recept
HDAC
EGFR
DNA.RNA.Synthes
COX
CDK
Calcium.Channe
Aurora.Kinase
Adrenergic.Receptor.an
Adrenergic.Receptor.a
AChR.antagonis
5HTR.antagonis
5HTR.agonist
Fusion_kmeans
CP_kmeans
Pathway

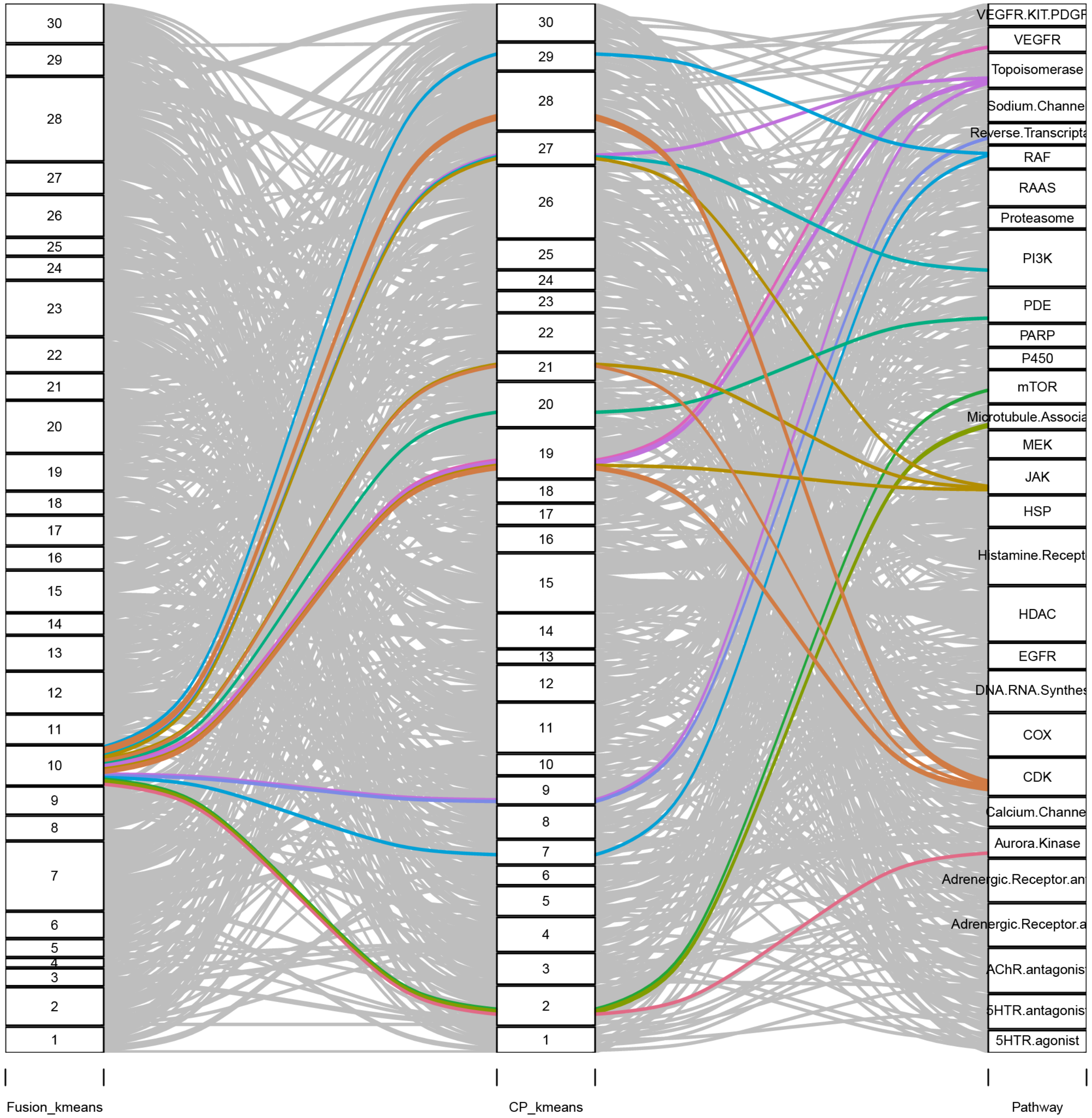
30
29
28
27
26
25
24
23
22
21
20
19
18
17
16
15
14
13
12
11
10
9
8
7
6
5
4
3
2
1
VEGFR.KIT.PDGF
VEGFR
Topoisomerase
Sodium.Channel
Reverse.Transcripta
RAF
RAAS
Proteasome
PI3K
PDE
PARP
P450
mTOR
Microtubule.Associa
MEK
JAK
HSP
Histamine.Recept
HDAC
EGFR
DNA.RNA.Synthes
COX
CDK
Calcium.Channe
Aurora.Kinase
Adrenergic.Receptor.an
Adrenergic.Receptor.a
AChR.antagonis
5HTR.antagonis
5HTR.agonist
Fusion_kmeans
CP_kmeans
Pathway

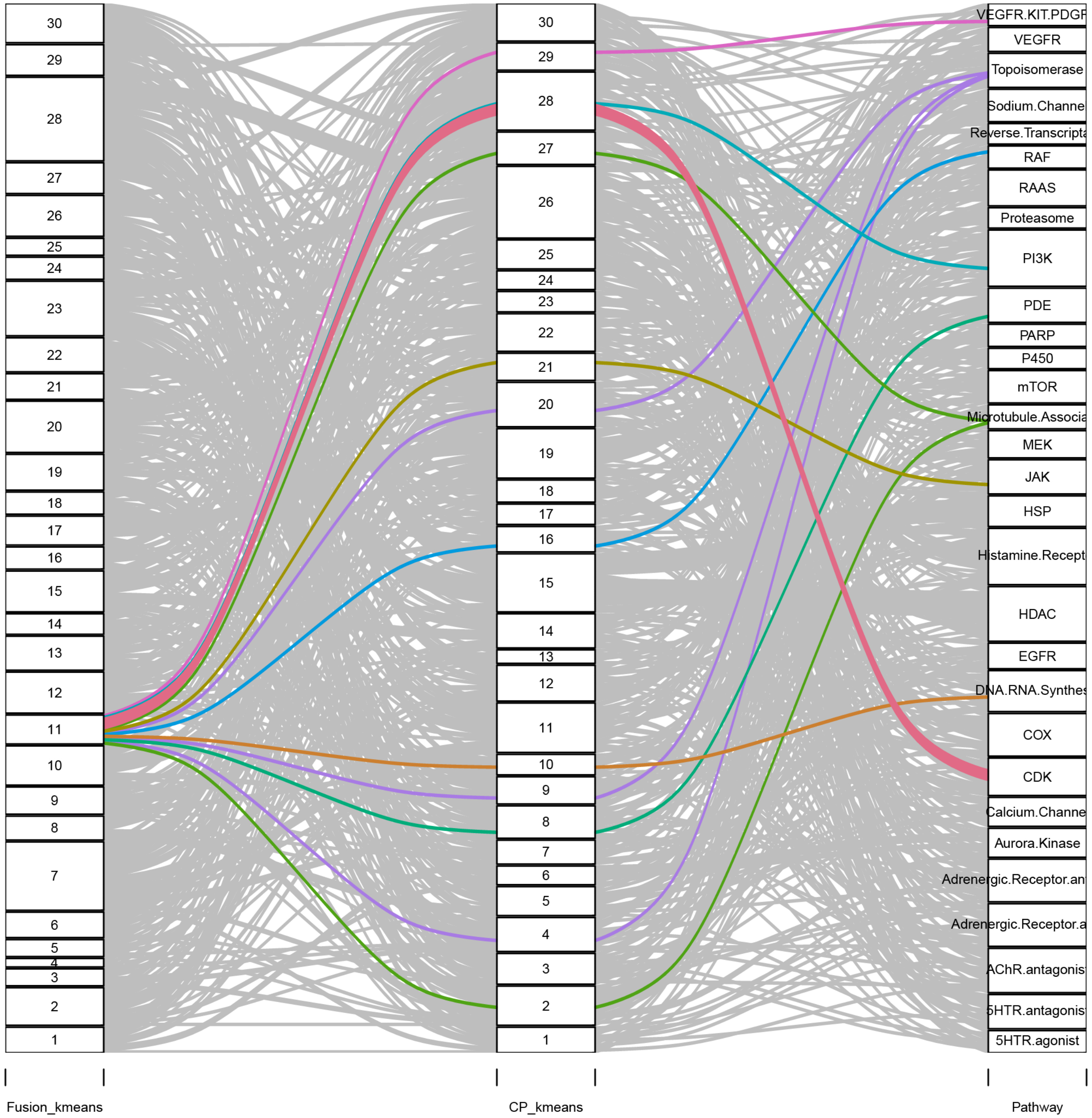
30
29
28
27
26
25
24
23
22
21
20
19
18
17
16
15
14
13
12
11
10
9
8
7
6
5
4
3
2
1
VEGFR.KIT.PDGF
VEGFR
Topoisomerase
Sodium.Channel
Reverse.Transcripta
RAF
RAAS
Proteasome
PI3K
PDE
PARP
P450
mTOR
Microtubule.Associa
MEK
JAK
HSP
Histamine.Recept
HDAC
EGFR
DNA.RNA.Synthes
COX
CDK
Calcium.Channe
Aurora.Kinase
Adrenergic.Receptor.an
Adrenergic.Receptor.a
AChR.antagonis
5HTR.antagonis
5HTR.agonist
Fusion_kmeans
CP_kmeans
Pathway

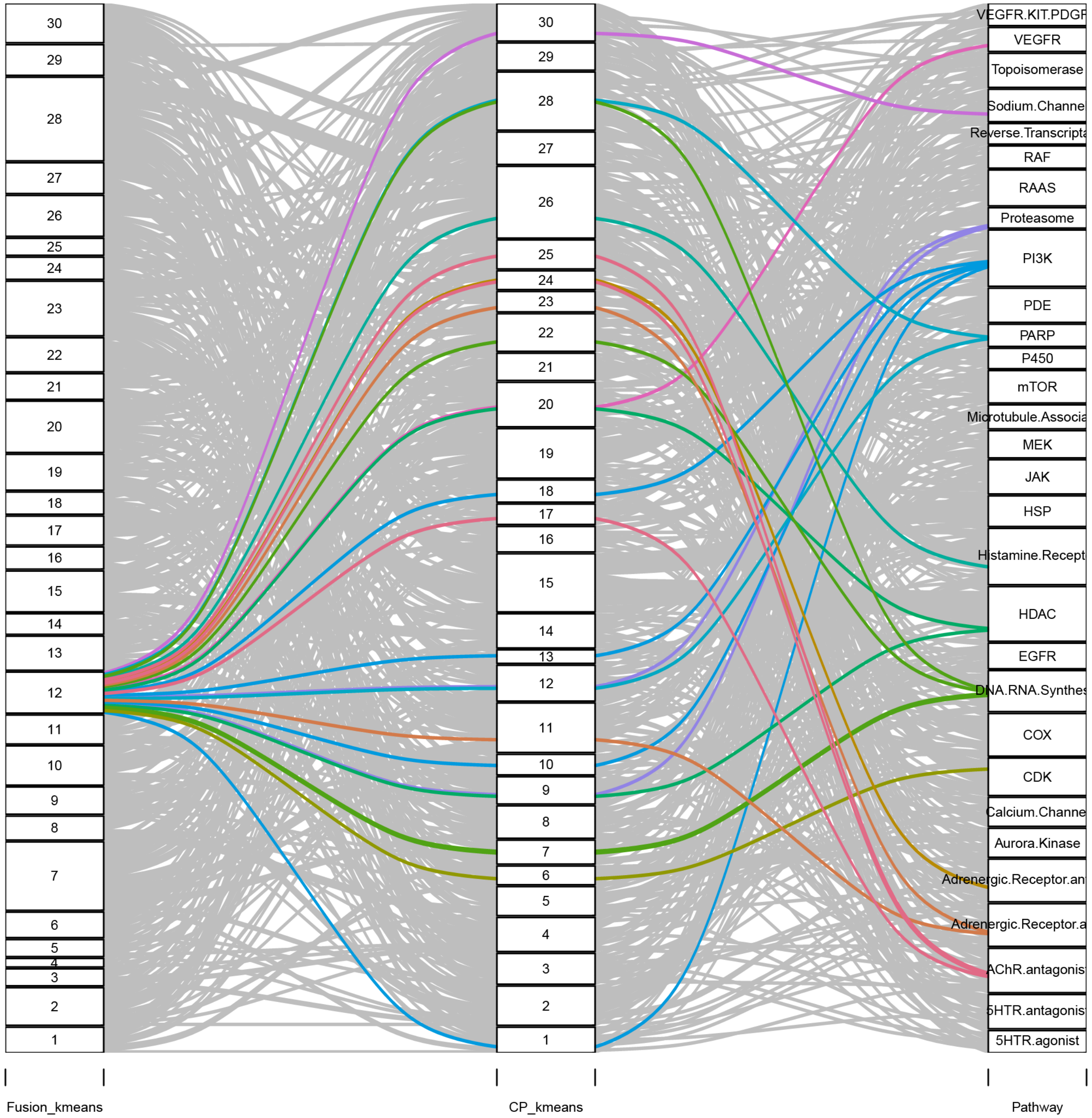

Fusion_kmeans
CP_kmeans
Pathway
30
29
28
27
26
25
24
23
22
21
20
19
18
17
16
15
14
13
12
11
10
9
8
7
6
5
4
3
2
1
VEGFR.KIT.PDGF
VEGFR
Topoisomerase
Sodium.Channel
Reverse.Transcripta
RAF
RAAS
Proteasome
PI3K
PDE
PARP
P450
mTOR
Microtubule.Associa
MEK
JAK
HSP
Histamine.Recept
HDAC
EGFR
DNA.RNA.Synthes
COX
CDK
Calcium.Channe
Aurora.Kinase
Adrenergic.Receptor.an
Adrenergic.Receptor.a
AChR.antagonis
5HTR.antagonis
5HTR.agonist

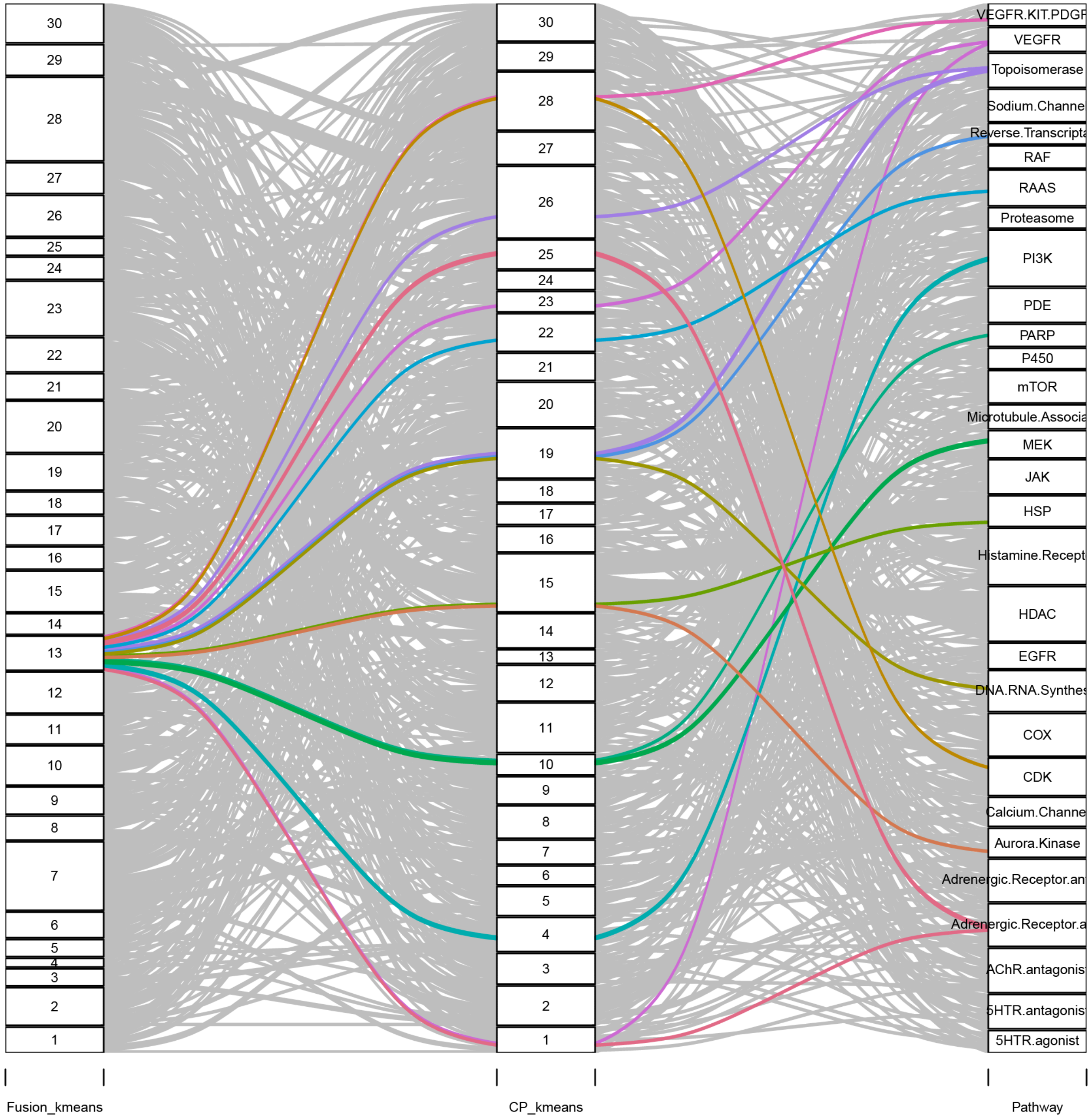

30
29
28
27
26
25
24
23
22
21
20
19
18
17
16
15
14
13
12
11
10
9
8
7
6
5
4
3
2
1
VEGFR.KIT.PDGF
VEGFR
Topoisomerase
Sodium.Channel
Reverse.Transcripta
RAF
RAAS
Proteasome
PI3K
PDE
PARP
P450
mTOR
Microtubule.Associa
MEK
JAK
HSP
Histamine.Recept
HDAC
EGFR
DNA.RNA.Synthes
COX
CDK
Calcium.Channe
Aurora.Kinase
Adrenergic.Receptor.an
Adrenergic.Receptor.a
AChR.antagonis
5HTR.antagonis
5HTR.agonist
Fusion_kmeans
CP_kmeans
Pathway

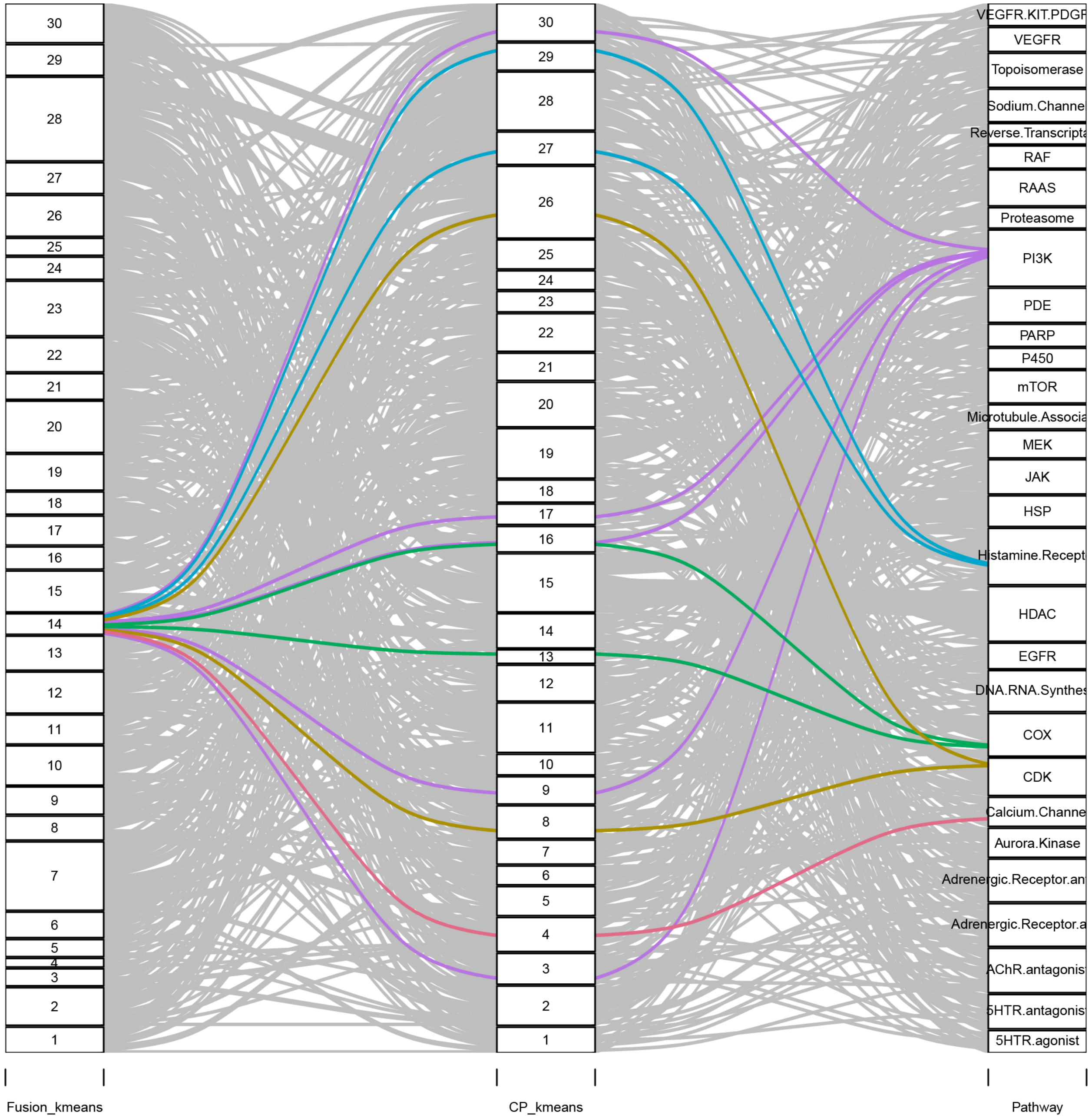
30
29
28
27
26
25
24
23
22
21
20
19
18
17
16
15
14
13
12
11
10
9
8
7
6
5
4
3
2
1
VEGFR.KIT.PDGF
VEGFR
Topoisomerase
Sodium.Channel
Reverse.Transcripta
RAF
RAAS
Proteasome
PI3K
PDE
PARP
P450
mTOR
Microtubule.Associa
MEK
JAK
HSP
Histamine.Recept
HDAC
EGFR
DNA.RNA.Synthes
COX
CDK
Calcium.Channe
Aurora.Kinase
Adrenergic.Receptor.an
Adrenergic.Receptor.a
AChR.antagonis
5HTR.antagonis
5HTR.agonist
Fusion_kmeans
CP_kmeans
Pathway

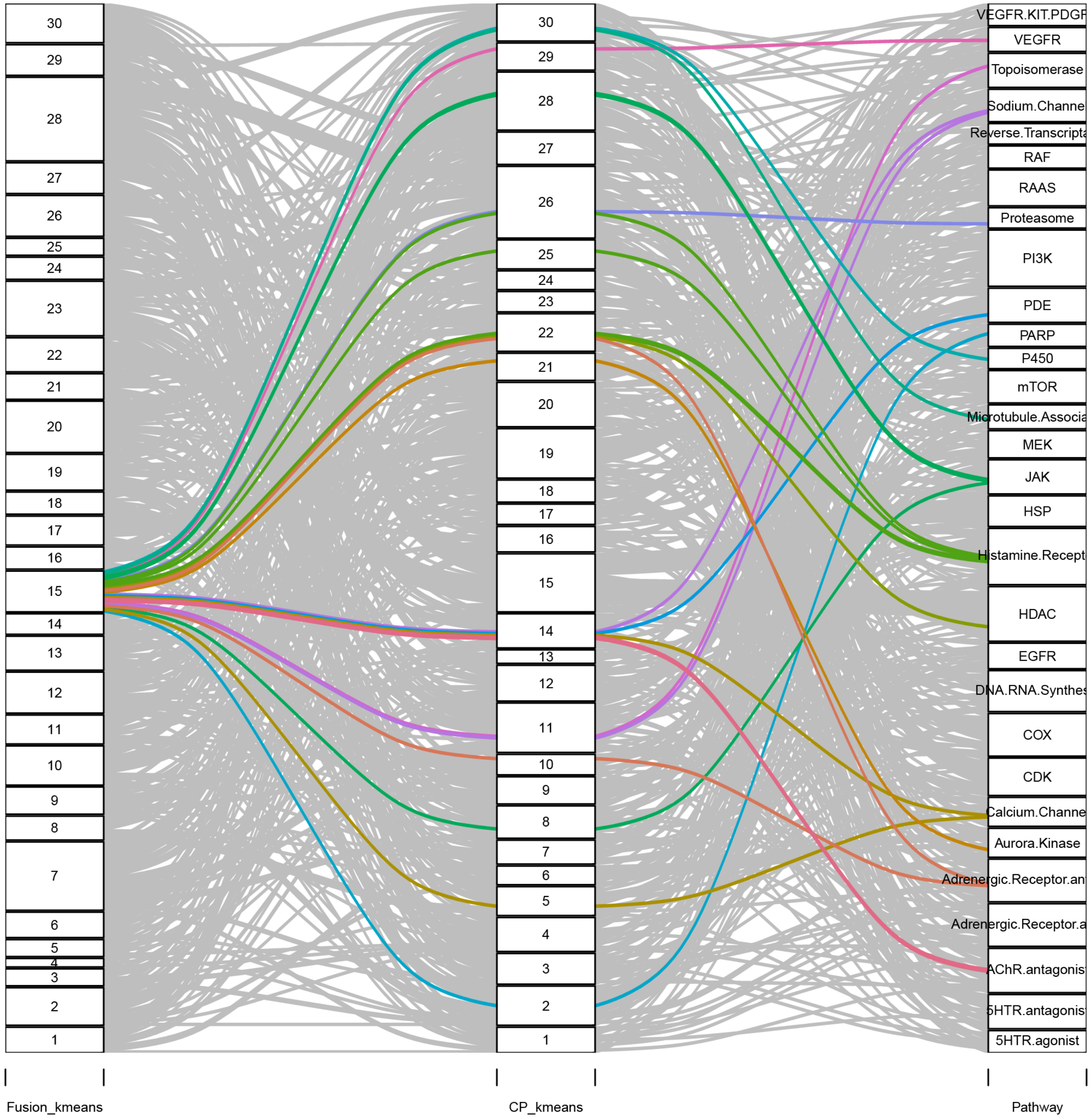
30
29
28
27
26
25
24
23
22
21
20
19
18
17
16
15
14
13
12
11
10
9
8
7
6
5
4
3
2
1
VEGFR.KIT.PDGF
VEGFR
Topoisomerase
Sodium.Channel
Reverse.Transcripta
RAF
RAAS
Proteasome
PI3K
PDE
PARP
P450
mTOR
Microtubule.Associa
MEK
JAK
HSP
Histamine.Recept
HDAC
EGFR
DNA.RNA.Synthes
COX
CDK
Calcium.Channe
Aurora.Kinase
Adrenergic.Receptor.an
Adrenergic.Receptor.a
AChR.antagonis
5HTR.antagonis
5HTR.agonist
Fusion_kmeans
CP_kmeans
Pathway

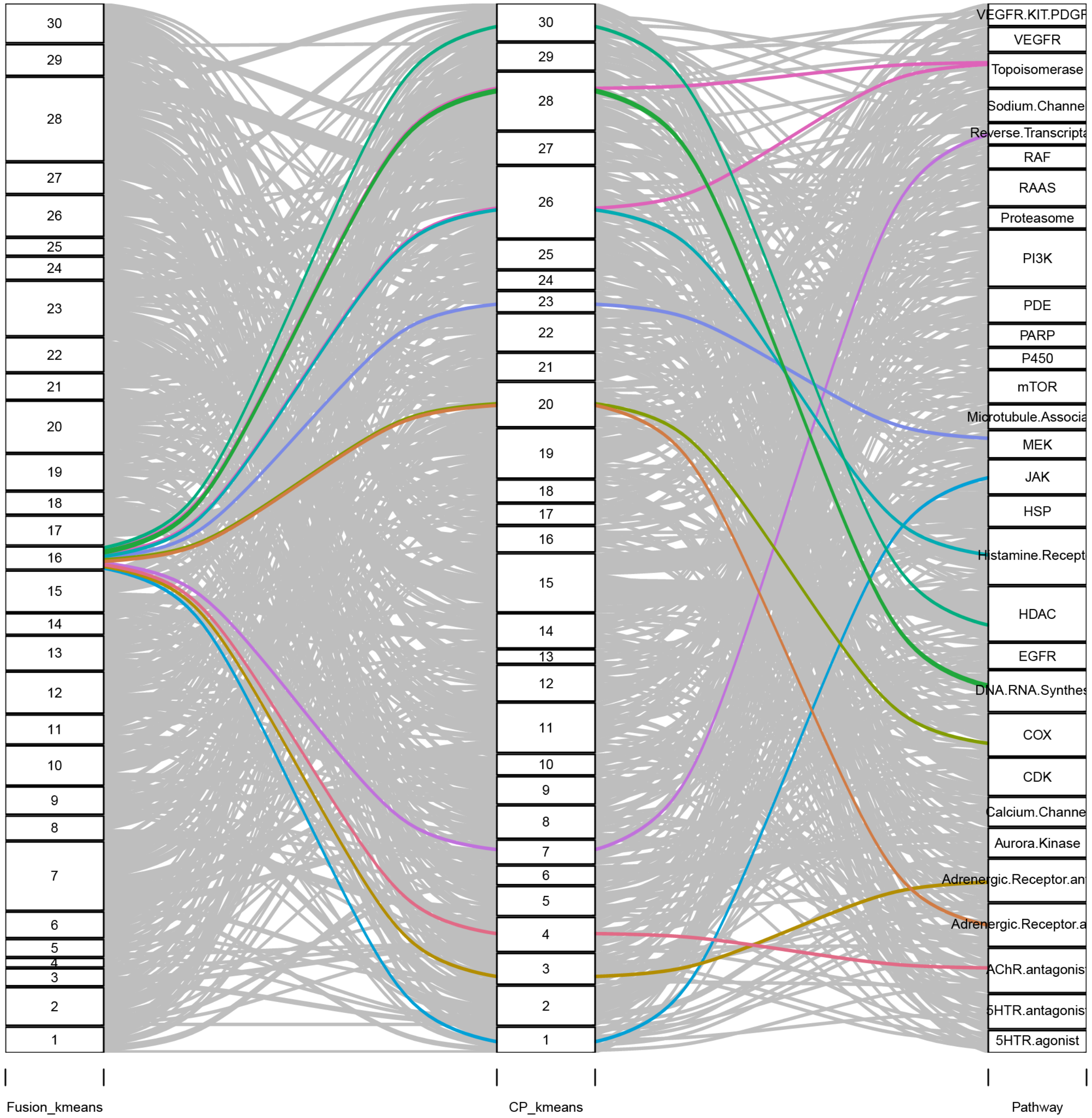
Fusion_kmeans
CP_kmeans
Pathway
VEGFR.KIT.PDGF
VEGFR
Topoisomerase
Sodium.Channel
Reverse.Transcripta
RAF
RAAS
Proteasome
PI3K
PDE
PARP
P450
mTOR
Microtubule.Associa
MEK
JAK
HSP
Histamine.Recept
HDAC
EGFR
DNA.RNA.Synthes
COX
CDK
Calcium.Channe
Aurora.Kinase
Adrenergic.Receptor.an
Adrenergic.Receptor.a
AChR.antagonis
5HTR.antagonis
5HTR.agonist

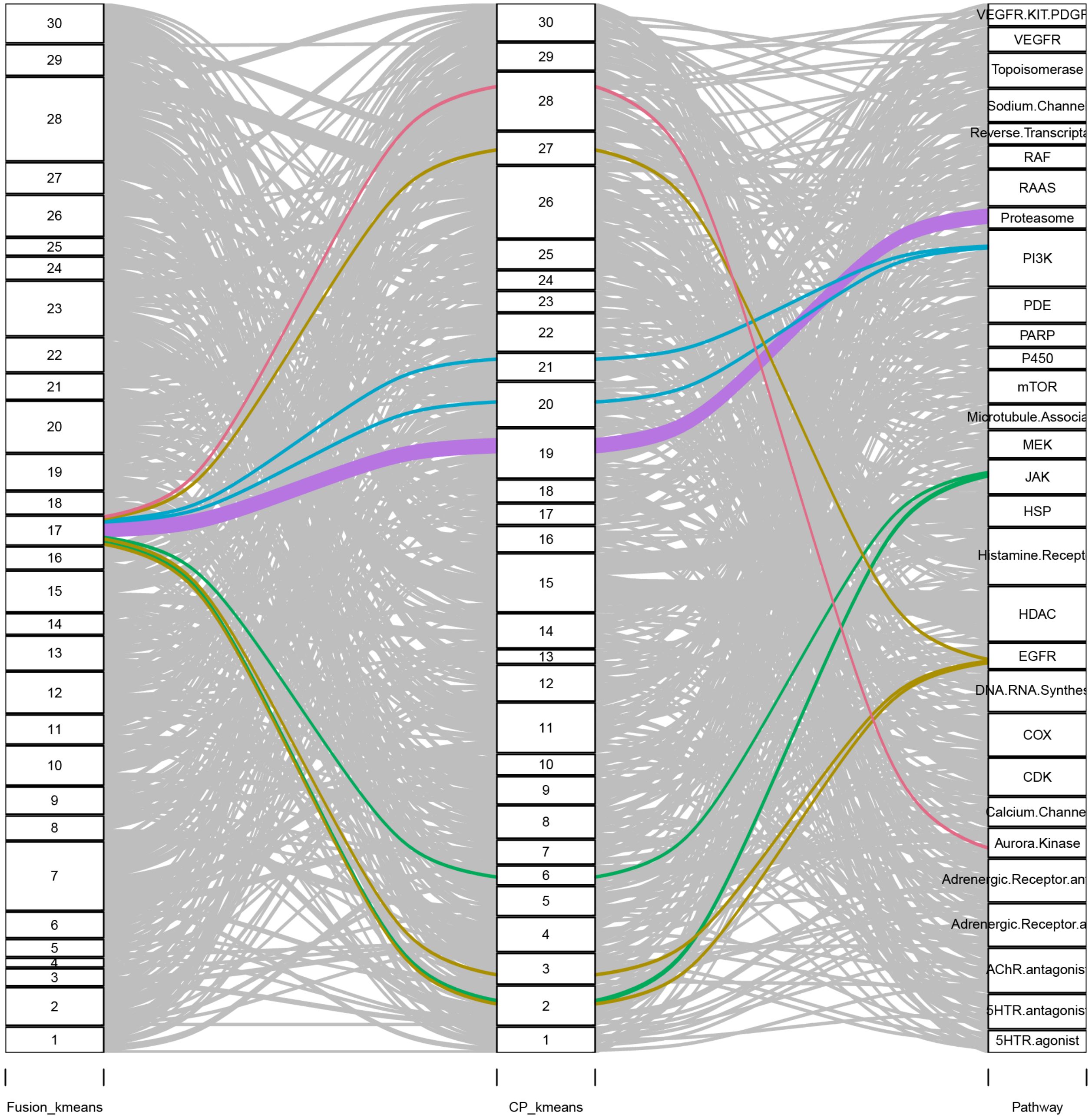
30
29
28
27
26
25
24
23
22
21
20
19
18
17
16
15
14
13
12
11
10
9
8
7
6
5
4
3
2
1
VEGFR.KIT.PDGF
VEGFR
Topoisomerase
Sodium.Channel
Reverse.Transcripta
RAF
RAAS
Proteasome
PI3K
PDE
PARP
P450
mTOR
Microtubule.Associa
MEK
JAK
HSP
Histamine.Recept
HDAC
EGFR
DNA.RNA.Synthes
COX
CDK
Calcium.Channe
Aurora.Kinase
Adrenergic.Receptor.an
Adrenergic.Receptor.a
AChR.antagonis
5HTR.antagonis
5HTR.agonist
Fusion_kmeans
CP_kmeans
Pathway

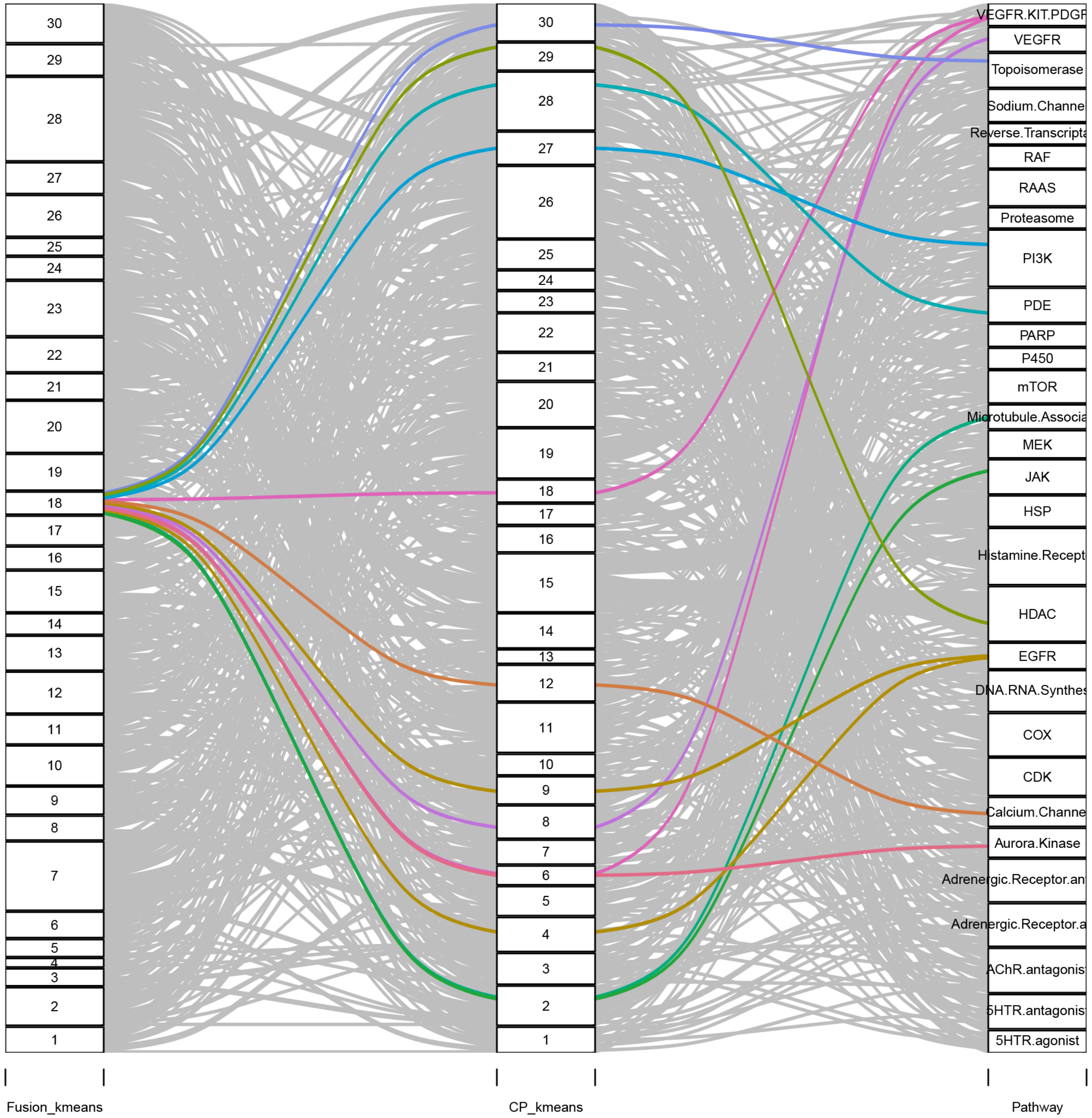
Fusion_kmeans
CP_kmeans
Pathway
VEGFR.KIT.PDGF
VEGFR
Topoisomerase
Sodium.Channel
Reverse.Transcripta
RAF
RAAS
Proteasome
PI3K
PDE
PARP
P450
mTOR
Microtubule.Associa
MEK
JAK
HSP
Histamine.Recept
HDAC
EGFR
DNA.RNA.Synthes
COX
CDK
Calcium.Channe
Aurora.Kinase
Adrenergic.Receptor.an
Adrenergic.Receptor.a
AChR.antagonis
5HTR.antagonis
5HTR.agonist

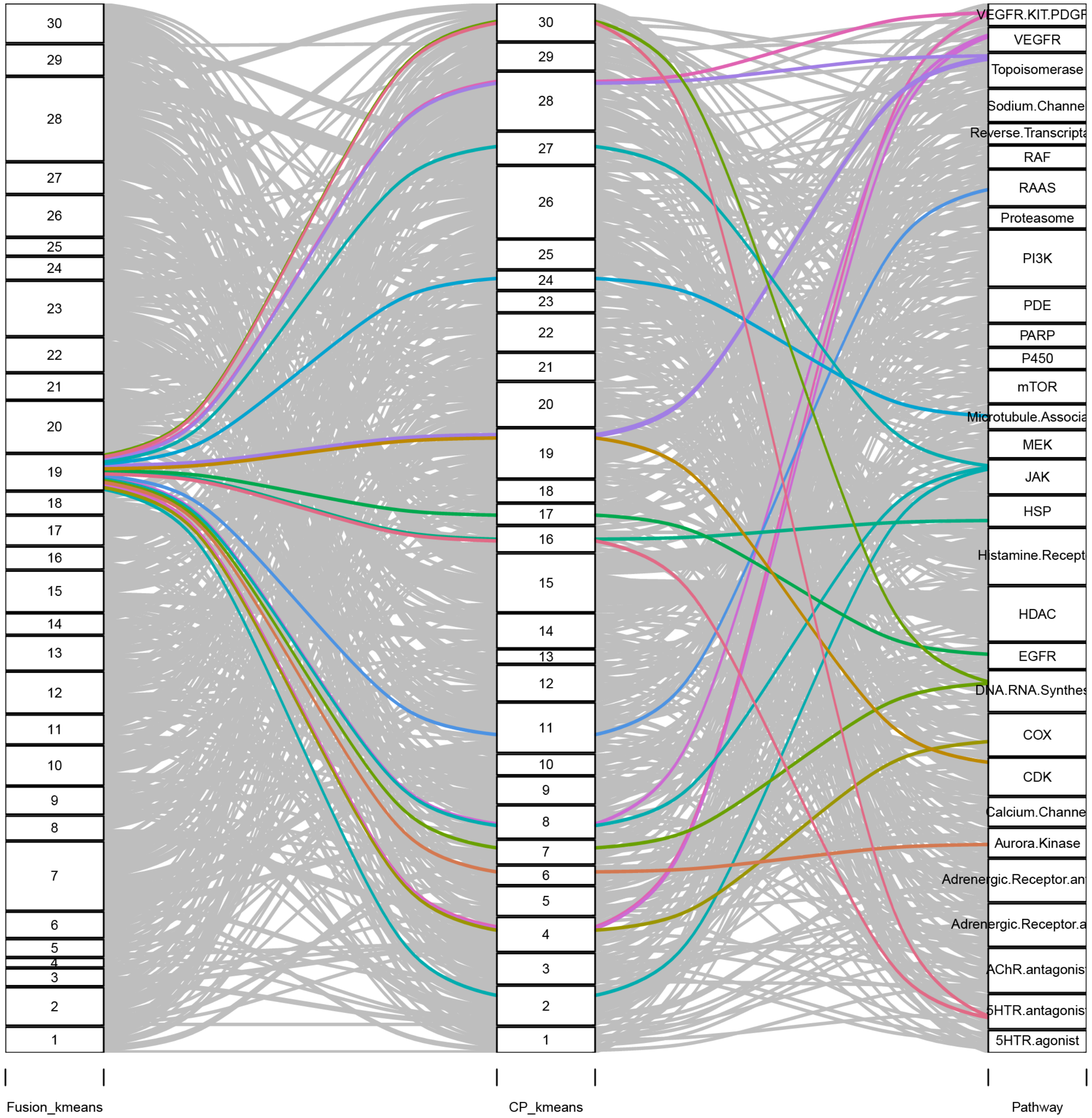

Fusion_kmeans
CP_kmeans
Pathway
VEGFR.KIT.PDGF
VEGFR
Topoisomerase
Sodium.Channel
Reverse.Transcripta
RAF
RAAS
Proteasome
PI3K
PDE
PARP
P450
mTOR
Microtubule.Associa
MEK
JAK
HSP
Histamine.Recept
HDAC
EGFR
DNA.RNA.Synthes
COX
CDK
Calcium.Channe
Aurora.Kinase
Adrenergic.Receptor.an
Adrenergic.Receptor.a
AChR.antagonist
5HTR.antagonist
5HTR.agonist

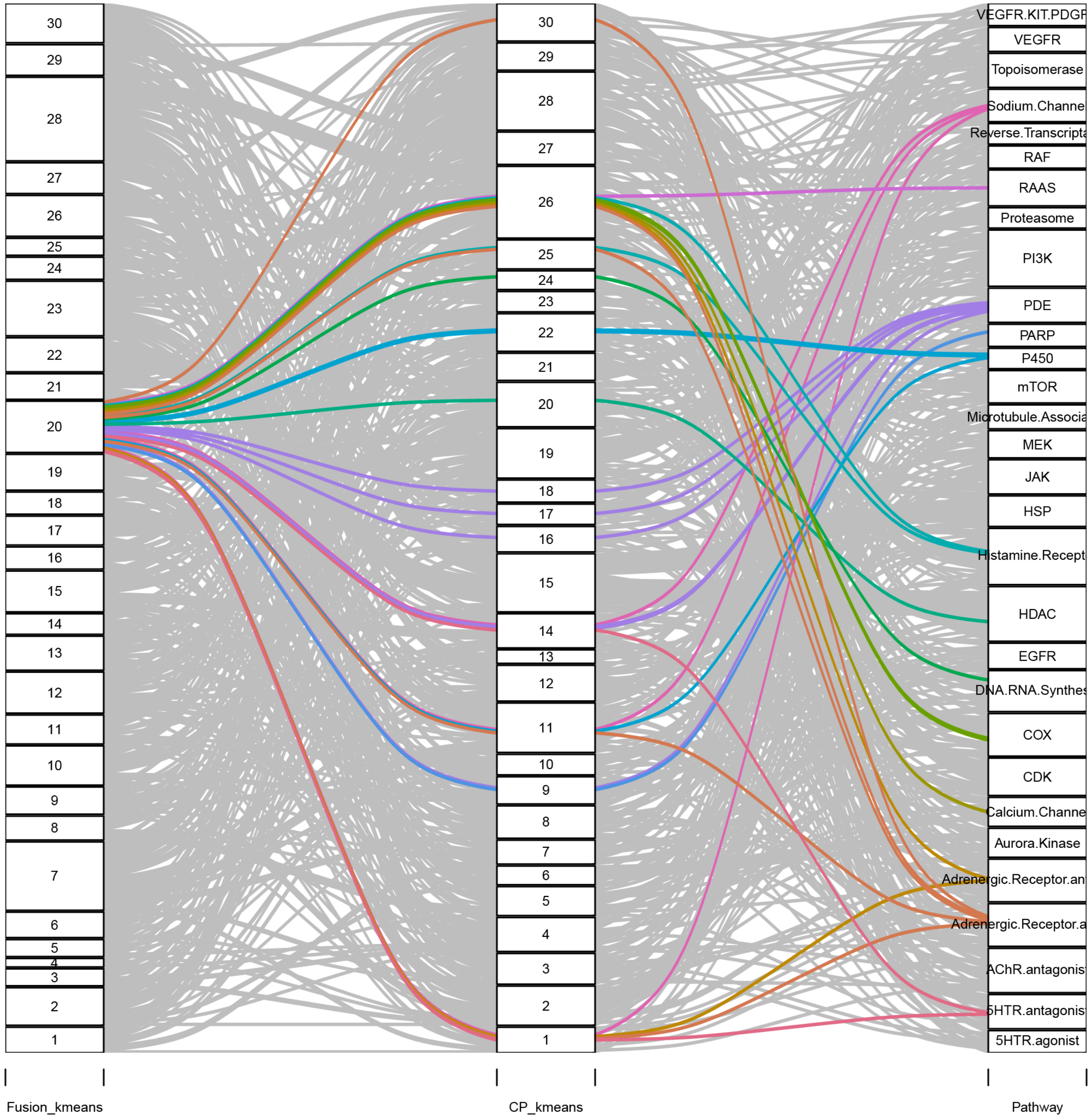

30
29
28
27
26
25
24
23
22
21
20
19
18
17
16
15
14
13
12
11
10
9
8
7
6
5
4
3
2
1
VEGFR.KIT.PDGF
VEGFR
Topoisomerase
Sodium.Channel
Reverse.Transcripta
RAF
RAAS
Proteasome
PI3K
PDE
PARP
P450
mTOR
Microtubule.Associa
MEK
JAK
HSP
Histamine.Recept
HDAC
EGFR
DNA.RNA.Synthes
COX
CDK
Calcium.Channe
Aurora.Kinase
Adrenergic.Receptor.an
Adrenergic.Receptor.a
AChR.antagonis
5HTR.antagonis
5HTR.agonist
Fusion_kmeans
CP_kmeans
Pathway

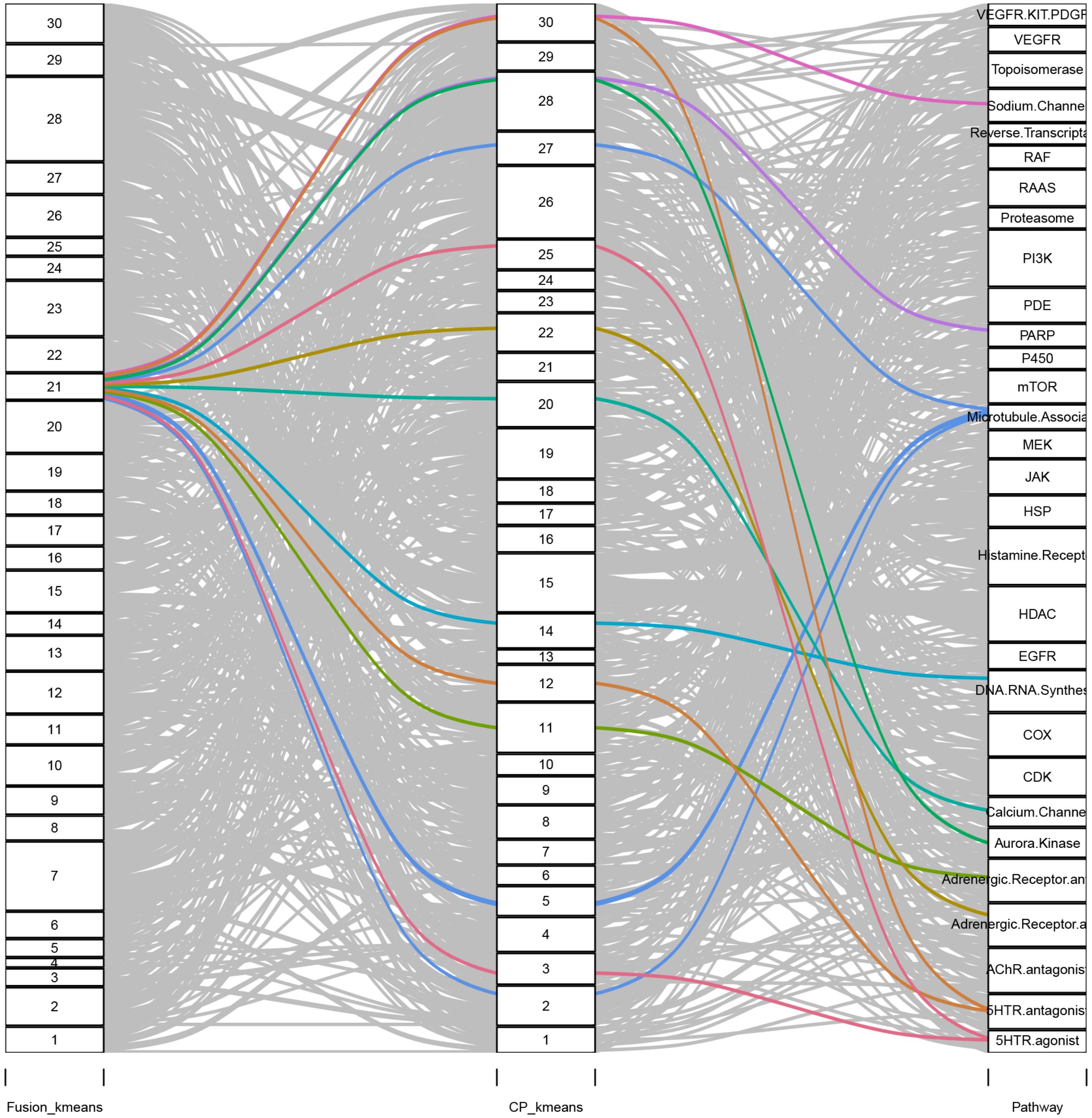
30
29
28
27
26
25
24
23
22
21
20
19
18
17
16
15
14
13
12
11
10
9
8
7
6
5
4
3
2
1
VEGFR.KIT.PDGF
VEGFR
Topoisomerase
Sodium.Channel
Reverse.Transcripta
RAF
RAAS
Proteasome
PI3K
PDE
PARP
P450
mTOR
Microtubule.Associa
MEK
JAK
HSP
Histamine.Recept
HDAC
EGFR
DNA.RNA.Synthes
COX
CDK
Calcium.Channe
Aurora.Kinase
Adrenergic.Receptor.an
Adrenergic.Receptor.a
AChR.antagonis
5HTR.antagonis
5HTR.agonist
Fusion_kmeans
CP_kmeans
Pathway

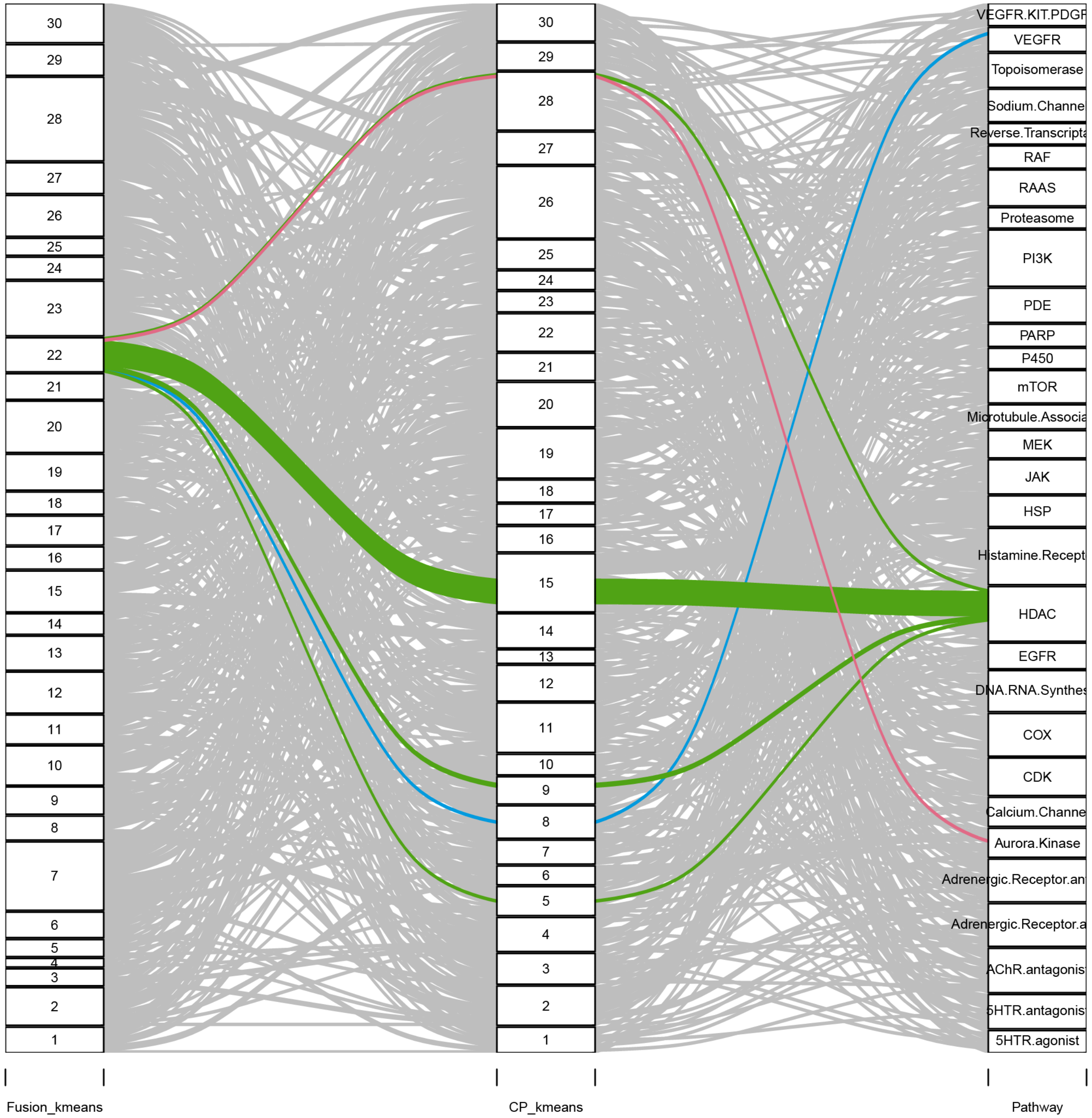
30
29
28
27
26
25
24
23
22
21
20
19
18
17
16
15
14
13
12
11
10
9
8
7
6
5
4
3
2
1
VEGFR.KIT.PDGF
VEGFR
Topoisomerase
Sodium.Channe
Reverse.Transcripta
RAF
RAAS
Proteasome
PI3K
PDE
PARP
P450
mTOR
Microtubule.Associa
MEK
JAK
HSP
Histamine.Recept
HDAC
EGFR
DNA.RNA.Synthes
COX
CDK
Calcium.Channe
Aurora.Kinase
Adrenergic.Receptor.an
Adrenergic.Receptor.a
AChR.antagonis
5HTR.antagonis
5HTR.agonist
Fusion_kmeans
CP_kmeans
Pathway

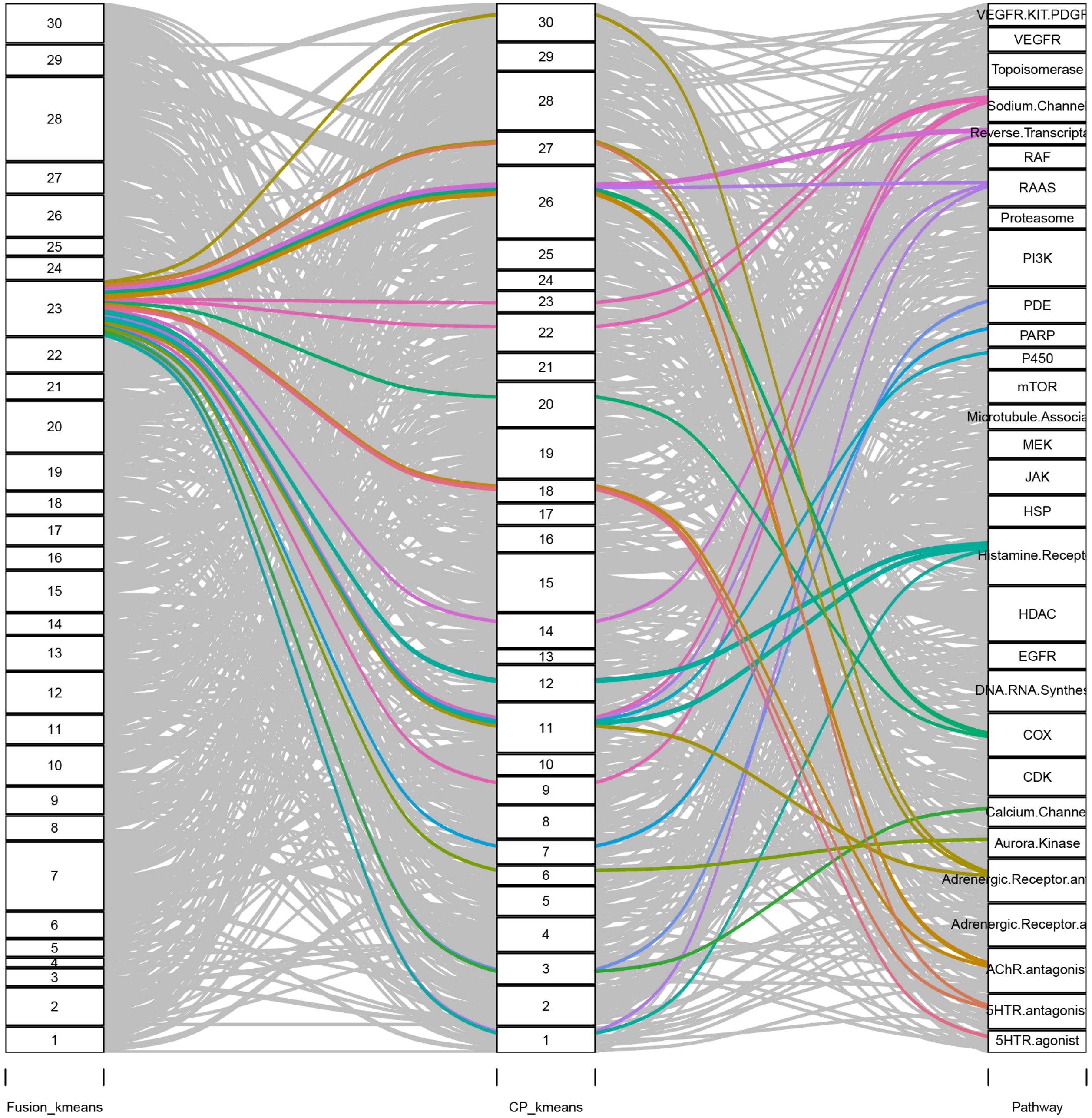

30
29
28
27
26
25
24
23
22
21
20
19
18
17
16
15
14
13
12
11
10
9
8
7
6
5
4
3
2
1
VEGFR.KIT.PDGF
VEGFR
Topoisomerase
Sodium.Channel
Reverse.Transcripta
RAF
RAAS
Proteasome
PI3K
PDE
PARP
P450
mTOR
Microtubule.Associa
MEK
JAK
HSP
Histamine.Recept
HDAC
EGFR
DNA.RNA.Synthes
COX
CDK
Calcium.Channe
Aurora.Kinase
Adrenergic.Receptor.an
Adrenergic.Receptor.a
AChR.antagonis
5HTR.antagonis
5HTR.agonist
Fusion_kmeans
CP_kmeans
Pathway

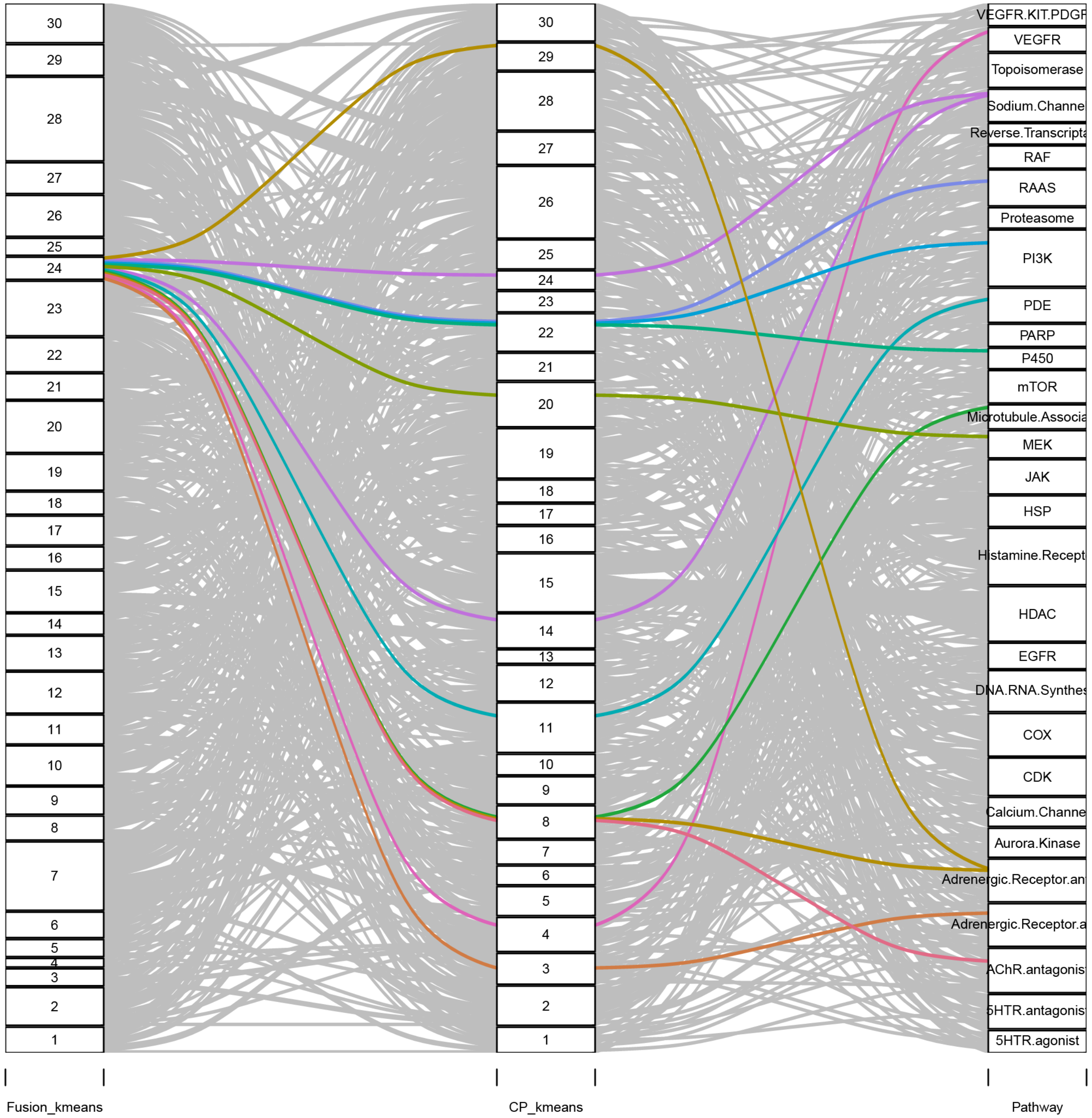

Fusion_kmeans
CP_kmeans
Pathway
VEGFR.KIT.PDGF
VEGFR
Topoisomerase
Sodium.Channel
Reverse.Transcripta
RAF
RAAS
Proteasome
PI3K
PDE
PARP
P450
mTOR
Microtubule.Associa
MEK
JAK
HSP
Histamine.Recept
HDAC
EGFR
DNA.RNA.Synthes
COX
CDK
Calcium.Channe
Aurora.Kinase
Adrenergic.Receptor.an
Adrenergic.Receptor.a
AChR.antagonis
5HTR.antagonis
5HTR.agonist

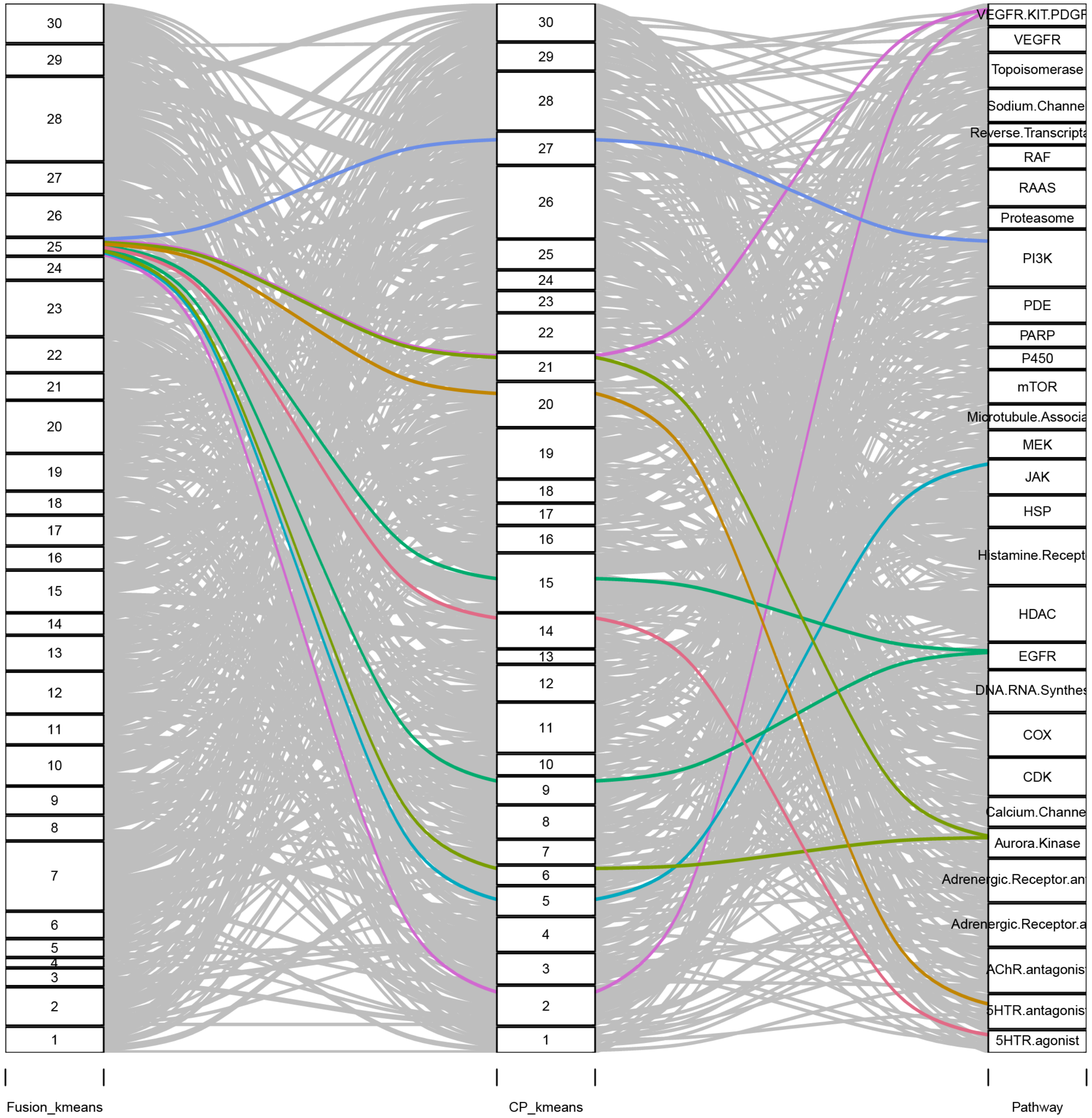
30
29
28
27
26
25
24
23
22
21
20
19
18
17
16
15
14
13
12
11
10
9
8
7
6
5
4
3
2
1
VEGFR.KIT.PDGF
VEGFR
Topoisomerase
Sodium.Channel
Reverse.Transcripta
RAF
RAAS
Proteasome
PI3K
PDE
PARP
P450
mTOR
Microtubule.Associa
MEK
JAK
HSP
Histamine.Recept
HDAC
EGFR
DNA.RNA.Synthes
COX
CDK
Calcium.Channe
Aurora.Kinase
Adrenergic.Receptor.an
Adrenergic.Receptor.a
AChR.antagonist
5HTR.antagonist
5HTR.agonist
Fusion_kmeans
CP_kmeans
Pathway

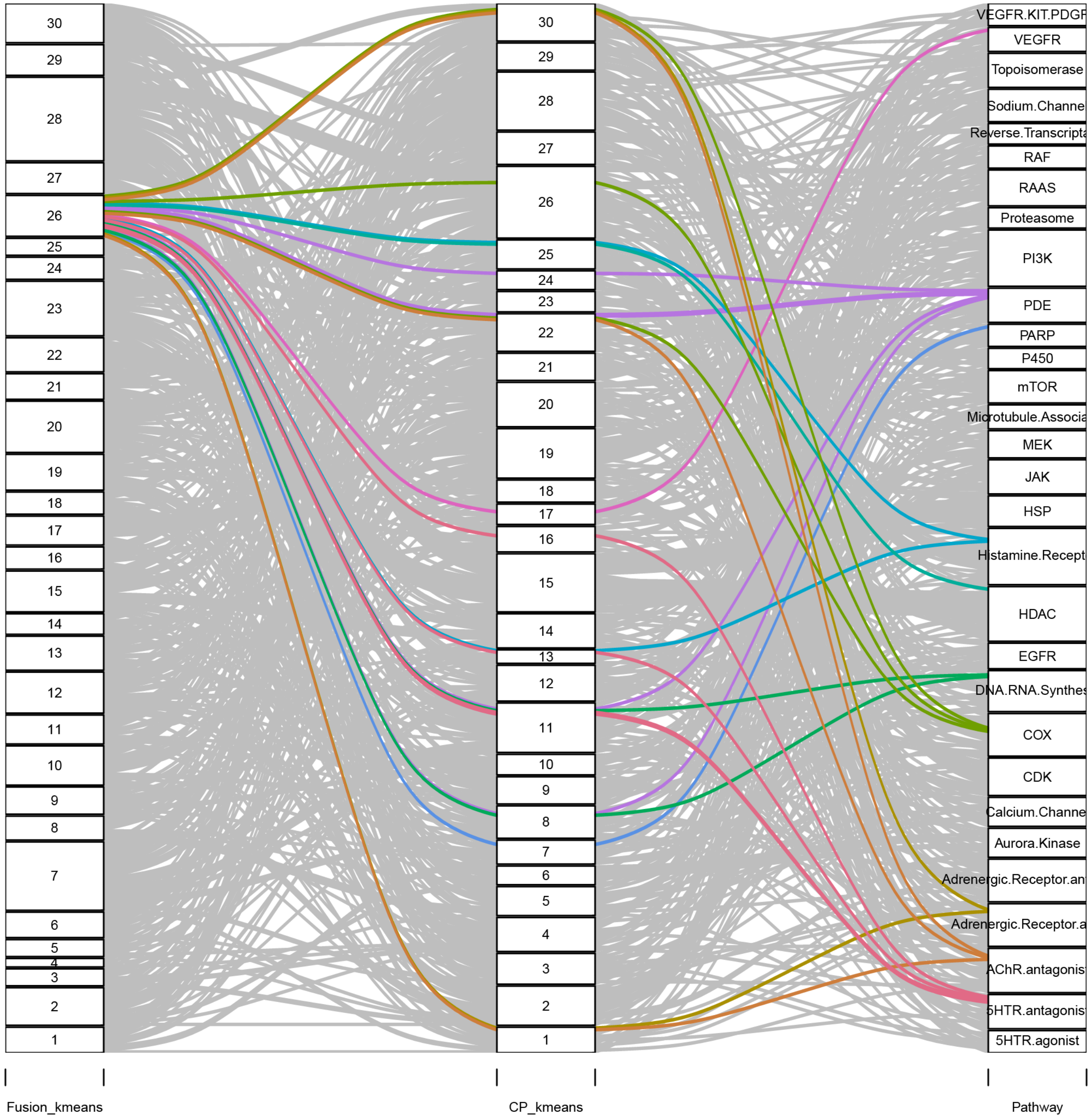
30
29
28
27
26
25
24
23
22
21
20
19
18
17
16
15
14
13
12
11
10
9
8
7
6
5
4
3
2
1
VEGFR.KIT.PDGF
VEGFR
Topoisomerase
Sodium.Channel
Reverse.Transcripta
RAF
RAAS
Proteasome
PI3K
PDE
PARP
P450
mTOR
Microtubule.Associa
MEK
JAK
HSP
Histamine.Recept
HDAC
EGFR
DNA.RNA.Synthes
COX
CDK
Calcium.Channe
Aurora.Kinase
Adrenergic.Receptor.an
Adrenergic.Receptor.a
AChR.antagonis
5HTR.antagonis
5HTR.agonist
Fusion_kmeans
CP_kmeans
Pathway

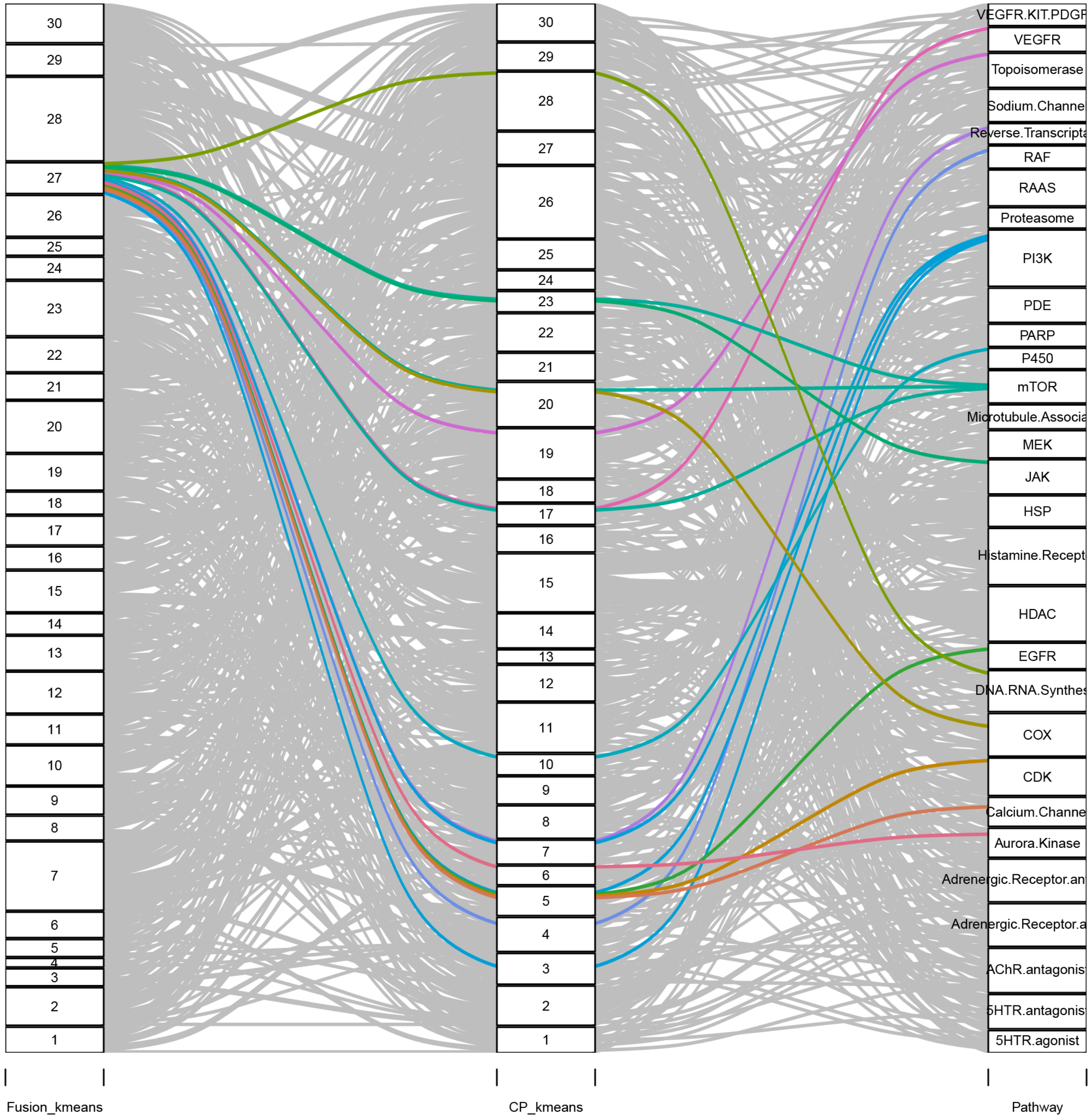
30
29
28
27
26
25
24
23
22
21
20
19
18
17
16
15
14
13
12
11
10
9
8
7
6
5
4
3
2
1
VEGFR.KIT.PDGF
VEGFR
Topoisomerase
Sodium.Channel
Reverse.Transcripta
RAF
RAAS
Proteasome
PI3K
PDE
PARP
P450
mTOR
Microtubule.Associa
MEK
JAK
HSP
Histamine.Recept
HDAC
EGFR
DNA.RNA.Synthes
COX
CDK
Calcium.Channe
Aurora.Kinase
Adrenergic.Receptor.an
Adrenergic.Receptor.a
AChR.antagonis
5HTR.antagonis
5HTR.agonist
Fusion_kmeans
CP_kmeans
Pathway

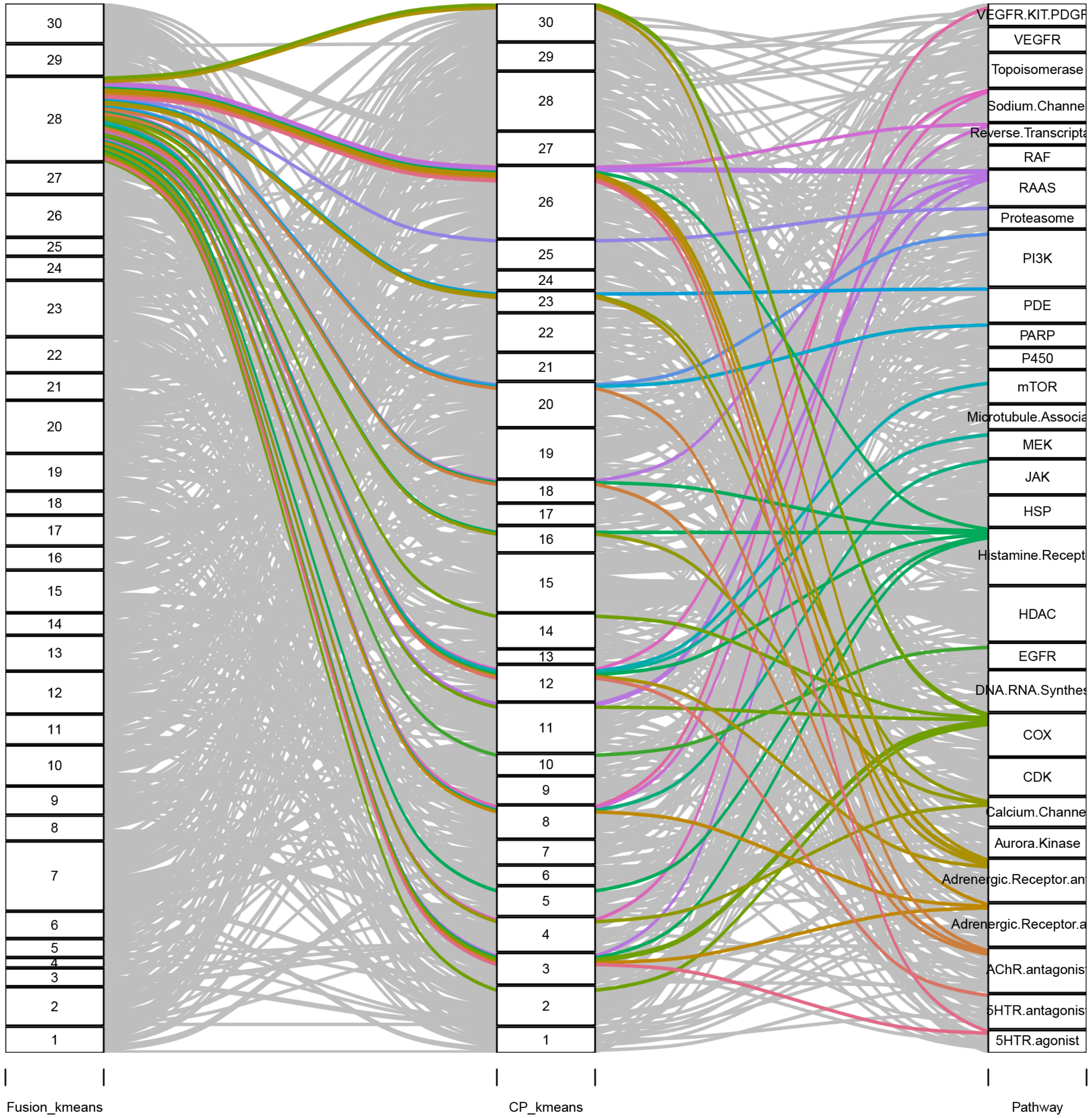
Fusion_kmeans
CP_kmeans
Pathway
30
29
28
27
26
25
24
23
22
21
20
19
18
17
16
15
14
13
12
11
10
9
8
7
6
5
4
3
2
1
VEGFR.KIT.PDGF
VEGFR
Topoisomerase
Sodium.Channel
Reverse.Transcripta
RAF
RAAS
Proteasome
PI3K
PDE
PARP
P450
mTOR
Microtubule.Associa
MEK
JAK
HSP
Histamine.Recept
HDAC
EGFR
DNA.RNA.Synthes
COX
CDK
Calcium.Channe
Aurora.Kinase
Adrenergic.Receptor.an
Adrenergic.Receptor.a
AChR.antagonis
5HTR.antagonis
5HTR.agonist

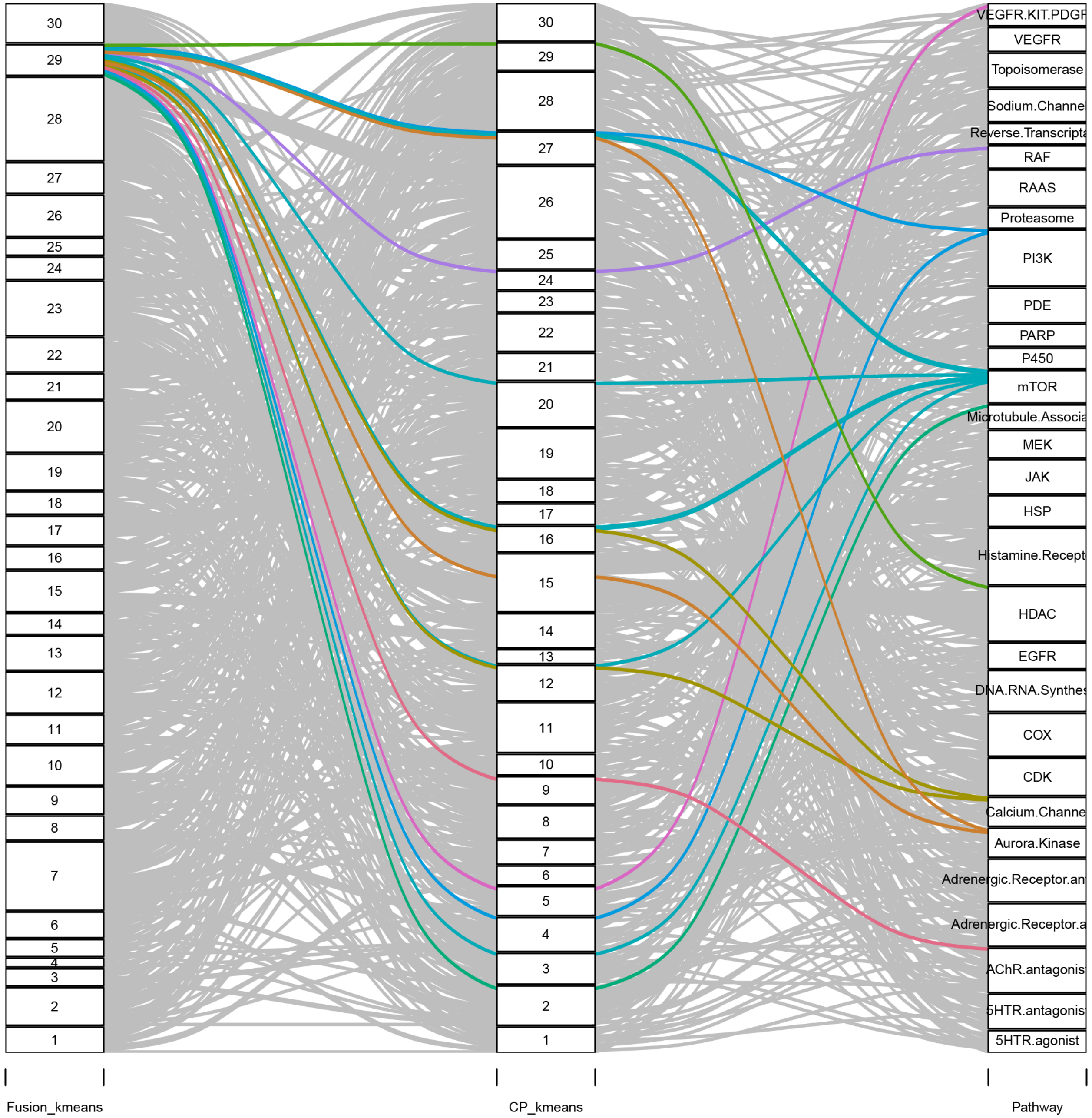
Fusion_kmeans
CP_kmeans
Pathway
VEGFR.KIT.PDGF
VEGFR
Topoisomerase
Sodium.Channe
Reverse.Transcripta
RAF
RAAS
Proteasome
PI3K
PDE
PARP
P450
mTOR
Microtubule.Associa
MEK
JAK
HSP
Histamine.Recept
HDAC
EGFR
DNA.RNA.Synthes
COX
CDK
Calcium.Channe
Aurora.Kinase
Adrenergic.Receptor.an
Adrenergic.Receptor.a
AChR.antagonis
5HTR.antagonis
5HTR.agonist

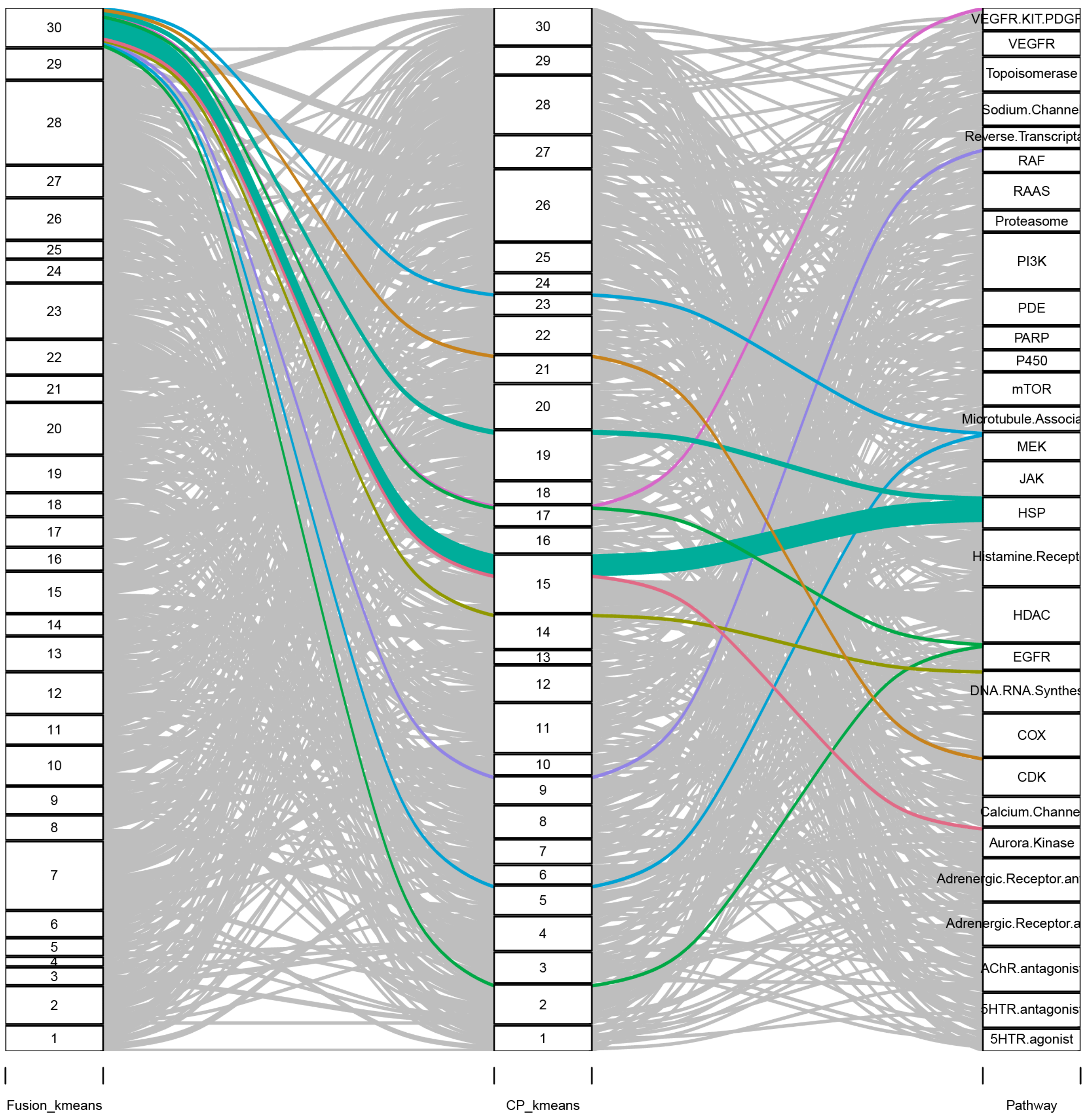
30
29
28
27
26
25
24
23
22
21
20
19
18
17
16
15
14
13
12
11
10
9
8
7
6
5
4
3
2
1
VEGFR.KIT.PDGF
VEGFR
Topoisomerase
Sodium.Channe
Reverse.Transcripta
RAF
RAAS
Proteasome
PI3K
PDE
PARP
P450
mTOR
Microtubule.Associa
MEK
JAK
HSP
Histamine.Recept
HDAC
EGFR
DNA.RNA.Synthes
COX
CDK
Calcium.Channe
Aurora.Kinase
Adrenergic.Receptor.an
Adrenergic.Receptor.a
AChR.antagonis
5HTR.antagonis
5HTR.agonist
Fusion_kmeans
CP_kmeans
Pathway

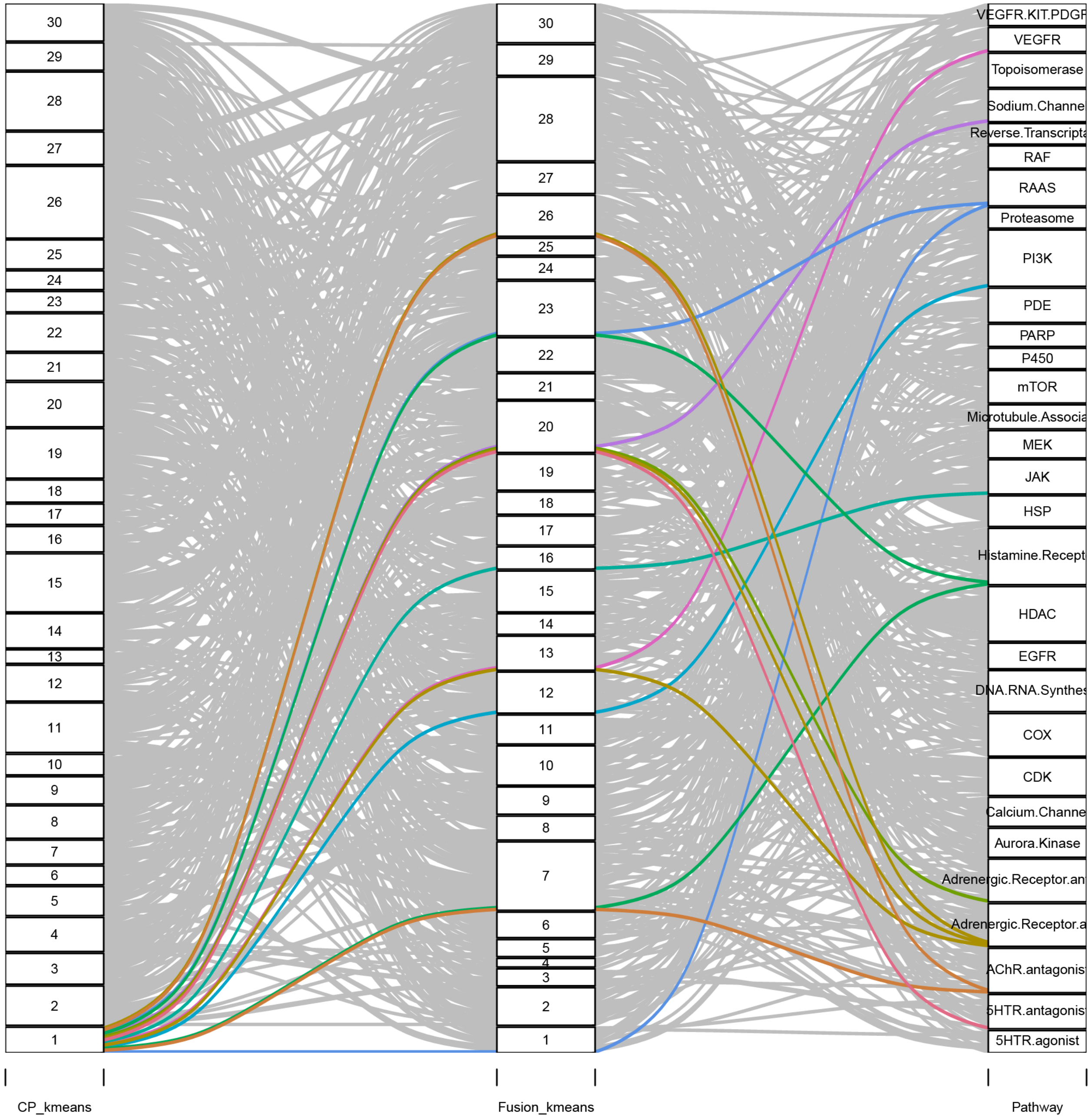

30
29
28
27
26
25
24
23
22
21
20
19
18
17
16
15
14
13
12
11
10
9
8
7
6
5
4
3
2
1
CP_kmeans
Fusion_kmeans
Pathway
VEGFR.KIT.PDGF
VEGFR
Topoisomerase
Sodium.Channel
Reverse.Transcripta
RAF
RAAS
Proteasome
PI3K
PDE
PARP
P450
mTOR
Microtubule.Associa
MEK
JAK
HSP
Histamine.Recept
HDAC
EGFR
DNA.RNA.Synthes
COX
CDK
Calcium.Channe
Aurora.Kinase
Adrenergic.Receptor.an
Adrenergic.Receptor.a
AChR.antagonis
5HTR.antagonis
5HTR.agonist

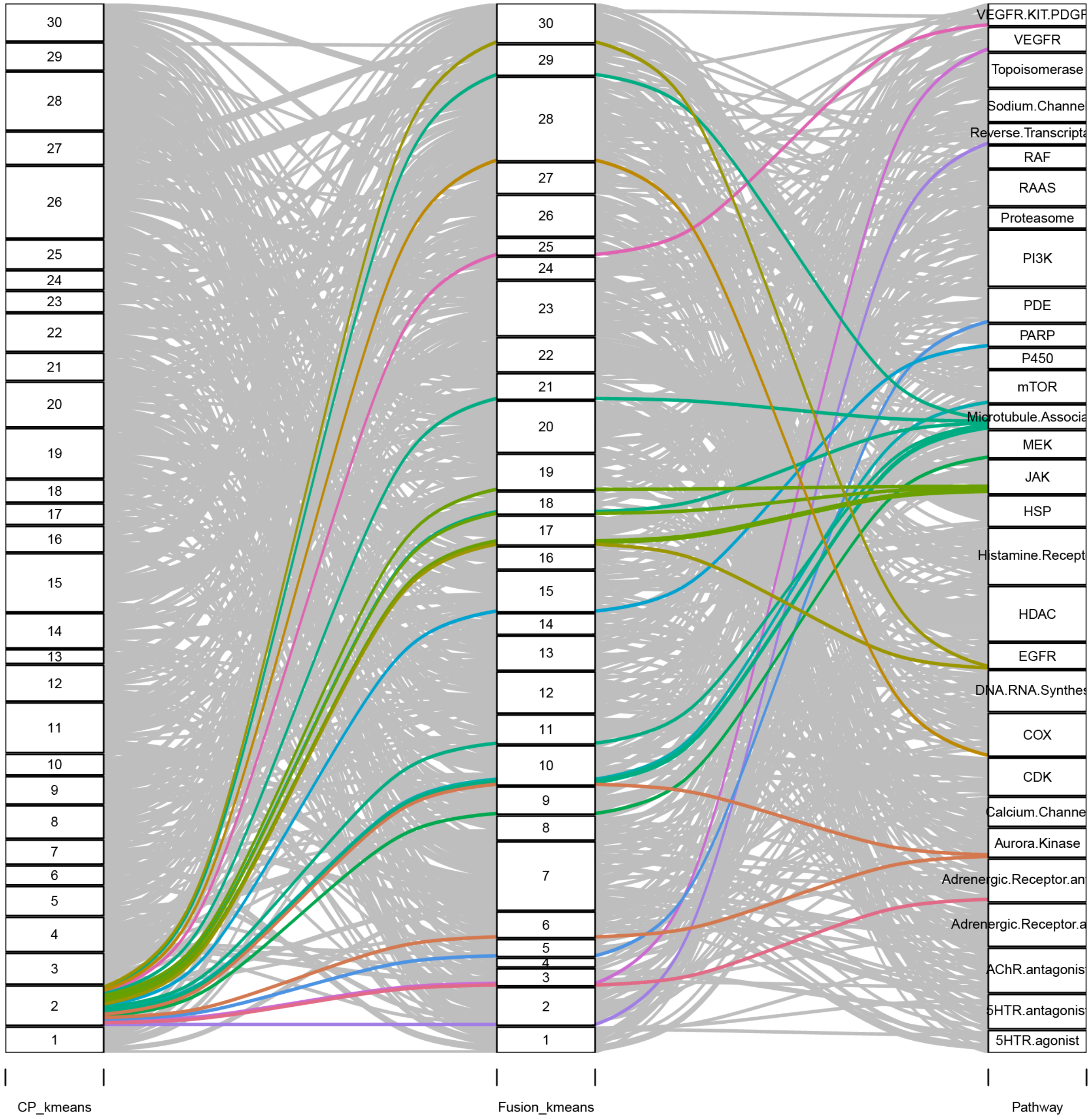
30
29
28
27
26
25
24
23
22
21
20
19
18
17
16
15
14
13
12
11
10
9
8
7
6
5
4
3
2
1
VEGFR.KIT.PDGF
VEGFR
Topoisomerase
Sodium.Channel
Reverse.Transcripta
RAF
RAAS
Proteasome
PI3K
PDE
PARP
P450
mTOR
Microtubule.Associa
MEK
JAK
HSP
Histamine.Recept
HDAC
EGFR
DNA.RNA.Synthes
COX
CDK
Calcium.Channe
Aurora.Kinase
Adrenergic.Receptor.an
Adrenergic.Receptor.a
AChR.antagonis
5HTR.antagonis
5HTR.agonist
CP_kmeans
Fusion_kmeans
Pathway

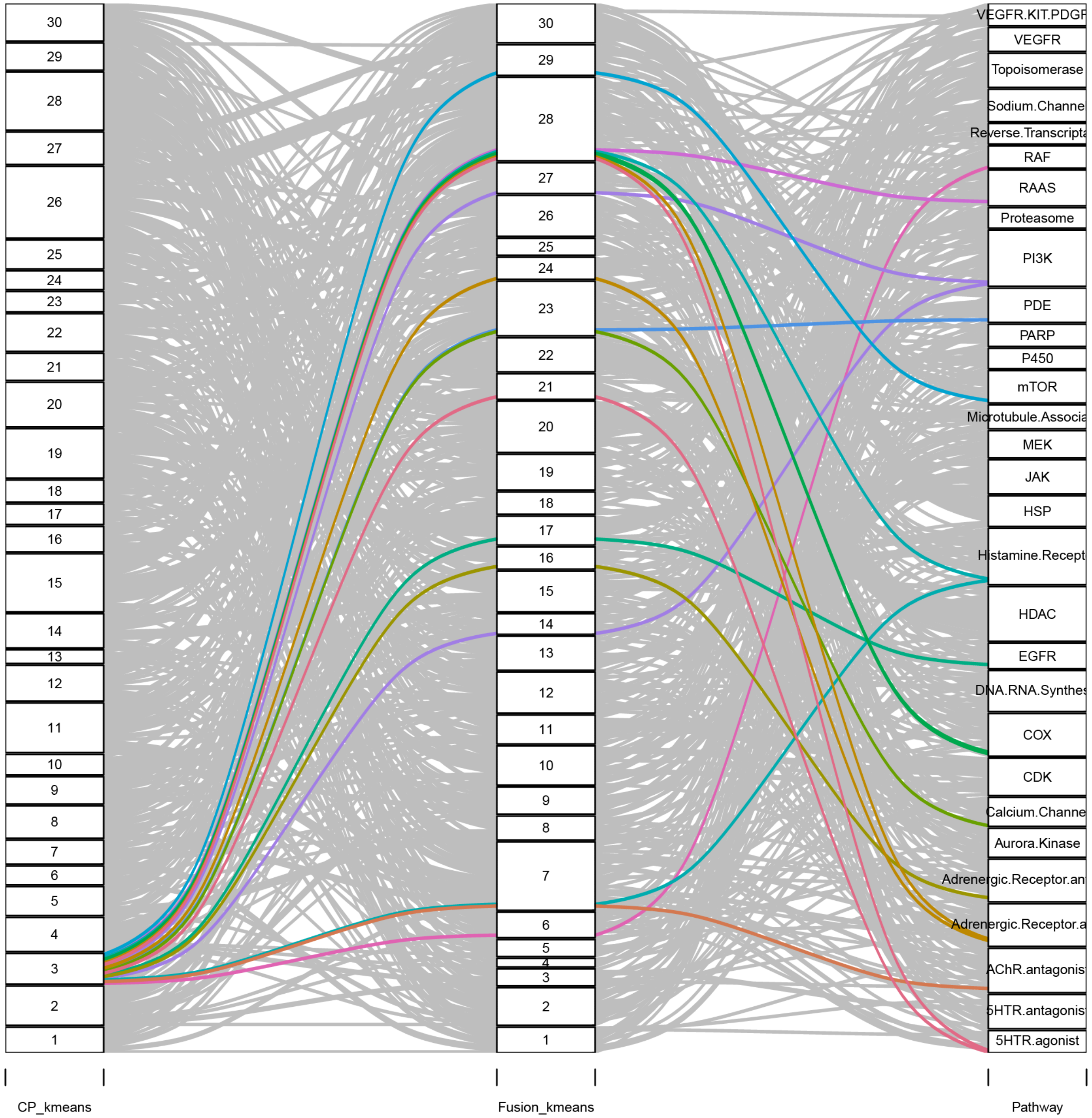

30
29
28
27
26
25
24
23
22
21
20
19
18
17
16
15
14
13
12
11
10
9
8
7
6
5
4
3
2
1
VEGFR.KIT.PDGF
VEGFR
Topoisomerase
Sodium.Channel
Reverse.Transcripta
RAF
RAAS
Proteasome
PI3K
PDE
PARP
P450
mTOR
Microtubule.Associa
MEK
JAK
HSP
Histamine.Recept
HDAC
EGFR
DNA.RNA.Synthes
COX
CDK
Calcium.Channe
Aurora.Kinase
Adrenergic.Receptor.ant
Adrenergic.Receptor.a
AChR.antagonist
5HTR.antagonist
5HTR.agonist
CP_kmeans
Fusion_kmeans
Pathway

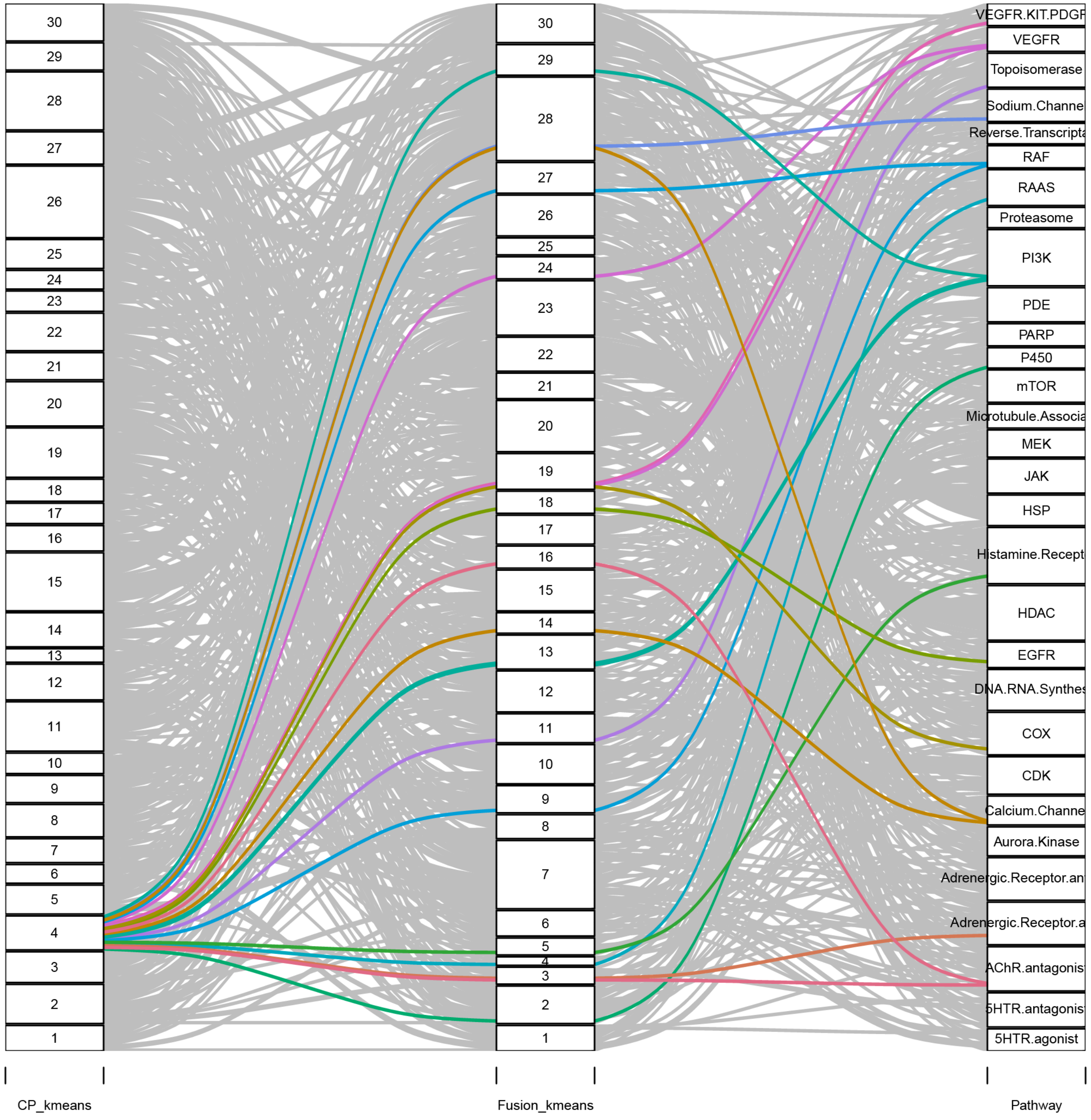
30
29
28
27
26
25
24
23
22
21
20
19
18
17
16
15
14
13
12
11
10
9
8
7
6
5
4
3
2
1
VEGFR.KIT.PDGF
VEGFR
Topoisomerase
Sodium.Channel
Reverse.Transcripta
RAF
RAAS
Proteasome
PI3K
PDE
PARP
P450
mTOR
Microtubule.Associa
MEK
JAK
HSP
Histamine.Recept
HDAC
EGFR
DNA.RNA.Synthes
COX
CDK
Calcium.Channe
Aurora.Kinase
Adrenergic.Receptor.an
Adrenergic.Receptor.a
AChR.antagonis
5HTR.antagonis
5HTR.agonist
CP_kmeans
Fusion_kmeans
Pathway

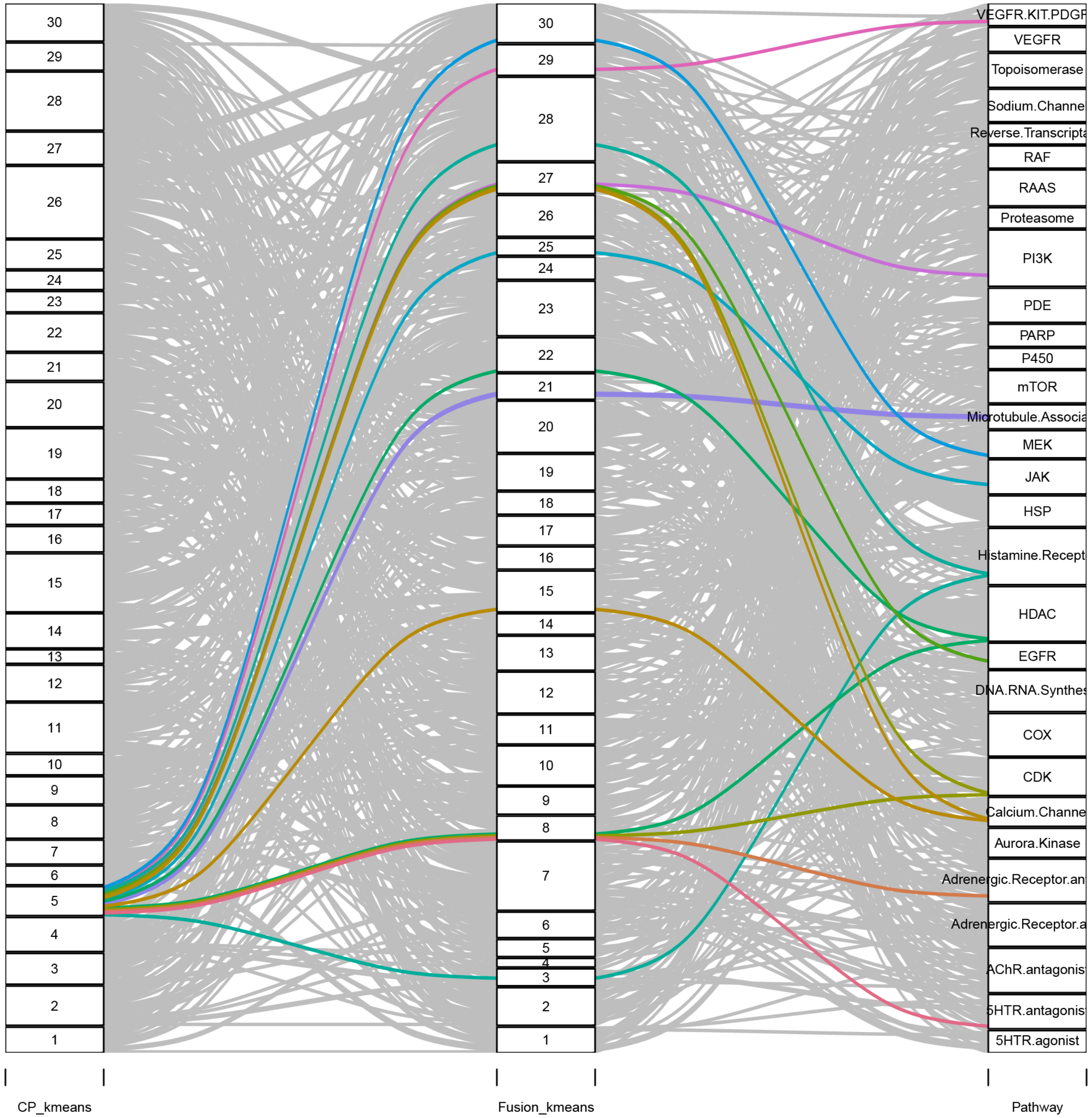
30
29
28
27
26
25
24
23
22
21
20
19
18
17
16
15
14
13
12
11
10
9
8
7
6
5
4
3
2
1
VEGFR.KIT.PDGF
VEGFR
Topoisomerase
Sodium.Channel
Reverse.Transcripta
RAF
RAAS
Proteasome
PI3K
PDE
PARP
P450
mTOR
Microtubule.Associa
MEK
JAK
HSP
Histamine.Recept
HDAC
EGFR
DNA.RNA.Synthes
COX
CDK
Calcium.Channe
Aurora.Kinase
Adrenergic.Receptor.an
Adrenergic.Receptor.a
AChR.antagonist
5HTR.antagonist
5HTR.agonist
CP_kmeans
Fusion_kmeans
Pathway

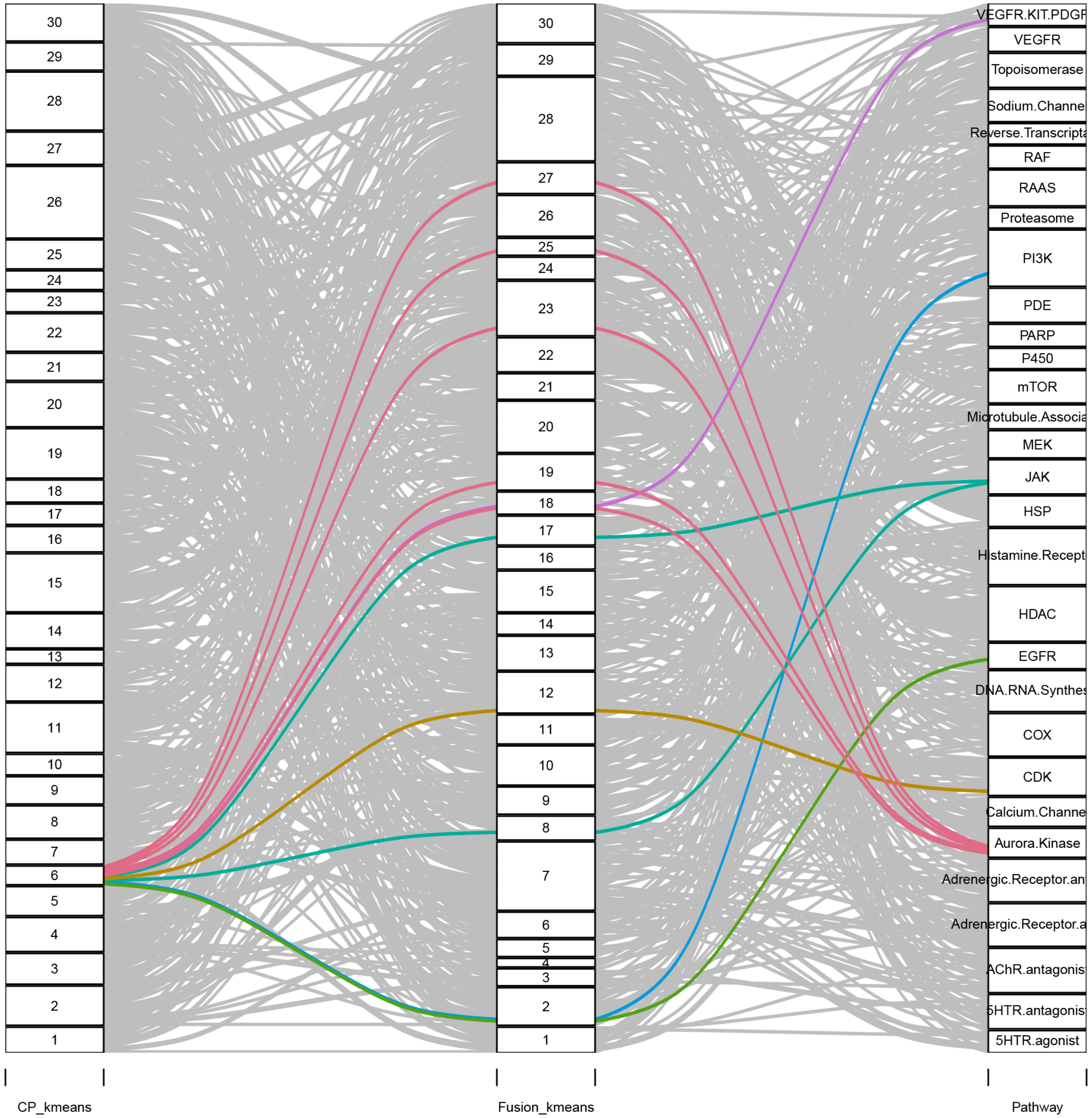
30
29
28
27
26
25
24
23
22
21
20
19
18
17
16
15
14
13
12
11
10
9
8
7
6
5
4
3
2
1
VEGFR.KIT.PDGF
VEGFR
Topoisomerase
Sodium.Channel
Reverse.Transcripta
RAF
RAAS
Proteasome
PI3K
PDE
PARP
P450
mTOR
Microtubule.Associa
MEK
JAK
HSP
Histamine.Recept
HDAC
EGFR
DNA.RNA.Synthes
COX
CDK
Calcium.Channe
Aurora.Kinase
Adrenergic.Receptor.an
Adrenergic.Receptor.a
AChR.antagonis
5HTR.antagonis
5HTR.agonist
CP_kmeans
Fusion_kmeans
Pathway

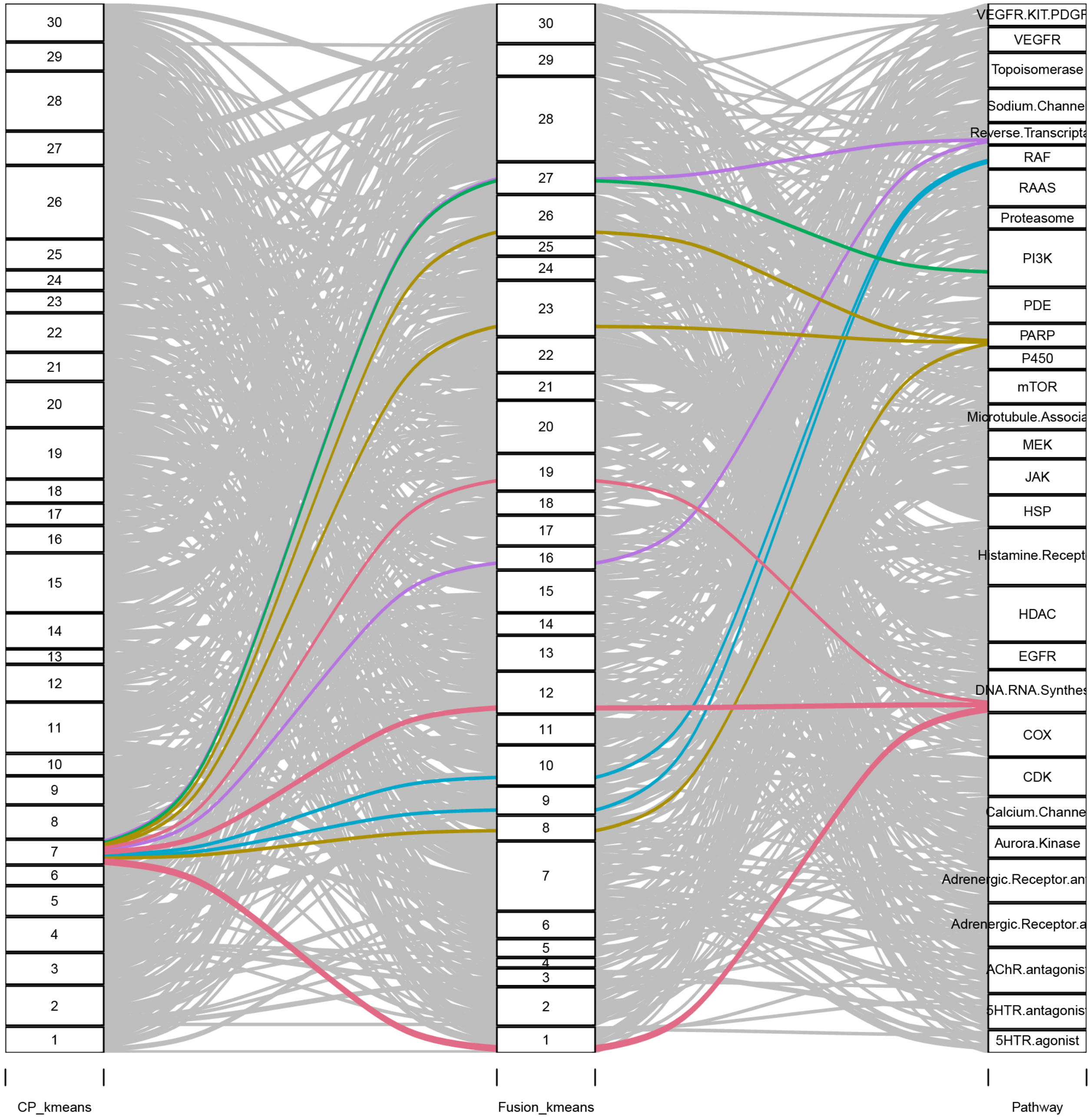
30
29
28
27
26
25
24
23
22
21
20
19
18
17
16
15
14
13
12
11
10
9
8
7
6
5
4
3
2
1
VEGFR.KIT.PDGF
VEGFR
Topoisomerase
Sodium.Channel
Reverse.Transcripta
RAF
RAAS
Proteasome
PI3K
PDE
PARP
P450
mTOR
Microtubule.Associa
MEK
JAK
HSP
Histamine.Recept
HDAC
EGFR
DNA.RNA.Synthes
COX
CDK
Calcium.Channe
Aurora.Kinase
Adrenergic.Receptor.an
Adrenergic.Receptor.a
AChR.antagonis
5HTR.antagonis
5HTR.agonist
CP_kmeans
Fusion_kmeans
Pathway

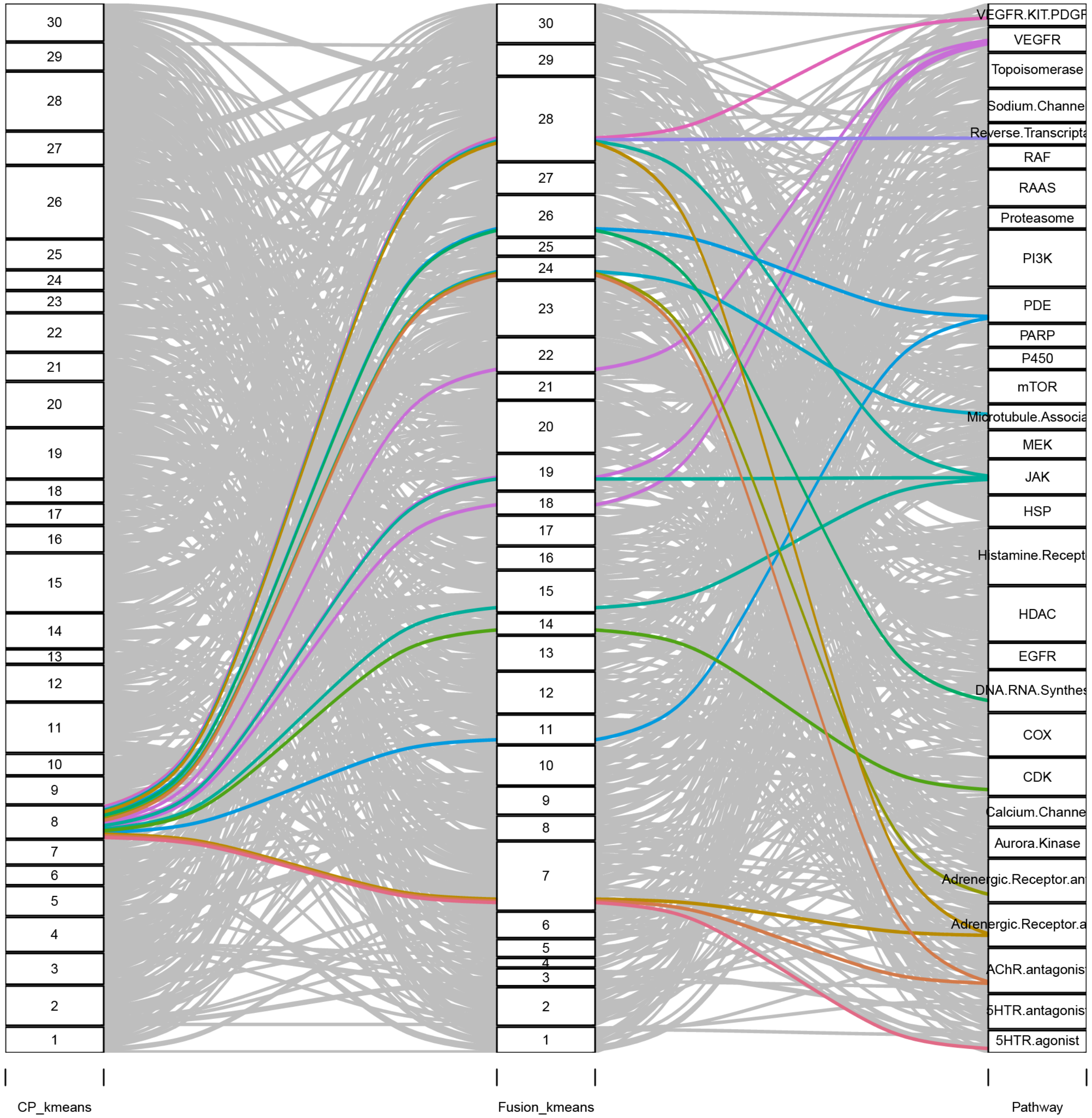
30
29
28
27
26
25
24
23
22
21
20
19
18
17
16
15
14
13
12
11
10
9
8
7
6
5
4
3
2
1
VEGFR.KIT.PDGF
VEGFR
Topoisomerase
Sodium.Channel
Reverse.Transcripta
RAF
RAAS
Proteasome
PI3K
PDE
PARP
P450
mTOR
Microtubule.Associa
MEK
JAK
HSP
Histamine.Recept
HDAC
EGFR
DNA.RNA.Synthes
COX
CDK
Calcium.Channe
Aurora.Kinase
Adrenergic.Receptor.ant
Adrenergic.Receptor.a
AChR.antagonist
5HTR.antagonist
5HTR.agonist
CP_kmeans
Fusion_kmeans
Pathway

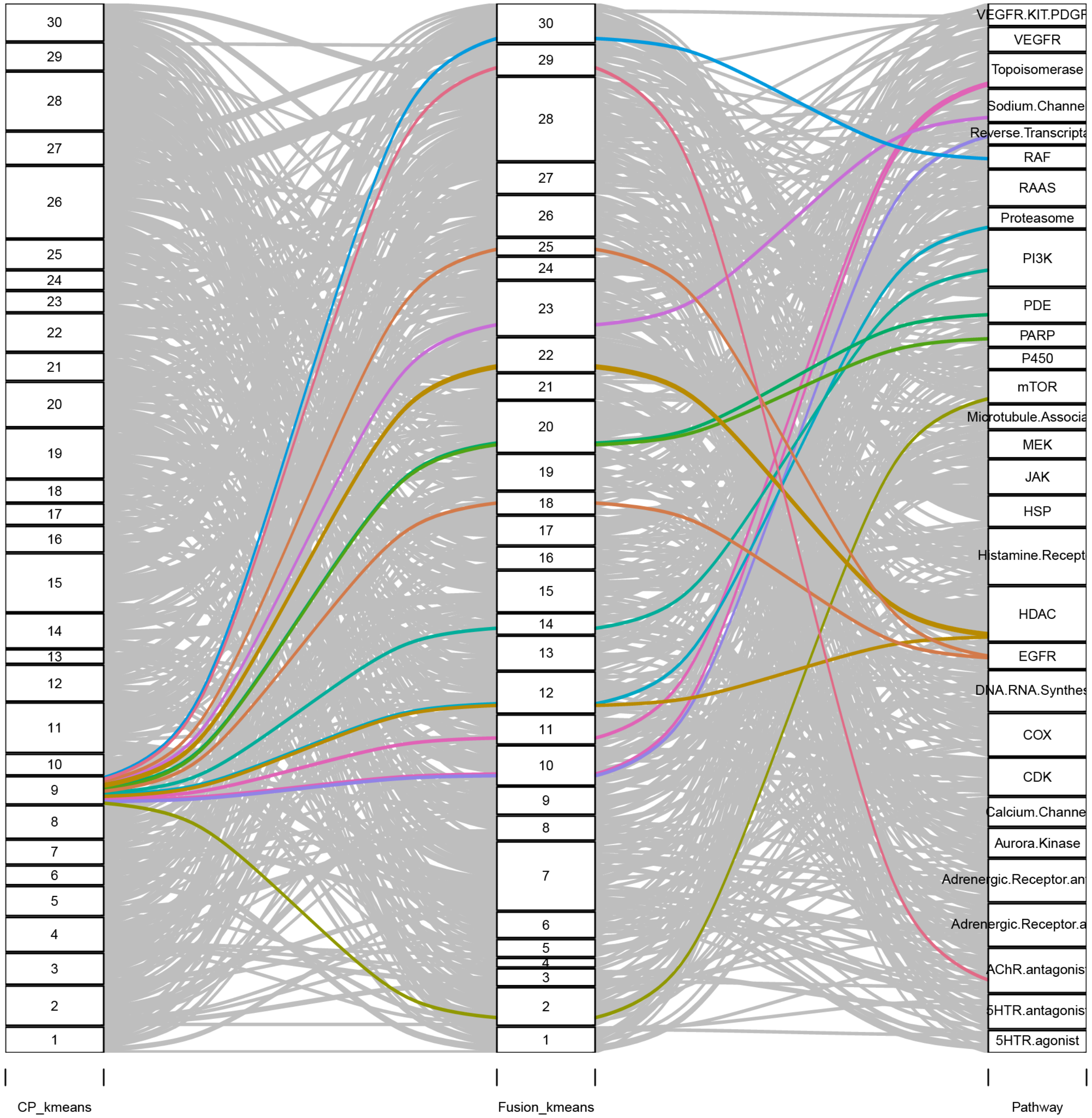

30
29
28
27
26
25
24
23
22
21
20
19
18
17
16
15
14
13
12
11
10
9
8
7
6
5
4
3
2
1
VEGFR.KIT.PDGF
VEGFR
Topoisomerase
Sodium.Channel
Reverse.Transcripta
RAF
RAAS
Proteasome
PI3K
PDE
PARP
P450
mTOR
Microtubule.Associa
MEK
JAK
HSP
Histamine.Recept
HDAC
EGFR
DNA.RNA.Synthes
COX
CDK
Calcium.Channe
Aurora.Kinase
Adrenergic.Receptor.an
Adrenergic.Receptor.a
AChR.antagonis
5HTR.antagonis
5HTR.agonist
CP_kmeans
Fusion_kmeans
Pathway

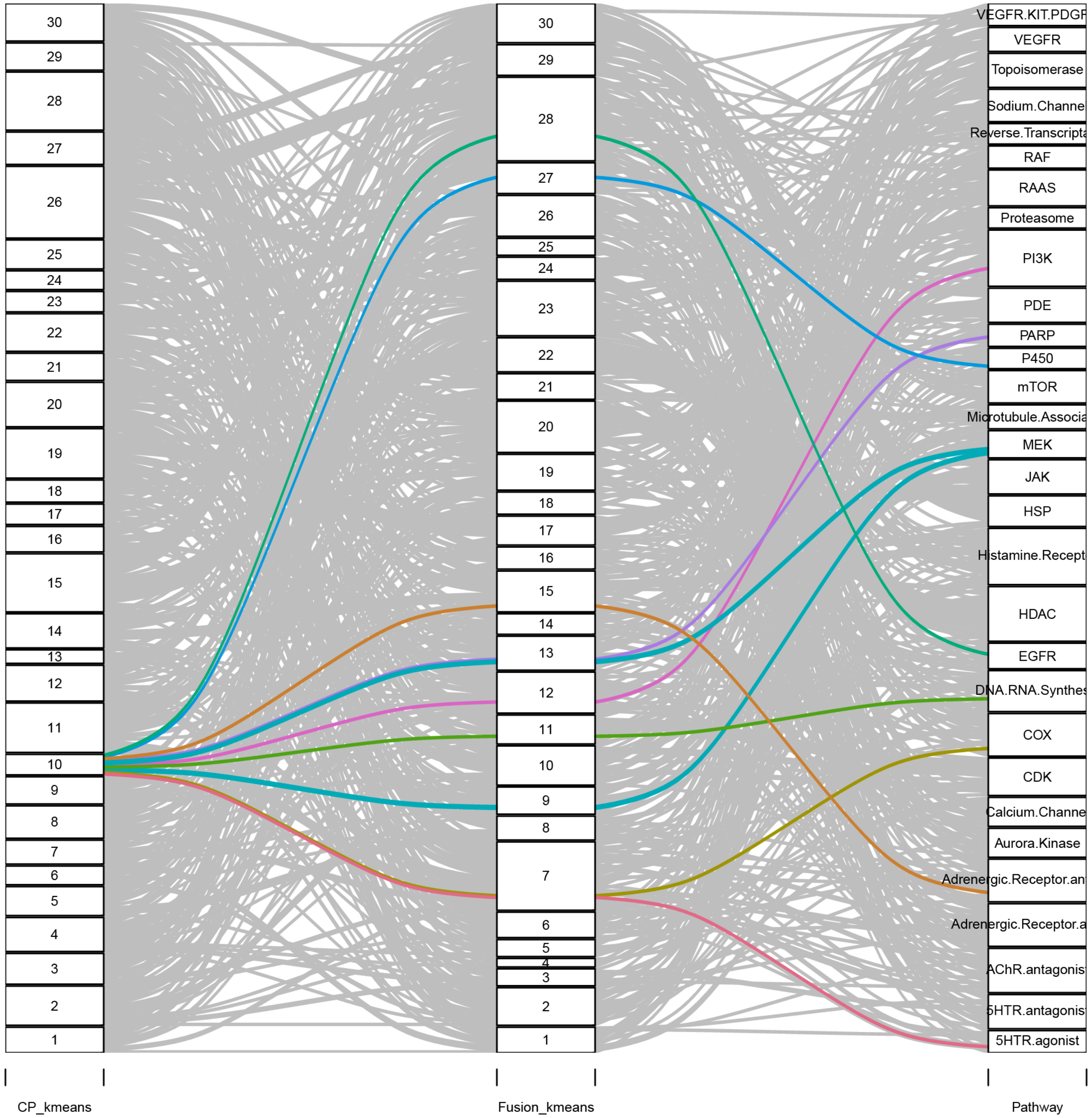
CP_kmeans
Fusion_kmeans
Pathway
VEGFR.KIT.PDGF
VEGFR
Topoisomerase
Sodium.Channel
Reverse.Transcripta
RAF
RAAS
Proteasome
PI3K
PDE
PARP
P450
mTOR
Microtubule.Associa
MEK
JAK
HSP
Histamine.Recept
HDAC
EGFR
DNA.RNA.Synthes
COX
CDK
Calcium.Channe
Aurora.Kinase
Adrenergic.Receptor.an
Adrenergic.Receptor.a
AChR.antagonis
5HTR.antagonis
5HTR.agonist

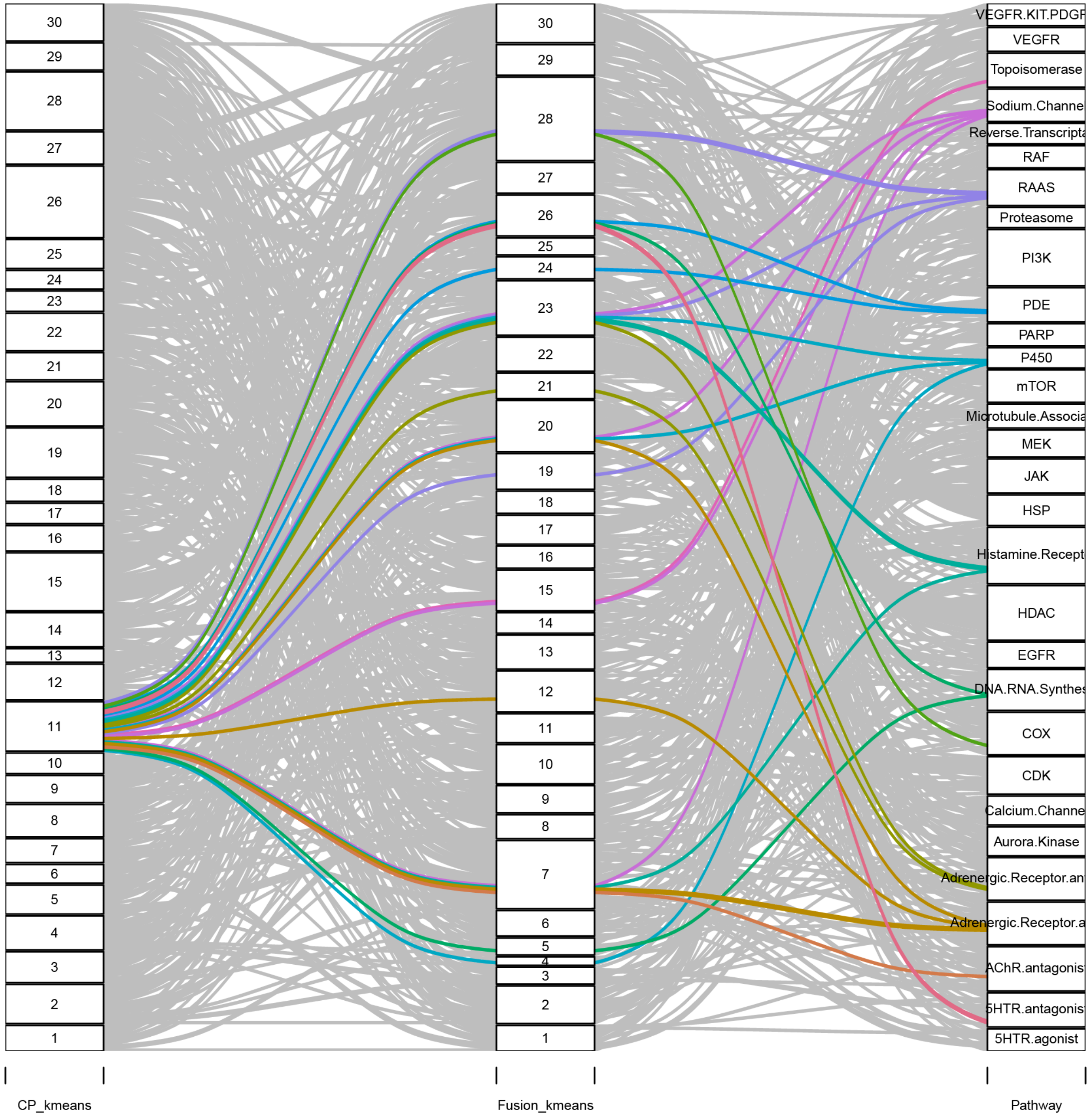
CP_kmeans
Fusion_kmeans
Pathway
30
29
28
27
26
25
24
23
22
21
20
19
18
17
16
15
14
13
12
11
10
9
8
7
6
5
4
3
2
1
VEGFR.KIT.PDGF
VEGFR
Topoisomerase
Sodium.Channel
Reverse.Transcripta
RAF
RAAS
Proteasome
PI3K
PDE
PARP
P450
mTOR
Microtubule.Associa
MEK
JAK
HSP
Histamine.Recept
HDAC
EGFR
DNA.RNA.Synthes
COX
CDK
Calcium.Channe
Aurora.Kinase
Adrenergic.Receptor.an
Adrenergic.Receptor.a
AChR.antagonis
5HTR.antagonis
5HTR.agonist

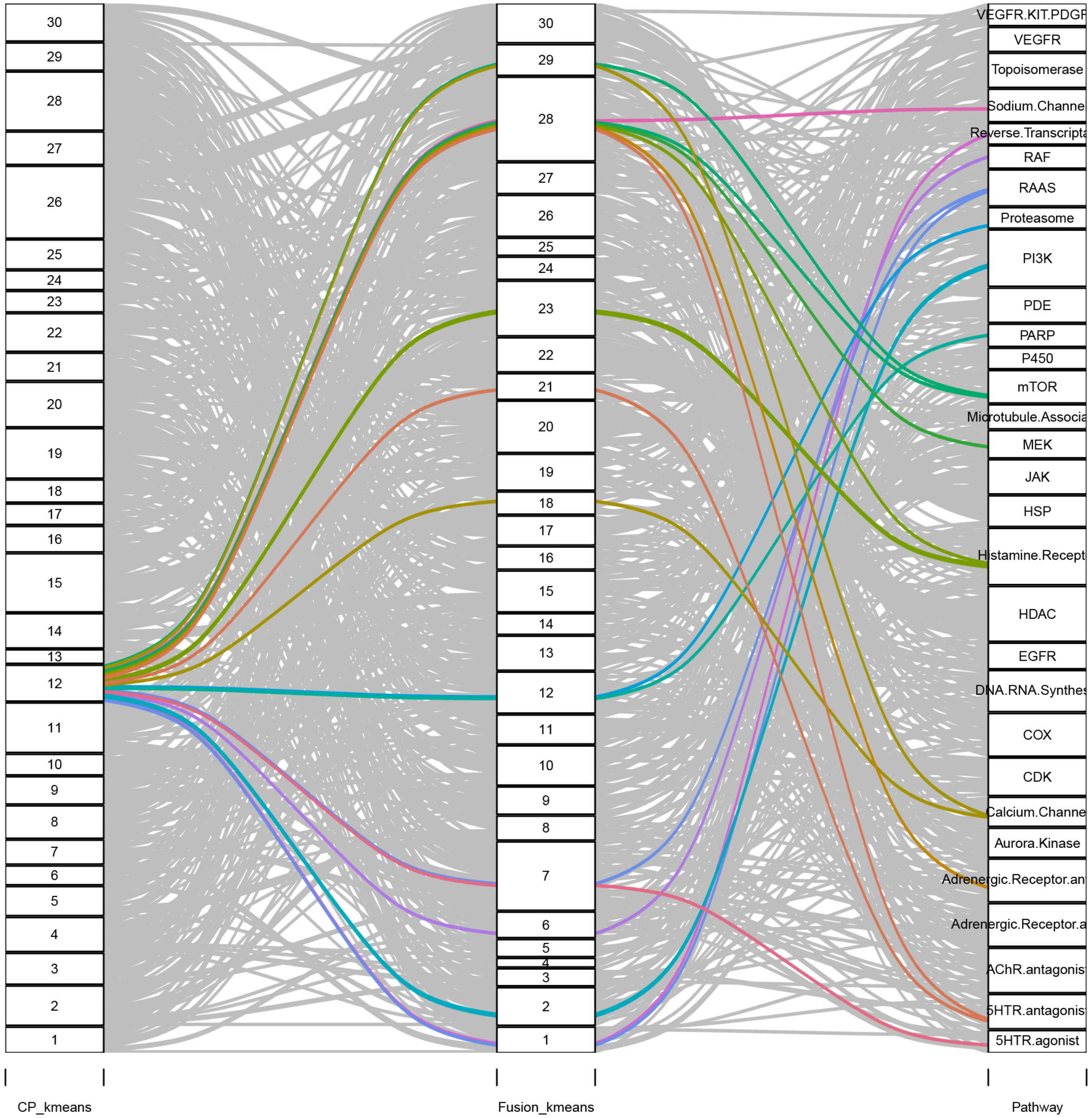
CP_kmeans
Fusion_kmeans
Pathway
VEGFR.KIT.PDGF
VEGFR
Topoisomerase
Sodium.Channel
Reverse.Transcripta
RAF
RAAS
Proteasome
PI3K
PDE
PARP
P450
mTOR
Microtubule.Associa
MEK
JAK
HSP
Histamine.Recept
HDAC
EGFR
DNA.RNA.Synthes
COX
CDK
Calcium.Channe
Aurora.Kinase
Adrenergic.Receptor.an
Adrenergic.Receptor.a
AChR.antagonis
5HTR.antagonis
5HTR.agonist

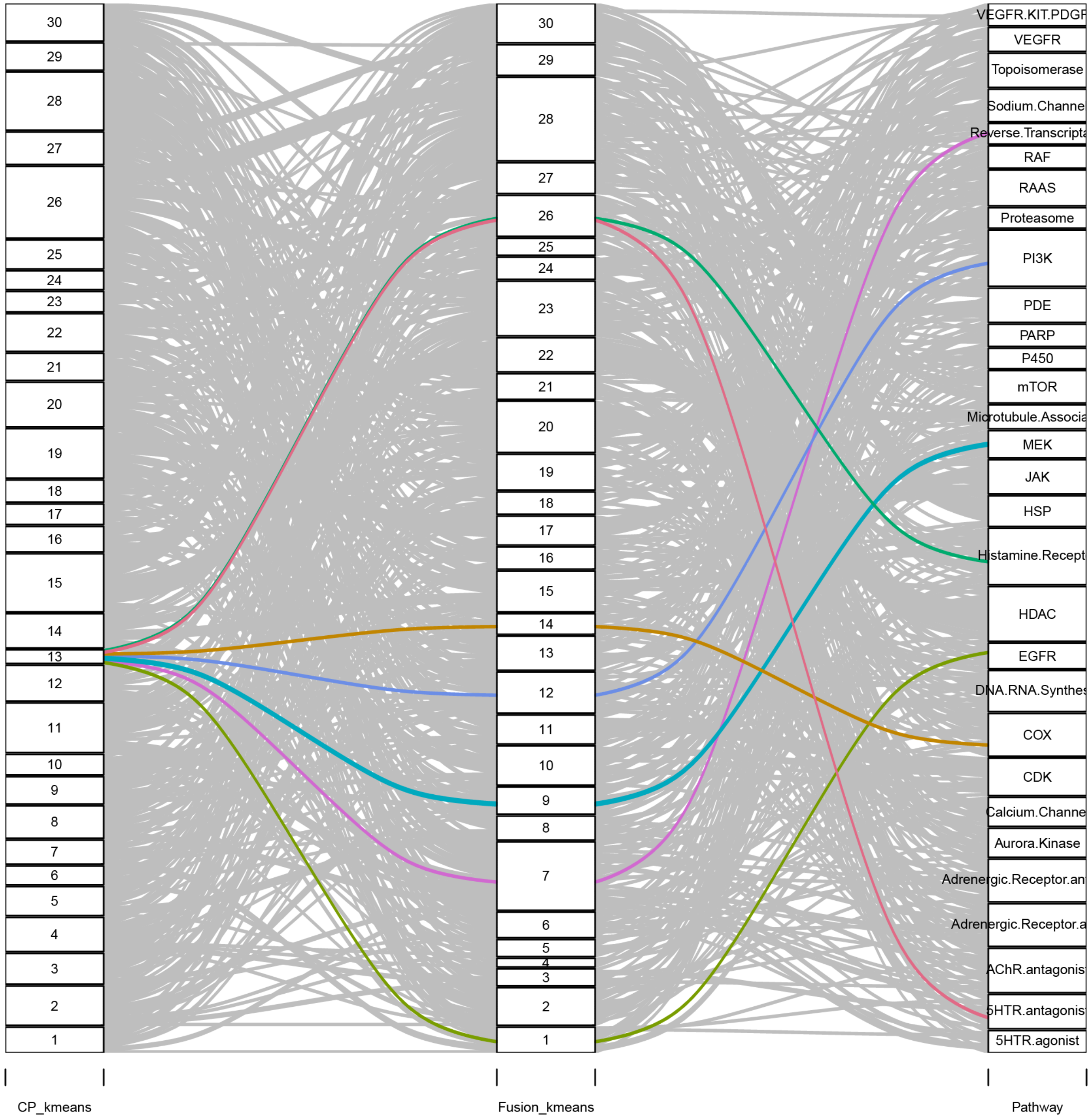

30
29
28
27
26
25
24
23
22
21
20
19
18
17
16
15
14
13
12
11
10
9
8
7
6
5
4
3
2
1
VEGFR.KIT.PDGF
VEGFR
Topoisomerase
Sodium.Channel
Reverse.Transcripta
RAF
RAAS
Proteasome
PI3K
PDE
PARP
P450
mTOR
Microtubule.Associa
MEK
JAK
HSP
Histamine.Recept
HDAC
EGFR
DNA.RNA.Synthes
COX
CDK
Calcium.Channe
Aurora.Kinase
Adrenergic.Receptor.an
Adrenergic.Receptor.a
AChR.antagonis
5HTR.antagonis
5HTR.agonist
CP_kmeans
Fusion_kmeans
Pathway

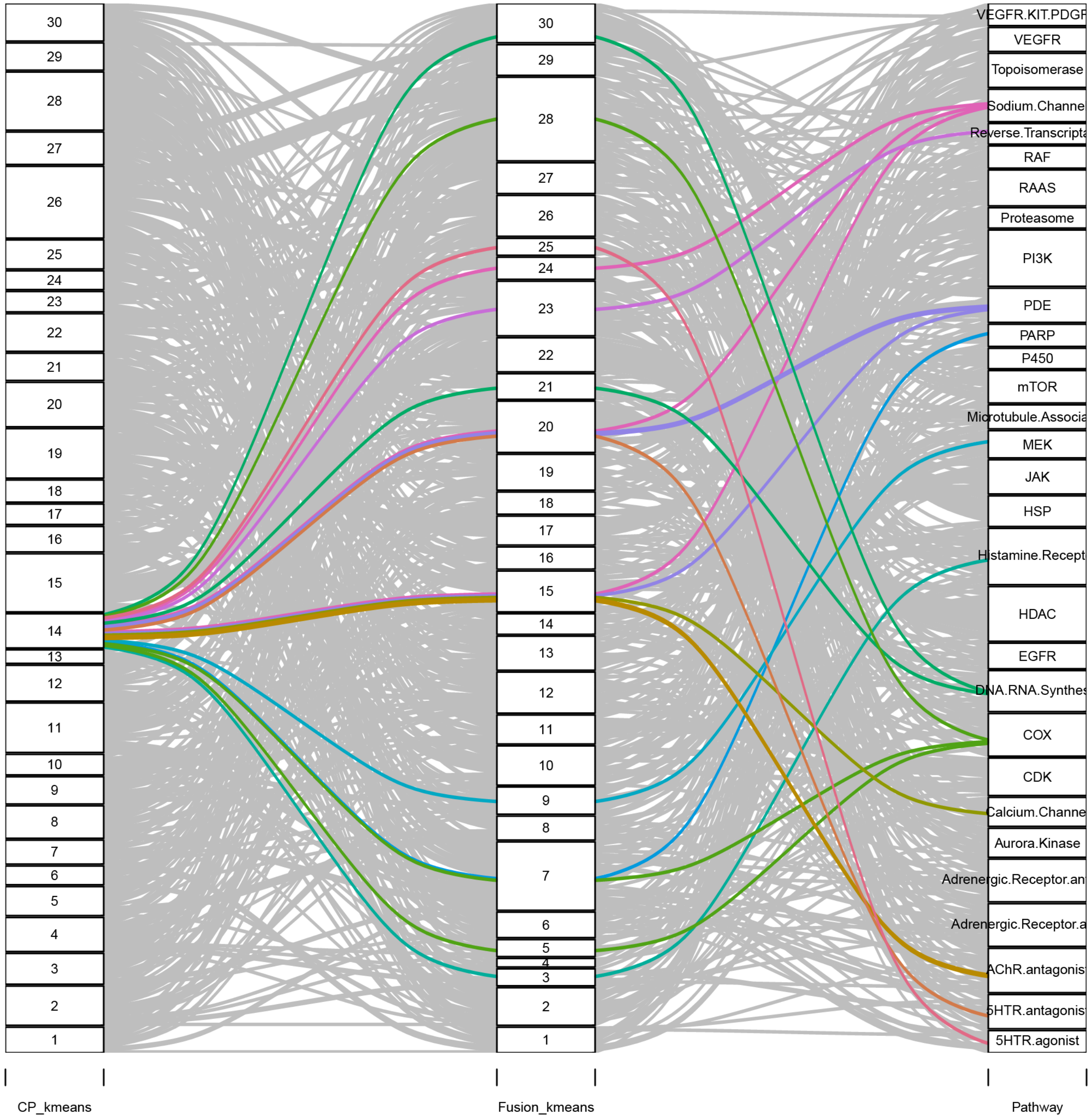
30
29
28
27
26
25
24
23
22
21
20
19
18
17
16
15
14
13
12
11
10
9
8
7
6
5
4
3
2
1
VEGFR.KIT.PDGF
VEGFR
Topoisomerase
Sodium.Channel
Reverse.Transcripta
RAF
RAAS
Proteasome
PI3K
PDE
PARP
P450
mTOR
Microtubule.Associa
MEK
JAK
HSP
Histamine.Recept
HDAC
EGFR
DNA.RNA.Synthes
COX
CDK
Calcium.Channe
Aurora.Kinase
Adrenergic.Receptor.an
Adrenergic.Receptor.a
AChR.antagonis
5HTR.antagonis
5HTR.agonist
CP_kmeans
Fusion_kmeans
Pathway

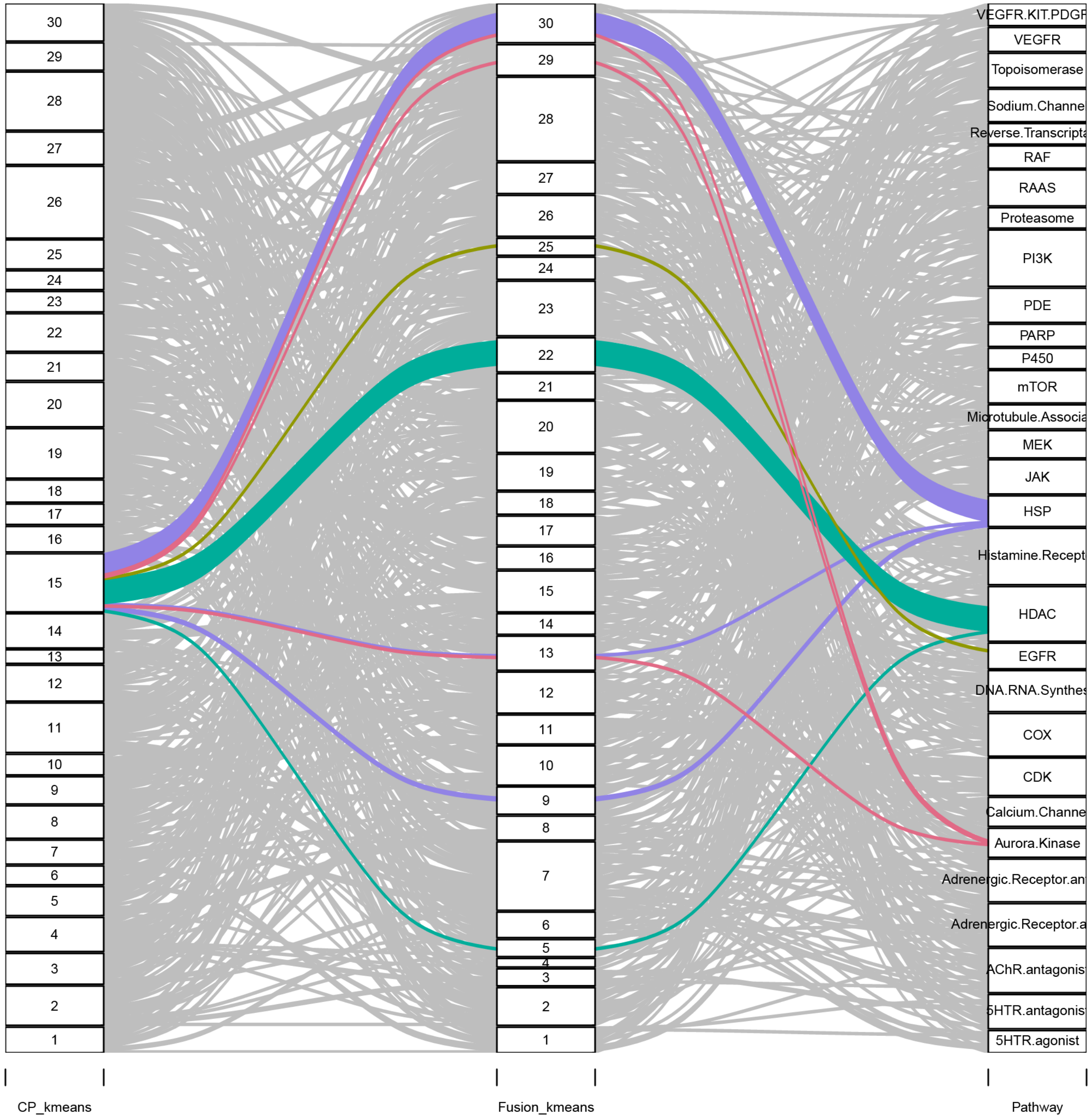
CP_kmeans
Fusion_kmeans
Pathway
30
29
28
27
26
25
24
23
22
21
20
19
18
17
16
15
14
13
12
11
10
9
8
7
6
5
4
3
2
1
VEGFR.KIT.PDGF
VEGFR
Topoisomerase
Sodium.Channel
Reverse.Transcripta
RAF
RAAS
Proteasome
PI3K
PDE
PARP
P450
mTOR
Microtubule.Associa
MEK
JAK
HSP
Histamine.Recept
HDAC
EGFR
DNA.RNA.Synthes
COX
CDK
Calcium.Channe
Aurora.Kinase
Adrenergic.Receptor.an
Adrenergic.Receptor.a
AChR.antagonis
5HTR.antagonis
5HTR.agonist

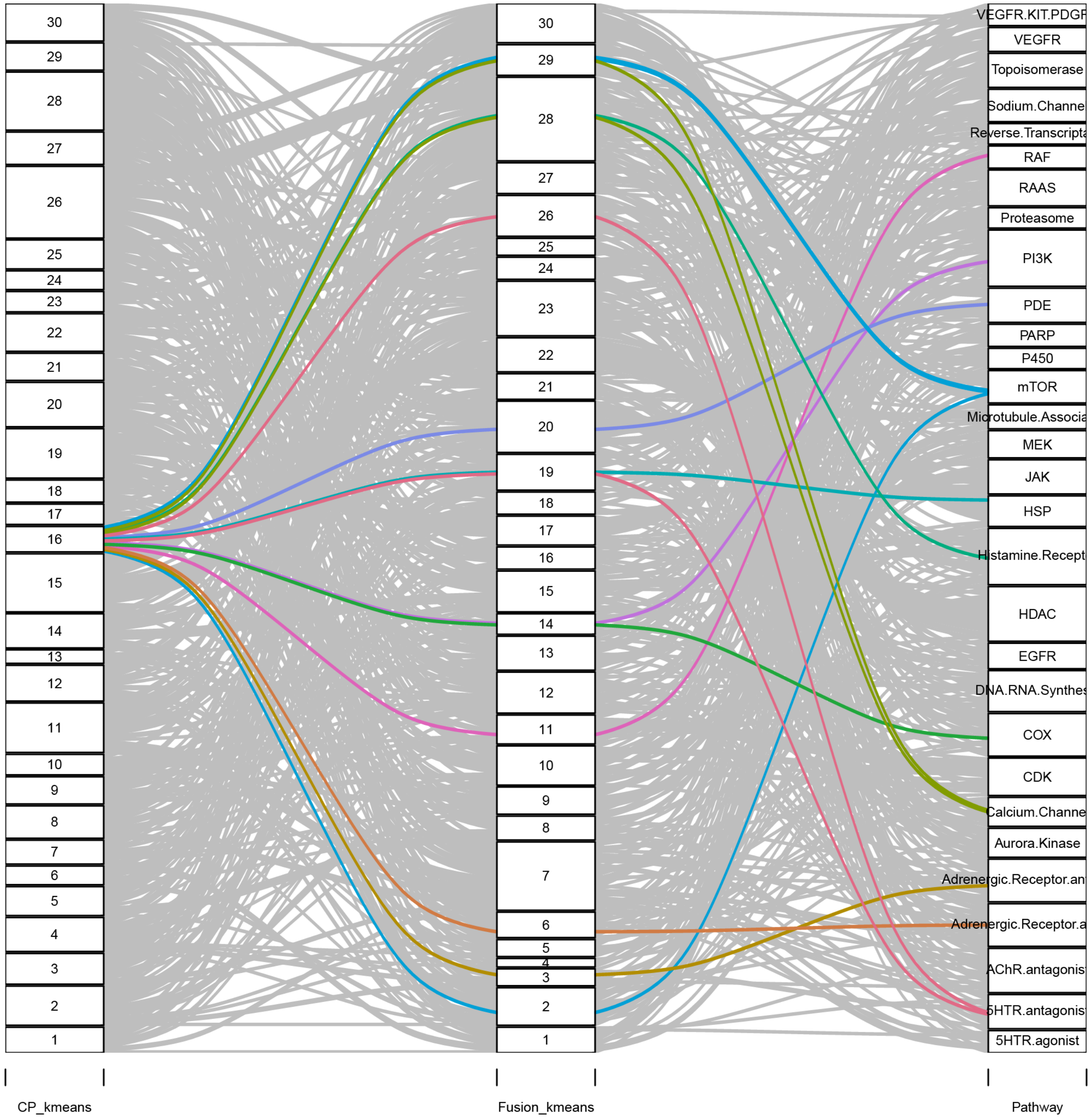

CP_kmeans
Fusion_kmeans
Pathway
VEGFR.KIT.PDGF
VEGFR
Topoisomerase
Sodium.Channe
Reverse.Transcripta
RAF
RAAS
Proteasome
PI3K
PDE
PARP
P450
mTOR
Microtubule.Associa
MEK
JAK
HSP
Histamine.Recept
HDAC
EGFR
DNA.RNA.Synthes
COX
CDK
Calcium.Channe
Aurora.Kinase
Adrenergic.Receptor.an
Adrenergic.Receptor.a
AChR.antagonis
5HTR.antagonis
5HTR.agonist

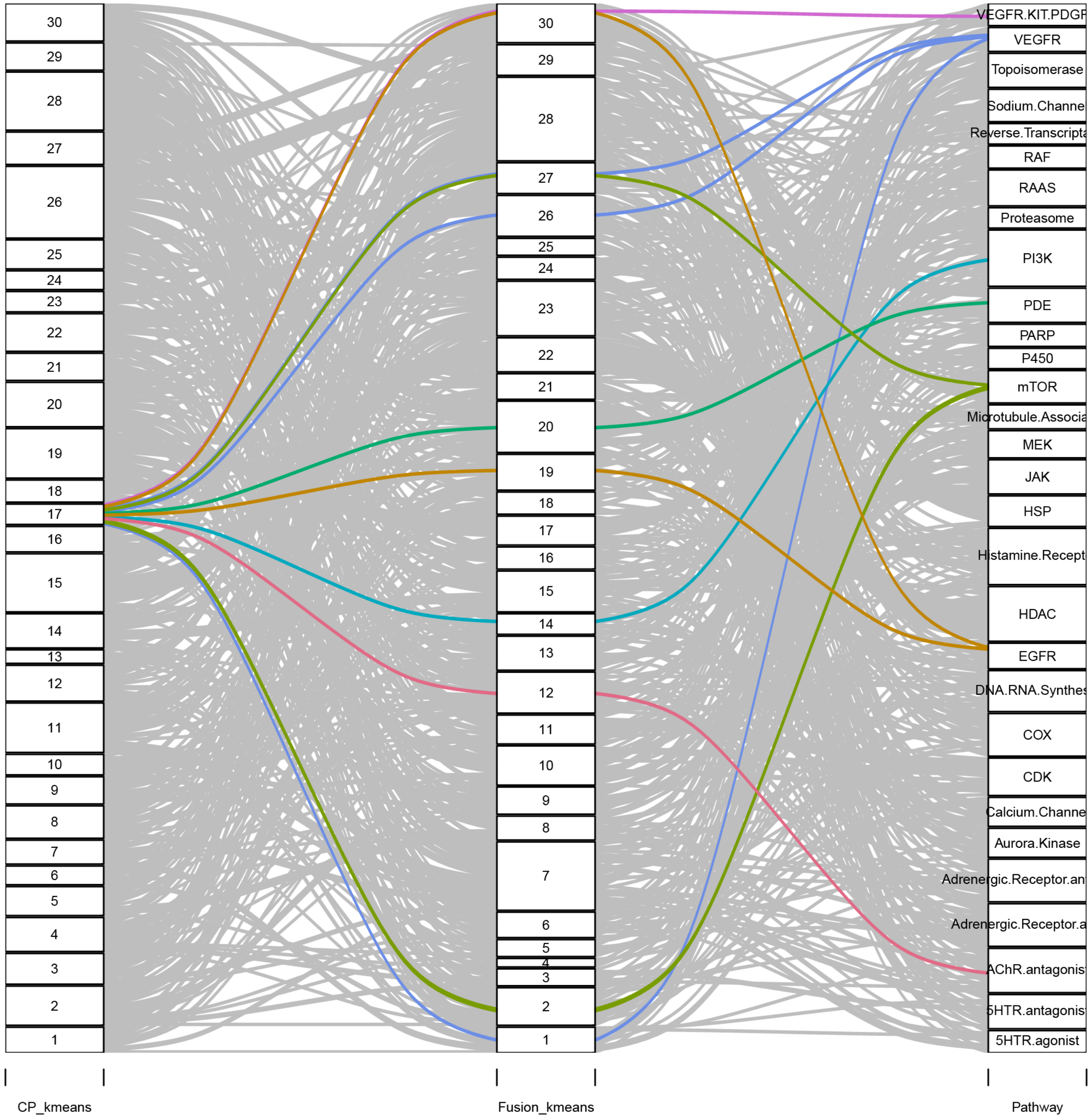
CP_kmeans
Fusion_kmeans
Pathway
30
29
28
27
26
25
24
23
22
21
20
19
18
17
16
15
14
13
12
11
10
9
8
7
6
5
4
3
2
1
VEGFR.KIT.PDGF
VEGFR
Topoisomerase
Sodium.Channel
Reverse.Transcripta
RAF
RAAS
Proteasome
PI3K
PDE
PARP
P450
mTOR
Microtubule.Associa
MEK
JAK
HSP
Histamine.Recept
HDAC
EGFR
DNA.RNA.Synthes
COX
CDK
Calcium.Channe
Aurora.Kinase
Adrenergic.Receptor.an
Adrenergic.Receptor.a
AChR.antagonis
5HTR.antagonis
5HTR.agonist

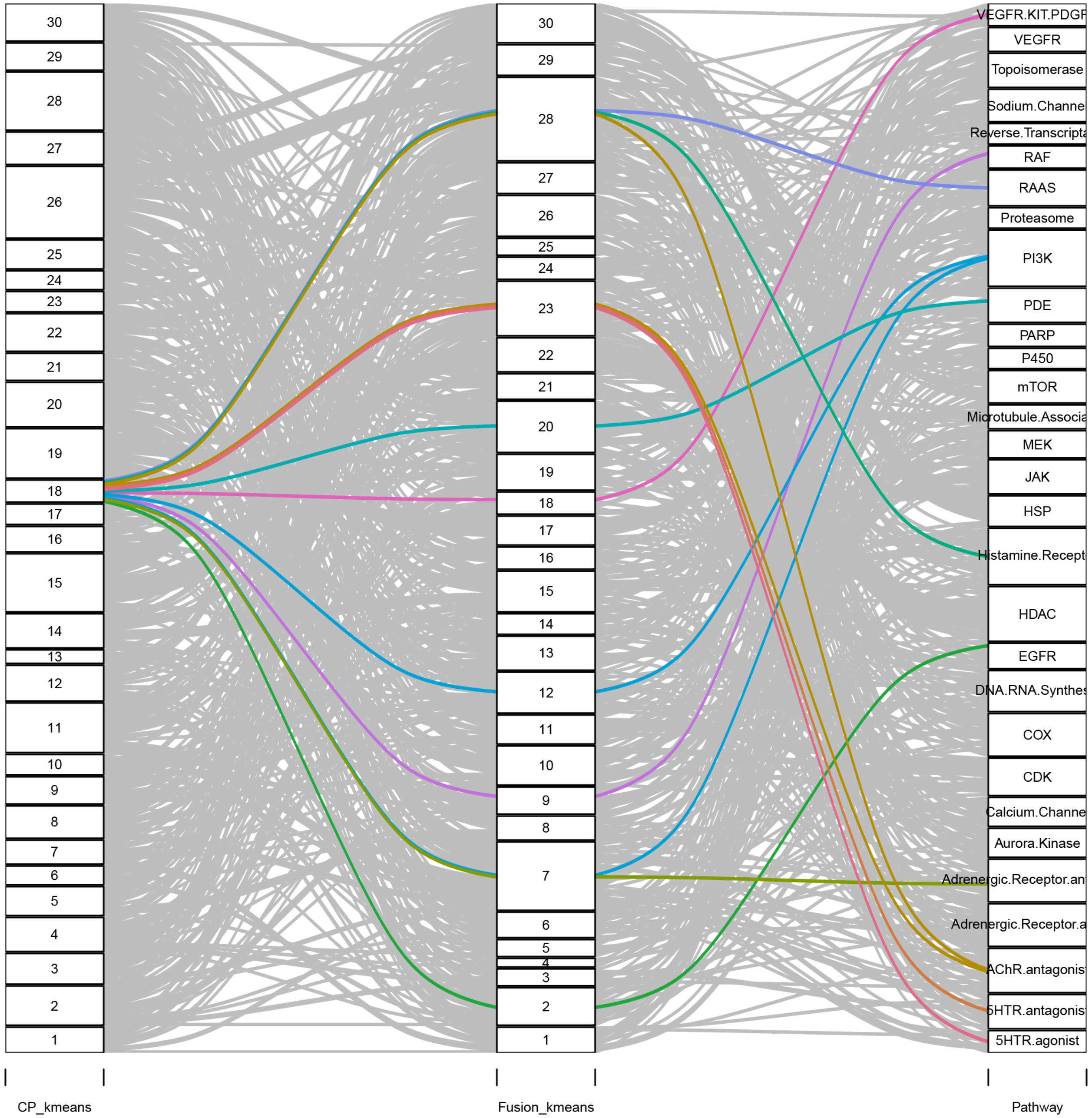

30
29
28
27
26
25
24
23
22
21
20
19
18
17
16
15
14
13
12
11
10
9
8
7
6
5
4
3
2
1
VEGFR.KIT.PDGF
VEGFR
Topoisomerase
Sodium.Channel
Reverse.Transcripta
RAF
RAAS
Proteasome
PI3K
PDE
PARP
P450
mTOR
Microtubule.Associa
MEK
JAK
HSP
Histamine.Recept
HDAC
EGFR
DNA.RNA.Synthes
COX
CDK
Calcium.Channe
Aurora.Kinase
Adrenergic.Receptor.ant
Adrenergic.Receptor.a
AChR.antagonis
5HTR.antagonis
5HTR.agonist
CP_kmeans
Fusion_kmeans
Pathway

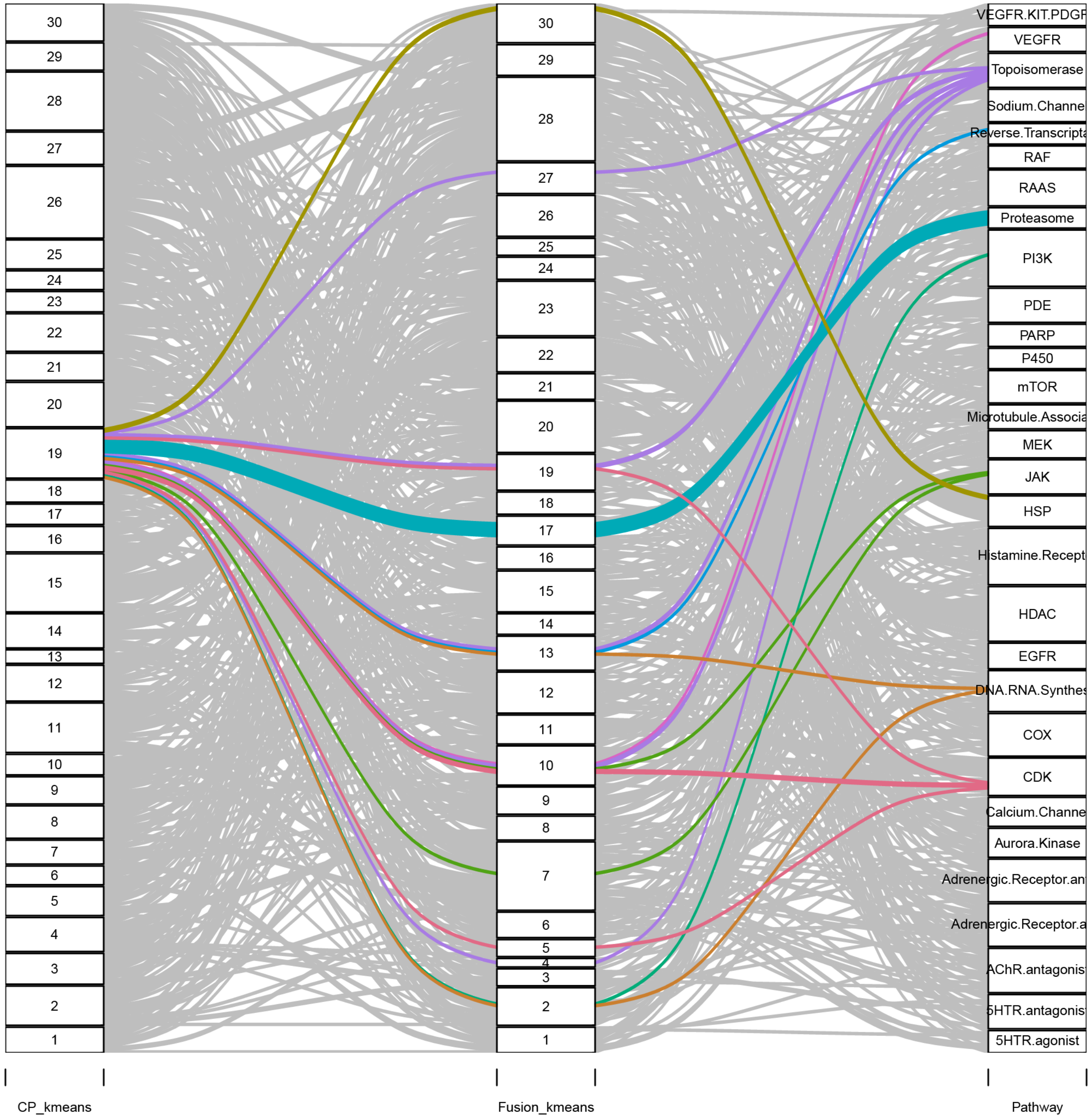

30
29
28
27
26
25
24
23
22
21
20
19
18
17
16
15
14
13
12
11
10
9
8
7
6
5
4
3
2
1
VEGFR.KIT.PDGF
VEGFR
Topoisomerase
Sodium.Channel
Reverse.Transcripta
RAF
RAAS
Proteasome
PI3K
PDE
PARP
P450
mTOR
Microtubule.Associa
MEK
JAK
HSP
Histamine.Recept
HDAC
EGFR
DNA.RNA.Synthes
COX
CDK
Calcium.Channe
Aurora.Kinase
Adrenergic.Receptor.an
Adrenergic.Receptor.a
AChR.antagonist
5HTR.antagonist
5HTR.agonist
CP_kmeans
Fusion_kmeans
Pathway

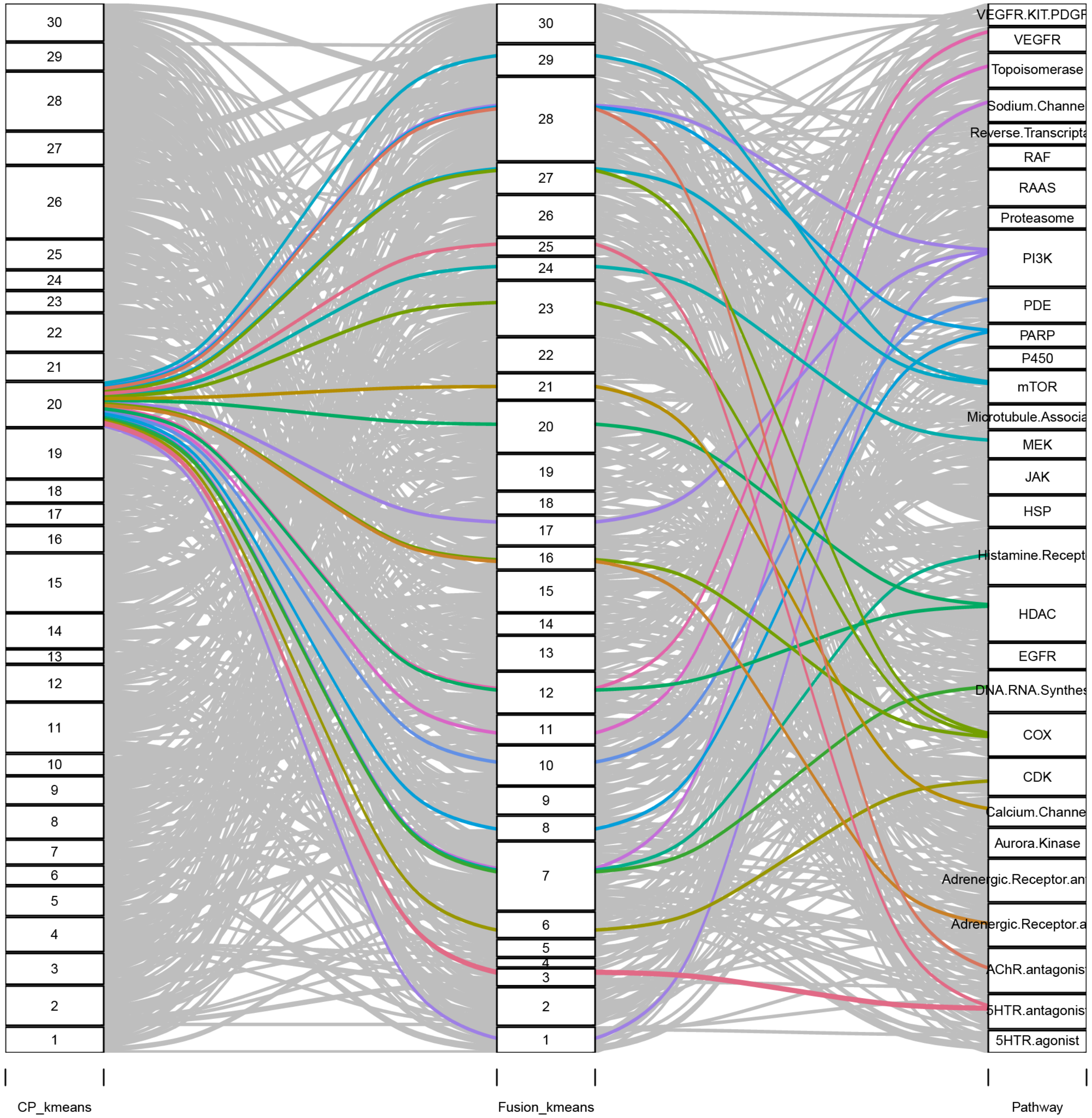

CP_kmeans
Fusion_kmeans
Pathway
VEGFR.KIT.PDGF
VEGFR
Topoisomerase
Sodium.Channel
Reverse.Transcripta
RAF
RAAS
Proteasome
PI3K
PDE
PARP
P450
mTOR
Microtubule.Associa
MEK
JAK
HSP
Histamine.Recept
HDAC
EGFR
DNA.RNA.Synthes
COX
CDK
Calcium.Channe
Aurora.Kinase
Adrenergic.Receptor.an
Adrenergic.Receptor.a
AChR.antagonis
5HTR.antagonis
5HTR.agonist

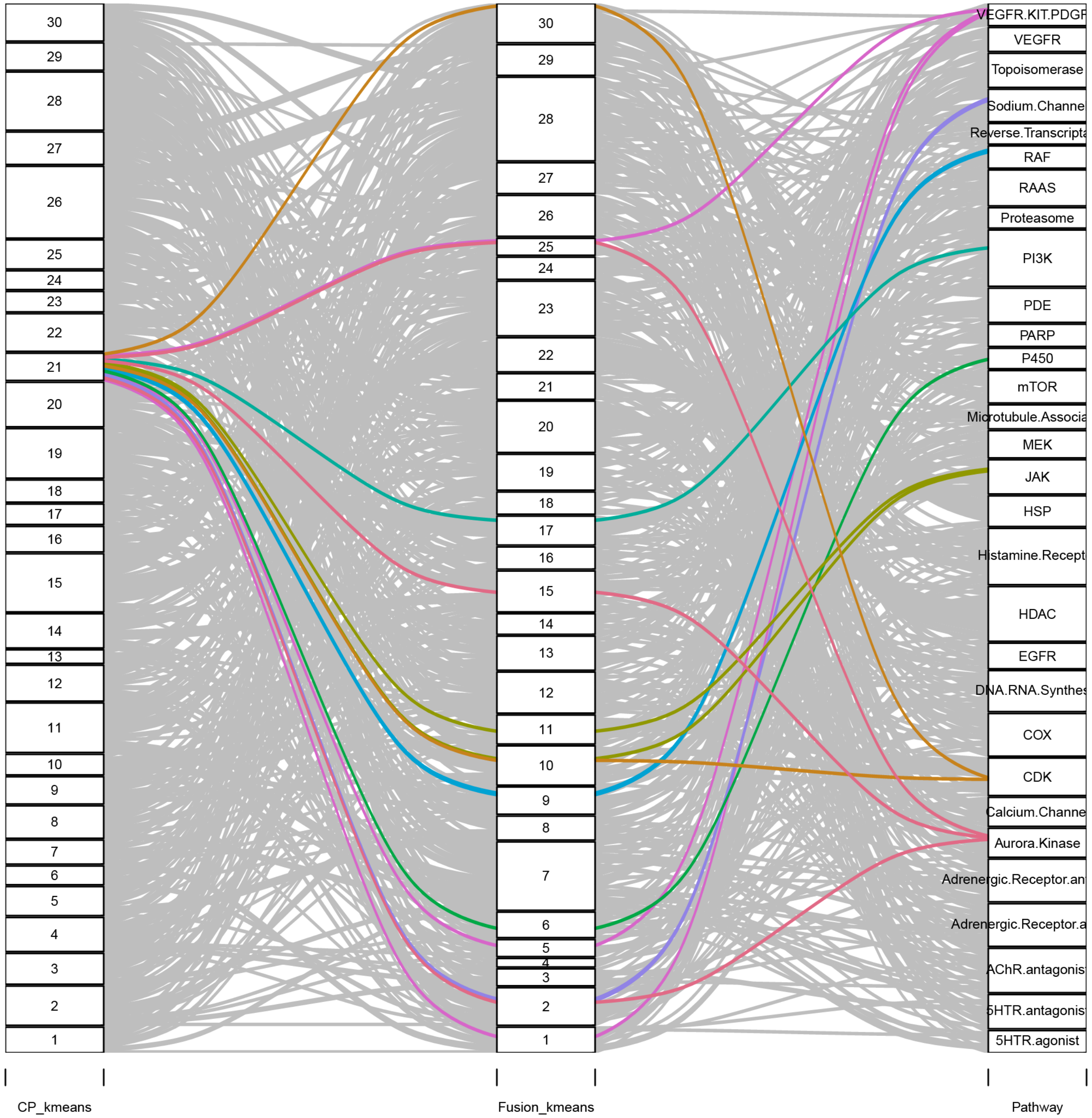
30
29
28
27
26
25
24
23
22
21
20
19
18
17
16
15
14
13
12
11
10
9
8
7
6
5
4
3
2
1
VEGFR.KIT.PDGF
VEGFR
Topoisomerase
Sodium.Channel
Reverse.Transcripta
RAF
RAAS
Proteasome
PI3K
PDE
PARP
P450
mTOR
Microtubule.Associa
MEK
JAK
HSP
Histamine.Recept
HDAC
EGFR
DNA.RNA.Synthes
COX
CDK
Calcium.Channe
Aurora.Kinase
Adrenergic.Receptor.an
Adrenergic.Receptor.a
AChR.antagonis
5HTR.antagonis
5HTR.agonist
CP_kmeans
Fusion_kmeans
Pathway

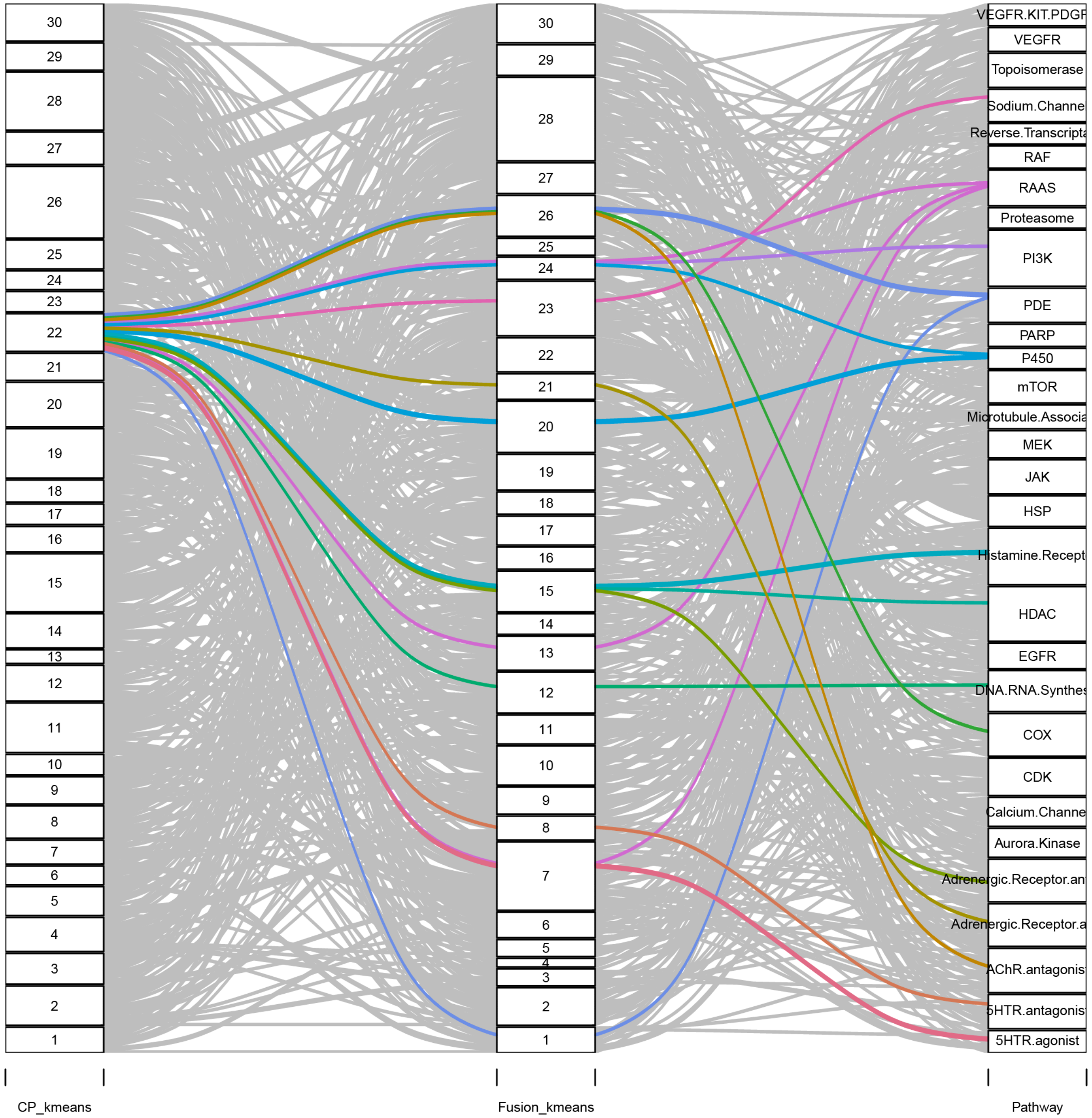
30
29
28
27
26
25
24
23
22
21
20
19
18
17
16
15
14
13
12
11
10
9
8
7
6
5
4
3
2
1
VEGFR.KIT.PDGF
VEGFR
Topoisomerase
Sodium.Channel
Reverse.Transcripta
RAF
RAAS
Proteasome
PI3K
PDE
PARP
P450
mTOR
Microtubule.Associa
MEK
JAK
HSP
Histamine.Recept
HDAC
EGFR
DNA.RNA.Synthes
COX
CDK
Calcium.Channe
Aurora.Kinase
Adrenergic.Receptor.an
Adrenergic.Receptor.a
AChR.antagonis
5HTR.antagonis
5HTR.agonist
CP_kmeans
Fusion_kmeans
Pathway

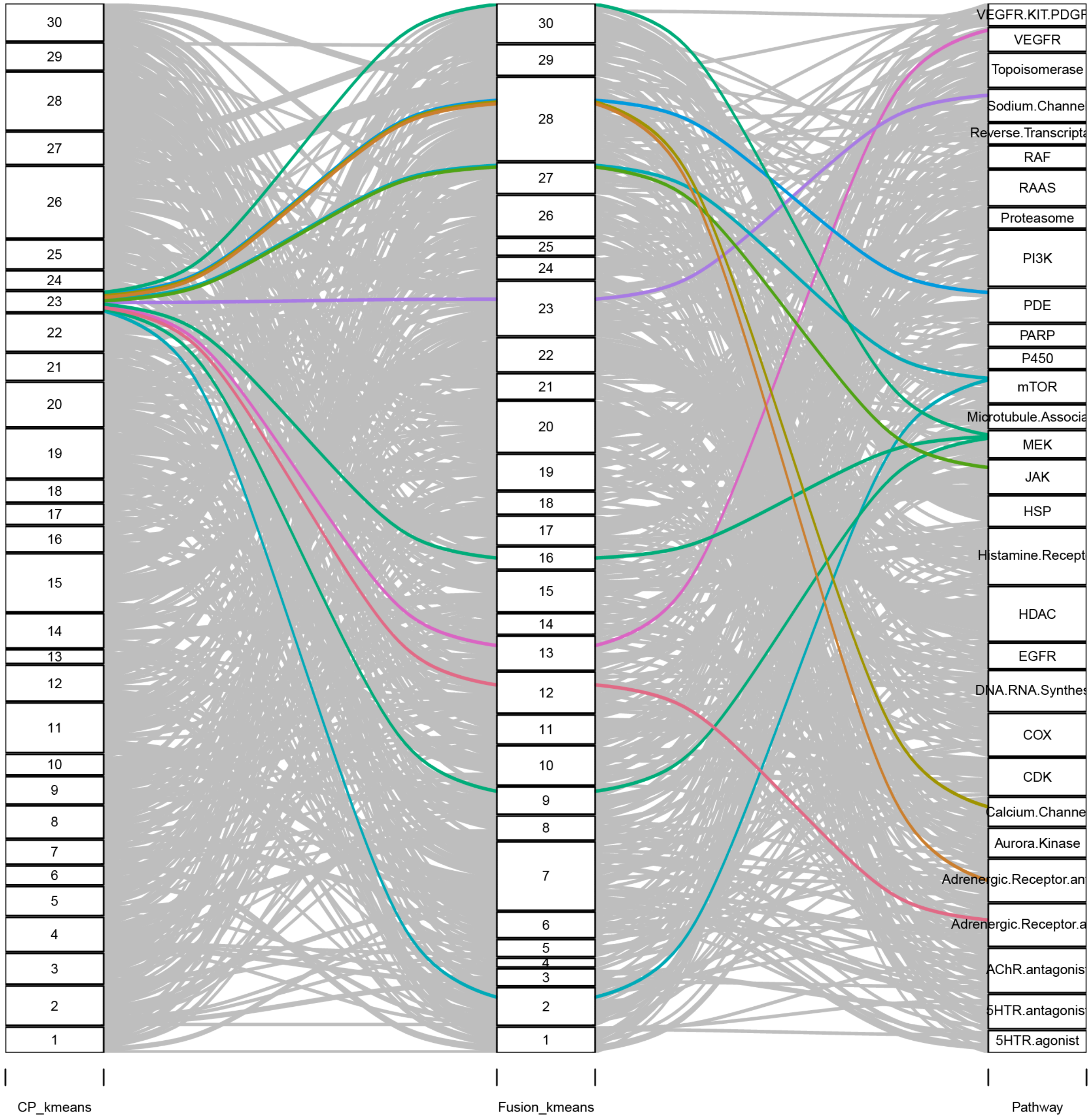

CP_kmeans
Fusion_kmeans
Pathway
VEGFR.KIT.PDGF
VEGFR
Topoisomerase
Sodium.Channel
Reverse.Transcripta
RAF
RAAS
Proteasome
PI3K
PDE
PARP
P450
mTOR
Microtubule.Associa
MEK
JAK
HSP
Histamine.Recept
HDAC
EGFR
DNA.RNA.Synthes
COX
CDK
Calcium.Channe
Aurora.Kinase
Adrenergic.Receptor.an
Adrenergic.Receptor.a
AChR.antagonis
5HTR.antagonis
5HTR.agonist

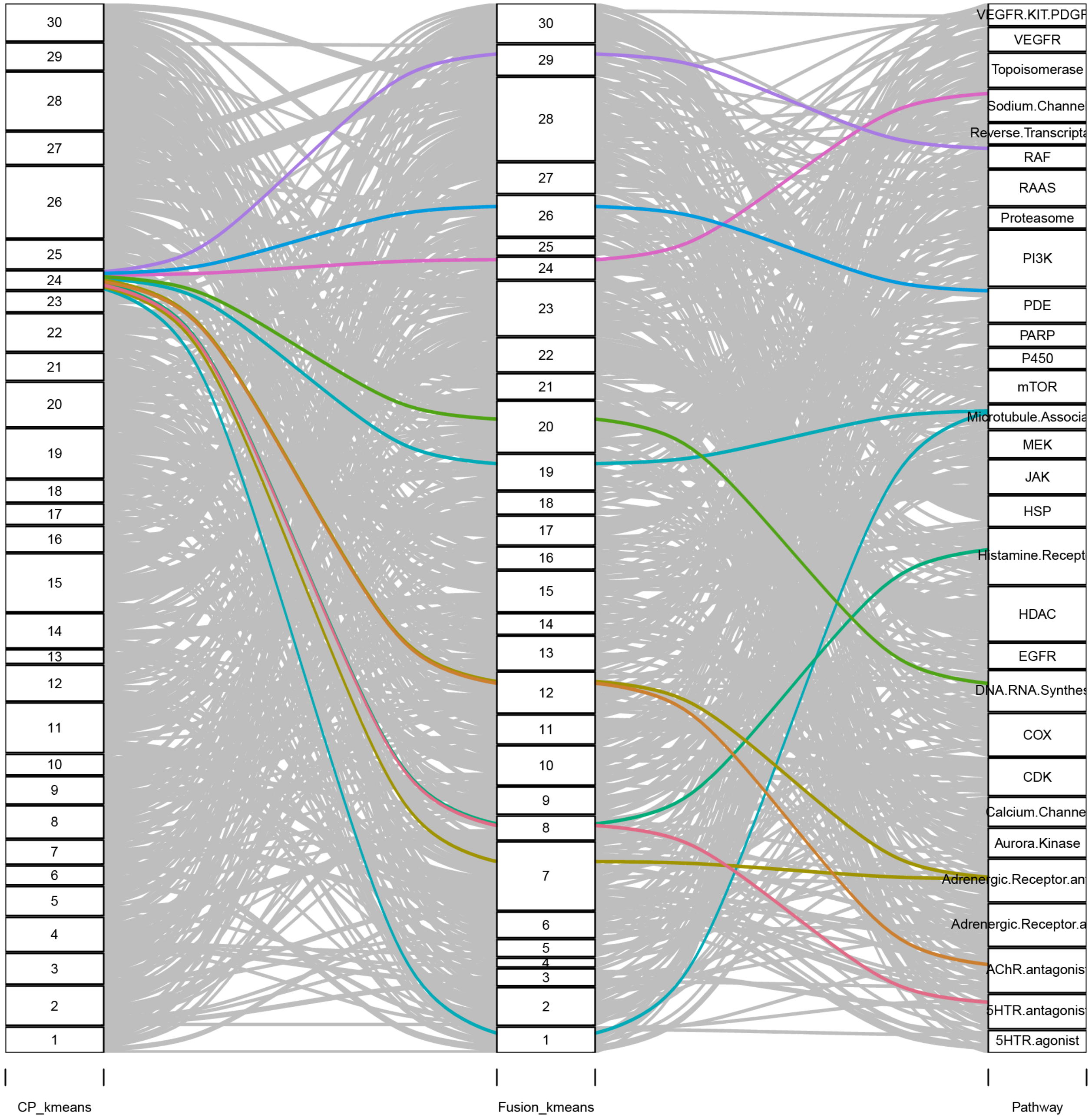

30
29
28
27
26
25
24
23
22
21
20
19
18
17
16
15
14
13
12
11
10
9
8
7
6
5
4
3
2
1
VEGFR.KIT.PDGF
VEGFR
Topoisomerase
Sodium.Channel
Reverse.Transcripta
RAF
RAAS
Proteasome
PI3K
PDE
PARP
P450
mTOR
Microtubule.Associa
MEK
JAK
HSP
Histamine.Recept
HDAC
EGFR
DNA.RNA.Synthes
COX
CDK
Calcium.Channe
Aurora.Kinase
Adrenergic.Receptor.an
Adrenergic.Receptor.a
AChR.antagonis
5HTR.antagonis
5HTR.agonist
CP_kmeans
Fusion_kmeans
Pathway

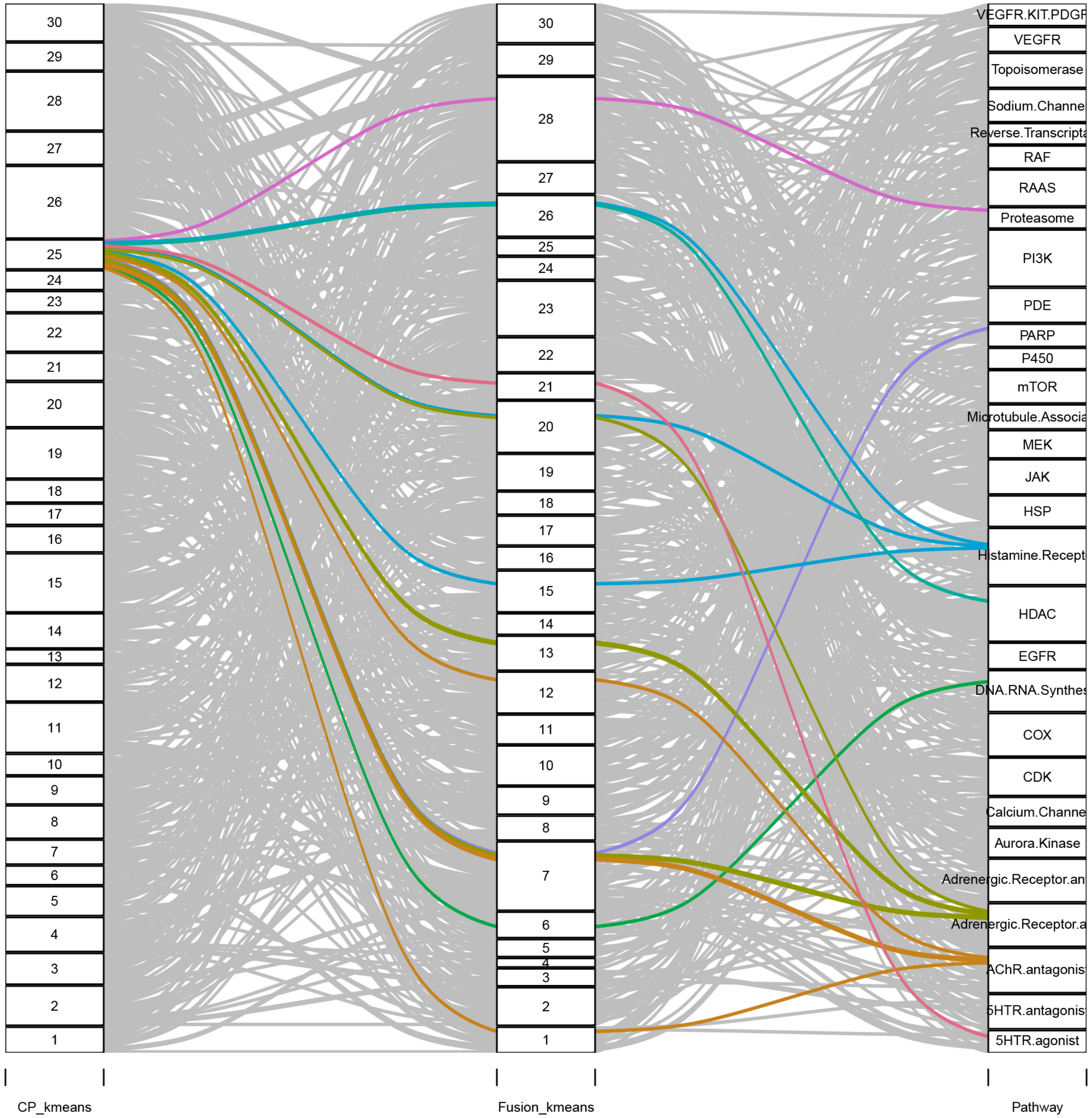
CP_kmeans
Fusion_kmeans
Pathway
30
29
28
27
26
25
24
23
22
21
20
19
18
17
16
15
14
13
12
11
10
9
8
7
6
5
4
3
2
1
VEGFR.KIT.PDGF
VEGFR
Topoisomerase
Sodium.Channel
Reverse.Transcripta
RAF
RAAS
Proteasome
PI3K
PDE
PARP
P450
mTOR
Microtubule.Associa
MEK
JAK
HSP
Histamine.Recept
HDAC
EGFR
DNA.RNA.Synthes
COX
CDK
Calcium.Channe
Aurora.Kinase
Adrenergic.Receptor.an
Adrenergic.Receptor.a
AChR.antagonis
5HTR.antagonis
5HTR.agonist

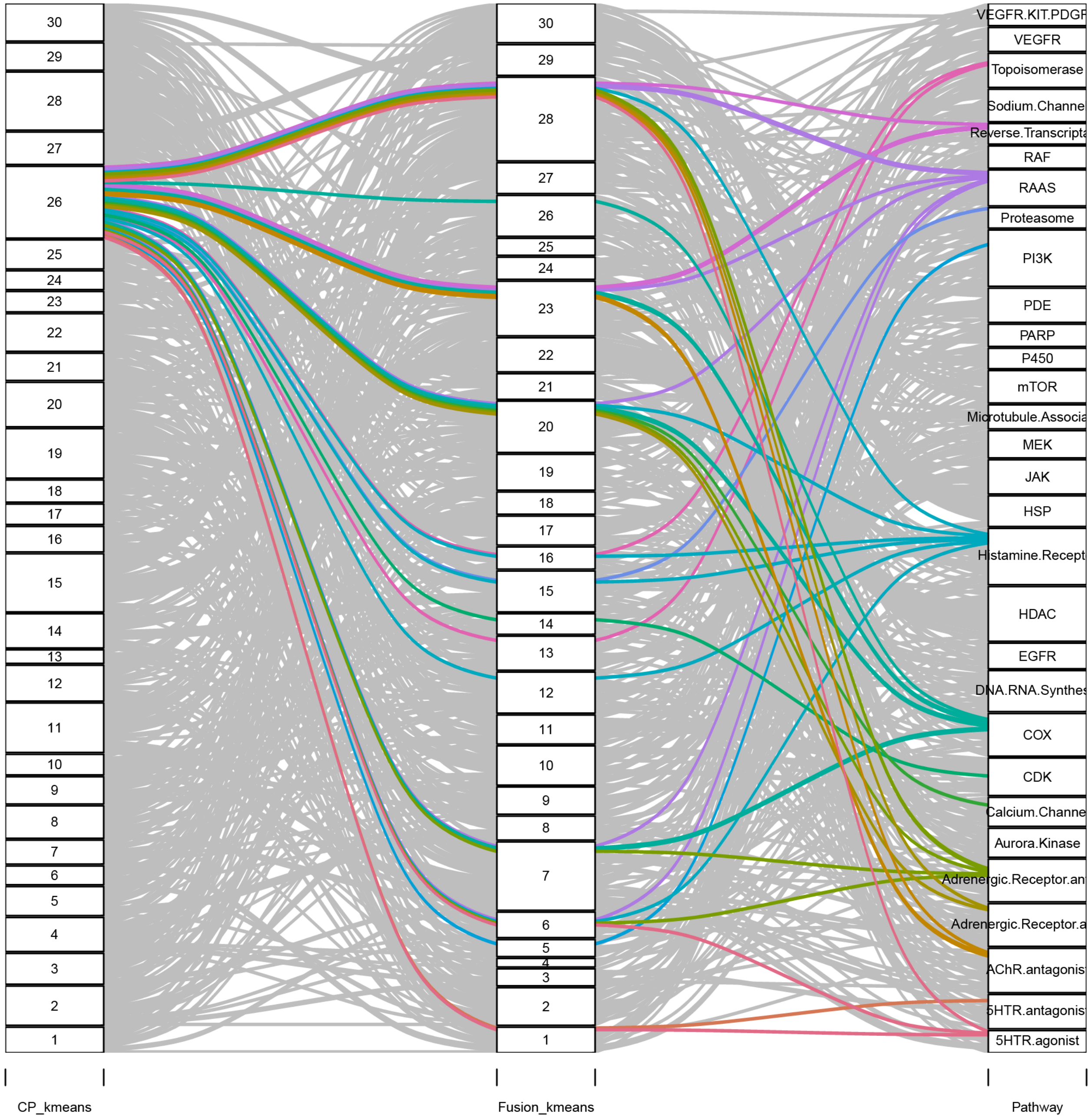
30
29
28
27
26
25
24
23
22
21
20
19
18
17
16
15
14
13
12
11
10
9
8
7
6
5
4
3
2
1
VEGFR.KIT.PDGF
VEGFR
Topoisomerase
Sodium.Channe
Reverse.Transcripta
RAF
RAAS
Proteasome
PI3K
PDE
PARP
P450
mTOR
Microtubule.Associa
MEK
JAK
HSP
Histamine.Recept
HDAC
EGFR
DNA.RNA.Synthes
COX
CDK
Calcium.Channe
Aurora.Kinase
Adrenergic.Receptor.an
Adrenergic.Receptor.a
AChR.antagonis
5HTR.antagonis
5HTR.agonist
CP_kmeans
Fusion_kmeans
Pathway

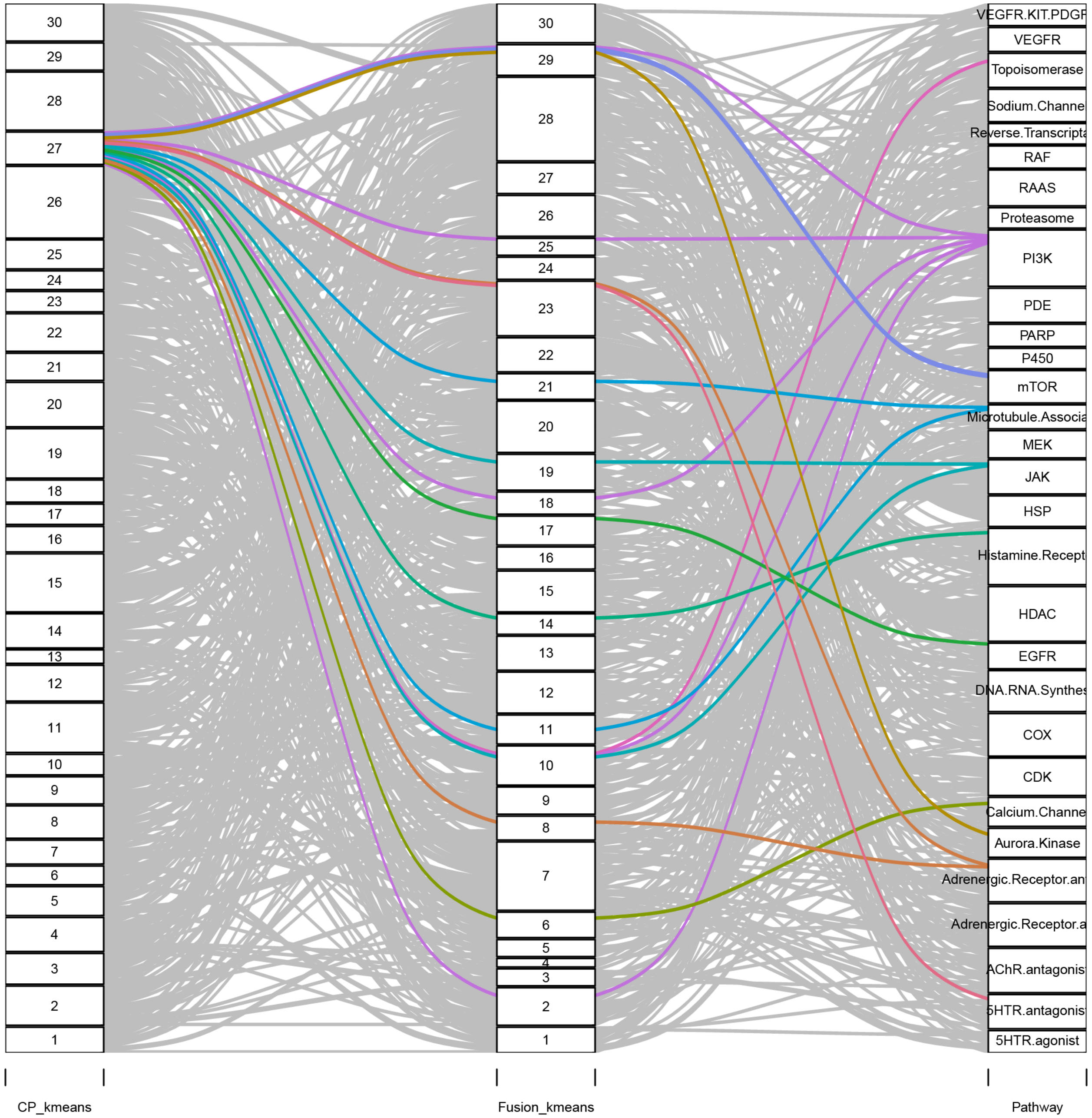

30
29
28
27
26
25
24
23
22
21
20
19
18
17
16
15
14
13
12
11
10
9
8
7
6
5
4
3
2
1
VEGFR.KIT.PDGF
VEGFR
Topoisomerase
Sodium.Channe
Reverse.Transcripta
RAF
RAAS
Proteasome
PI3K
PDE
PARP
P450
mTOR
Microtubule.Associa
MEK
JAK
HSP
Histamine.Recept
HDAC
EGFR
DNA.RNA.Synthes
COX
CDK
Calcium.Channe
Aurora.Kinase
Adrenergic.Receptor.an
Adrenergic.Receptor.a
AChR.antagonis
5HTR.antagonis
5HTR.agonist
CP_kmeans
Fusion_kmeans
Pathway

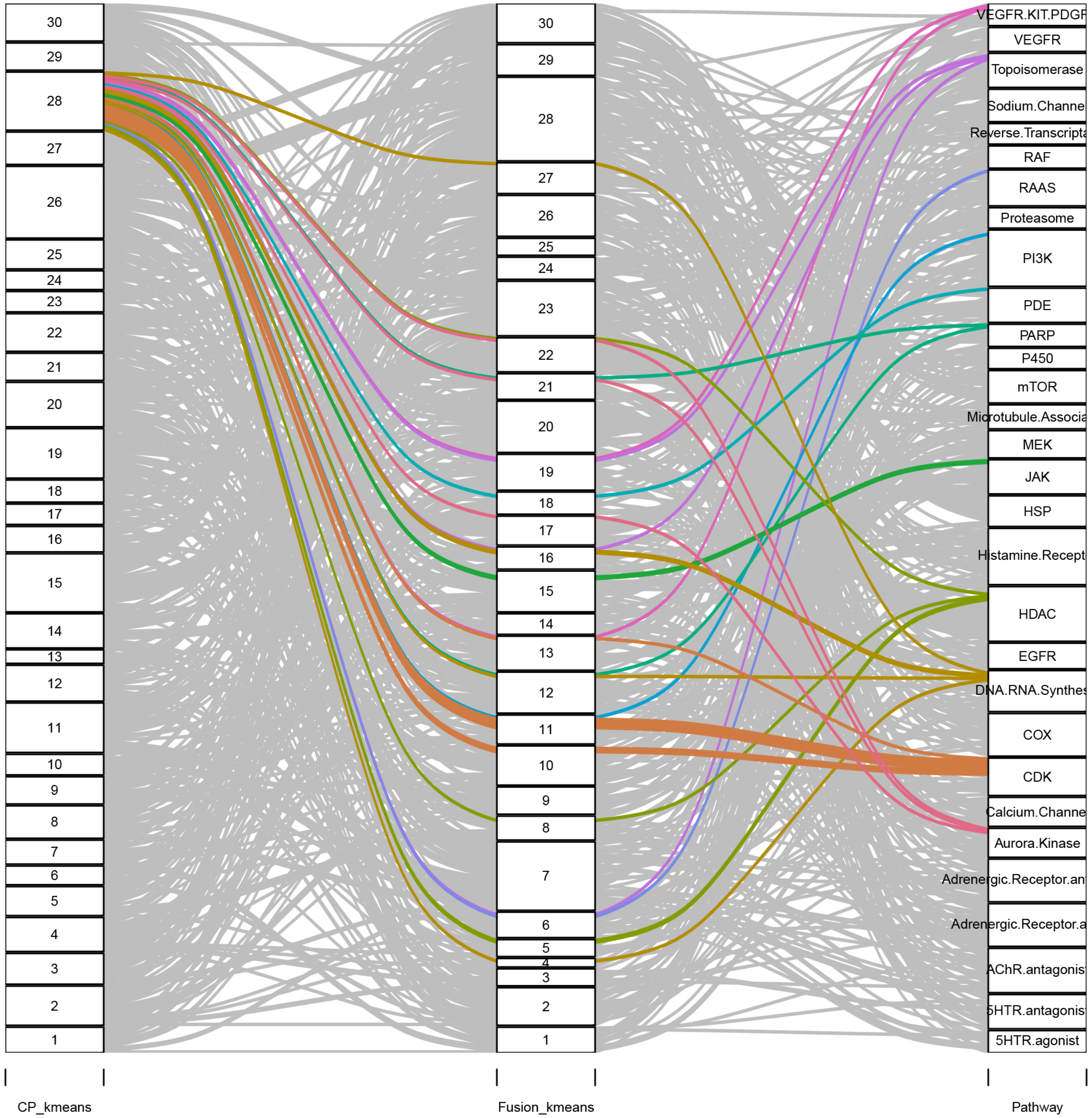
30
29
28
27
26
25
24
23
22
21
20
19
18
17
16
15
14
13
12
11
10
9
8
7
6
5
4
3
2
1
VEGFR.KIT.PDGF
VEGFR
Topoisomerase
Sodium.Channel
Reverse.Transcripta
RAF
RAAS
Proteasome
PI3K
PDE
PARP
P450
mTOR
Microtubule.Associa
MEK
JAK
HSP
Histamine.Recept
HDAC
EGFR
DNA.RNA.Synthes
COX
CDK
Calcium.Channe
Aurora.Kinase
Adrenergic.Receptor.an
Adrenergic.Receptor.a
AChR.antagonis
5HTR.antagonis
5HTR.agonist
CP_kmeans
Fusion_kmeans
Pathway

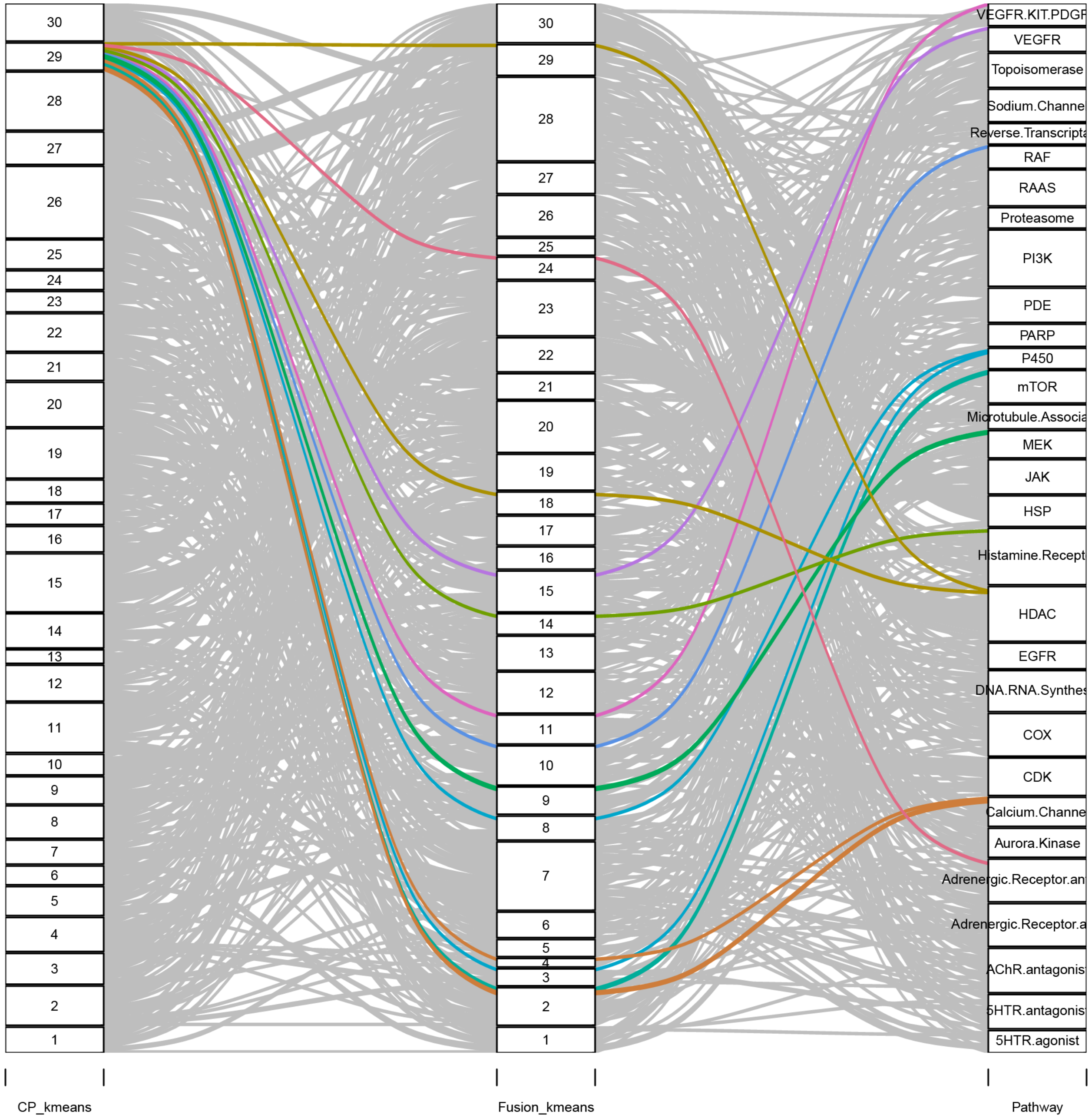

30
29
28
27
26
25
24
23
22
21
20
19
18
17
16
15
14
13
12
11
10
9
8
7
6
5
4
3
2
1
VEGFR.KIT.PDGF
VEGFR
Topoisomerase
Sodium.Channe
Reverse.Transcripta
RAF
RAAS
Proteasome
PI3K
PDE
PARP
P450
mTOR
Microtubule.Associa
MEK
JAK
HSP
Histamine.Recept
HDAC
EGFR
DNA.RNA.Synthes
COX
CDK
Calcium.Channe
Aurora.Kinase
Adrenergic.Receptor.an
Adrenergic.Receptor.a
AChR.antagonis
5HTR.antagonis
5HTR.agonist
CP_kmeans
Fusion_kmeans
Pathway

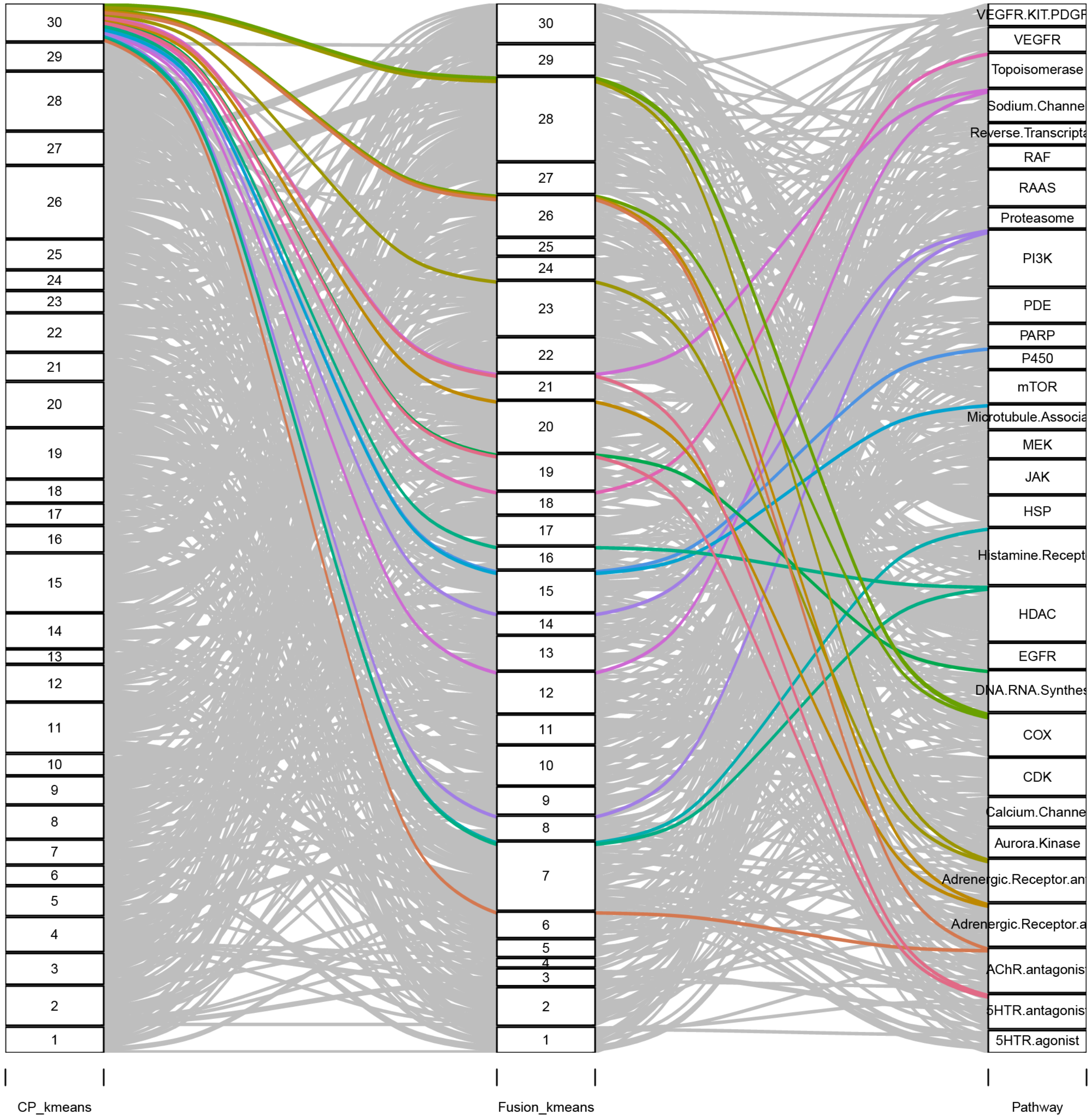
CP_kmeans
Fusion_kmeans
Pathway
30
29
28
27
26
25
24
23
22
21
20
19
18
17
16
15
14
13
12
11
10
9
8
7
6
5
4
3
2
1
VEGFR.KIT.PDGF
VEGFR
Topoisomerase
Sodium.Channel
Reverse.Transcripta
RAF
RAAS
Proteasome
PI3K
PDE
PARP
P450
mTOR
Microtubule.Associa
MEK
JAK
HSP
Histamine.Recept
HDAC
EGFR
DNA.RNA.Synthes
COX
CDK
Calcium.Channe
Aurora.Kinase
Adrenergic.Receptor.an
Adrenergic.Receptor.a
AChR.antagonis
5HTR.antagonis
5HTR.agonist

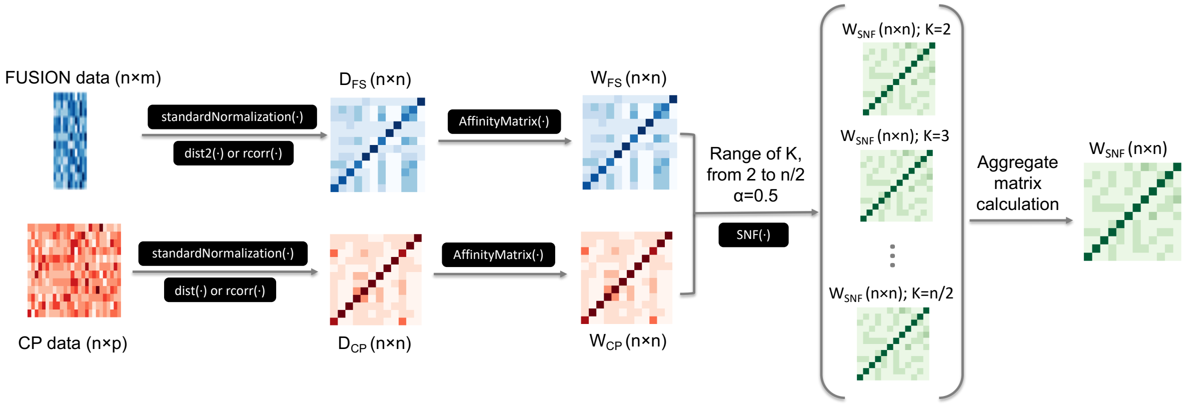

Supplementary Figure 7: Adapted similarity network fusion workflow.
All functions are from the SNFtool R package unless otherwise indicated. Normalized FUSION expression signatures and CP fingerprints are subjected to Z-score transformation by the standardNormalization function, followed by similarity matrix calculation using either the dist2 function for Euclidean Distance, or distanceMatrix function (ClassDiscovery package) for Pearson distance. Affinity matrices were then calculated by the AffinityMatrix function. Similarity network fusion (K=number of neighbors, α=hyperparameter) is applied to W_{FS} and W_{CP} across a range of K values from 2 to n/2 to generate a set of $W_{SNF\text{-}K}$ matrices. An aggregate matrix is then calculated as described in Supplemental Note 4.

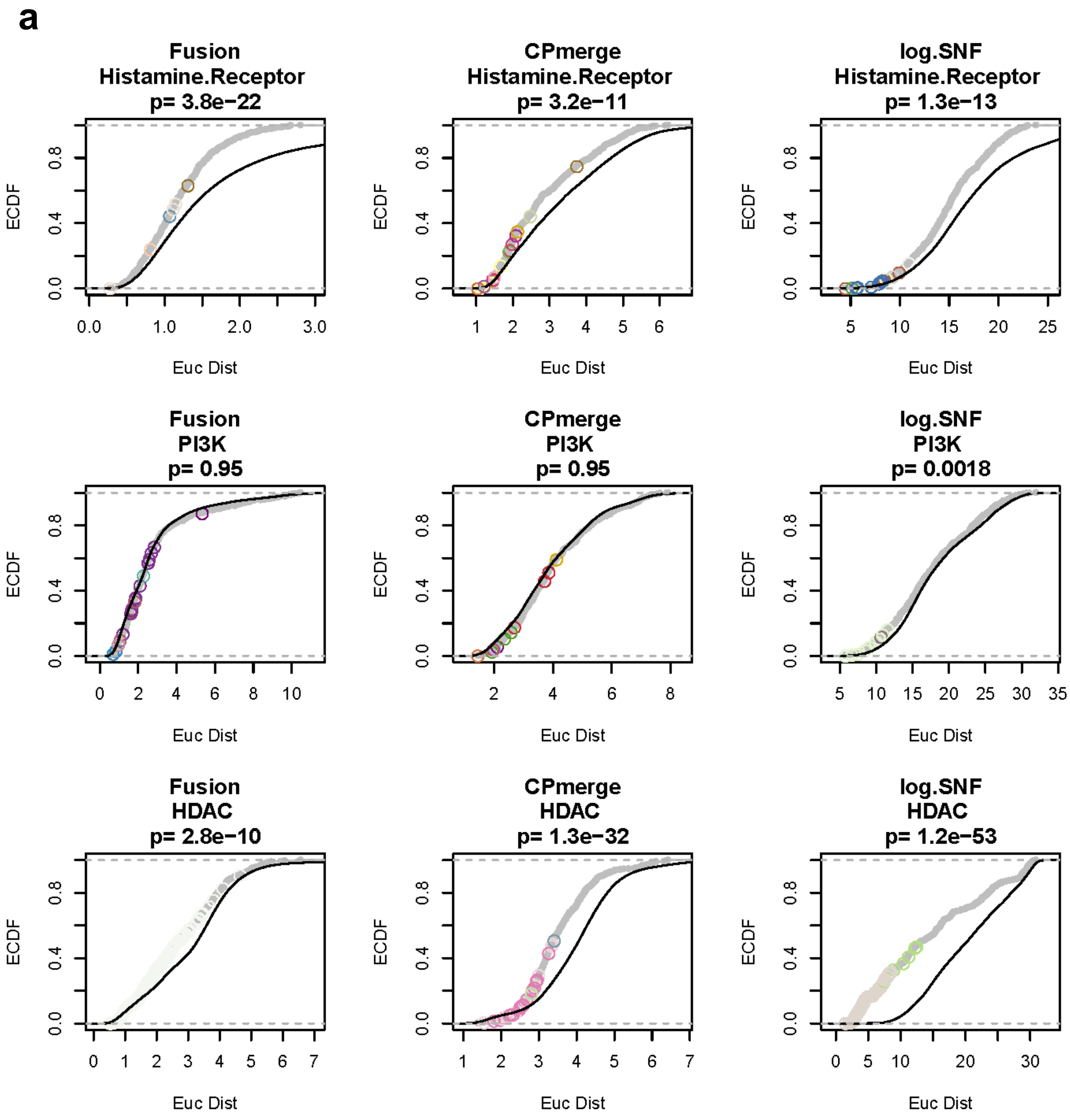

Supplementary Figure 8: CDF plots comparing pairwise associations between in-class and out-of-class associations. In-class-associations are colored in gray, with colored circles corresponding to cluster membership in the associated APC maps. Out-of-class associations are colored in black. KS-test p-values are shown above each plot. (**a**) CDF plots comparing pairwise Euclidean distances and (**b**) pairwise Pearson correlations.

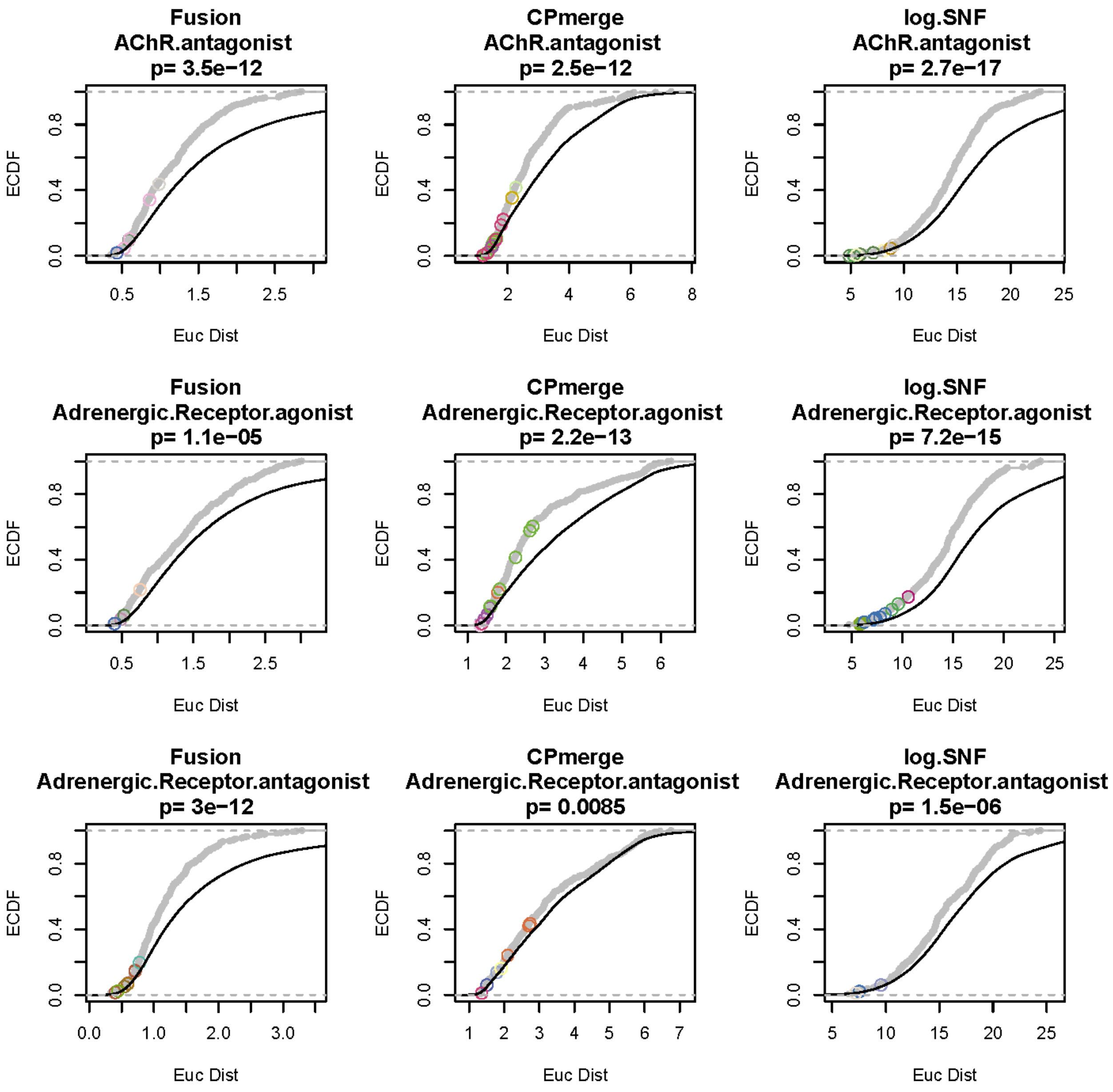
Fusion
AChR.antagonist
p= 3.5e−12
CPmerge
AChR.antagonist
p= 2.5e−12
log.SNF
AChR.antagonist
p= 2.7e−17
Fusion
Adrenergic.Receptor.agonist
p= 1.1e−05
CPmerge
Adrenergic.Receptor.agonist
p= 2.2e−13
log.SNF
Adrenergic.Receptor.agonist
p= 7.2e−15
Fusion
Adrenergic.Receptor.antagonist
p= 3e−12
CPmerge
Adrenergic.Receptor.antagonist
p= 0.0085
log.SNF
Adrenergic.Receptor.antagonist
p= 1.5e−06
ECDF
Euc Dist

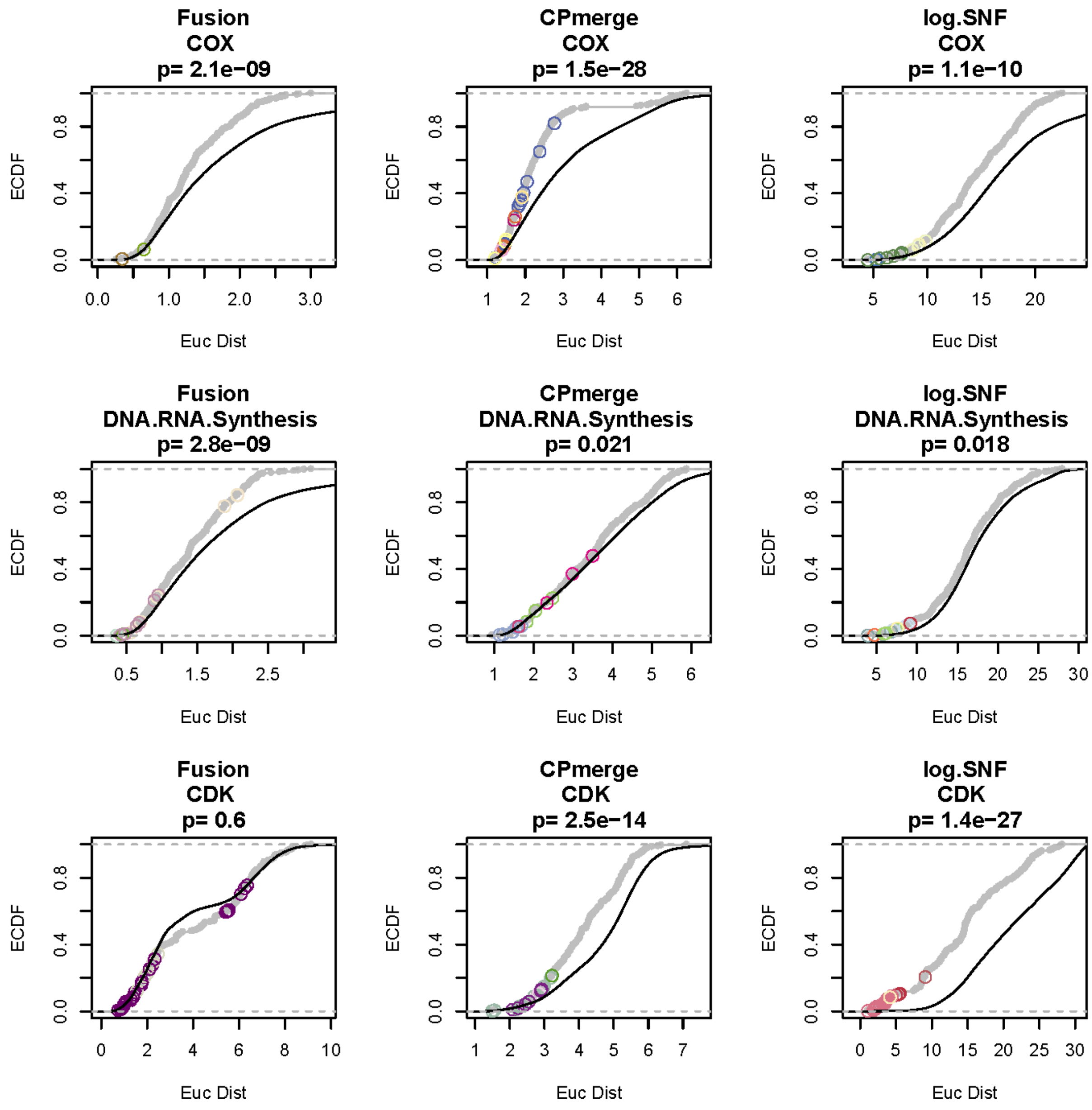
Fusion
COX
p= 2.1e−09
ECDF
0.0 0.4 0.8
0.0 1.0 2.0 3.0
Euc Dist
CPmerge
COX
p= 1.5e−28
ECDF
0.0 0.4 0.8
1 2 3 4 5 6
Euc Dist
log.SNF
COX
p= 1.1e−10
ECDF
0.0 0.4 0.8
5 10 15 20
Euc Dist
Fusion
DNA.RNA.Synthesis
p= 2.8e−09
ECDF
0.0 0.4 0.8
0.5 1.5 2.5
Euc Dist
CPmerge
DNA.RNA.Synthesis
p= 0.021
ECDF
0.0 0.4 0.8
1 2 3 4 5 6
Euc Dist
log.SNF
DNA.RNA.Synthesis
p= 0.018
ECDF
0.0 0.4 0.8
5 10 15 20 25 30
Euc Dist
Fusion
CDK
p= 0.6
ECDF
0.0 0.4 0.8
0 2 4 6 8 10
Euc Dist
CPmerge
CDK
p= 2.5e−14
ECDF
0.0 0.4 0.8
1 2 3 4 5 6 7
Euc Dist
log.SNF
CDK
p= 1.4e−27
ECDF
0.0 0.4 0.8
0 5 10 15 20 25 30
Euc Dist

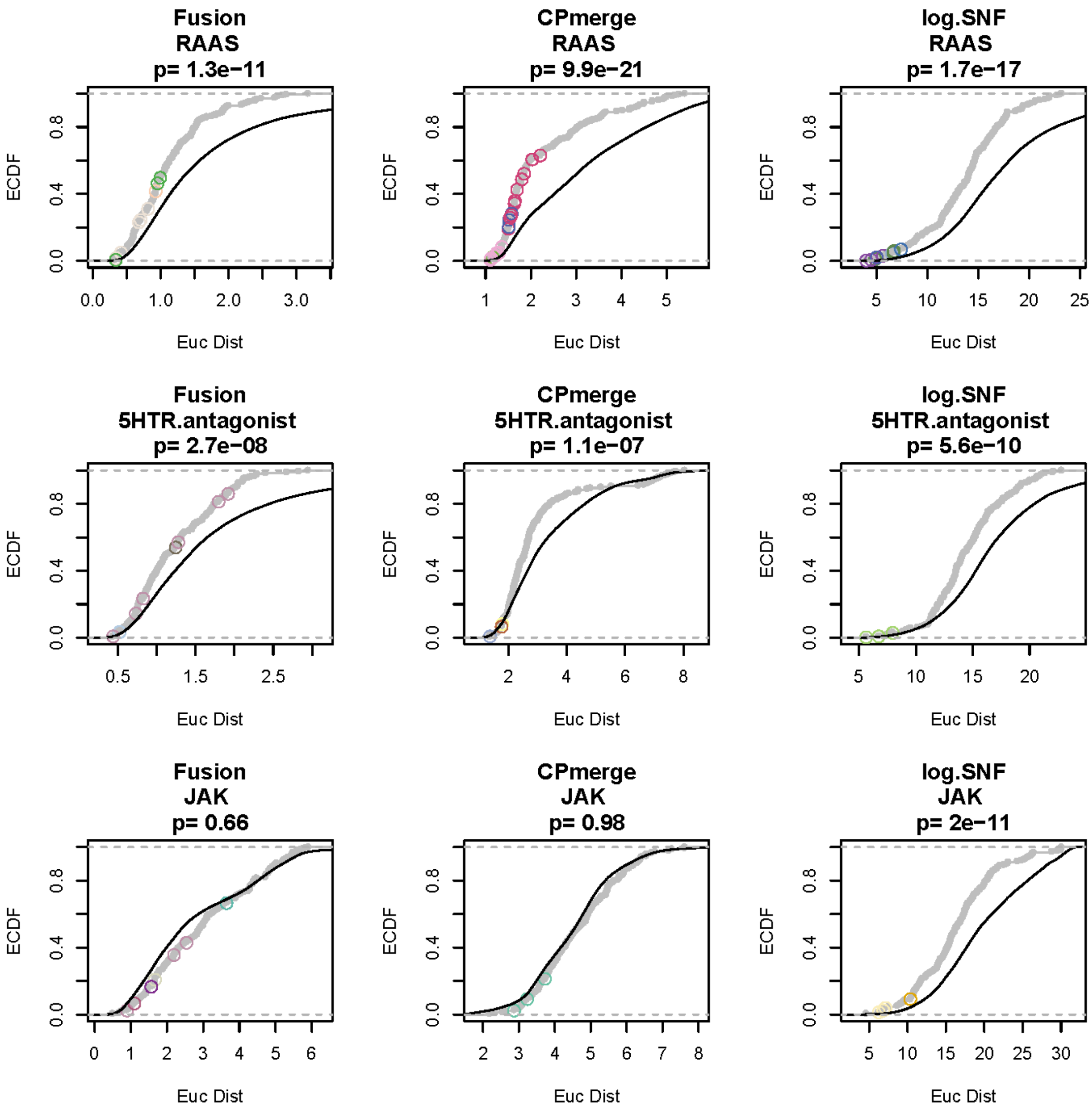
Fusion
RAAS
p= 1.3e−11
CPmerge
RAAS
p= 9.9e−21
log.SNF
RAAS
p= 1.7e−17
Fusion
5HTR.antagonist
p= 2.7e−08
CPmerge
5HTR.antagonist
p= 1.1e−07
log.SNF
5HTR.antagonist
p= 5.6e−10
Fusion
JAK
p= 0.66
CPmerge
JAK
p= 0.98
log.SNF
JAK
p= 2e−11
ECDF
Euc Dist

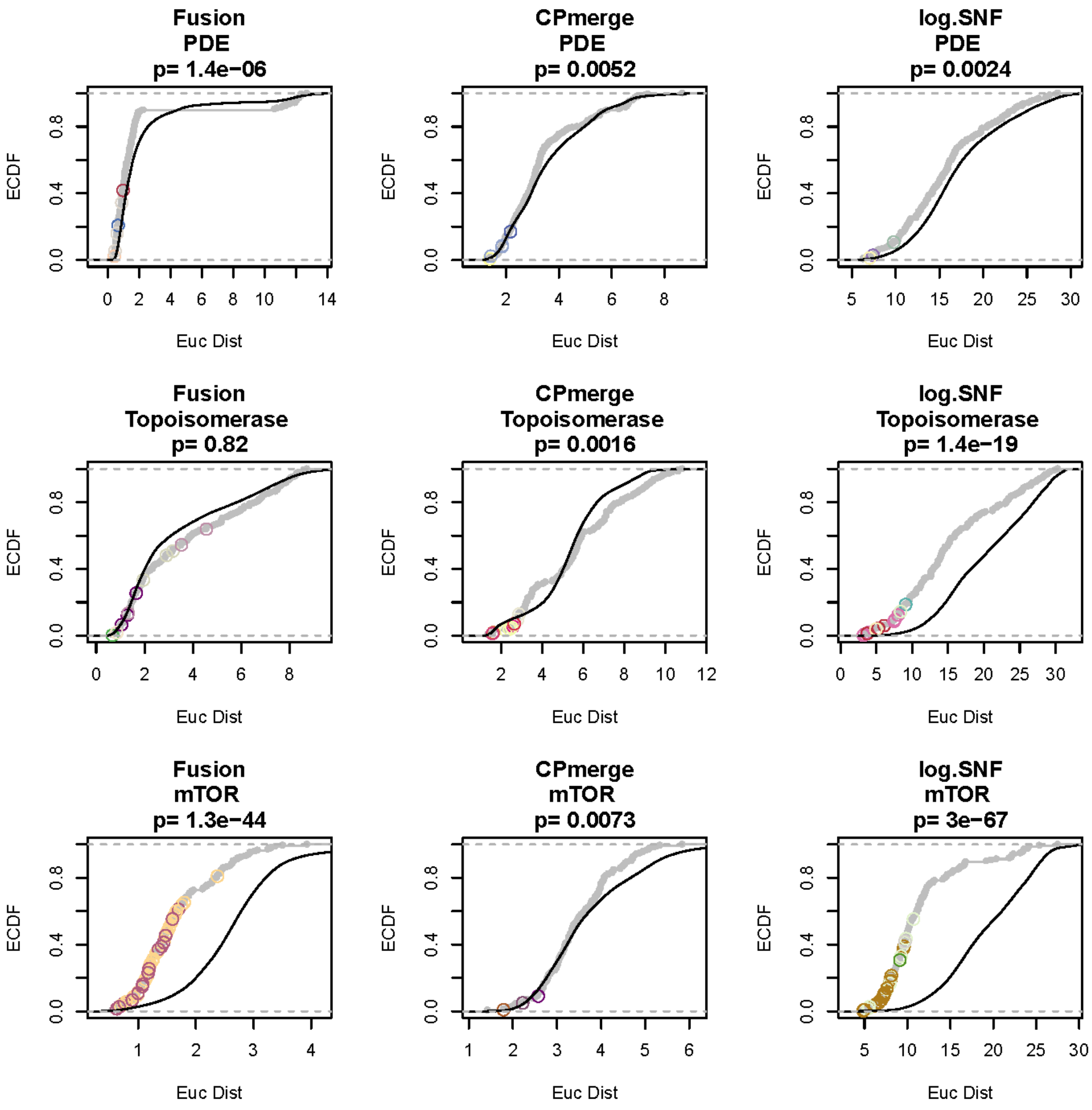
Fusion
PDE
p= 1.4e−06
CPmerge
PDE
p= 0.0052
log.SNF
PDE
p= 0.0024
Fusion
Topoisomerase
p= 0.82
CPmerge
Topoisomerase
p= 0.0016
log.SNF
Topoisomerase
p= 1.4e−19
Fusion
mTOR
p= 1.3e−44
CPmerge
mTOR
p= 0.0073
log.SNF
mTOR
p= 3e−67
ECDF
Euc Dist

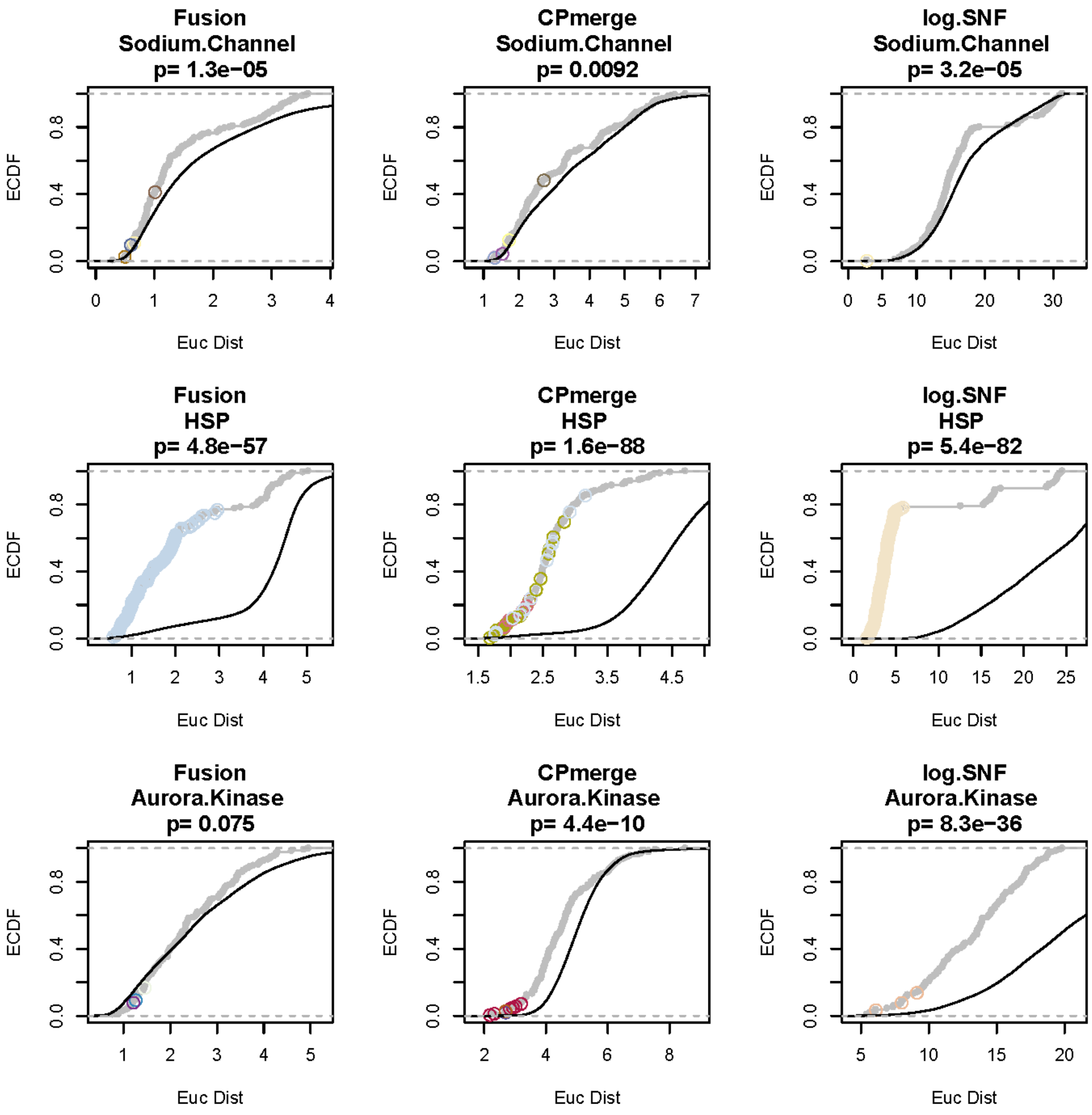

Fusion
Sodium.Channel
p= 1.3e−05
CPmerge
Sodium.Channel
p= 0.0092
log.SNF
Sodium.Channel
p= 3.2e−05
Fusion
HSP
p= 4.8e−57
CPmerge
HSP
p= 1.6e−88
log.SNF
HSP
p= 5.4e−82
Fusion
Aurora.Kinase
p= 0.075
CPmerge
Aurora.Kinase
p= 4.4e−10
log.SNF
Aurora.Kinase
p= 8.3e−36
ECDF
Euc Dist

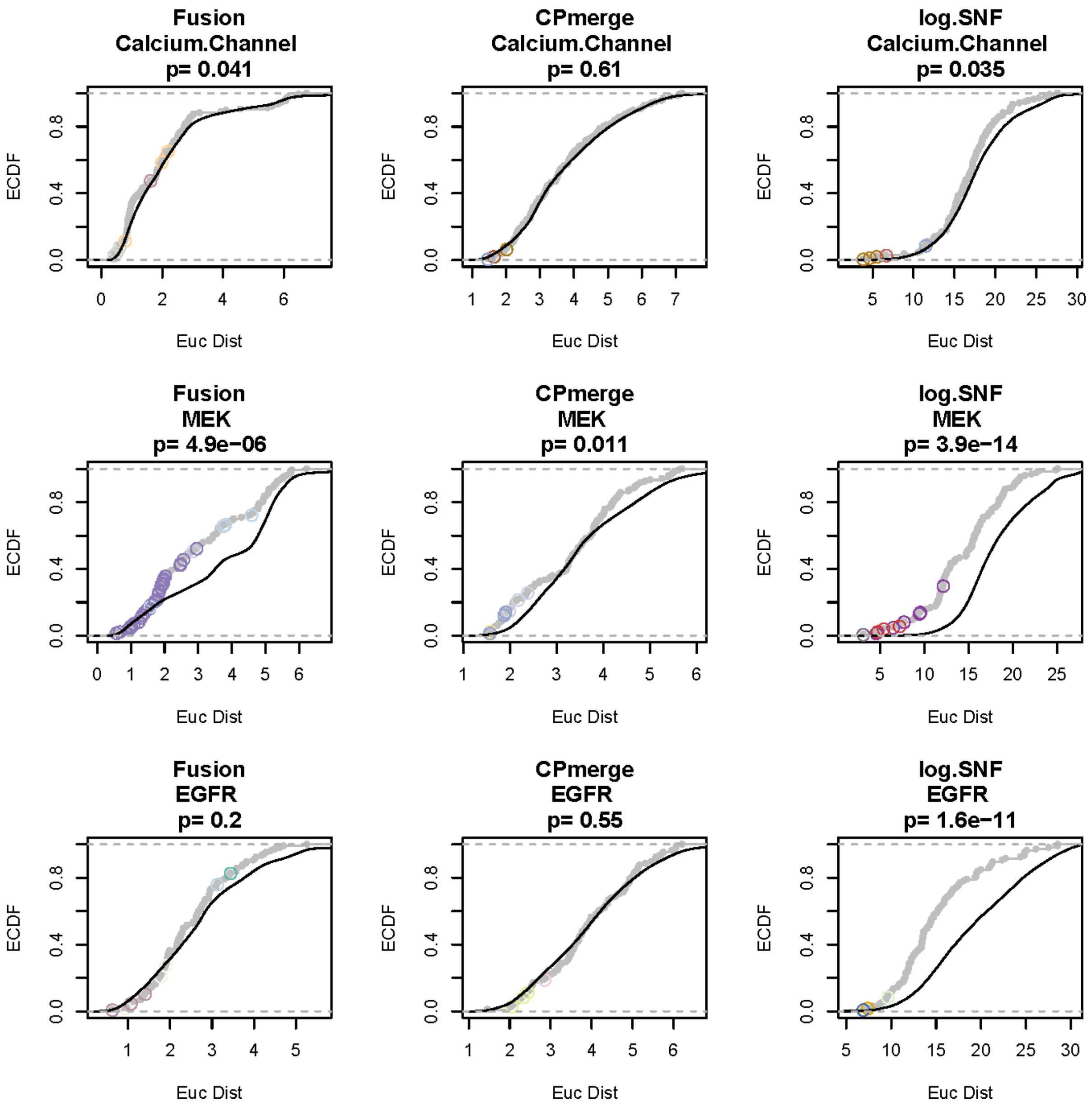
Fusion
Calcium.Channel
p= 0.041
CPmerge
Calcium.Channel
p= 0.61
log.SNF
Calcium.Channel
p= 0.035
Fusion
MEK
p= 4.9e−06
CPmerge
MEK
p= 0.011
log.SNF
MEK
p= 3.9e−14
Fusion
EGFR
p= 0.2
CPmerge
EGFR
p= 0.55
log.SNF
EGFR
p= 1.6e−11
ECDF
Euc Dist
0.0
0.4
0.8

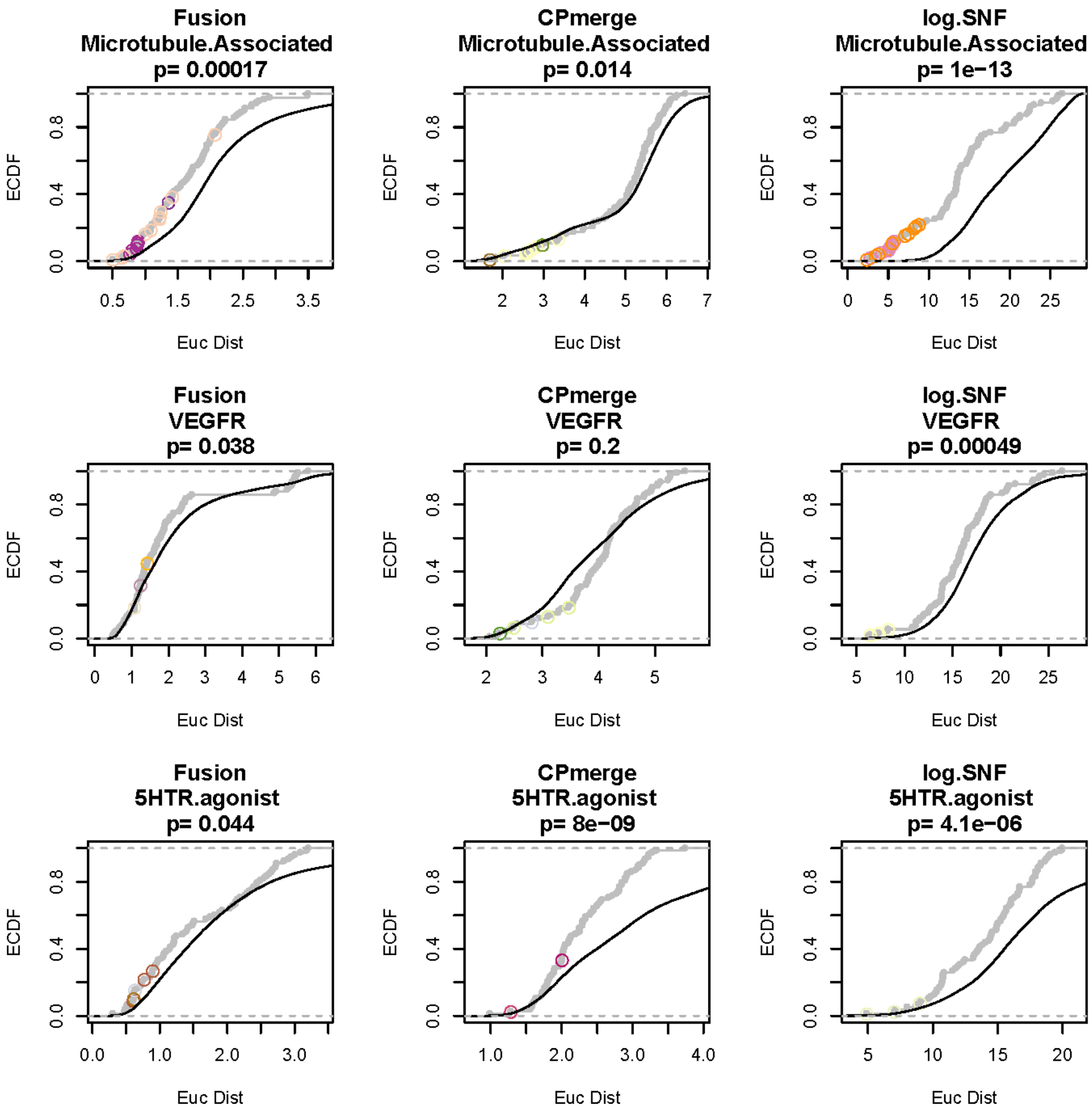
Fusion
Microtubule.Associated
p= 0.00017
CPmerge
Microtubule.Associated
p= 0.014
log.SNF
Microtubule.Associated
p= 1e−13
Fusion
VEGFR
p= 0.038
CPmerge
VEGFR
p= 0.2
log.SNF
VEGFR
p= 0.00049
Fusion
5HTR.agonist
p= 0.044
CPmerge
5HTR.agonist
p= 8e−09
log.SNF
5HTR.agonist
p= 4.1e−06
ECDF
Euc Dist

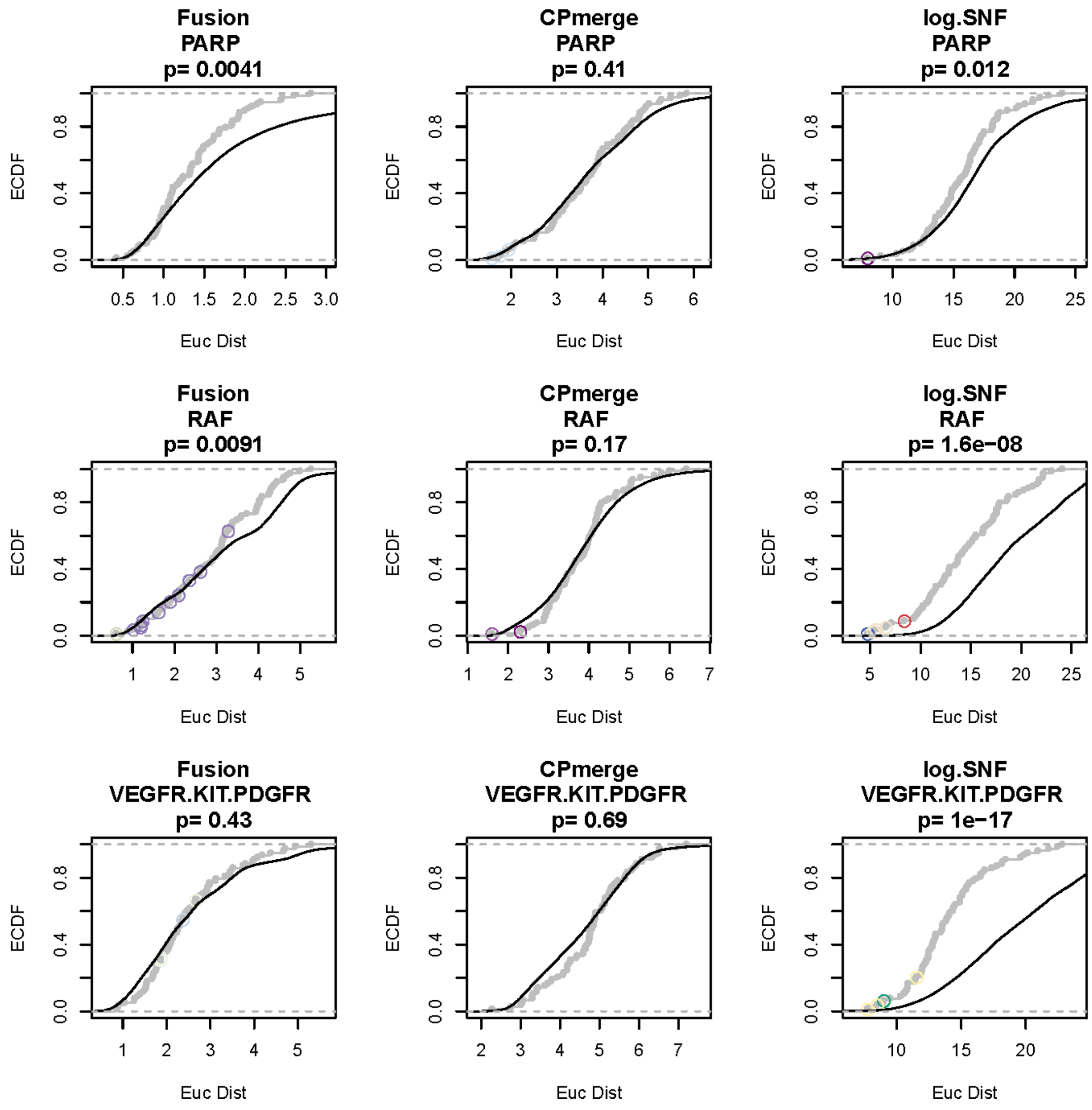
Fusion
PARP
p= 0.0041
CPmerge
PARP
p= 0.41
log.SNF
PARP
p= 0.012
Fusion
RAF
p= 0.0091
CPmerge
RAF
p= 0.17
log.SNF
RAF
p= 1.6e−08
Fusion
VEGFR.KIT.PDGFR
p= 0.43
CPmerge
VEGFR.KIT.PDGFR
p= 0.69
log.SNF
VEGFR.KIT.PDGFR
p= 1e−17
ECDF
Euc Dist

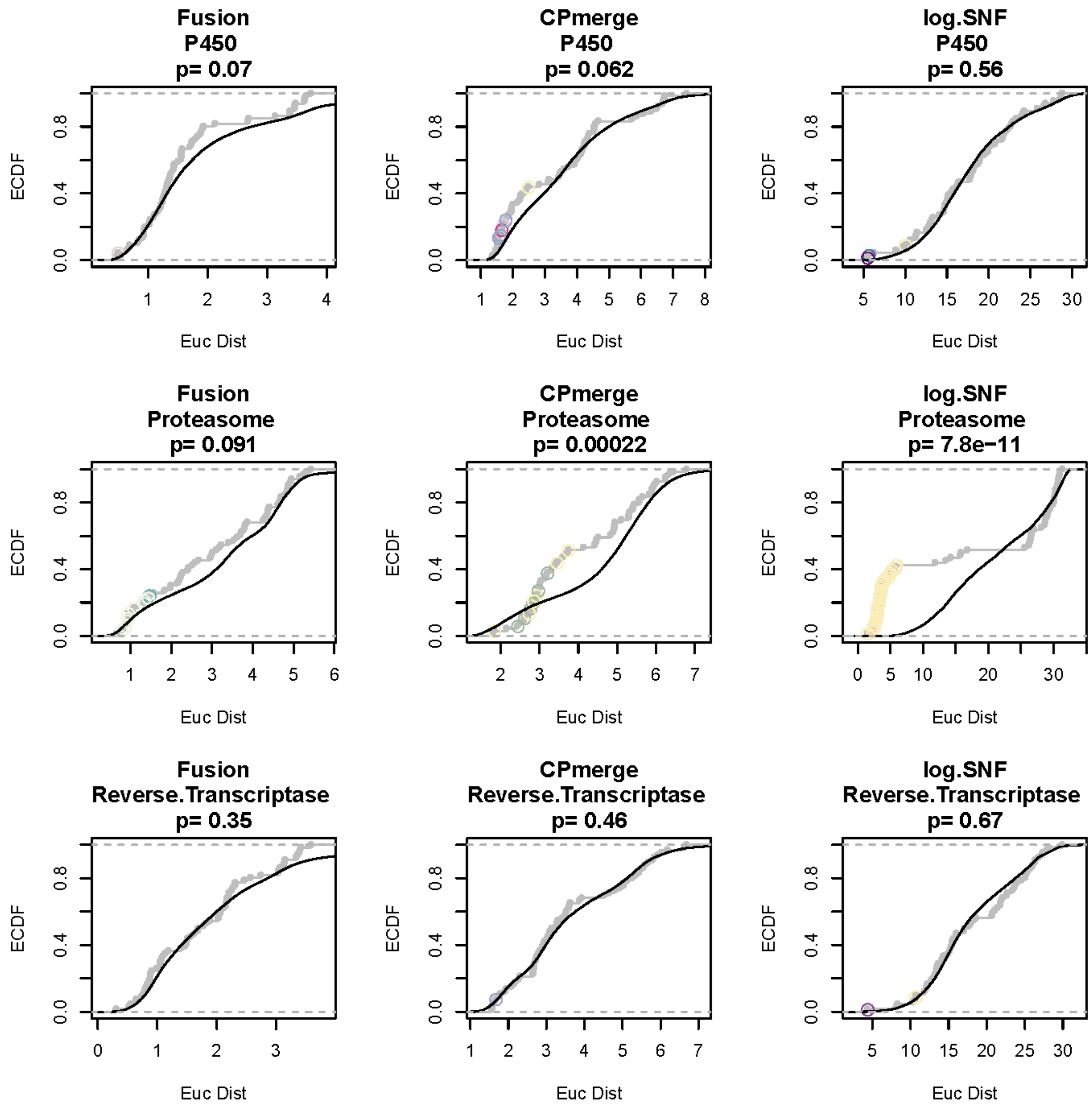
Fusion
P450
p= 0.07
CPmerge
P450
p= 0.062
log.SNF
P450
p= 0.56
Fusion
Proteasome
p= 0.091
CPmerge
Proteasome
p= 0.00022
log.SNF
Proteasome
p= 7.8e−11
Fusion
Reverse.Transcriptase
p= 0.35
CPmerge
Reverse.Transcriptase
p= 0.46
log.SNF
Reverse.Transcriptase
p= 0.67
ECDF
0.0
0.4
0.8
Euc Dist

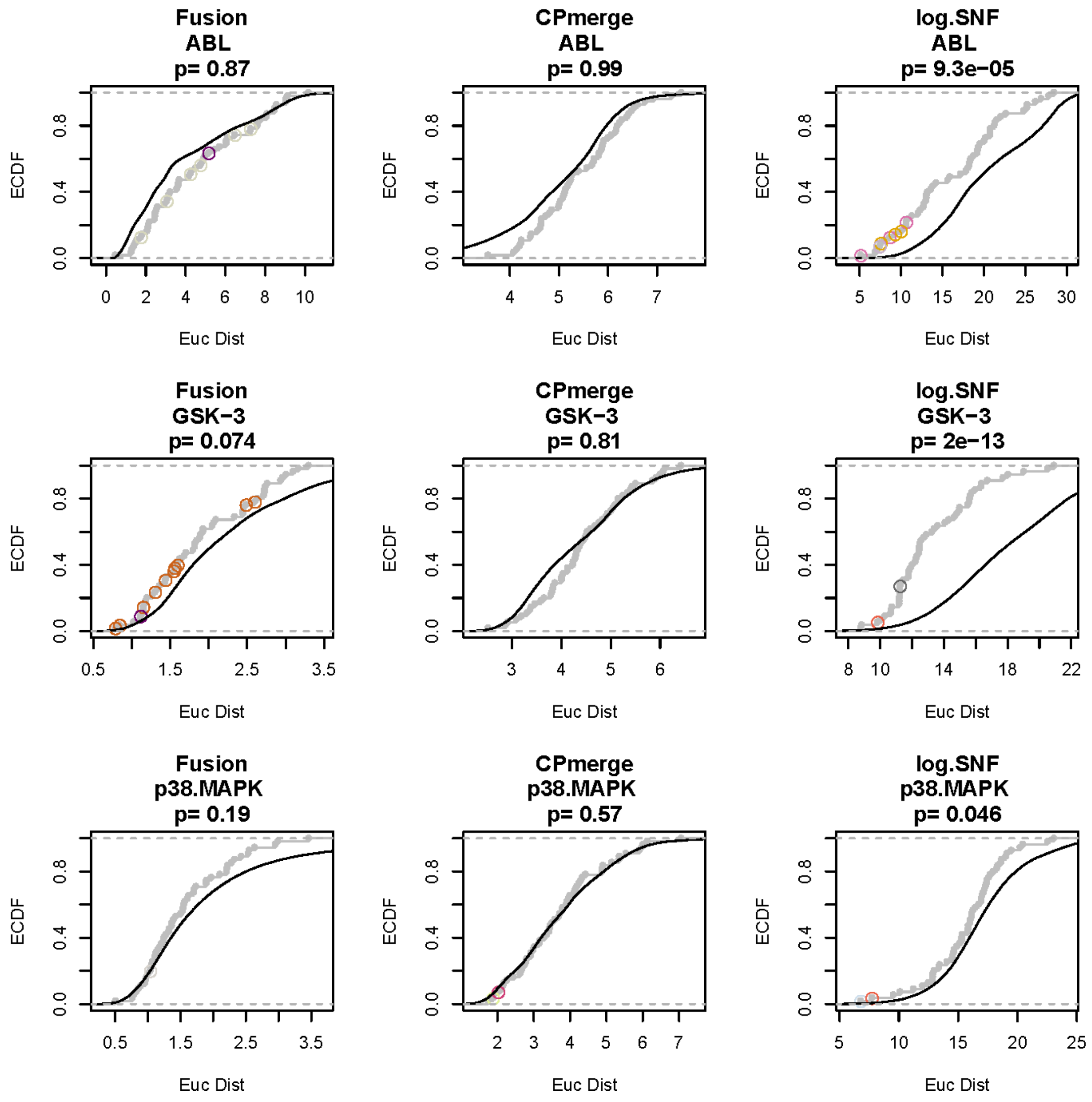
Fusion
ABL
p= 0.87
CPmerge
ABL
p= 0.99
log.SNF
ABL
p= 9.3e−05
Fusion
GSK−3
p= 0.074
CPmerge
GSK−3
p= 0.81
log.SNF
GSK−3
p= 2e−13
Fusion
p38.MAPK
p= 0.19
CPmerge
p38.MAPK
p= 0.57
log.SNF
p38.MAPK
p= 0.046
ECDF
Euc Dist

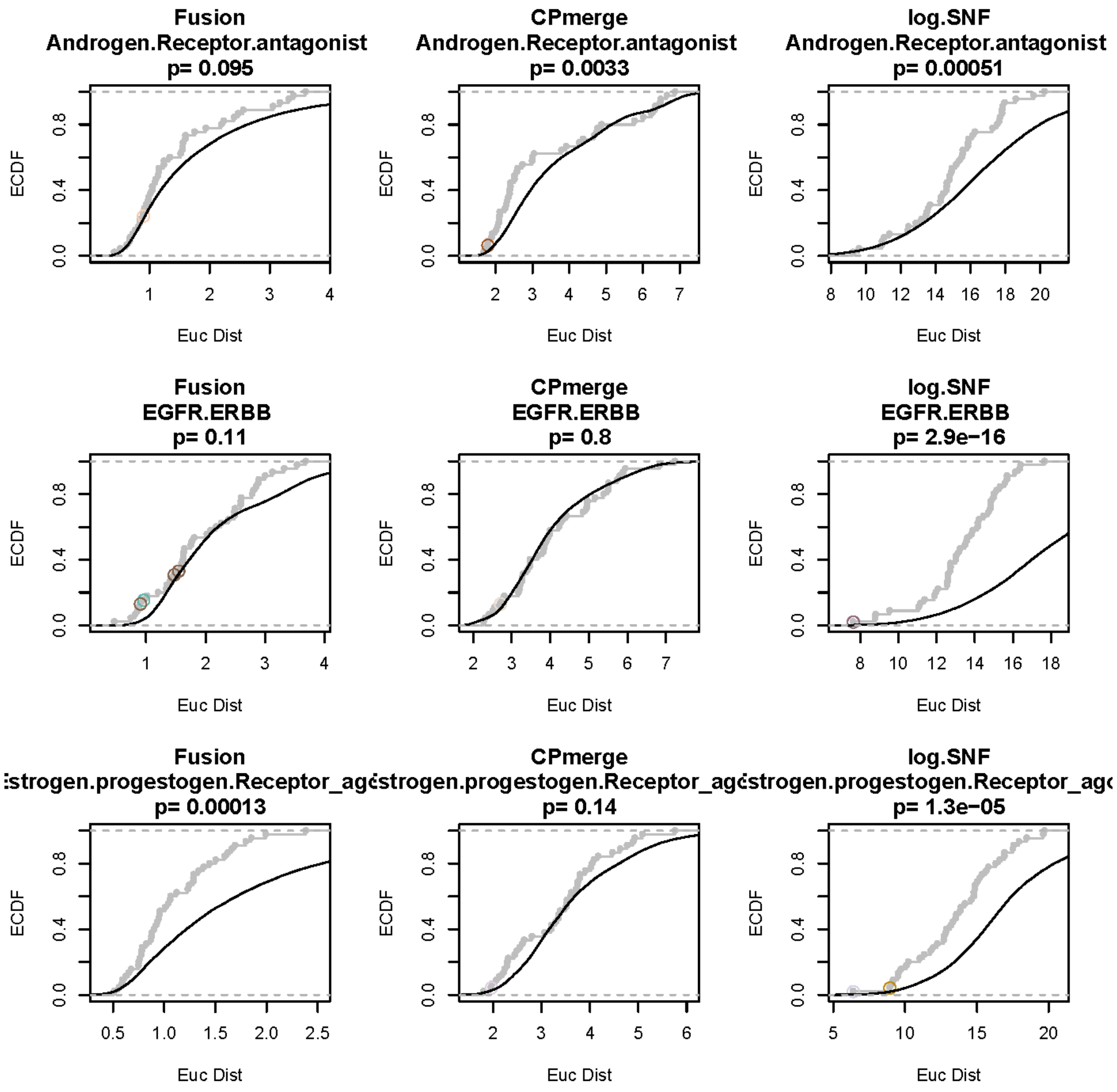
Fusion
Androgen.Receptor.antagonist
p= 0.095
CPmerge
Androgen.Receptor.antagonist
p= 0.0033
log.SNF
Androgen.Receptor.antagonist
p= 0.00051
Fusion
EGFR.ERBB
p= 0.11
CPmerge
EGFR.ERBB
p= 0.8
log.SNF
EGFR.ERBB
p= 2.9e−16
Fusion
:strogen.progestogen.Receptor_agc
p= 0.00013
CPmerge
:strogen.progestogen.Receptor_agc
p= 0.14
log.SNF
:strogen.progestogen.Receptor_agc
p= 1.3e−05
ECDF
Euc Dist

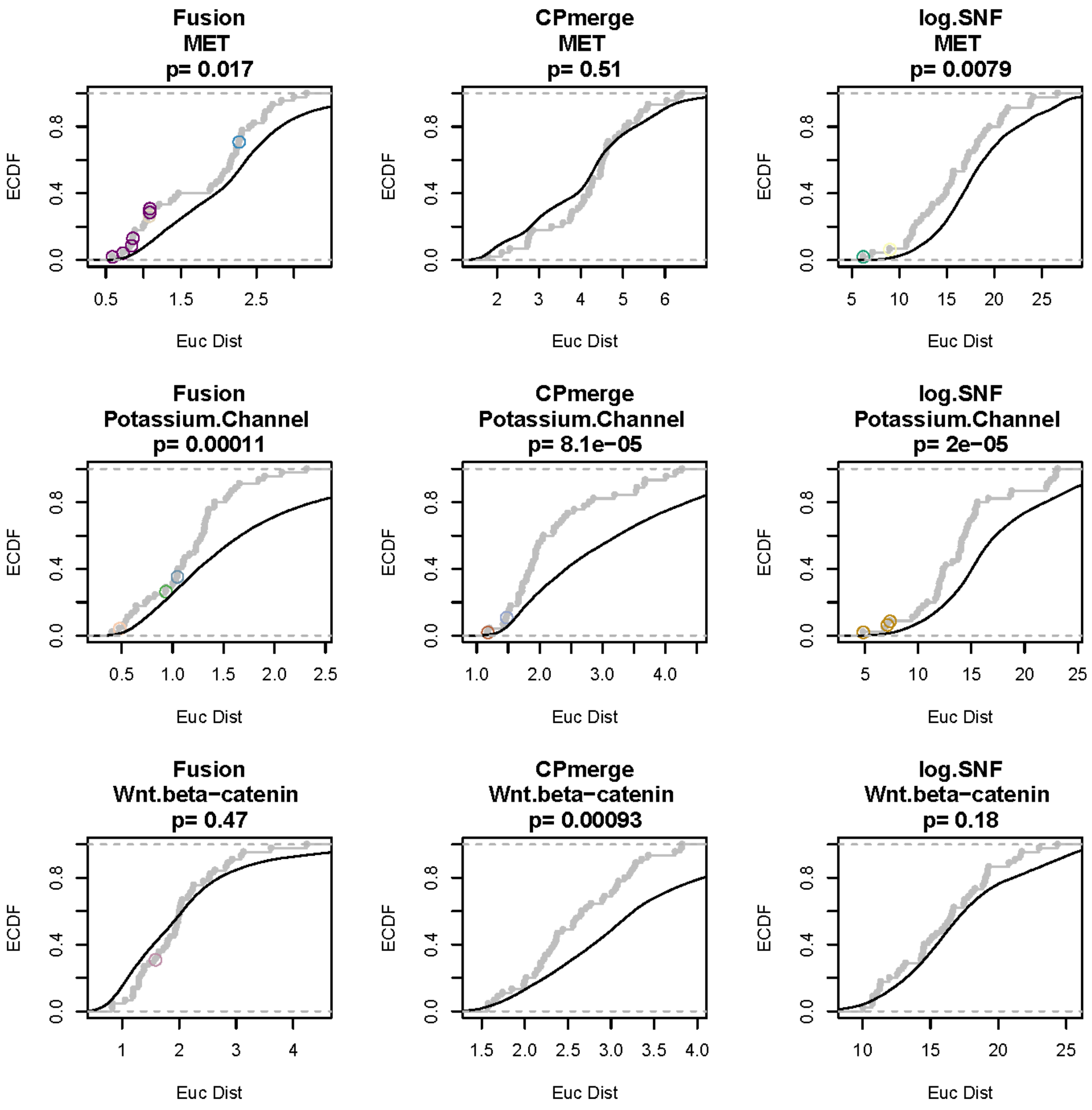
Fusion
MET
p= 0.017
CPmerge
MET
p= 0.51
log.SNF
MET
p= 0.0079
Fusion
Potassium.Channel
p= 0.00011
CPmerge
Potassium.Channel
p= 8.1e−05
log.SNF
Potassium.Channel
p= 2e−05
Fusion
Wnt.beta−catenin
p= 0.47
CPmerge
Wnt.beta−catenin
p= 0.00093
log.SNF
Wnt.beta−catenin
p= 0.18
ECDF
Euc Dist

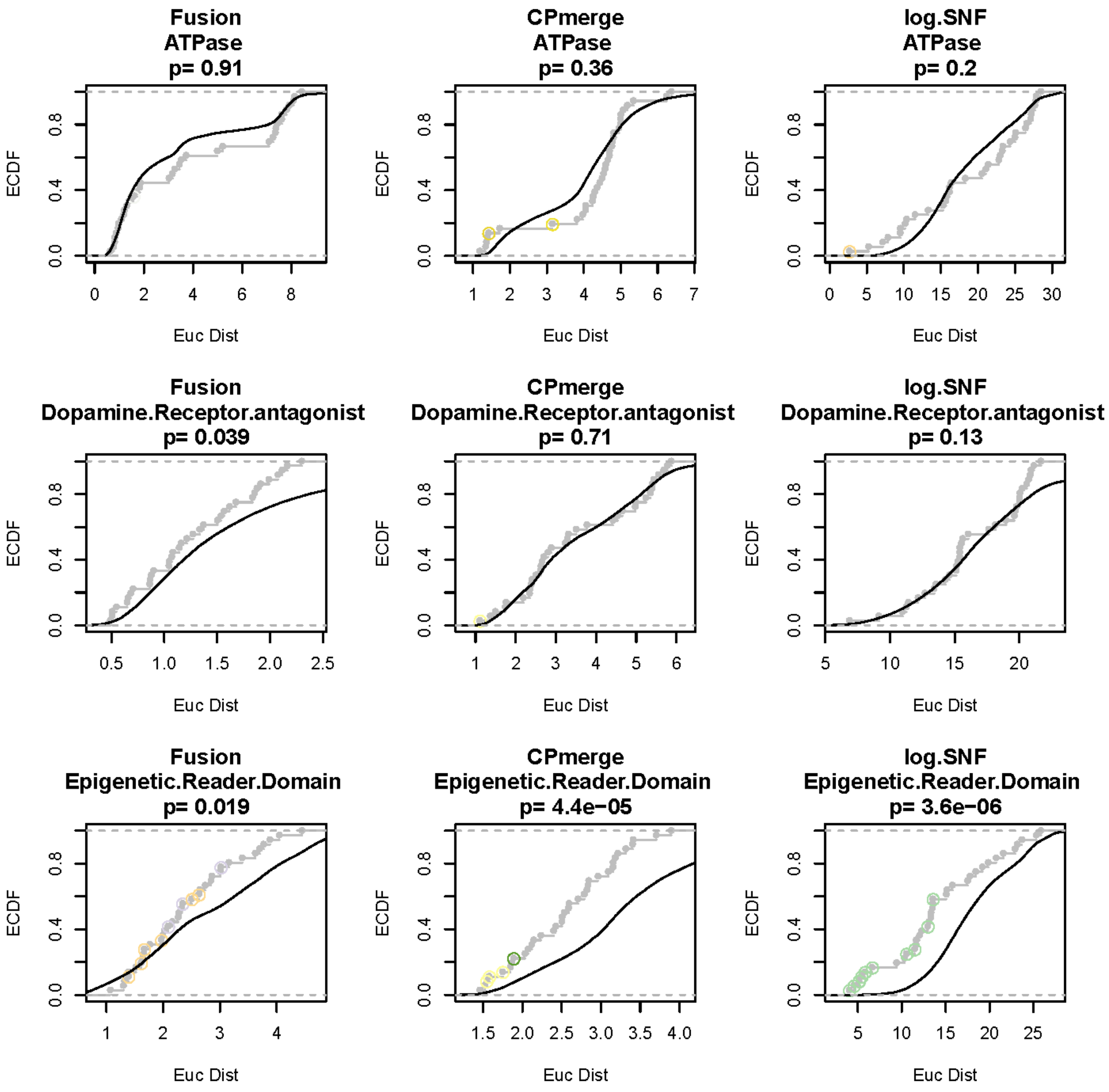
Fusion
ATPase
p= 0.91
CPmerge
ATPase
p= 0.36
log.SNF
ATPase
p= 0.2
Fusion
Dopamine.Receptor.antagonist
p= 0.039
CPmerge
Dopamine.Receptor.antagonist
p= 0.71
log.SNF
Dopamine.Receptor.antagonist
p= 0.13
Fusion
Epigenetic.Reader.Domain
p= 0.019
CPmerge
Epigenetic.Reader.Domain
p= 4.4e−05
log.SNF
Epigenetic.Reader.Domain
p= 3.6e−06
ECDF
Euc Dist

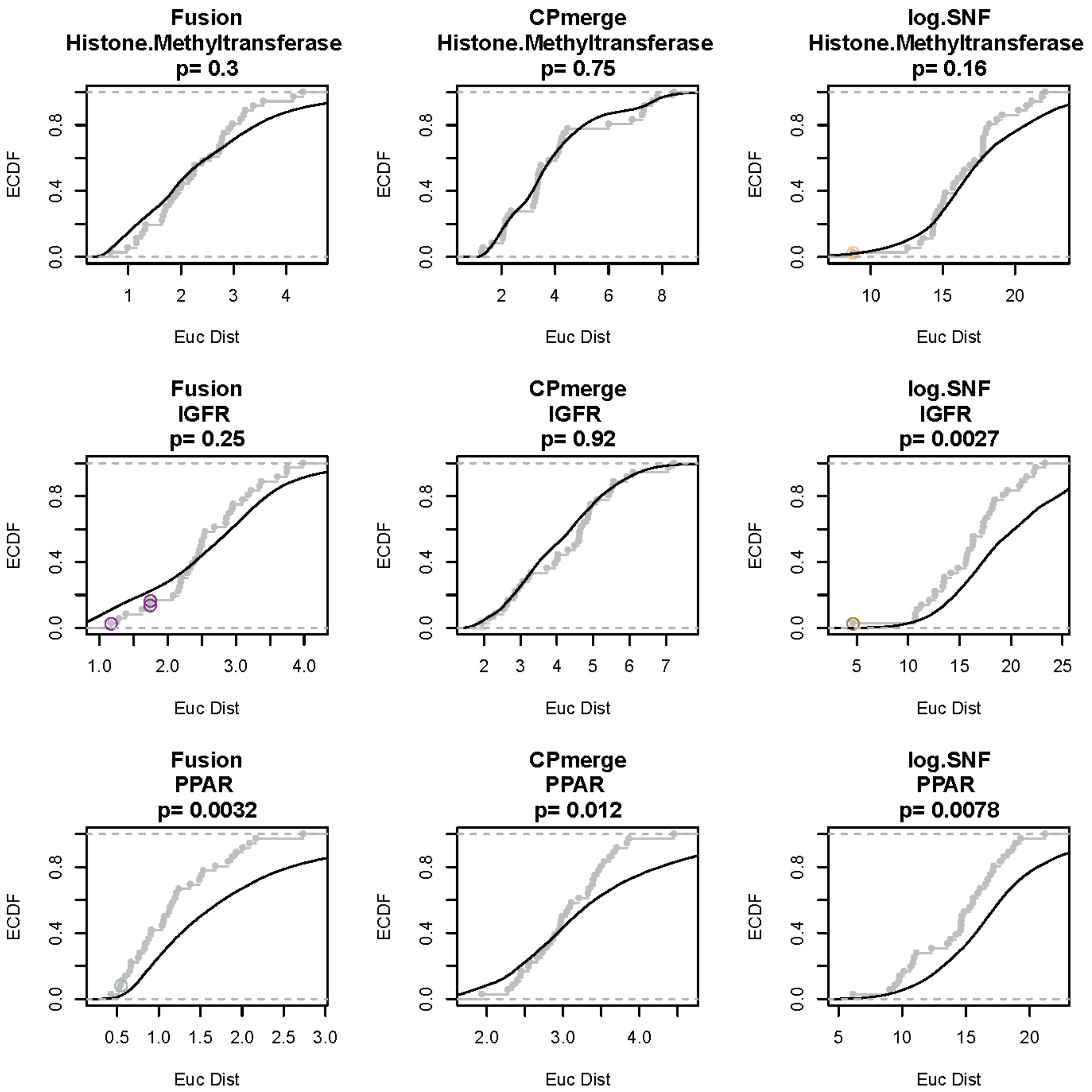
Fusion
Histone.Methyltransferase
p= 0.3
CPmerge
Histone.Methyltransferase
p= 0.75
log.SNF
Histone.Methyltransferase
p= 0.16
Fusion
IGFR
p= 0.25
CPmerge
IGFR
p= 0.92
log.SNF
IGFR
p= 0.0027
Fusion
PPAR
p= 0.0032
CPmerge
PPAR
p= 0.012
log.SNF
PPAR
p= 0.0078
ECDF
Euc Dist

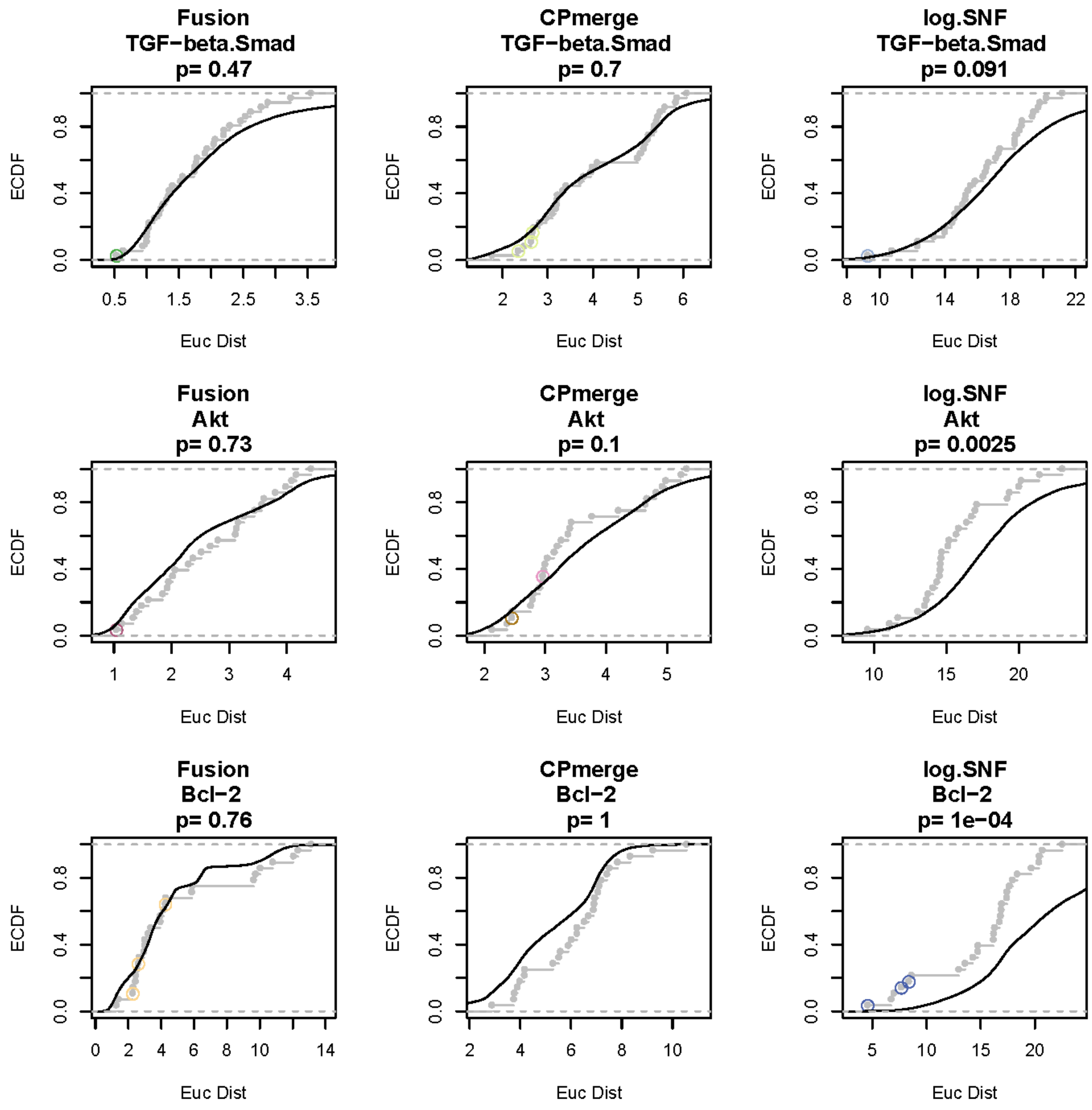
Fusion
TGF−beta.Smad
p= 0.47
CPmerge
TGF−beta.Smad
p= 0.7
log.SNF
TGF−beta.Smad
p= 0.091
Fusion
Akt
p= 0.73
CPmerge
Akt
p= 0.1
log.SNF
Akt
p= 0.0025
Fusion
Bcl−2
p= 0.76
CPmerge
Bcl−2
p= 1
log.SNF
Bcl−2
p= 1e−04
ECDF
Euc Dist

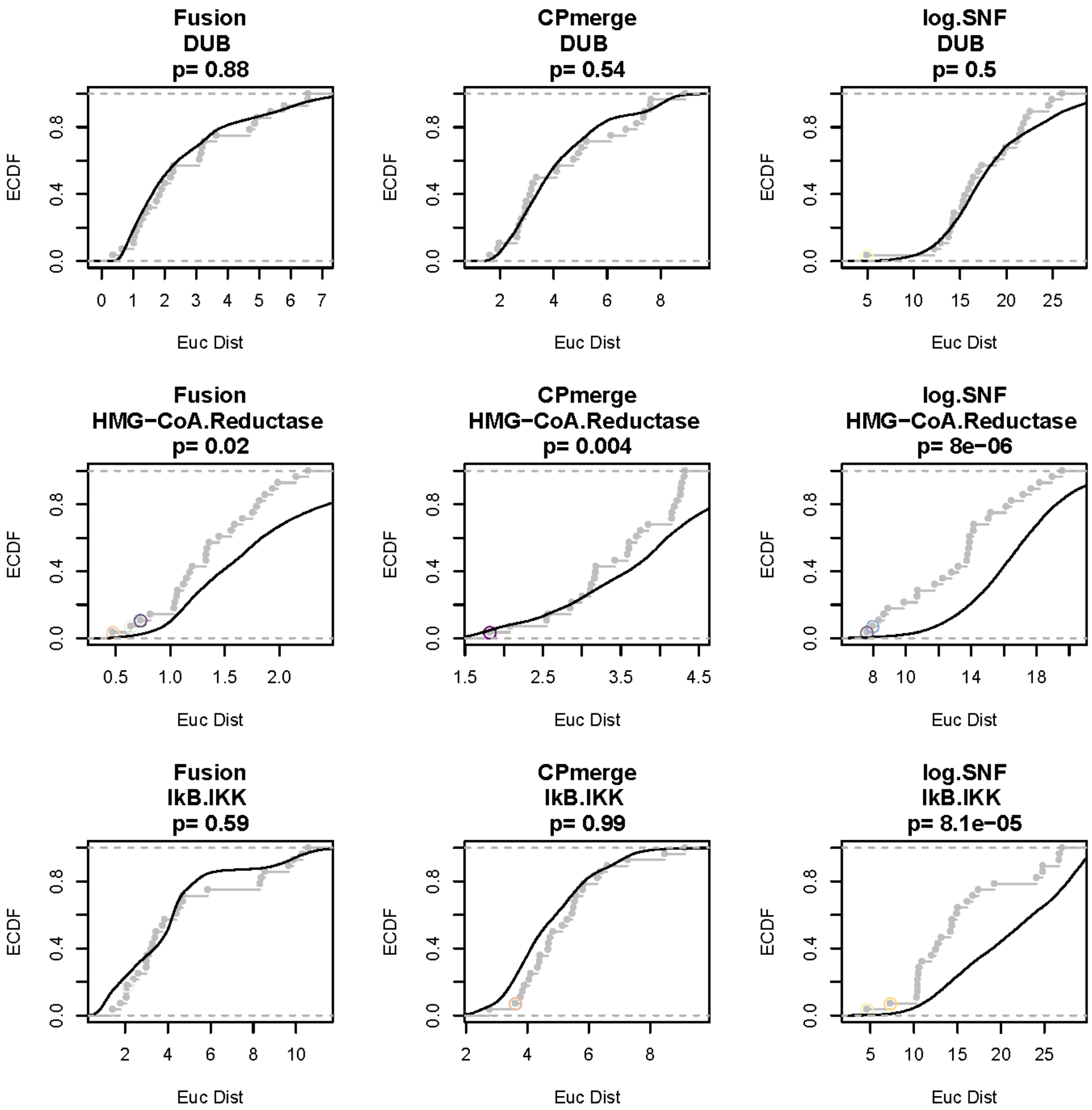

Fusion
DUB
p= 0.88
CPmerge
DUB
p= 0.54
log.SNF
DUB
p= 0.5
Fusion
HMG−CoA.Reductase
p= 0.02
CPmerge
HMG−CoA.Reductase
p= 0.004
log.SNF
HMG−CoA.Reductase
p= 8e−06
Fusion
IkB.IKK
p= 0.59
CPmerge
IkB.IKK
p= 0.99
log.SNF
IkB.IKK
p= 8.1e−05
ECDF
Euc Dist

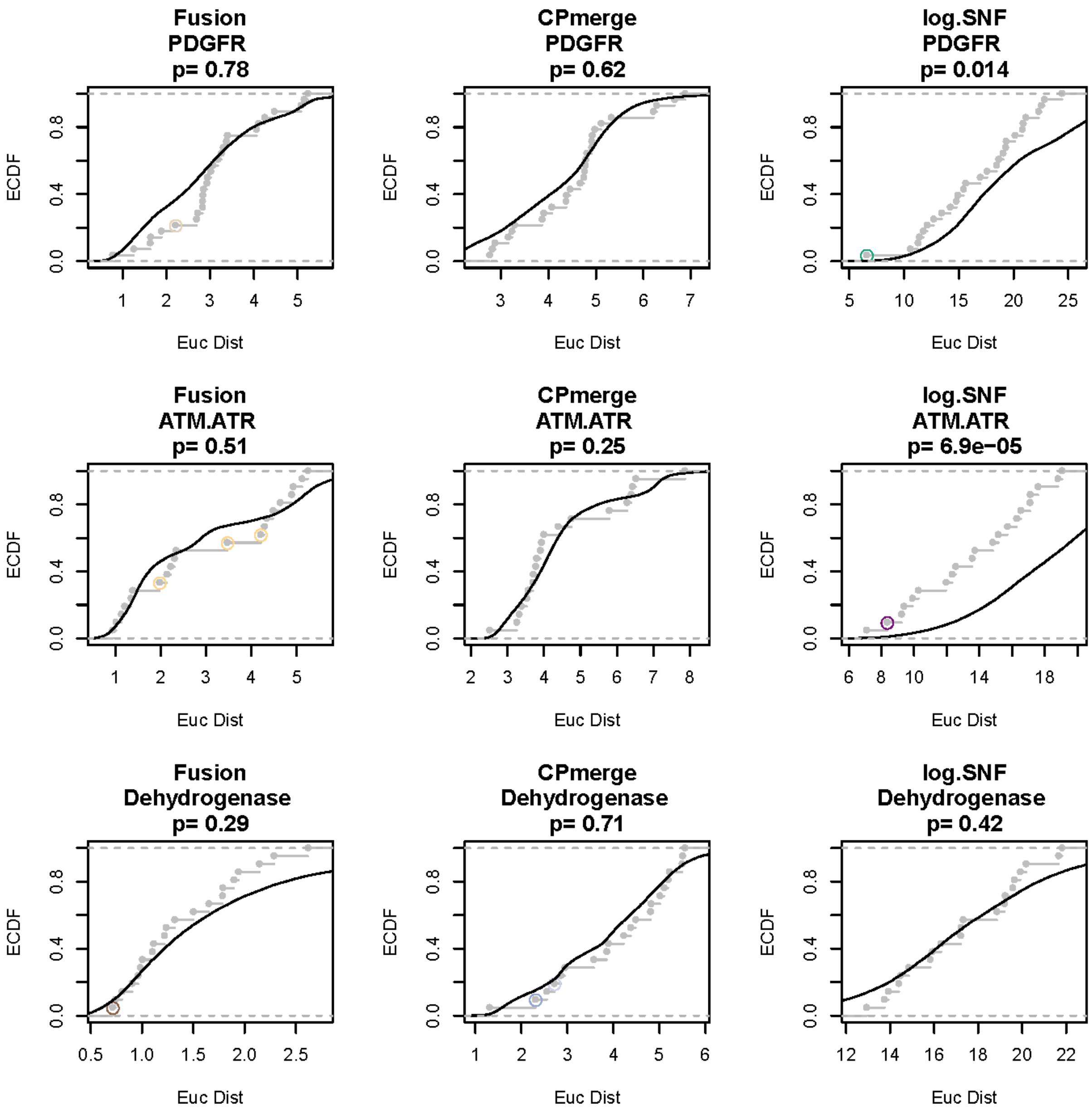
Fusion
PDGFR
p= 0.78
CPmerge
PDGFR
p= 0.62
log.SNF
PDGFR
p= 0.014
Fusion
ATM.ATR
p= 0.51
CPmerge
ATM.ATR
p= 0.25
log.SNF
ATM.ATR
p= 6.9e−05
Fusion
Dehydrogenase
p= 0.29
CPmerge
Dehydrogenase
p= 0.71
log.SNF
Dehydrogenase
p= 0.42
ECDF
Euc Dist

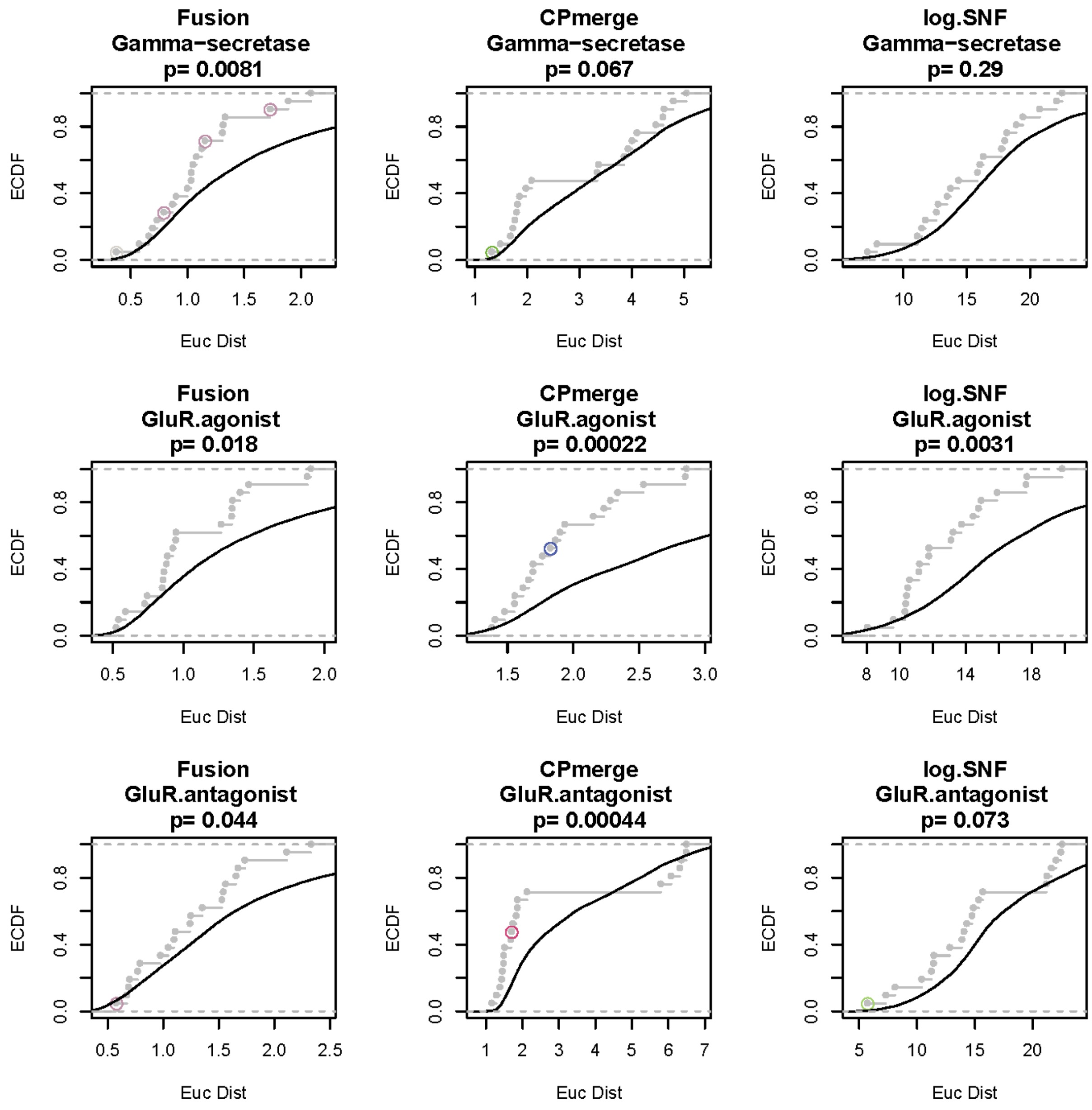

Fusion
Gamma−secretase
p= 0.0081
CPmerge
Gamma−secretase
p= 0.067
log.SNF
Gamma−secretase
p= 0.29
Fusion
GluR.agonist
p= 0.018
CPmerge
GluR.agonist
p= 0.00022
log.SNF
GluR.agonist
p= 0.0031
Fusion
GluR.antagonist
p= 0.044
CPmerge
GluR.antagonist
p= 0.00044
log.SNF
GluR.antagonist
p= 0.073
ECDF
Euc Dist

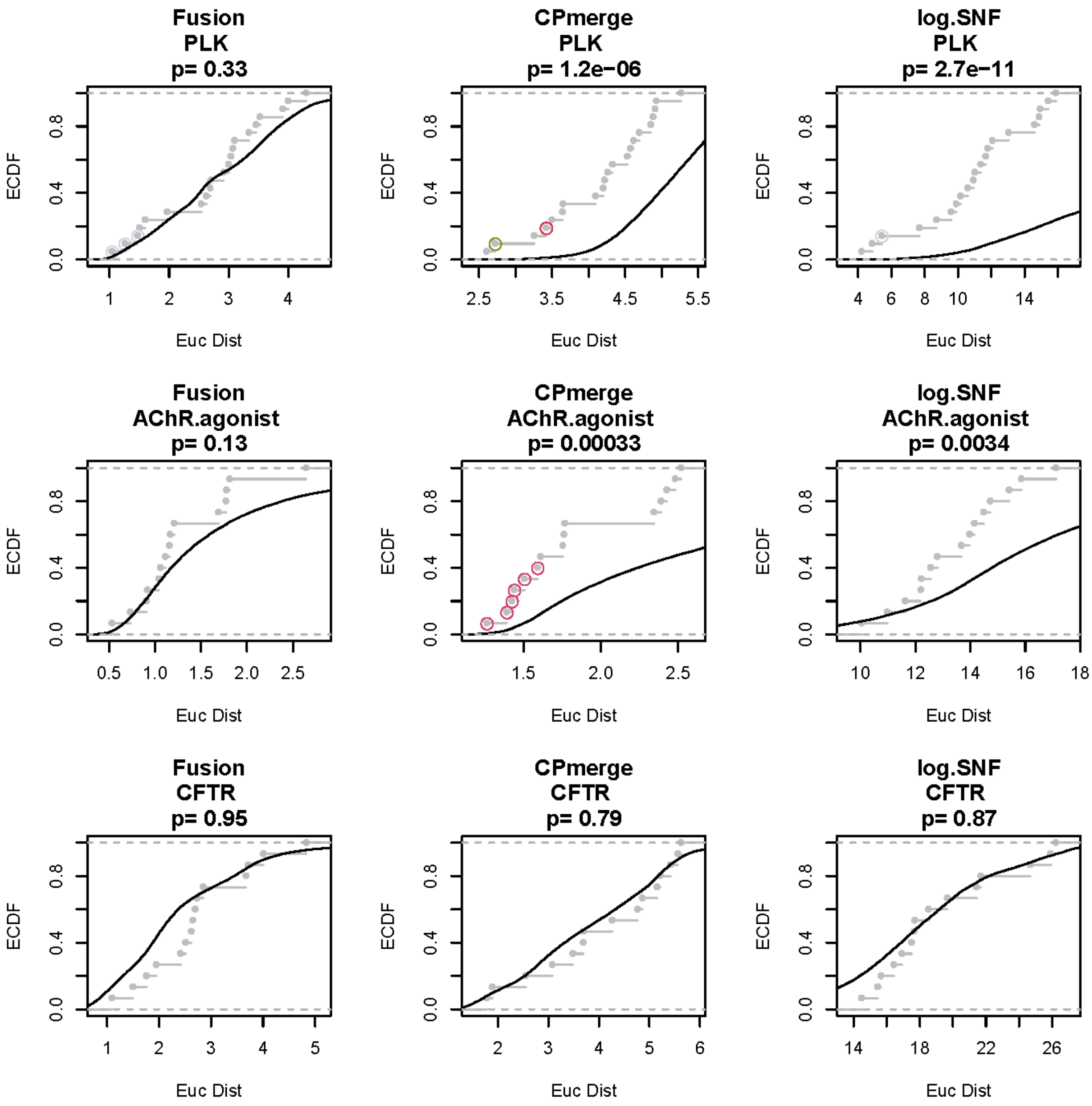
Fusion
PLK
p= 0.33
CPmerge
PLK
p= 1.2e−06
log.SNF
PLK
p= 2.7e−11
Fusion
AChR.agonist
p= 0.13
CPmerge
AChR.agonist
p= 0.00033
log.SNF
AChR.agonist
p= 0.0034
Fusion
CFTR
p= 0.95
CPmerge
CFTR
p= 0.79
log.SNF
CFTR
p= 0.87
ECDF
Euc Dist
0.0
0.4
0.8

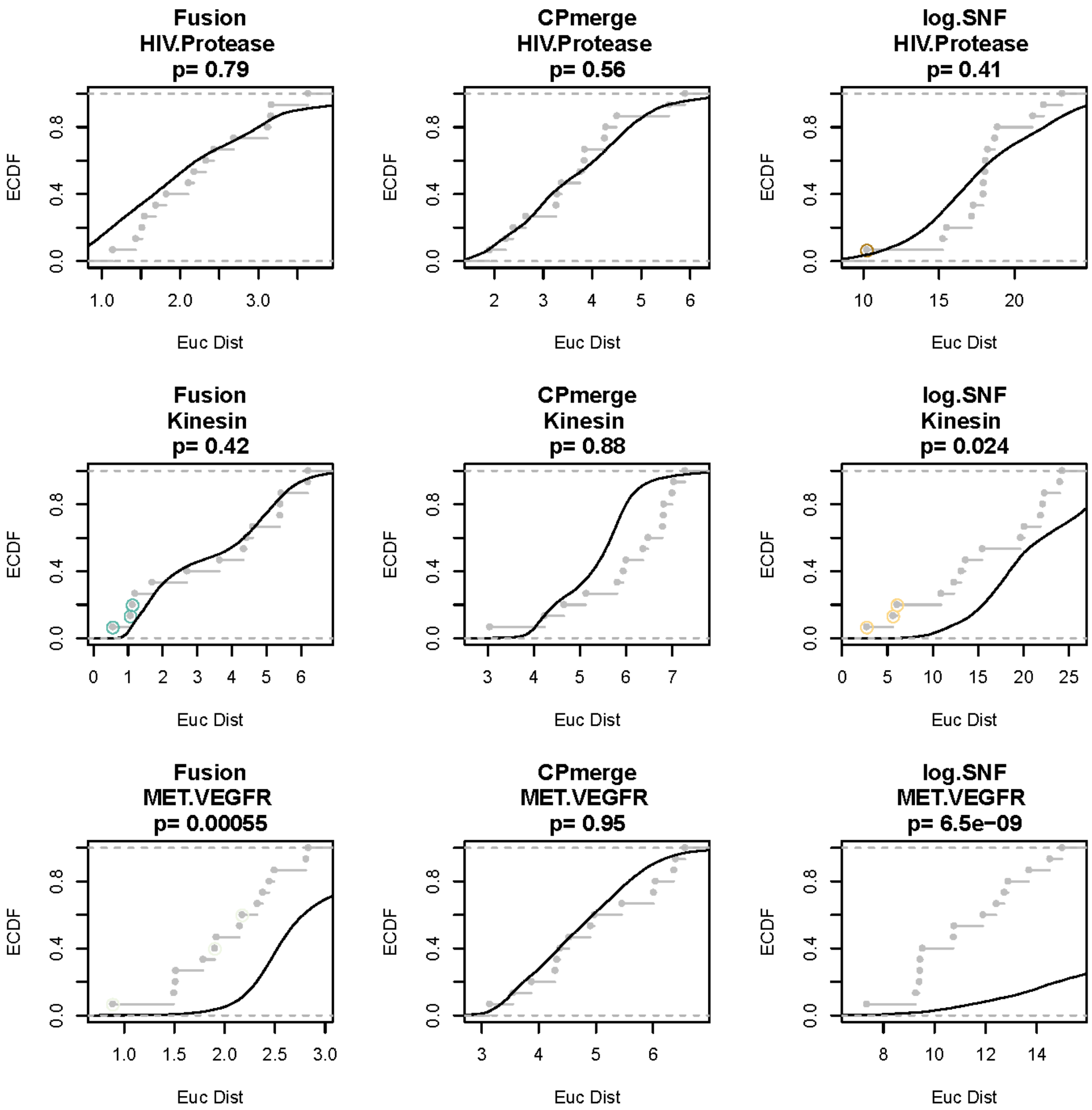
Fusion
HIV.Protease
p= 0.79
CPmerge
HIV.Protease
p= 0.56
log.SNF
HIV.Protease
p= 0.41
Fusion
Kinesin
p= 0.42
CPmerge
Kinesin
p= 0.88
log.SNF
Kinesin
p= 0.024
Fusion
MET.VEGFR
p= 0.00055
CPmerge
MET.VEGFR
p= 0.95
log.SNF
MET.VEGFR
p= 6.5e−09
ECDF
Euc Dist

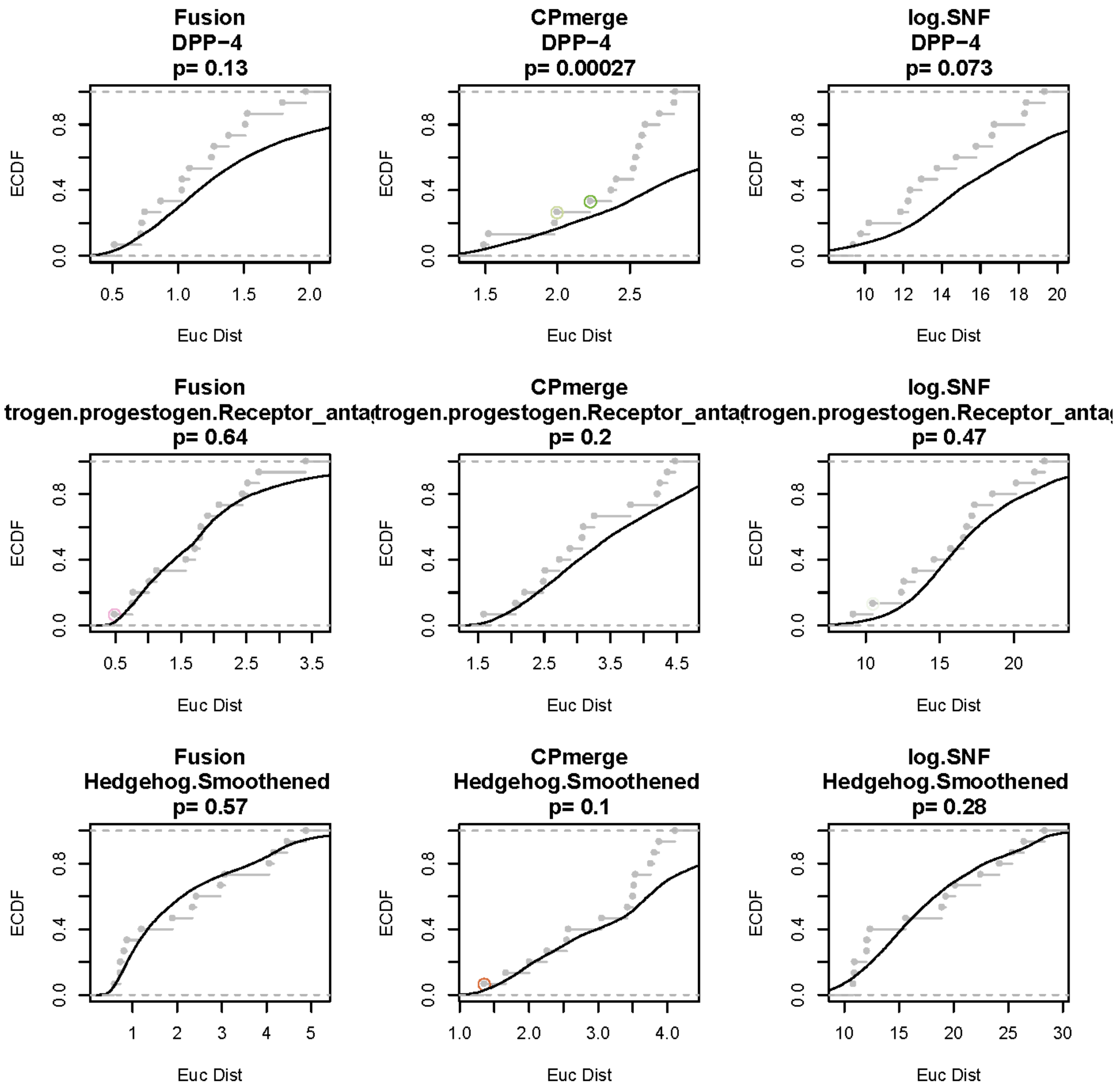
Fusion
DPP−4
p= 0.13
CPmerge
DPP−4
p= 0.00027
log.SNF
DPP−4
p= 0.073
Fusion
trogen.progestogen.Receptor_anta
p= 0.64
CPmerge
trogen.progestogen.Receptor_anta
p= 0.2
log.SNF
trogen.progestogen.Receptor_anta
p= 0.47
Fusion
Hedgehog.Smoothened
p= 0.57
CPmerge
Hedgehog.Smoothened
p= 0.1
log.SNF
Hedgehog.Smoothened
p= 0.28
ECDF
Euc Dist

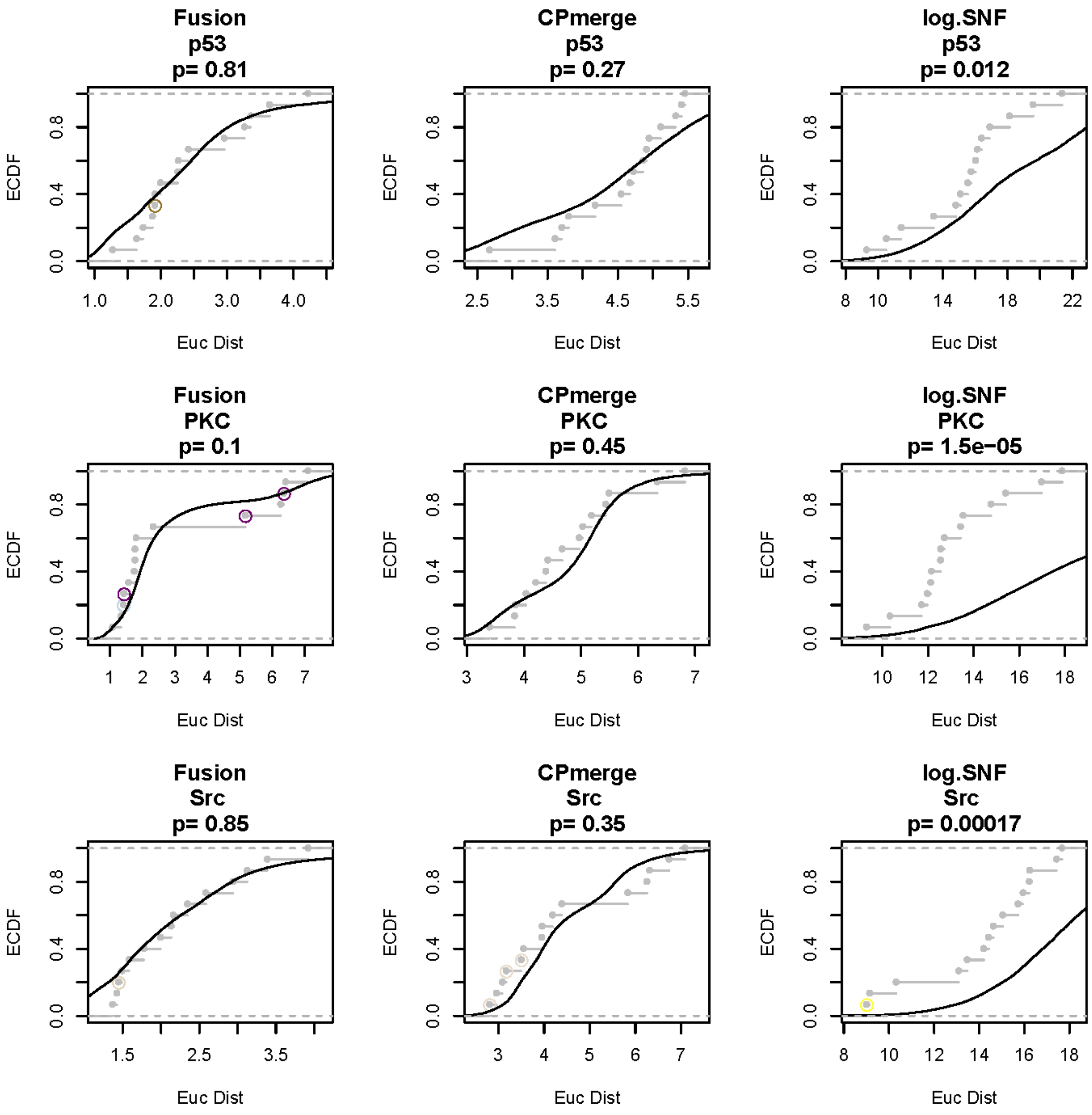

Fusion
p53
p= 0.81
CPmerge
p53
p= 0.27
log.SNF
p53
p= 0.012
Fusion
PKC
p= 0.1
CPmerge
PKC
p= 0.45
log.SNF
PKC
p= 1.5e−05
Fusion
Src
p= 0.85
CPmerge
Src
p= 0.35
log.SNF
Src
p= 0.00017
ECDF
Euc Dist

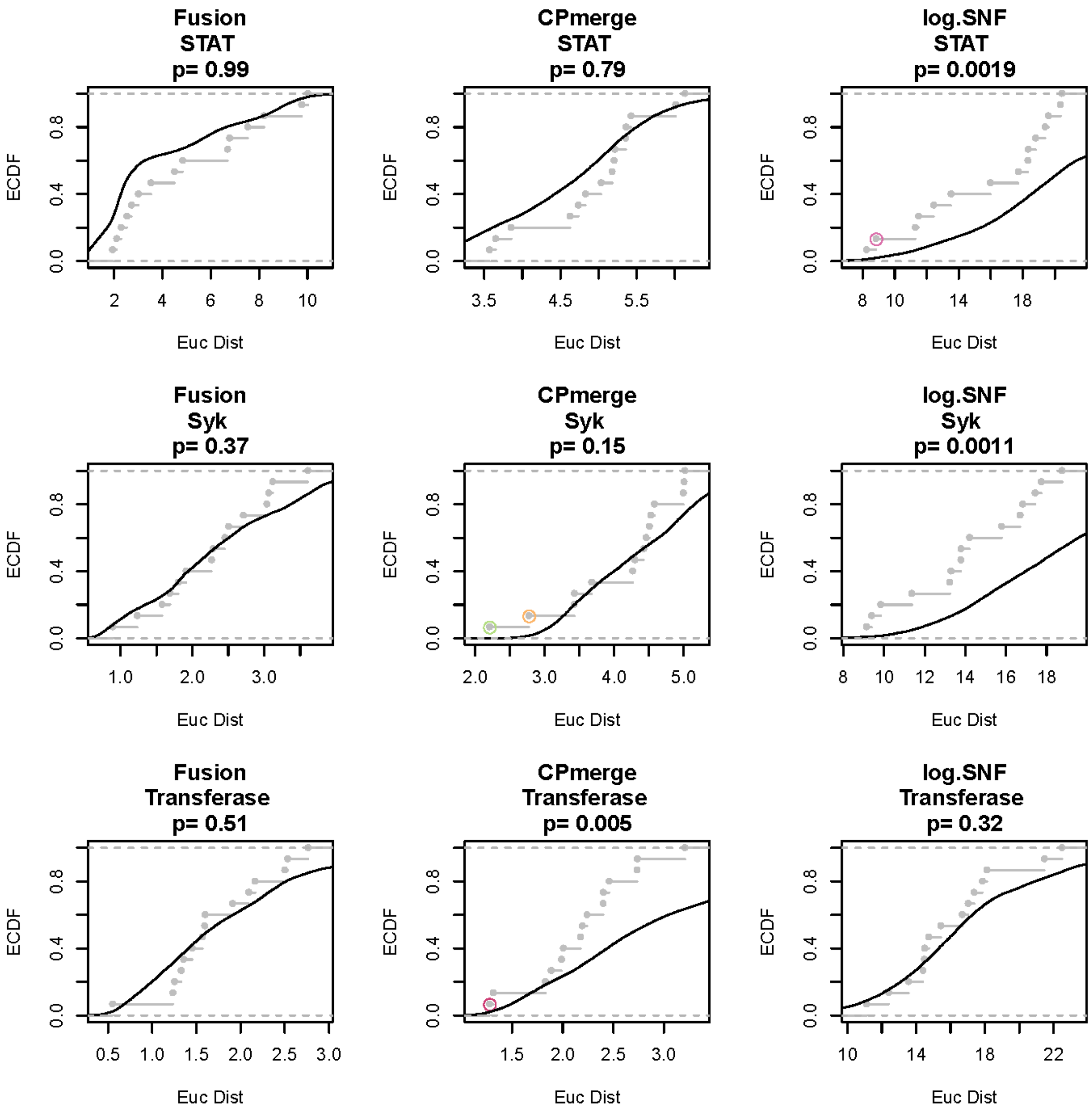
Fusion
STAT
p= 0.99
CPmerge
STAT
p= 0.79
log.SNF
STAT
p= 0.0019
Fusion
Syk
p= 0.37
CPmerge
Syk
p= 0.15
log.SNF
Syk
p= 0.0011
Fusion
Transferase
p= 0.51
CPmerge
Transferase
p= 0.005
log.SNF
Transferase
p= 0.32
ECDF
Euc Dist

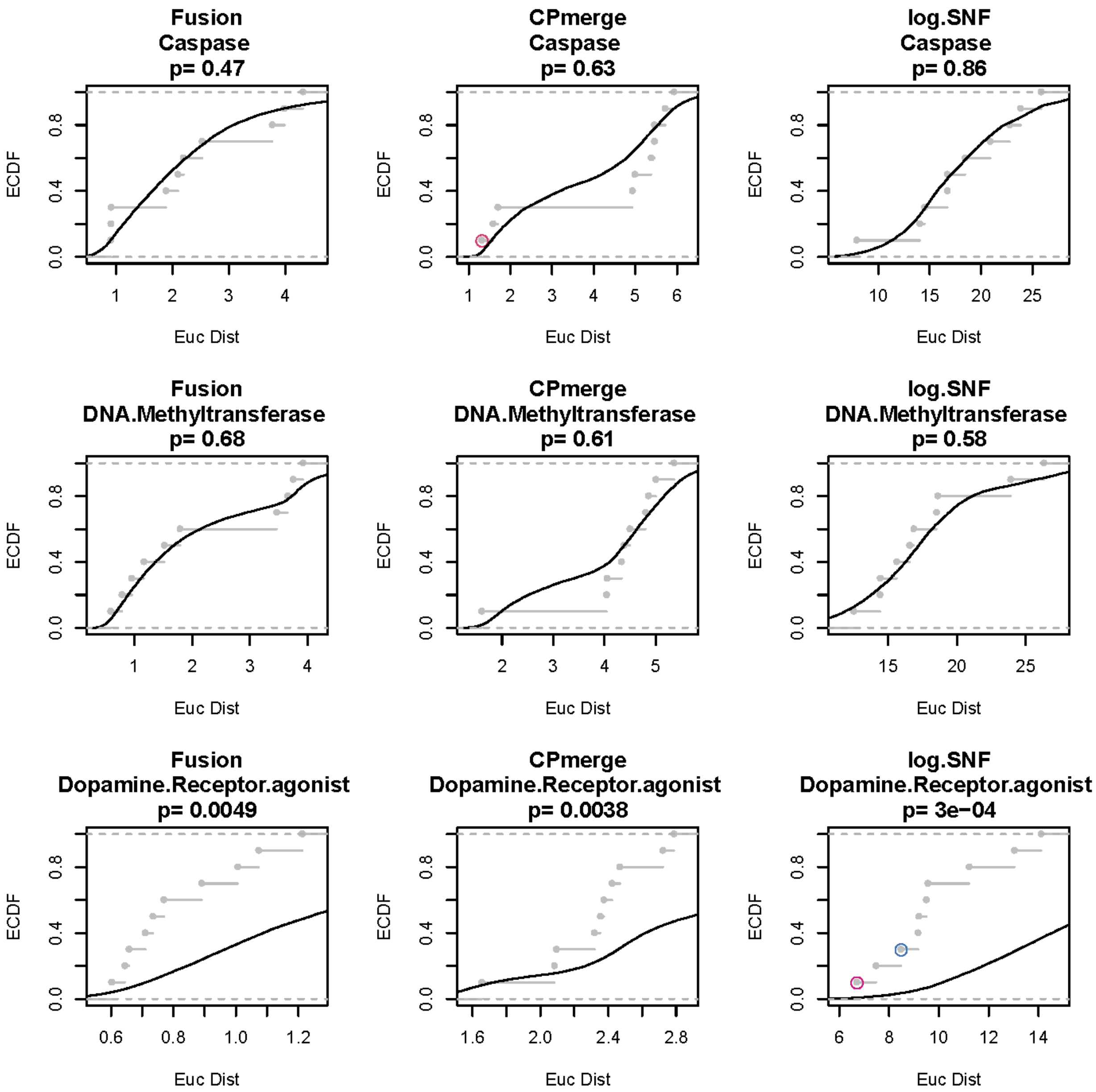
Fusion
Caspase
p= 0.47
CPmerge
Caspase
p= 0.63
log.SNF
Caspase
p= 0.86
Fusion
DNA.Methyltransferase
p= 0.68
CPmerge
DNA.Methyltransferase
p= 0.61
log.SNF
DNA.Methyltransferase
p= 0.58
Fusion
Dopamine.Receptor.agonist
p= 0.0049
CPmerge
Dopamine.Receptor.agonist
p= 0.0038
log.SNF
Dopamine.Receptor.agonist
p= 3e−04
ECDF
Euc Dist

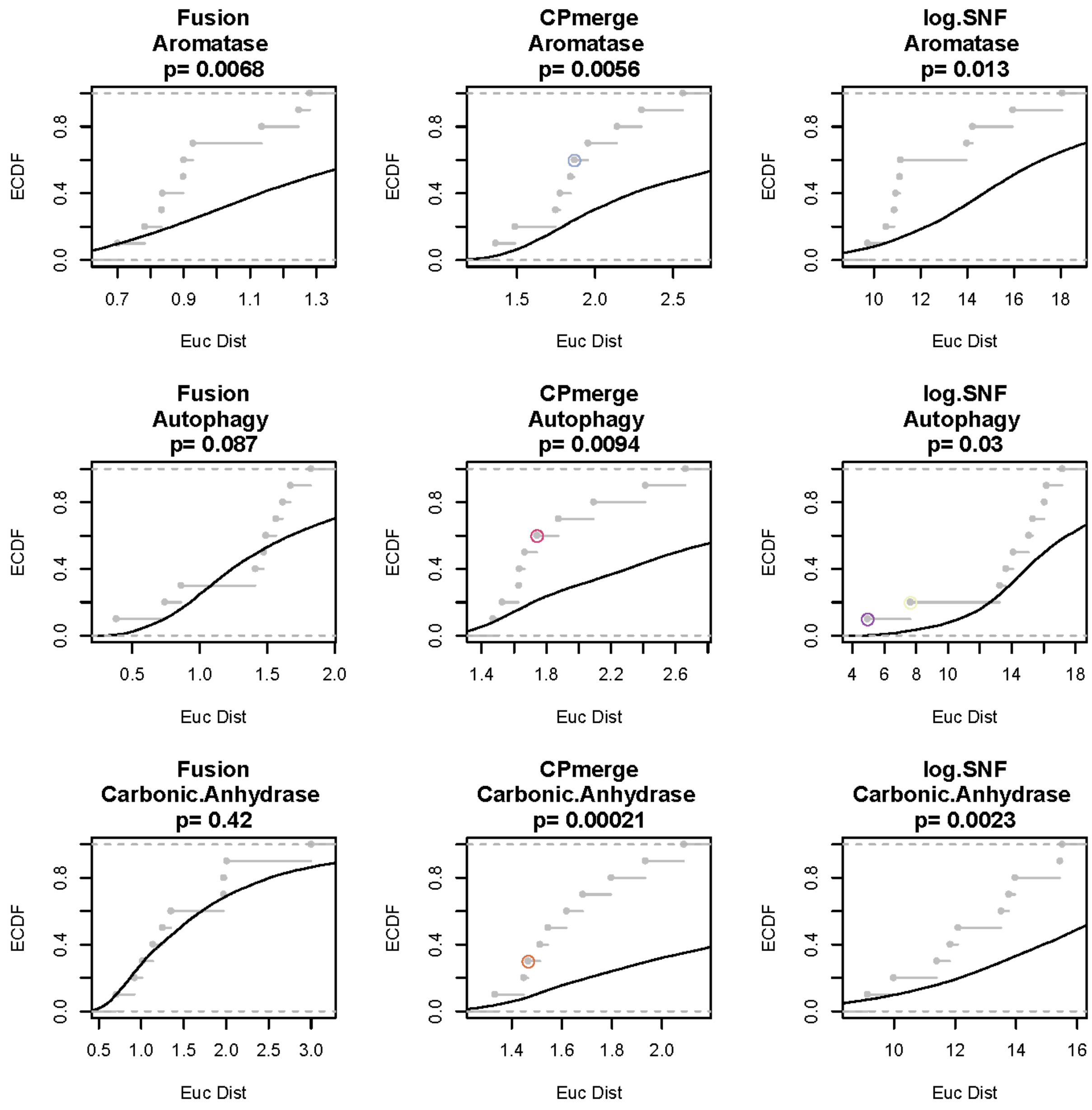
Fusion
Aromatase
p= 0.0068
CPmerge
Aromatase
p= 0.0056
log.SNF
Aromatase
p= 0.013
Fusion
Autophagy
p= 0.087
CPmerge
Autophagy
p= 0.0094
log.SNF
Autophagy
p= 0.03
Fusion
Carbonic.Anhydrase
p= 0.42
CPmerge
Carbonic.Anhydrase
p= 0.00021
log.SNF
Carbonic.Anhydrase
p= 0.0023
ECDF
Euc Dist

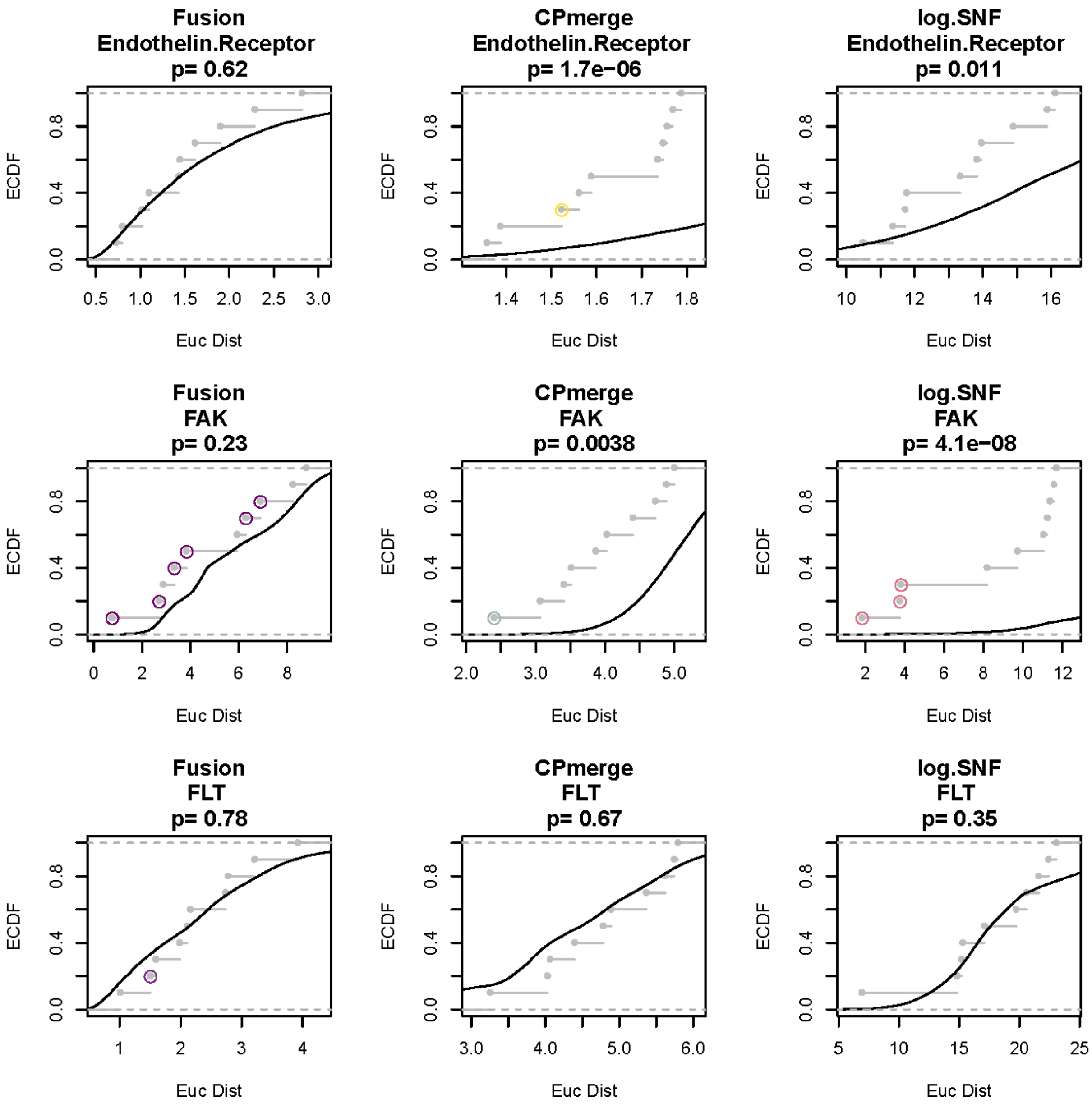

Fusion
Endothelin.Receptor
p= 0.62
CPmerge
Endothelin.Receptor
p= 1.7e−06
log.SNF
Endothelin.Receptor
p= 0.011
Fusion
FAK
p= 0.23
CPmerge
FAK
p= 0.0038
log.SNF
FAK
p= 4.1e−08
Fusion
FLT
p= 0.78
CPmerge
FLT
p= 0.67
log.SNF
FLT
p= 0.35
ECDF
Euc Dist

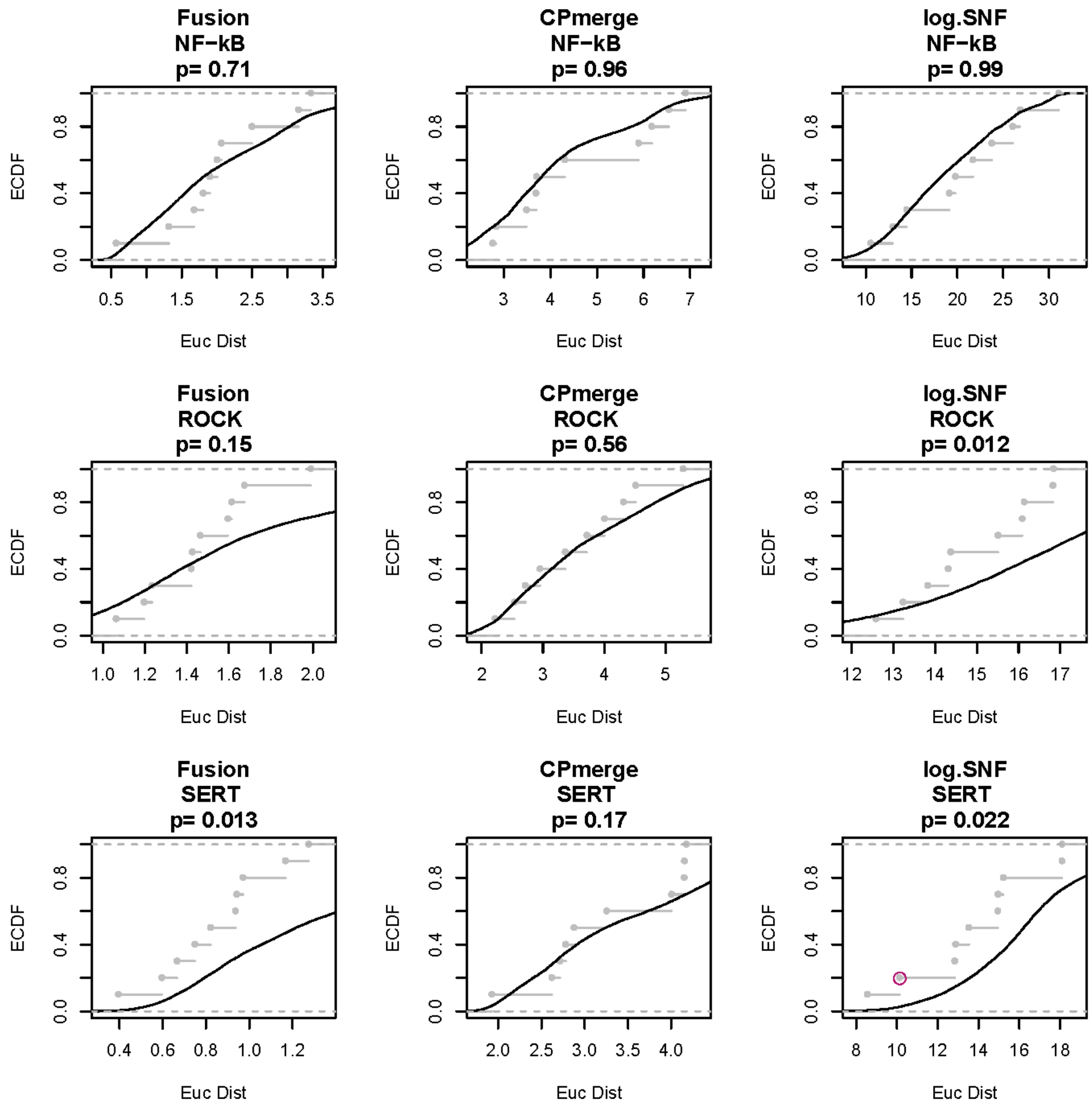
Fusion
NF−kB
p= 0.71
CPmerge
NF−kB
p= 0.96
log.SNF
NF−kB
p= 0.99
Fusion
ROCK
p= 0.15
CPmerge
ROCK
p= 0.56
log.SNF
ROCK
p= 0.012
Fusion
SERT
p= 0.013
CPmerge
SERT
p= 0.17
log.SNF
SERT
p= 0.022
ECDF
Euc Dist

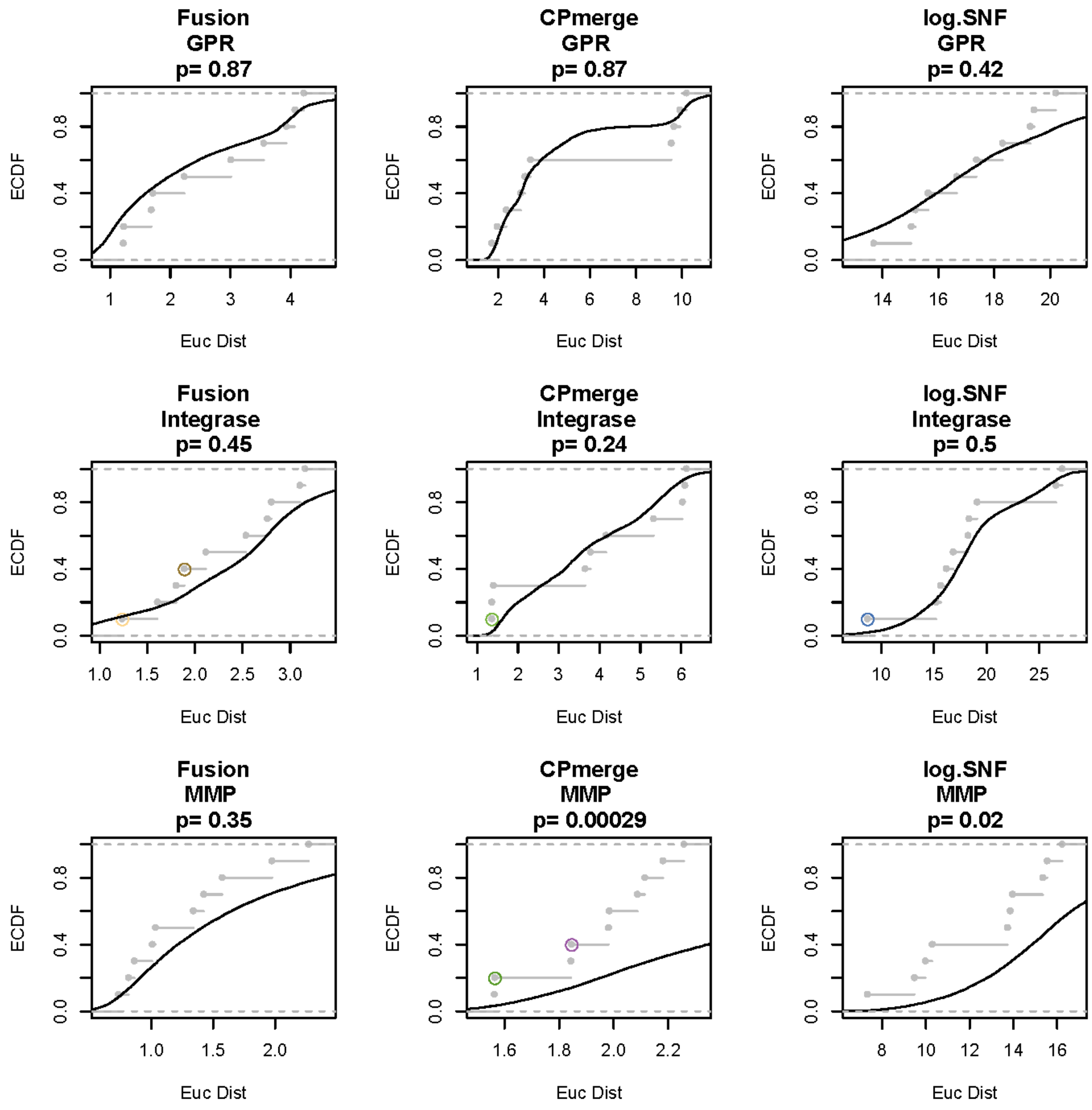
Fusion
GPR
p= 0.87
CPmerge
GPR
p= 0.87
log.SNF
GPR
p= 0.42
Fusion
Integrase
p= 0.45
CPmerge
Integrase
p= 0.24
log.SNF
Integrase
p= 0.5
Fusion
MMP
p= 0.35
CPmerge
MMP
p= 0.00029
log.SNF
MMP
p= 0.02
ECDF
Euc Dist

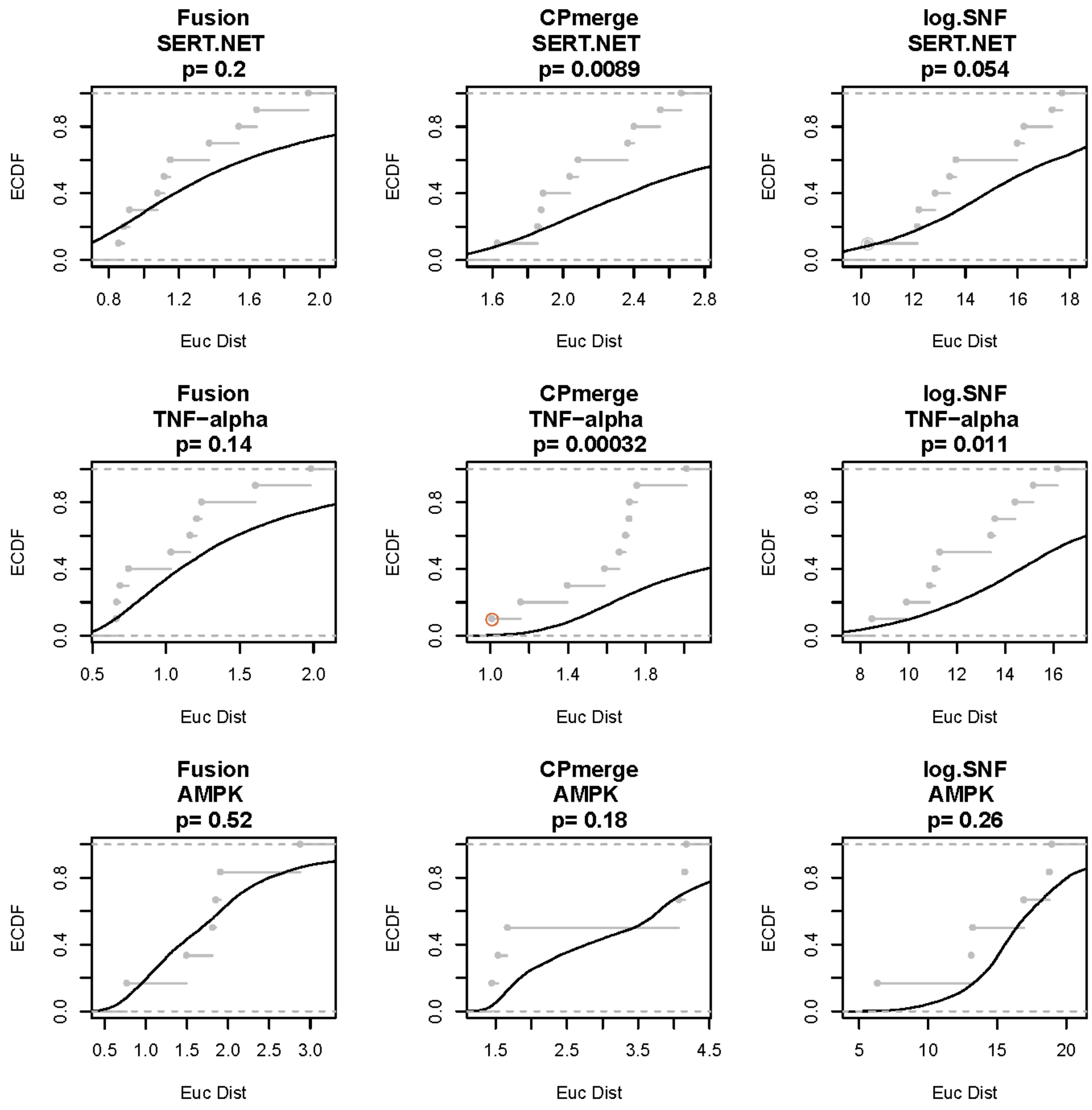
Fusion
SERT.NET
p= 0.2
CPmerge
SERT.NET
p= 0.0089
log.SNF
SERT.NET
p= 0.054
Fusion
TNF−alpha
p= 0.14
CPmerge
TNF−alpha
p= 0.00032
log.SNF
TNF−alpha
p= 0.011
Fusion
AMPK
p= 0.52
CPmerge
AMPK
p= 0.18
log.SNF
AMPK
p= 0.26
ECDF
Euc Dist

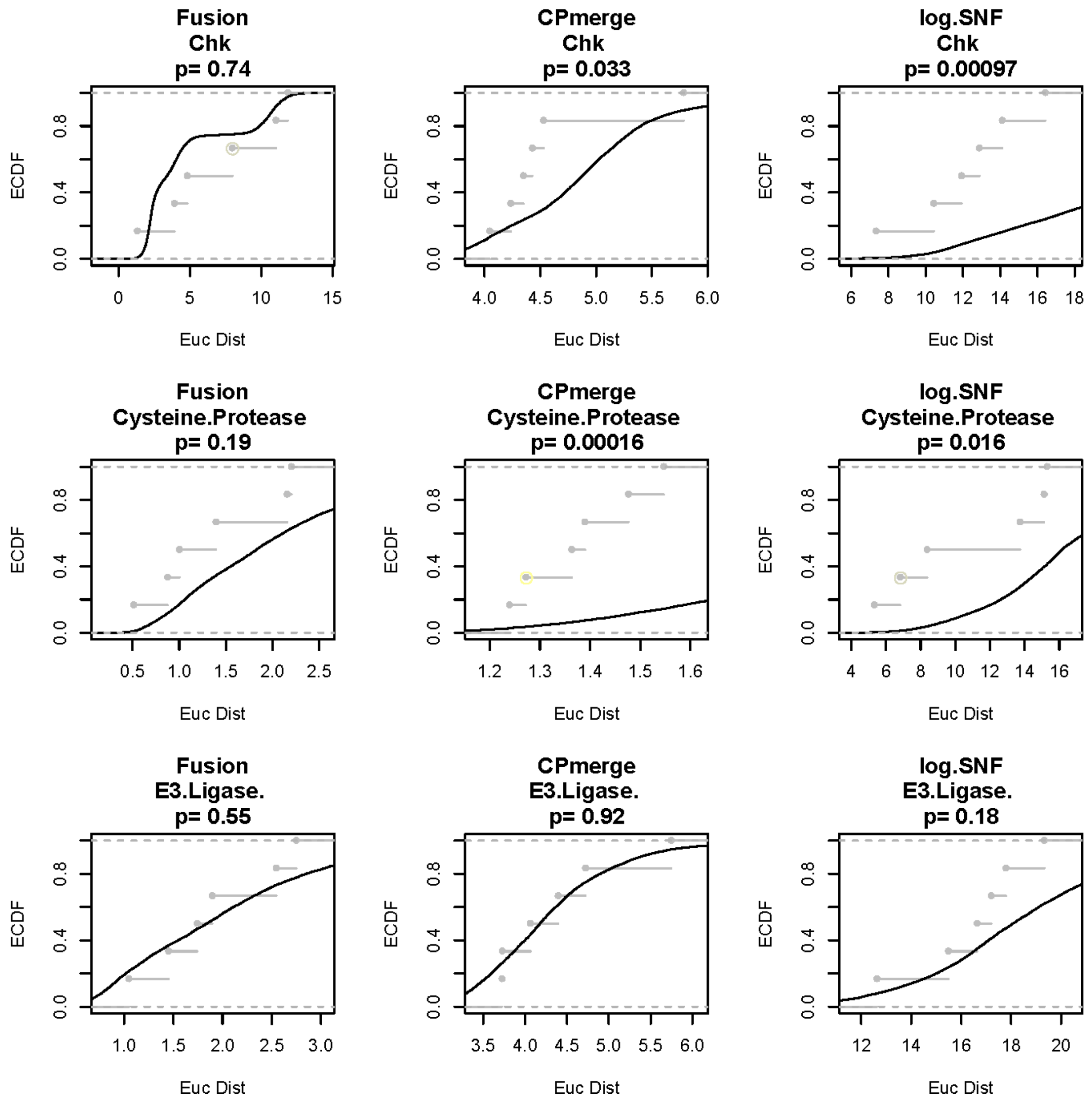
Fusion
Chk
p= 0.74
CPmerge
Chk
p= 0.033
log.SNF
Chk
p= 0.00097
Fusion
Cysteine.Protease
p= 0.19
CPmerge
Cysteine.Protease
p= 0.00016
log.SNF
Cysteine.Protease
p= 0.016
Fusion
E3.Ligase.
p= 0.55
CPmerge
E3.Ligase.
p= 0.92
log.SNF
E3.Ligase.
p= 0.18
ECDF
Euc Dist

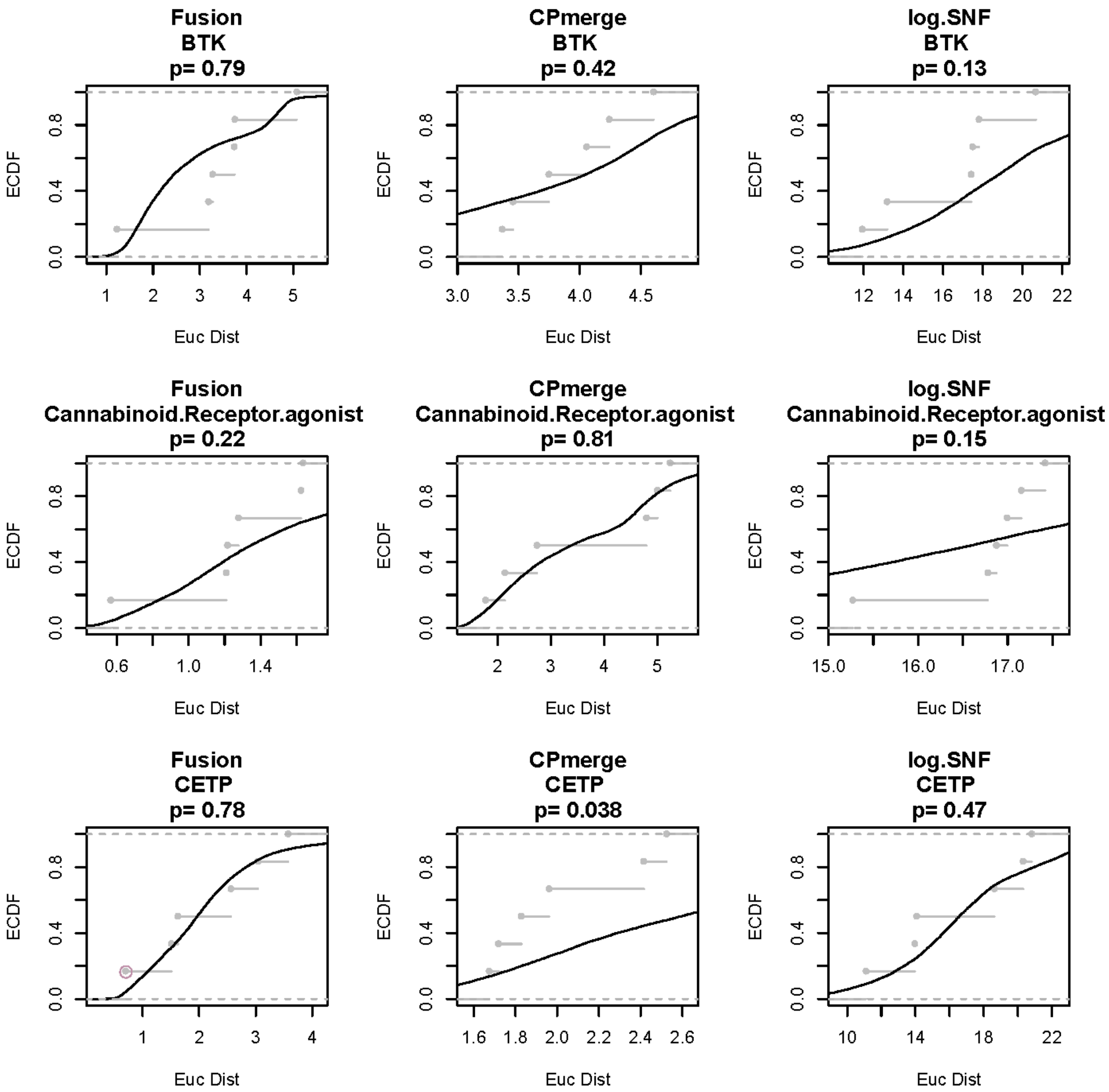
Fusion
BTK
p= 0.79
CPmerge
BTK
p= 0.42
log.SNF
BTK
p= 0.13
Fusion
Cannabinoid.Receptor.agonist
p= 0.22
CPmerge
Cannabinoid.Receptor.agonist
p= 0.81
log.SNF
Cannabinoid.Receptor.agonist
p= 0.15
Fusion
CETP
p= 0.78
CPmerge
CETP
p= 0.038
log.SNF
CETP
p= 0.47
ECDF
Euc Dist

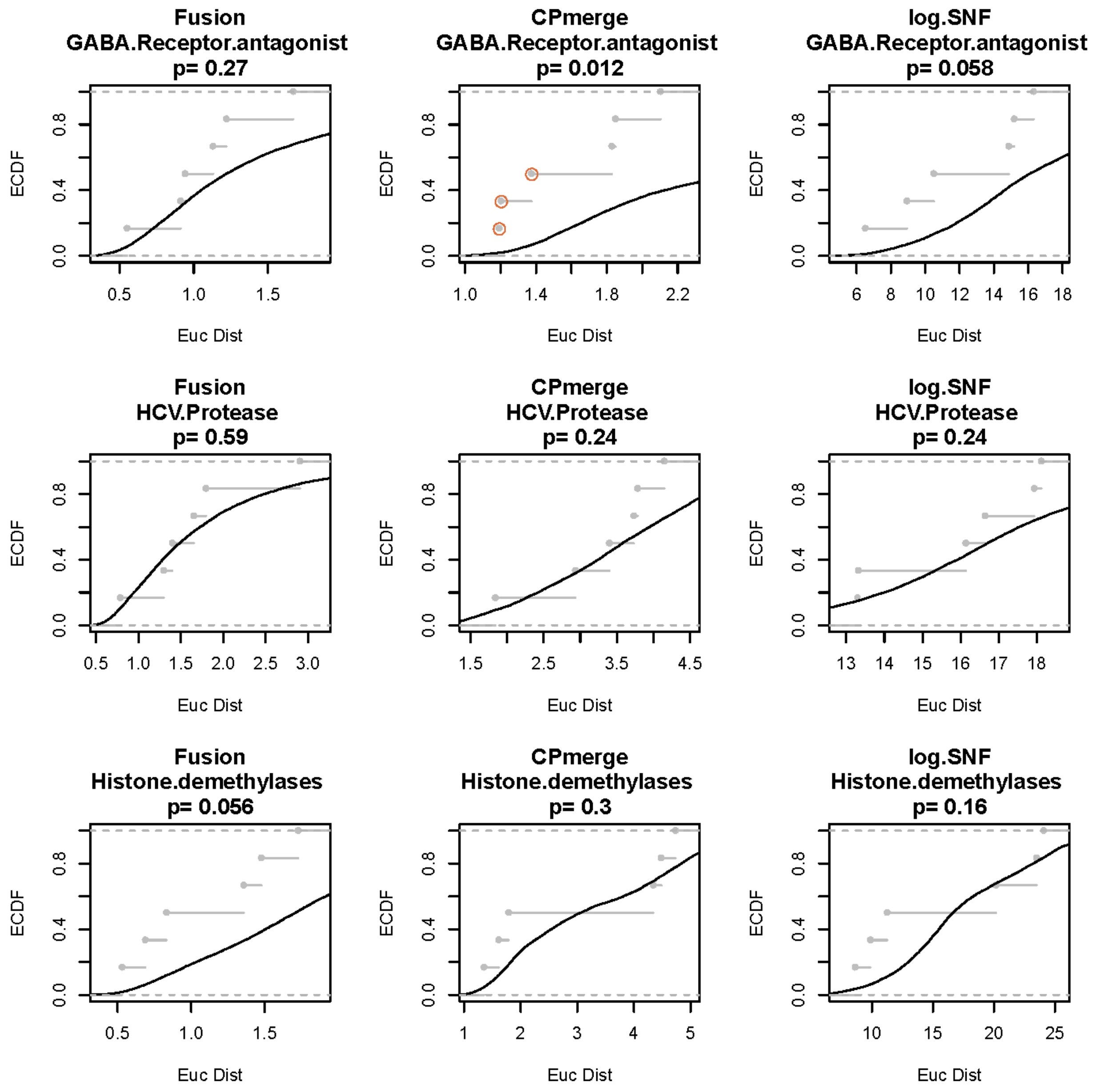
Fusion
GABA.Receptor.antagonist
p= 0.27
CPmerge
GABA.Receptor.antagonist
p= 0.012
log.SNF
GABA.Receptor.antagonist
p= 0.058
Fusion
HCV.Protease
p= 0.59
CPmerge
HCV.Protease
p= 0.24
log.SNF
HCV.Protease
p= 0.24
Fusion
Histone.demethylases
p= 0.056
CPmerge
Histone.demethylases
p= 0.3
log.SNF
Histone.demethylases
p= 0.16
ECDF
Euc Dist

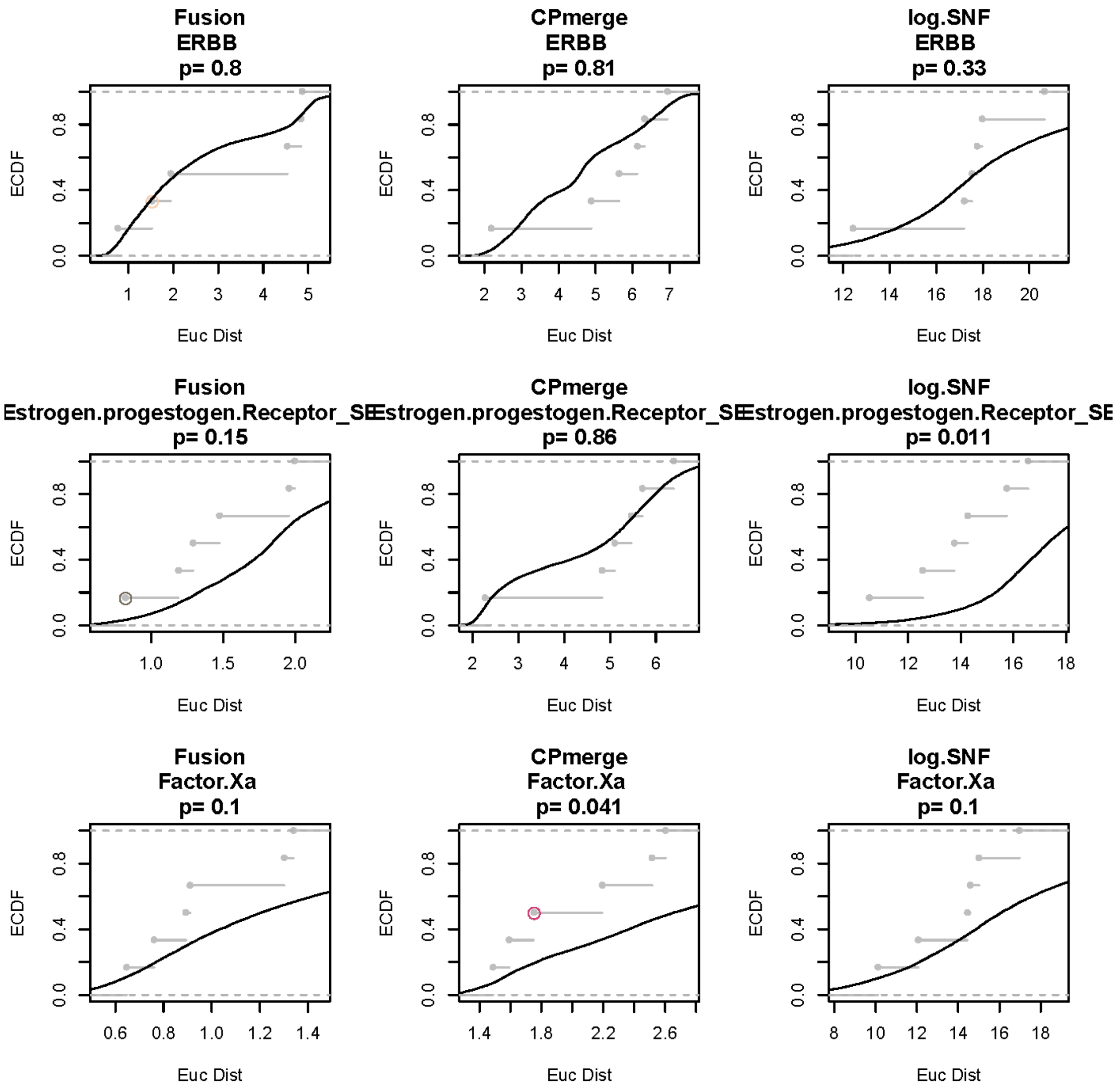
Fusion
ERBB
p= 0.8
CPmerge
ERBB
p= 0.81
log.SNF
ERBB
p= 0.33
Fusion
Estrogen.progestogen.Receptor_SE
p= 0.15
CPmerge
Estrogen.progestogen.Receptor_SE
p= 0.86
log.SNF
Estrogen.progestogen.Receptor_SE
p= 0.011
Fusion
Factor.Xa
p= 0.1
CPmerge
Factor.Xa
p= 0.041
log.SNF
Factor.Xa
p= 0.1
ECDF
Euc Dist

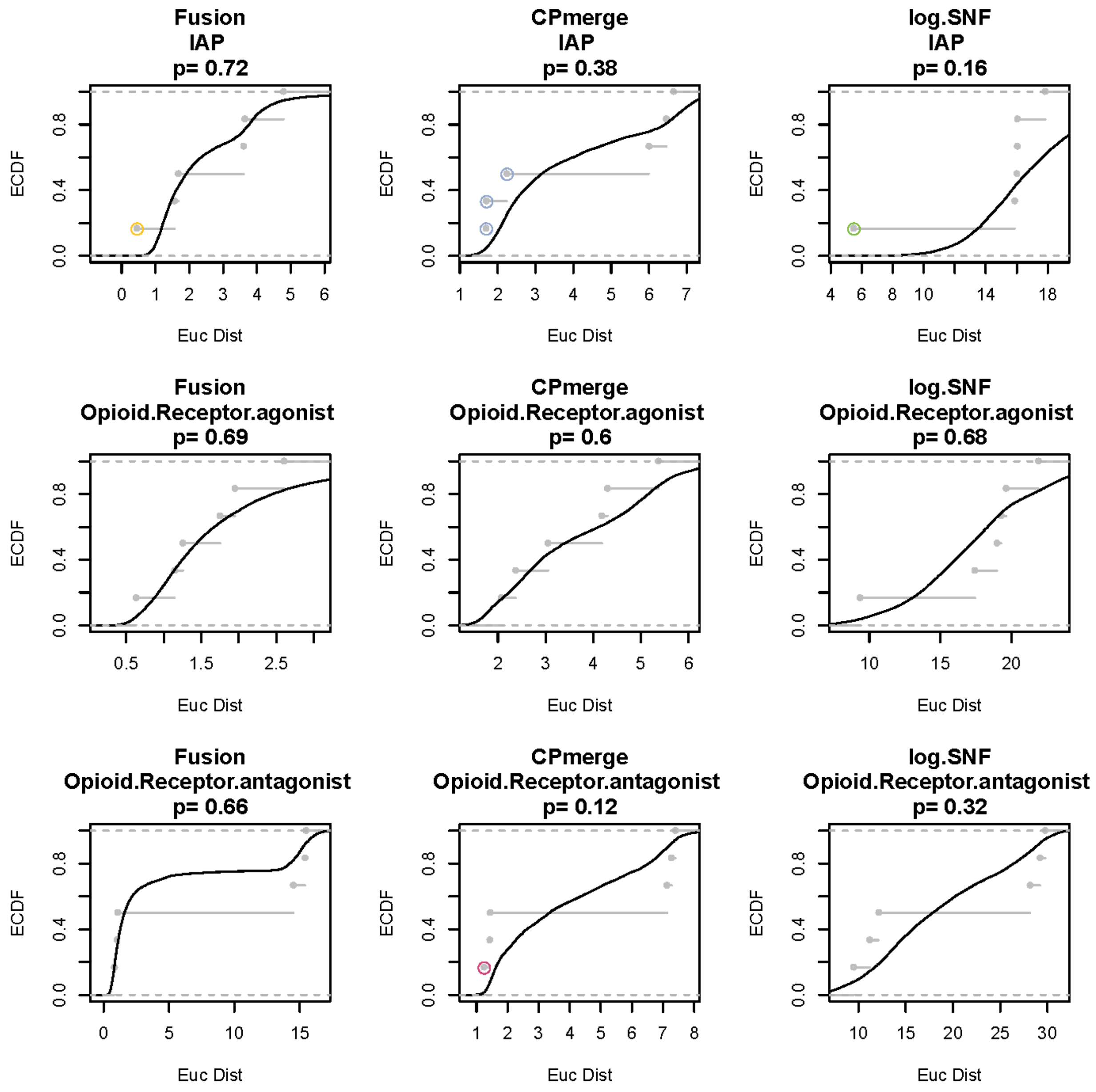

Fusion
IAP
p= 0.72
CPmerge
IAP
p= 0.38
log.SNF
IAP
p= 0.16
Fusion
Opioid.Receptor.agonist
p= 0.69
CPmerge
Opioid.Receptor.agonist
p= 0.6
log.SNF
Opioid.Receptor.agonist
p= 0.68
Fusion
Opioid.Receptor.antagonist
p= 0.66
CPmerge
Opioid.Receptor.antagonist
p= 0.12
log.SNF
Opioid.Receptor.antagonist
p= 0.32
ECDF
Euc Dist

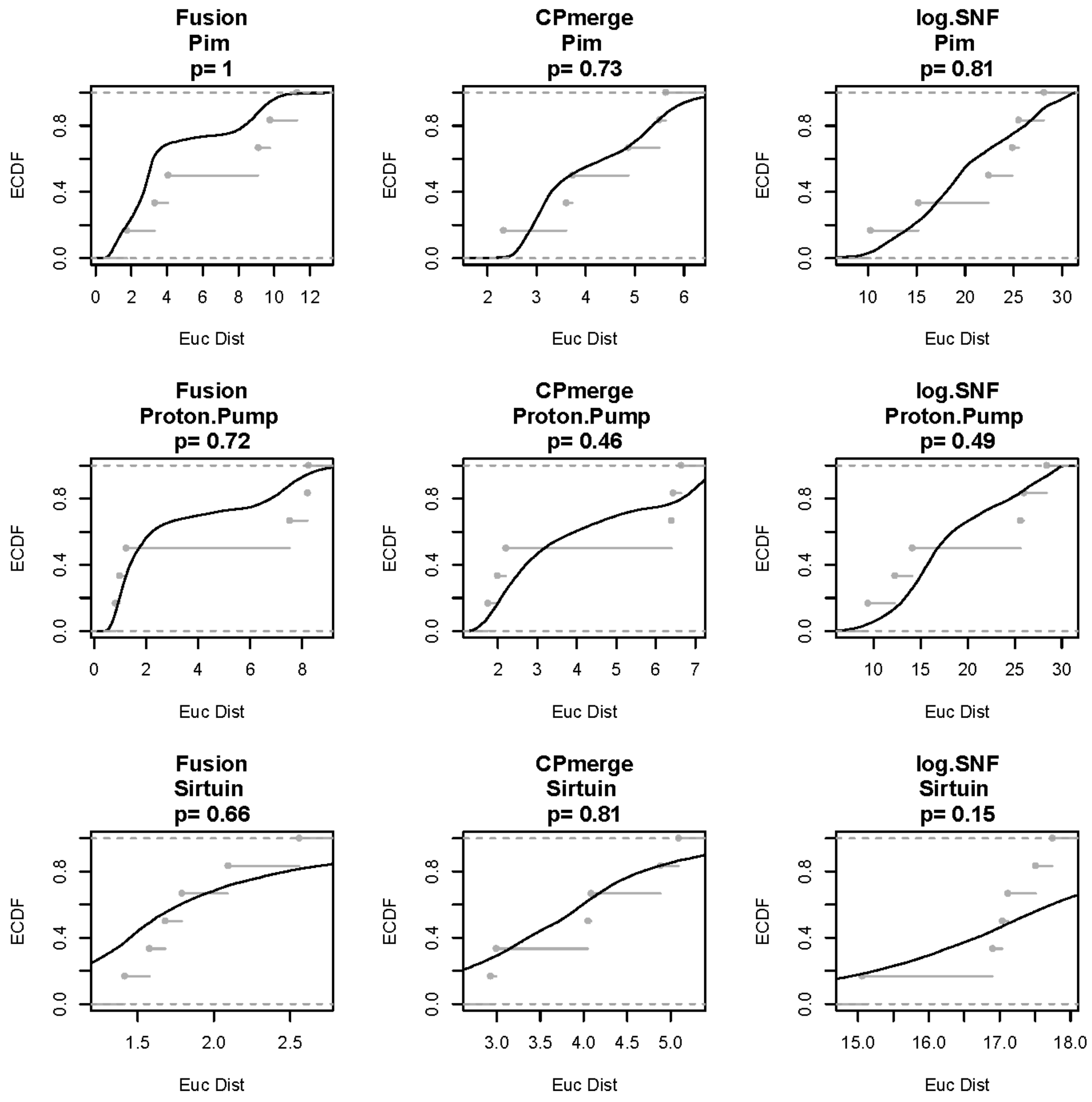
Fusion
Pim
p= 1
CPmerge
Pim
p= 0.73
log.SNF
Pim
p= 0.81
Fusion
Proton.Pump
p= 0.72
CPmerge
Proton.Pump
p= 0.46
log.SNF
Proton.Pump
p= 0.49
Fusion
Sirtuin
p= 0.66
CPmerge
Sirtuin
p= 0.81
log.SNF
Sirtuin
p= 0.15
ECDF
Euc Dist

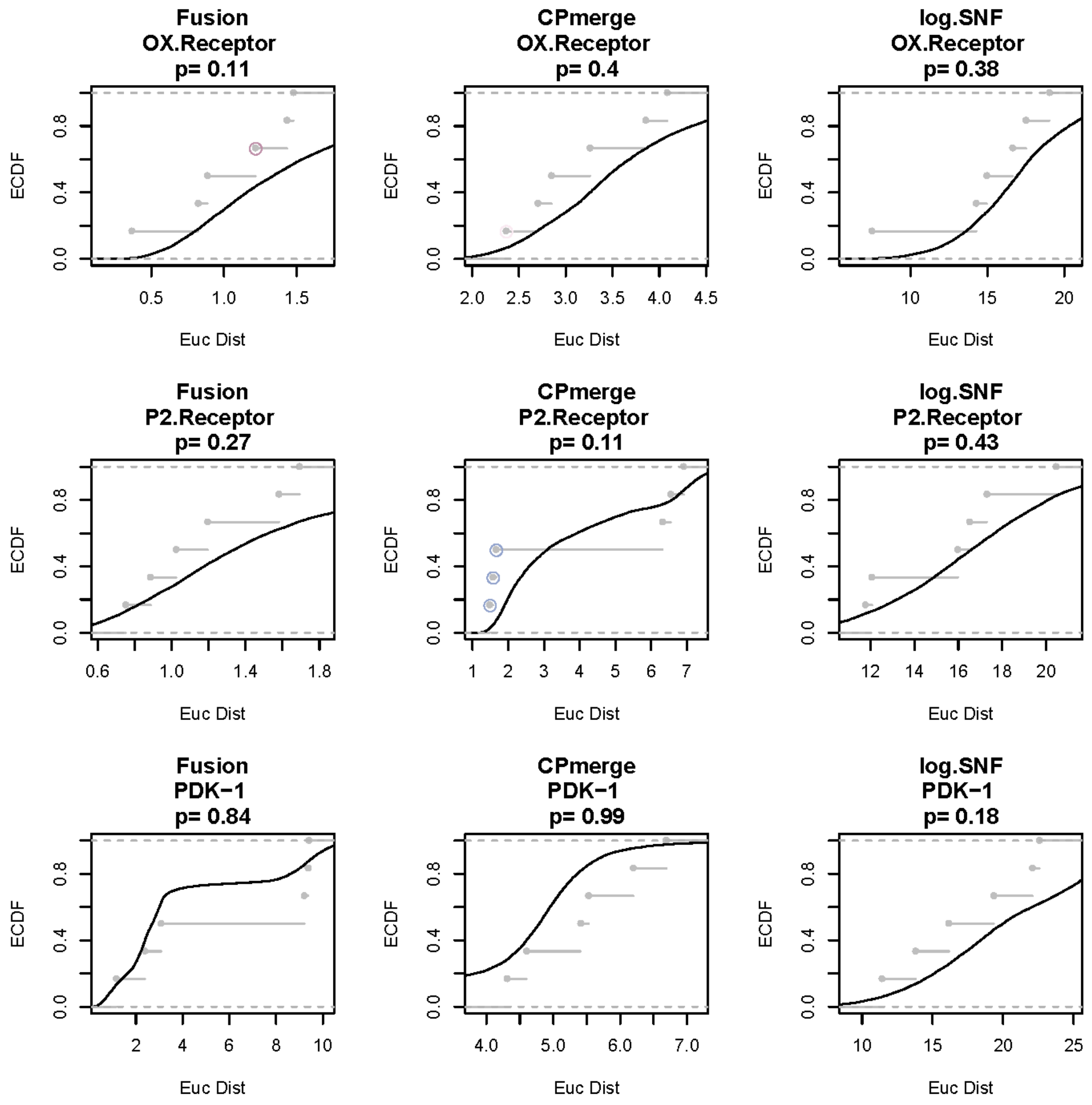
Fusion
OX.Receptor
p= 0.11
CPmerge
OX.Receptor
p= 0.4
log.SNF
OX.Receptor
p= 0.38
Fusion
P2.Receptor
p= 0.27
CPmerge
P2.Receptor
p= 0.11
log.SNF
P2.Receptor
p= 0.43
Fusion
PDK−1
p= 0.84
CPmerge
PDK−1
p= 0.99
log.SNF
PDK−1
p= 0.18
ECDF
Euc Dist

b

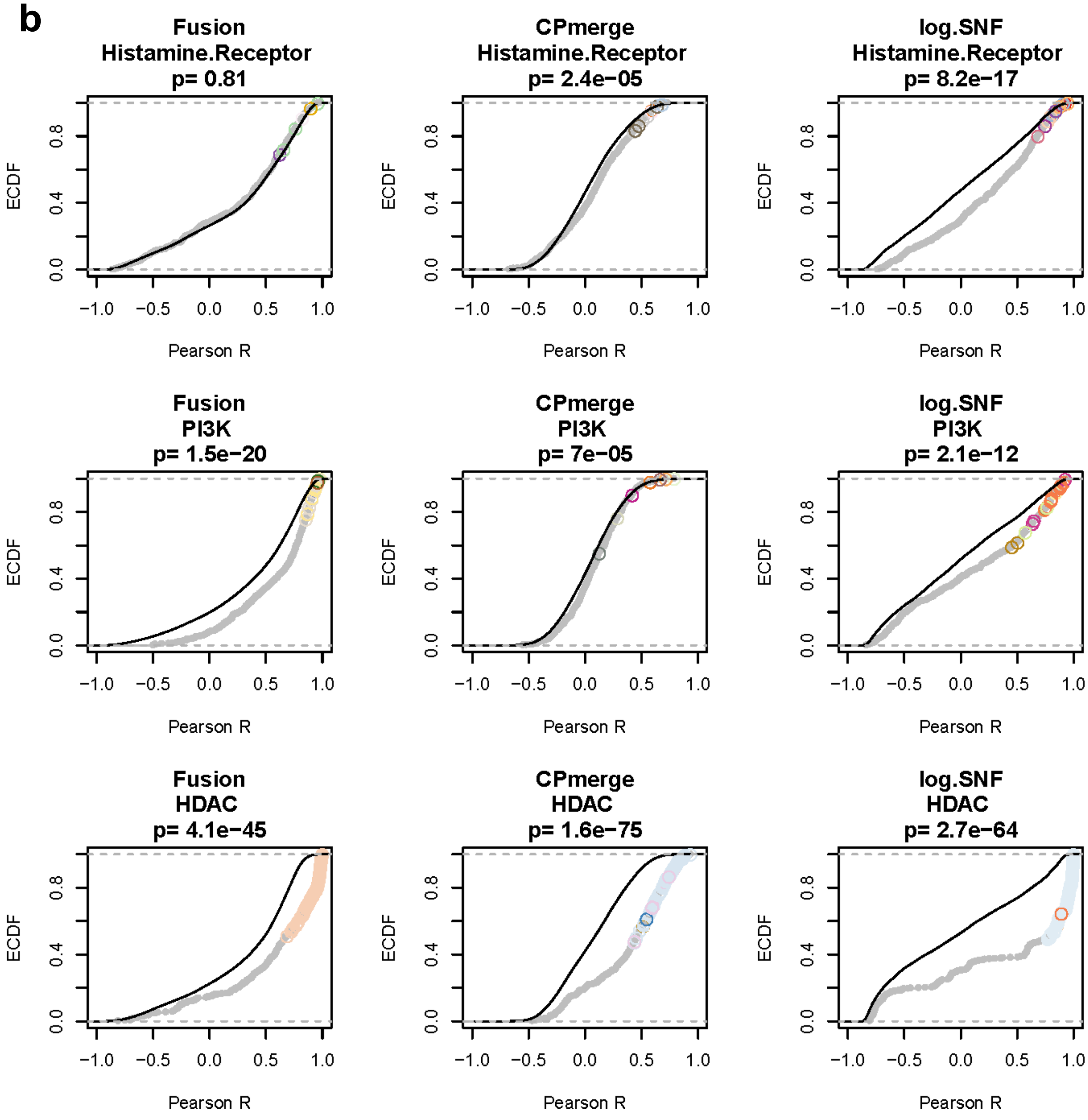
Fusion
Histamine.Receptor
p= 0.81
CPmerge
Histamine.Receptor
p= 2.4e−05
log.SNF
Histamine.Receptor
p= 8.2e−17
Fusion
PI3K
p= 1.5e−20
CPmerge
PI3K
p= 7e−05
log.SNF
PI3K
p= 2.1e−12
Fusion
HDAC
p= 4.1e−45
CPmerge
HDAC
p= 1.6e−75
log.SNF
HDAC
p= 2.7e−64
ECDF
0.0
0.4
0.8
−1.0
−0.5
0.0
0.5
1.0
Pearson R

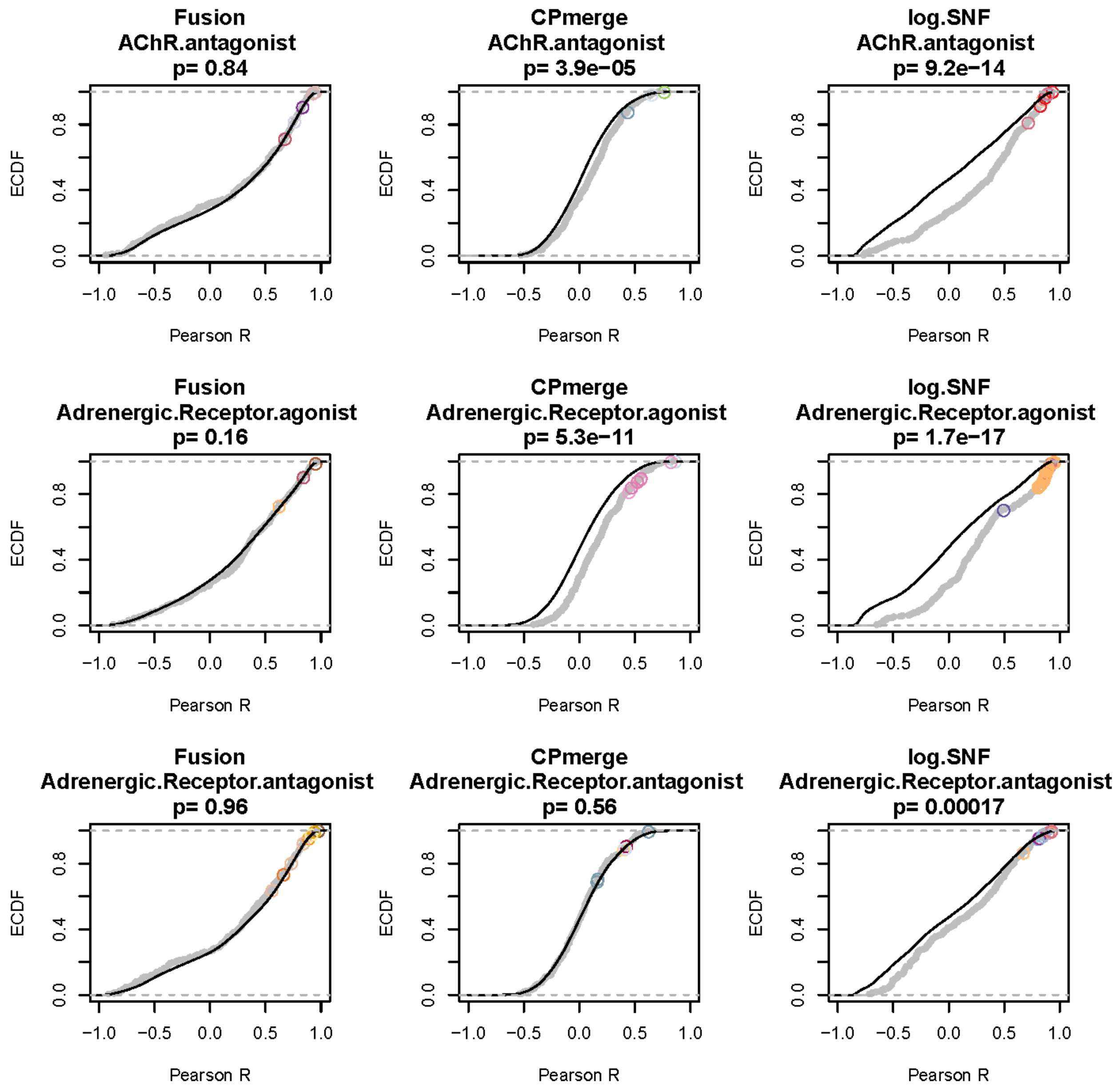

Fusion
AChR.antagonist
p= 0.84
CPmerge
AChR.antagonist
p= 3.9e−05
log.SNF
AChR.antagonist
p= 9.2e−14
Fusion
Adrenergic.Receptor.agonist
p= 0.16
CPmerge
Adrenergic.Receptor.agonist
p= 5.3e−11
log.SNF
Adrenergic.Receptor.agonist
p= 1.7e−17
Fusion
Adrenergic.Receptor.antagonist
p= 0.96
CPmerge
Adrenergic.Receptor.antagonist
p= 0.56
log.SNF
Adrenergic.Receptor.antagonist
p= 0.00017
ECDF
0.0
0.4
0.8
Pearson R
−1.0
−0.5
0.0
0.5
1.0

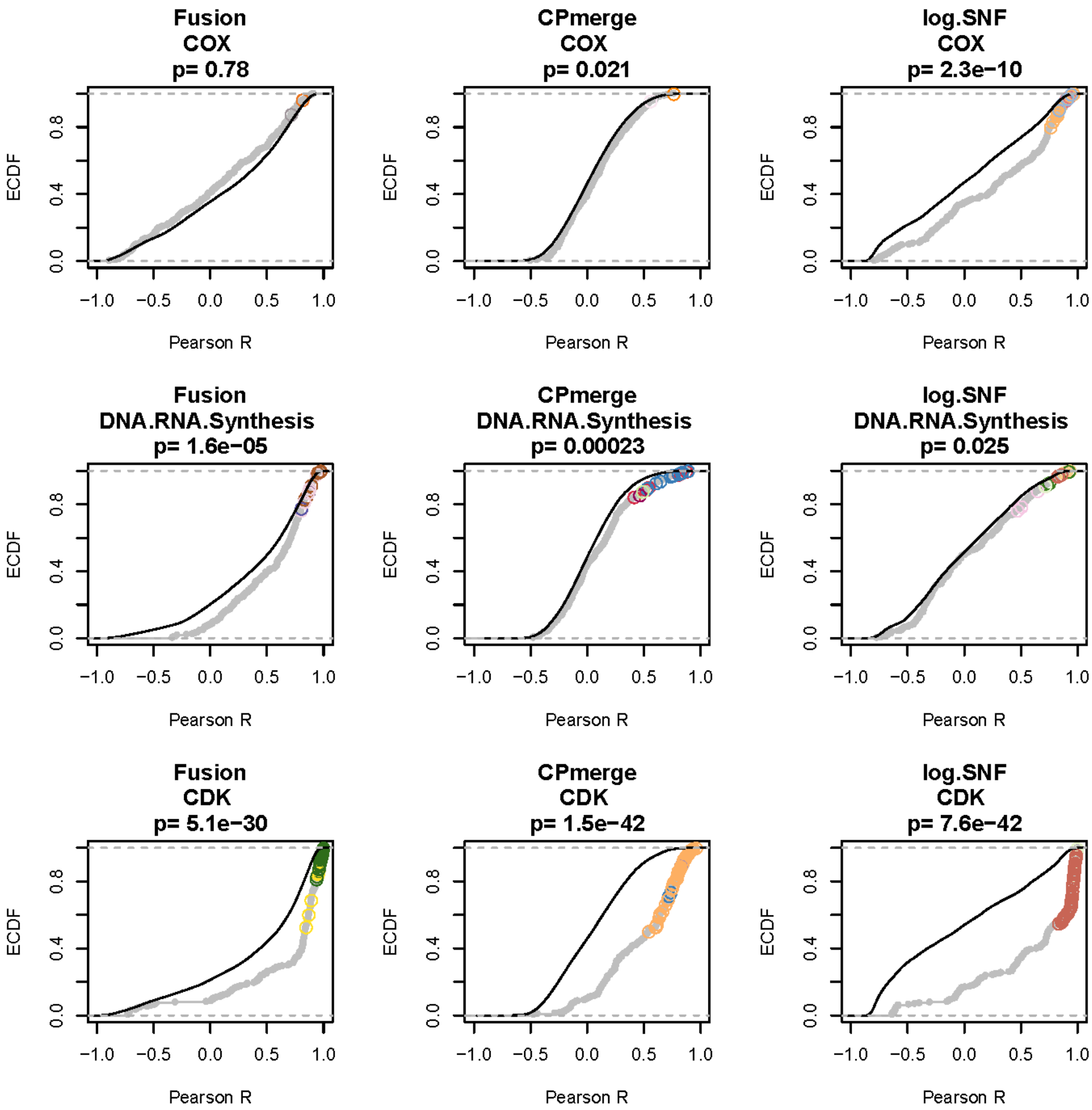
Fusion
COX
p= 0.78
CPmerge
COX
p= 0.021
log.SNF
COX
p= 2.3e−10
Fusion
DNA.RNA.Synthesis
p= 1.6e−05
CPmerge
DNA.RNA.Synthesis
p= 0.00023
log.SNF
DNA.RNA.Synthesis
p= 0.025
Fusion
CDK
p= 5.1e−30
CPmerge
CDK
p= 1.5e−42
log.SNF
CDK
p= 7.6e−42
ECDF
Pearson R
0.0
0.4
0.8
−1.0
−0.5
0.0
0.5
1.0

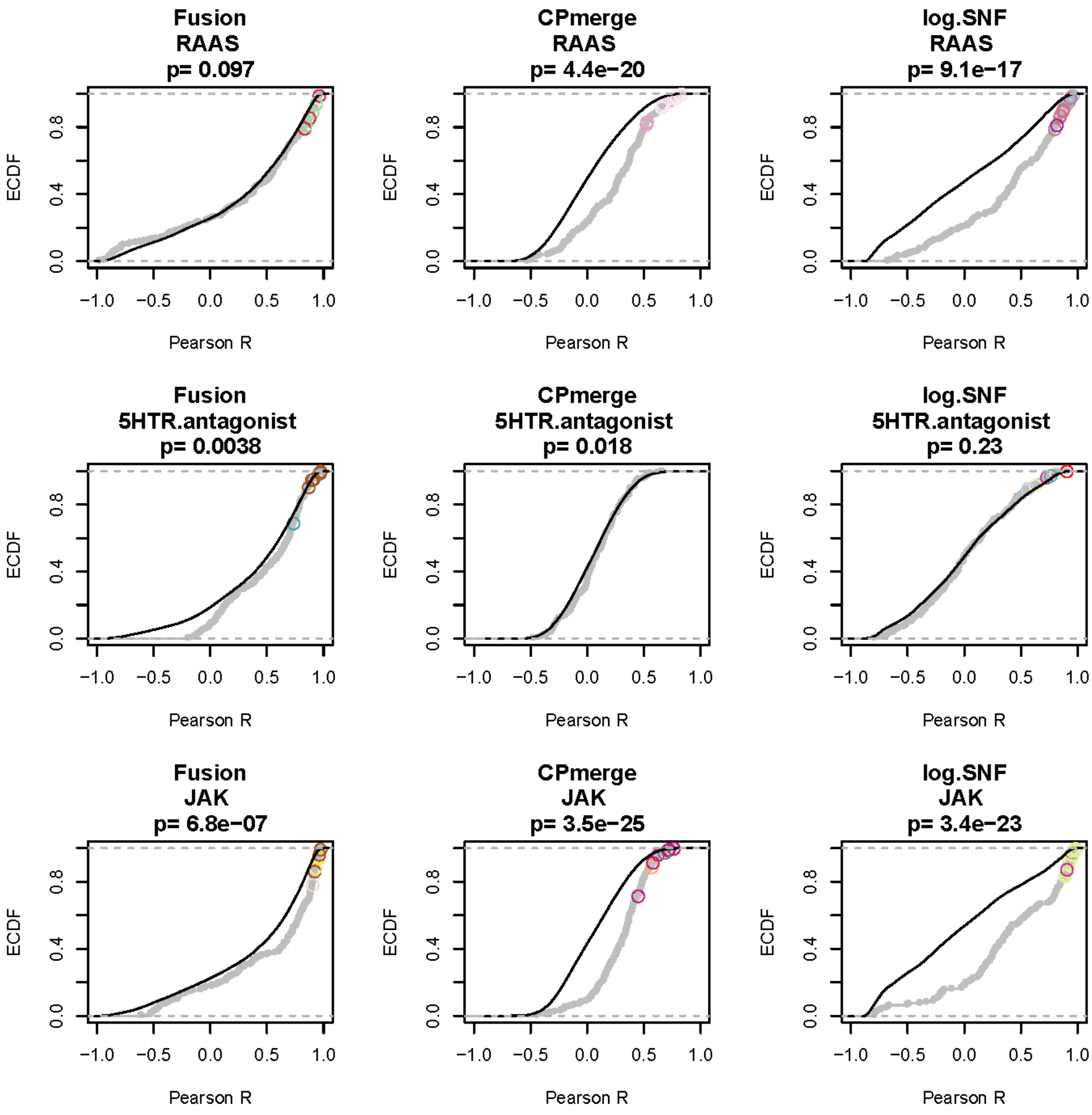
Fusion
RAAS
p= 0.097
CPmerge
RAAS
p= 4.4e−20
log.SNF
RAAS
p= 9.1e−17
Fusion
5HTR.antagonist
p= 0.0038
CPmerge
5HTR.antagonist
p= 0.018
log.SNF
5HTR.antagonist
p= 0.23
Fusion
JAK
p= 6.8e−07
CPmerge
JAK
p= 3.5e−25
log.SNF
JAK
p= 3.4e−23
ECDF
Pearson R
0.0
0.4
0.8
−1.0
−0.5
0.0
0.5
1.0

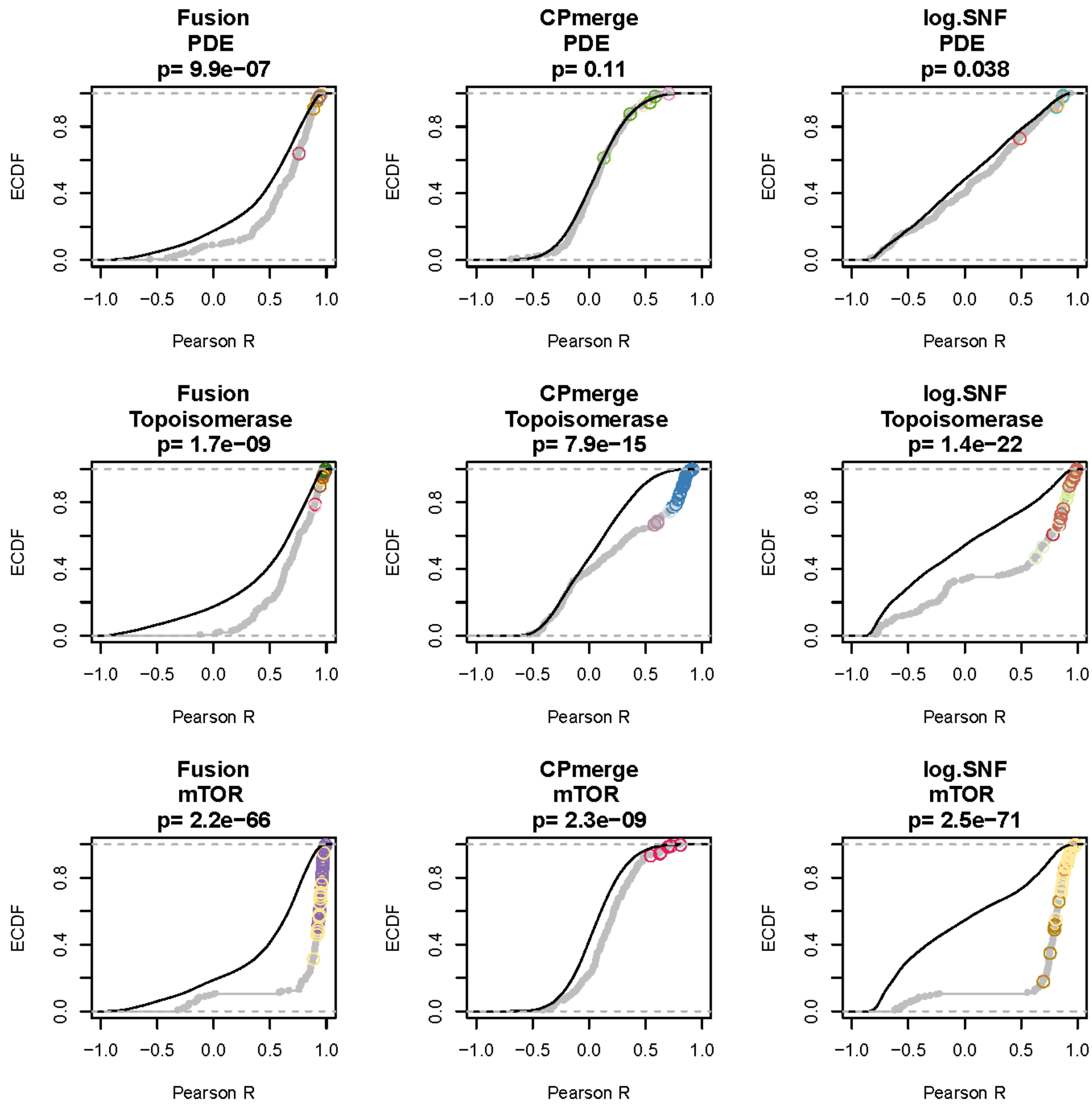
Fusion
PDE
p= 9.9e−07
CPmerge
PDE
p= 0.11
log.SNF
PDE
p= 0.038
Fusion
Topoisomerase
p= 1.7e−09
CPmerge
Topoisomerase
p= 7.9e−15
log.SNF
Topoisomerase
p= 1.4e−22
Fusion
mTOR
p= 2.2e−66
CPmerge
mTOR
p= 2.3e−09
log.SNF
mTOR
p= 2.5e−71
ECDF
Pearson R
0.0
0.4
0.8
−1.0
−0.5
0.0
0.5
1.0

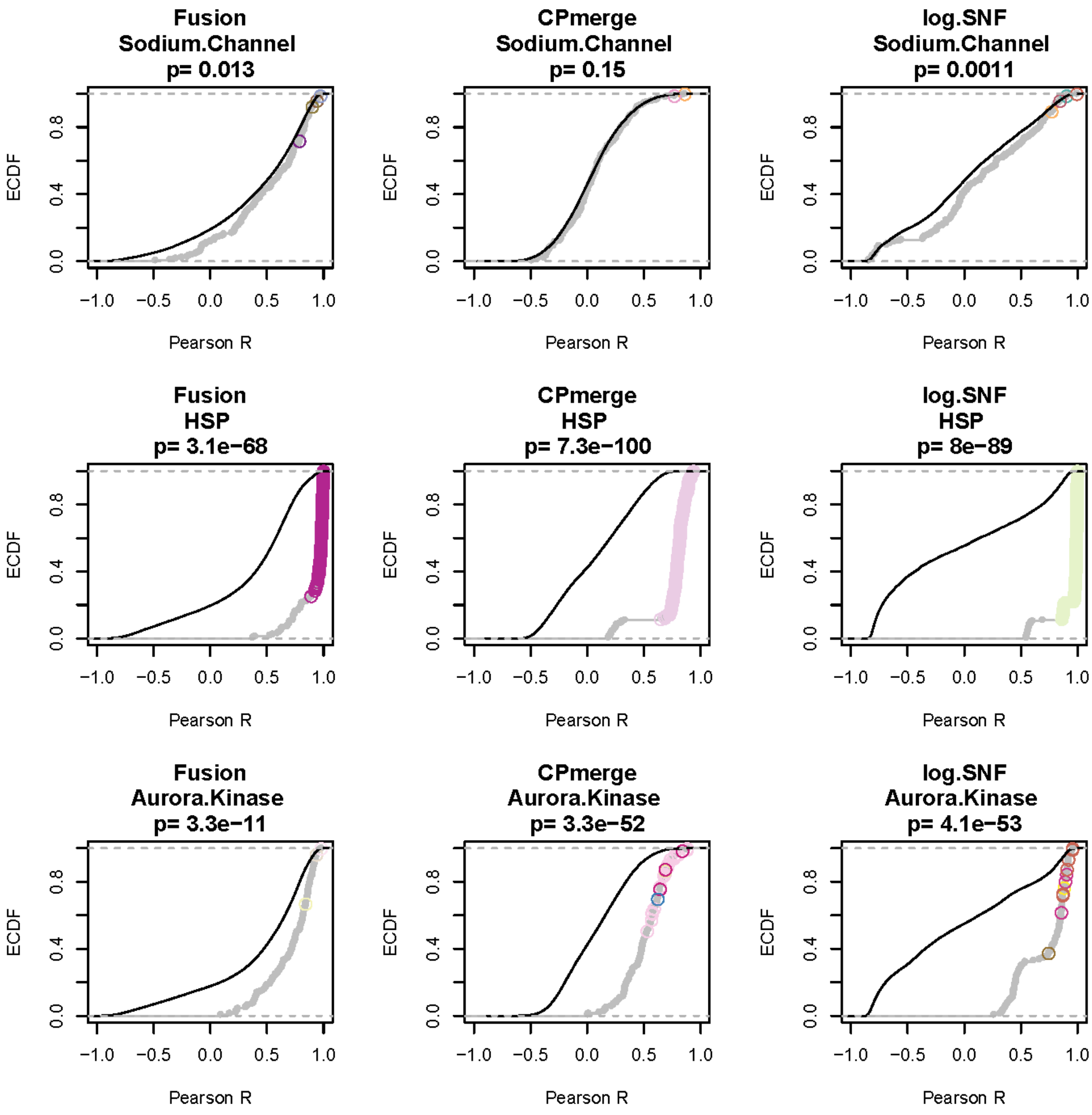
Fusion
Sodium.Channel
p= 0.013
CPmerge
Sodium.Channel
p= 0.15
log.SNF
Sodium.Channel
p= 0.0011
Fusion
HSP
p= 3.1e−68
CPmerge
HSP
p= 7.3e−100
log.SNF
HSP
p= 8e−89
Fusion
Aurora.Kinase
p= 3.3e−11
CPmerge
Aurora.Kinase
p= 3.3e−52
log.SNF
Aurora.Kinase
p= 4.1e−53
ECDF
Pearson R
0.0
0.4
0.8
−1.0
−0.5
0.0
0.5
1.0

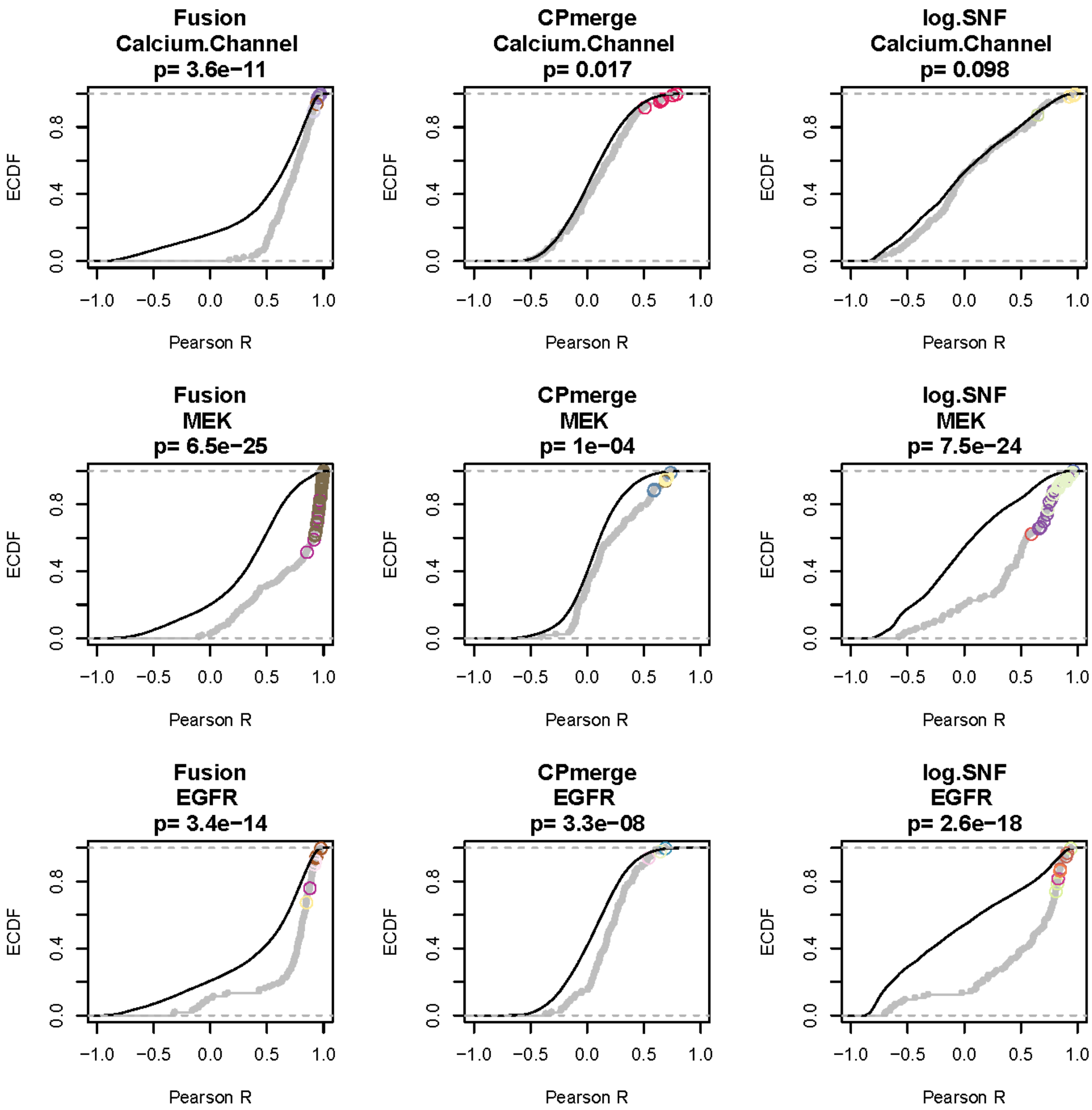
Fusion
Calcium.Channel
p= 3.6e−11
CPmerge
Calcium.Channel
p= 0.017
log.SNF
Calcium.Channel
p= 0.098
Fusion
MEK
p= 6.5e−25
CPmerge
MEK
p= 1e−04
log.SNF
MEK
p= 7.5e−24
Fusion
EGFR
p= 3.4e−14
CPmerge
EGFR
p= 3.3e−08
log.SNF
EGFR
p= 2.6e−18
ECDF
Pearson R
0.0
0.4
0.8
−1.0
−0.5
0.0
0.5
1.0

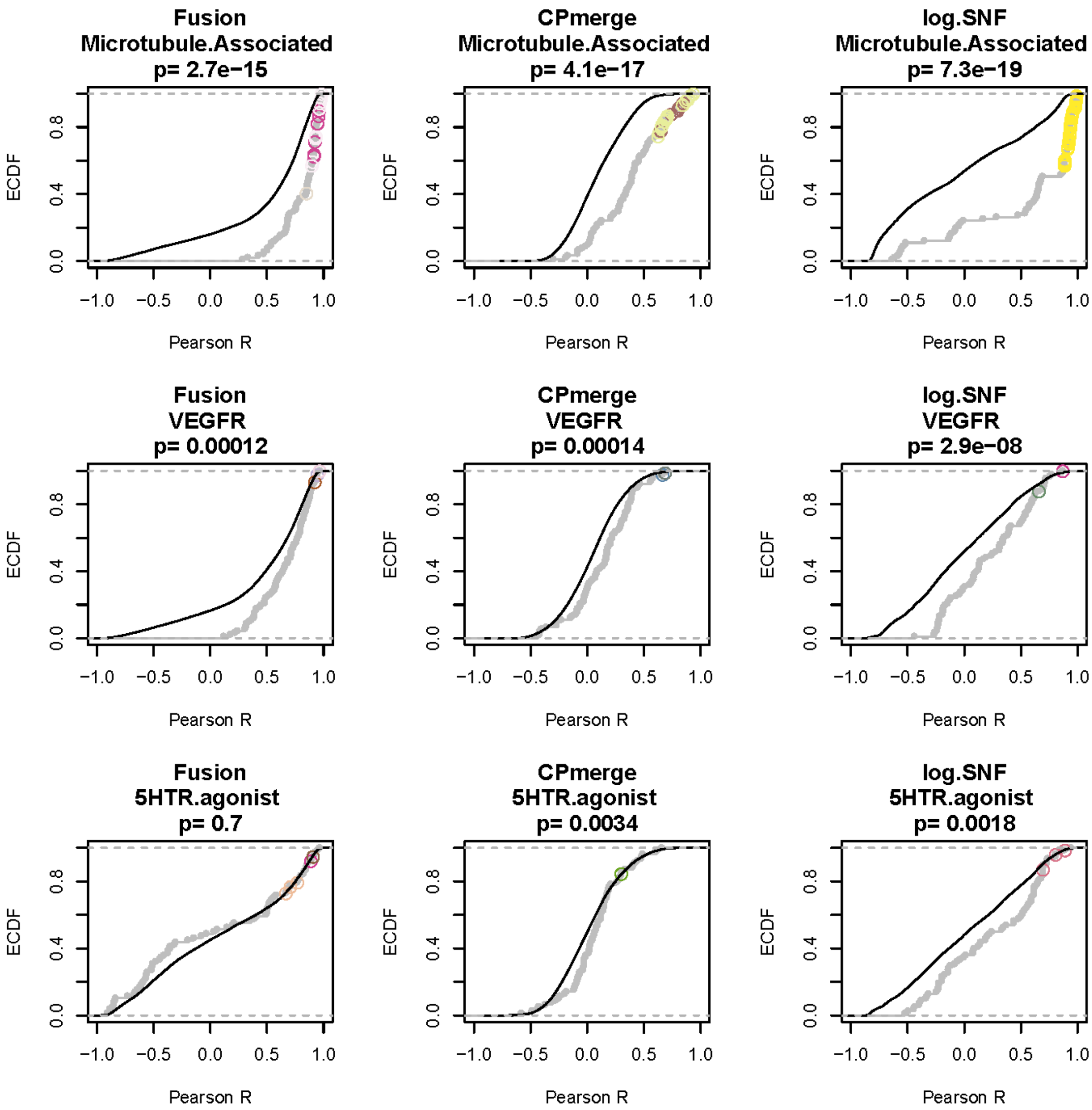
Fusion
Microtubule.Associated
p= 2.7e−15
CPmerge
Microtubule.Associated
p= 4.1e−17
log.SNF
Microtubule.Associated
p= 7.3e−19
Fusion
VEGFR
p= 0.00012
CPmerge
VEGFR
p= 0.00014
log.SNF
VEGFR
p= 2.9e−08
Fusion
5HTR.agonist
p= 0.7
CPmerge
5HTR.agonist
p= 0.0034
log.SNF
5HTR.agonist
p= 0.0018
ECDF
0.0
0.4
0.8
−1.0
−0.5
0.0
0.5
1.0
Pearson R

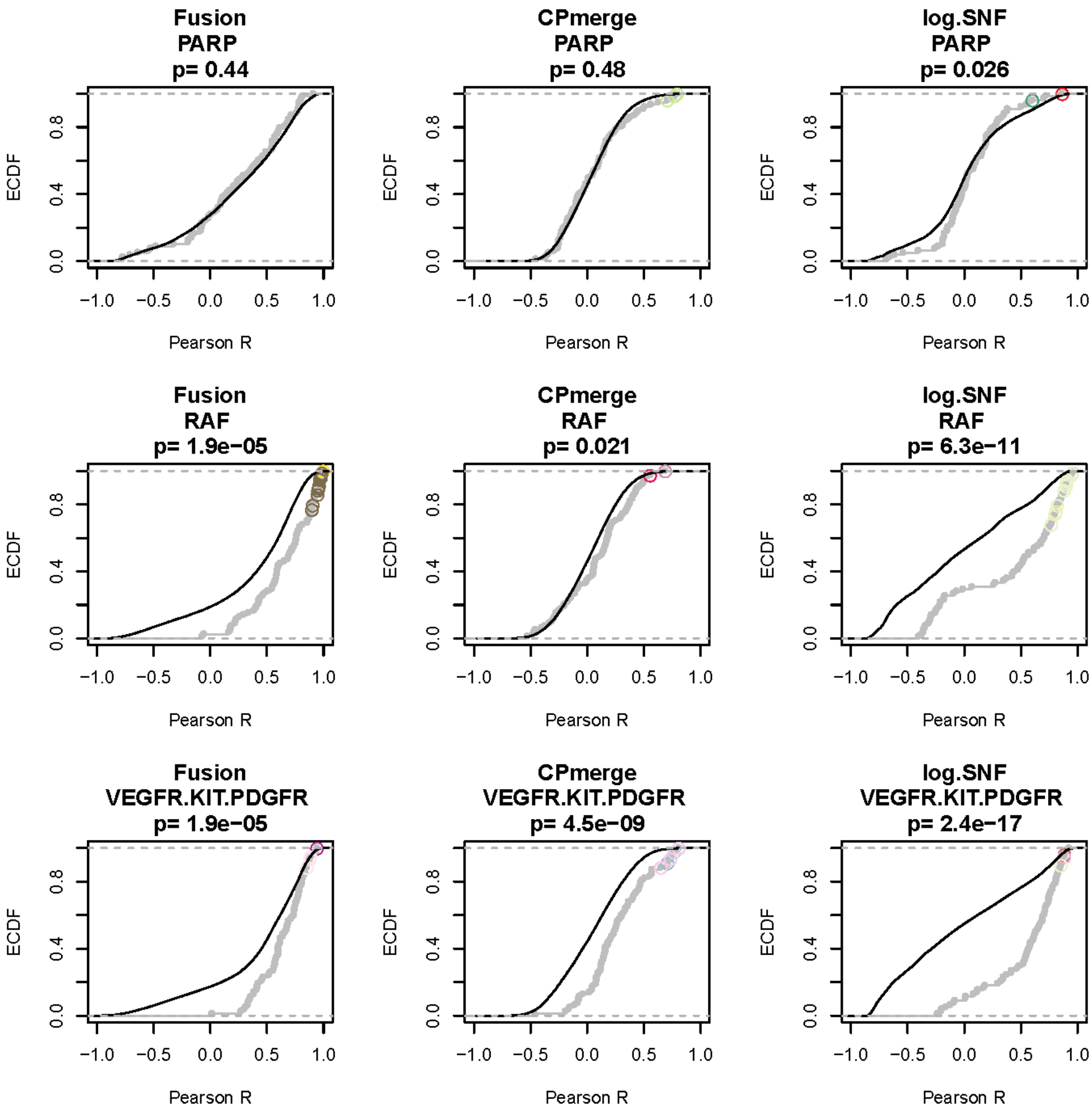
Fusion
PARP
p= 0.44
CPmerge
PARP
p= 0.48
log.SNF
PARP
p= 0.026
Fusion
RAF
p= 1.9e−05
CPmerge
RAF
p= 0.021
log.SNF
RAF
p= 6.3e−11
Fusion
VEGFR.KIT.PDGFR
p= 1.9e−05
CPmerge
VEGFR.KIT.PDGFR
p= 4.5e−09
log.SNF
VEGFR.KIT.PDGFR
p= 2.4e−17
ECDF
0.0
0.4
0.8
−1.0
−0.5
0.0
0.5
1.0
Pearson R

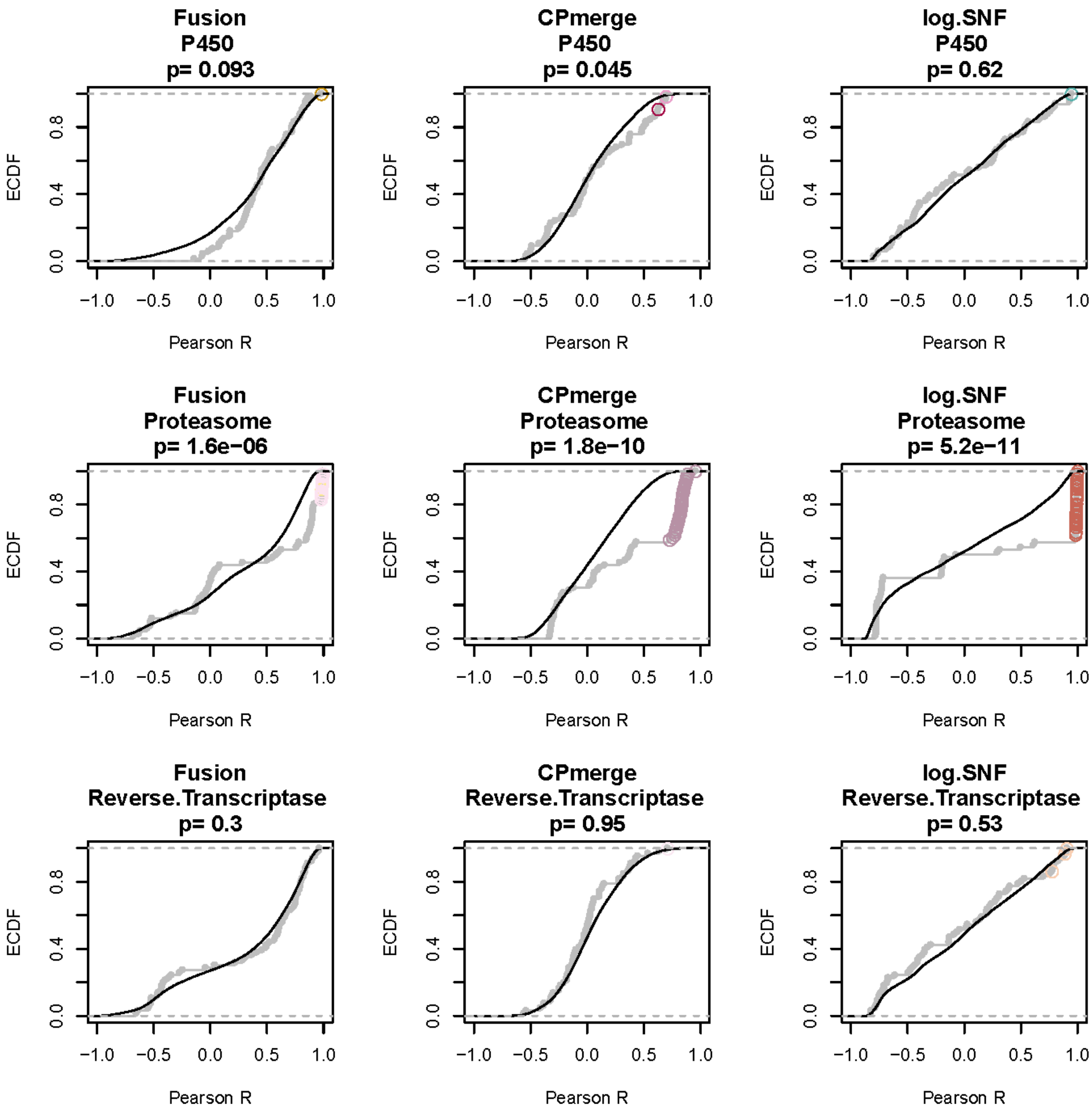
Fusion
P450
p= 0.093
CPmerge
P450
p= 0.045
log.SNF
P450
p= 0.62
Fusion
Proteasome
p= 1.6e−06
CPmerge
Proteasome
p= 1.8e−10
log.SNF
Proteasome
p= 5.2e−11
Fusion
Reverse.Transcriptase
p= 0.3
CPmerge
Reverse.Transcriptase
p= 0.95
log.SNF
Reverse.Transcriptase
p= 0.53
ECDF
0.0
0.4
0.8
−1.0
−0.5
0.0
0.5
1.0
Pearson R

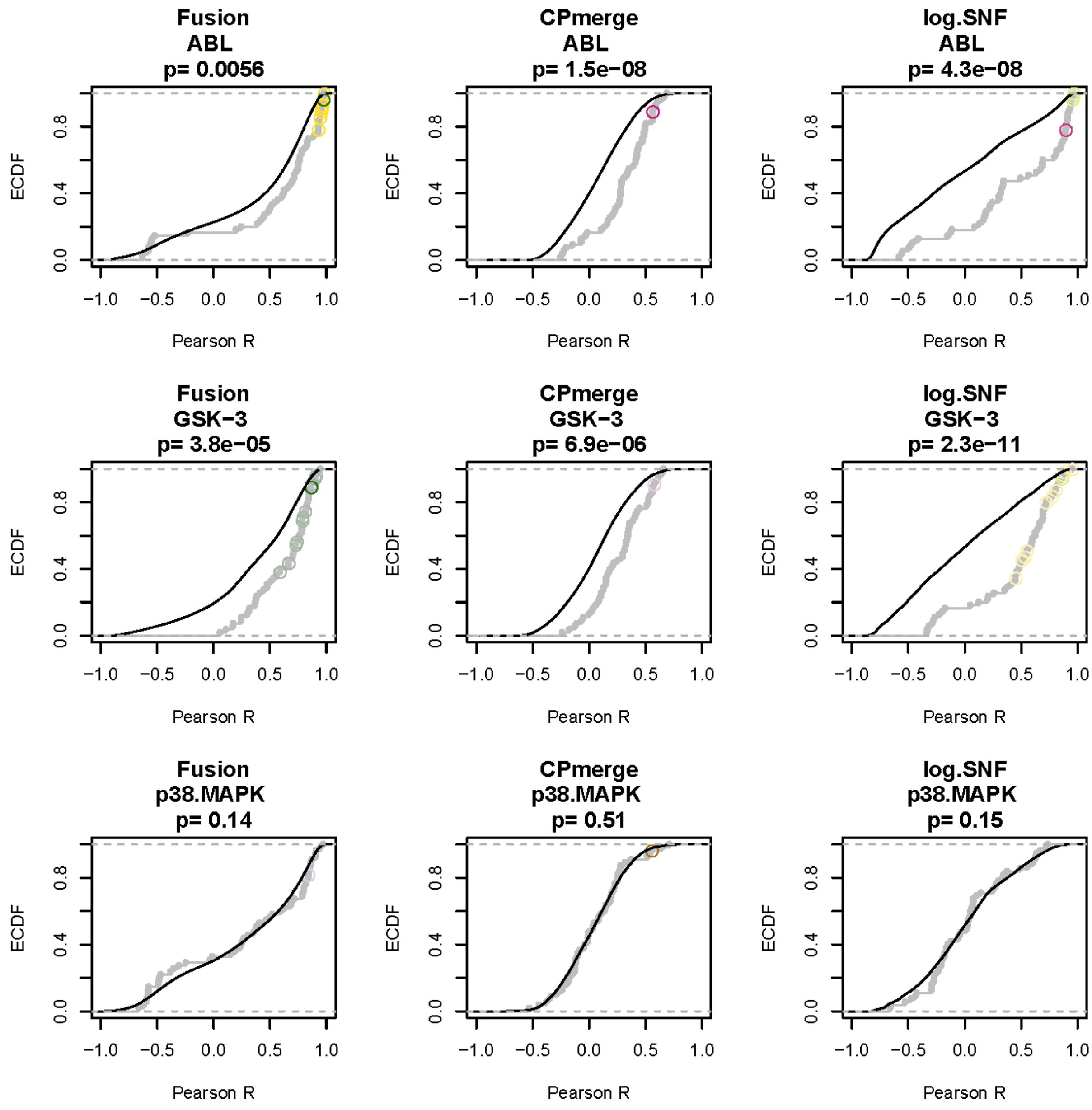
Fusion
ABL
p= 0.0056
CPmerge
ABL
p= 1.5e−08
log.SNF
ABL
p= 4.3e−08
Fusion
GSK−3
p= 3.8e−05
CPmerge
GSK−3
p= 6.9e−06
log.SNF
GSK−3
p= 2.3e−11
Fusion
p38.MAPK
p= 0.14
CPmerge
p38.MAPK
p= 0.51
log.SNF
p38.MAPK
p= 0.15
ECDF
0.0
0.4
0.8
−1.0
−0.5
0.0
0.5
1.0
Pearson R

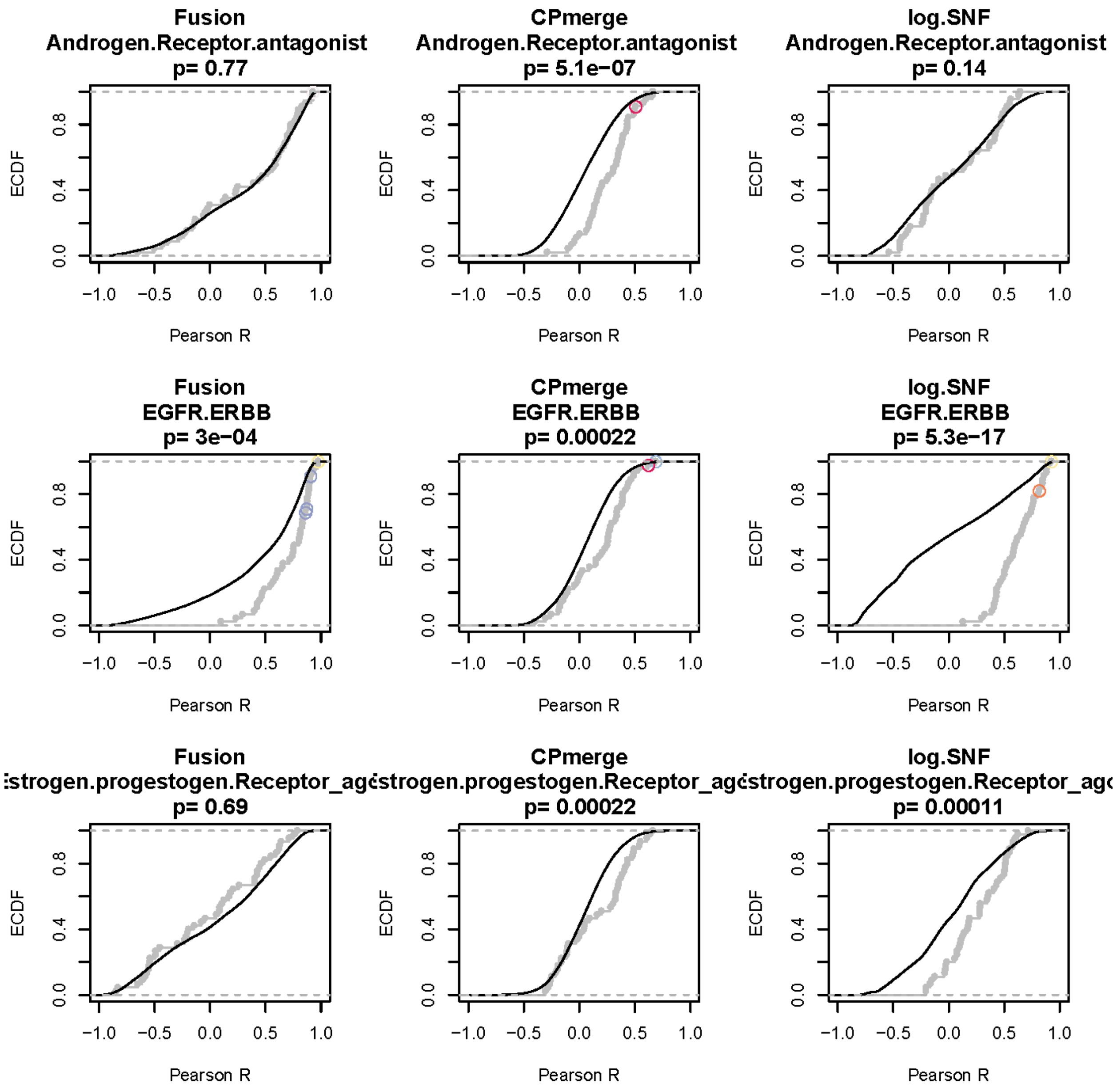
Fusion
Androgen.Receptor.antagonist
p= 0.77
CPmerge
Androgen.Receptor.antagonist
p= 5.1e−07
log.SNF
Androgen.Receptor.antagonist
p= 0.14
Fusion
EGFR.ERBB
p= 3e−04
CPmerge
EGFR.ERBB
p= 0.00022
log.SNF
EGFR.ERBB
p= 5.3e−17
Fusion
strogen.progestogen.Receptor_ag
p= 0.69
CPmerge
strogen.progestogen.Receptor_ag
p= 0.00022
log.SNF
strogen.progestogen.Receptor_ag
p= 0.00011
ECDF
Pearson R
0.0
0.4
0.8
−1.0
−0.5
0.0
0.5
1.0

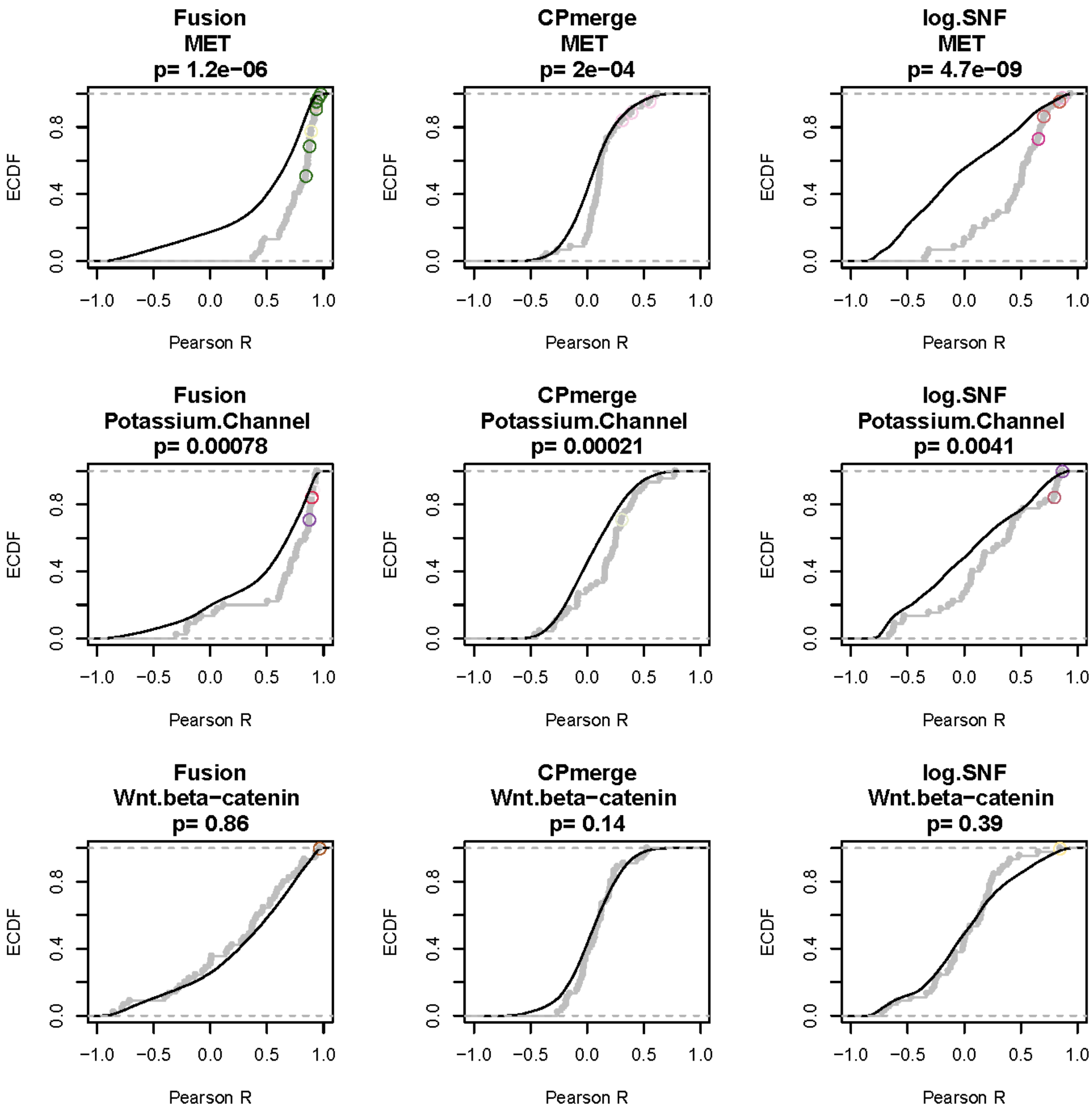

Fusion
MET
p= 1.2e−06
CPmerge
MET
p= 2e−04
log.SNF
MET
p= 4.7e−09
Fusion
Potassium.Channel
p= 0.00078
CPmerge
Potassium.Channel
p= 0.00021
log.SNF
Potassium.Channel
p= 0.0041
Fusion
Wnt.beta−catenin
p= 0.86
CPmerge
Wnt.beta−catenin
p= 0.14
log.SNF
Wnt.beta−catenin
p= 0.39
ECDF
0.0
0.4
0.8
−1.0
−0.5
0.0
0.5
1.0
Pearson R

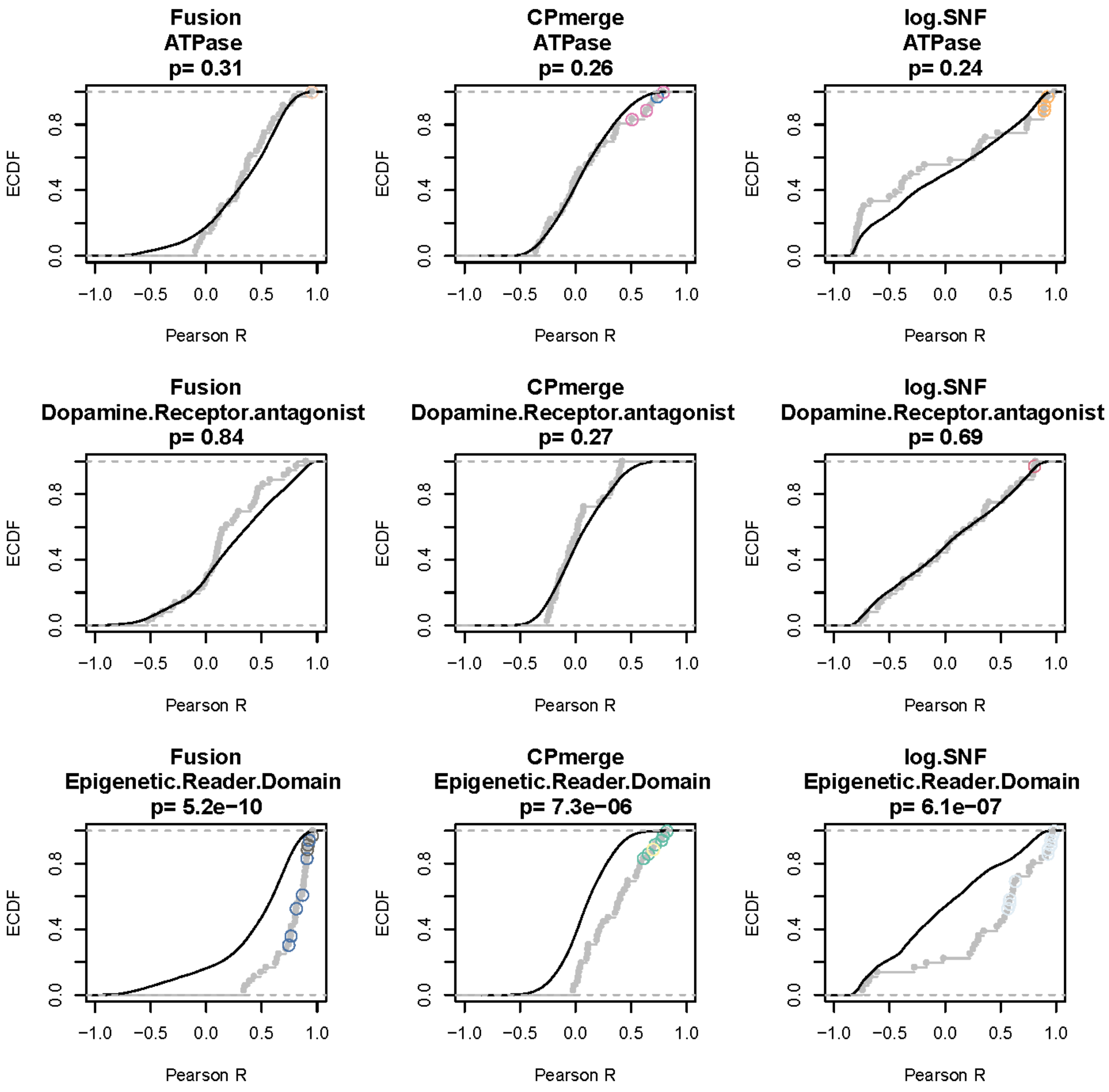
Fusion
ATPase
p= 0.31
CPmerge
ATPase
p= 0.26
log.SNF
ATPase
p= 0.24
Fusion
Dopamine.Receptor.antagonist
p= 0.84
CPmerge
Dopamine.Receptor.antagonist
p= 0.27
log.SNF
Dopamine.Receptor.antagonist
p= 0.69
Fusion
Epigenetic.Reader.Domain
p= 5.2e−10
CPmerge
Epigenetic.Reader.Domain
p= 7.3e−06
log.SNF
Epigenetic.Reader.Domain
p= 6.1e−07
ECDF
0.0
0.4
0.8
−1.0
−0.5
0.0
0.5
1.0
Pearson R

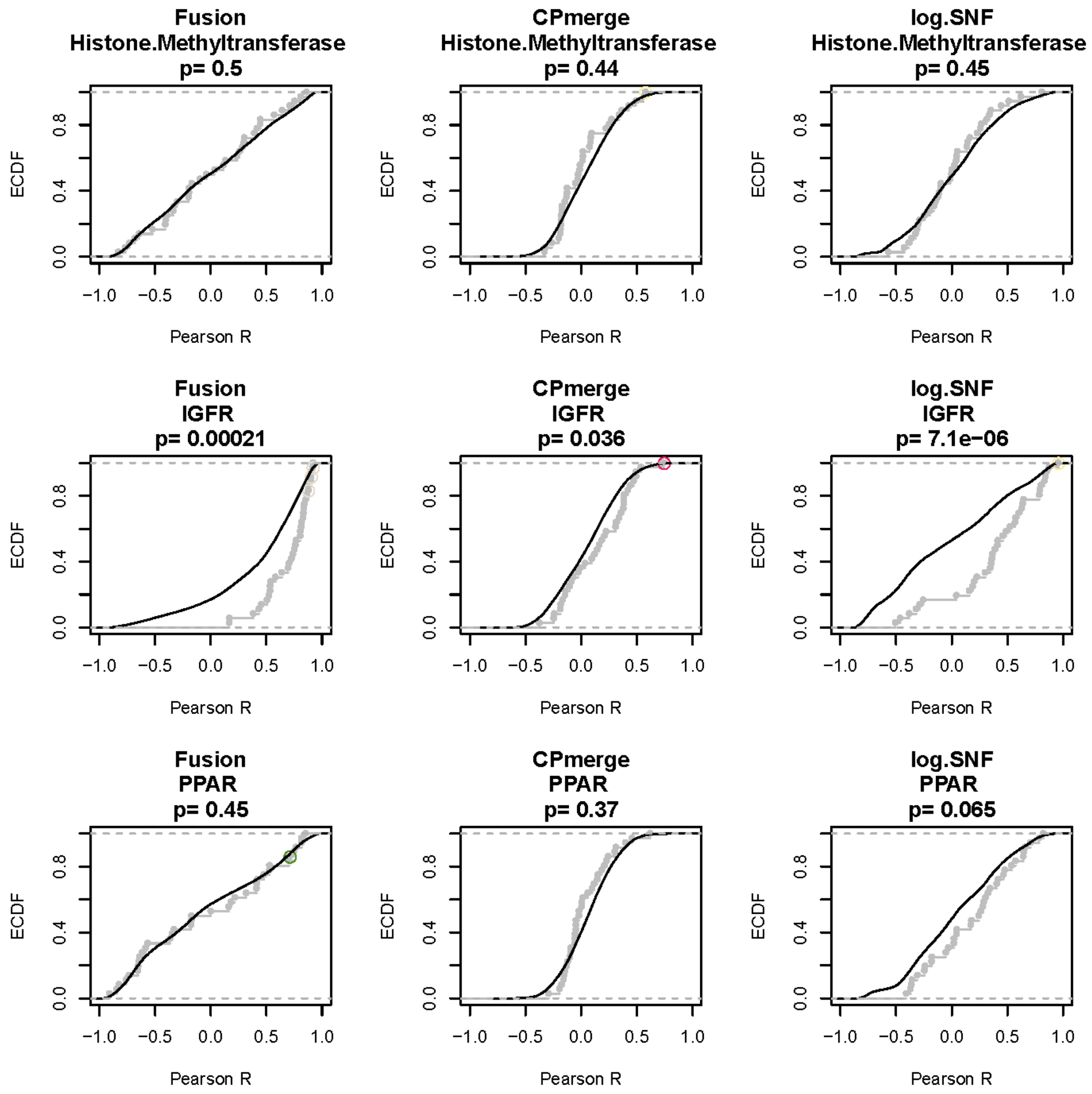
Fusion
Histone.Methyltransferase
p= 0.5
CPmerge
Histone.Methyltransferase
p= 0.44
log.SNF
Histone.Methyltransferase
p= 0.45
Fusion
IGFR
p= 0.00021
CPmerge
IGFR
p= 0.036
log.SNF
IGFR
p= 7.1e−06
Fusion
PPAR
p= 0.45
CPmerge
PPAR
p= 0.37
log.SNF
PPAR
p= 0.065
ECDF
0.0
0.4
0.8
−1.0
−0.5
0.0
0.5
1.0
Pearson R

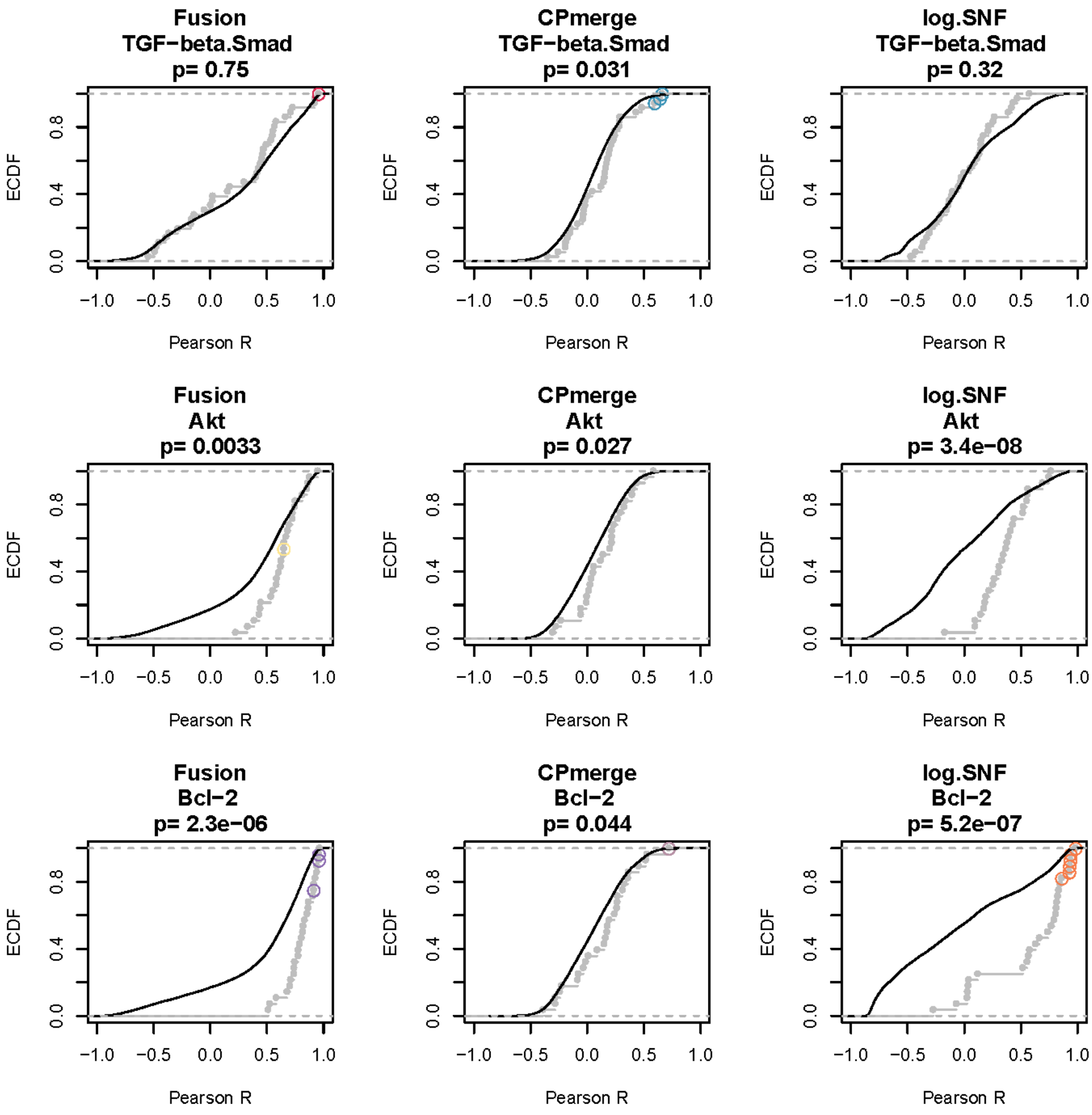
Fusion
TGF−beta.Smad
p= 0.75
CPmerge
TGF−beta.Smad
p= 0.031
log.SNF
TGF−beta.Smad
p= 0.32
Fusion
Akt
p= 0.0033
CPmerge
Akt
p= 0.027
log.SNF
Akt
p= 3.4e−08
Fusion
Bcl−2
p= 2.3e−06
CPmerge
Bcl−2
p= 0.044
log.SNF
Bcl−2
p= 5.2e−07
ECDF
Pearson R
0.0
0.4
0.8
−1.0
−0.5
0.0
0.5
1.0

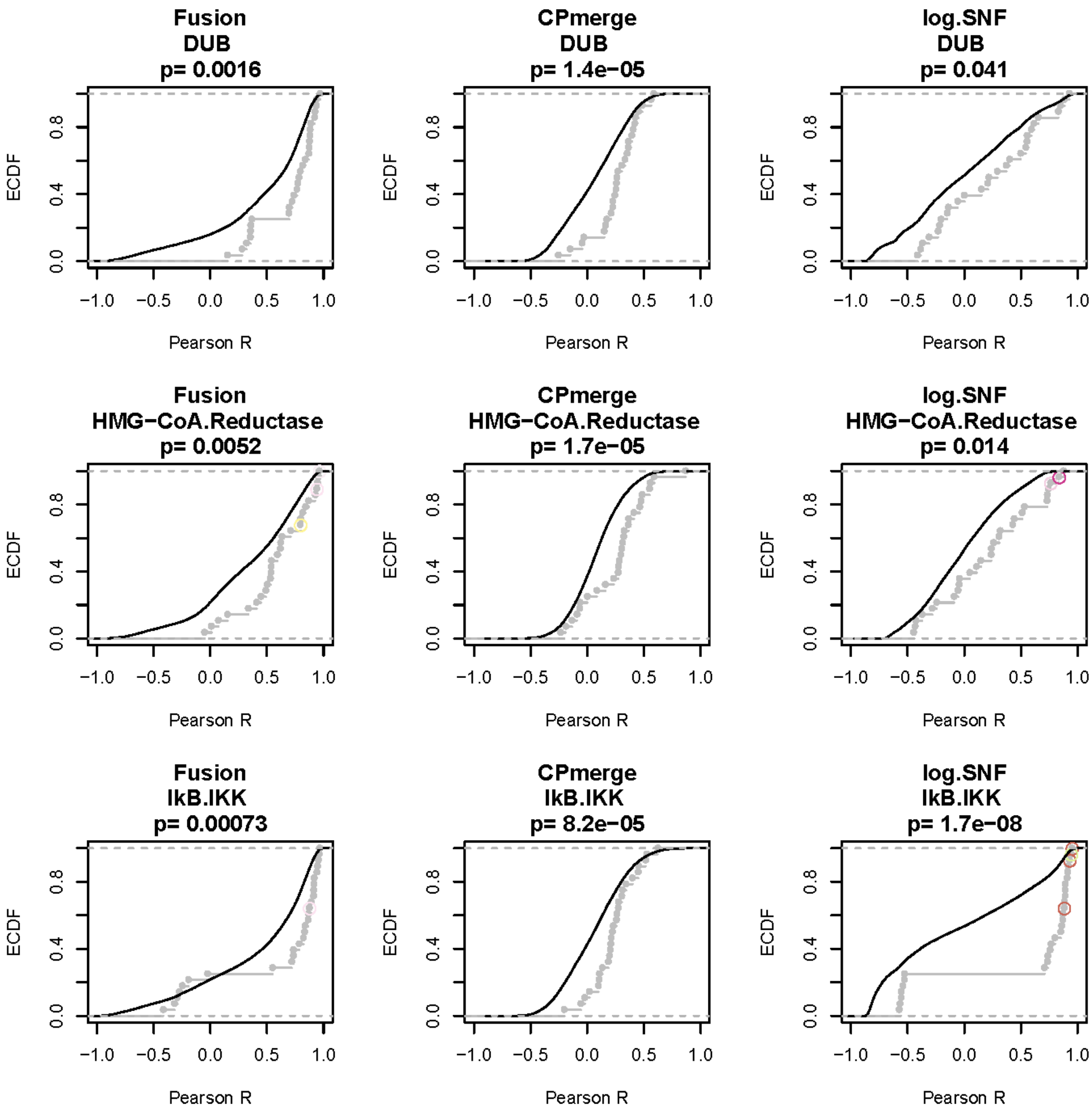

Fusion
DUB
p= 0.0016
CPmerge
DUB
p= 1.4e−05
log.SNF
DUB
p= 0.041
Fusion
HMG−CoA.Reductase
p= 0.0052
CPmerge
HMG−CoA.Reductase
p= 1.7e−05
log.SNF
HMG−CoA.Reductase
p= 0.014
Fusion
IkB.IKK
p= 0.00073
CPmerge
IkB.IKK
p= 8.2e−05
log.SNF
IkB.IKK
p= 1.7e−08
ECDF
Pearson R
0.0
0.4
0.8
−1.0
−0.5
0.0
0.5
1.0

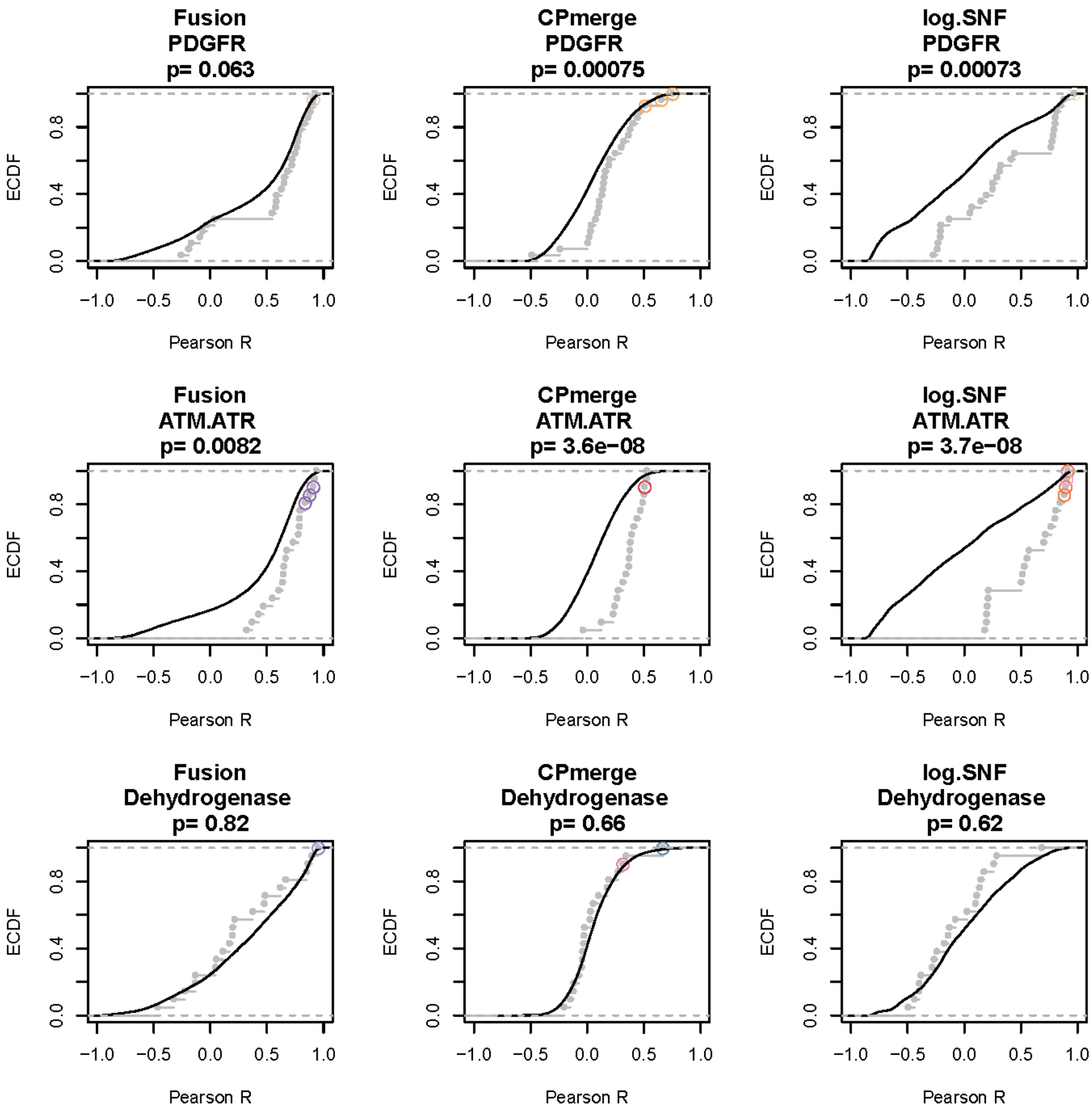
Fusion
PDGFR
p= 0.063
CPmerge
PDGFR
p= 0.00075
log.SNF
PDGFR
p= 0.00073
Fusion
ATM.ATR
p= 0.0082
CPmerge
ATM.ATR
p= 3.6e−08
log.SNF
ATM.ATR
p= 3.7e−08
Fusion
Dehydrogenase
p= 0.82
CPmerge
Dehydrogenase
p= 0.66
log.SNF
Dehydrogenase
p= 0.62
ECDF
Pearson R
0.0
0.4
0.8
−1.0
−0.5
0.0
0.5
1.0

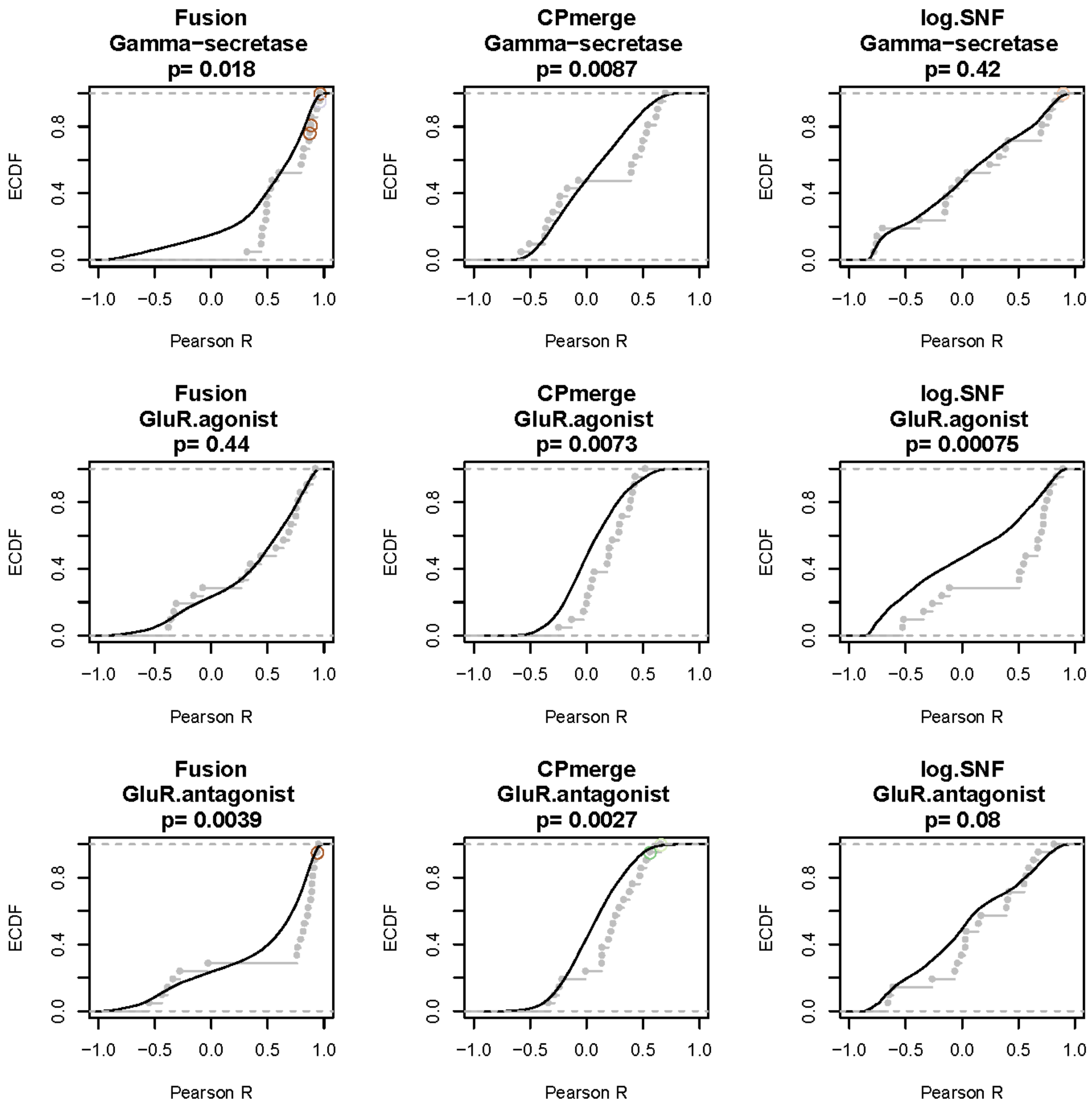
Fusion
Gamma−secretase
p= 0.018
CPmerge
Gamma−secretase
p= 0.0087
log.SNF
Gamma−secretase
p= 0.42
Fusion
GluR.agonist
p= 0.44
CPmerge
GluR.agonist
p= 0.0073
log.SNF
GluR.agonist
p= 0.00075
Fusion
GluR.antagonist
p= 0.0039
CPmerge
GluR.antagonist
p= 0.0027
log.SNF
GluR.antagonist
p= 0.08
ECDF
0.0
0.4
0.8
Pearson R
−1.0
−0.5
0.0
0.5
1.0

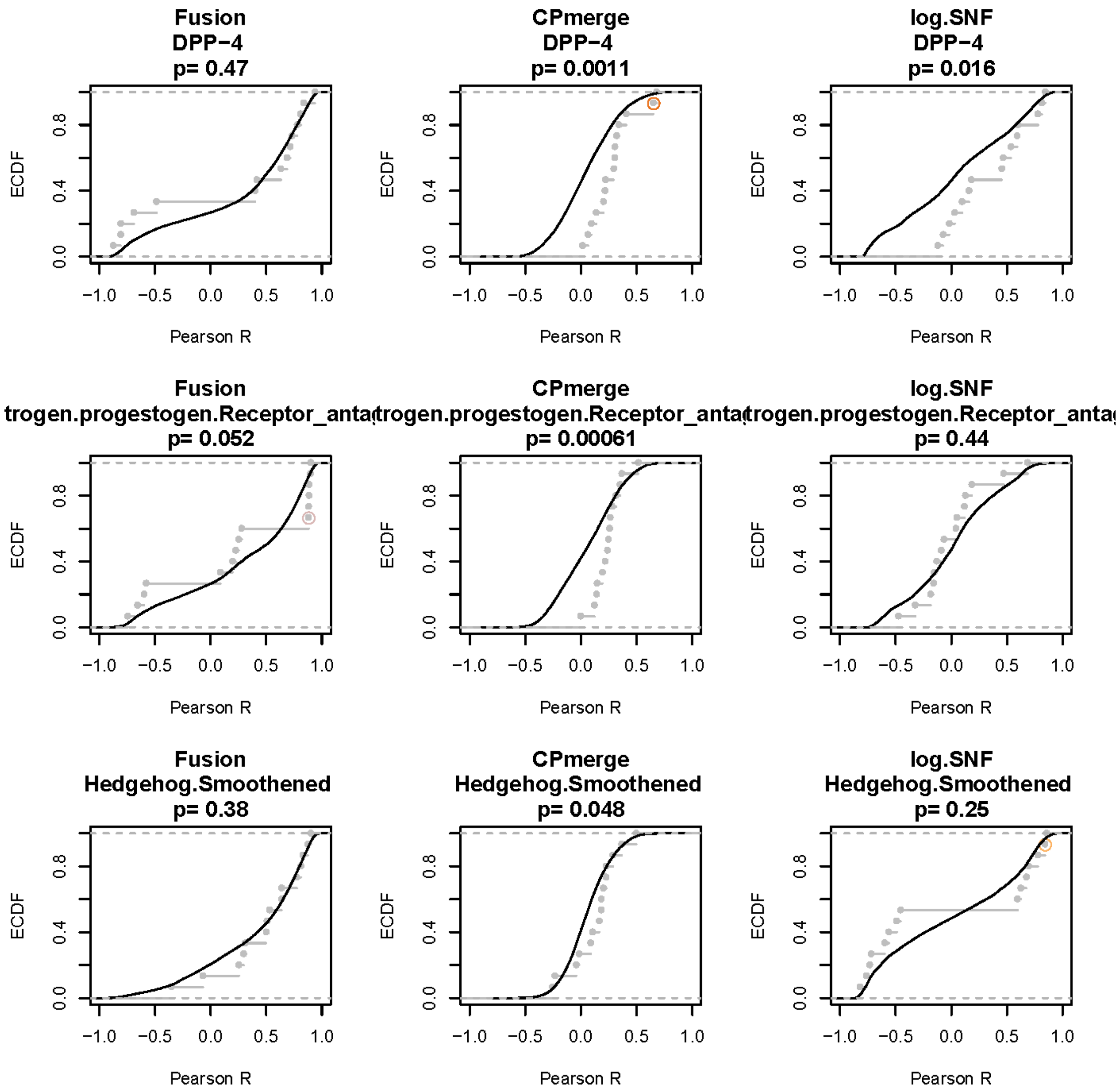
Fusion
DPP−4
p= 0.47
CPmerge
DPP−4
p= 0.0011
log.SNF
DPP−4
p= 0.016
Fusion
trogen.progestogen.Receptor_anta
p= 0.052
CPmerge
trogen.progestogen.Receptor_anta
p= 0.00061
log.SNF
trogen.progestogen.Receptor_anta
p= 0.44
Fusion
Hedgehog.Smoothened
p= 0.38
CPmerge
Hedgehog.Smoothened
p= 0.048
log.SNF
Hedgehog.Smoothened
p= 0.25
ECDF
0.0
0.4
0.8
Pearson R
−1.0
−0.5
0.0
0.5
1.0

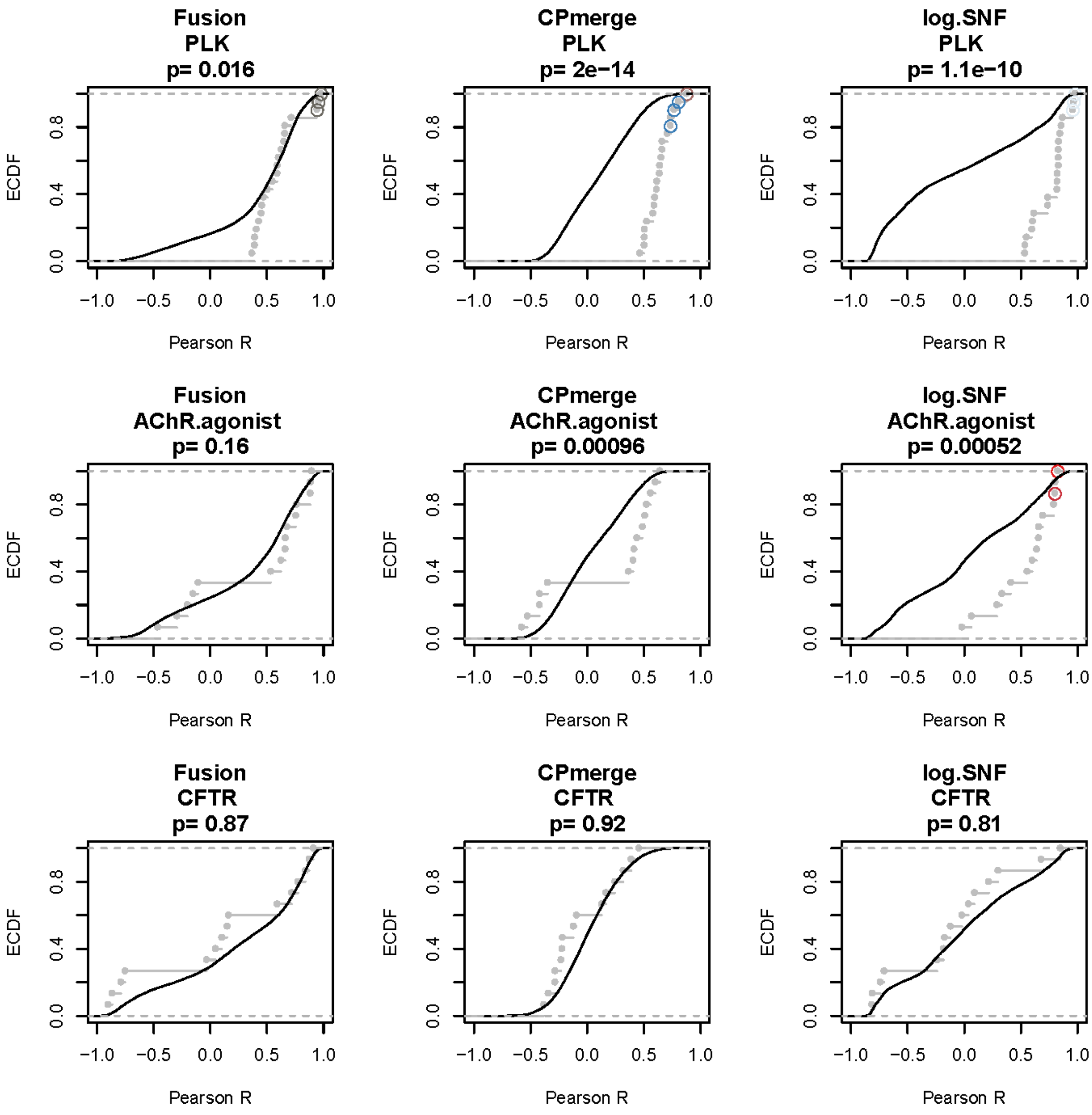

Fusion
PLK
p= 0.016
CPmerge
PLK
p= 2e−14
log.SNF
PLK
p= 1.1e−10
Fusion
AChR.agonist
p= 0.16
CPmerge
AChR.agonist
p= 0.00096
log.SNF
AChR.agonist
p= 0.00052
Fusion
CFTR
p= 0.87
CPmerge
CFTR
p= 0.92
log.SNF
CFTR
p= 0.81
ECDF
0.0
0.4
0.8
Pearson R
−1.0
−0.5
0.0
0.5
1.0

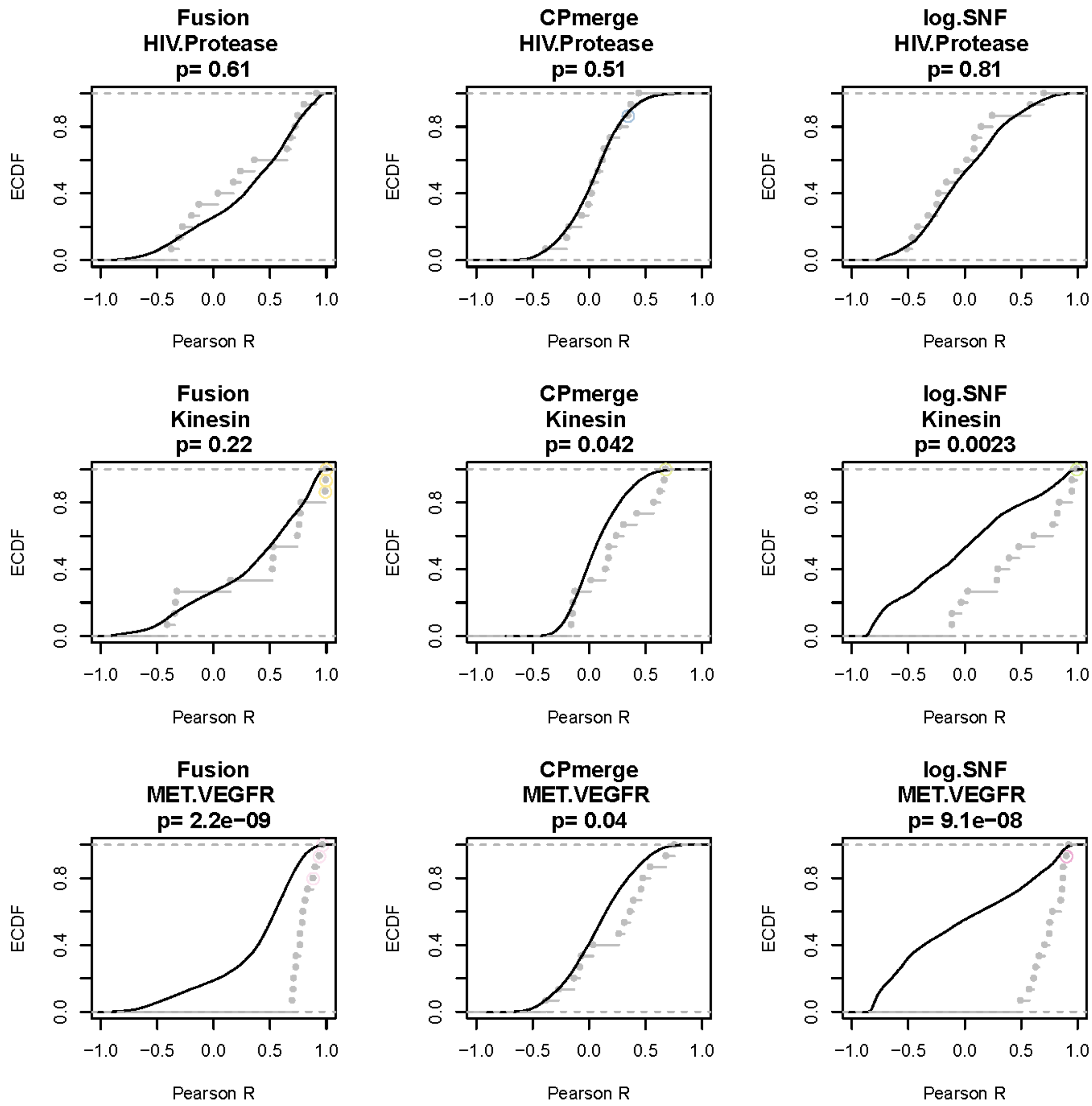
Fusion
HIV.Protease
p= 0.61
CPmerge
HIV.Protease
p= 0.51
log.SNF
HIV.Protease
p= 0.81
Fusion
Kinesin
p= 0.22
CPmerge
Kinesin
p= 0.042
log.SNF
Kinesin
p= 0.0023
Fusion
MET.VEGFR
p= 2.2e−09
CPmerge
MET.VEGFR
p= 0.04
log.SNF
MET.VEGFR
p= 9.1e−08
ECDF
0.0
0.4
0.8
−1.0
−0.5
0.0
0.5
1.0
Pearson R

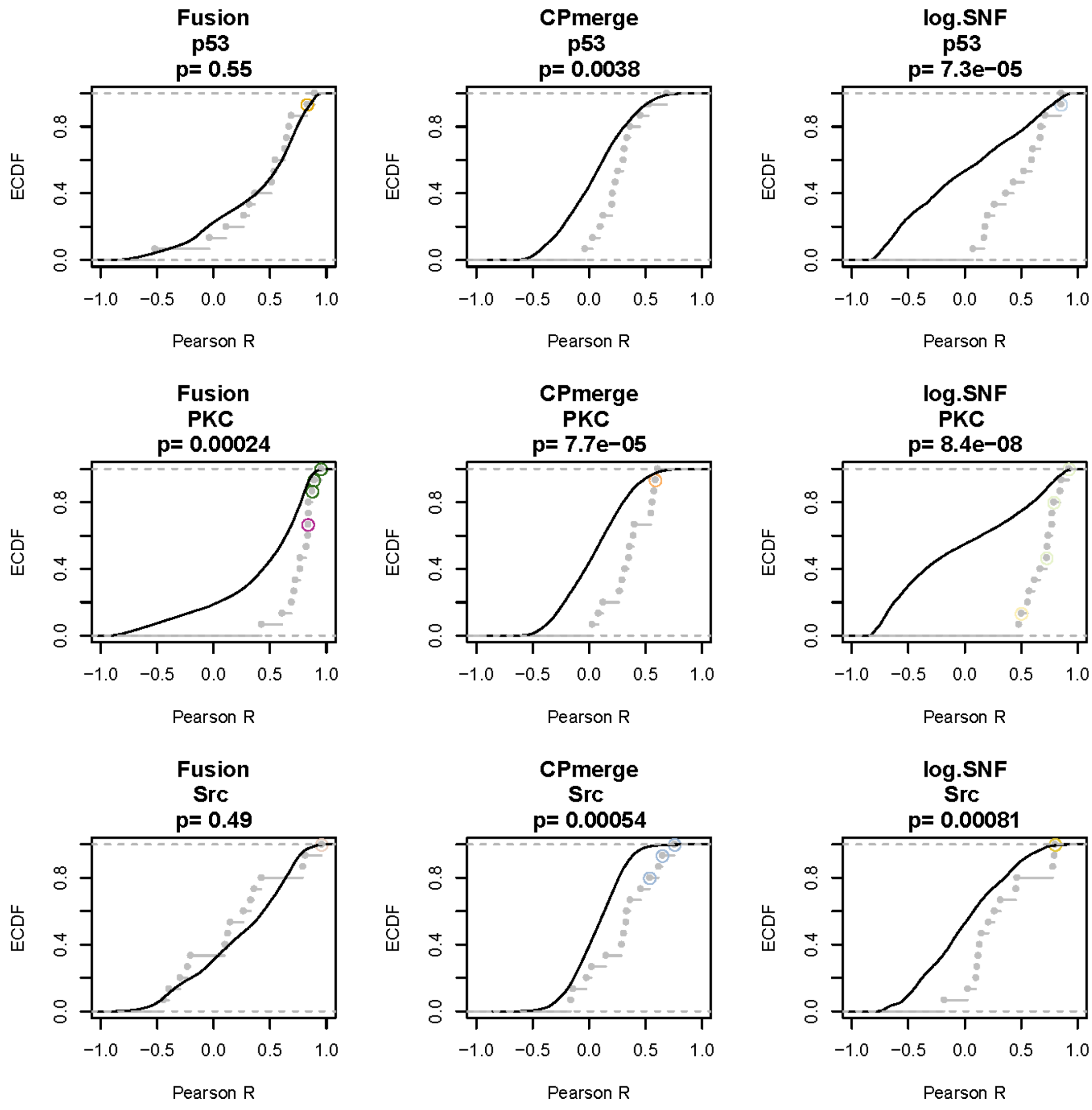
Fusion
p53
p= 0.55
CPmerge
p53
p= 0.0038
log.SNF
p53
p= 7.3e−05
Fusion
PKC
p= 0.00024
CPmerge
PKC
p= 7.7e−05
log.SNF
PKC
p= 8.4e−08
Fusion
Src
p= 0.49
CPmerge
Src
p= 0.00054
log.SNF
Src
p= 0.00081
ECDF
0.0
0.4
0.8
−1.0
−0.5
0.0
0.5
1.0
Pearson R

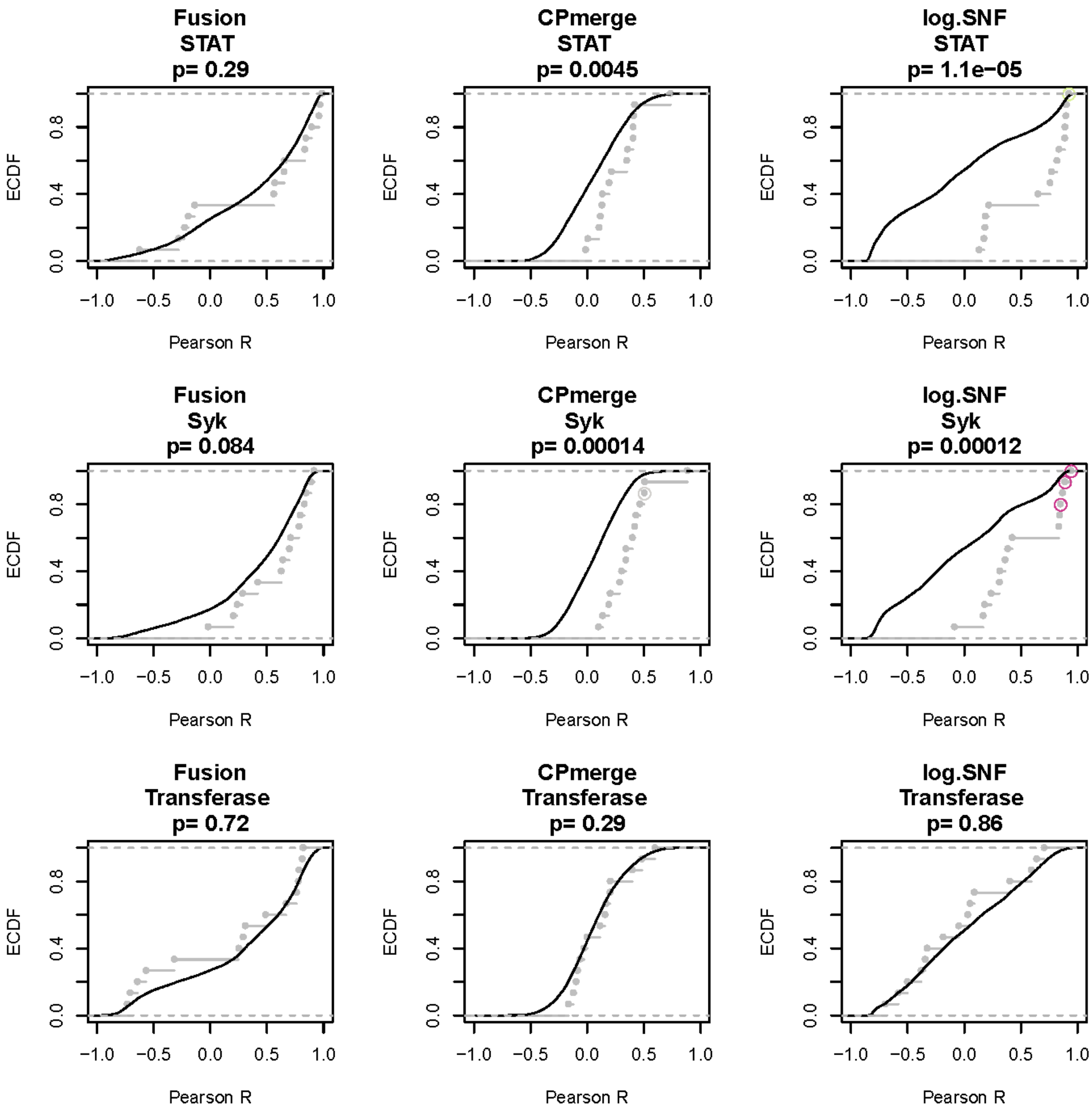
Fusion
STAT
p= 0.29
CPmerge
STAT
p= 0.0045
log.SNF
STAT
p= 1.1e−05
Fusion
Syk
p= 0.084
CPmerge
Syk
p= 0.00014
log.SNF
Syk
p= 0.00012
Fusion
Transferase
p= 0.72
CPmerge
Transferase
p= 0.29
log.SNF
Transferase
p= 0.86
ECDF
0.0
0.4
0.8
−1.0
−0.5
0.0
0.5
1.0
Pearson R

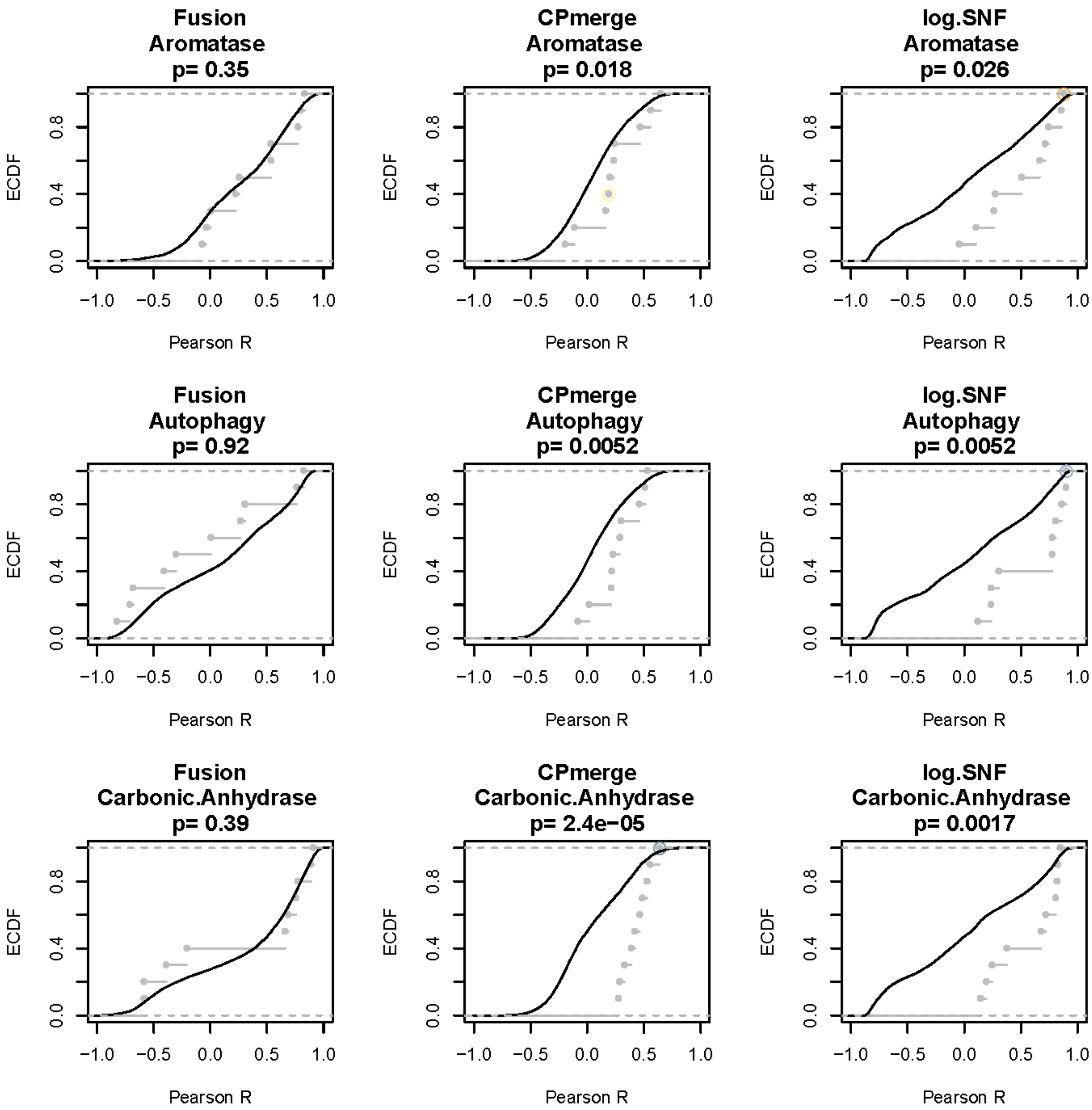
Fusion
Aromatase
p= 0.35
CPmerge
Aromatase
p= 0.018
log.SNF
Aromatase
p= 0.026
Fusion
Autophagy
p= 0.92
CPmerge
Autophagy
p= 0.0052
log.SNF
Autophagy
p= 0.0052
Fusion
Carbonic.Anhydrase
p= 0.39
CPmerge
Carbonic.Anhydrase
p= 2.4e−05
log.SNF
Carbonic.Anhydrase
p= 0.0017
ECDF
Pearson R
0.0
0.4
0.8
−1.0
−0.5
0.0
0.5
1.0

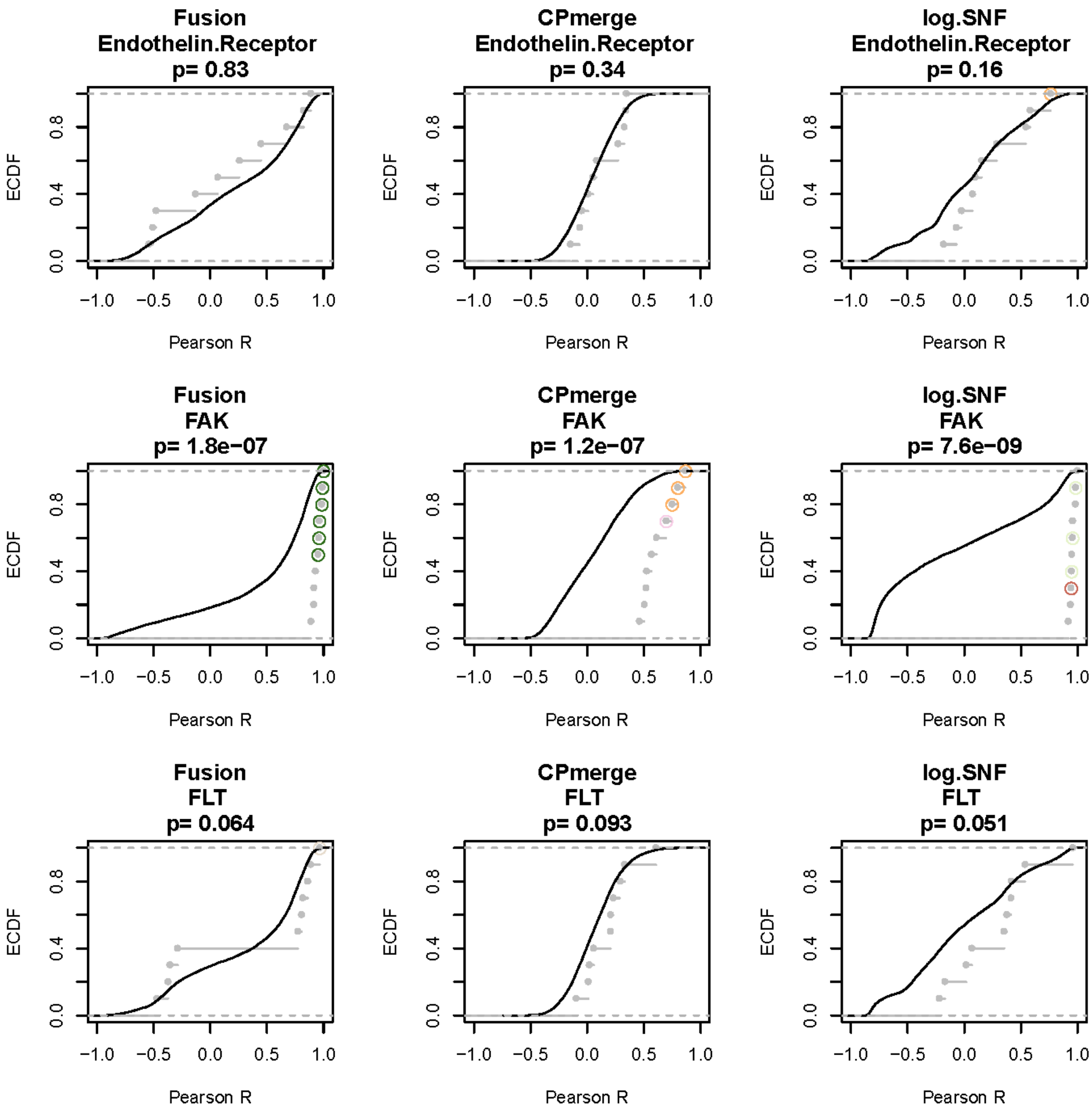

Fusion
Endothelin.Receptor
p= 0.83
CPmerge
Endothelin.Receptor
p= 0.34
log.SNF
Endothelin.Receptor
p= 0.16
Fusion
FAK
p= 1.8e−07
CPmerge
FAK
p= 1.2e−07
log.SNF
FAK
p= 7.6e−09
Fusion
FLT
p= 0.064
CPmerge
FLT
p= 0.093
log.SNF
FLT
p= 0.051
ECDF
Pearson R
0.0
0.4
0.8
−1.0
−0.5
0.0
0.5
1.0

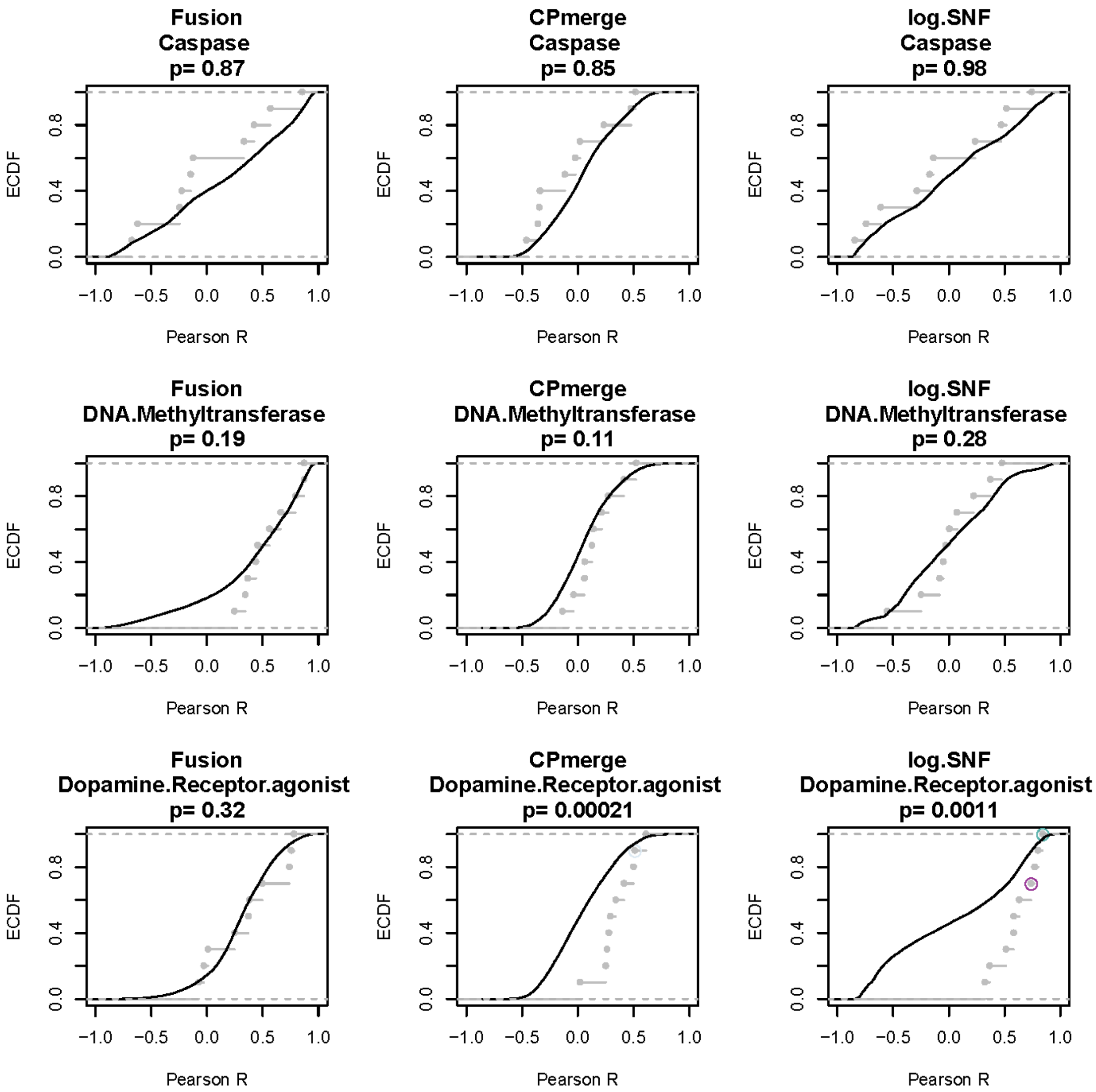
Fusion
Caspase
p= 0.87
CPmerge
Caspase
p= 0.85
log.SNF
Caspase
p= 0.98
Fusion
DNA.Methyltransferase
p= 0.19
CPmerge
DNA.Methyltransferase
p= 0.11
log.SNF
DNA.Methyltransferase
p= 0.28
Fusion
Dopamine.Receptor.agonist
p= 0.32
CPmerge
Dopamine.Receptor.agonist
p= 0.00021
log.SNF
Dopamine.Receptor.agonist
p= 0.0011
ECDF
0.0
0.4
0.8
−1.0
−0.5
0.0
0.5
1.0
Pearson R

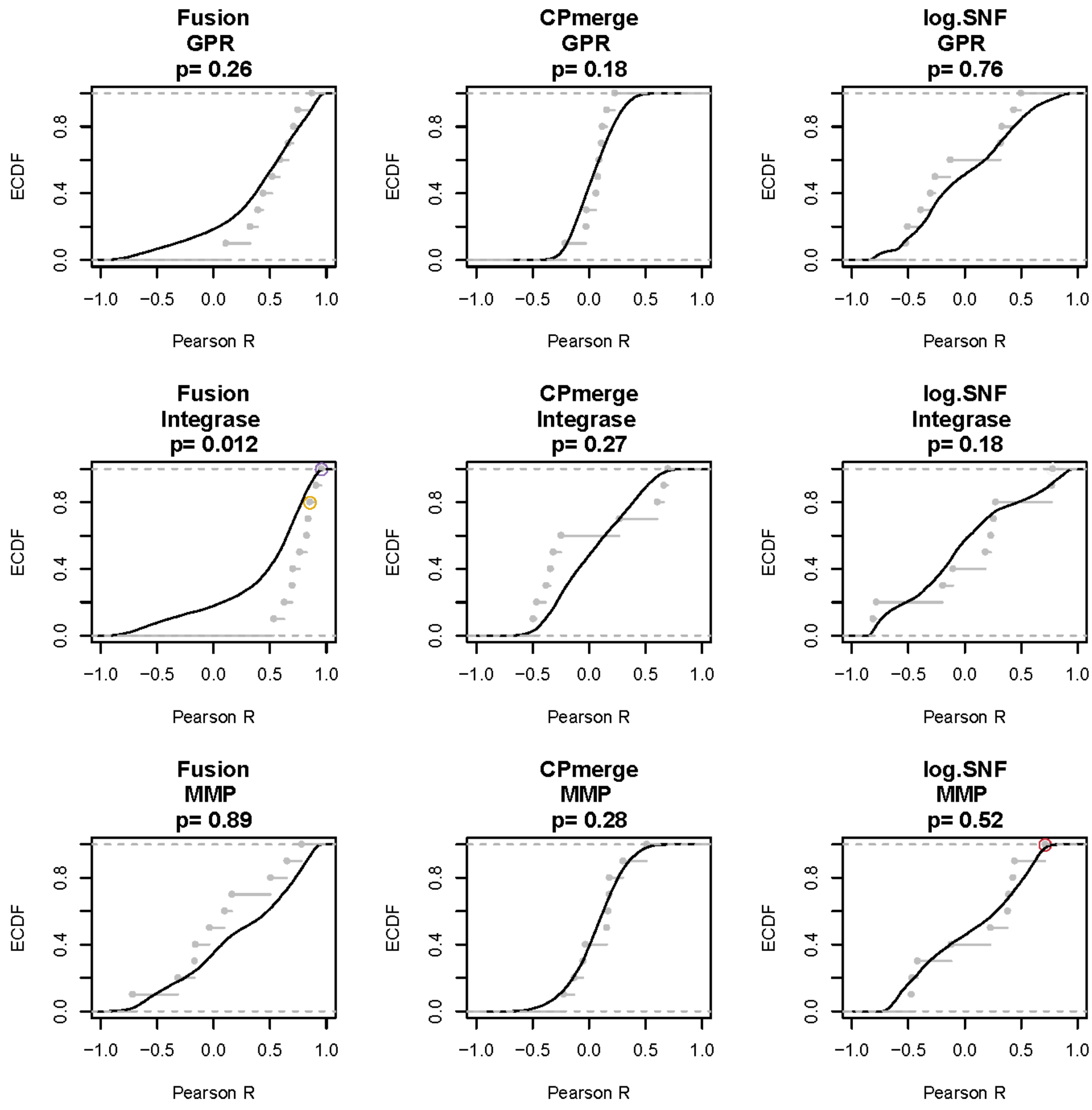
Fusion
GPR
p= 0.26
CPmerge
GPR
p= 0.18
log.SNF
GPR
p= 0.76
Fusion
Integrase
p= 0.012
CPmerge
Integrase
p= 0.27
log.SNF
Integrase
p= 0.18
Fusion
MMP
p= 0.89
CPmerge
MMP
p= 0.28
log.SNF
MMP
p= 0.52
ECDF
0.0
0.4
0.8
−1.0
−0.5
0.0
0.5
1.0
Pearson R

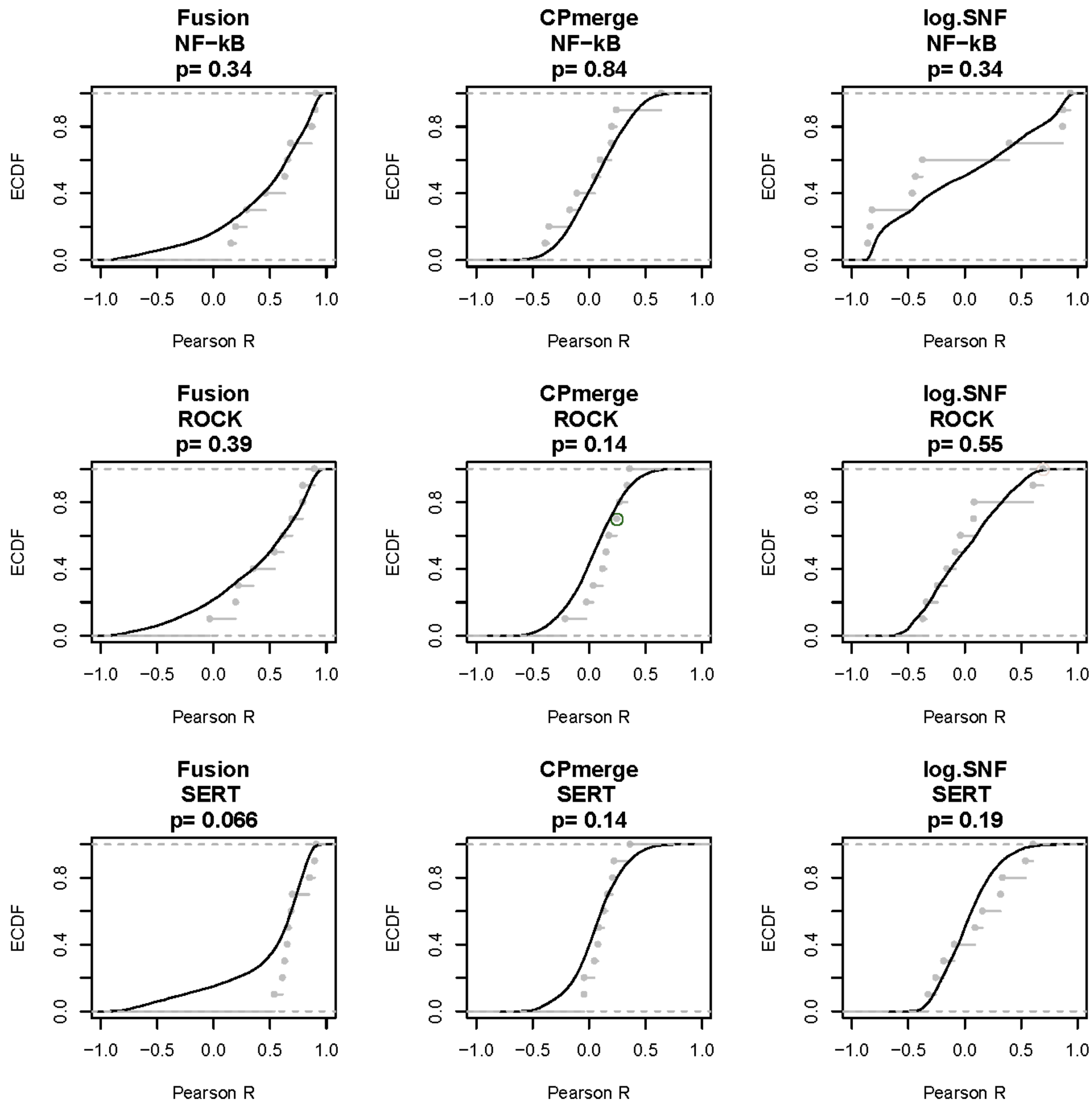
Fusion
NF−kB
p= 0.34
CPmerge
NF−kB
p= 0.84
log.SNF
NF−kB
p= 0.34
Fusion
ROCK
p= 0.39
CPmerge
ROCK
p= 0.14
log.SNF
ROCK
p= 0.55
Fusion
SERT
p= 0.066
CPmerge
SERT
p= 0.14
log.SNF
SERT
p= 0.19
ECDF
0.0
0.4
0.8
−1.0
−0.5
0.0
0.5
1.0
Pearson R

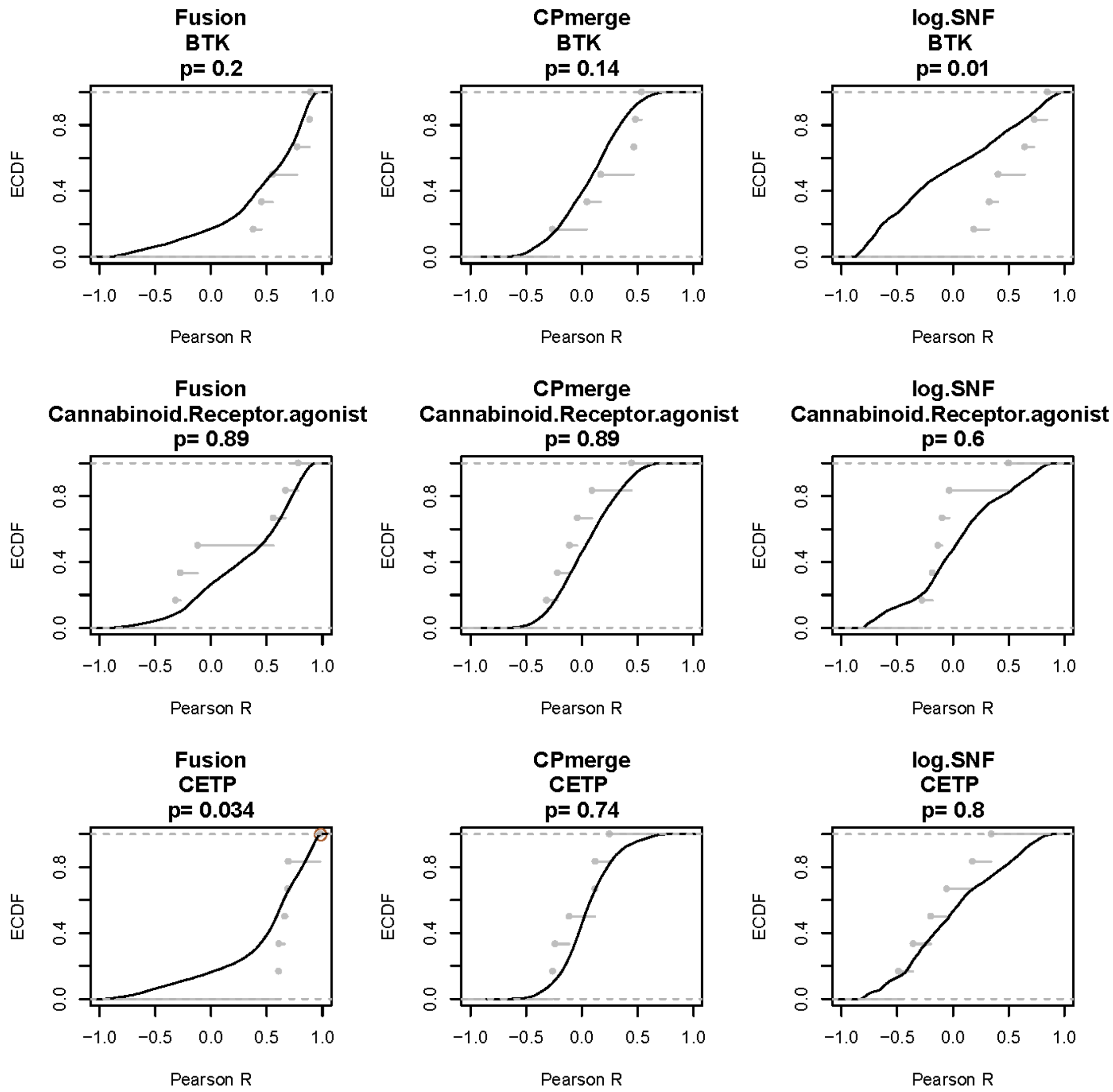
Fusion
BTK
p= 0.2
CPmerge
BTK
p= 0.14
log.SNF
BTK
p= 0.01
Fusion
Cannabinoid.Receptor.agonist
p= 0.89
CPmerge
Cannabinoid.Receptor.agonist
p= 0.89
log.SNF
Cannabinoid.Receptor.agonist
p= 0.6
Fusion
CETP
p= 0.034
CPmerge
CETP
p= 0.74
log.SNF
CETP
p= 0.8
ECDF
0.0
0.4
0.8
−1.0
−0.5
0.0
0.5
1.0
Pearson R

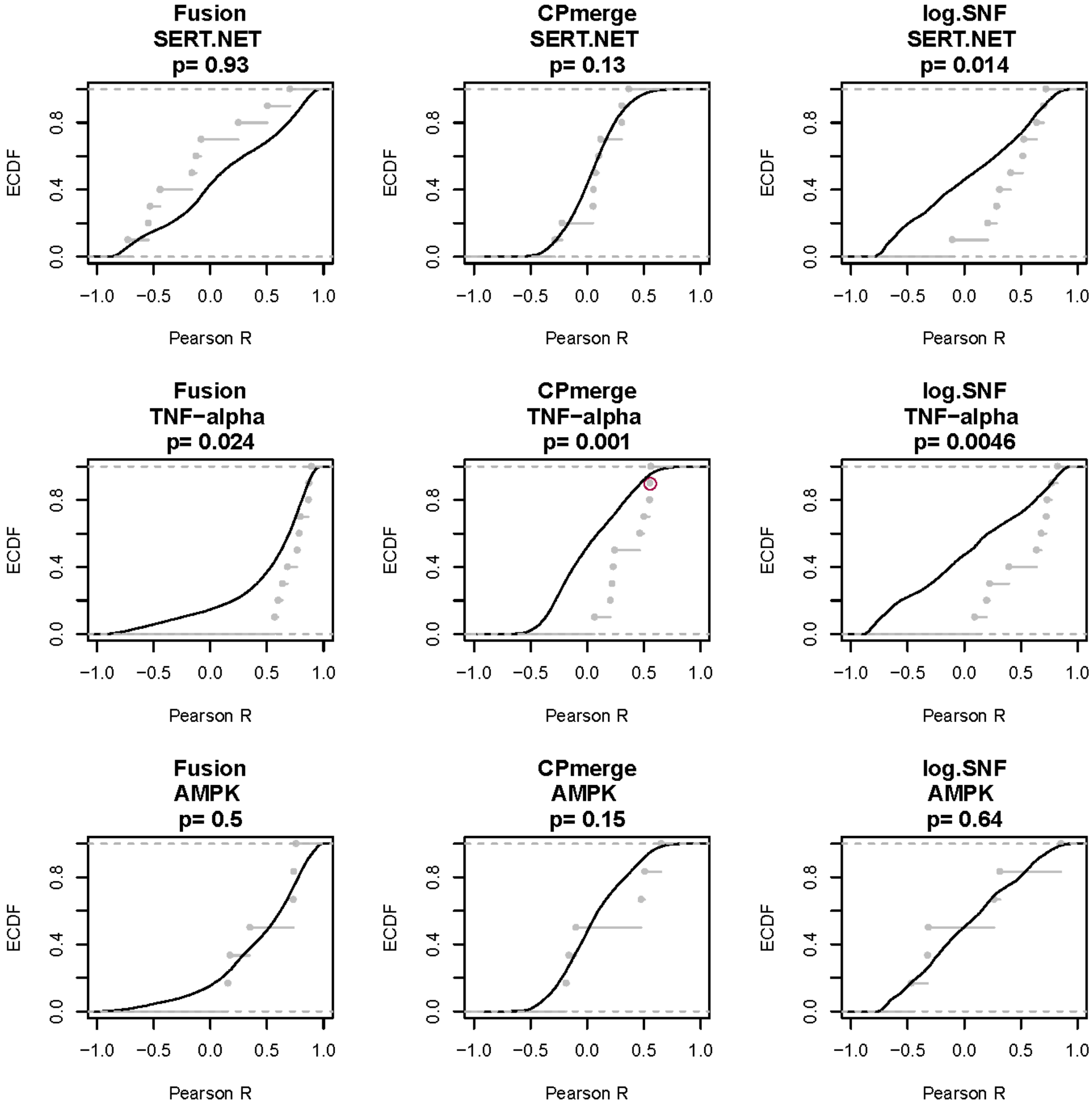

Fusion
SERT.NET
p= 0.93
CPmerge
SERT.NET
p= 0.13
log.SNF
SERT.NET
p= 0.014
Fusion
TNF−alpha
p= 0.024
CPmerge
TNF−alpha
p= 0.001
log.SNF
TNF−alpha
p= 0.0046
Fusion
AMPK
p= 0.5
CPmerge
AMPK
p= 0.15
log.SNF
AMPK
p= 0.64
ECDF
0.0
0.4
0.8
−1.0
−0.5
0.0
0.5
1.0
Pearson R

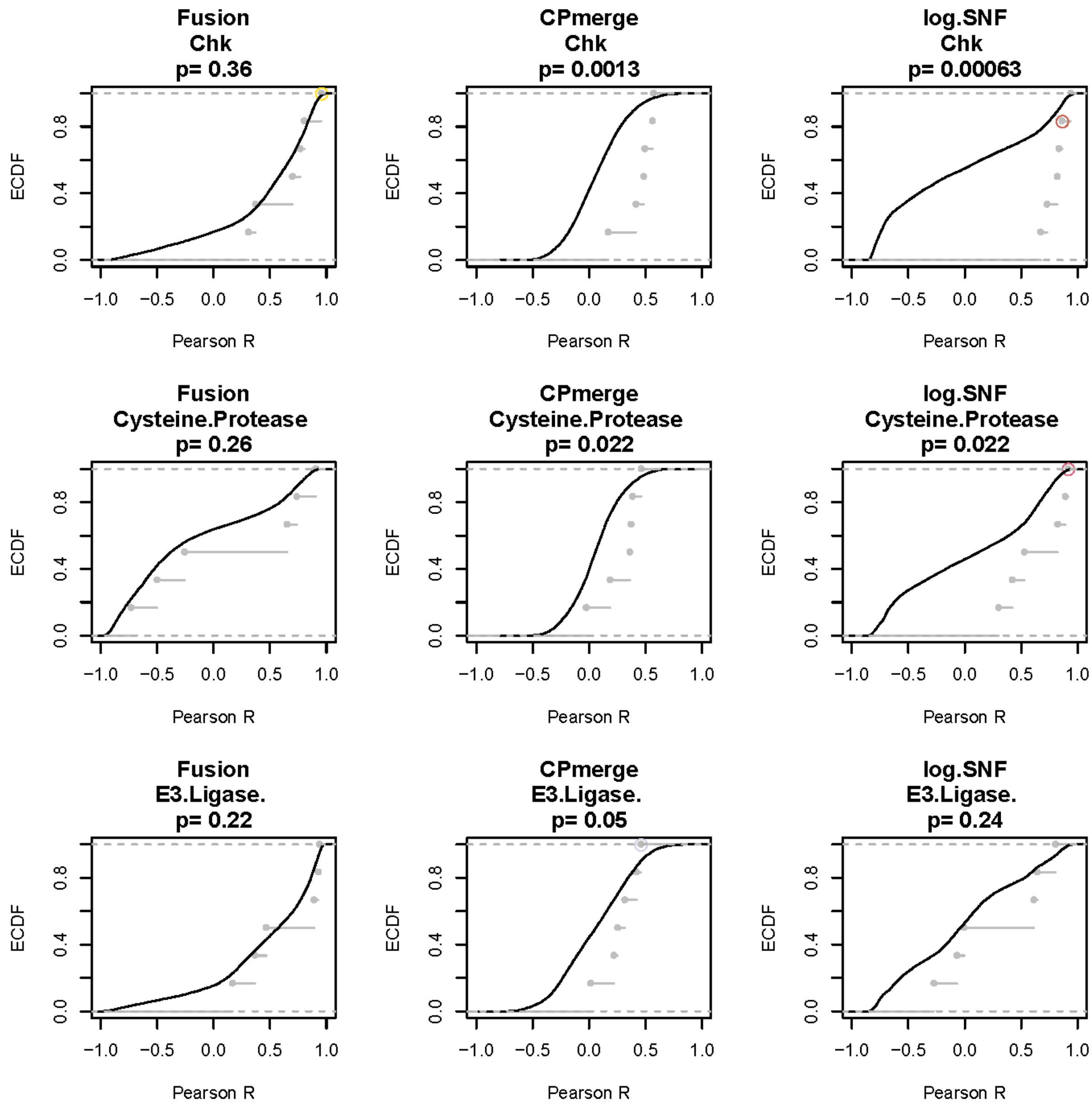

Fusion
Chk
p= 0.36
CPmerge
Chk
p= 0.0013
log.SNF
Chk
p= 0.00063
Fusion
Cysteine.Protease
p= 0.26
CPmerge
Cysteine.Protease
p= 0.022
log.SNF
Cysteine.Protease
p= 0.022
Fusion
E3.Ligase.
p= 0.22
CPmerge
E3.Ligase.
p= 0.05
log.SNF
E3.Ligase.
p= 0.24
ECDF
0.0
0.4
0.8
−1.0
−0.5
0.0
0.5
1.0
Pearson R

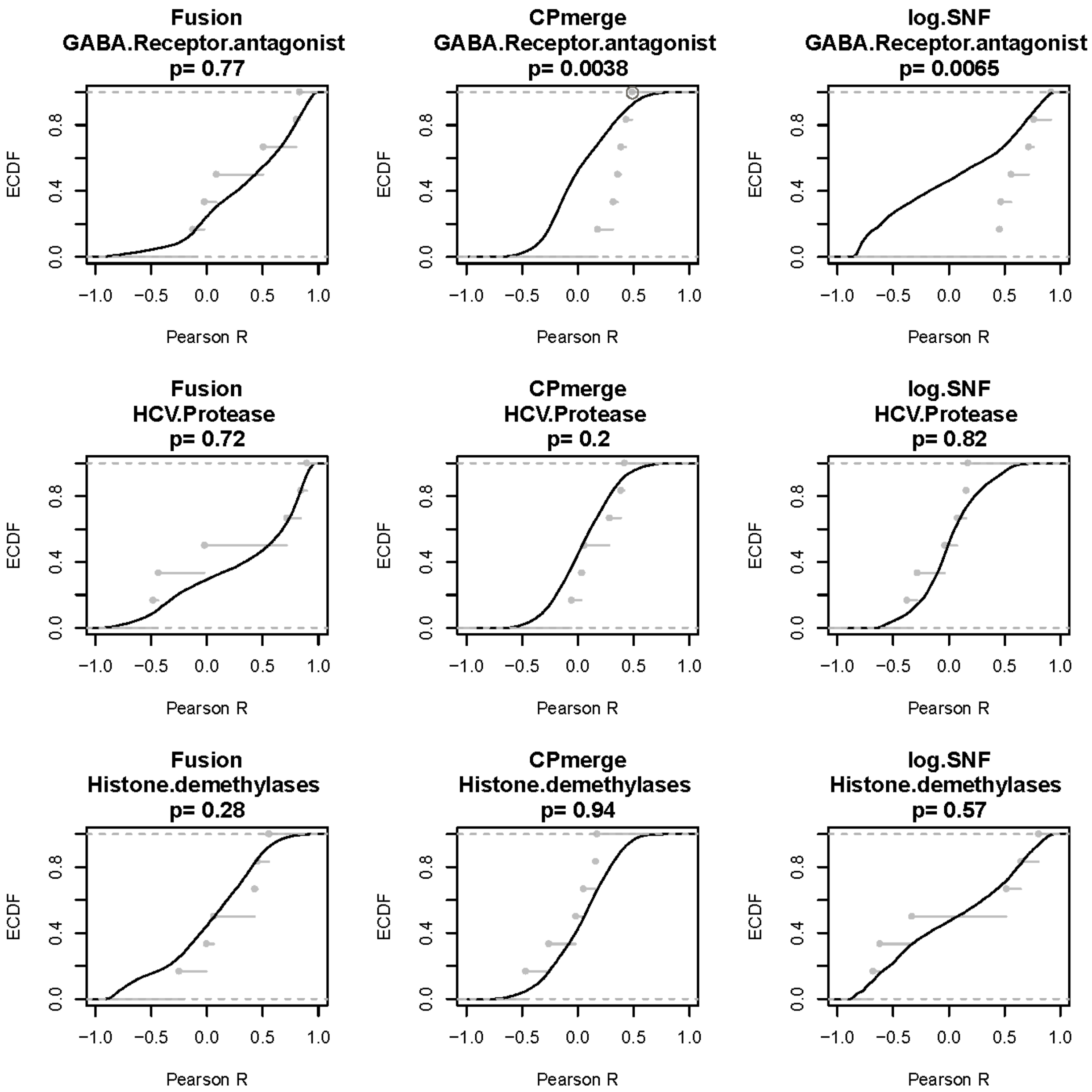
Fusion
GABA.Receptor.antagonist
p= 0.77
CPmerge
GABA.Receptor.antagonist
p= 0.0038
log.SNF
GABA.Receptor.antagonist
p= 0.0065
Fusion
HCV.Protease
p= 0.72
CPmerge
HCV.Protease
p= 0.2
log.SNF
HCV.Protease
p= 0.82
Fusion
Histone.demethylases
p= 0.28
CPmerge
Histone.demethylases
p= 0.94
log.SNF
Histone.demethylases
p= 0.57
ECDF
0.0
0.4
0.8
−1.0
−0.5
0.0
0.5
1.0
Pearson R

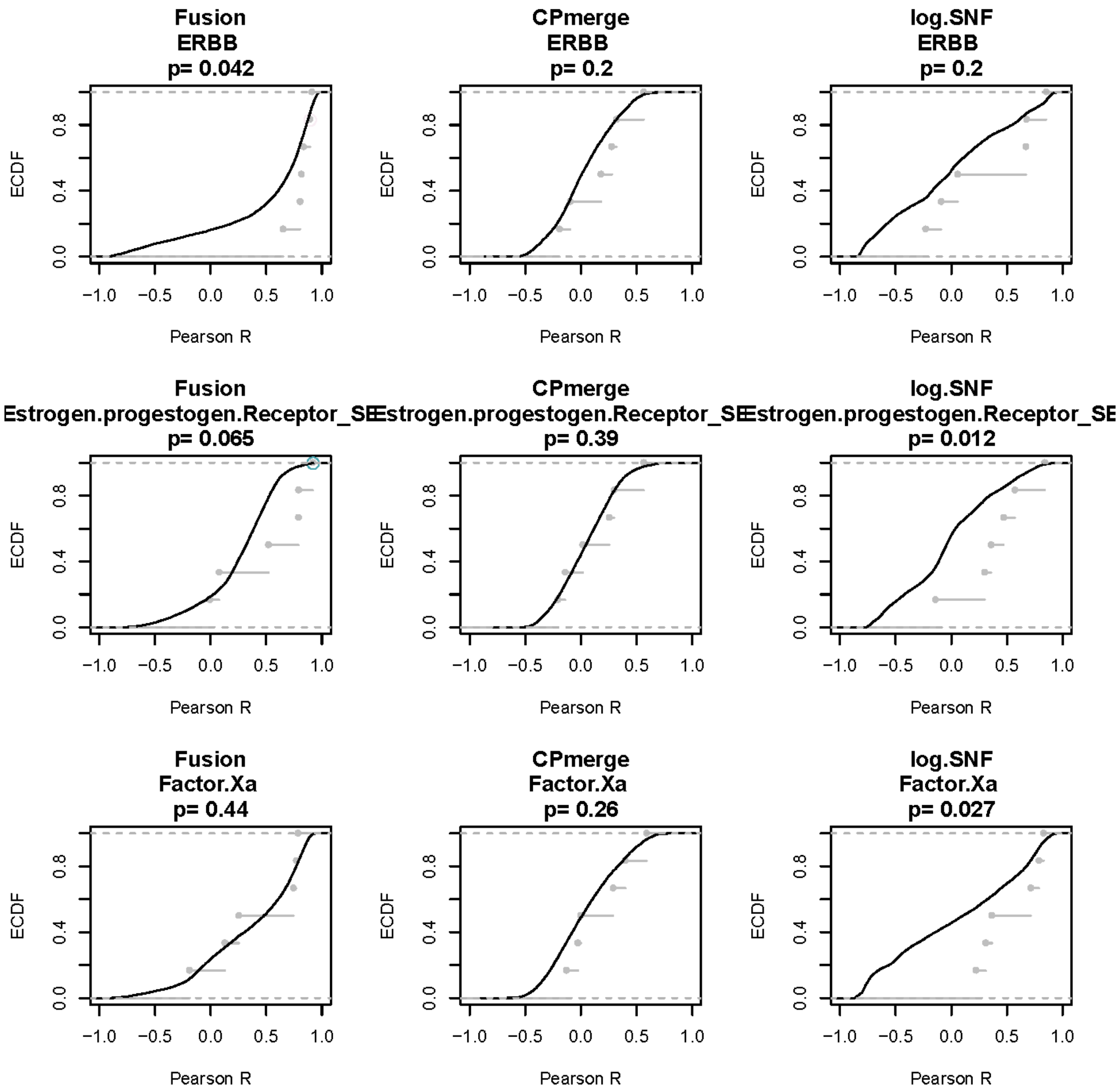
Fusion
ERBB
p= 0.042
CPmerge
ERBB
p= 0.2
log.SNF
ERBB
p= 0.2
Fusion
Estrogen.progestogen.Receptor_SE
p= 0.065
CPmerge
Estrogen.progestogen.Receptor_SE
p= 0.39
log.SNF
Estrogen.progestogen.Receptor_SE
p= 0.012
Fusion
Factor.Xa
p= 0.44
CPmerge
Factor.Xa
p= 0.26
log.SNF
Factor.Xa
p= 0.027
ECDF
0.0
0.4
0.8
−1.0
−0.5
0.0
0.5
1.0
Pearson R

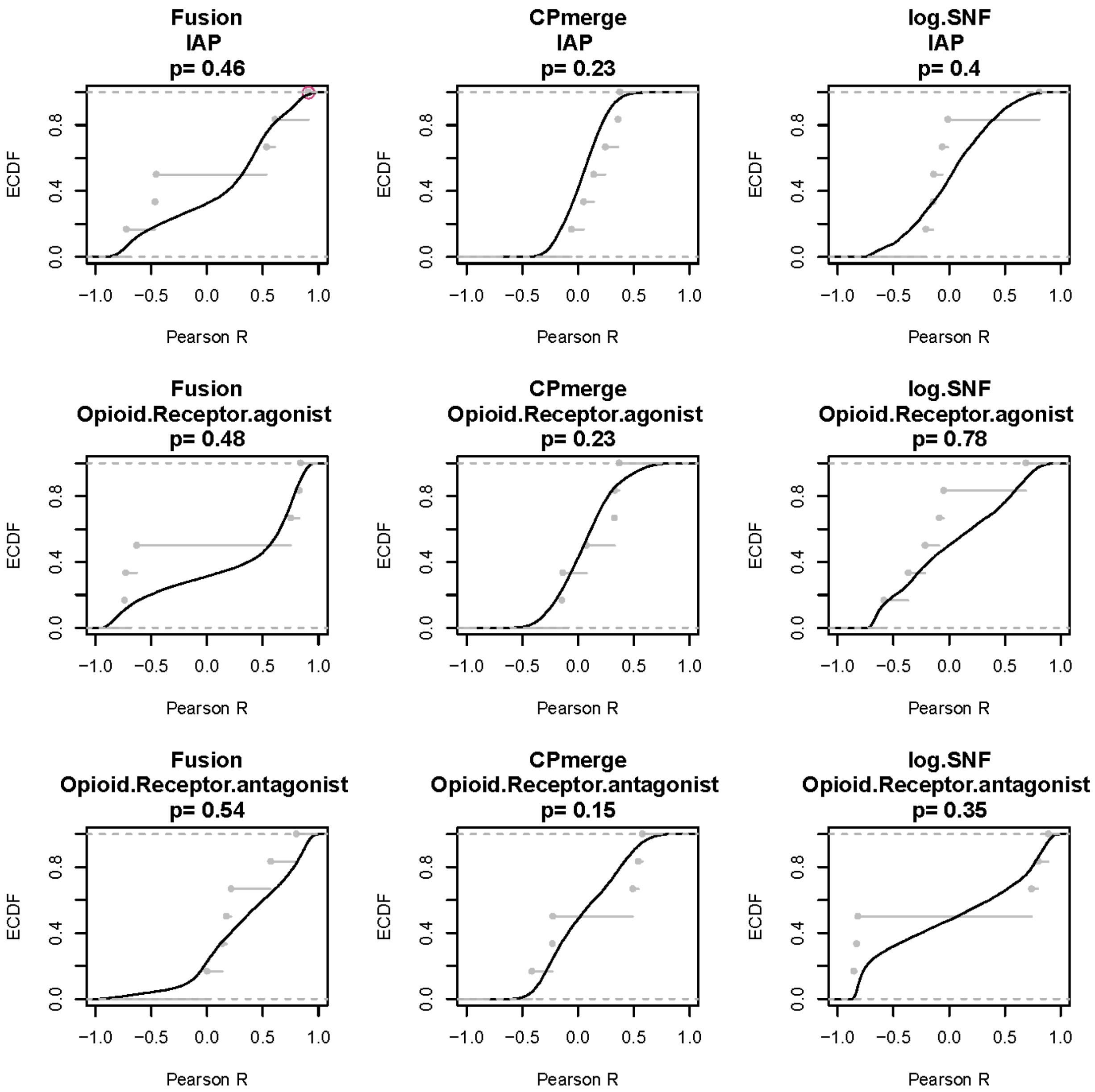
Fusion
IAP
p= 0.46
CPmerge
IAP
p= 0.23
log.SNF
IAP
p= 0.4
Fusion
Opioid.Receptor.agonist
p= 0.48
CPmerge
Opioid.Receptor.agonist
p= 0.23
log.SNF
Opioid.Receptor.agonist
p= 0.78
Fusion
Opioid.Receptor.antagonist
p= 0.54
CPmerge
Opioid.Receptor.antagonist
p= 0.15
log.SNF
Opioid.Receptor.antagonist
p= 0.35
ECDF
Pearson R
0.0
0.4
0.8
−1.0
−0.5
0.0
0.5
1.0

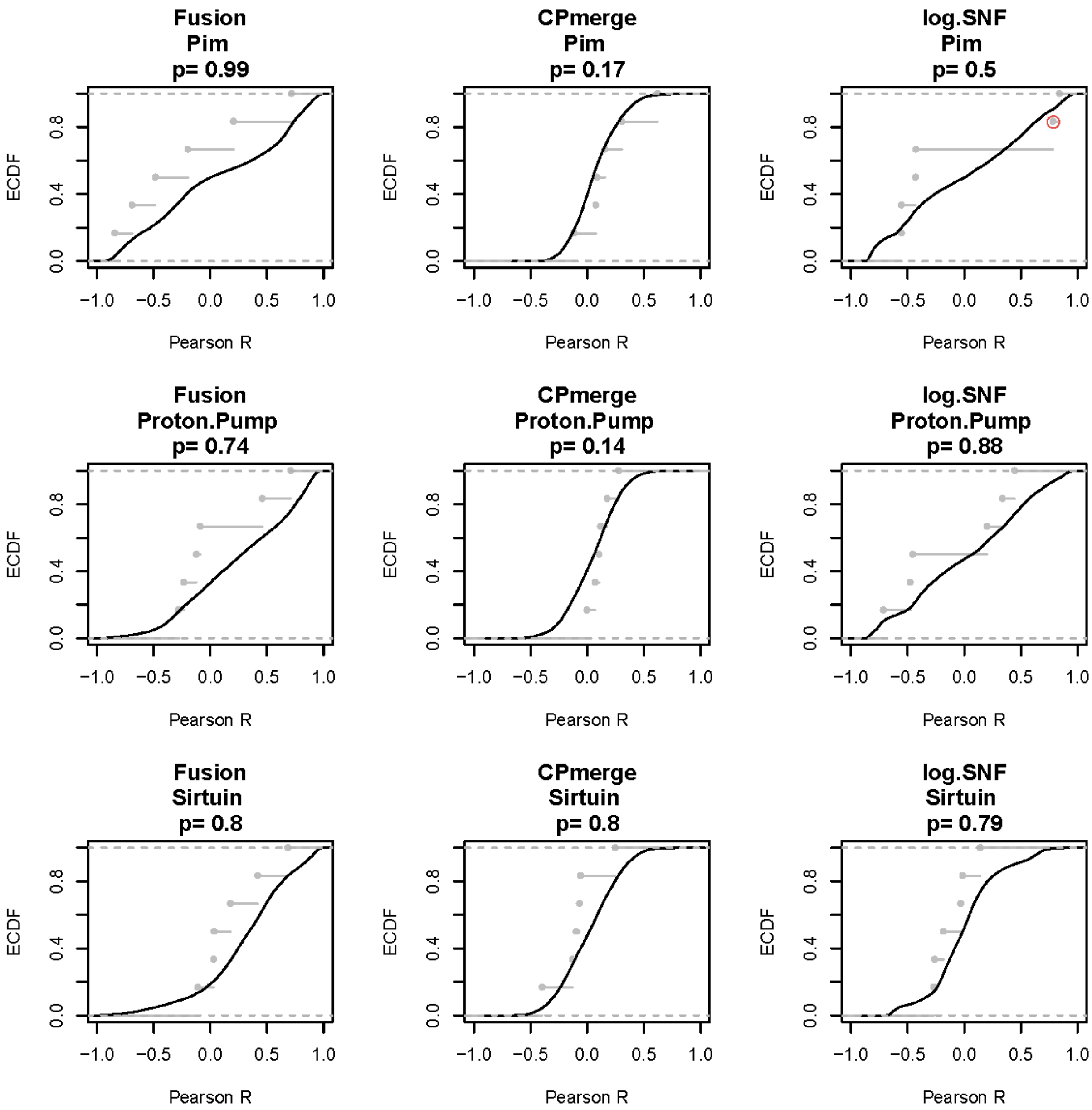
Fusion
Pim
p= 0.99
CPmerge
Pim
p= 0.17
log.SNF
Pim
p= 0.5
Fusion
Proton.Pump
p= 0.74
CPmerge
Proton.Pump
p= 0.14
log.SNF
Proton.Pump
p= 0.88
Fusion
Sirtuin
p= 0.8
CPmerge
Sirtuin
p= 0.8
log.SNF
Sirtuin
p= 0.79
ECDF
0.0
0.4
0.8
−1.0
−0.5
0.0
0.5
1.0
Pearson R

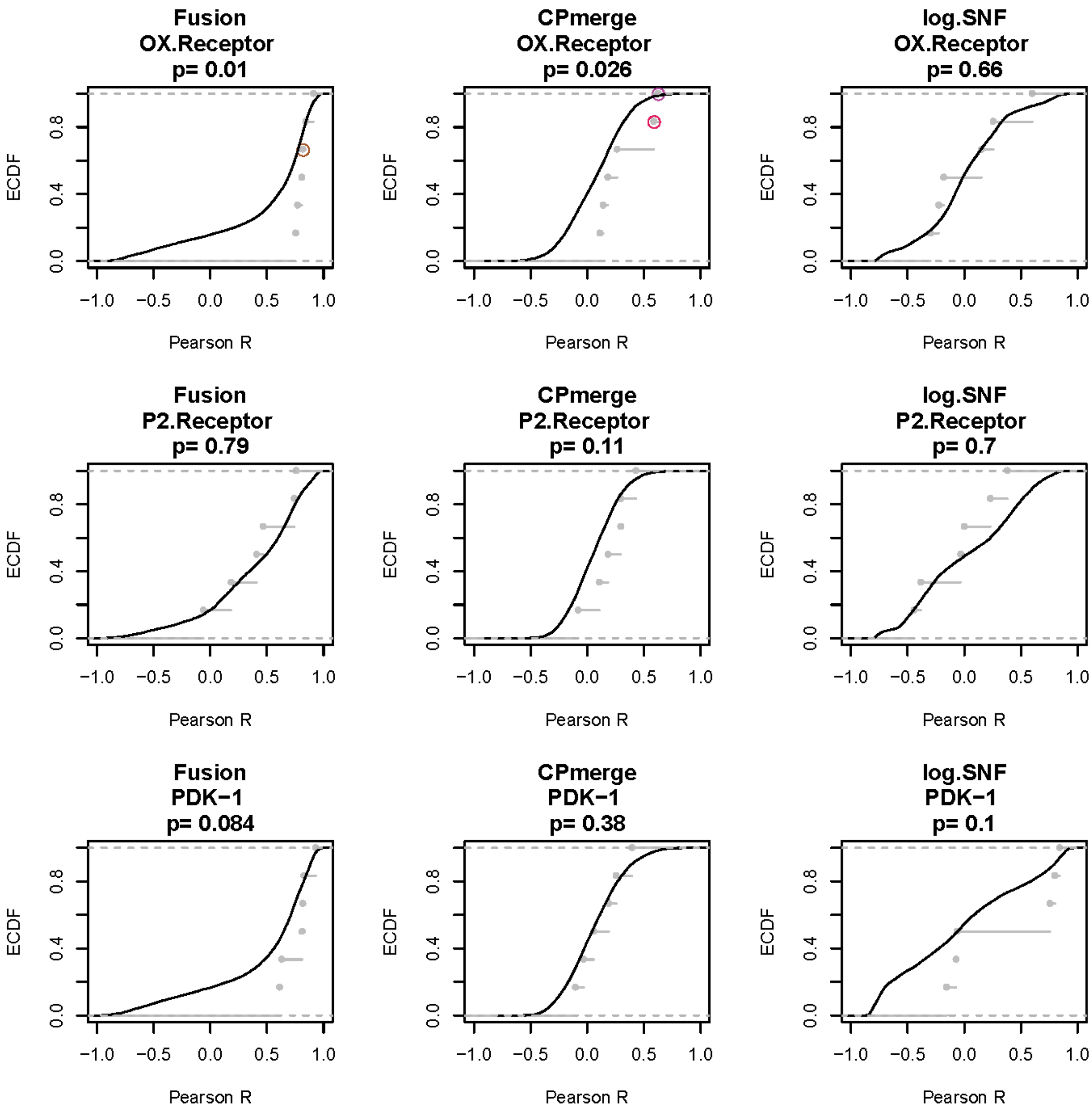

Fusion
OX.Receptor
p= 0.01
CPmerge
OX.Receptor
p= 0.026
log.SNF
OX.Receptor
p= 0.66
Fusion
P2.Receptor
p= 0.79
CPmerge
P2.Receptor
p= 0.11
log.SNF
P2.Receptor
p= 0.7
Fusion
PDK−1
p= 0.084
CPmerge
PDK−1
p= 0.38
log.SNF
PDK−1
p= 0.1
ECDF
0.0
0.4
0.8
−1.0
−0.5
0.0
0.5
1.0
Pearson R

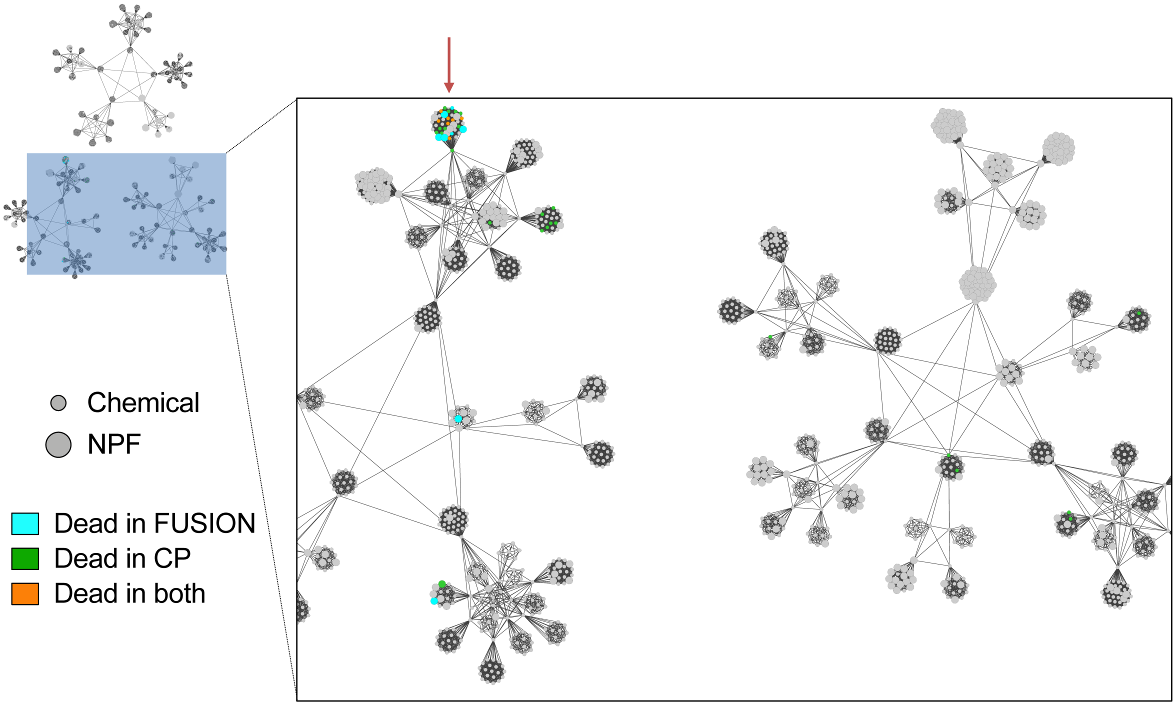

Supplementary Figure 9: “Dead” Compounds in CP and FUSION cluster together in the SNF-Euclidean APC map. Red arrow indicates a cluster enriched with compounds that were flagged as dead in either or both datasets.

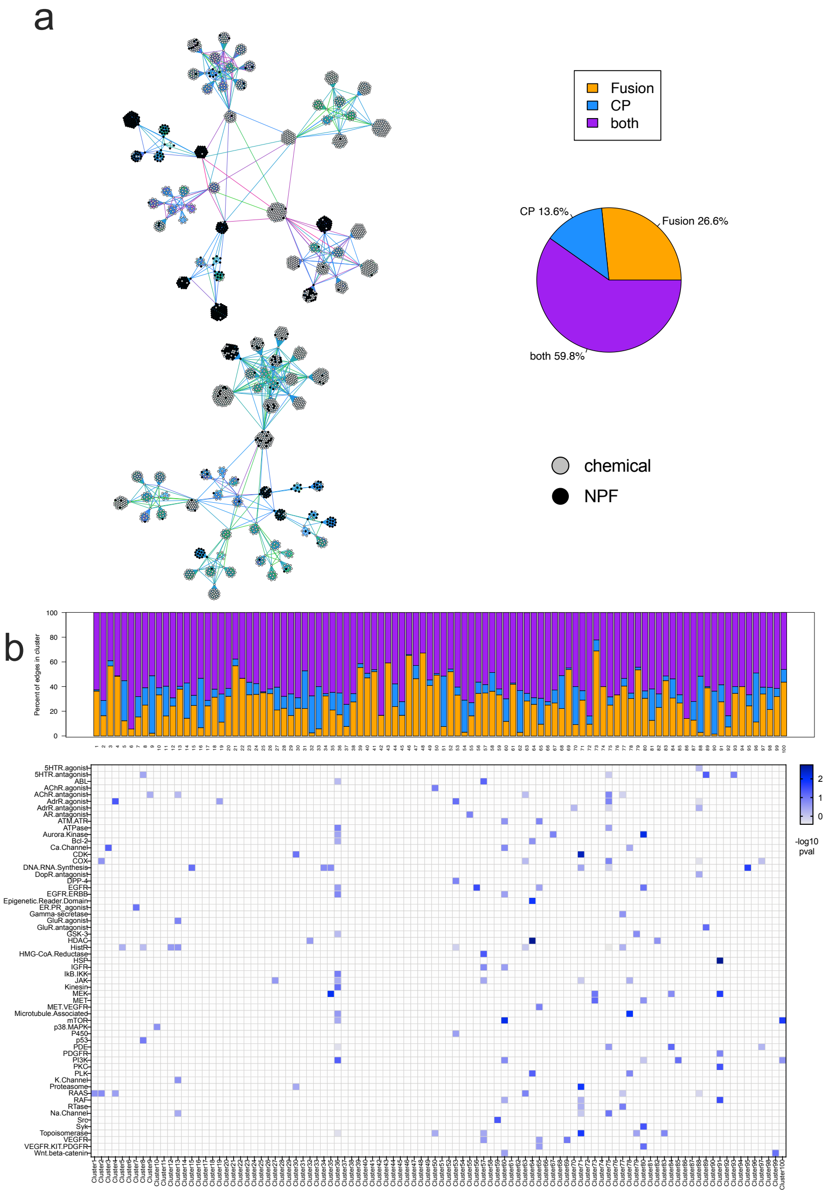

Supplementary Figure 10. Affinity propagation clustering map of the SNF-pearson network. A) Hierarchical affinity propagation clustering map of the SNF network using Pearson correlation as the similarity metric. Edges are colored based on contribution from individual datasets: Orange, supported by FUSION; blue, supported by CP; purple, supported by both datasets. Perturbagen type is indicated by node color: black, NPF; gray, pure chemical. B) Bar plot showing the percent of total edges in each APC cluster that are supported by FUSION, CP, or both datasets. Clusters are labeled by cluster number. C) Heatmap showing minus log10 p-values calculated by hypergeometric test for each target annotation class, per APC cluster. Target classes without significant enrichment in any cluster are omitted (Bonferroni-corrected alpha = 0.0016).

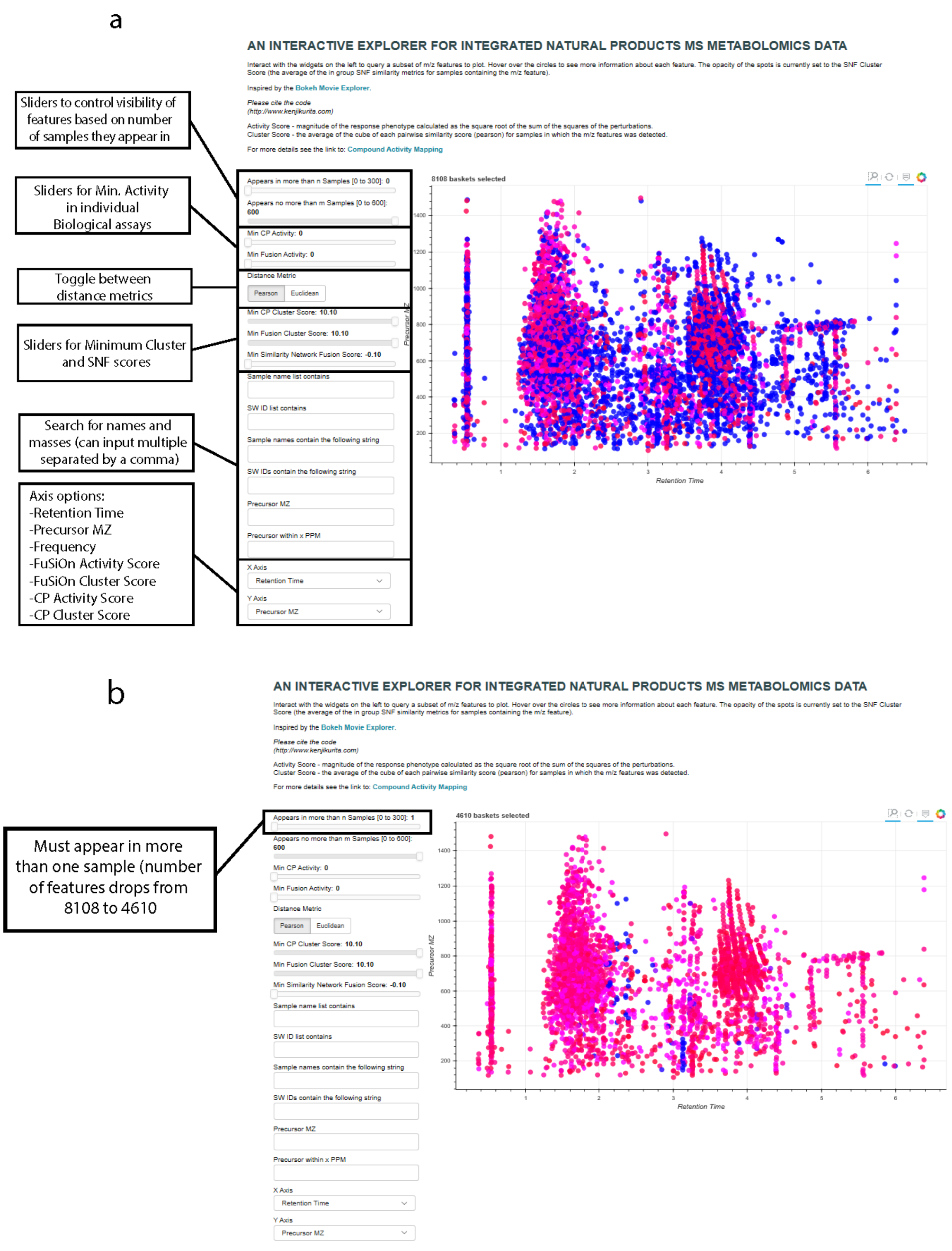
a
AN INTERACTIVE EXPLORER FOR INTEGRATED NATURAL PRODUCTS MS METABOLOMICS DATA
Sliders to control visibility of features based on number of samples they appear in
Sliders for Min. Activity in individual Biological assays
Toggle between distance metrics
Sliders for Minimum Cluster and SNF scores
Search for names and masses (can input multiple separated by a comma)
Axis options:
-Retention Time
-Precursor MZ
-Frequency
-FuSiOn Activity Score
-FuSiOn Cluster Score
-CP Activity Score
-CP Cluster Score
8108 baskets selected
Appears in more than n Samples [0 to 300]: 0
Appears no more than m Samples [0 to 600]: 600
Min CP Activity: 0
Min Fusion Activity: 0
Distance Metric
Pearson
Euclidean
Min CP Cluster Score: 10.10
Min Fusion Cluster Score: 10.10
Min Similarity Network Fusion Score: -0.10
Sample name list contains
SW ID list contains
Sample names contain the following string
SW IDs contain the following string
Precursor MZ
Precursor within x PPM
X Axis
Retention Time
Y Axis
Precursor MZ
Retention Time
b
AN INTERACTIVE EXPLORER FOR INTEGRATED NATURAL PRODUCTS MS METABOLOMICS DATA
Must appear in more than one sample (number of features drops from 8108 to 4610
4610 baskets selected
Appears in more than n Samples [0 to 300]: 1
Retention Time

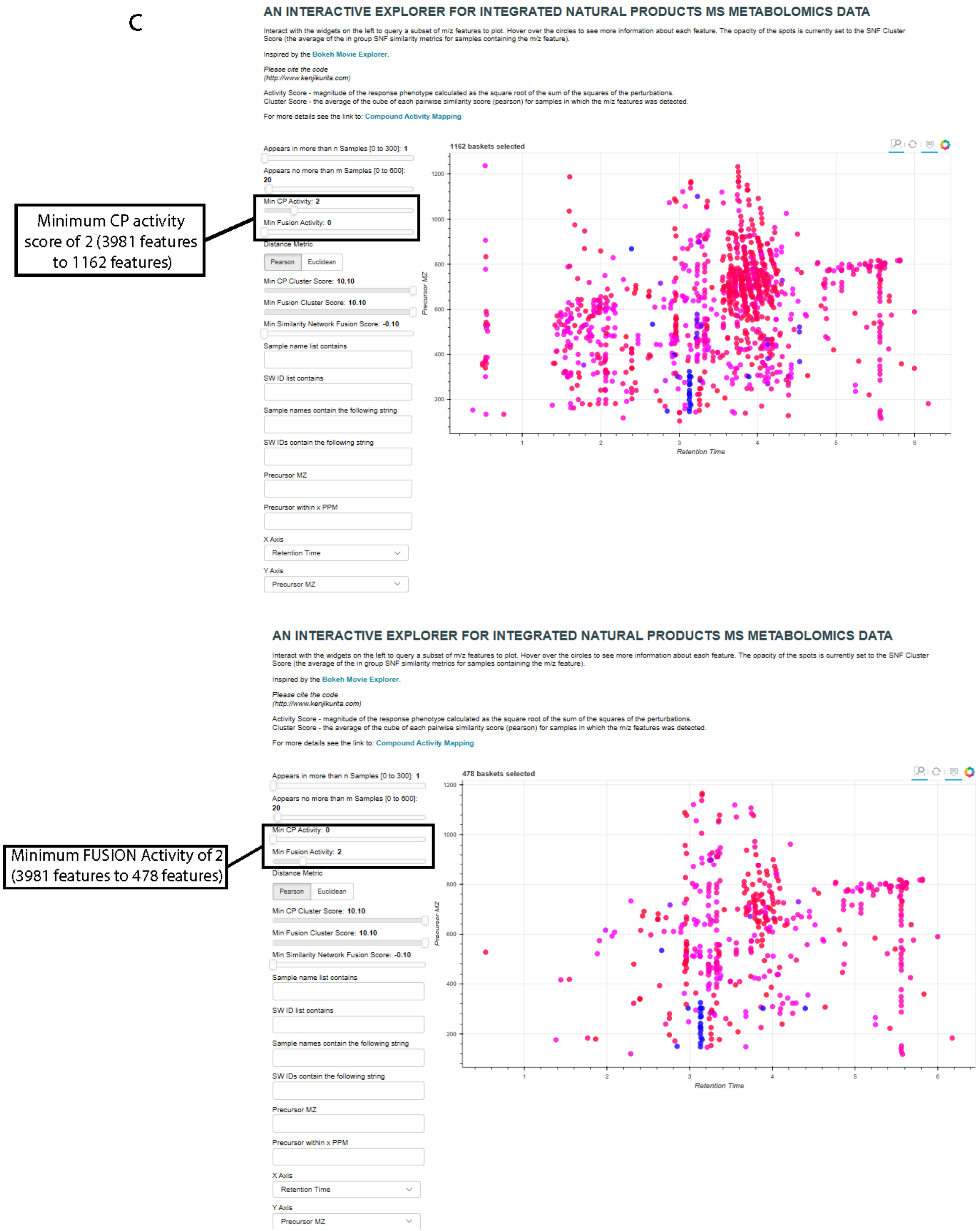
C
AN INTERACTIVE EXPLORER FOR INTEGRATED NATURAL PRODUCTS MS METABOLOMICS DATA
Interact with the widgets on the left to query a subset of m/z features to plot. Hover over the circles to see more information about each feature. The opacity of the spots is currently set to the SNF Cluster Score (the average of the in group SNF similarity metrics for samples containing the m/z feature).
Inspired by the Bokeh Movie Explorer.
Please cite the code
(http://www.kenjikurita.com)
Activity Score - magnitude of the response phenotype calculated as the square root of the sum of the squares of the perturbations.
Cluster Score - the average of the cube of each pairwise similarity score (pearson) for samples in which the m/z features was detected.
For more details see the link to: Compound Activity Mapping
Appears in more than n Samples [0 to 300]: 1
Appears no more than m Samples [0 to 600]: 20
Min CP Activity: 2
Min Fusion Activity: 0
Distance Metric
Pearson
Euclidean
Min CP Cluster Score: 10.10
Min Fusion Cluster Score: 10.10
Min Similarity Network Fusion Score: -0.10
Sample name list contains
SW ID list contains
Sample names contain the following string
SW IDs contain the following string
Precursor MZ
Precursor within x PPM
X Axis
Retention Time
Y Axis
Precursor MZ
1162 baskets selected
Precursor MZ
Retention Time
Minimum CP activity score of 2 (3981 features to 1162 features)
AN INTERACTIVE EXPLORER FOR INTEGRATED NATURAL PRODUCTS MS METABOLOMICS DATA
Min CP Activity: 0
Min Fusion Activity: 2
478 baskets selected
Minimum FUSION Activity of 2 (3981 features to 478 features)

d

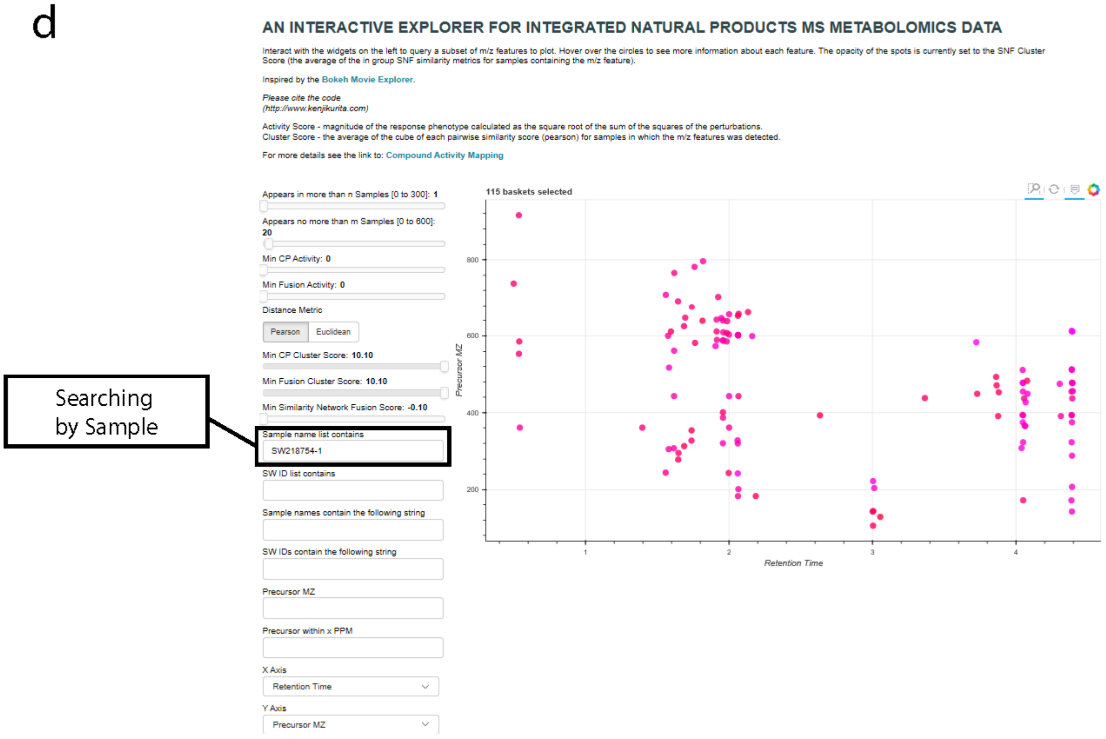

Supplementary Figure 11: Bokeh Server demonstration of functionality. (**a**) Labeled webpage for the bokeh server showing functionality and controllable components. (**b**) Minimum Occurrence set to 2 reduces the number of singleton ms features to ~4000. (**c**) Depiction of setting activity minimums in both CP and FUSION. (**d**) Searching bokeh server by sample (SW218754-1) to display all features in that fraction. General search features: Multiple codes may be searched at the same time using “, (space)” to show features in common among all searched codes. Using the text boxes and sliders on the left side of the plot, the feature list can be filtered to include only candidate features of interest. For example, increasing the Minimum Similarity Network Fusion Score slider removes features with low SNF Scores that are poorly correlated with any specific phenotype. Inserting a list of sample names into the 'Sample list name contains' text box filters the results to show only features present in a specific cluster of interest. Features are color-coded by SNF Score, from blue (strongly active) to red (weakly active). In addition, hovering the mouse over each feature in the plot reveals a pop-up window containing information about the feature including mass spectrometric data (*m/z*, rt, CCS) and distribution across the sample set.

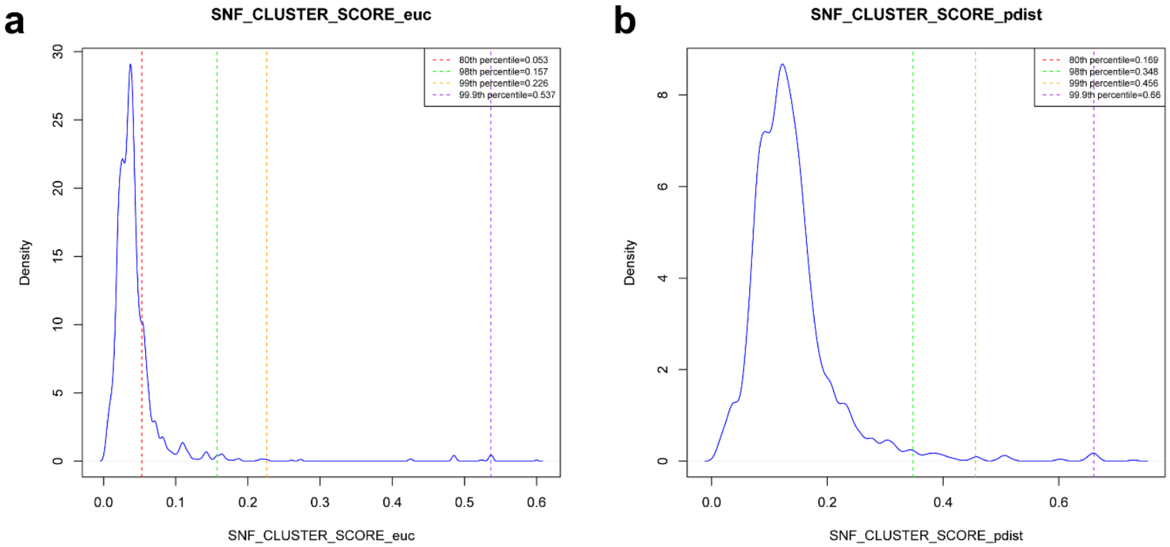

Supplementary Figure 12. Density distributions of SNF scores for unique metabolites in the natural product fraction library. SNF scores are shown as density plots using (**a**) Euclidean distance and (**b**) Pearson Correlation as the similarity metric. Percentiles are indicated by dotted lines as labeled.

a

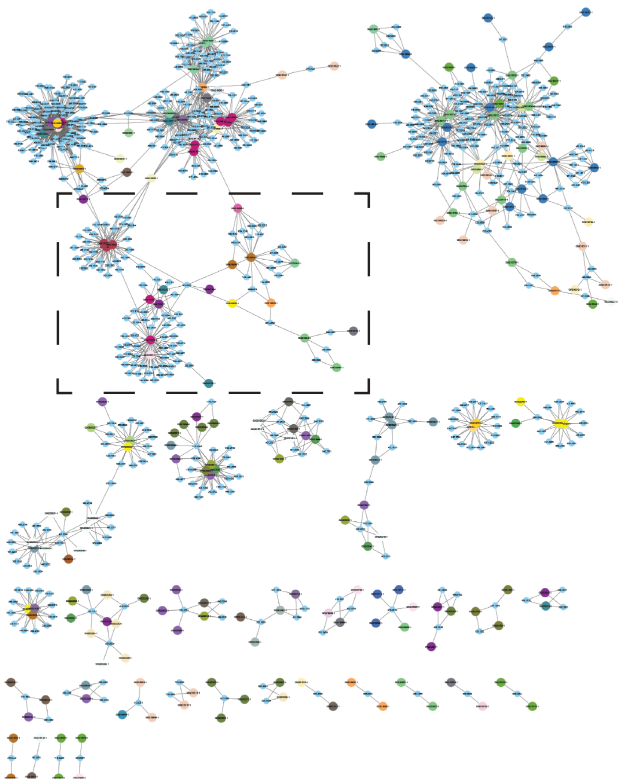

b

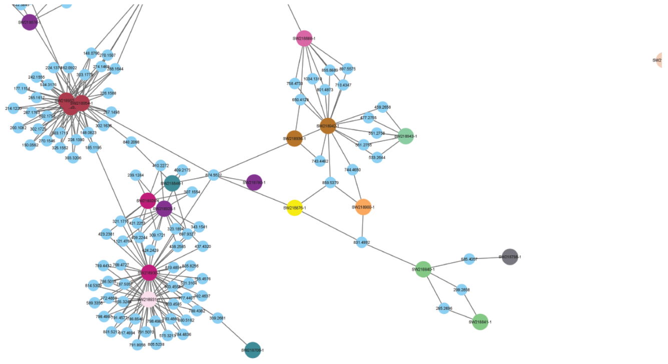

Supplementary Figure 13: Compound Activity map for combined SNF profiles and untargeted metabolomics features.
Large nodes represent extracts. Small nodes represent mass spectrometry features. Edges represent presence of mass spectrometric features in connected extracts. Only mass spectrometric features with predicted SNF scores >0.06 are included. A) Full Compound Activity Map. Large nodes color coded by AOC assignment class. B) Expansion of a representative region of the APC map with large nodes coloured by APC cluster.

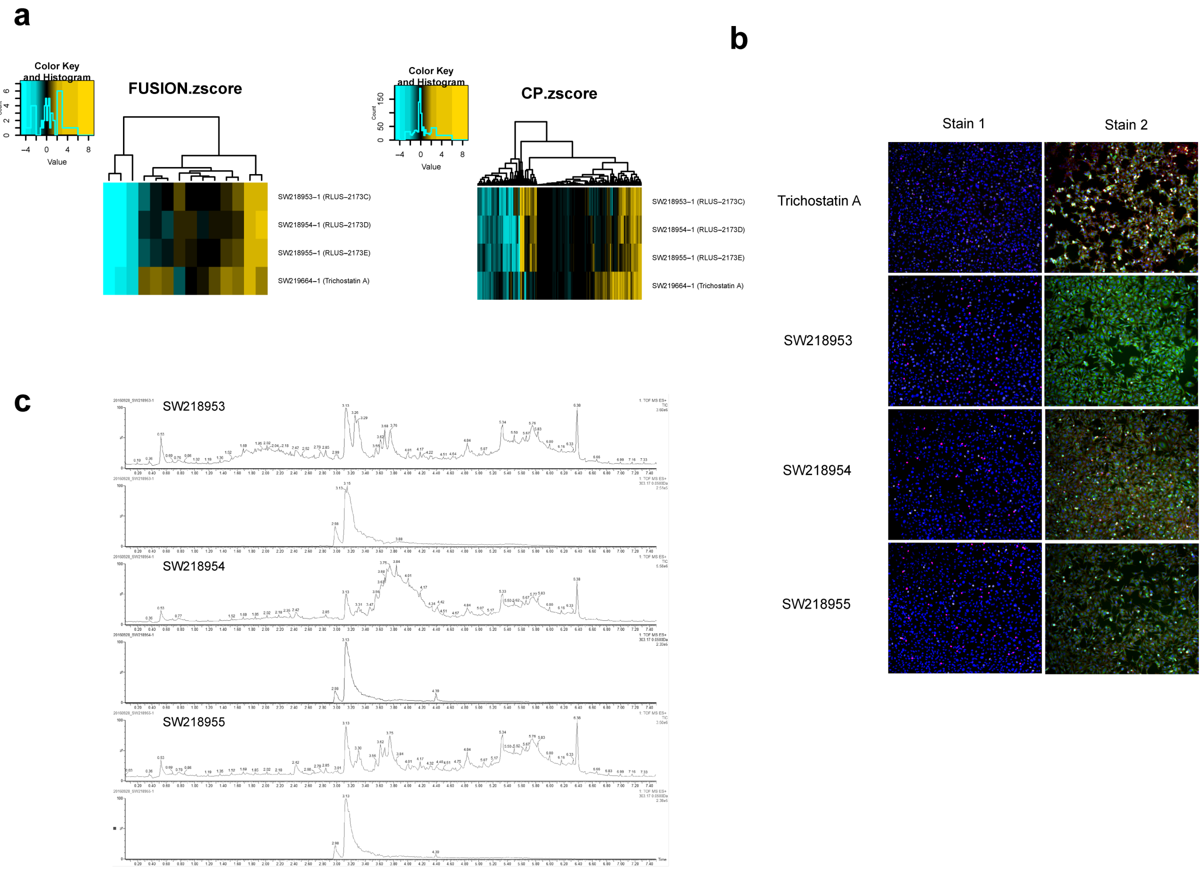

Supplementary Figure 14: Signatures from natural product fractions containing trichostatin A. (**a**) Heatmaps of FUSION z-scores and CP fingerprints, and (**b**) CP images from natural products containing trichostatin A and pure trichostatin A. (**c**) TIC and EIC 303.1703 (Trichostatin A) of SW218953, SW218954, and SW218955.

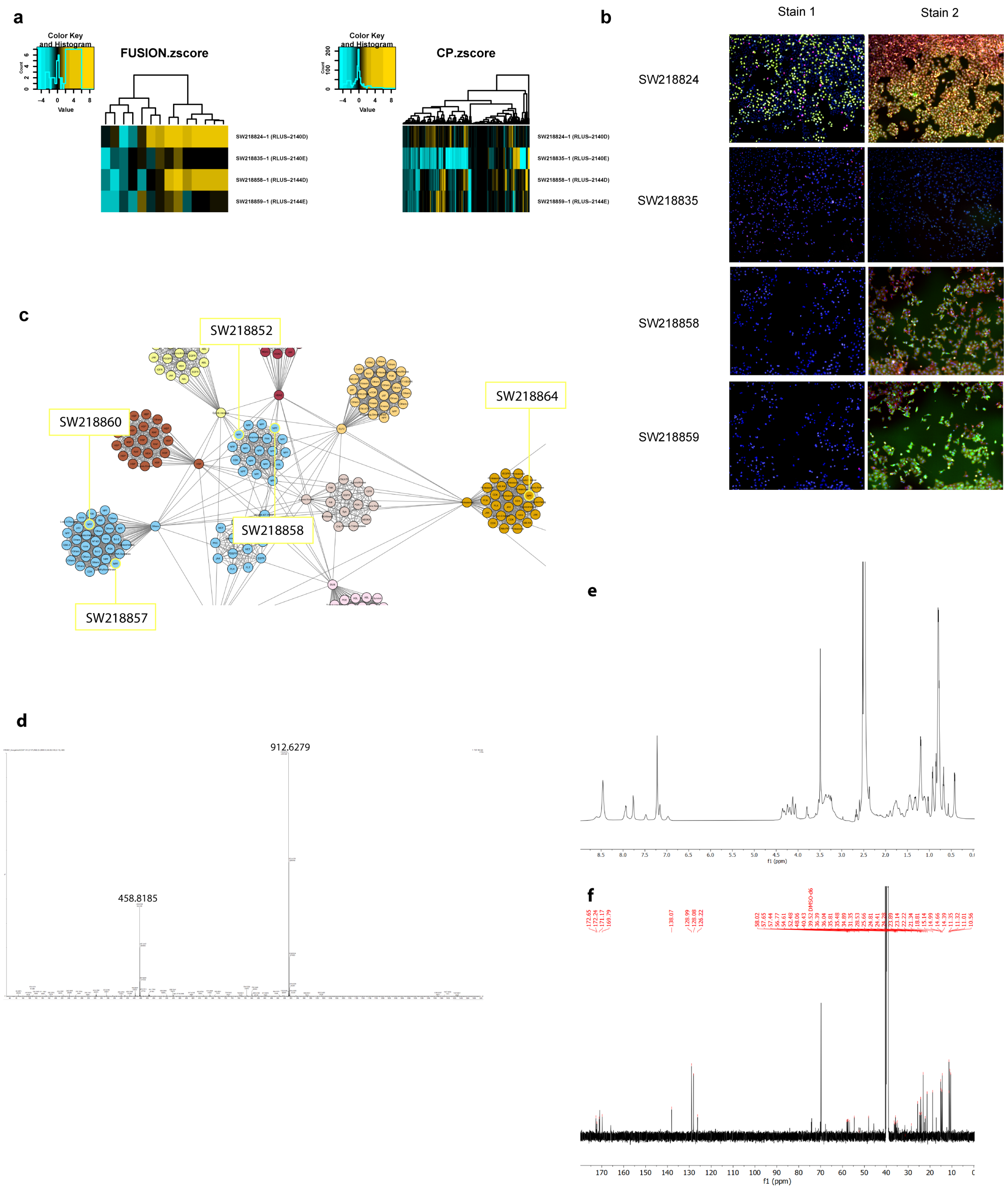

Supplementary Figure 15. Signatures from natural product fractions containing surugamide. (**a**) Heatmaps of FUSION z-scores and CP fingerprints, and (**b**) CP images from natural products containing surugamides. (**c**) SNF-Euclidean APC cluster showing proximity of surugamide-containing natural product fractions. Nodes are labeled by either target class or perturbagen type. Clusters are shown as colored if they are significantly enriched for particular target class, otherwise are colored light blue. (**d**) HRMS spectrum of surugamide A. (**e**) ^{1}H NMR spectrum and (**f**) ^{13}C NMR spectrum for surugamide A in DMSO-*d6* at 600 MHz.

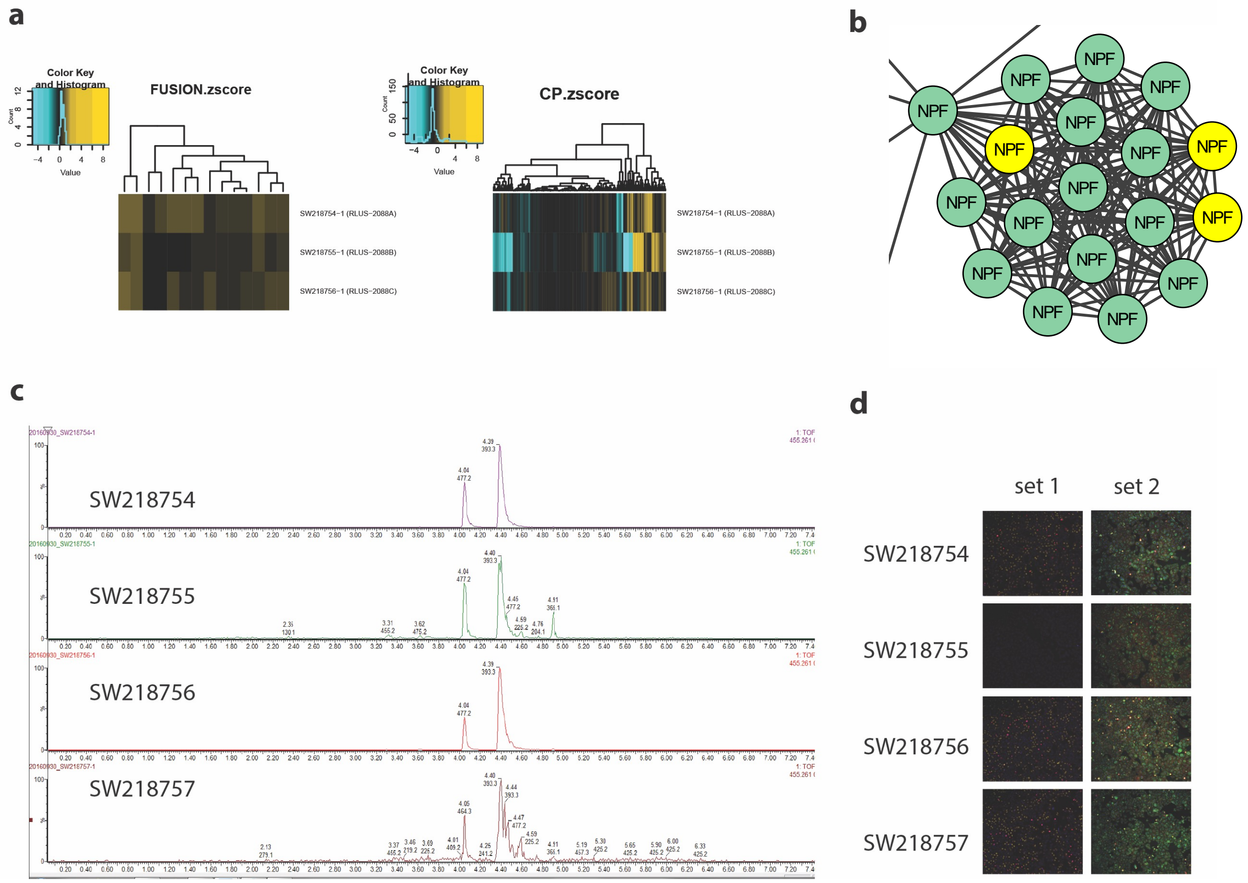

Supplementary Figure 16. Signatures from natural product fractions containing parkamycin A.

(a) Heatmaps of FUSION z-scores and CP fingerprints, (b) SNF-Euclidean APC cluster showing proximity of parkamycin A containing natural product fractions, with SW218754, SW218756 ,and SW218757 highlighted in yellow. Clusters are labeled and colored as described in Supplementary Figure 10., (c) EIC of m/z 455.2560 in adjacent fractions all containing parkamycin A (d) CP images from natural products containing parkamycin A.

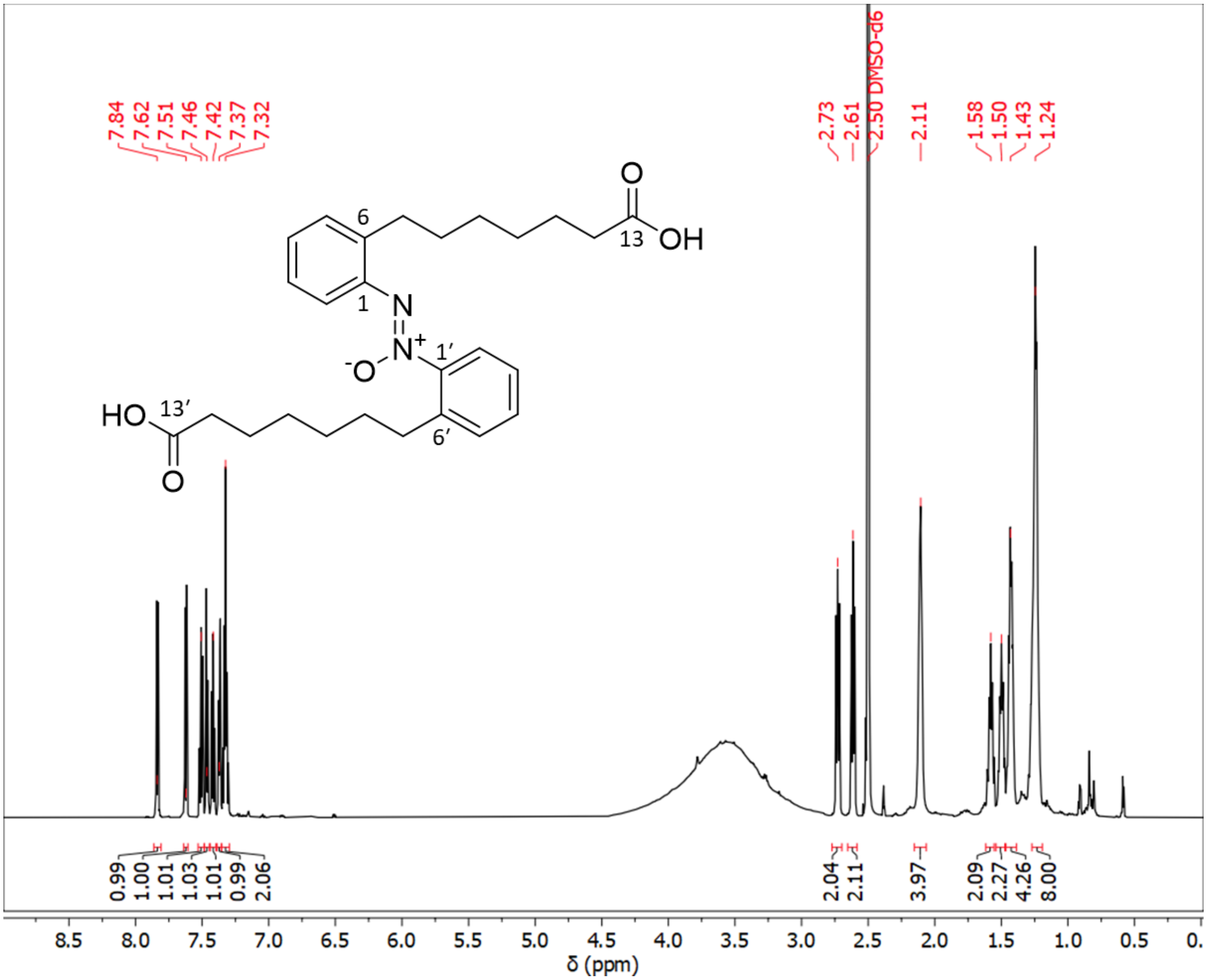

Supplementary Figure 17. ^{1}H NMR of parkamycin A.

^{1}H NMR spectrum collected at 600 MHz in DMSO-*d6*

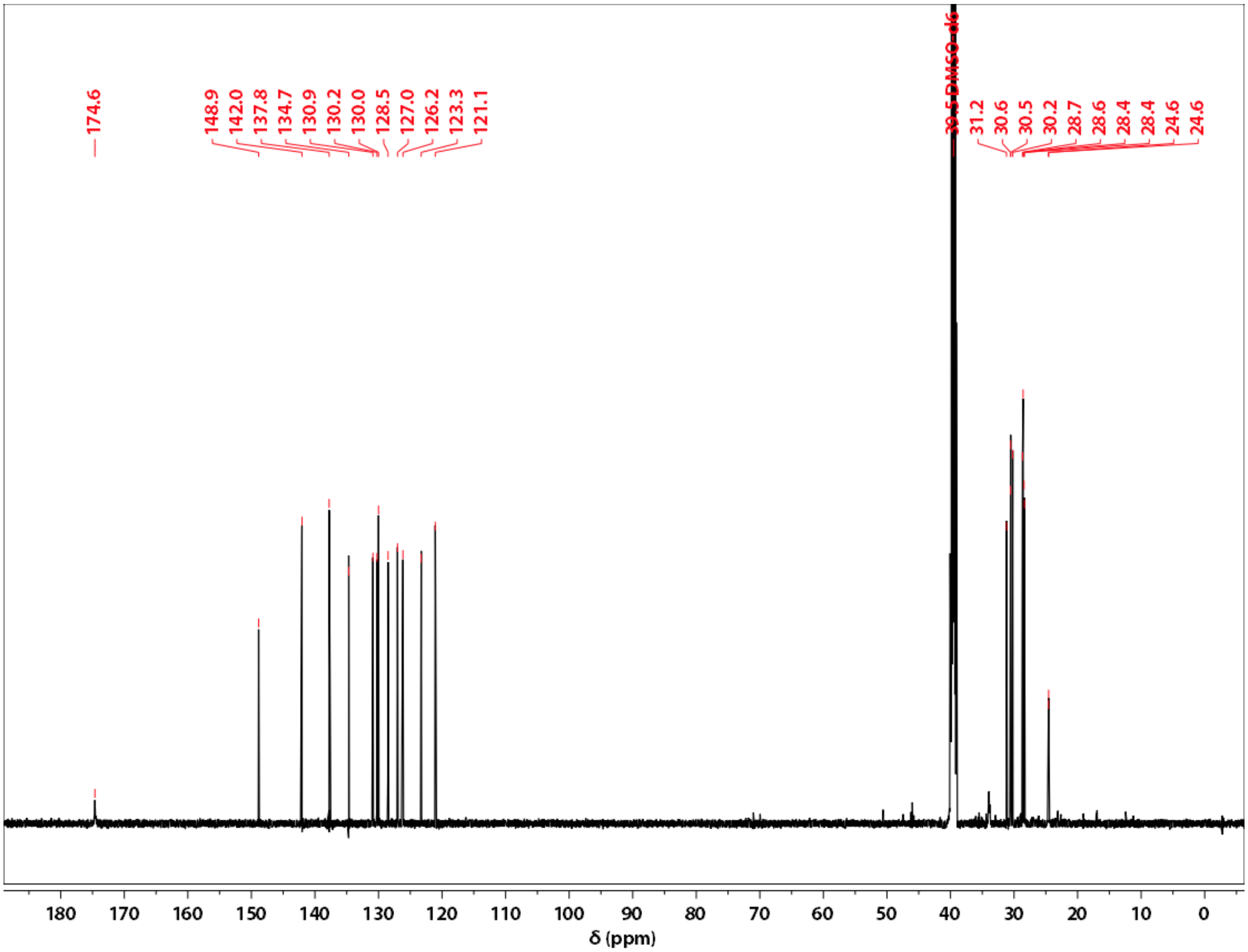

Supplementary Figure 18. ^{13}C NMR of parkamycin A.

^{13}C NMR spectrum collected at 150 MHz in DMSO-*d6*

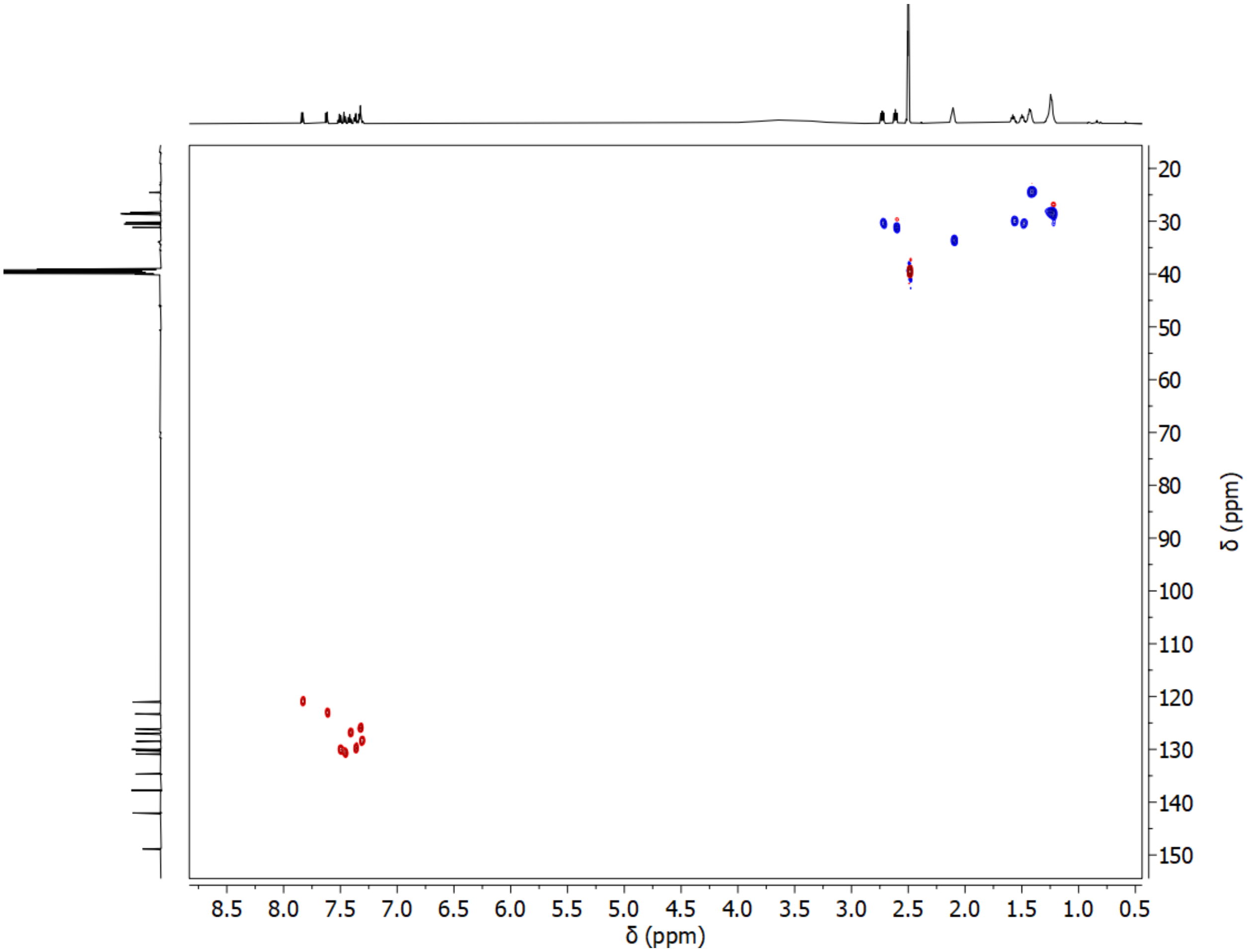

Supplementary Figure 19. HSQC NMR spectrum of parkamycin A.

HSQC NMR spectrum collected at 600 MHz in DMSO-d6

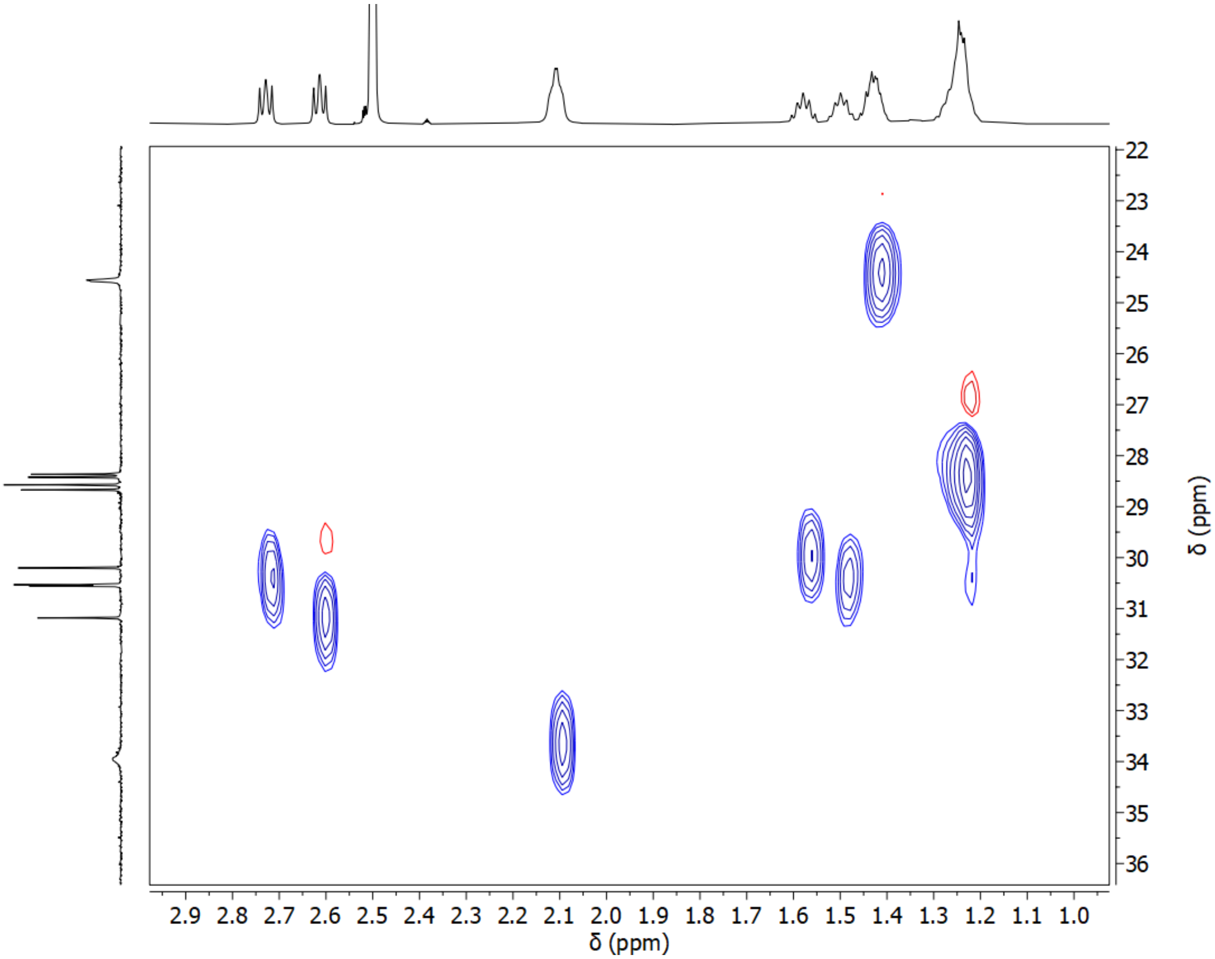

Supplementary Figure 20. Expanded HSQC NMR spectrum of parkamycin A, region 1.

Expanded HSQC NMR spectrum collected at 600 MHz in DMSO-*d6*

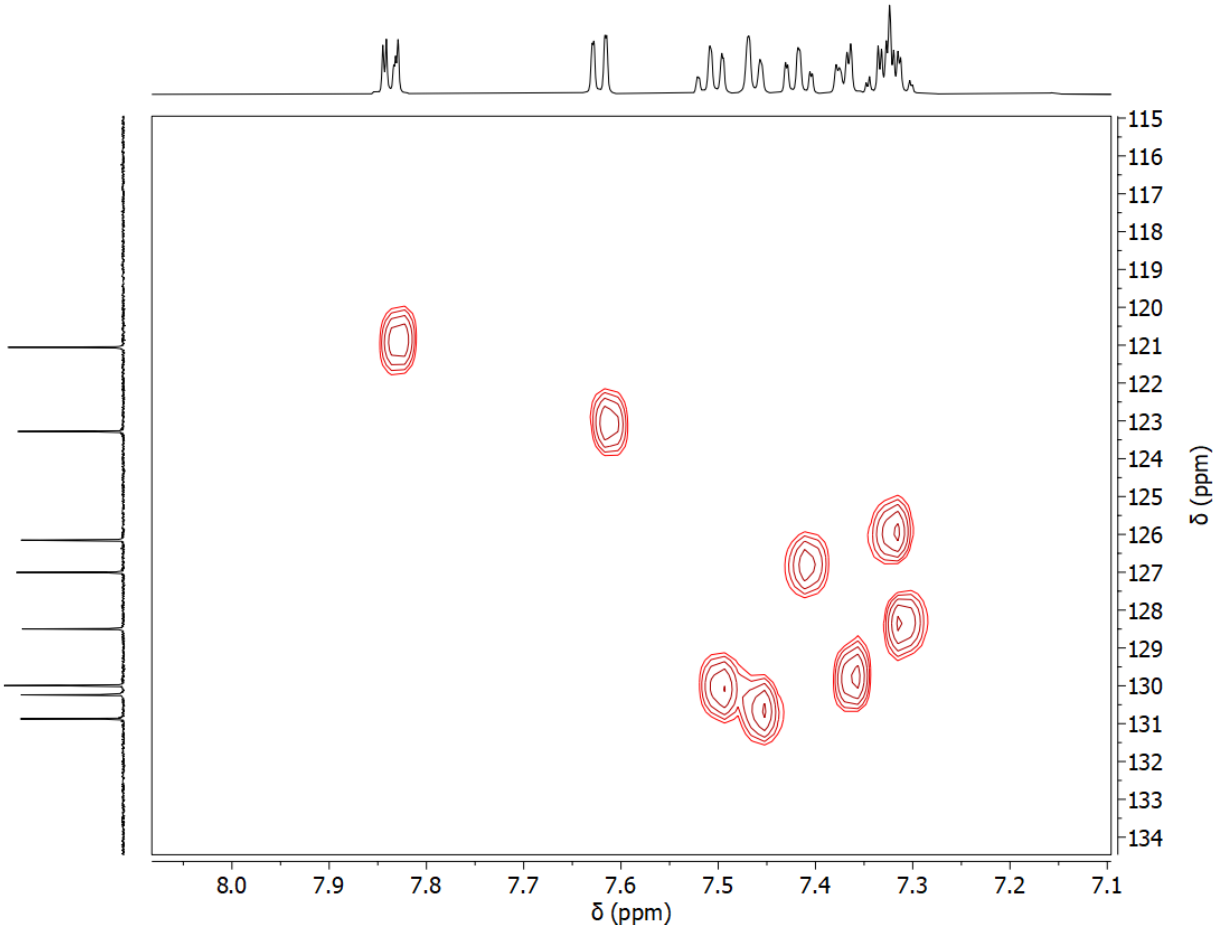

Supplementary Figure 21. Expanded HSQC NMR spectrum of parkamycin A, region 2.

Expanded HSQC NMR spectrum collected at 600 MHz in DMSO-*d6*

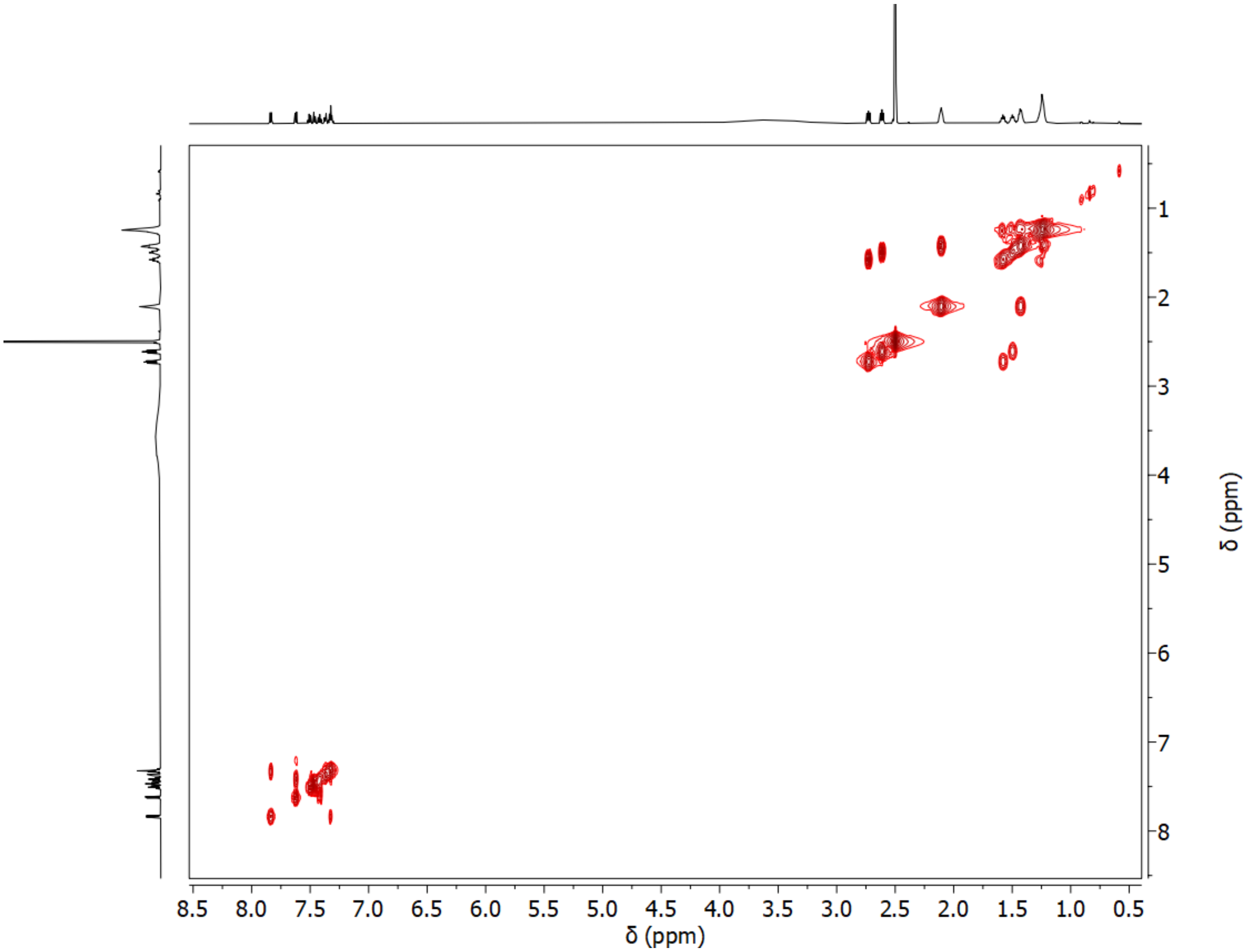

Supplementary Figure 22. COSY NMR spectrum of parkamycin A.

COSY NMR spectrum collected at 600 MHz in DMSO-*d6*

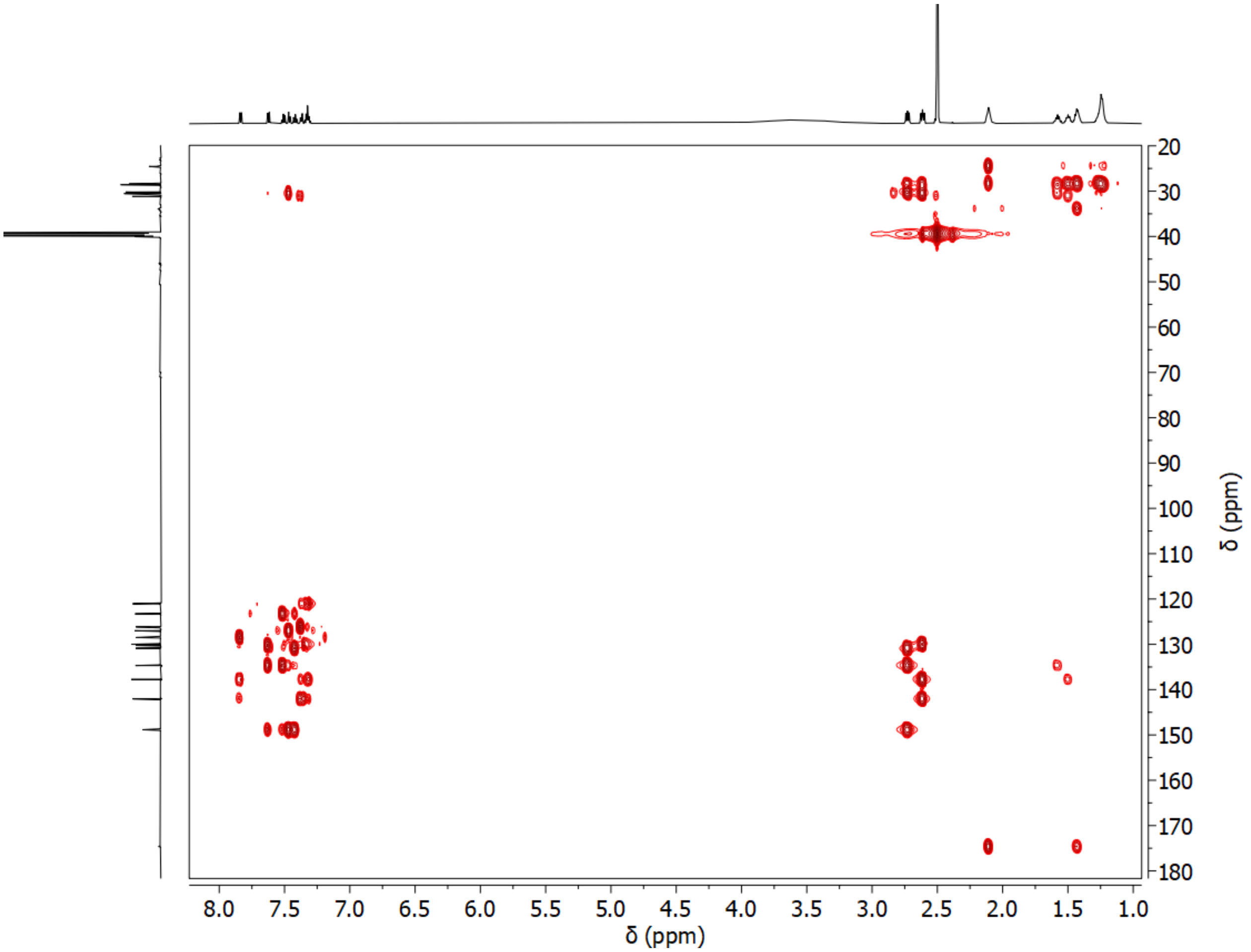

Supplementary Figure 23. HMBC NMR spectrum of parkamycin A.

HMBC NMR spectrum collected at 600 MHz in DMSO-*d6*

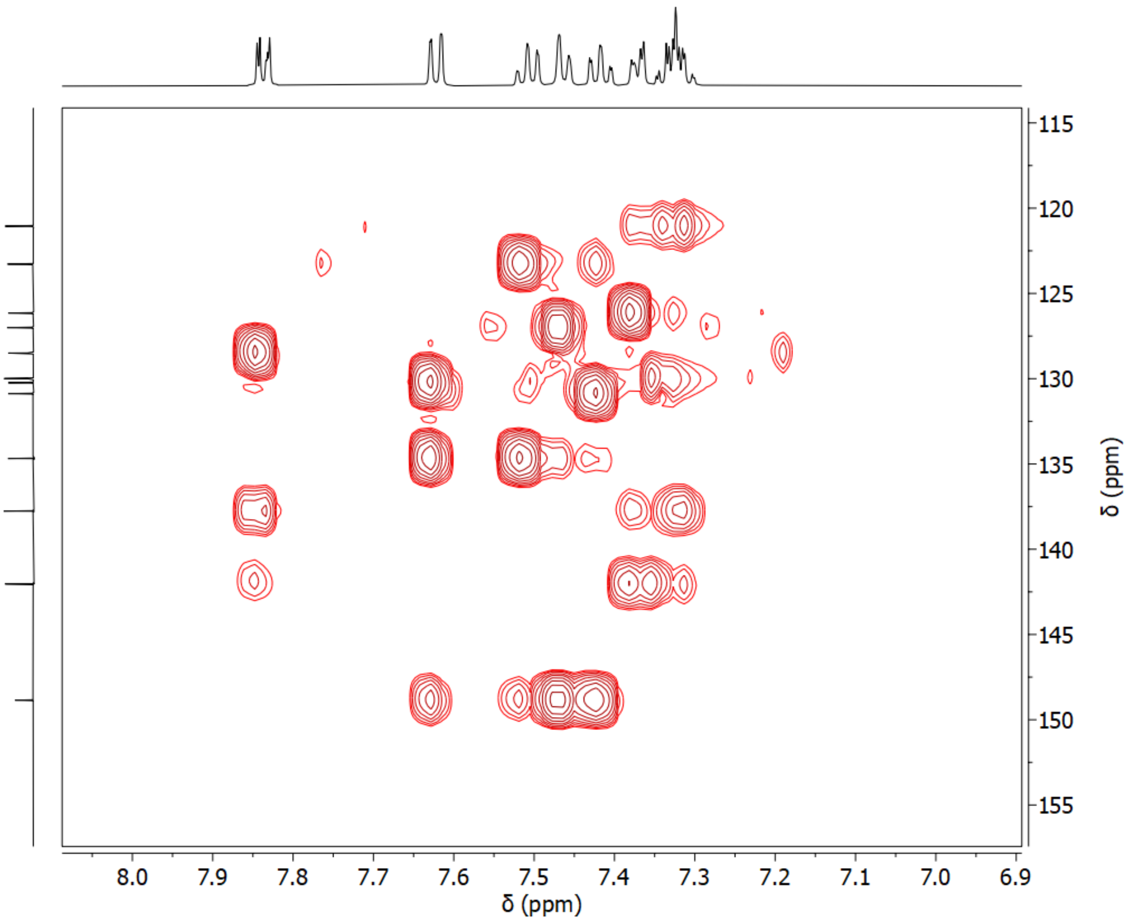

Supplementary Figure 24. Expanded HMBC NMR spectrum of parkamycin A, region 1.

Expanded HMBC NMR spectrum collected at 600 MHz in DMSO-*d6*

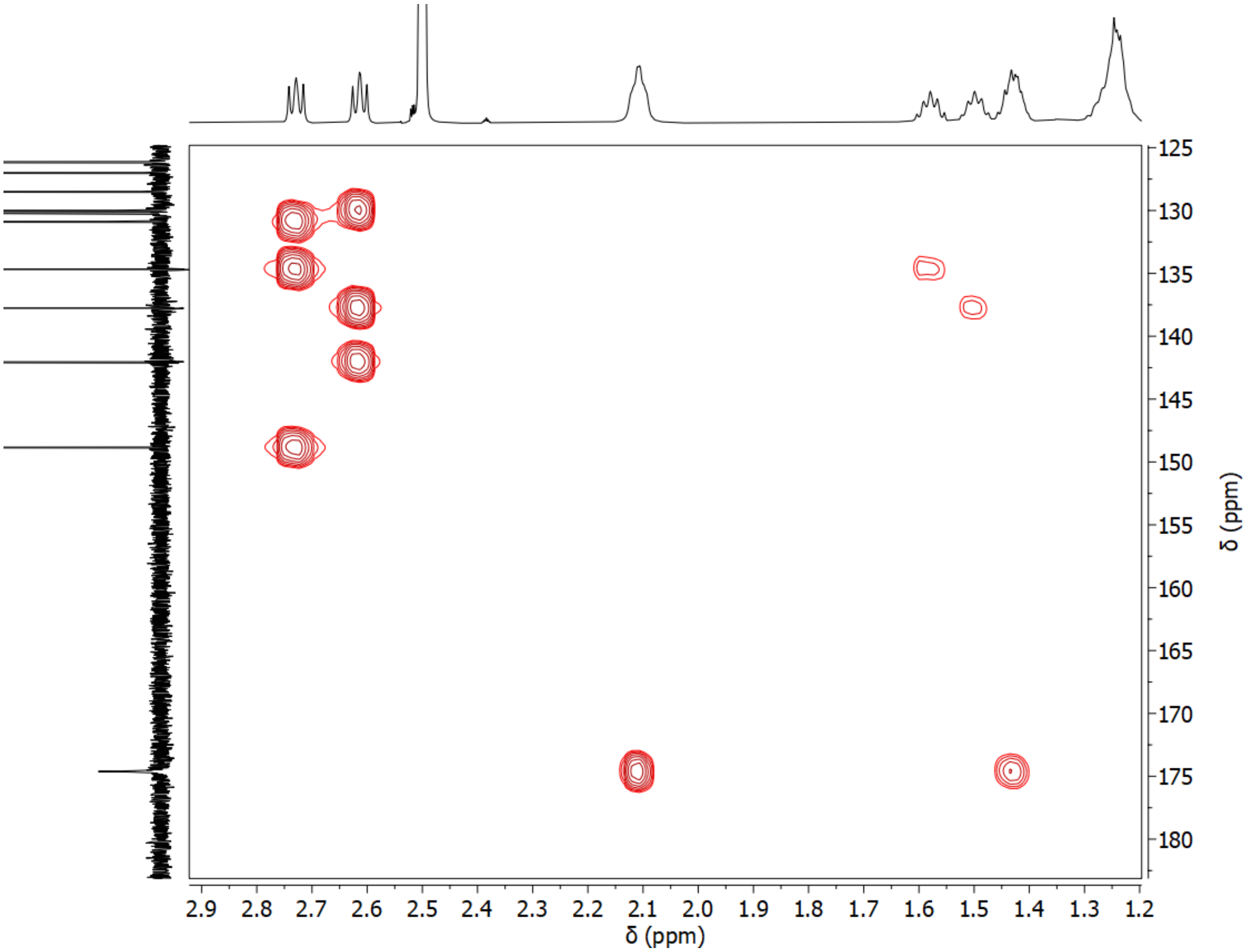

Supplementary Figure 25. Expanded HMBC NMR spectrum of parkamycin A, region 2.

Expanded HMBC NMR spectrum collected at 600 MHz in DMSO-*d6*

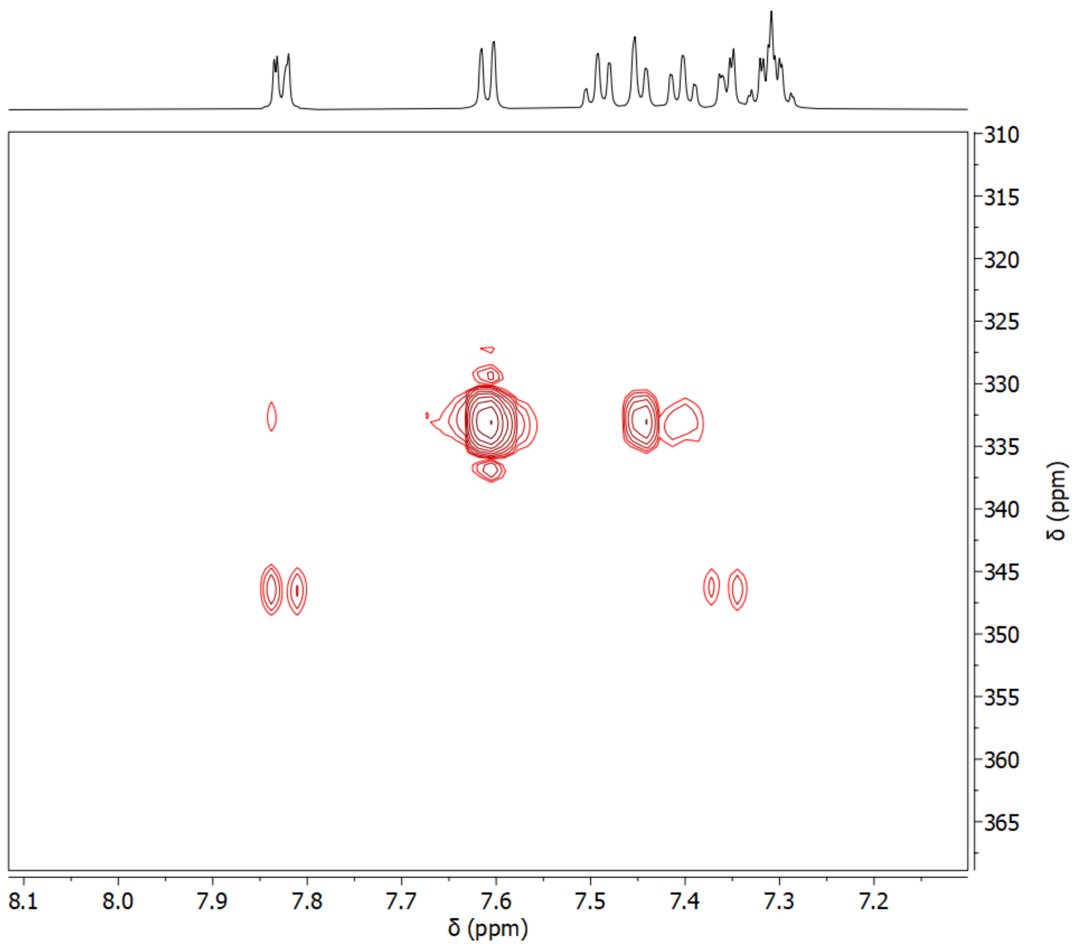

Supplementary Figure 26. ^{15}N-HMBC NMR spectrum of parkamycin A.

^{15}N-HMBC NMR spectrum collected at 600 MHz in DMSO-*d6*

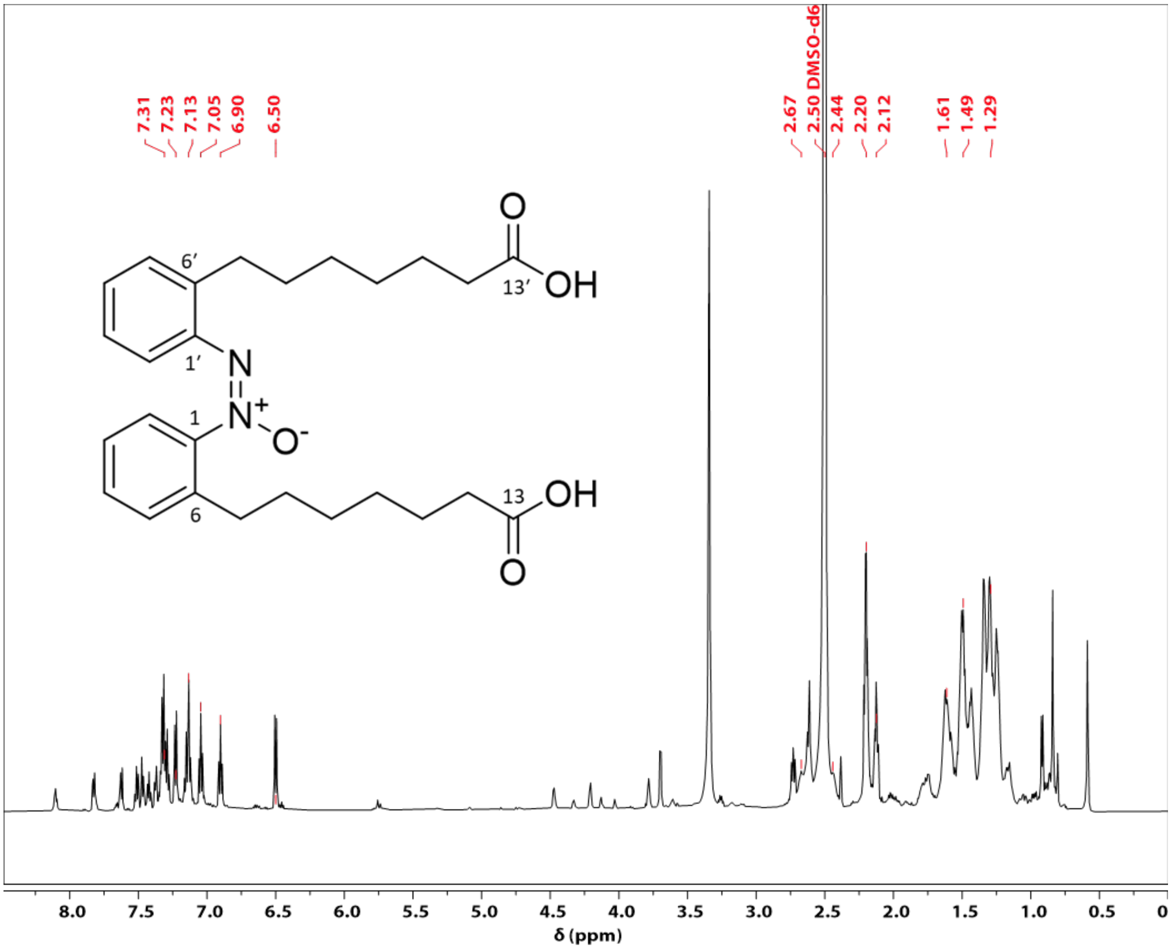

Supplementary Figure 27. ^{1}H NMR spectrum of parkamycin B (semi pure).

^{1}H NMR spectrum collected at 600 MHz in DMSO-*d6*

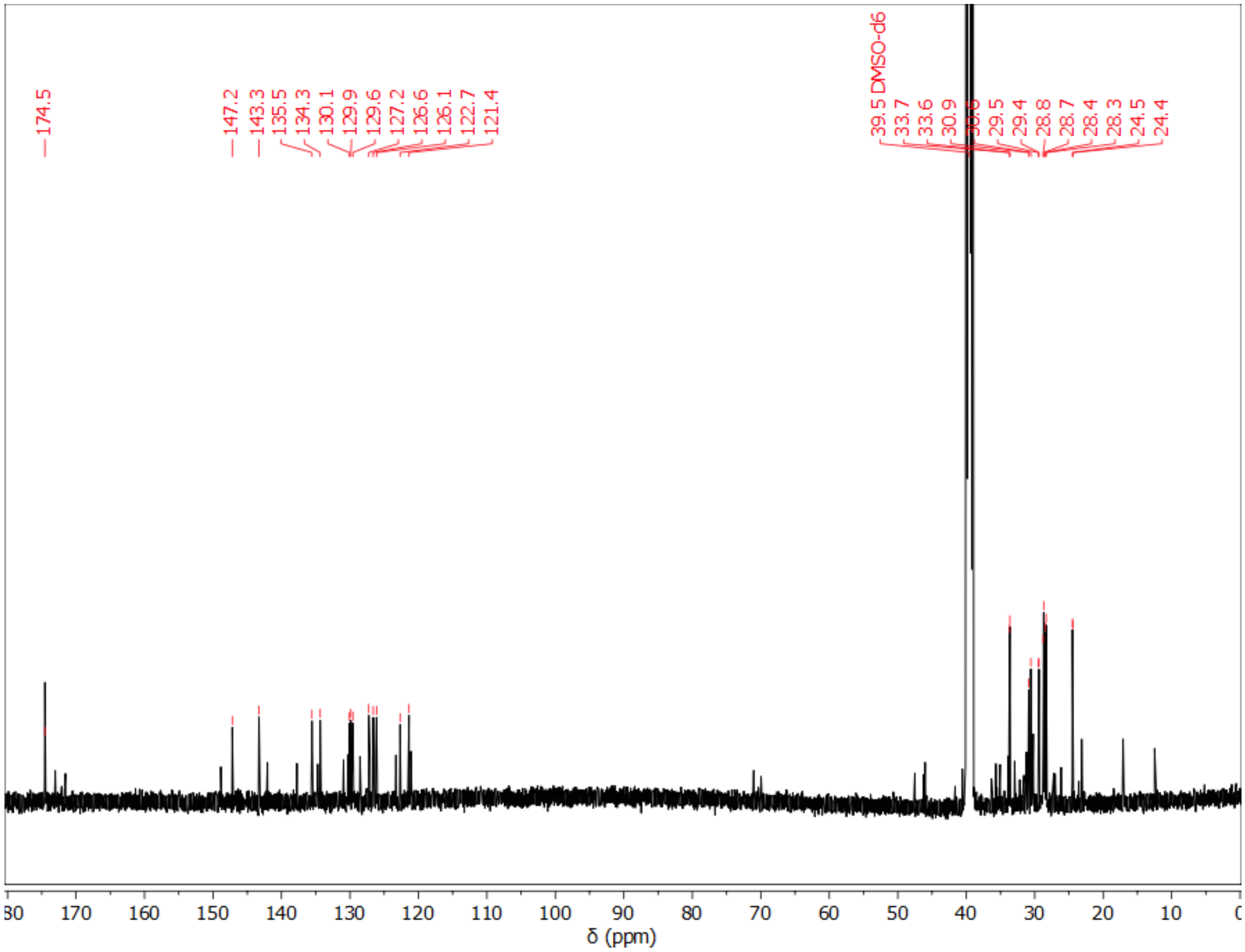

Supplementary Figure 28. ^{13}C NMR spectrum of parkamycin B (semi pure).

^{13}C NMR spectrum collected at 150 MHz in DMSO-*d6*

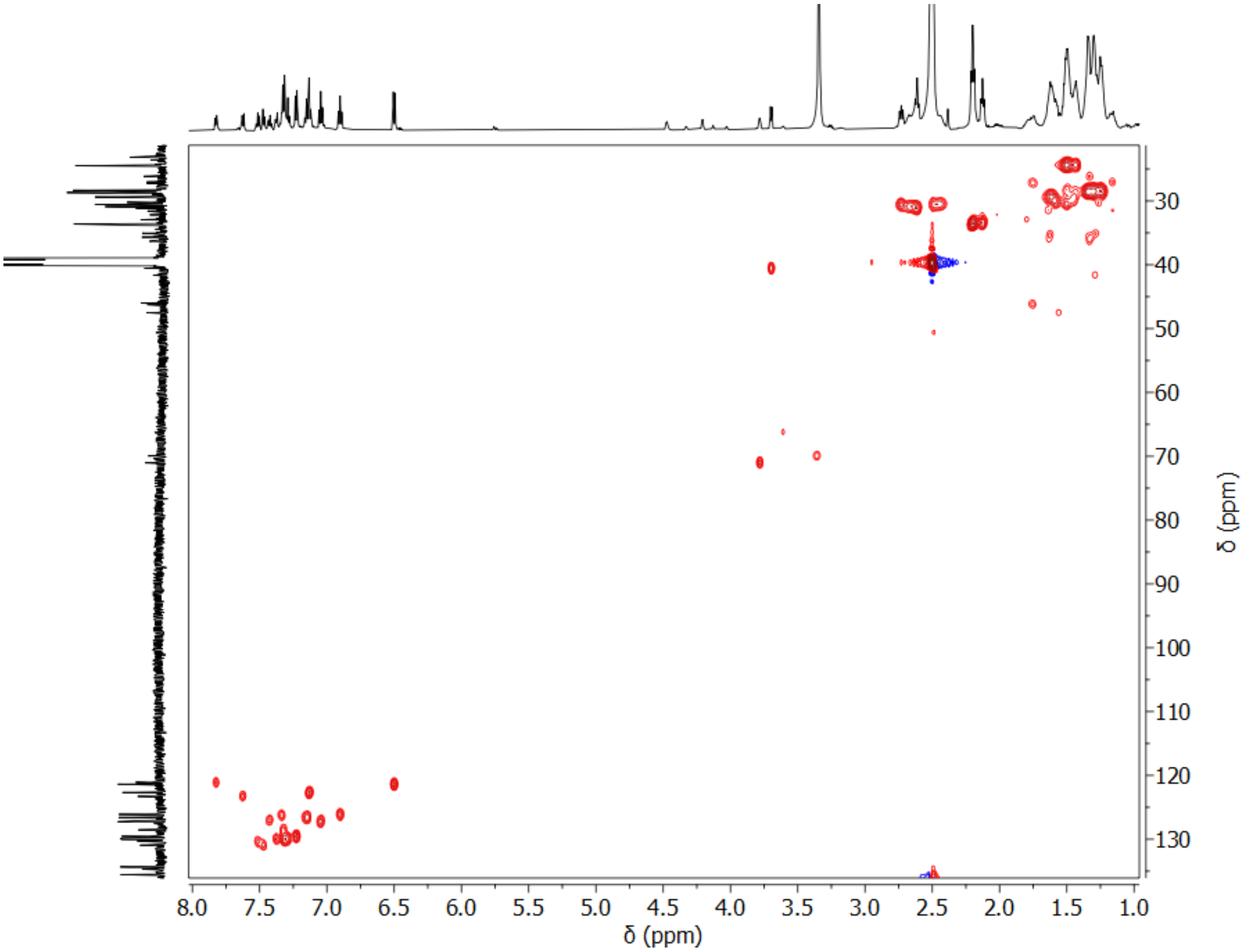

Supplementary Figure 29. HSQC NMR spectrum of parkamycin B (semi pure).

HSQC NMR spectrum collected at 600 MHz in DMSO-*d6*

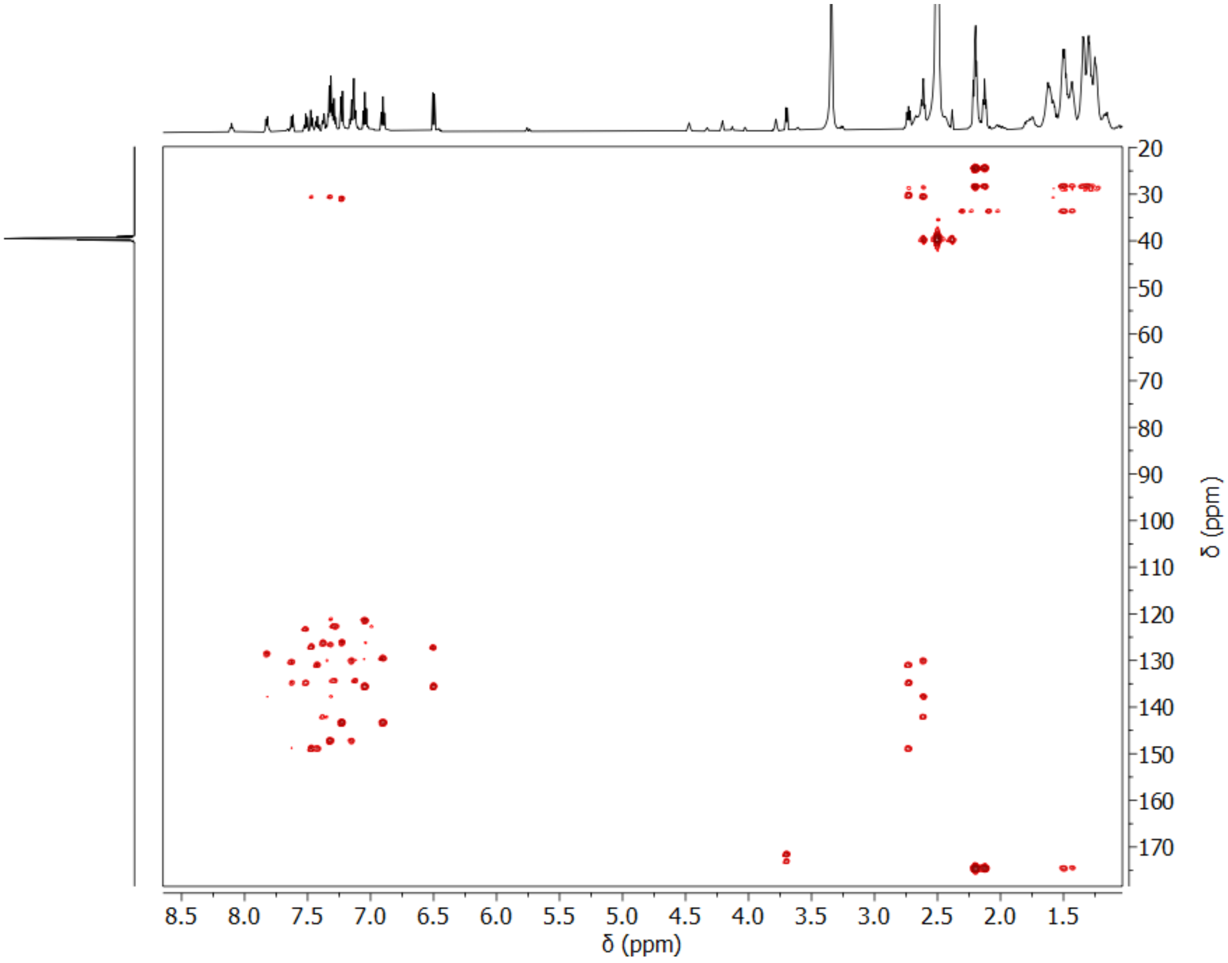

Supplementary Figure 30. HMBC NMR spectrum of parkamycin B (semi pure).

HMBC NMR spectrum collected at 600 MHz in DMSO-*d6*

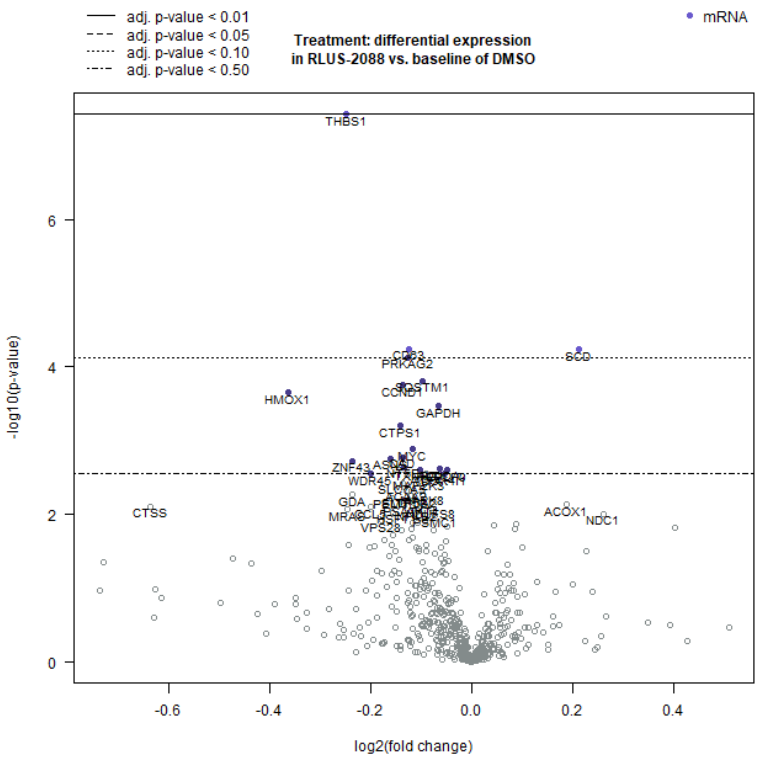

Supplementary Figure 31. Volcano plot displaying differential gene expression in the Nanostring metabolism panel after treatment with RLUS-2088 (Parkamycin A).

Differential gene expression is shown as log2 fold change of treated compared to DMSO. Horizontal lines indicate various False Discovery Rate (FDR) thresholds. Genes are colored if the resulting p-value is below the given FDR or p-value threshold. The 40 most statistically significant genes are labeled in the plot.

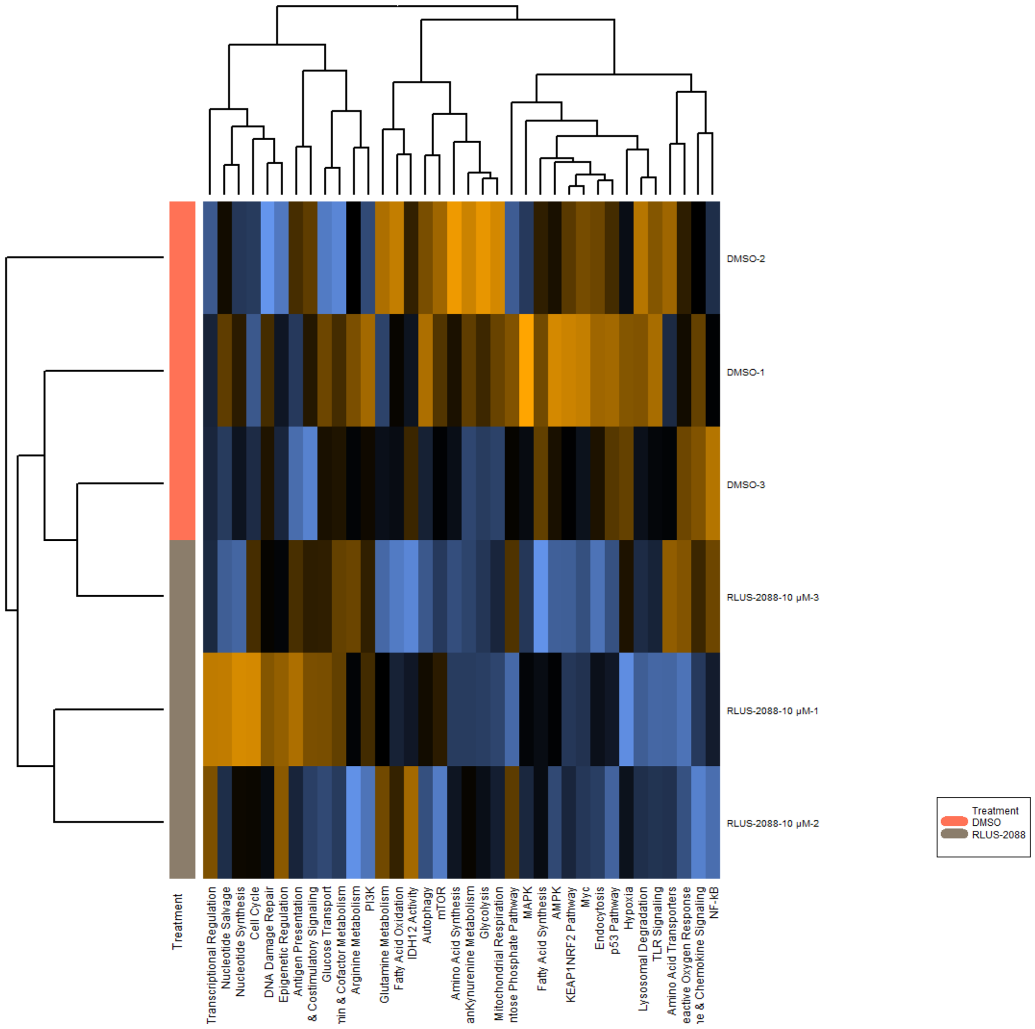

Supplementary Figure 32. Metabolic pathway analysis on gene expression data collected after parkamycin treatment.

Heatmap of pathway scores. Orange indicates upregulation; blue indicates downregulation. Scores are displayed on the same scale via a Z-transformation.

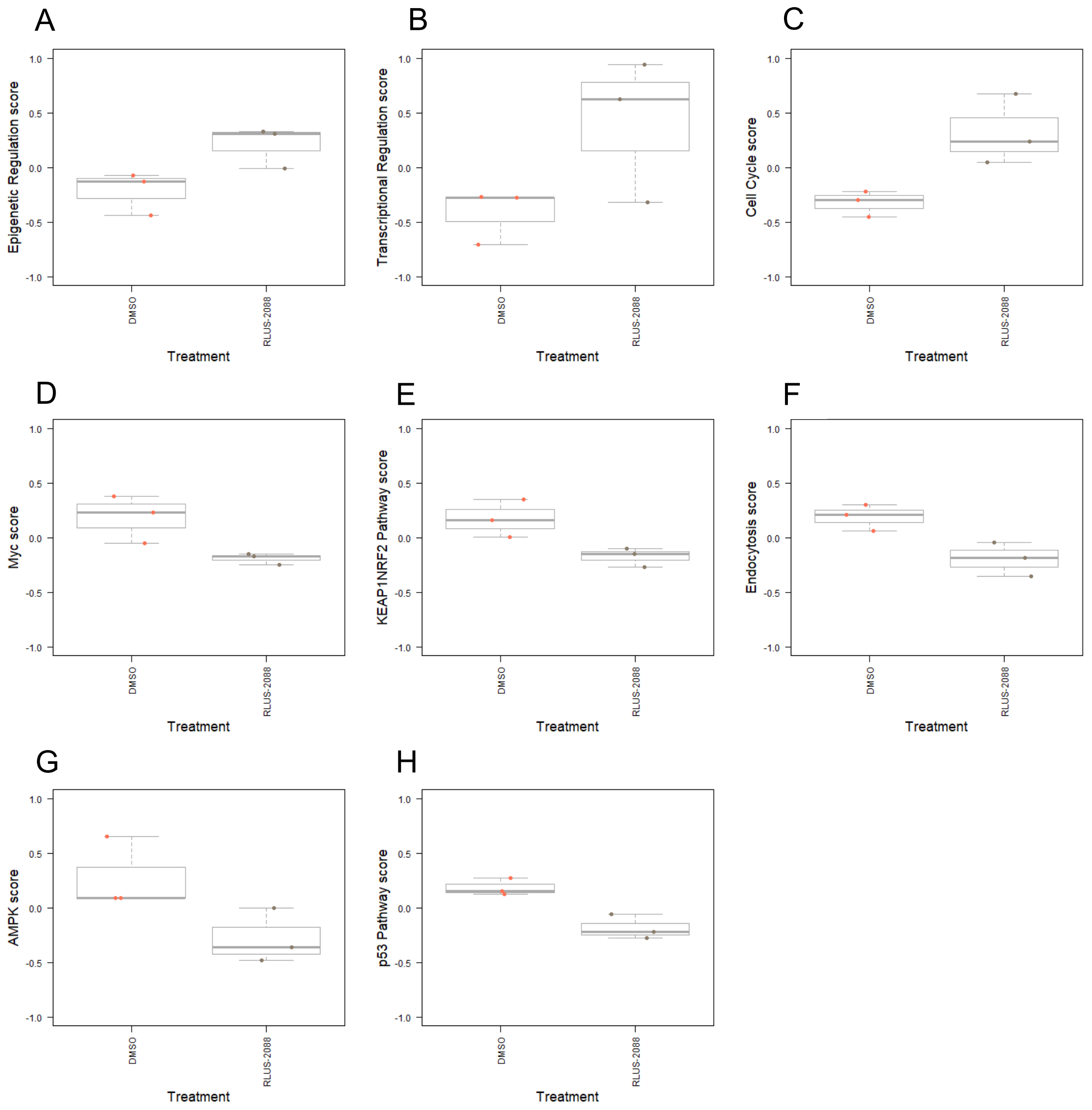

Supplementary Figure 33. Comparison of pathway scores for select pathways altered with RLUS2088 (Parkamycin-A) treatment compared to DMSO. Data shown is from n=3 independent biological replicates. A) epigenetic regulation, B) transcriptional regulation, C) cell cycle signaling, D) Myc, E) KEAP1/NRF2 pathway, F) endocytosis, G) AMPK , H) p53 pathway.

Supplementary Table 1. Selleck library target representation. The number of Selleck compounds in each target class. See Dataset S1 for a full list of compounds and annotations,

Class	Count
Others	674
none	115
I3K	33
Histamine Receptor	33
HDAC	32
AChR antagonist	26
COX	26
Adrenergic Receptor antagonist	25
Adrenergic Receptor agonist	25
DNA/RNA Synthesis	24
CDK	22
RAAS	21
PDE	20
5HTR antagonist	20
VEGFR	20
JAK	20
Topoisomerase	20
Sodium Channel	19
mTOR	19
HSP (e.g. HSP90)	18
Aurora Kinase	17
EGFR	17
Calcium Channel	17
MEK	16
Microtubule Associated	14
PARP	13
RAF	13
5HTR agonist	13
Reverse Transcriptase	12
P450 (e.g. CYP17)	12
Proteasome	12
Estrogen/progestogen Receptor_agonist	11
p38 MAPK	11
GSK-3	11
EGFR_ERBB	10
Potassium Channel	10
Androgen Receptor antagonist	10
Wnt/beta-catenin	10
IGF-1R	9
ATPase	9

TGF-beta/Smad	9
PPAR	9
ABL	9
Histone Methyltransferase	9
Epigenetic Reader Domain	9
c-Met	9
Dopamine Receptor antagonist	9
Bcl-2	8
HMG-CoA Reductase	8
IkB/IKK	8
DUB	8
Akt	8
PDGFR	7
GluR agonist	7
Gamma-secretase	7
GluR antagonist	7
PLK	7
Dehydrogenase	7
FAK	7
ATM/ATR	7
HIV Protease	6
Syk	6
DPP-4	6
STAT	6
Estrogen/progestogen Receptor_antagonist	6
Transferase	6
Src	6
PKC	6
AChR agonist	6
Hedgehog/Smoothened	6
p53	6
Kinesin	6
CFTR	6
Autophagy	5
c-Kit	5
Endothelin Receptor	5
SERT/NET	5
Aromatase	5
FLT3	5
SERT	5
ROCK	5
Caspase	5
VEGFR_cKIT_PDGFR	5
Carbonic Anhydrase	5

MMP	5
Dopamine Receptor agonist	5
NF-kB	5
TNF-alpha	5
Integrase	5
DNA Methyltransferase	5
GPR	5
Sirtuin	4
Histone demethylases	4
HCV Protease	4
Opioid Receptor agonist	4
Factor Xa	4
E3 Ligase	4
GABA Receptor antagonist	4
Cysteine Protease	4
Opioid Receptor antagonist	4
PDK-1	4
BTK	4
MET_VEGFR	4
Proton Pump	4
P2 Receptor	4
Pim	4
CETP	4
Estrogen/progestogen Receptor_SERM	4
IAP	4
OX Receptor	4
Chk	4
Cannabinoid Receptor agonist	4
AMPK	4
Serine Protease	3
DHFR	3
BMP	3
Androgen Receptor agonist	3
FAAH	3
FGFR	3
SGLT	3
S1P Receptor	3
LRRK2	3
ALK	3
Rac	3
Mdm2	3
CRM1	3
Hydroxylase	3
Rho	3

p97	3
5-alpha Reductase	3
HIF	3
MAO	3
JNK	3
S6 Kinase	3
Telomerase	3
ELF4	2
Liver X Receptor	2
PERK	2
CaSR	2
VDA	2
P-gp	2
Beta Amyloid	2
VEGFR_PDGFR	2
ERK	2
LPA Receptor	2
Dynamin	2
ELF2	2
HER2	2
PKA	2
Cannabinoid Receptor antagonist	2
PAK	2
cAMP	2
ERBB	2
CSF-1R	2
GABA Receptor agonist	2
IDO	2
TRPV	2
FXR	2
Phospholipase (e.g. PLA)	2
Ferroptosis	2
Cathepsin K	2
E2	1
DDP-4	1
MTH	1
NOD1	1
MNK	1
Survivin	1
IDH2	1
CXCR	1
PDHK	1
BMI	1
BET	1

Fo-ATPase	1
Tie-2	1
gp120/CD4	1
MBT	1
Wee1	1
Histone Acetyltransferase	1
Axl	1
Substance P	1
AAAD/DOPA decarboxylase inhibitor	1
MT Receptor	1
Vasopressin Receptor	1
ribonucleotide reductase	1
c-Myc	1
APE	1
PAFR	1
IL Receptor	1
Ras	1
Ephrin receptor	1
GDP/GTP Exchange Factor Inhibitor	1
Procollagen C Proteinase	1
CCR	1
Integrin	1
Notch	1
Arp2/3	1
E1 Activating	1
DNA-PK	1
ATGL	1
Ftase	1
Phosphorylase	1

Supplementary Table 2: K-means cluster membership in FUSION and CP for compounds in the top 30 largest target classes.

SWID	Other_ ID	Pathway	Cmpd.Name	Fusion_ kmeans	CP_kmeans
SW220242-1	S2852	5HTR.agonist	BRL-54443	1	26
SW197244-4	S2025	5HTR.agonist	Urapidil HCl	6	26
SW197596-2	S1385	5HTR.agonist	Mosapride Citrate	7	22
SW197624-3	S1432	5HTR.agonist	Sumatriptan Succinate	7	22
SW219416-1	S1488	5HTR.agonist	Naratriptan	7	8
SW219573-1	S4109	5HTR.agonist	Lorcaserin HCl	7	10
SW219882-1	S1436	5HTR.agonist	Tianeptine sodium	7	12
SW197762-2	S1649	5HTR.agonist	Zolmitriptan	21	3
SW219198-2	S2875	5HTR.agonist	Prucalopride	21	25
SW197669-2	S1607	5HTR.agonist	Rizatriptan Benzoate	23	18
SW197521-3	S1975	5HTR.agonist	Aripiprazole	25	14
SW219418-1	S2096	5HTR.agonist	Almotriptan Malate	28	26
SW220149-1	S3180	5HTR.agonist	Eletriptan HBr	28	3
SW100810-5	S1390	5HTR.antagonist	Ondansetron HCl	1	26
SW219360-1	S8021	5HTR.antagonist	Vortioxetine (Lu AA21004) HBr	3	20
SW219880-1	S4053	5HTR.antagonist	Sertraline HCl	3	20
SW196337-3	S3183	5HTR.antagonist	Amitriptyline HCl	8	24
SW196384-4	S2541	5HTR.antagonist	Clomipramine HCl	8	22
SW219879-1	S1283	5HTR.antagonist	Asenapine	8	5
SW219571-1	S2663	5HTR.antagonist	WAY-100635 Maleate	19	30
SW219811-1	S2865	5HTR.antagonist	VUF 10166	19	16
SW219375-1	S2677	5HTR.antagonist	BRL-15572	20	14
SW220247-1	S2459	5HTR.antagonist	Clozapine	20	1
SW220018-1	S2894	5HTR.antagonist	SB742457	21	12
SW220129-1	S2849	5HTR.antagonist	SB269970 HCl	21	30
SW198927-2	S1898	5HTR.antagonist	Tropisetron	23	18
SW219157-1	S8010	5HTR.antagonist	PRX-08066 Maleic acid	23	27
SW219941-1	S2856	5HTR.antagonist	SB271046	25	20
SW196882-3	S2232	5HTR.antagonist	Ketanserin	26	11
SW219177-1	S1243	5HTR.antagonist	Agomelatine	26	13
SW219736-1	S2698	5HTR.antagonist	RS-127445	26	16
SW220248-1	S2493	5HTR.antagonist	Olanzapine	26	11
SW197348-4	S1615	5HTR.antagonist	Risperidone	28	12
SW219131-1	S2497	AChR.antagonist	Pancuronium dibromide	1	25
SW219141-1	S3048	AChR.antagonist	Solifenacin succinate	3	4
SW102176-4	S2054	AChR.antagonist	Orphenadrine Citrate	6	30
SW196544-3	S2471	AChR.antagonist	Gallamine Triethiodide	7	1
SW196713-3	S4034	AChR.antagonist	Diphemanil Methylsulfate	7	25
SW197005-3	S4027	AChR.antagonist	Flavoxate HCl	7	3
SW219039-1	S4024	AChR.antagonist	Homatropine Methylbromide	7	25

SW219041-1	S2547	AChR.antagonist	Tiotropium Bromide hydrate	7	11
SW220294-1	S2130	AChR.antagonist	Atropine	7	8
SW219082-1	S4025	AChR.antagonist	Homatropine Bromide	12	25
SW219426-1	S3144	AChR.antagonist	Darifenacin HBr	12	24
SW219846-1	S2659	AChR.antagonist	5-hydroxymethyl Tolterodine (PNU 200577; 5-HMT; 5-HM)	12	17
SW196970-2	S1285	AChR.antagonist	Biperiden HCl	15	14
SW197495-2	S2550	AChR.antagonist	Tolterodine tartrate	15	14
SW196787-4	S3117	AChR.antagonist	Oxybutynin chloride	16	4
SW196691-3	S1913	AChR.antagonist	Tropicamide	23	18
SW199029-2	S1608	AChR.antagonist	Pyridostigmine Bromide	23	26
SW199663-2	S2087	AChR.antagonist	Rivastigmine Tartrate	23	26
SW219290-1	S2240	AChR.antagonist	Fesoterodine Fumarate	24	8
SW199343-2	S2508	AChR.antagonist	Scopolamine HBr	26	30
SW219038-1	S1978	AChR.antagonist	Methscopolamine	26	22
SW219548-1	S4014	AChR.antagonist	Hyoscyamine	26	1
SW219130-1	S1397	AChR.antagonist	Rocuronium Bromide	28	20
SW219146-1	S2549	AChR.antagonist	Trospium chloride	28	18
SW219176-1	S4031	AChR.antagonist	Aclidinium Bromide	28	26
SW219209-1	S3047	AChR.antagonist	Otilonium Bromide	29	9
SW220145-1	S3083	Adrenergic.Receptor.agonist	Indacaterol Maleate	3	4
SW199659-2	S2020	Adrenergic.Receptor.agonist	Formoterol Hemifumarate	6	16
SW196632-3	S2495	Adrenergic.Receptor.agonist	Oxymetazoline HCl	7	25
SW197098-3	S4043	Adrenergic.Receptor.agonist	Tetrahydrozoline HCl	7	11
SW197214-4	S2438	Adrenergic.Receptor.agonist	Synephrine HCl	7	8
SW219274-1	S2522	Adrenergic.Receptor.agonist	L-Adrenaline	7	25
SW219274-2	S2521	Adrenergic.Receptor.agonist	Epinephrine Bitartrate	7	11
SW196701-3	S2519	Adrenergic.Receptor.agonist	Naphazoline HCl	12	23
SW197212-2	S2566	Adrenergic.Receptor.agonist	Isoprenaline HCl	12	11
SW197214-5	S2362	Adrenergic.Receptor.agonist	Synephrine	13	25
SW219274-3	S3061	Adrenergic.Receptor.agonist	Epinephrine HCl	13	25
SW219607-1	S3075	Adrenergic.Receptor.agonist	Dexmedetomidine	13	1
SW196915-3	S4277	Adrenergic.Receptor.agonist	Bambuterol HCl	16	20

SW197048-3	S2516	Adrenergic.Receptor.agonist	Xylazine HCl	20	30
SW197681-3	S2458	Adrenergic.Receptor.agonist	Clonidine HCl	20	11
SW219096-1	S2545	Adrenergic.Receptor.agonist	Scopine	20	1
SW220261-1	S3185	Adrenergic.Receptor.agonist	Adrenalone HCl	20	25
SW220301-1	S4009	Adrenergic.Receptor.agonist	Mirabegron	20	26
SW199068-2	S1437	Adrenergic.Receptor.agonist	Tizanidine HCl	21	22
SW219239-1	S3060	Adrenergic.Receptor.agonist	Medetomidine HCl	24	3
SW197044-3	S4065	Adrenergic.Receptor.agonist	Guanabenz Acetate	26	30
SW219275-1	S2533	Adrenergic.Receptor.agonist	Ritodrine HCl	26	1
SW219446-1	S2569	Adrenergic.Receptor.agonist	Phenylephrine HCl	28	3
SW219456-1	S2092	Adrenergic.Receptor.agonist	Detomidine HCl	28	8
SW219607-2	S2090	Adrenergic.Receptor.agonist	Dexmedetomidine HCl (Precedex)	28	26
SW197547-3	S1831	Adrenergic.Receptor.antagonist	Carvedilol	3	2
SW219269-1	S1549	Adrenergic.Receptor.antagonist	Nebivolol	3	16
SW199199-2	S1856	Adrenergic.Receptor.antagonist	Metoprolol Tartrate	6	26
SW196705-3	S4010	Adrenergic.Receptor.antagonist	Acebutolol HCl	7	26
SW196913-4	S1409	Adrenergic.Receptor.antagonist	Alfuzosin HCl	7	18
SW219414-1	S4076	Adrenergic.Receptor.antagonist	Propranolol HCl	7	24
SW196892-3	S2517	Adrenergic.Receptor.antagonist	Maprotiline HCl	8	27
SW197099-3	S1324	Adrenergic.Receptor.antagonist	Doxazosin Mesylate	8	5
SW219365-1	S2691	Adrenergic.Receptor.antagonist	BMY 7378	12	24
SW196595-3	S4291	Adrenergic.Receptor.antagonist	Labetalol HCl	15	22
SW197155-3	S4278	Adrenergic.Receptor.antagonist	Carteolol HCl	15	10
SW196909-3	S4124	Adrenergic.Receptor.antagonist	Tolazoline HCl	16	3
SW197151-3	S1206	Adrenergic.Receptor.antagonist	Bisoprolol fumarate	20	26
SW197352-4	S2509	Adrenergic.Receptor.antagonist	Sotalol	20	1
SW196570-4	S2126	Adrenergic.Receptor.antagonist	Naftopidil	21	11
SW196637-3	S2038	Adrenergic.Receptor.antagonist	Phentolamine Mesylate	23	11

SW196917-4	S1827	Adrenergic.Receptor.antagonist	Betaxolol hydrochloride (Betoptic)	23	27
SW196917-5	S2091	Adrenergic.Receptor.antagonist	Betaxolol	23	30
SW197327-3	S2499	Adrenergic.Receptor.antagonist	Phenoxybenzamine HCl	24	29
SW222230-1	S2113	Adrenergic.Receptor.antagonist	Cisatracurium Besylate	24	8
SW196457-4	S2059	Adrenergic.Receptor.antagonist	Terazosin HCl	28	26
SW196570-5	S1387	Adrenergic.Receptor.antagonist	Naftopidil DiHCl	28	23
SW197240-3	S4123	Adrenergic.Receptor.antagonist	Timolol Maleate	28	12
SW219300-1	S2086	Adrenergic.Receptor.antagonist	Ivabradine HCl	28	30
SW219765-1	S1613	Adrenergic.Receptor.antagonist	Silodosin	28	26
SW219886-1	S1154	Aurora.Kinase	SNS-314 Mesylate	2	21
SW219458-1	S7065	Aurora.Kinase	MK-8745	6	2
SW220051-1	S2718	Aurora.Kinase	TAK-901	10	2
SW219462-1	S1107	Aurora.Kinase	Danusertib (PHA-739358)	13	15
SW219643-1	S1100	Aurora.Kinase	MLN8054	15	21
SW219491-1	S1171	Aurora.Kinase	CYC116	17	28
SW220282-1	S1103	Aurora.Kinase	ZM 447439	18	6
SW219480-1	S1519	Aurora.Kinase	CCT129202	19	6
SW219431-1	S1147	Aurora.Kinase	Barasertib (AZD1152-HQPA)	21	28
SW219731-1	S2719	Aurora.Kinase	AMG-900	22	28
SW220174-1	S2770	Aurora.Kinase	MK-5108 (VX-689)	23	6
SW212828-2	S1048	Aurora.Kinase	VX-680 (Tozasertib; MK-0457)	25	6
SW219481-1	S2744	Aurora.Kinase	CCT137690	25	21
SW219771-1	S1133	Aurora.Kinase	Alisertib (MLN8237)	27	6
SW219373-1	S1454	Aurora.Kinase	PHA-680632	29	15
SW220199-1	S1451	Aurora.Kinase	Aurora A Inhibitor I	29	27
SW219406-1	S1529	Aurora.Kinase	Hesperadin	30	15
SW219347-1	S2481	Calcium.Channel	Manidipine	2	29
SW219784-1	S1293	Calcium.Channel	Cilnidipine	2	29
SW219238-1	S1747	Calcium.Channel	Nimodipine	4	29
SW219299-1	S1885	Calcium.Channel	Felodipine	6	27
SW197582-2	S2017	Calcium.Channel	Benidipine HCl	14	4
SW197620-3	S1425	Calcium.Channel	Ranolazine 2HCl	15	14
SW219737-1	S2491	Calcium.Channel	Nitrendipine	15	5
SW219720-1	S2403	Calcium.Channel	Tetrandrine	18	12
SW196411-3	S2573	Calcium.Channel	Tetracaine HCl	20	26
SW219572-1	S2721	Calcium.Channel	Nilvadipine	21	20
SW220228-1	S1905	Calcium.Channel	Amlodipine	23	3
SW219840-1	S1994	Calcium.Channel	Lacidipine	27	5
SW196530-3	S2030	Calcium.Channel	Flunarizine 2HCl	28	4
SW220017-1	S1662	Calcium.Channel	Isradipine	28	23

SW220087-1	S2080	Calcium.Channel	Clevidipine Butyrate	28	16
SW219236-1	S3053	Calcium.Channel	Azelnidipine	29	12
SW219348-1	S2482	Calcium.Channel	Manidipine 2HCl	29	16
SW219166-1	S2014	CDK	BMS-265246	5	19
SW219356-1	S1572	CDK	BS-181 HCl	6	20
SW220101-1	S7440	CDK	LEE011	8	5
SW219405-1	S7158	CDK	LY2835219	10	19
SW219631-1	S7461	CDK	LDC000067	10	19
SW219878-1	S2742	CDK	PHA-767491	10	28
SW219949-1	S2751	CDK	Milciclib (PHA-848125)	10	28
SW219954-1	S2735	CDK	MK-8776 (SCH 900776)	10	21
SW220252-1	S1487	CDK	PHA-793887	10	28
SW219231-1	S2679	CDK	Flavopiridol HCl	11	28
SW219310-1	S8058	CDK	P276-00	11	28
SW219478-1	S1145	CDK	SNS-032 (BMS-387032)	11	28
SW219680-1	S2621	CDK	AZD5438	11	28
SW220016-1	S2768	CDK	Dinaciclib (SCH727965)	11	28
SW220191-1	S2688	CDK	R547	11	28
SW219950-1	S7320	CDK	TG003	12	6
SW220195-1	S1153	CDK	Roscovitine (Seliciclib;CYC202)	13	28
SW220039-1	S7114	CDK	NU6027	14	26
SW220083-1	S7509	CDK	ML167	14	8
SW219396-1	S1249	CDK	JNJ-7706621	19	19
SW220131-1	S1116	CDK	Palbociclib (PD-0332991) HCl	27	5
SW219609-1	S1524	CDK	AT7519	30	21
SW219668-1	S3043	COX	Rofecoxib	5	14
SW196700-3	S4078	COX	Mefenamic Acid	7	14
SW196989-3	S2577	COX	Phenacetin	7	26
SW197564-2	S4049	COX	Valdecoxib	7	10
SW219542-1	S4011	COX	Ampiroxicam	7	26
SW196831-3	S3023	COX	Bufexamac	14	13
SW219543-1	S2121	COX	Licofelone	14	16
SW196404-3	S1903	COX	Diclofenac Sodium	16	20
SW219723-1	S4295	COX	Meclofenamate Sodium	19	4
SW196982-3	S3200	COX	Triflusal	20	26
SW219769-1	S2903	COX	Lumiracoxib	20	26
SW196753-3	S1959	COX	Tolfenamic Acid	23	26
SW197105-3	S1626	COX	Naproxen	23	26
SW219862-1	S1713	COX	Piroxicam	23	20
SW196824-3	S2602	COX	Acemetacin	26	26
SW197312-3	S4051	COX	Nabumetone	26	30
SW219241-1	S2531	COX	Asaraldehyde	26	22
SW199611-3	S1261	COX	Celecoxib	27	20
SW196448-3	S2108	COX	Flunixin Meglumin	28	30
SW196784-3	S1645	COX	Ketoprofen	28	2
SW196785-3	S2040	COX	Nimesulide	28	11
SW197293-4	S1646	COX	Ketorolac	28	30
SW203738-2	S1638	COX	Ibuprofen	28	3

SW219801-1	S3008	COX	Zaltoprofen	28	3
SW220125-1	S2047	COX	Lornoxicam	28	14
SW197496-2	S1289	DNA.RNA.Synthesis	Carmofur	1	7
SW197705-2	S1192	DNA.RNA.Synthesis	Raltitrexed	1	7
SW199617-3	S1209	DNA.RNA.Synthesis	Fluorouracil (5-Fluoracil; 5-FU)	1	7
SW199090-2	S1305	DNA.RNA.Synthesis	Mercaptopurine (6-MP)	2	19
SW197258-4	S4288	DNA.RNA.Synthesis	Chloroambucil	4	28
SW219115-1	S4297	DNA.RNA.Synthesis	Mupirocin	5	11
SW220050-1	S2029	DNA.RNA.Synthesis	Uridine	6	25
SW220241-1	S1300	DNA.RNA.Synthesis	FT-207 (NSC 148958)	7	20
SW222225-1	S1166	DNA.RNA.Synthesis	Cisplatin	11	10
SW000346-2	S2554	DNA.RNA.Synthesis	Daphnetin	12	22
SW218080-2	S1218	DNA.RNA.Synthesis	Clofarabine	12	28
SW218086-2	S1213	DNA.RNA.Synthesis	Nelarabine	12	7
SW218146-2	S1229	DNA.RNA.Synthesis	Fludarabine Phosphate	12	7
SW222226-1	S1214	DNA.RNA.Synthesis	Bleomycin Sulfate	13	19
SW197746-4	S1199	DNA.RNA.Synthesis	Cladribine	16	28
SW199649-2	S1714	DNA.RNA.Synthesis	Gemcitabine	16	28
SW219867-1	S1221	DNA.RNA.Synthesis	Dacarbazine	19	30
SW220273-1	S1299	DNA.RNA.Synthesis	Floxuridine	19	7
SW219881-1	S1983	DNA.RNA.Synthesis	Adenine HCl	20	24
SW197177-4	S1302	DNA.RNA.Synthesis	Ifosfamide	21	14
SW219116-1	S2794	DNA.RNA.Synthesis	Sofosbuvir (PSI-7977; GS-7977)	26	11
SW219388-1	S1334	DNA.RNA.Synthesis	Flupirtine maleate	26	8
SW219151-1	S1224	DNA.RNA.Synthesis	Oxaliplatin	27	28
SW220171-1	S1156	DNA.RNA.Synthesis	Capecitabine	30	14
SW219272-1	S1143	EGFR	AG-490 (Tyrphostin B42)	1	13
SW219447-1	S1023	EGFR	Erlotinib HCl (OSI-744)	2	18
SW219475-1	S7284	EGFR	CO-1686 (AVL-301)	2	6
SW219315-1	S1173	EGFR	WZ4002	17	3
SW219395-1	S1179	EGFR	WZ8040	17	2
SW219714-1	S2728	EGFR	AG-1478 (Tyrphostin AG-1478)	17	27
SW199108-4	S1025	EGFR	Gefitinib (ZD1839)	18	4
SW219863-1	S7297	EGFR	AZD9291	18	9
SW219293-1	S2205	EGFR	OSI-420	19	17
SW219394-1	S1170	EGFR	WZ3146	25	15
SW219476-1	S7206	EGFR	CNX-2006	25	9
SW218184-2	S7039	EGFR	PD168393	27	5
SW219523-1	S8009	EGFR	AG-18	28	10
SW219267-1	S1392	EGFR	Pelitinib (EKB-569)	30	2
SW219698-1	S2922	EGFR	Icotinib	30	17
SW219372-1	S2818	HDAC	CI994 (Tacedinaline)	5	28
SW219374-1	S7292	HDAC	RG2833 (RGFP109)	5	28

SW219836-1	S8001	HDAC	Rocilinostat (ACY-1215)	5	15
SW219287-2	S8049	HDAC	Tubastatin A	7	30
SW219287-1	S2627	HDAC	Tubastatin A HCl	8	28
SW219401-1	S7229	HDAC	RGFP966	8	5
SW219449-1	S7324	HDAC	TMP269	12	9
SW219695-1	S7595	HDAC	Santacruzamate A (CAY10683)	12	20
SW219169-2	S1168	HDAC	Valproic acid sodium salt (Sodium valproate)	15	22
SW220150-1	S2012	HDAC	PCI-34051	16	30
SW219627-1	S1422	HDAC	Droxinostat	18	29
SW219738-1	S1484	HDAC	MC1568	20	20
SW199536-4	S1047	HDAC	Vorinostat (SAHA; MK0683)	22	15
SW218130-2	S1122	HDAC	Mocetinostat (MGCD0103)	22	28
SW218266-2	S1090	HDAC	PCI-24781 (Abexinostat)	22	15
SW219369-1	S1030	HDAC	Panobinostat (LBH589)	22	15
SW219379-1	S2779	HDAC	M344	22	15
SW219385-1	S1095	HDAC	LAQ824 (Dacinostat)	22	15
SW219429-1	S1515	HDAC	Pracinostat (SB939)	22	15
SW219445-1	S1085	HDAC	Belinostat (PXD101)	22	15
SW219469-1	S2170	HDAC	Givinostat (ITF2357)	22	15
SW219664-1	S1045	HDAC	Trichostatin A (TSA)	22	15
SW219667-1	S1053	HDAC	Entinostat (MS-275)	22	9
SW219675-1	S2693	HDAC	Resminostat	22	15
SW219772-1	S2244	HDAC	AR-42	22	15
SW219796-1	S1096	HDAC	Quisinostat (JNJ-26481585)	22	15
SW219824-1	S8043	HDAC	Scriptaid	22	15
SW219934-1	S1194	HDAC	CUDC-101	22	15
SW220090-1	S7473	HDAC	Nexturastat A	22	9
SW220304-1	S3020	HDAC	Romidepsin (FK228; Depsipeptide)	22	5
SW219199-1	S4125	HDAC	Sodium Phenylbutyrate	26	25
SW219084-1	S2239	HDAC	Tubacin	29	29
SW196835-3	S1847	Histamine.Receptor	Clemastine Fumarate	3	14
SW197416-3	S1358	Histamine.Receptor	Loratadine	3	5
SW196450-4	S2044	Histamine.Receptor	Cyproheptadine HCl	5	4
SW196380-2	S1845	Histamine.Receptor	Cimetidine	6	26
SW196927-3	S4139	Histamine.Receptor	Cyclizine 2HCl	7	20
SW196969-3	S3176	Histamine.Receptor	Betahistine 2HCl	7	11

SW197446-3	S3146	Histamine.Receptor	Tripelennamine HCl	7	1
SW199568-2	S3208	Histamine.Receptor	Fexofenadine HCl	7	3
SW219552-1	S4118	Histamine.Receptor	Histamine 2HCl	7	30
SW197471-3	S2552	Histamine.Receptor	Azelastine HCl	8	24
SW219888-1	S3186	Histamine.Receptor	Azatadine dimaleate	12	26
SW196707-3	S1382	Histamine.Receptor	Mianserin HCl	14	27
SW219889-1	S3052	Histamine.Receptor	Rupatadine Fumarate	14	29
SW196598-4	S1357	Histamine.Receptor	Lidocaine	15	25
SW196972-3	S1291	Histamine.Receptor	Cetirizine DiHCl	15	22
SW219424-1	S2905	Histamine.Receptor	JNJ-7777120	15	22
SW220161-1	S3037	Histamine.Receptor	Bepotastine Besilate	15	26
SW197397-2	S2078	Histamine.Receptor	Famotidine	16	26
SW197031-3	S2585	Histamine.Receptor	Brompheniramine hydrogen maleate	20	26
SW197771-3	S2494	Histamine.Receptor	Olopatadine HCl	20	25
SW196372-4	S1816	Histamine.Receptor	Chlorpheniramine Maleate	23	1
SW196887-4	S2024	Histamine.Receptor	Ketotifen Fumarate	23	11
SW197026-2	S2308	Histamine.Receptor	Hesperetin	23	11
SW197646-3	S1880	Histamine.Receptor	Roxatidine Acetate HCl	23	12
SW219167-1	S2813	Histamine.Receptor	Ciproxifan	23	12
SW196800-3	S1801	Histamine.Receptor	Ranitidine	26	13
SW219837-1	S4131	Histamine.Receptor	Levodropropizine	26	25
SW196508-3	S1890	Histamine.Receptor	Nizatidine	28	18
SW196577-3	S4026	Histamine.Receptor	Hydroxyzine 2HCl	28	16
SW197234-3	S4293	Histamine.Receptor	Promethazine HCl	28	5
SW197792-3	S4012	Histamine.Receptor	Desloratadine	28	3
SW219521-1	S3163	Histamine.Receptor	Benztropine mesylate	28	12
SW219706-1	S2065	Histamine.Receptor	Lafutidine	28	26
SW219489-1	S7122	HSP	XL888	9	15
SW220214-1	S8039	HSP	PU-H71	9	15
SW219742-1	S2639	HSP	SNX-2112 (PF-04928473)	13	15
SW219775-1	S1052	HSP	Elesclomol (STA-4783)	19	16
SW218138-2	S1163	HSP	AT13387	30	15
SW219302-1	S1141	HSP	17-AAG (Tanespimycin)	30	15
SW219303-1	S1142	HSP	17-DMAG (Alvespimycin) HCl	30	19
SW219319-1	S1069	HSP	AUY922 (NVP-AUY922)	30	15
SW219510-1	S2713	HSP	Geldanamycin	30	15
SW219606-1	S2685	HSP	KW-2478	30	15
SW219719-1	S7340	HSP	CH5138303	30	15
SW220092-1	S7458	HSP	VER-49009	30	19
SW220107-1	S7282	HSP	NMS-E973	30	15
SW220153-1	S2656	HSP	PF-04929113 (SNX-5422)	30	15
SW220170-1	S7459	HSP	VER-50589	30	15

SW220175-1	S7097	HSP	HSP990 (NVP-HSP990)	30	15
SW220186-1	S1175	HSP	BIIB021	30	15
SW220253-1	S1159	HSP	Ganetespib (STA-9090)	30	15
SW219203-1	S8004	JAK	ZM 39923 HCl	7	19
SW219757-1	S7036	JAK	XL019	8	6
SW219153-1	S2796	JAK	WP1066	10	27
SW219679-1	S2219	JAK	CYT387	10	19
SW219864-1	S8057	JAK	Pacritinib (SB1518)	10	21
SW220119-1	S1134	JAK	AT9283	11	21
SW220020-1	S7605	JAK	Filgotinib (GLPG0634)	15	28
SW220096-1	S2851	JAK	Baricitinib (LY3009104; INCB028050)	15	28
SW220207-1	S2902	JAK	S-Ruxolitinib (INCB018424)	15	8
SW220133-2	S2789	JAK	Tofacitinib (CP-690550;Tasocitinib)	16	1
SW218187-2	S2736	JAK	TG101348 (SAR302503)	17	6
SW219632-1	S2692	JAK	TG101209	17	2
SW219960-1	S2179	JAK	LY2784544	17	2
SW219437-1	S2686	JAK	NVP-BSK805 2HCl	18	2
SW219486-1	S2162	JAK	AZD1480	19	2
SW219623-1	S2214	JAK	AZ 960	19	27
SW220243-1	S2867	JAK	WHI-P154	19	8
SW219454-1	S2806	JAK	CEP-33779	25	5
SW222338-1	S1378	JAK	Ruxolitinib (INCB018424)	27	23
SW220133-1	S5001	JAK	Tofacitinib (CP-690550) Citrate	28	8
SW202561-3	S1008	MEK	Selumetinib (AZD6244)	9	23
SW218101-2	S1036	MEK	PD0325901	9	10
SW219366-1	S1102	MEK	U0126-EtOH	9	29
SW219605-1	S1568	MEK	PD318088	9	2
SW219691-1	S1475	MEK	Pimasertib (AS-703026)	9	10
SW219692-1	S2134	MEK	AZD8330	9	13
SW219839-1	S1066	MEK	SL-327	9	14
SW219910-1	S7007	MEK	MEK162 (ARRY-162; ARRY-438162)	9	29
SW220152-1	S2617	MEK	TAK-733	9	13
SW218089-2	S2673	MEK	Trametinib (GSK1120212)	13	10
SW218254-2	S1177	MEK	PD98059	13	10
SW219634-1	S1531	MEK	BIX 02189	16	23
SW197494-3	S2310	MEK	Honokiol	24	20
SW219635-1	S1530	MEK	BIX 02188	28	12
SW218136-2	S1089	MEK	Refametinib (RDEA119; Bay 86-9766)	30	5

SW219604-1	S1020	MEK	PD184352 (CI-1040)	30	23
SW219216-1	S7493	Microtubule.Associated	INH1	1	24
SW219847-1	S1148	Microtubule.Associated	Docetaxel	10	2
SW220268-1	S1364	Microtubule.Associated	Epothilone B (EPO906; Patupilone)	10	2
SW219685-1	S1165	Microtubule.Associated	ABT-751 (E7010)	11	27
SW220274-1	S1297	Microtubule.Associated	Epothilone A	11	2
SW219359-1	S7336	Microtubule.Associated	CW069	15	30
SW219257-1	S4269	Microtubule.Associated	Vinorelbine Tartrate	18	2
SW219306-1	S7494	Microtubule.Associated	INH6	19	24
SW102861-5	S2775	Microtubule.Associated	Nocodazole	21	27
SW219468-1	S1241	Microtubule.Associated	Vincristine	21	2
SW219940-1	S1248	Microtubule.Associated	Vinblastine	21	5
SW220198-1	S2195	Microtubule.Associated	CYT997 (Lexibulin)	21	5
SW219615-1	S4071	Microtubule.Associated	Griseofulvin	24	8
SW219830-1	S1150	Microtubule.Associated	Paclitaxel	29	2
SW219073-1	S1039	mTOR	Rapamycin (Sirolimus)	2	29
SW219138-1	S1044	mTOR	Temsirolimus (CCI-779; NSC 683864)	2	29
SW219218-1	S1120	mTOR	Everolimus (RAD001)	2	23
SW219472-1	S2696	mTOR	GDC-0980 (RG7422)	2	9
SW220210-1	S2811	mTOR	INK 128 (MLN0128)	2	17
SW220211-1	S2218	mTOR	PP242	2	17
SW222224-1	S1022	mTOR	Ridaforolimus (Deforolimus; MK-8669)	2	16
SW219676-1	S2238	mTOR	Palomid 529 (P529)	10	2
SW219762-1	S8050	mTOR	ETP-46464	27	20
SW220190-1	S2699	mTOR	CH5132799	27	17
SW220246-1	S2624	mTOR	OSI-027	27	23
SW219732-1	S2406	mTOR	Chrysophanic Acid	28	12
SW218287-2	S1555	mTOR	AZD8055	29	20
SW219487-1	S2661	mTOR	WYE-125132 (WYE-132)	29	12
SW219671-1	S1266	mTOR	WYE-354	29	16
SW219704-1	S2783	mTOR	AZD2014	29	27
SW219922-1	S2689	mTOR	WAY-600	29	27
SW220188-1	S1226	mTOR	KU-0063794	29	3
SW222245-1	S7091	mTOR	Zotarolimus(ABT-578)	29	16
SW219628-1	S2187	P450	Avasimibe	2	4
SW196888-4	S1353	P450	Ketoconazole	3	29
SW197561-4	S2046	P450	Pioglitazone HCl	4	11
SW196866-2	S2262	P450	Apigenin	6	21
SW219553-1	S2900	P450	Cobicistat (GS-9350)	8	29
SW219642-1	S1195	P450	TAK-700 (Orteronel)	15	30

SW101224-2	S2526	P450	Alizarin	20	22
SW197571-2	S1442	P450	Voriconazole	20	22
SW219043-1	S2555	P450	Clarithromycin	20	11
SW219329-1	S2394	P450	Naringenin	23	11
SW219546-1	S2921	P450	PF-4981517	24	22
SW219229-1	S2268	P450	Baicalein	27	10
SW219192-1	S4273	PARP	3-Aminobenzamide	7	25
SW219820-1	S1004	PARP	Veliparib (ABT-888)	7	14
SW218142-2	S1060	PARP	Olaparib (AZD2281; Ku-0059436)	8	7
SW219891-2	S2886	PARP	PJ34	8	20
SW219192-2	S1132	PARP	INO-1001	12	12
SW219655-1	S7048	PARP	BMN 673	12	28
SW218112-2	S1087	PARP	Iniparib (BSI-201)	13	10
SW219891-1	S7300	PARP	PJ34 HCl	15	2
SW219733-1	S8038	PARP	UPF 1069	20	9
SW219936-1	S2178	PARP	AG-14361	21	28
SW219802-1	S7029	PARP	AZD2461	23	7
SW219544-1	S1098	PARP	Rucaparib (AG-014699;PF-01367338)	26	7
SW220067-1	S7438	PARP	ME0328	28	20
SW197603-2	S1512	PDE	Tadalafil	1	22
SW219717-1	S1550	PDE	Pimobendan	5	2
SW219816-1	S7224	PDE	Deltarasin	10	20
SW196433-3	S2320	PDE	Luteolin	11	8
SW199053-2	S1294	PDE	Cilostazol	15	14
SW222234-1	S2687	PDE	PF-2545920	18	28
SW197648-2	S3172	PDE	Anagrelide HCl	20	9
SW197737-2	S2515	PDE	Vardenafil HCl Trihydrate	20	14
SW199664-3	S1431	PDE	Sildenafil Citrate	20	14
SW219217-1	S4019	PDE	Avanafil	20	16
SW219244-1	S2127	PDE	S- (+)-Rolipram	20	17
SW219280-1	S1455	PDE	Cilomilast	20	18
SW197542-3	S1929	PDE	Irsogladine	23	3
SW219125-1	S2312	PDE	Icariin	24	11
SW196583-4	S1430	PDE	Rolipram	26	22
SW196679-3	S1504	PDE	Dyphylline	26	8
SW219741-1	S2620	PDE	GSK256066	26	24
SW219856-1	S8034	PDE	Apremilast (CC-10004)	26	22
SW220196-1	S2131	PDE	Roflumilast	26	11
SW199286-2	S1673	PDE	Aminophylline	28	23
SW219311-1	S2671	PI3K	AS-252424	1	20
SW202556-4	S1065	PI3K	GDC-0941	2	12
SW218117-2	S1038	PI3K	PI-103	2	12
SW219415-1	S1360	PI3K	GSK1059615	2	27
SW219812-1	S1118	PI3K	XL147	2	19
SW220128-1	S2814	PI3K	BYL719	2	6
SW220216-1	S2767	PI3K	3-Methyladenine	5	26
SW219482-1	S7018	PI3K	CZC24832	7	18
SW219650-1	S8002	PI3K	GSK2636771	8	30

SW219158-1	S2749	PI3K	BGT226 (NVP-BGT226)	10	27
SW219506-1	S1205	PI3K	PIK-75	11	28
SW113275-2	S2682	PI3K	CAY10505	12	13
SW219297-1	S1352	PI3K	TG100-115	12	1
SW219823-1	S2226	PI3K	CAL-101 (Idelalisib; GS-1101)	12	18
SW219919-1	S2681	PI3K	AS-604850	12	10
SW220182-1	S1072	PI3K	ZSTK474	13	4
SW220212-1	S2227	PI3K	PIK-294	13	4
SW217688-2	S1105	PI3K	LY294002	14	30
SW218196-2	S2636	PI3K	A66	14	3
SW218249-2	S1169	PI3K	TGX-221	14	16
SW219525-1	S2870	PI3K	TG100713	14	9
SW219822-1	S7028	PI3K	IPI-145 (INK1197)	14	17
SW219502-1	S2658	PI3K	GSK2126458 (GSK458)	17	21
SW219871-1	S2759	PI3K	CUDC-907	17	20
SW218129-2	S1219	PI3K	YM201636	18	27
SW219556-1	S2207	PI3K	PIK-293	24	22
SW219873-1	S7356	PI3K	HS-173	25	27
SW219187-1	S1462	PI3K	AZD6482	27	3
SW219245-1	S1489	PI3K	PIK-93	27	5
SW220201-1	S7016	PI3K	VS-5584 (SB2343)	27	7
SW218114-2	S1523	PI3K	SAR245409 (XL765)	28	20
SW218149-2	S2247	PI3K	BKM120 (NVP-BKM120; Buparlisib)	29	27
SW219545-1	S2758	PI3K	Wortmannin	29	4
SW219392-1	S1386	Proteasome	Nafamostat Mesylate	12	12
SW219683-1	S7462	Proteasome	PI-1840	12	9
SW199665-2	S3017	Proteasome	Aspirin	15	26
SW208077-3	S1013	Proteasome	Bortezomib (PS-341)	17	19
SW218090-2	S2853	Proteasome	Carfilzomib (PR-171)	17	19
SW219161-1	S1157	Proteasome	CEP-18770 (Delanzomib)	17	19
SW219743-1	S2180	Proteasome	MLN2238	17	19
SW219744-1	S2181	Proteasome	MLN9708	17	19
SW219780-1	S2619	Proteasome	MG-132	17	19
SW220115-1	S7172	Proteasome	ONX-0914 (PR-957)	17	19
SW220116-1	S7049	Proteasome	Oprozomib (ONX 0912)	17	19
SW197284-3	S2101	Proteasome	Gabexate Mesylate	28	25
SW219493-1	S3046	RAAS	Azilsartan	1	12
SW219848-1	S1793	RAAS	Ramipril	1	1
SW220041-1	S2037	RAAS	Candesartan Cilexetil	4	4
SW197658-2	S1894	RAAS	Valsartan	6	28
SW197676-3	S1738	RAAS	Telmisartan	6	26
SW199393-2	S2581	RAAS	Quinapril HCl	7	12
SW220093-1	S2109	RAAS	Imidapril HCl	7	26

SW222231-1	S2199	RAAS	Aliskiren Hemifumarate	7	22
SW197672-2	S1657	RAAS	Enalaprilat Dihydrate	13	22
SW220029-1	S2664	RAAS	Clinofibrate	19	11
SW199641-2	S1359	RAAS	Losartan Potassium (DuP 753)	20	26
SW199650-2	S1604	RAAS	Olmesartan Medoxomil	23	11
SW219263-1	S2079	RAAS	Moexipril HCl	23	26
SW220127-1	S7098	RAAS	PD123319	23	1
SW197591-3	S1284	RAAS	Benazepril HCl	24	22
SW198780-2	S1941	RAAS	Enalapril Maleate	28	26
SW199612-2	S1578	RAAS	Candesartan	28	11
SW219164-1	S2051	RAAS	Captopril	28	11
SW219383-1	S2081	RAAS	Cilazapril Monohydrate	28	26
SW219451-1	S2099	RAAS	Temocapril HCl	28	18
SW219494-1	S3057	RAAS	Azilsartan Medoxomil	28	3
SW212797-2	S2872	RAF	GW5074	6	3
SW218185-2	S2720	RAF	ZM 336372	6	12
SW219204-1	S1104	RAF	GDC-0879	9	18
SW219448-1	S2746	RAF	AZ 628	9	21
SW219503-1	S2807	RAF	Dabrafenib (GSK2118436)	9	21
SW219895-1	S7291	RAF	TAK-632	9	7
SW220064-1	S7108	RAF	LGX818	9	4
SW202562-3	S1040	RAF	Sorafenib Tosylate	10	7
SW202562-4	S7397	RAF	Sorafenib	10	29
SW219923-1	S2161	RAF	RAF265 (CHIR-265)	11	16
SW218095-2	S1267	RAF	Vemurafenib (PLX4032; RG7204)	27	4
SW218119-2	S1152	RAF	PLX-4720	29	24
SW220229-1	S2220	RAF	SB590885	30	9
SW220279-1	S1398	Reverse.Transcriptase	Stavudine (d4T)	1	12
SW220193-1	S2914	Reverse.Transcriptase	Dapivirine (TMC120)	2	2
SW198799-2	S2579	Reverse.Transcriptase	Zidovudine	7	13
SW220232-1	S7303	Reverse.Transcriptase	Rilpivirine	10	9
SW219933-1	S1718	Reverse.Transcriptase	Adefovir Dipivoxil	13	19
SW220151-1	S1400	Reverse.Transcriptase	Tenofovir Disoproxil Fumarate	16	7
SW197364-4	S1719	Reverse.Transcriptase	Zalcitabine	23	26
SW197614-3	S1706	Reverse.Transcriptase	Lamivudine	23	26
SW220172-1	S1704	Reverse.Transcriptase	Emtricitabine	23	14
SW219570-1	S3080	Reverse.Transcriptase	Etravirine (TMC125)	27	7
SW197569-2	S1742	Reverse.Transcriptase	Nevirapine	28	26
SW198619-2	S1702	Reverse.Transcriptase	Didanosine	28	8
SW219101-1	S4016	Sodium.Channel	Ouabain	2	21
SW219113-1	S4290	Sodium.Channel	Digoxin	2	21

SW196688-3	S4080	Sodium.Channel	Triamterene	7	20
SW220143-1	S2524	Sodium.Channel	Phenytoin sodium	7	11
SW196719-3	S4023	Sodium.Channel	Procaine HCl	12	30
SW219770-1	S1256	Sodium.Channel	Rufinamide	15	11
SW220114-1	S2118	Sodium.Channel	Ibutilide Fumarate	15	14
SW196333-5	S2560	Sodium.Channel	Amiloride hydrochloride dihydrate	20	11
SW197468-3	S1391	Sodium.Channel	Oxcarbazepine	20	14
SW203757-2	S2525	Sodium.Channel	Phenytoin	20	1
SW197486-3	S3024	Sodium.Channel	Lamotrigine	21	30
SW196805-4	S1614	Sodium.Channel	Riluzole	23	9
SW196878-3	S4294	Sodium.Channel	Procainamide HCl	23	23
SW197338-3	S1828	Sodium.Channel	Proparacaine HCl	23	22
SW220141-1	S1693	Sodium.Channel	Carbamazepine	23	11
SW196964-3	S2500	Sodium.Channel	Propafenone HCl	24	24
SW198832-2	S3064	Sodium.Channel	Ambroxol HCl	24	14
SW196475-3	S4038	Sodium.Channel	Dibucaine HCl	28	12
SW220277-1	S2785	Sodium.Channel	A-803467	28	4
SW219924-1	S7518	Topoisomerase	Voreloxin (SNS-595)	4	19
SW203763-2	S1342	Topoisomerase	Genistein	6	28
SW196414-3	S1288	Topoisomerase	Camptothecin	10	19
SW196745-6	S1889	Topoisomerase	Mitoxantrone	10	9
SW219079-1	S1393	Topoisomerase	Pirarubicin	10	19
SW219806-1	S7261	Topoisomerase	Beta-Lapachone	10	27
SW219421-1	S1228	Topoisomerase	Idarubicin HCl	11	4
SW219441-1	S1208	Topoisomerase	Doxorubicin (Adriamycin)	11	20
SW219442-1	S1223	Topoisomerase	Epirubicin HCl	11	9
SW197554-3	S1465	Topoisomerase	Moxifloxacin HCl	13	26
SW219048-1	S1225	Topoisomerase	Etoposide	13	19
SW220163-1	S1198	Topoisomerase	Irinotecan	13	19
SW196774-3	S3181	Topoisomerase	Flumequine	15	11
SW197608-4	S4119	Topoisomerase	Pefloxacin Mesylate Dihydrate	16	26
SW197790-4	S2217	Topoisomerase	Irinotecan HCl Trihydrate	16	28
SW219056-1	S3603	Topoisomerase	Betulinic acid	18	30
SW197557-5	S1231	Topoisomerase	Topotecan HCl	19	19
SW219948-1	S4908	Topoisomerase	SN-38	19	28
SW220315-1	S2423	Topoisomerase	10-Hydroxycamptothecin	19	19

SW219946-1	S1367	Topoisomerase	Amonafide	27	19
SW219896-1	S1084	VEGFR	Brivanib (BMS-540215)	1	17
SW218092-2	S1046	VEGFR	Vandetanib (ZD6474)	3	2
SW218116-2	S1003	VEGFR	Linifanib (ABT-869)	10	19
SW220084-1	S7258	VEGFR	SKLB1002	12	20
SW219259-1	S1164	VEGFR	Lenvatinib (E7080)	13	23
SW219897-1	S1138	VEGFR	Brivanib Alaninate (BMS-582664)	13	1
SW198937-2	S1101	VEGFR	Vatalanib (PTK787) 2HCl	15	29
SW218301-2	S1010	VEGFR	Nintedanib (BIBF 1120)	18	8
SW219500-1	S2896	VEGFR	ZM 323881 HCl	19	4
SW219791-1	S2845	VEGFR	Semaxanib (SU5416)	19	8
SW218300-2	S1032	VEGFR	Motesanib Diphosphate (AMG-706)	22	8
SW220296-1	S2221	VEGFR	Apatinib	24	4
SW219944-1	S2842	VEGFR	SAR131675	26	17
SW219943-1	S2897	VEGFR	ZM 306416	27	17
SW219464-1	S1005	VEGFR.KIT.PDGFR	Axitinib	1	21
SW218156-2	S1363	VEGFR.KIT.PDGFR	Ki8751	5	21
SW218097-2	S1178	VEGFR.KIT.PDGFR	Regorafenib (BAY 73-4506)	11	29
SW218082-2	S1035	VEGFR.KIT.PDGFR	Pazopanib HCl (GW786034 HCl)	13	28
SW219261-1	S1017	VEGFR.KIT.PDGFR	Cediranib (AZD2171)	18	18
SW219787-1	S1018	VEGFR.KIT.PDGFR	Dovitinib (TKI-258; CHIR-258)	18	6
SW218082-3	S3012	VEGFR.KIT.PDGFR	Pazopanib	19	28
SW219794-1	S1220	VEGFR.KIT.PDGFR	OSI-930	19	4
SW219407-1	S1042	VEGFR.KIT.PDGFR	Sunitinib Malate	25	2
SW219787-2	S2769	VEGFR.KIT.PDGFR	Dovitinib (TKI-258) Dilactic Acid	25	21
SW219262-1	S1557	VEGFR.KIT.PDGFR	KRN 633	28	8
SW220176-1	S2231	VEGFR.KIT.PDGFR	Telatinib	29	5
SW219364-1	S1207	VEGFR.KIT.PDGFR	Tivozanib (AV-951)	30	17

Supplementary Table 3: SNF-Euclidean APC hypergeometric test p-values.
Hypergeometric test p-values for Selleck target classes in SNF-Euclidean APC clusters. Bonferroni-corrected alpha = 0.0006

Class	Cluster Number	-log10 p-value	Significant?
P450	1	4.25605576	TRUE
ATM.ATR	1	5.04517835	TRUE
Hist.Receptor	4	2.2897748	FALSE
COX	6	1.91982167	FALSE
RAAS	6	2.13377808	FALSE
Hist.Receptor	6	3.43010914	TRUE
AdrR.agonist	6	4.02855491	TRUE
Hist.Receptor	9	1.95941317	FALSE
Hist.Receptor	10	1.75917031	FALSE
RAAS	10	3.45658465	TRUE
Reverse.Transcriptase	10	4.49051096	TRUE
Hist.Receptor	13	2.69927303	FALSE
AChR.antagonist	13	3.0056789	FALSE
PDE	14	2.68768419	FALSE
K.Channel	14	3.62607173	TRUE
Adr.Receptor.antagonist	15	2.5148078	FALSE
PI3K	17	3.14300227	FALSE
COX	19	3.3097259	TRUE
HDAC	20	1.79545626	FALSE
K.Channel	20	3.29865781	TRUE
DNA.RNA.Synthesis	25	6.20147175	TRUE
AChR.antagonist	26	2.84683694	FALSE
RAAS	26	3.21292655	FALSE
COX	26	4.02855491	TRUE
PI3K	27	3.05651353	FALSE
JAK	39	3.71566364	TRUE
CDK	42	8.86025664	TRUE
PDE	43	3.48380466	TRUE
DNA.RNA.Synthesis	45	3.1743779	FALSE
Aurora.Kinase	45	3.63343507	TRUE
Reverse.Transcriptase	45	4.11305913	TRUE
VEGFR.KIT.PDGFR	45	5.79439169	TRUE
MEK	46	4.4173404	TRUE
RAF	48	5.61784122	TRUE
HSP	48	15.6575773	TRUE

PLK	49	4.77132139	TRUE
HDAC	49	15.6575773	TRUE
Hist.Receptor	64	2.05864879	FALSE
AChR.antagonist	64	2.35315313	FALSE
Adr.Receptor.agonist	64	2.40247918	FALSE
COX	64	2.40247918	FALSE
Dopamine.Receptor.antagonist	65	4.92012054	TRUE
Topoisomerase	66	3.41531571	TRUE
PI3K	67	2.13035048	FALSE
Topoisomerase	67	2.7625374	FALSE
Bcl-2	67	4.0269807	TRUE
Hist.Receptor	68	1.95941317	FALSE
COX	68	2.30034797	FALSE
VEGFR	71	4.97183704	TRUE
HMG-CoA.Reductase	73	5.67048662	TRUE
Src	74	6.64992842	TRUE
Epigenetic.Reader.Domain	76	14.3111371	TRUE
Hist.Receptor	77	2.13035048	FALSE
Adr.Receptor.antagonist	77	2.4761153	FALSE
AChR.antagonist	80	2.88696492	FALSE
Hist.Receptor	81	2.42654657	FALSE
ER.PR_antagonist	81	4.78604926	TRUE
RAF	82	5.49803799	TRUE
MEK	82	7.51565297	TRUE
HDAC	83	9.41983968	TRUE
AChR.antagonist	86	2.21944195	FALSE
Adr.Receptor.antagonist	86	2.26828361	FALSE
5HTR.agonist	86	4.58709819	TRUE
p38.MAPK	87	4.82954492	TRUE
STAT	88	4.17883559	TRUE
ABL	88	4.8251888	TRUE
Topoisomerase	88	5.04851238	TRUE
RAF	90	4.14395889	TRUE
ATPase	90	6.73816904	TRUE
ABL	91	4.37914804	TRUE
Adr.Receptor.antagonist	93	3.50631673	TRUE
ER.PR_agonist	97	4.67738131	TRUE
EGFR.ERBB	98	4.44183346	TRUE
DNA.RNA.Synthesis	99	3.4727626	TRUE
PI3K	100	3.20179911	FALSE
Wnt.beta-catenin	100	3.70337698	TRUE

IGFR	100	3.85458471	TRUE
Ca.Channel	100	4.37616146	TRUE
mTOR	100	14.7784985	TRUE
Microtubule.Associated	101	7.23326999	TRUE
MEK	102	9.30487308	TRUE
Topoisomerase	103	2.97361122	FALSE
Sodium.Channel	103	3.04111994	FALSE
IkB.IKK	103	4.24542125	TRUE
JAK	103	4.3690592	TRUE
CDK	103	5.68016436	TRUE
Proteasome	103	15.9545898	TRUE
MET	105	4.9666167	TRUE
PDGFR	105	5.29514851	TRUE
PARP	106	4.70583723	TRUE
PDE	107	2.84249965	FALSE
Histone.Methyltransferase	108	4.59476586	TRUE
Aurora.Kinase	109	5.95513653	TRUE
p38.MAPK	110	5.06645713	TRUE
JAK	111	3.48380466	TRUE
EGFR	111	3.87285091	TRUE
MET.VEGFR	111	5.21210644	TRUE
ABL	111	6.22196478	TRUE
DNA.RNA.Synthesis	115	4.31493643	TRUE
RAAS	117	2.86452526	FALSE
Sodium.Channel	117	2.99566445	FALSE
DNA.RNA.Synthesis	117	3.9787859	TRUE
Kinesin	118	6.76986956	TRUE
Topoisomerase	119	5.95198865	TRUE
PLK	122	4.83449691	TRUE
STAT	122	5.0753188	TRUE
Microtubule.Associated	122	9.48944986	TRUE
EGFR	123	4.39394247	TRUE
PI3K	123	4.88317226	TRUE
mTOR	123	10.0294242	TRUE
RAAS	124	2.77899116	FALSE
5HTR.antagonist	124	2.84249965	FALSE
5HTR.antagonist	127	2.48838075	FALSE
Adr.Receptor.agonist	127	3.3097259	TRUE
GluR.antagonist	127	5.7801431	TRUE

Supplementary Table 4: Target class enrichment in APC clusters. APC clusters that are enriched with a single class from the Selleck library and the NPFs that are associated with those clusters.

Class	Cluster number	Number of NPFs	NPF IDs
HSP	48	0	
HDAC	49	3	SW218953 through SW218955
Epigenetic.Reader.Domain	76	0	
mTOR	100	1	SW218859
Proteasome	103	1	SW218864
mTOR	123	0	

Supplementary Table 5: Cluster #49 members. NPFs and Selleck compounds that are present in the APC cluster #49, which is enriched in HDAC inhibitors.

Name	Class	Compound	MW
SW218140-2	PLK	Volasertib (BI 6727)	618.81
SW218130-2	HDAC	Mocetinostat (MGCD0103)	396.44
SW199536-4	HDAC	Vorinostat (SAHA, MK0683)	264.3
SW218954-1	NPF	RLUS-2173D	NA
SW218953-1	NPF	RLUS-2173C	NA
SW218266-2	HDAC	PCI-24781 (Abexinostat)	397.42
SW218187-2	JAK	TG101348 (SAR302503)	524.68
SW219379-1	HDAC	M344	307.39
SW219369-1	HDAC	Panobinostat (LBH589)	349.43
SW219316-1	PLK	Ro3280	543.61
SW218955-1	NPF	RLUS-2173E	NA
SW219469-1	HDAC	Givinostat (ITF2357)	475.97
SW219445-1	HDAC	Belinostat (PXD101)	318.35
SW219429-1	HDAC	Pracinostat (SB939)	358.48
SW219385-1	HDAC	LAQ824 (Dacinostat)	379.46
SW219796-1	HDAC	Quisinostat (JNJ-26481585)	394.48
SW219772-1	HDAC	AR-42	312.36
SW219675-1	HDAC	Resminostat	349.4
SW219664-1	HDAC	Trichostatin A (TSA)	302.4
SW220090-1	HDAC	Nexturastat A	341.4
SW219934-1	HDAC	CUDC-101	434.49
SW219824-1	HDAC	Scriptaid	326.35

Supplementary Table 6: Differential expression of top 20 genes in the Nanostring metabolism panel.

Differential expression is shown as log2 fold change. P-values are Benjamini-Yekutieli adjusted. *Rawdata-Nanostringmetabolismpanel included as separate attachment.

	Differential Expression- Top20											
	**Log2 fold**	**std error (**	**Lower con**	**Upper con**	**Linear fold**	**Lower con**	**Upper con**	**P-value**	**BY.p.value**	**method**	**Gene.sets**	**probe.ID**
THBS1-mRNA	-0.248	0.0123	-0.272	-0.224	0.842	0.828	0.856	3.76E-08	0.000132	loglinear	Myc, PI3K	NM_003246.2:3465
CD63-mRNA	-0.124	0.016	-0.155	-0.0921	0.918	0.898	0.938	5.72E-05	0.0663	loglinear	Lysosomal Degradation	NM_001780.4:350
SCD-mRNA	0.212	0.0276	0.158	0.266	1.16	1.12	1.2	5.83E-05	0.0663	loglinear	AMPK, Fatty Acid Synthesis	NM_005063.4:2025
PRKAG2-mRNA	-0.125	0.0168	-0.158	-0.0917	0.917	0.897	0.938	7.55E-05	0.0663	loglinear	AMPK, Autophagy, Fatty Acid Oxidation, Mitocho	NM_016203.3:1895
SQSTM1-mRNA	-0.0961	0.0144	-0.124	-0.0679	0.936	0.917	0.954	0.000155	0.103	loglinear	Cytokine & Chemokine Signaling	NM_003900.3:1445
CCND1-mRNA	-0.135	0.0206	-0.175	-0.0947	0.911	0.886	0.936	0.000177	0.103	loglinear	AMPK, Cell Cycle, Cytokine & Chemokine Signalin	NM_053056.2:690
HMOX1-mRNA	-0.363	0.0572	-0.475	-0.251	0.777	0.719	0.84	0.000222	0.111	loglinear	Cytokine & Chemokine Signaling, KEAP1NRF2 Pat	NM_002133.2:781
GAPDH-mRNA	-0.0641	0.0108	-0.0853	-0.043	0.957	0.943	0.971	0.000344	0.151	loglinear	Glycolysis	NM_001256799.1:386
CTPS1-mRNA	-0.141	0.0261	-0.192	-0.0901	0.907	0.875	0.939	0.000633	0.247	loglinear	Nucleotide Synthesis	NM_001301237.1:580
MYC-mRNA	-0.117	0.0242	-0.164	-0.0692	0.922	0.892	0.953	0.00132	0.464	loglinear	Cell Cycle, Cytokine & Chemokine Signaling, MAP	NM_002467.3:1610
CAD-mRNA	-0.135	0.0292	-0.193	-0.078	0.911	0.875	0.947	0.00169	0.499	loglinear	Amino Acid Synthesis, Myc, Nucleotide Synthesis	NM_004341.3:2380
ASNS-mRNA	-0.159	0.0345	-0.226	-0.091	0.896	0.855	0.939	0.00177	0.499	loglinear	Amino Acid Synthesis, Glutamine Metabolism	NM_183356.2:1644
ZNF43-mRNA	-0.235	0.0516	-0.336	-0.133	0.85	0.792	0.912	0.00189	0.499	loglinear	Transcriptional Regulation	NM_003423.2:3835
NT5E-mRNA	-0.134	0.0305	-0.194	-0.0743	0.911	0.874	0.95	0.00231	0.499	loglinear	Nucleotide Synthesis, Vitamin & Cofactor Metab	NM_002526.2:1214
ALDOA-mRNA	-0.0631	0.0145	-0.0915	-0.0347	0.957	0.939	0.976	0.00241	0.499	loglinear	Glycolysis, Pentose Phosphate Pathway	NM_184041.2:1455
TXNRD1-mRNA	-0.102	0.0236	-0.148	-0.0561	0.932	0.902	0.962	0.00247	0.499	loglinear	p53 Pathway	NM_001093771.1:1009
RPLP0-mRNA	-0.0481	0.0111	-0.0699	-0.0263	0.967	0.953	0.982	0.00251	0.499	loglinear	Cytokine & Chemokine Signaling	NM_001002.3:250
COX4I1-mRNA	-0.0529	0.0125	-0.0774	-0.0285	0.964	0.948	0.98	0.00282	0.499	loglinear	Mitochondrial Respiration, p53 Pathway	NM_001318797.1:50
WDR45-mRNA	-0.2	0.0473	-0.293	-0.108	0.87	0.816	0.928	0.00283	0.499	loglinear	Autophagy	NM_007075.3:1390
ATF4-mRNA	-0.0885	0.0209	-0.129	-0.0476	0.941	0.914	0.968	0.00284	0.499	loglinear	MAPK, PI3K, Transcriptional Regulation	NM_001675.2:1151

References

1 Jensen, P. R., Gontang, E., Mafnas, C., Mincer, T. J. & Fenical, W. Culturable marine actinomycete diversity from tropical Pacific Ocean sediments. *Environ. Microbiol.* **7**, 1039-1048, doi:10.1111/j.1462-2920.2005.00785.x (2005).

2 Hong, K. *et al.* Actinomycetes for marine drug discovery isolated from mangrove soils and plants in China. *Mar. Drugs* **7**, 24-44, doi:10.3390/md7010024 (2009).

3 Woehrmann, M. H. *et al.* Large-scale cytological profiling for functional analysis of bioactive compounds. *Mol. Biosyst.* **9**, 2604-2617, doi:10.1039/c3mb70245f (2013).

4 Potts, M. B. *et al.* Using functional signature ontology (FUSION) to identify mechanisms of action for natural products. *Sci. Signal.* **6**, ra90, doi:10.1126/scisignal.2004657 (2013).

5 Wang, B. *et al.* Similarity network fusion for aggregating data types on a genomic scale. *Nat. Methods.* **11**, 333-337, doi:10.1038/nmeth.2810 (2014).

6 Kim, H. S. *et al.* Systematic identification of molecular subtype-selective vulnerabilities in non-small-cell lung cancer. *Cell* **155**, 552-566, doi:10.1016/j.cell.2013.09.041 (2013).

7 Kurita, K. L., Glassey, E. & Linington, R. G. Integration of high-content screening and untargeted metabolomics for comprehensive functional annotation of natural product libraries. *Proc. Natl. Acad. Sci. USA* **112**, 11999-12004, doi:10.1073/pnas.1507743112 (2015).
